# Intron size minimisation in teleosts

**DOI:** 10.1186/s12864-022-08760-w

**Published:** 2022-09-01

**Authors:** Lars Martin Jakt, Arseny Dubin, Steinar Daae Johansen

**Affiliations:** 1 Faculty for bioscience and aquaculture, Nord University, Universitetsalléen 11, Bodoe, 8026 Norway; 2 Currently at: Parental Investment and Immune Dynamics, GEOMAR Helmholtz Centre for Ocean Research, Düsternbrookerweg 20, Kiel, D-24105 Germany

**Keywords:** Genome size, Intron length, Teleost, Vertebrate evolution

## Abstract

**Background:**

Spliceosomal introns are parts of primary transcripts that are removed by RNA splicing. Although introns apparently do not contribute to the function of the mature transcript, in vertebrates they comprise the majority of the transcribed region increasing the metabolic cost of transcription. The persistence of long introns across evolutionary time suggests functional roles that can offset this metabolic cost. The teleosts comprise one of the largest vertebrate clades. They have unusually compact and variable genome sizes and provide a suitable system for analysing intron evolution.

**Results:**

We have analysed intron lengths in 172 vertebrate genomes and show that teleost intron lengths are relatively short, highly variable and bimodally distributed. Introns that were long in teleosts were also found to be long in mammals and were more likely to be found in regulatory genes and to contain conserved sequences. Our results argue that intron length has decreased in parallel in a non-random manner throughout teleost evolution and represent a deviation from the ancestral state.

**Conclusion:**

Our observations indicate an accelerated rate of intron size evolution in the teleosts and that teleost introns can be divided into two classes by their length. Teleost intron sizes have evolved primarily as a side-effect of genome size evolution and small genomes are dominated by short introns (<256 base pairs). However, a non-random subset of introns has resisted this process across the teleosts and these are more likely have functional roles in all vertebrate clades.

**Supplementary Information:**

The online version contains supplementary material available at (10.1186/s12864-022-08760-w).

## Background

The presence of spliceosomal introns within both coding and non-coding transcripts is a fundamental property of eukaryotes that separates them from eubacteria and archaea [1, 2]. It is well-known that spliceosomal introns can be alternatively spliced increasing the functional complexity of the transcriptome [3], but the presence of introns by itself also has important roles in translation efficiency and non-sense mediated decay [4]. Introns can also encode regulatory RNAs such as microRNAs, snoRNAs or lncRNAs [5, 6]. A very interesting example is the processing of functional snoRNAs from introns in pseudogenes [7, 8]. Nevertheless, it is unclear as to what proportion of introns have functional roles.

Conservation of intron positions within orthologous transcripts has been observed across all eukaryotic kingdoms [9, 10]. Interestingly the extent of this conservation is not well correlated with phylogenetic distance with more conservation found between human and *Arabidopsis* than between human and *Drosophila* [9]. Remarkably, around 80% of intron positions in orthologous transcripts are conserved between human and the sea anemone *Nematostella vectensis* [11]. In contrast, 76% of introns in the chordate *Oikopleura* are unique with only 17% found at ancestral positions [12]. Hence, although introns (and their positions) are generally well conserved, it appears that intron loss and gain has been accelerated in specific clades.

The presence of splice sites in a transcript has a range of beneficial effects leading to more effective and better regulated translation (eg. intron mediated enhancement [13] and nonsense-mediated decay [4]), however, the effect of intron length is unclear. In *Drosophila* species it seems that long and very short introns are selected against and thus likely to be detrimental to evolutionary success [14]. Similarly, house-keeping genes and other highly expressed genes tend to have shorter introns, perhaps simply reflecting the metabolic cost of excessive transcription [15].

Nevertheless, long introns are maintained in many species, and intron length is well correlated with genome size [16] (Fig. S1), suggesting that long introns in general may arise as a side-effect rather than through the accumulation of function. However, evolutionary conservation of intron size across diverse clades would argue for function since it is clear that introns can both grow and shrink in size. This is supported by the tendency for developmental regulators to contain long introns [12] and the observation that mammalian introns containing conserved sequences are longer [17] than those that do not. Interestingly, the enrichment for long introns in developmental regulators is reversed in both axolotl [18] and lungfish [19], where introns are generally shorter in such genes. Both lungfish and axolotl have giant genomes (ca. 43 and 32 Gb respectively) and contain introns that have expanded roughly in line with their genomes. The depletion of long introns in developmental genes in these species suggests that excessive intron length can also be selected against for reasons other than metabolic cost.

In mammals, 3 to 6% of transcribed sequence is exonic, while the vast majority is composed of introns. Consistent with their smaller genome sizes, teleosts have much shorter introns with the transcribed regions being around 10-20% exonic (Fig. S1). During the annotation of the genome of *Lophius piscatorius*[20] we noticed that the distribution of log transformed intron sizes was clearly bimodal, with a very large number of short introns (shorter than 256 base pairs, bp) forming a sharp peak separated from a broad peak of larger introns indicating that introns in *Lophius piscatorius* can be divided into two separate classes (data not shown).

In order to determine whether a bimodal intron size distribution is a general and or specific property of teleosts we extended our analysis to include the majority of vertebrate genomes present in the Ensembl database [21]. Our observations show that a) teleost introns do in general have bimodal size distributions, b) teleost intron length predicts intron length in other vertebrates, c) long introns are associated with specific biological functions and d) are more likely to contain sequence conserved across the vertebrate clade. We also show that intron size variation in teleosts is likely to have arisen as a result of the parallel evolution towards small introns throughout the clade, and that a similar process of intron size reduction is likely to have occurred specifically in the Aves, but not other Sauria clades. Both of these processes are likely to have resulted from an evolutionary pressure for smaller genomes or as a side-effect of directional neutral evolution.

## Results

### Teleost introns can be divided into long and short introns

#### Bimodal distributions across the vertebrates

**Fig. 1:**
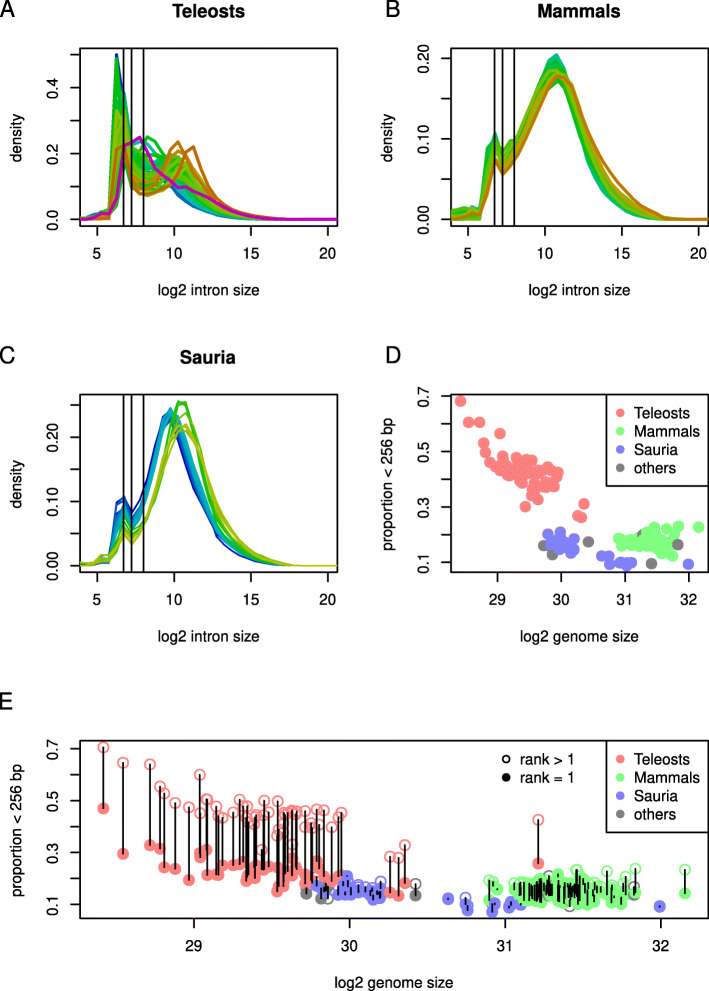
Vertebrate intron size. **A**-**C**, distributions of log_2_ intron size in teleosts (A), mammals (B) and Sauria (C). Colour of lines indicate log genome size from blue (small) to purple (large); x-axis, log_2_ intron size, y-axis density. Distributions for teleosts are much more varied than for mammals or Sauria, but are generally bimodal with an antimode at 2^8^ bases (indicated by vertical line). The distributions of mammals and Sauria also suggest bimodality with a peak of shorter introns at 105 bp. Distributions for Sauria separate naturally into two groups of different genome size ranges; these correspond to Aves (birds) and non-Aves species, with Aves species (blues) having both smaller genomes and introns. **D**, log_2_ genome size (x-axis) plotted against the fraction of introns shorter than 2^8^ base pairs (y-axis). Colour indicates taxonomic membership as indicated. **E**, points plotted as in (D), but divided into first introns (filled circles, rank = 1) and other introns (rank > 1). Pairs of points for each species are joined by vertical lines. First introns are much less likely to be smaller than 2^8^ bp in teleosts

We made use of the Ensembl database to obtain gene coordinates from 172 different vertebrate species and calculated the distributions of intron sizes for all canonical transcripts from each species (Fig. 1 and S2). Teleost distributions were very variable, but the majority of species had bimodal distributions with an antimode (trough) at approximately 256 (2^8^) bp separating short introns peaking at around 76 bp from a more broadly distributed population of longer introns.

Notably, this included species both from the dominant Percomorphaceae clade (42 of 54 teleost species analysed) and others (eg. *Danio rerio*, *Denticeps clupeoides*). Interestingly, this typical distribution was not observed in *Hucho hucho* and its most closely related non-salmonid species *Esox lucius*, as well as some other species (eg. *Clupea harengus*, *Electrophorus electricus*). In smaller genomes the short intron peak (< 256bp) was almost completely dominant obscuring the much smaller peak of long introns (Fig. 1A, S2, additional file 1).

A preliminary analysis of the species in Ensembl version 104, that includes many additional salmonids appears to support the notion of a clade specific distribution for the salmonids and *Esox lucius* that doees not have the typical teleost antimode (additional file 2).

We also observed bimodality in the distributions of genomes belonging to both the Mammalia and Sauria clades with a minor peak (76 bp) of introns shorter than 150 bp (Fig. 1 B, C, S2 and additional file 1, 2) as previously reported [22].

In addition the intron size distributions in the Sauria could be clearly divided into two groups corresponding to Aves and non-Aves species. Aves genomes contained shorter introns consistent with their generally smaller genome sizes (Fig. 1C).

The bimodal distribution observed in the teleosts was not seen in other aquatic vertebrates. The most closely related non-teleost analysed here, *Lepisosteus oculatus*, had a distribution more similar to mammals and birds then to teleosts. We also observed a very clear bimodal distribution in the jawless vertebrate *Eptatretus burgeri* (hagfish), but this distribution was different to that observed in teleosts (additional file 1). We thus infer that the bimodality observed across the teleosts is teleost specific.

In general, the vast majority of intron sizes reported were larger than 75 bp and it is likely that this represents a lower bound for the size of a vertebrate intron. The proportion of very small (<32 bp) introns varied across the vertebrates in a manner unrelated to phylogeny. Genomes with higher proportions of such introns were usually from highly fragmented assemblies with short scaffold lengths (Fig. S3). Hence, intron sizes reported to be smaller than this lower-bound are likely to represent annotation errors.

#### The proportion of long introns reflects genome size

Since the short intron peak seemed to dominate the small teleost genomes, we plotted the proportion of introns shorter than 256 bp against genome size to determine whether this represents a general property (Fig. 1 D). Indeed a strong correlation between genome size and the fraction of short (< 256 bp) introns was readily observed across the teleosts. This suggests that the relationship observed between median intron and genome size [16] does not simply reflect differences in the size of all introns, but may also arise from a binary change in intron size status.

#### First introns are more likely to be long in teleosts

The first intron of a transcript is generally both longer and more likely to contain regulatory elements [23–25] and we asked whether first introns were more likely to belong to the longer class. Indeed, first introns were markedly more likely to be longer than 256 bp across all teleosts (Fig. 1E and S2). Although first introns were in general longer across the vertebrates we did not observe a major partition around 256 bp for non-teleost species and the differences in distributions were greater for teleost species (Fig. S4). Interestingly, the fractions of long first introns in teleosts were comparable to the fractions of long introns in Aves species with similar genome sizes (Fig. 1E).

### Evolution of vertebrate intron sizes

#### An intron orthology

To investigate how intron size has evolved across the vertebrates we constructed an intron orthology by pairwise alignments of orthologous genes. To simplify the orthology we concentrated on gene families that mostly have only a single orthologue in each species. Since we are primarily interested in intron evolution in teleosts we made use of an orthology based on alignments to *Danio rerio* transcripts as these are derived from the most complete teleost genome annotation. This intron orthology contains a total of 63,068 introns from 5,752 genes. Although we did not find orthologues in all species for all *Danio rerio* introns, the majority of introns were represented by at least 156 species and 90% (154/172) of species had orthologues to at least 47,609 *Danio rerio* introns (Fig. S5).

#### Long teleost introns predict long mammalian introns

**Fig. 2:**
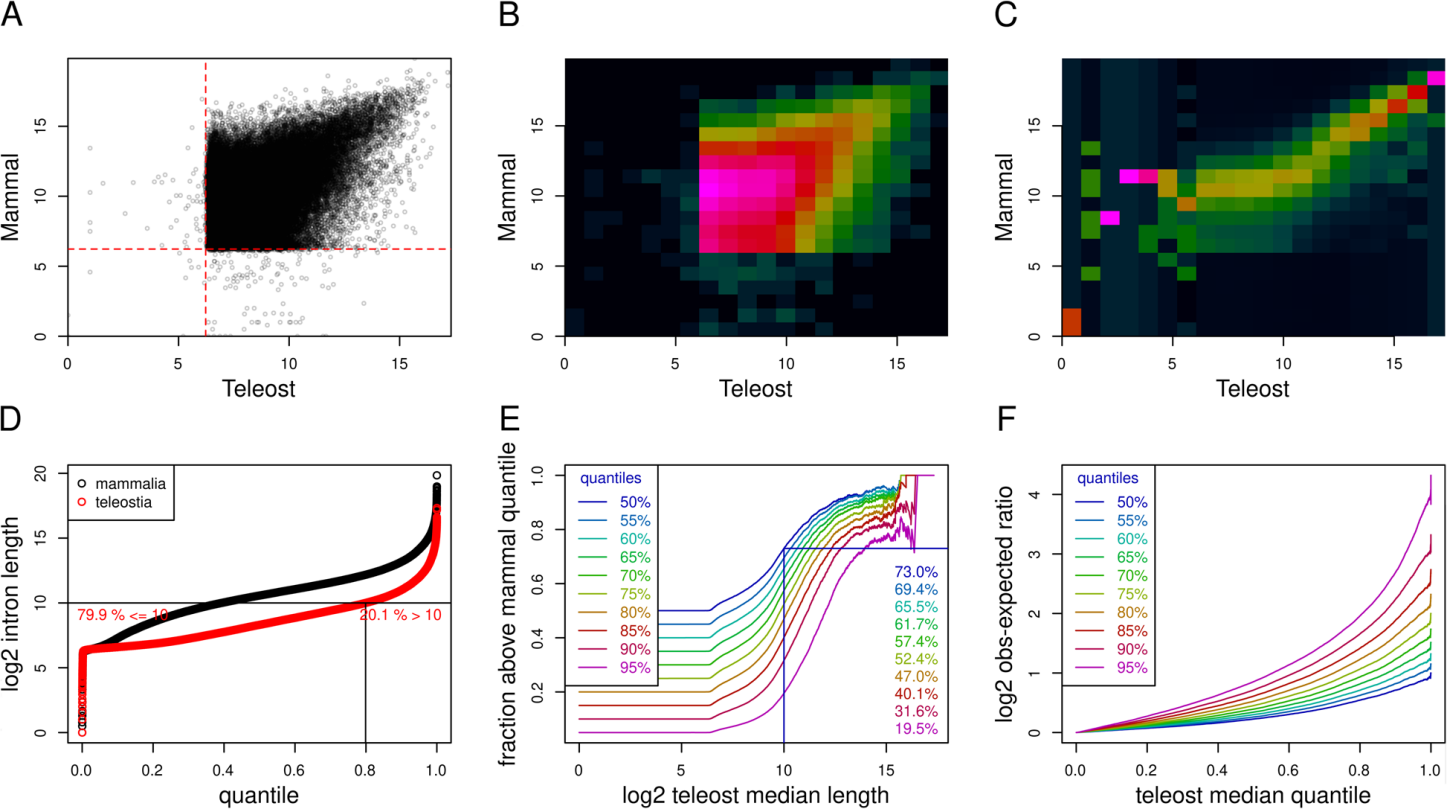
Teleost intron size predicts mammalian intron size. **A**, median log_2_ intron sizes in teleosts (x-axis) plotted against those in mammals (y-axis) indicate a strong correlation in size for longer introns. The vast majority of introns in both teleosts and mammals are longer than 75 bases (indicated by the dashed red lines); however we observe indicated sizes shorter than these in both clades that are likely to represent annotation artefacts rather than real short introns. **B**, and **C**, two-dimensional histograms for points in A. Colours range from dark blue (low) to bright pink (high) and indicate either the log transformed counts of the number of points within the indicated ranges (B) or, counts scaled by column to have means and standard deviations of 0 and 1 respectively. **D**, quantile plot of log_2_ median teleost and mammal intron sizes. 20% of introns have a median length of 10^2^ (1024) bp or more in teleosts. **E**, proportion of introns having a median length above the indicated quantiles in mammals plotted against log_2_ teleost median length. Of the 20% of introns whose median teleost length are longer than 2^10^ bp, 73 and 20% have mammal lengths longer than 50 and 95% of mammalian introns (see right inset). **F**, excess of long introns in mammals (log_2_ observed / expected ratios) for sets indicated in E

We first asked to what extent intron size in teleosts predicts intron size in other clades by comparing the median lengths of orthologue sets in mammals and teleosts (Fig. 2 A-C). The median (across species) intron size in teleosts and mammals were highly correlated for introns whose teleost median lengths were above 1000 (2^10^) bp (Fig. 2 A-C). This represents 20% of the full intron set (Fig. 2 D); 73% of the mammalian orthologues of these introns had median intron lengths longer than the 50^th^ percentile (i.e. median of median lengths) and 20% were longer than the 95^th^ percentile (Fig. 2E). In general, the longer the teleost median length, the stronger the prediction (observed / expected) of long intron size in mammals (Fig. 2F).

These observations suggest that introns which have remained long across the majority of teleosts do not represent a random sub-set, and that these contain functional sequences which are conserved in mammalian species.

#### Intron size mutual information

**Fig. 3:**
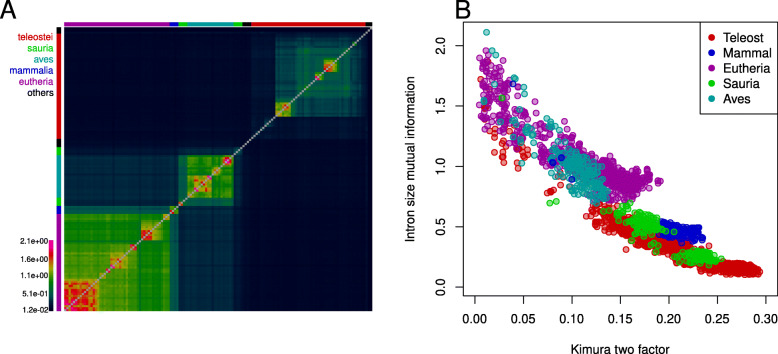
Conservation of intron sizes across the vertebrates. **A**, Mutual information between intron size for all pairs of species having less than 2.5% of introns shorter than 2^5^ bp. Species are arranged by taxonomic order obtained from a neigbhour-joining tree based on exon alignments (Fig. S6). High mutual information is seen within taxonomic groupings, but is much lower for teleosts than mammals and the Sauria. **B**, Kimura-two factor distances (x-axis, based on exon alignments) plotted against mutual information (y-axis) for all within clade species pairs. Mutual information is generally lower within the teleosts than mammals for equalivent exon derived distances indicating a higher rate of change in intron size within the teleosts

To determine to what extent intron size correlates between species we calculated the mutual information in intron size between pairs of species. For this analysis we excluded genomes where the fraction of predicted introns shorter than 32 bp was larger than 2.5% (Fig. S3) since they obscured otherwise clear general trends. We observed clearly significant levels of mutual information for all pairs of species indicating some conservation of intron sizes across all vertebrates (significance judged by Monte-Carlo sampling of 10,000 random permutations). The amount of mutual information between species was clearly a function of taxonomic grouping with high mutual information observed within both the mammalian and Sauria clades (Fig. 3A).

Interestingly, the extent of mutual information between teleost species was much lower than that observed for mammal and sauria pairs. This is suggestive of an increased rate of intron size evolution in teleosts. To ensure that this was not merely a function of the evolutionary distances of the represented teleost species we also calculated pairwise Kimura-two factor distances for all species based on pairwise alignments of 472 (gene) orthologues. The mutual information for a given Kimura distance was lowest for pairs of teleost species and highest for pairs of mammalian species (Fig. 3B). This argues for an increased rate of intron size evolution in teleosts.

#### Intron size reduction in teleostei and aves

**Fig. 4:**
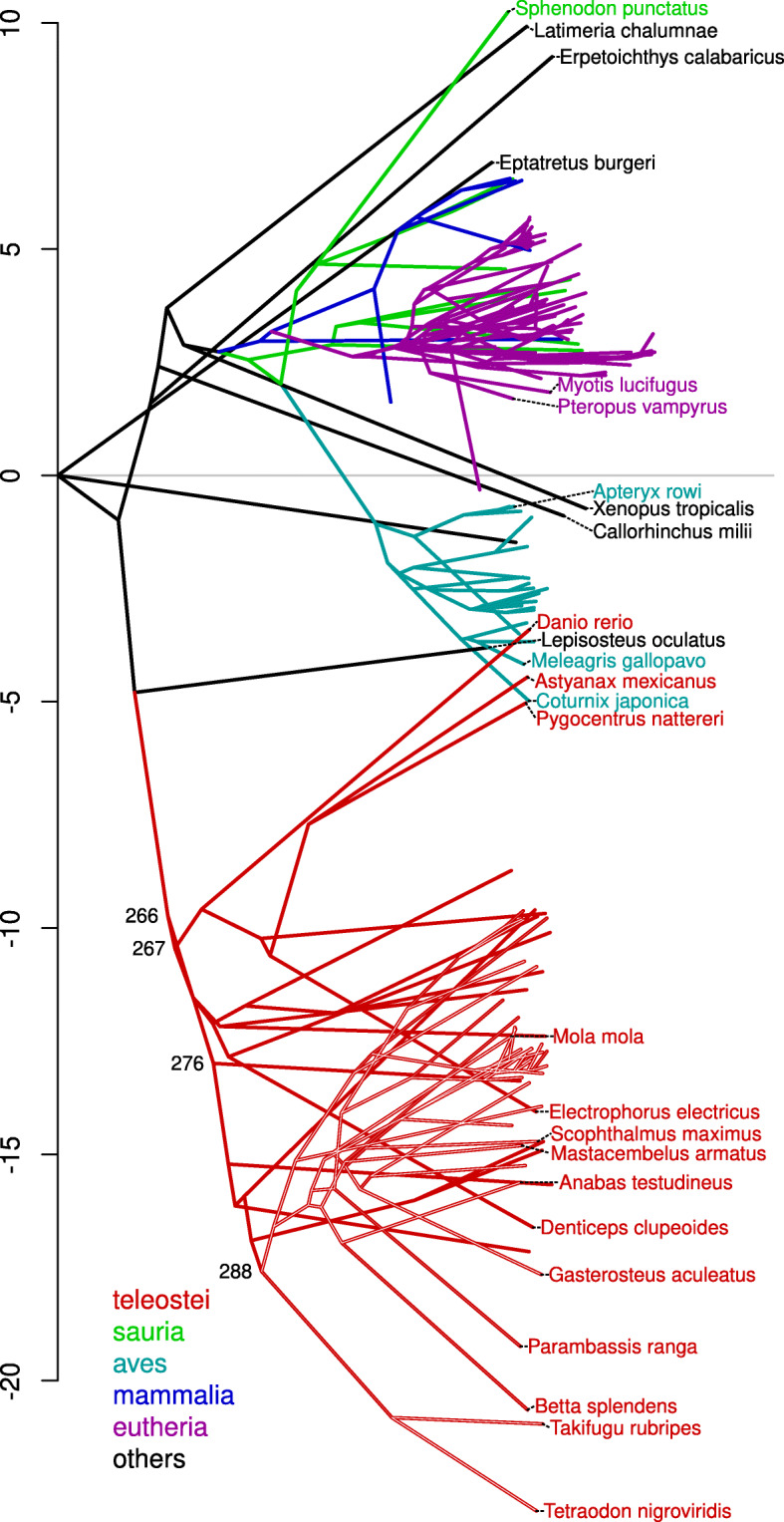
Evolution of intron size. Neighbour joining tree derived from exon alignments with the y-position of nodes determined by changes in intron size inferred by Sankoff maximum parsimony. Positions along the x-axis indicate cumulative Kimura two-factor distance from ancestors, positions along the y-axis indicate cumulative changes in mean (10×log_2_) intron size from ancestors. Colours of lines indicate taxonomic clade membership of leaf and inferred ancestral nodes. Intron sizes in teleosts (red) and aves (cyan) appear to have decreased dramatically from their respective ancestral states

Teleost genomes and intron sizes are in general smaller than those of other vertebrates, suggesting a decrease in genome size that is specific to teleosts (i.e. a synapomorphy). However, it is also possible that the common vertebrate ancestor possessed a small genome with small introns and that this expanded in size in non-teleost clades. To evaluate the likelihood of these alternatives we used Sankoff maximum parsimony on a neighbour-joining tree (Fig. S6) created from pairwise Kimura two factor distances (as used in Fig. 3) to infer the sizes of introns in ancestors. This inference (Fig. 4) is consistent with intron sizes in the common vertebrate ancestor having been more similar to extant mammalian and Sauria species, and that intron sizes in the teleost clade decreased drastically both before and after divergence from *Lepisosteus oculatus*.

Three teleost species have unusually large genomes and corresponding introns; *Danio rerio*, *Astyanax mexicanus* and *Pygocentrus nattereri*. All three are derived from a branch diverging early in teleost evolution and *A. mexicanus* and *P. nattereri* have an exclusive common ancestor. Nevertheless, all these three species appear to have experienced independent increases in intron sizes since their common ancestors suggesting an ongoing process of genome size expansion presumably through the accumulation of transposable elements (TEs) [26].

Perhaps most striking is the apparent intron size reduction in avian species; avians are members of the Sauria clade, but compared to other Sauria species they have much smaller intron sizes. The majority of this intron size reduction appears to have occurred in the evolution of their common ancestor with smaller changes after divergence which is consistent with an inference of small genomes in the extinct pterosaur lineage [27] and in extant alligators [28]. Other Sauria species appear to have experienced intron size expansion rather than contraction with *Sphenodon punctatus* having one of the largest genomes in the data set.

We also asked to what extent intron size minimisation is an ongoing process by inspecting the difference in intron size between extant species and their most recent inferred ancestor. Most species appear to have experienced recent increases in intron size (Fig. S7). However, the teleost species with the smallest genomes had shorter introns than their most recent ancestors, suggesting that introns in these species are likely to become shorter in their descendants.

Importantly, there is relatively little conservation in intron size within the teleosts suggesting an ongoing process of intron size diversification. Although the majority of decrease in intron size seems to have occurred prior to the most recent common teleost ancestor, most teleost species have introns considerably smaller than this. This suggests a subsequent parallel decrease in intron size across the teleost clade.

#### Size dependent intron size evolution

**Fig. 5:**
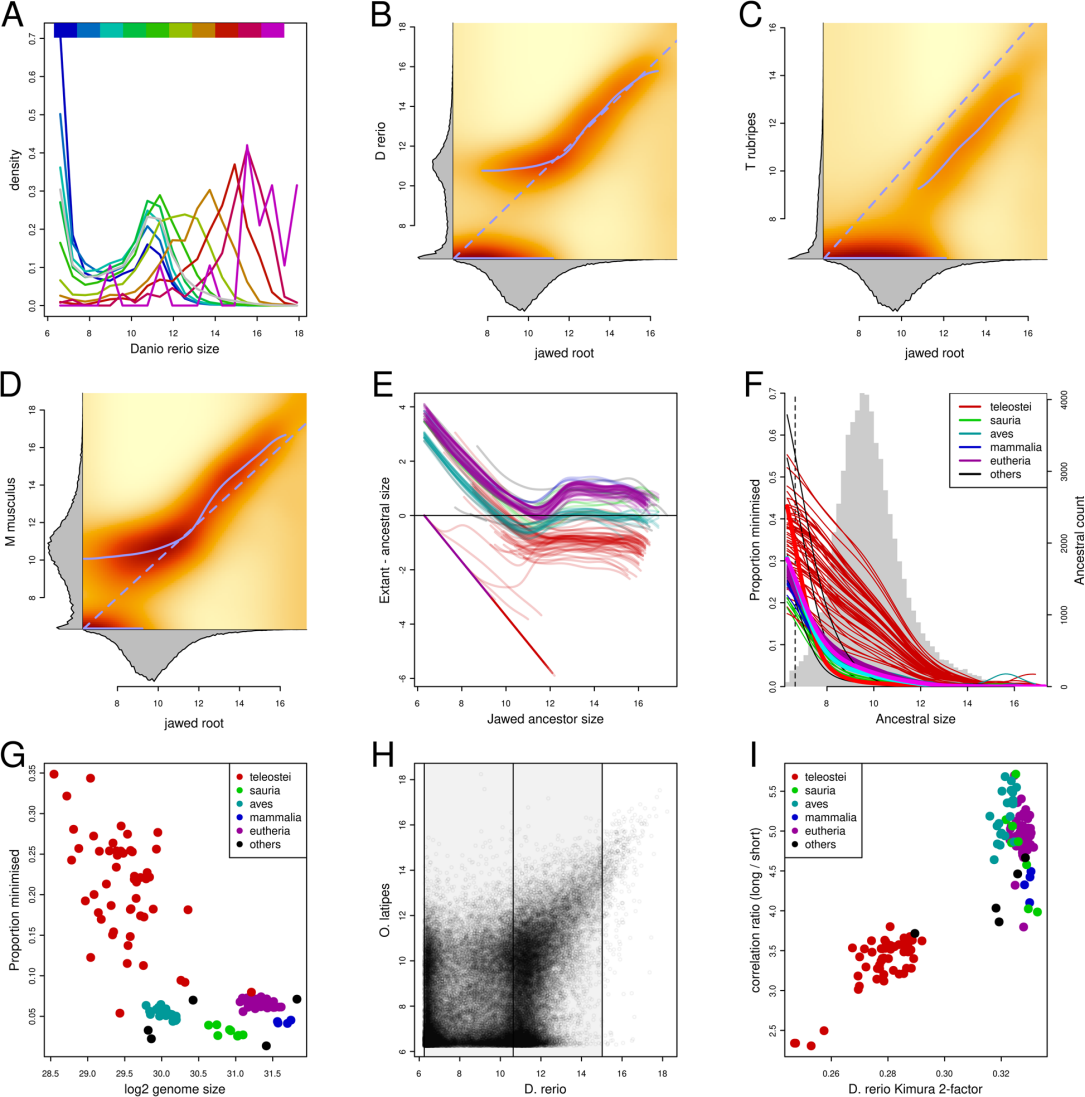
Size dependent intron size evolution. **A**) Distributions of intron sizes in *D. rerio* discretised by their inferred size in the joint vertebrate ancestor (JVA). Colours indicate the size in the JVA as shown at the top. Introns that are short in the JVA have bimodal distributions in *D. rerio* with peaks at 76 and ca. 2000 bp. **B**-**D**) Heatmap representations of the joint distributions of intron sizes in the JVA and *D. rerio* (B), *T. rubripes* (C) and *M. musculus* (D). Solid blue lines indicate mode lines (distribution peaks); dashed lines indicate the diagonal. The marginal distributions are shown in the margins. The joint distributions were scaled by column and blurred to facilitate the extraction of the mode lines. **E**) Mode lines of the change in intron size from the JVA to extant vertebrates. Lines are equivalent to those extracted in B-C, but rotated to emphasise change in size. Colours indicate taxons as in (F). **F**) Proportion of introns minimised (between 76 and 100 bp long) as a function of size in the JVA. Background histogram gives the inferred distribution of intron sizes in the JVA (right axis). Thick solid lines give the proportions of introns minimised in the clade specific common ancestors. Note that only the teleost ancestor (red) is significantly different from the extant species indicating extensive heterogeneity in the sets of minimised introns in teleosts. **G**) Proportion of minimised introns (75-100 bp) in vertebrates plotted against genome size. Colours indicate taxons. **H**) Intron sizes in *O. latipes* plotted against *D. rerio*. The division into short and long sets of introns used to calculate correlation coefficents for (I) are indicated by the shaded regions. **I**) Introns in *D. rerio* lying between 76 bp and the 99th percentile were divided into two sets of equal size ranges (Fig. 5H) and the correlation in sizes between these introns and their orthologues in other species calculated. The plot shows the ratios of correlation coefficients for introns long and short in *D. rerio* plotted against the Kimura two-factor distances between the respective species pairs. Long introns are generally more correlated than short ones and this effect is stronger in more evolutionary diverged pairs of species. All intron sizes are log2 transformed

We next considered how intron size has evolved in different vertebrate clades by comparing extant intron sizes with those inferred for the ancestor of the jawed vertebrates (JVA, node 259, Fig. S6). Introns inferred as shorter than ca. 2000 bp in the JVA were bimodally distributed in *Danio rerio*, with two peaks at around 76 and 2000 bp (Fig. 5A,B). The shorter of these corresponds to what is likely to be the minimal size of vertebrate introns. Introns longer than 2000 bp in the JVA had distributions in *Danio rerio* with peaks corresponding to their JVA size. In general, the shorter the JVA length the larger the proportion of minimised introns in *Danio rerio*.

In contrast, introns shorter than 2000 bp in the JVA were predominantly minimised in *Takifugu rubripes*, with longer introns having broad distributions peaking at less than half of the JVA size (Fig. 5C). The pattern in *Mus musculus* was more similar to that of *Danio rerio* with a bimodal distribution for short introns (Fig. 5D).

We calculated the distributions of intron size change between the JVA and the extant species as a function of JVA size and traced the mode lines (i.e. peaks of distributions discretised by their size in the JVA; Fig. 5E). Dual modes of size change were observed for short introns across the vertebrates, with the lower mode at the likely vertebrate minimal intron size (76 bp). The range of JVA sizes for which bimodality and minimisation was observed was much larger in the teleosts, with peaks of minimised introns observed for introns up to 2000 bp long in the JVA. The second mode line was biphasic with introns shorter than 2000 bp in the JVA having a mode associated with a constant extant size of ca. 2000 bp (upper solid lines in Fig. 5B-D). Introns longer than this in the JVA had extant modes correlating with JVA size; these were longest in the mammals and shortest in the teleosts.

Introns which were inferred to be short in the JVA were more likely to be minimised across the vertebrates. However, the rate of minimisation was much higher in teleosts, especially for introns inferred to be long in the JVA (Fig. 5F). The overall rate of minimisation was fairly constant in the birds and mammals, but varied extensively with genome size in the teleosts (Fig. 5F,G).

We note that the distribution of inferred intron size in the JVA does not have a peak of minimally sized introns (Fig. 5B-D). This is unlike all extant distributions and is likely to reflect the limitations of the ancestral state inference. The JVA is likely to have had a peak of minimal introns, but it may not be possible to infer these using maximum parsimony if their state has not been maintained in a sufficient number of extant species.

These observations suggest that introns have evolved differently depending on their size with longer introns being more conserved in size. If this is true, then we should see less conservation in extant intron length for short introns compared to long ones. To assess this we first plotted the intron size in *Oryzias latipes* against *Danio rerio* (Fig. 5H). Although *Danio rerio* has one of the larger genomes with a smaller proportion of minimised introns it nevertheless has a large number of minimal introns that are not shared with *Oryzias latipes*, suggesting that minimisation has occurred in parallel in the two lineages giving rise to these species. In general, there appears to be very little correlation in intron size for short introns whereas longer introns appear well correlated between the two species. This is consistent with a scenario where short introns evolve quickly, with relatively rapid shuffling between minimised and non-minimal states, whereas longer introns are less likely to change. This is also consistent with the relative lack of minimised introns inferred in the JVA.

To test whether this pattern is general we compared the correlation coefficients between introns short and long in *Danio rerio* and their orthologues in other vertebrates (Fig. 5I). The correlation coefficients for long introns were between two to six times larger than those for short introns. This difference increased with phylogenetic distance as would be expected if the evolution of long introns is more constrained than that of short ones (for identically sized intron sets the correlation will be the same; as evolution proceeds, the loss of correlation proceeds faster in the shorter set and the difference increases). Similar results were obtained when using a range of species to define the short and long intron sets.

Taken together these observations suggest that short and long introns have evolved in different manners and that long intron size is more likely to represent ancestral size than short introns. This difference in mode of evolution may have resulted from the presence of function in long introns or as a side-effect of the molecular mechanisms underlying intron size evolution.

### Functional association

#### Genes with long introns are enriched for specific biological functions

Retention of intron sequence or length across the vertebrates suggests the presence of some regulatory function. If this is the case, then genes which do not contain any long introns should be less likely to be highly regulated and this ought to be reflected in their biological and molecular function. To test this we selected nested sets of genes containing at least one intron that is long (>*T*_*i*_ where *T* was a series of thresholds) across the teleosts and performed gene ontology (GO) enrichment analysis using human annotation. Sets of genes containing long introns were strongly enriched for specific biological process (BP), molecular function (MF) and cellular compartment (CC) terms. The enriched BP and MF terms were related to system development, cell signalling and transcriptional regulation (data not shown). This is consistent with genes containing long introns being more regulated than other genes and previous observations of short introns in highly expressed and house-keeping genes [15, 29].

Since this gene selection could have been biased towards genes containing large numbers of introns we also selected genes by randomly selecting introns from the intron orthology and performed the same analyses. Although such genes were strongly enriched for specific GO terms as would be expected (due to preferential selection of genes with many introns), these terms did not overlap with those found by selecting genes with long introns.

**Fig. 6:**
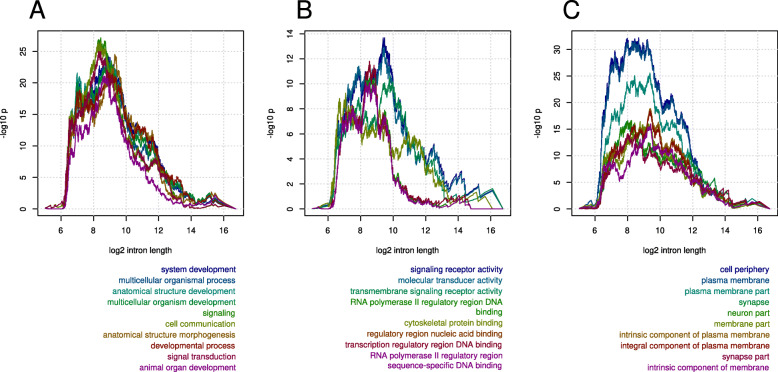
Depletion of gene ontology (GO) categories in genes without long introns. Depletion probabilities for gene sets with short exons only calculated by the hypergeometric distribution. Genes were ordered by the minimum teleost length of their longest introns and the probability of observing the observed, or fewer, number of members of the indicated GO categories calculated for all nested sub-sets. Analyses were calculated separately for **A**) biological process (BP), **B**) molecular function (MF) and **C**) cellular compartment (CC). The probabilites (y-axis, -log_10_p) are shown plotted against the size (log_2_ transformed) of the largest intron of the nested set (x-axis)

To further investigate the functional associations we ordered genes by their longest minimal teleost intron length, determined the number of genes belonging to enriched GO terms for all nested sets (from short to long) and calculated depletion statistics (Fig. 6, Fig. S8-10, additional file 3). For most terms the strongest depletion probability occurred between 2^8^-2^9^ bp (Fig. S8-10, additional file 3). This position roughly corresponds with the observed antimode observed in teleosts lengths and is consistent with distinct functional roles for short and long introns in teleosts.

#### Long introns are enriched for conserved sequences

If conservation of intron length is related to the retention of regulatory sequences then this should be reflected in an increase in sequence conservation. To test this we aligned sets of intron sequences from *Danio rerio* chosen by their lengths across the teleosts to their orthologues in other species. Because regulatory function may be present in several regions across the intron we performed local alignments and extracted all non-overlapping alignments to *Danio rerio* sequences. High scores were more easily found from alignments of introns that were consistently long across the teleosts (Fig. S11, additional file 4) than for variable length introns (Fig. S12, additional file 5). These included alignments across the full length of *Danio rerio* introns, as well as single and multiple windows of conservation. Although high scoring alignments were most common to other teleosts, a number of *Danio rerio* regions could be aligned across the vertebrate clade.

**Fig. 7:**
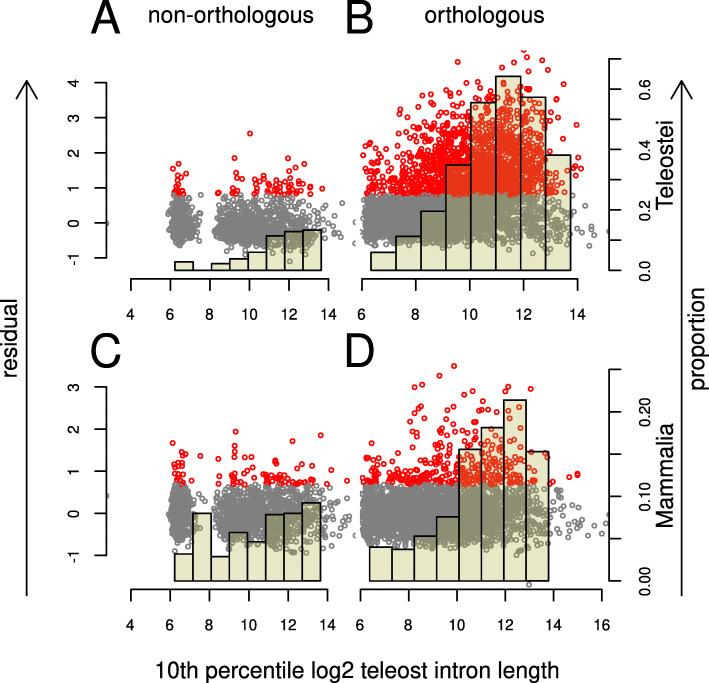
Conservation of intron sequences. Intron orthologue sets were chosen by their minimal length in teleosts and the *D. rerio* intron sequence locally aligned (Smith-Waterman) to the orthologues of the set (**B**, **D**). As a control, we also aligned a non-orthologous *D. rerio* sequence to each orthologue set (**A**, **C**). The top-scoring alignment for each orthologue set was identified and filtered to remove repeat sequences. The remaining control alignments were used to construct a null model relating the search space ($l_{dr} \times {\sum l_{\notin dr}}$, where *l*_*dr*_ is the *D. rerio*, and *l*_∉*dr*_ the other intron lengths) to the frequency of alignment scores). Plots show the deviation from the null models for teleosts (**A**, **B**) and mammals (**C**, **D**) plotted against 10th percentile teleost intron length with each point representing the maximally scoring alignment for a given orthologue set. Points in red are outside of the 95th percentile of model residual values. Bars show the proportion of points within the indicated range that are outside of the 95th percentile (right y-axis). Left panels (**A**, **C**) show data from the control alignments of non-orthologous sequences and indicate that the model only partially compensates for the increase in search space since we observe higher residuals for longer teleost introns. Nevertheless it is clear that an increased teleost intron length is associated with an increased sequence conservation and that this effect is not limited to teleost sequences

To compensate for the fact that alignments of long sequences to each other are intrinsically more likely to yield high scoring alignments, we made use of alignments to non-orthologous introns to create models representing operational null hypotheses (Fig. S13) relating the frequency of maximally scoring alignments to the total search space (see Fig. S15-S24 and additional files 6–15 for examples of alignments). Sequences from introns long in all teleosts were much more likely to diverge from these models (Fig. 7 and Fig. S14). This effect increased with minimum teleost intron length with significant local alignments to *Danio rerio* sequences against teleost and mammalian introns found in up to 60% and 20% of introns longer than 2000 bp.

## Discussion

### Intron sizes are exponentially distributed

It has previously been reported that introns specifically in *Danio rerio*, but not in other teleosts have a bimodal distribution [26]. We show here that bimodality in intron length is a general property found in most teleost clades. Our analysis differs from Moss *et. al* [26] in that we log transformed intron lengths. We believe that this is correct because the probability of an intron changing in size is likely to be a function of its length [17] and this means that the expected distributions are exponential. Furthermore, the fact that log-transformation gives rise to distributions that approximate normality argues for the suitability of log-transformation.

Moss *et al.* interpreted the appearance of a second peak of larger introns as a result of expansion of intron lengths due to transposable element (TE) expansion specifically in the *Danio* lineage. Our results, based on a large number of recently available vertebrate genomes, suggest instead that bimodality is the norm across the teleosts and may have arisen as a result of loss of ancestral sequences rather than expansion.

### Two types of teleost introns

Clear bimodal distributions are rare in genomic data and indicate the division of the underlying entities into distinct classes. Our observations argue that in most teleosts introns can be divided into two classes; short (< 256 bp) and long (> 256 bp) introns. The simplest explanation for our observations is that introns shorter than the antimode contain only sequences necessary for efficient spliceosome association [30] whereas longer introns, especially those that are long across the teleosts, also contain regulatory or other functional elements. However, we also observed a smaller peak of short introns at around 76 bp across the vertebrates with an antimode at ca. 150 bp (Fig. S2); the teleost specific peak is contiguous with this, but is both broader and higher. The peak seen across the vertebrates is similar to the sharp peaks of small intron sizes observed in both plants and metazoan clades, and it has been argued that the positions of these peaks represent clade specific (eg. ∼50 bp in *Caenorhabditis elegans*) optimal intron sizes [22]. Hence, the teleost specific major short intron peak is unlikely to merely contain a set of minimal introns and the cause for the position of the antimode (∼256 bp) remains unclear. We note that the apparent absence of this antimode in a number of teleost clades (esp. the salmonids) suggest means for identifying the molecular or evolutionary mechanisms responsible for the typical teleost distribution observed here.

In teleosts, the frequency of introns shorter than 76 bp drops rapidly and the peak of shorter introns appears to either lie very close to, or be coincident with, a putative vertebrate minimal intron size. In most teleosts (including those with larger genomes like *Danio rerio*) this peak represents the most common intron size suggesting either selection for minimal intron size or molecular mechanisms favouring intron size reduction. The general lack of conservation of minimised introns indicate that any such process must be balanced by other processes that enlarge introns. Nevertheless, the preponderance of minimally sized introns in teleosts suggests that the balance of these competing processes has favoured intron size minimisation. The variance in the proportion of minimised introns, and the correlation of this minimisation with genome size (Fig. 5H,I) suggests that the balance of the competing processes varies and that this variance is not specific to introns but applies equally to non-coding sequences in general.

### An ongoing process of intron size diversification

Our observations suggest that vertebrate ancestral intron sizes were in general longer than in the teleosts indicating an active loss of intron sequence across the teleosts. The alternative hypothesis, that ancestral introns (and presumably genomes) were small would indicate a reduced speed of intron growth in teleosts compared to other vertebrates. This seems less likely given that intron sizes are more variable within the teleosts than other vertebrate clades. An active process of intron and associated genome size reduction within the teleosts also seems more likely since the teleosts descend from an ancestor that underwent an additional round of genome duplication [31] and as such would be expected to initially have a larger than typical genome.

A similar decrease in genome and intron sizes has previously been reported in birds (Aves) and in flying vertebrates generally [27, 32, 33]. In birds it has been suggested that both genome and intron size reduction is related to the increased metabolic demands of flight; however, alligators also appear to have smaller introns and it is unclear as to whether genome shrinkage occurred prior to powered flight [28]. Similar explanations have been suggested for genome size reduction in teleosts, though the lack of a correlation between genome size and metabolic rate makes this less likely [34].

### Introns with function

Why some introns retain length across the vertebrates even in species where there is an apparent evolutionary pressure for genome size minimisation is unclear, but argues for function, either of the length itself, or encoded in sequence. Introns can contain regulatory regions [23] and indeed, in mammals, introns containing higher densities of conserved sequences are longer than those lacking such sequences [17]. Such conserved sequences may include not only cis-regulatory elements but can also encode functional molecules such as miRNAs, snoRNAs or lncRNAS, and even exons of overlapping genes. Intron length is more strongly conserved in genes associated with embryonic development [35] and the length of introns from genes that are co-expressed or that belong to the same protein complexes appear to have co-evolved [36] suggesting that the intron length per-se can have functional implications. This role might be partly explained by the fact that intron length can affect the delay between transcription and protein expression which can change the timing of regulatory networks [25, 37].

The observation that introns that are long across the teleost clade are also likely to be long in other vertebrates supports the notion that these introns have specifically retained length for functional reasons. This is consistent with vertebrate introns being divided into two classes; those containing longer functional regions and those that do not. Although both classes would be subject to minimisation by evolutionary processes, the minimal size of the former would be larger than the latter. In teleosts, which have evolved small genomes, loss of sequence from the latter class of introns would have been more prevalent than from the former class.

Our observations argue that long and short introns have different modes of evolution with short introns being less likely to have conserved length (Fig. 5) across species. This difference could be due to the selection against loss of functional sequences but it could also arise as a side-effect of the specific mechanisms that change intron size. For example, the insertion of a transposable element into a short intron represents a larger fractional length change for a short intron compared to long introns; thus although the frequency of such events may be linearly related to intron length, the consequences of the events are not equivalent and could lead to apparent noise in short intron size where the distinction between short and long introns depend on the frequency and size spectrum of mutations that cause the changes.

It is also possible that sequence has been lost from a specific set of introns rather than there being a selective retention of sequence in introns with function. This could conceivably occur as a result of increased recombination rates at such introns, or because of an increased evolutionary selection against length at such loci (eg. as a result of high gene expression [17]). Untangling these alternatives is difficult because selective retention and loss both result in similar outcomes. However, a selective loss of intron sequence would be more likely to be associated with genes or genome regions rather than specific introns and this could potentially be used to argue for one scenario.

### Intron and genome size

The small sizes of teleost genomes and introns are intrinsically linked, with intron size reflecting genome size. Since a reduction in intron size cannot markedly reduce genome size (Fig. S1) it is likely that the short introns are a side effect of the evolution of small genomes in the teleost lineage.

Why teleosts specifically should have small genome sizes remains unclear, but may be related to the high fecundity and associated developmental strategies of the teleosts. Most teleosts produce large numbers of small eggs giving rise to extreme number of offspring, and within the teleosts [34] and many other clades [38] there appears to be a relationship between egg size and genome size. Additionally, although not universal across all clades [39], both a small propagule (the size of the stage that leaves its parents) and a high fecundity are strongly associated with a large effective population size (N _*e*_) in metazoans [40].

The distribution of intron sizes in teleosts with smaller genomes (*Takifugu rubripes*, *Tetraodon nigroviridis*, *Betta splendens*) is reminiscent of those in *Drosophila* species [14, 41, 42], where about half of introns are between 45 and 110 bases long. In *Drosophila*, large introns are found preferentially at sites of low recombination leading to the idea that an amelioration of Hill-Robertson interference (the competition between two linked loci for fixation in the absence of recombination) may be a driver for increased intron size and that species with lower N _*e*_ will have larger introns [41]. In addition, a large N _*e*_ supports more effective purifying selection [43, 44] that might be able to reduce the rate of fixation of mutations leading to larger introns even where these have only very small negative effects. These ideas are consistent with the decreased N _*e*_ and increased genome sizes of groundwater living asellid isopods [44]. Hence, the small size of teleost genomes (and introns) may simply be a consequence of the typical teleost breeding strategy of having large numbers of small offspring. This is supported by the observation that marine fish tend to have larger N _*e*_, but smaller genomes than freshwater species [45].

It has also been argued that the process of recombination itself leads to an increased deletion bias and underlies genome contraction in Avian species [46]. Recombination rates may thus affect intron size both during the generation and selection of genetic variance. Our observations show that the lengths of long introns are generally conserved across the vertebrates; if these are to be explained by recombination rate variance, then recombination rates at these introns should be low throughout the vertebrates. This is testable given adequate recombination rate maps.

## Conclusion

Teleosts have both genome and intron sizes that are unusually small for vertebrate species. We show here that teleost intron sizes are likely to have decreased from those in the last common vertebrate ancestor. This decrease appears to have started in the ancestor of the teleosts but to have continued in the independent teleost lineages, suggesting either selection by common evolutionary pressure or molecular mechanisms specific to the teleosts.

A subset of introns have escaped from this process and maintained their lengths. Members of this set are also more likely to be long in both mammalian and avian species suggesting a function dependent on the sequence content or the length itself that is conserved across the vertebrates.

It is unclear as to why introns have shrunk in the teleost lineages, but it is likely to be a side-effect of a general evolution towards smaller genomes. This may have been driven by neutral evolution or by selective forces. In either case, the decrease in genome size may have facilitated teleost specific developmental strategies.

## Methods

All analyses, unless specifically mentioned were performed using a mixture of SQL, Perl, R and C. All data visualisations were created using core R functions. Detailed descriptions of methods, species and genes used are provided in the supplementary methods.

### Intron sizes and orthology

Exon and intron sizes were inferred from genomic coordinates obtained from 172 (54 Teleost, 80 Mammalia, 31 Sauria and 7 other vertebrates) species using locally installed Ensembl [21] core 98 databases. The intron orthology was constructed from a set of 6114 protein families which had a single orthologue in at least 130 of the 172 species according to the protein family classification of the Ensembl compara 98 database. Orthologous introns were identified by aligning transcript sequences modified to have ’sticky’ meta-characters representing introns at exon-exon boundaries to their *Danio rerio* orthologues using a Needleman-Wunsch algorithm [47] implemented as an extension to R in C. Introns aligning to each other were considered orthologous.

### Intron size conservation

Mutual information estimates were obtained using the entropy package https://CRAN.R-project.org/package=entropy[48]. Kimura two factor distances [49] were based on all against all (species) alignments of the members of 472 protein families that had members in at least 170 of the 172 species. These distances were used to construct a neighbour-joining tree using the nj function of the ape package https://cran.r-project.org/web/packages/ape/index.html[50]. The ancestral lengths of introns of the internal nodes of this tree were inferred using an implementation of Sankoff maximum parsimony [51] with intron lengths discretized to integral 10×*log*_2_ values and transition costs being the difference in intron length state. The Sankoff maximum parsimony was implemented as an R plug-in in C and Kimura two factor distances calculated in R.

The major modes of ancestor to extant intron size evolution were derived from the joint distribution of introns longer than 75 bp discretised to integral 10×*log*_2_ sizes. The resulting two-dimensional distributions were scaled column-wise such that the column means and standard deviations were 0 and 1 respectively and then smoothened by a serial convolution in both dimensions by a normal distribution with a mean of 0 and standard deviation of 6 (Gaussian blur). Peaks in columns representing ancestral sizes were joined and smoothened with a normal kernel to facilitate visualisation of the behaviour across the vertebrates.

Size discretised correlation coefficients between two species were calculated for the set of introns larger than or equal to 76 bp in both species by dividing the subset of introns lying within the 99th percentile (to remove the effects of outliers) in one species into two sets (Fig. 5H) containing equal size ranges. The correlation coefficients were then calculated separately for the orthologous introns in the two sets. The sizes of introns were thus constrained for only one of the species.

### Gene ontology

Initial GO statistics were obtained using human annotation from the org.Hs.eg.db [52] and GOstats [53] packages. Detailed analyses of enriched / depleted GO terms were based on the org.Hs.eg.db annotation using the phyper function to calculate enrichment and depletion probabilities of nested sets of genes ordered by their minimal teleost length.

### Intron sequence conservation

We used a recursive Smith-Waterman [54] implementation to identify all local alignments between pairs of intron sequences. The implementation first identifies the optimal local alignment between the pair, and then recurses to find additional alignments that do not overlap within the seed species (*D. rerio* for all analyses shown). To allow comparisons between introns with different types of length distributions across the teleosts we selected five sets of introns, ‘long’: 1594 introns with a min teleost length of 1024 bp, ‘med’: 2000 introns with a min teleost length between 256 and 1024 bases, two sets of 2000 ‘short’ introns with min teleost lengths between 90 and 256 bases, and a set of ‘ctl’ introns with a median intron length less than 256 bp but with a *D. rerio* length of more than 1024 bp. The ‘ctl’ and one of the ‘short’ sets were sampled randomly from the allowable subset, the ‘long’ set included all introns satisfying the criteria. All other sets used the 2000 introns with the smallest variance in teleost intron length. Alignments where the individual search space (*l*_1_×*l*_2_ where *l*_1_ and *l*_2_ are the lengths of the two sequences) was larger than 1e8 where excluded to reduce memory requirements.

To simplify the statistical assessment we focused on the top scoring alignment for each *D. rerio* intron. To compensate for the effect of the differences in search space on the observed top scores we performed alignments for the ‘ctl’, ‘med’ and ‘long’ intron sets but using a non-orthologous *D. rerio* sequence of similar length. The resulting alignment scores were used to obtain a linear model ($log(S) \propto log({l_{dr} \times {\sum l_{\notin dr}}})$, where *S* is the maximum score observed and *l*_*dr*_ is the *D. rerio*, and *l*_∉*dr*_ the other intron lengths) representing the likelihood of observing a given score for a given alignment search space (Fig. S13). In order to remove the effect of simple repeats and other repetitive sequences from the effects we screened all aligned sequences (for both the orthologous and non-orthologous alignments) by BLASTn of *D. rerio* sequences against the *D. rerio* genome. Any aligned *D. rerio* sequence which had more than 2 sequences aligned to 50% of its length or no sequences at all (indicating simple repeats) were removed from further consideration. Not surprisingly, far more introns were removed from the non-orthologous alignments (Fig. S13, S14). The divergence from this model was calculated for both orthologous and non-orthologous sets and considered as a measure of effective sequence conservation.

## Supplementary Information

**Additional file 1** Vertebrate intron size distributions. *log*_2_ transformed intron size distributions for vertebrates. Blue and red lines indicate the distributions of all and first introns respectively. Panels are ordered by genome size from small to large. The major vertebrate clade (teleost, sauria, mammals and others) are indicated for each plot. Dashed vertical lines indicates the inferred mammalian small peak (105 bp) and the mammal and teleost antimodes at approximately 150 and 256 bp respectively. All intron sizes are from Ensembl version 98.

**Additional file 2** Vertebrate intron size distributions. *log*_2_ transformed intron size distributions for vertebrates taken from Ensembl version 104. The species taxonomic identifcation taken from the NCBI taxonomy is shown on the right for each species. The two vertical red lines indicate the positions of the antimode and peak of larger intron sizes in *D. rerio*.

**Additional file 3** Gene ontology depletion statistics. Individual panels show statistics associated with an under-representation of members of gene ontology groups in nested sets of human genes ordered by the minimum size of their largest orthologous teleost intron. The statistics are shown plotted against the size (*log*_2_ transformed) of the largest teleost intron of the nested set (x-axis). The statistics shown are: *log*_2_(*observed*/*expected*) (black), −*log*_10_ hypergeometric probabilities associated with the observations (red), *q* the number of members of the gene ontology set (blue) and *k*, the total number of genes in the nested set (purple). The dashed vertical lines show the position of the minimal hypergeometric p-values. The minimal p-values are associated with an inflexion in the observed / expected ratios and occur close to the antimode of the typical teleost intron length distribution. Pages 1-7: Biological Process (BP), 8: Molecular Function (MF), 9-11 Cellular Compartment (CC).

**Additional file 4** Local alignments of *D. rerio* intron sequences to vertebrate orthologues for a set of introns that are long (>1024 bp) across the teleosts. Alignments were performed recursively to identify all non-overlapping alignments to the *D. rerio* sequence.

**Additional file 5** Local alignments of *D. rerio* intron sequences to vertebrate orthologues for a set of introns with variable teleost lengths; introns were long (>1024 bp) in *D. rerio* but had a median teleost length less than 256 bp. Alignments were performed recursively to identify all non-overlapping alignments to the *D. rerio* sequence.

Additional file 6–15 Transcript and intron alignments for points in Fig. S14. Each panel shows the maximally scoring alignment between *D. rerio* and teleost intron orthologues (lower) and transcript alignment (upper) used to establish the intron orthology. Grey, white and red parts indicate aligned exonic sequence, gaps and positions of intron meta-characters respectively. Colours in intron alignment represent bases (A blue, C cyan, G green, T brown, N grey, gap white). Curves lying between sequence representations show a normal kernel density smoothed estimate of local similarity (9 bp window, standard deviation two); vertical lines indicate matches. Region between exon and intron alignments indicates the location of the maximally scoring alignment in the introns. Upper sequence *D. rerio*. Files 6–10 and 11–15 contain alignments to teleost and mammalian sequences respectively. Each file corresponds to one panel in Fig. S17 and to one specific teleost size class: Files 6,11: long (E,J), 7,12: medium (D,I), 8,13: short.2 (C,H), 9,14 short (B, G) and 10,15 ctl (A,F).

## Data Availability

All data used in this study is publicly available from Ensembl http://www.ensembl.org version 98 available by ftp from ftp.ensembl.org/pub/release-98/. Gene coordinates, orthology information and genome sequence can be found in the mysql, compara and fasta subdirectories respectively. Code written for the analyses carried out is described in the supplementary section and is available from https://github.com/lmjakt/ in the repositories intron_size_evo_1 (https://github.com/lmjakt/intron_size_evo_1) and intron_size_evo_2 (https://github.com/lmjakt/intron_size_evo_2).

## Abbreviations

bp: base pairs
TE: Transposable Element
JVA: Jawed Vertebrate Ancestor
GO: Gene Ontology
BP: Biological Process
MF: Molecular Function
CC: Cellular Compartment
