# Supplementary figures and images for: Intron size minimisation in teleosts
Source: BMC Genomics. 2022 Sep 1;23:628. doi: 10.1186/s12864-022-08760-w (PMC9438311; doi:10.1186/s12864-022-08760-w)

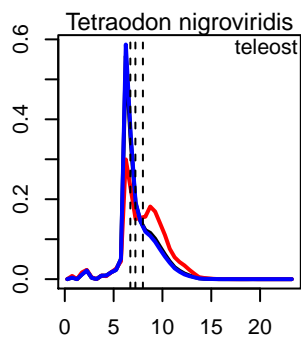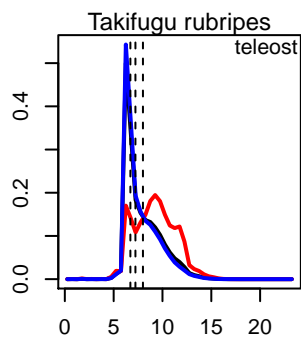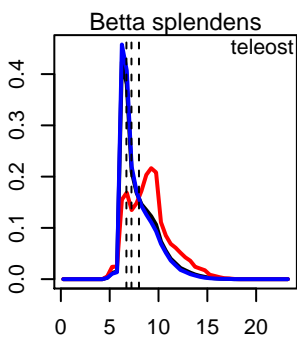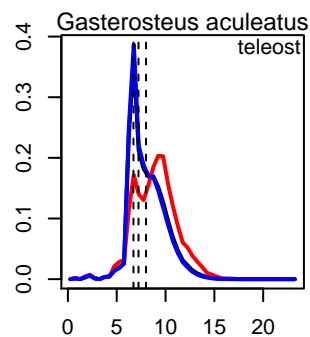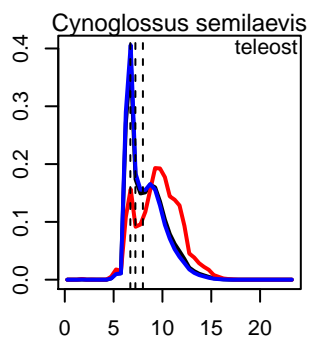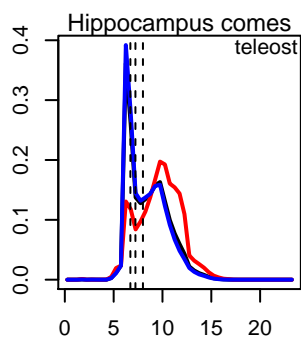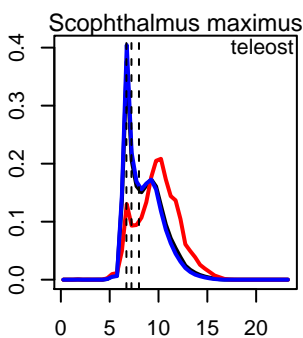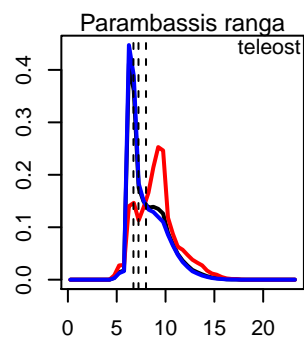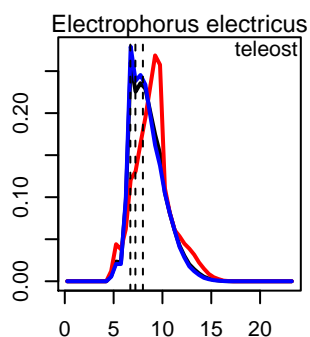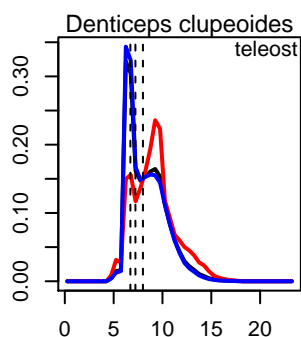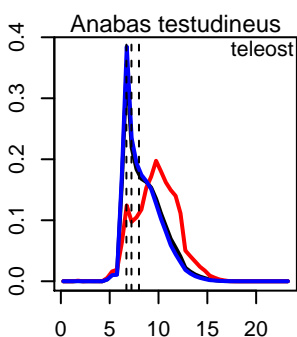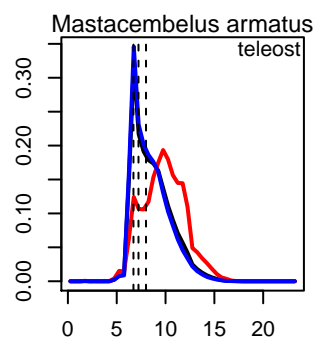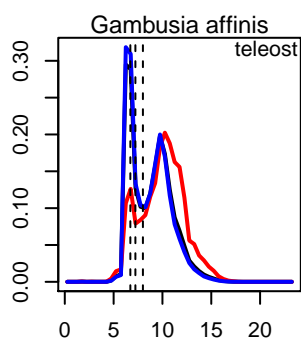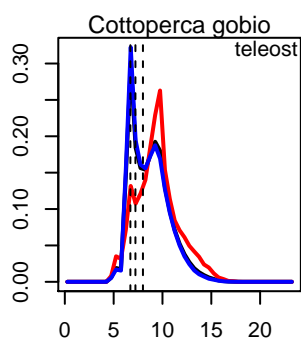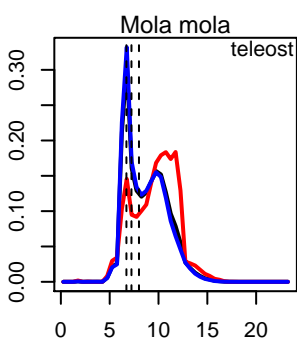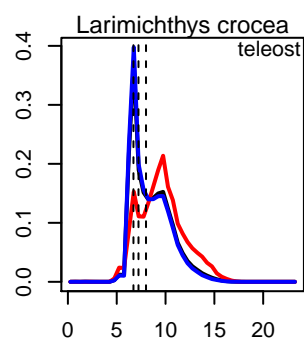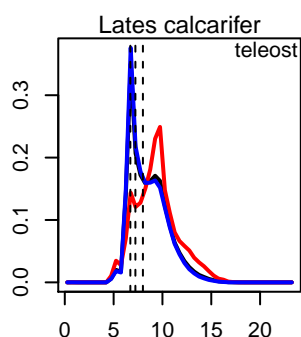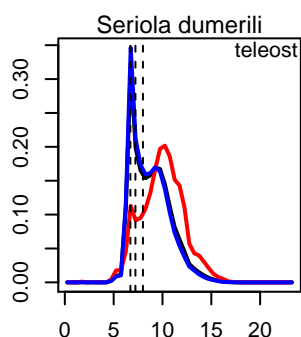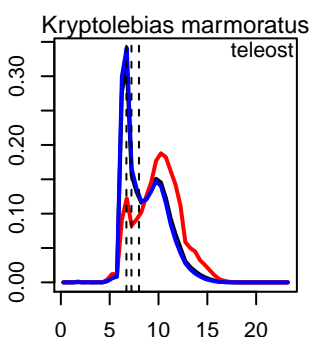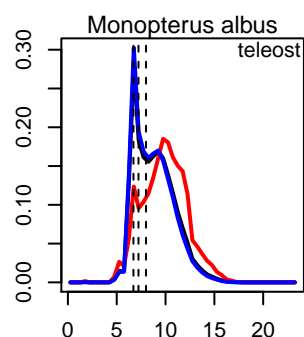

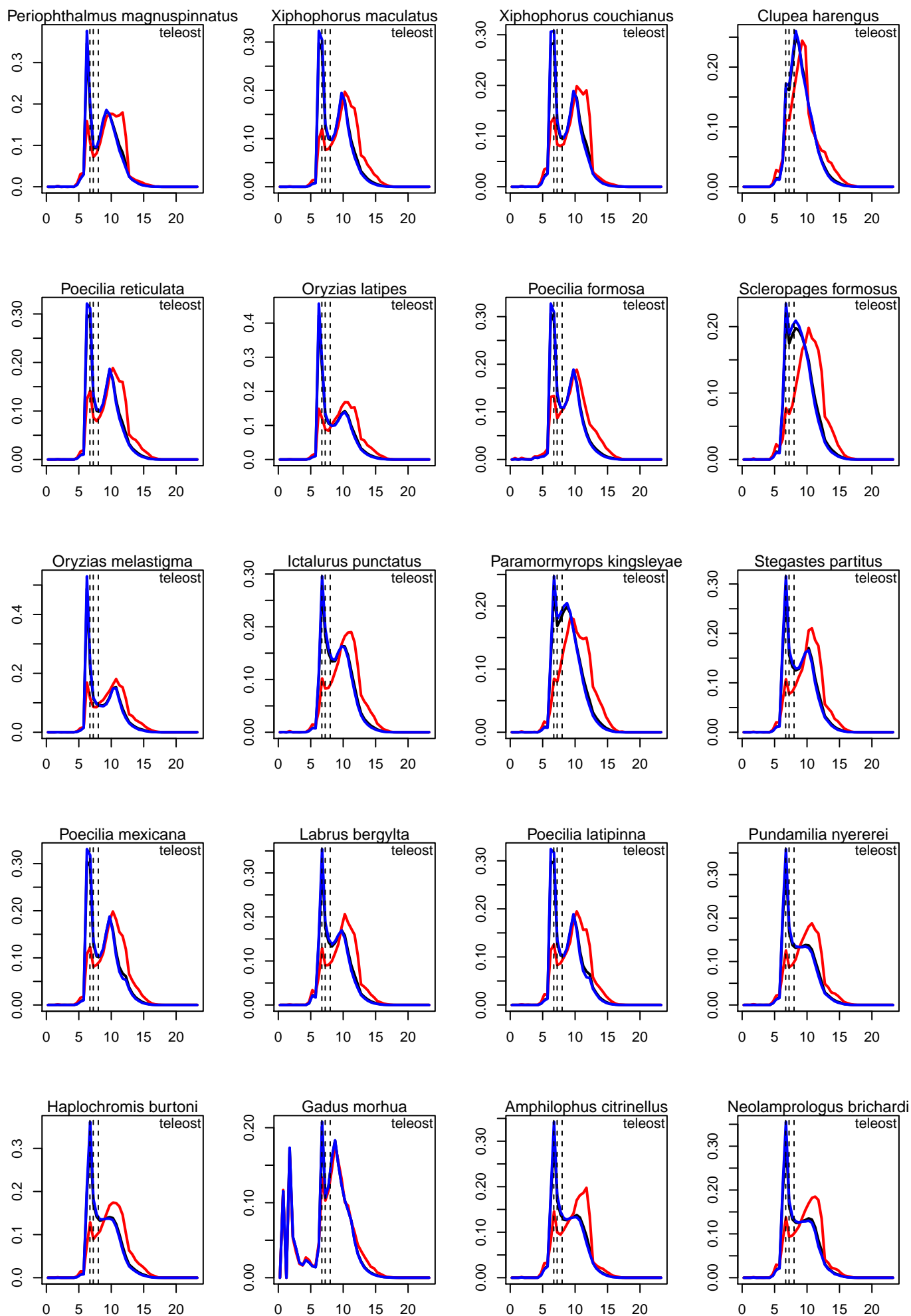

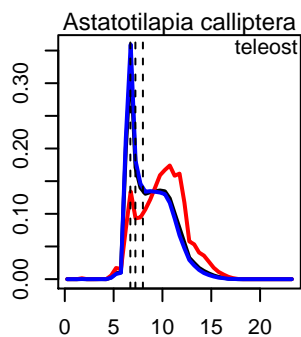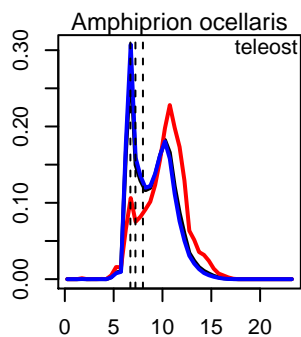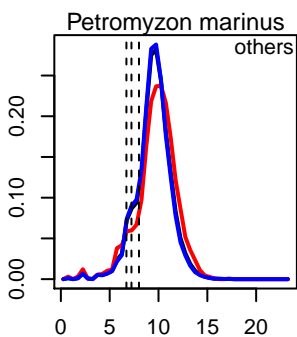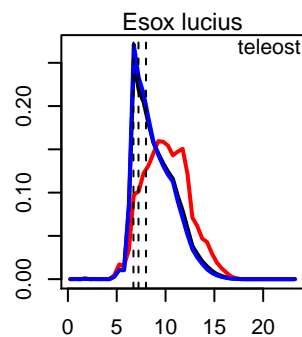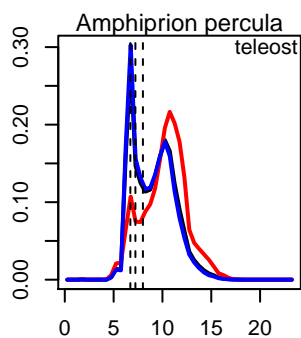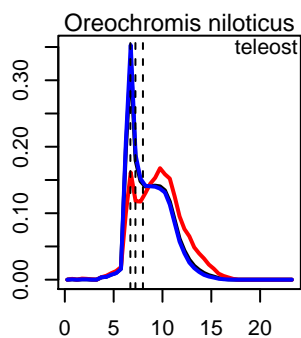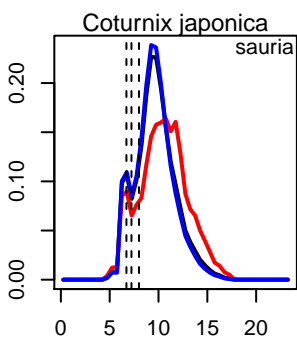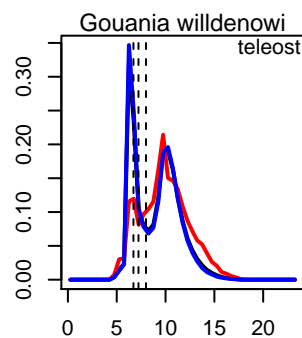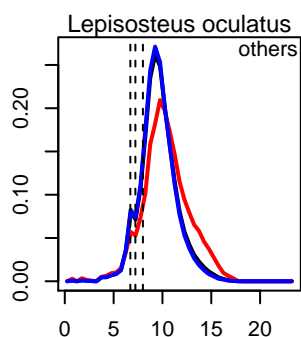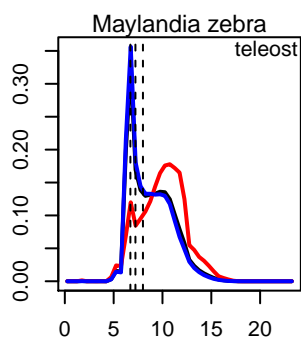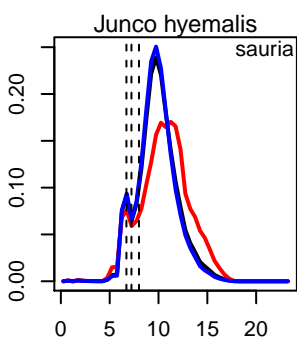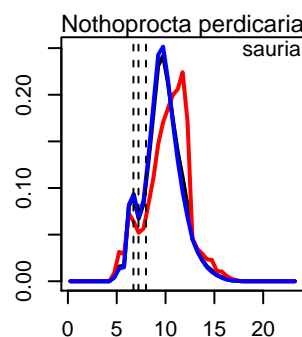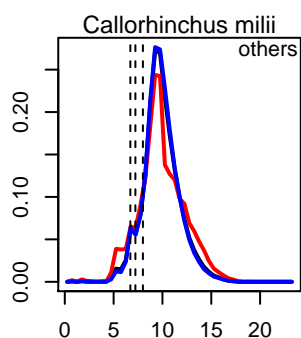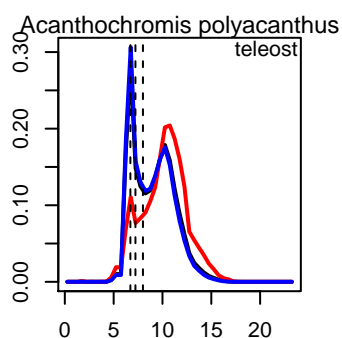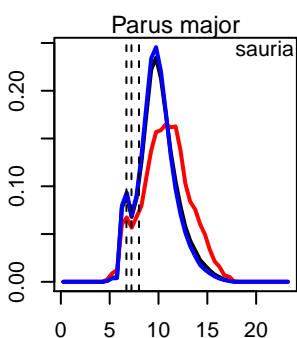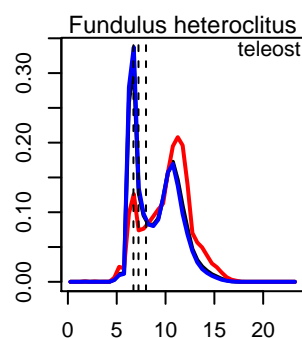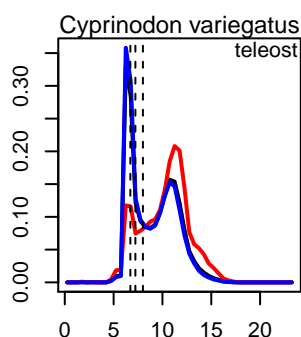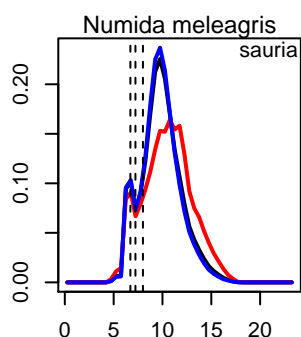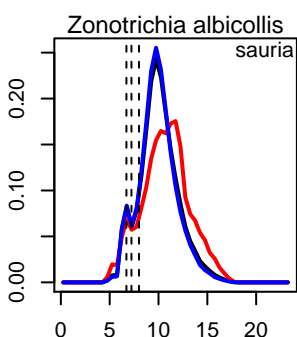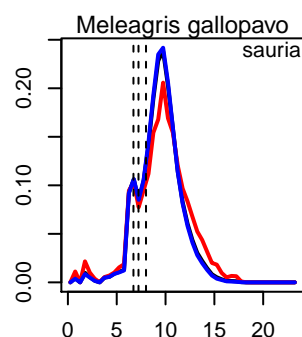

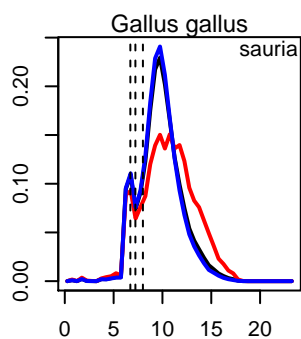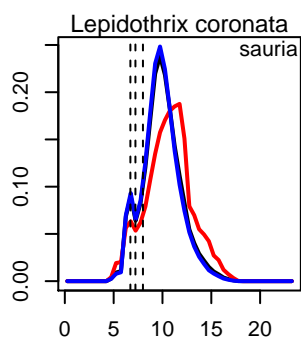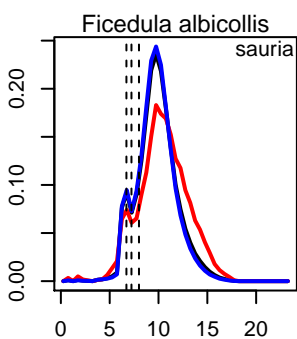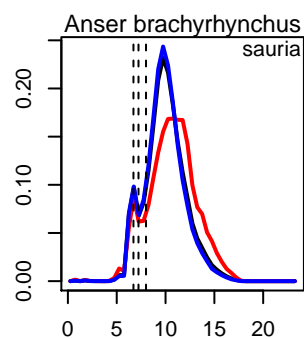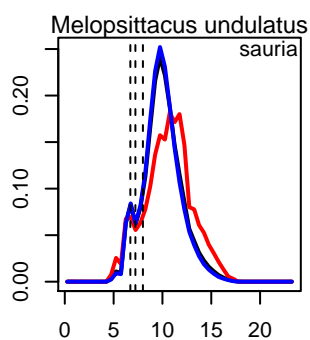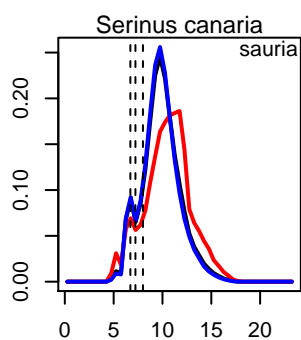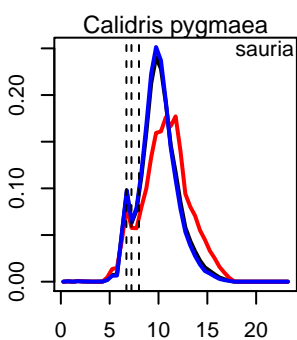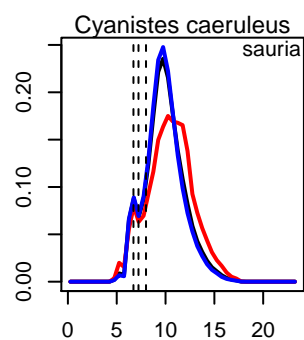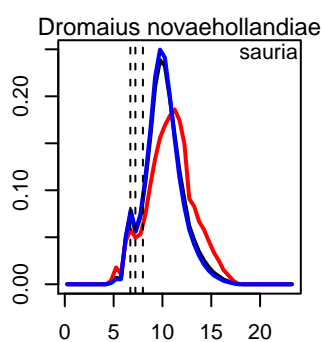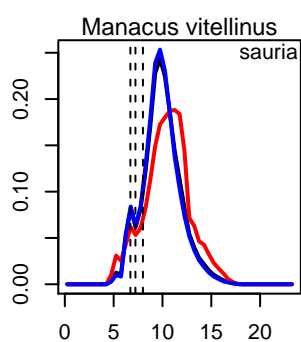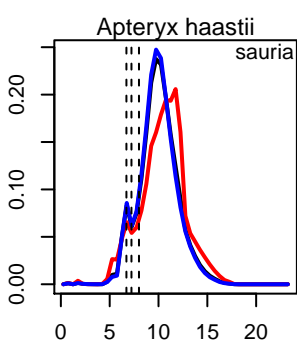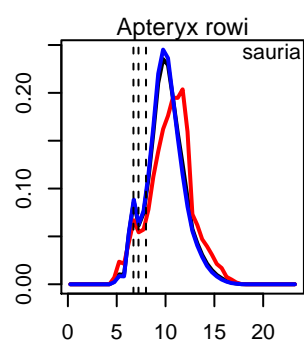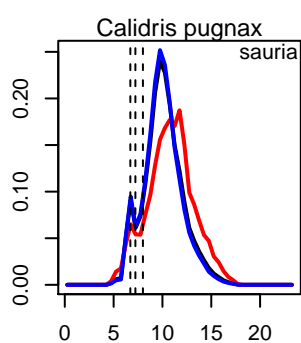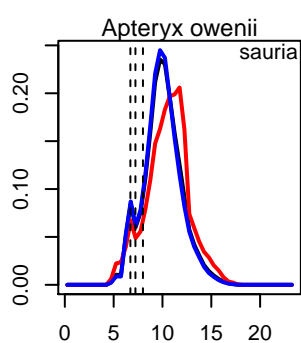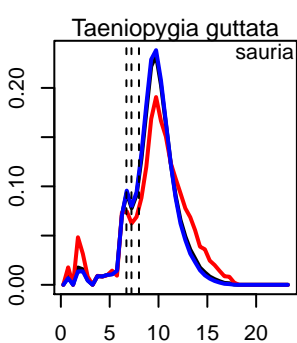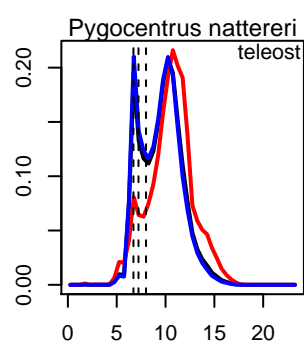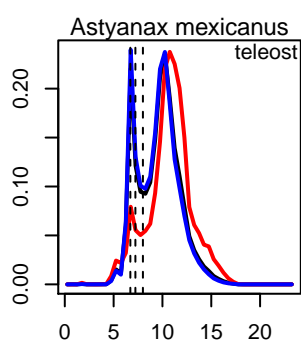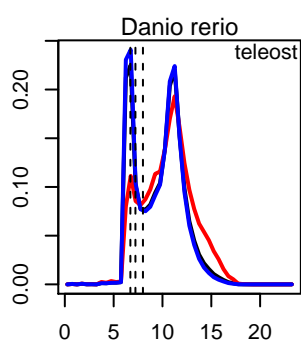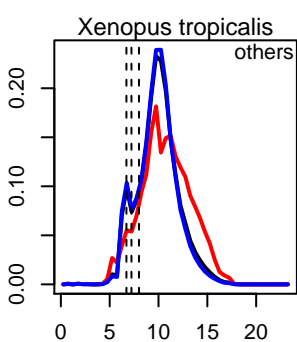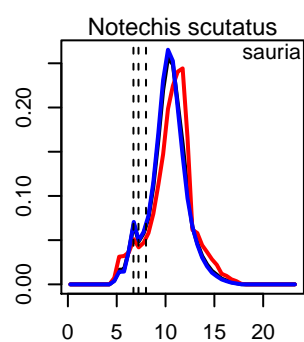

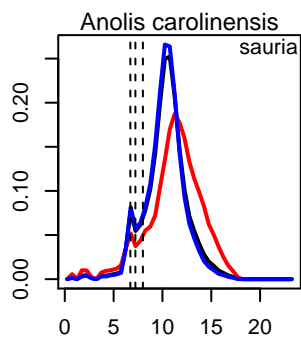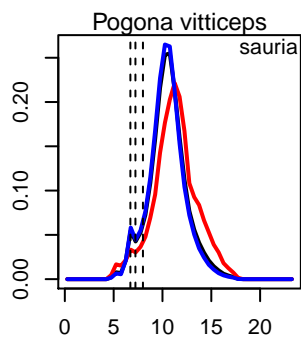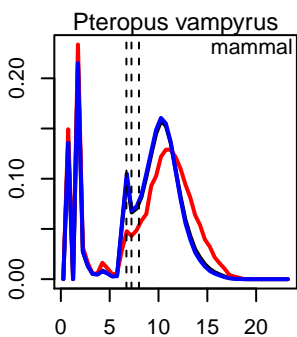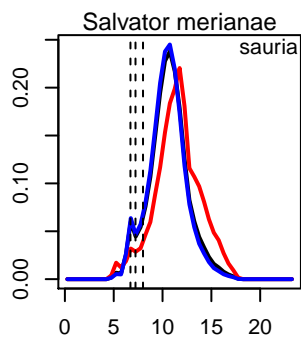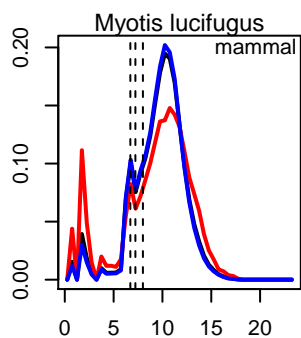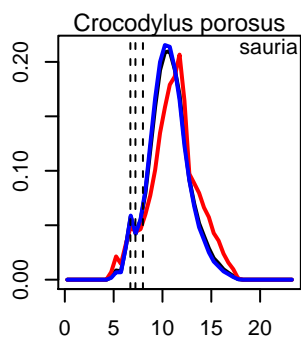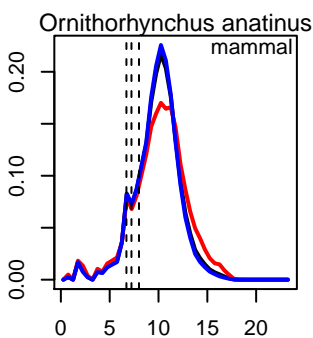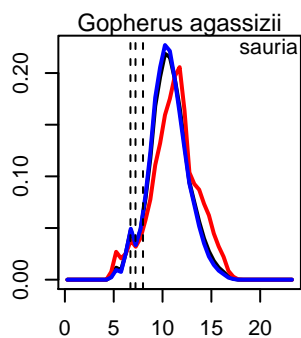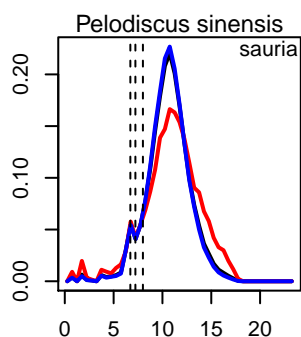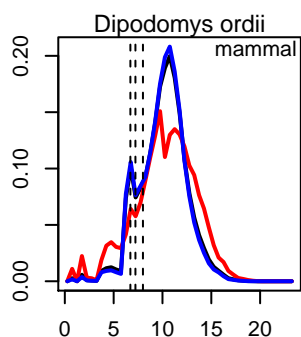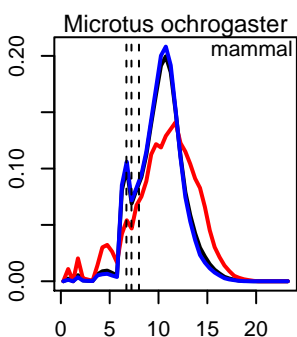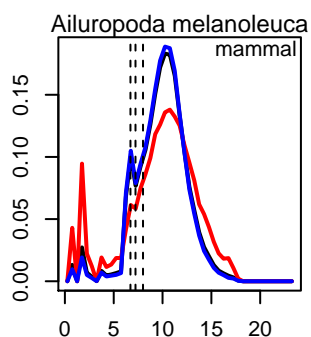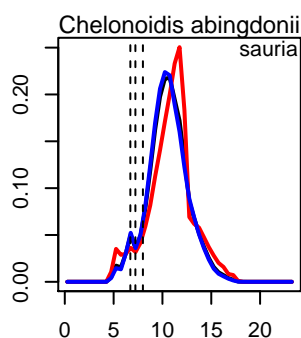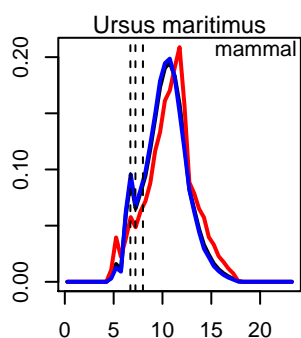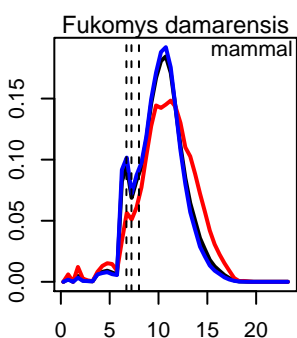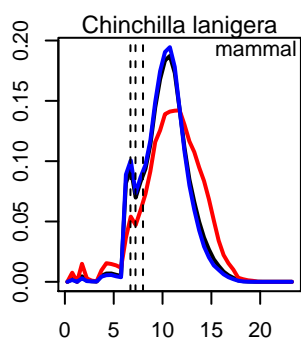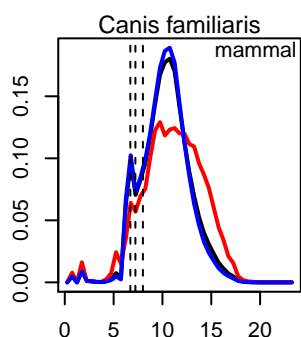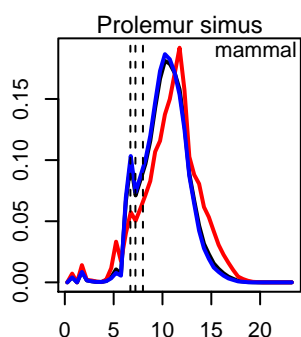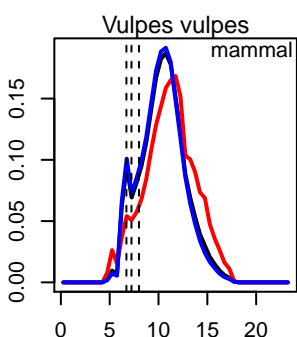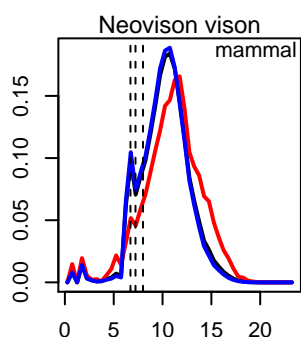

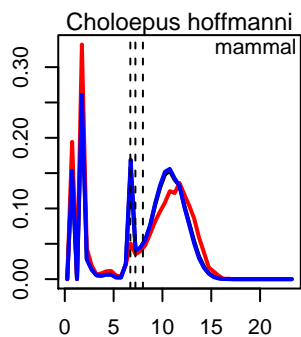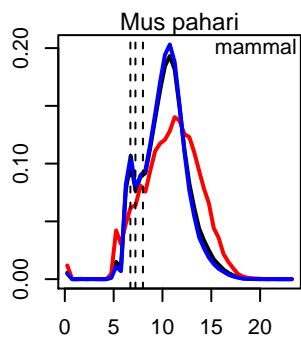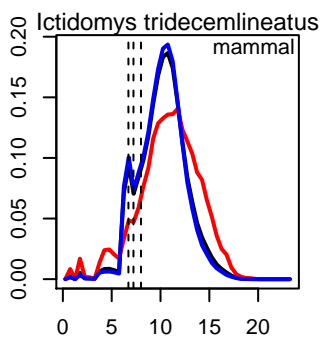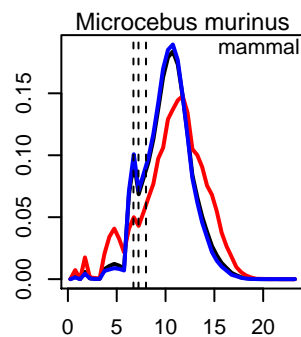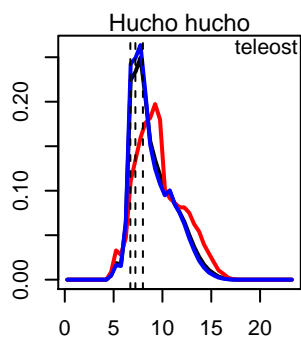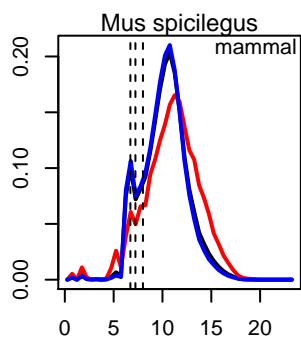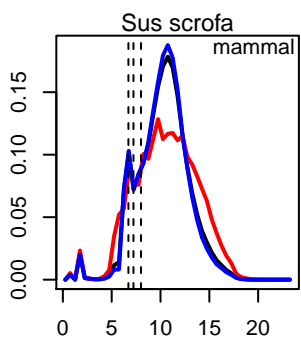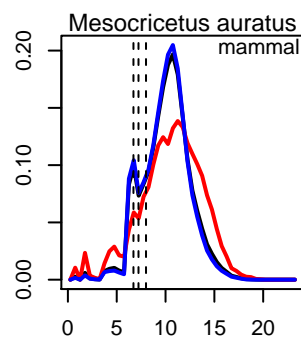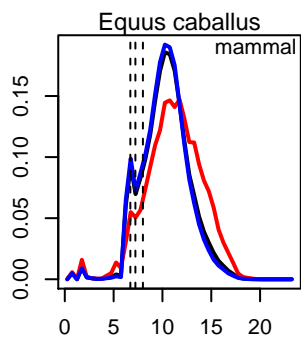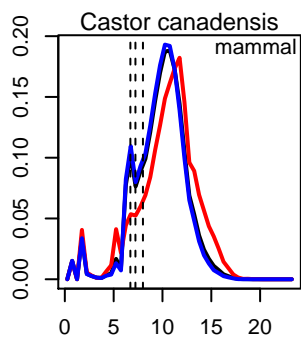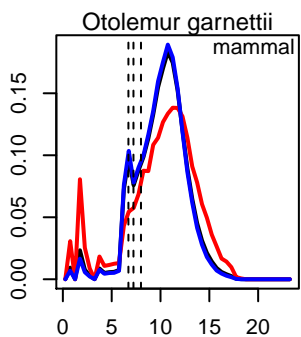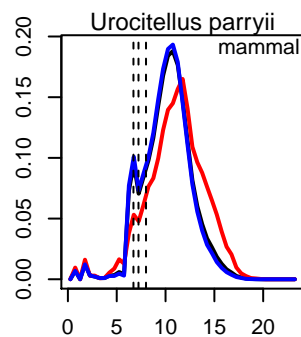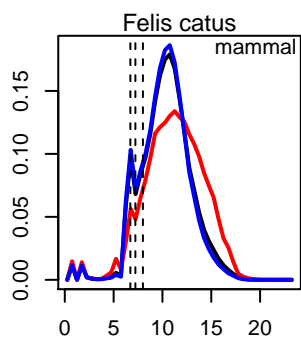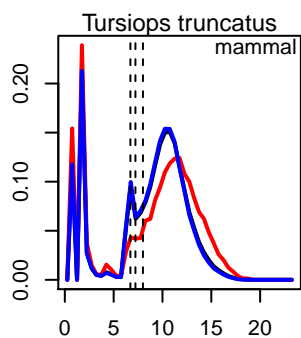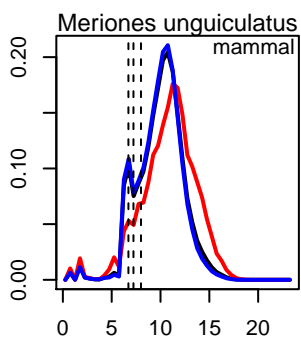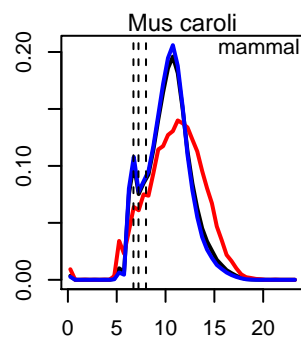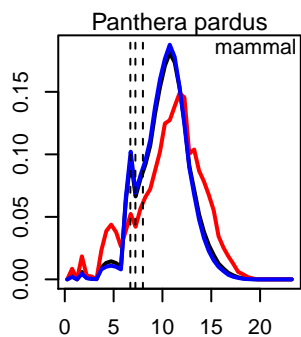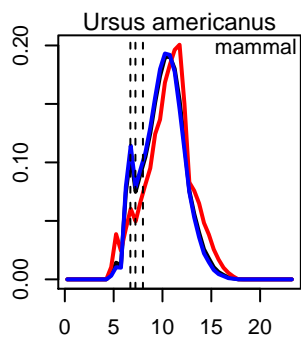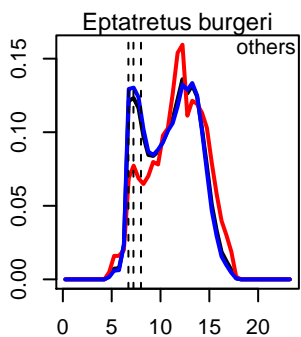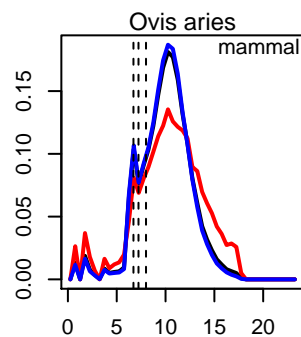

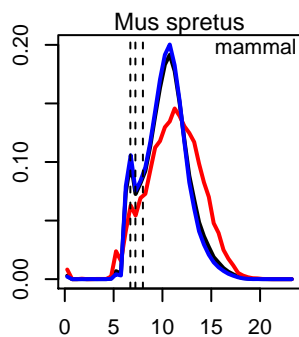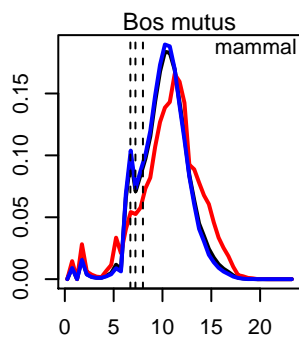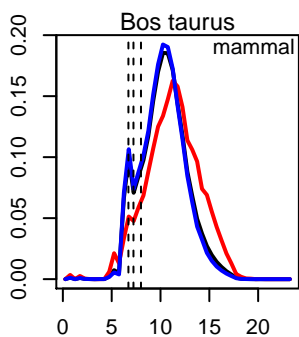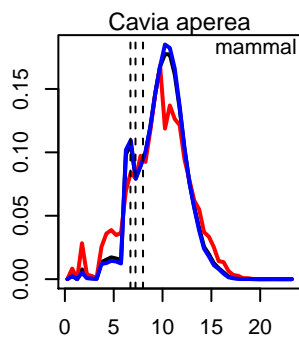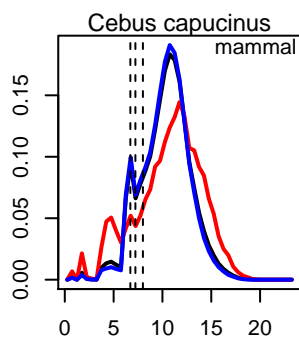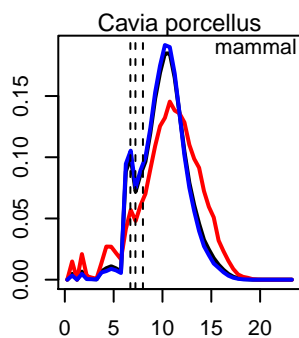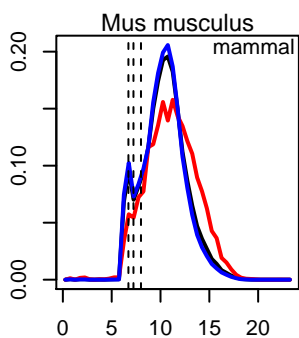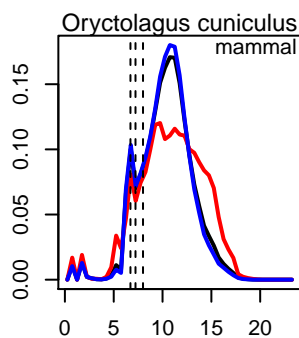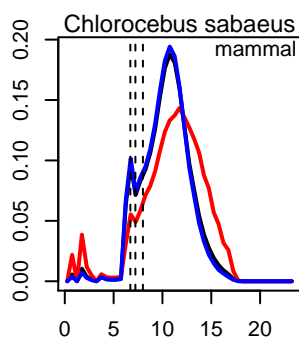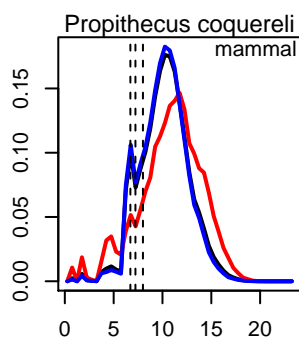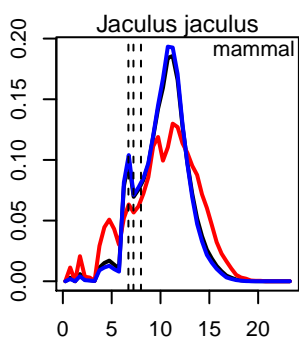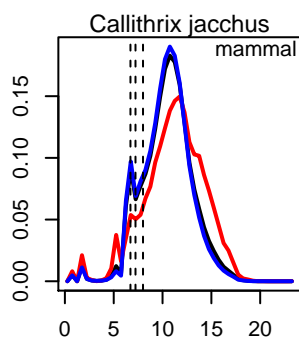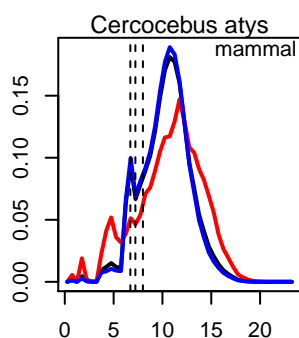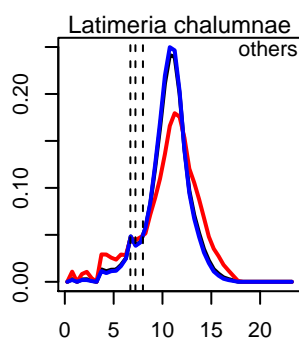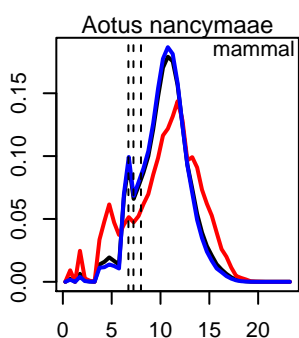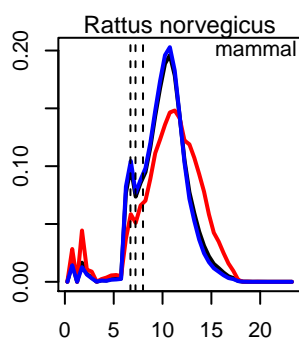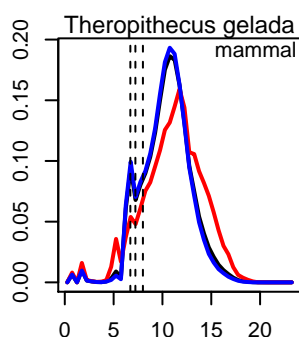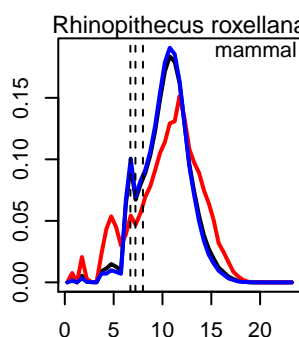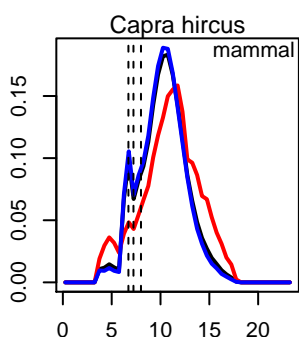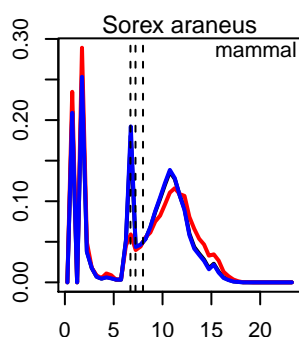

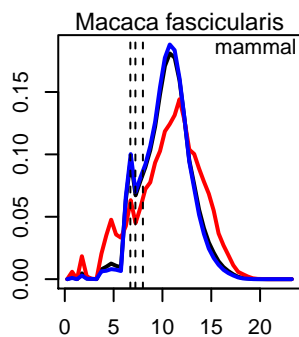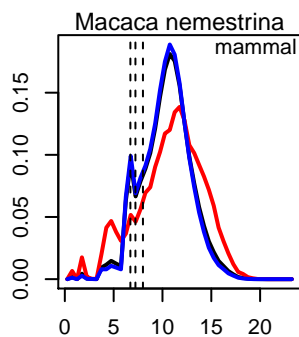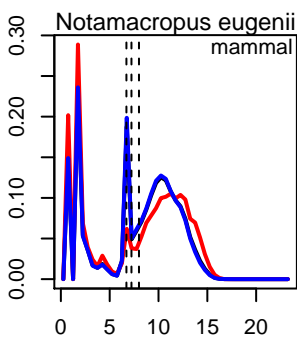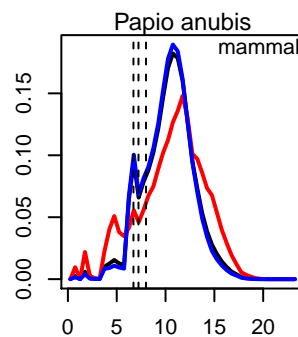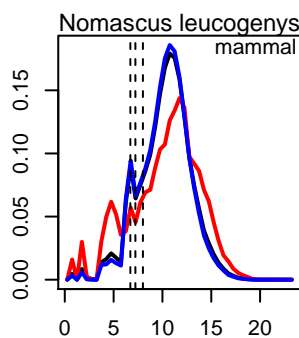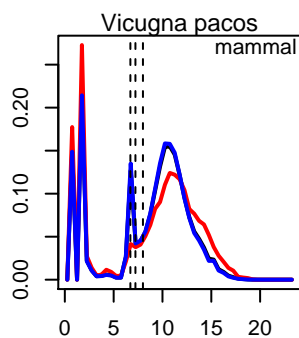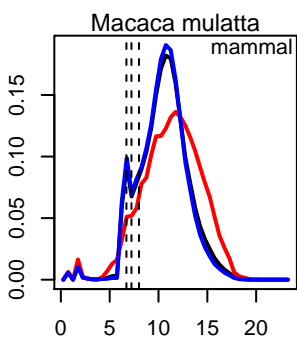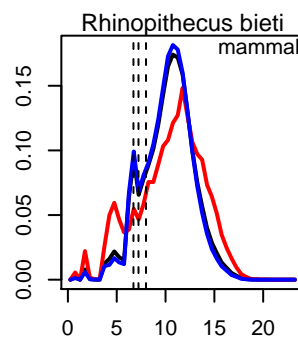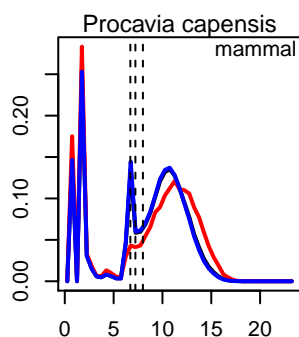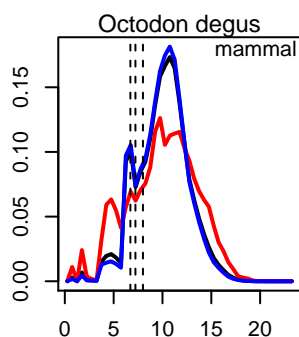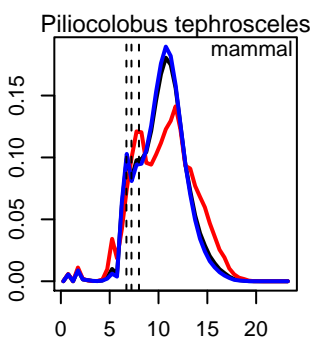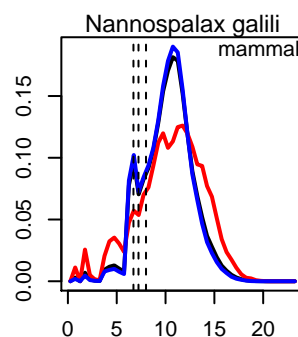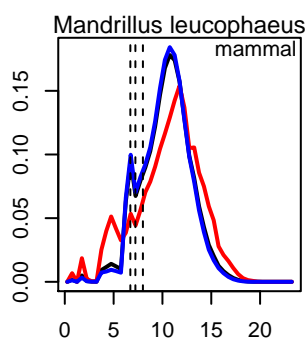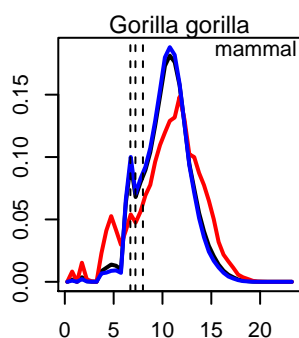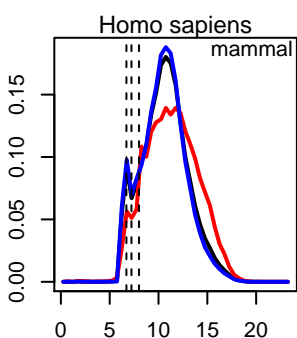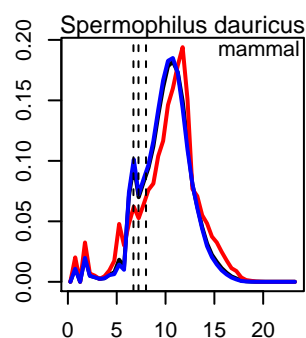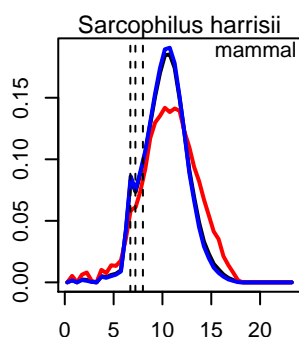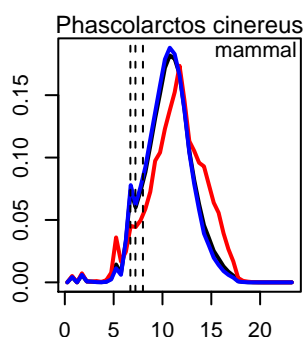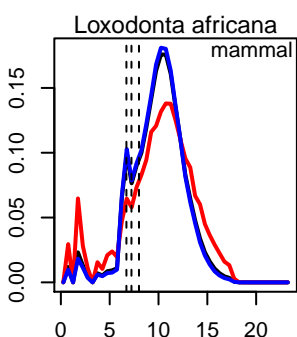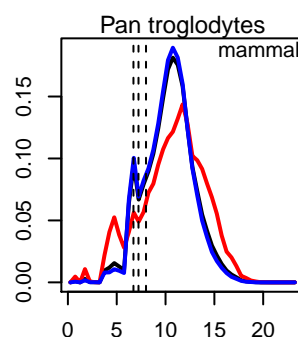

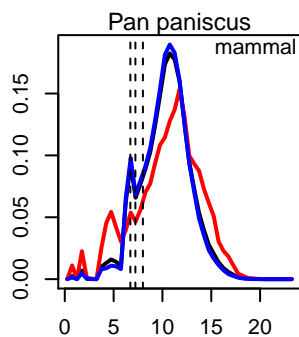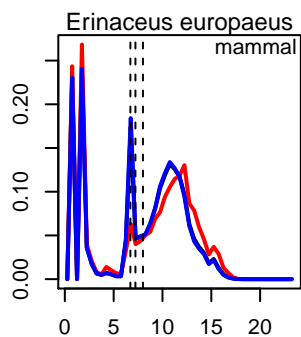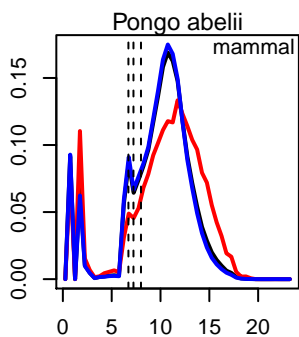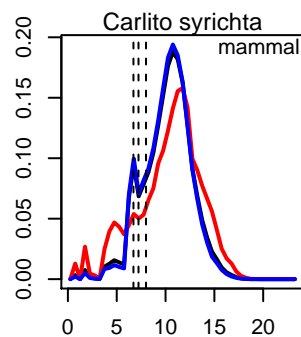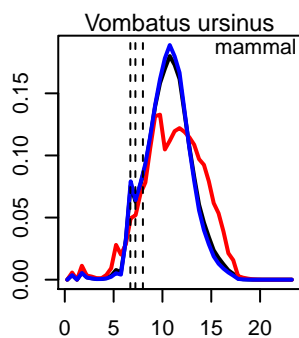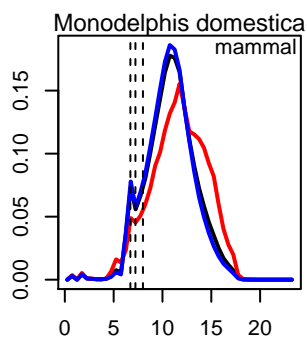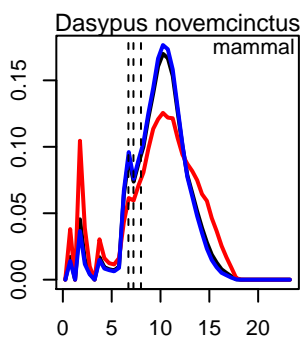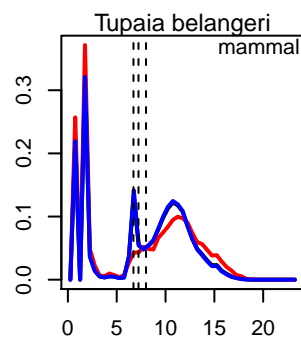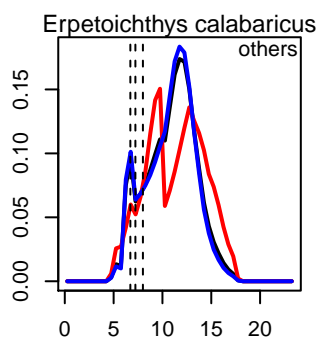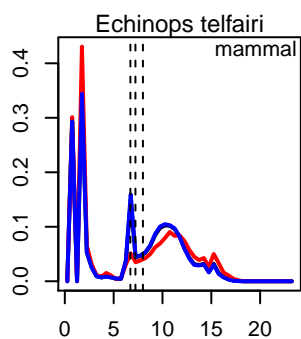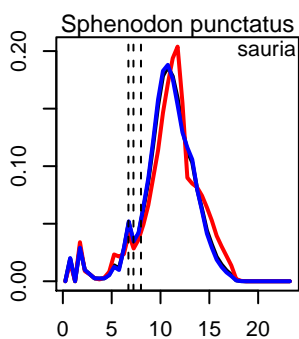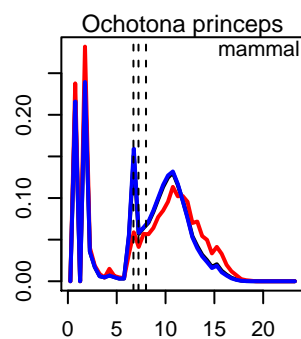

Supplement: Supplementary file 1 — Additional file 1 Vertebrate intron size distributions. log2 transformed intron size distributions for vertebrates. Blue and red lines indicate the distributions of all and first introns respectively. Panels are ordered by genome size from small to large. The major vertebrate clade (teleost, sauria, mammals and others) are indicated for each plot. Dashed vertical lines indicates the inferred mammalian small peak (105 bp) and the mammal and teleost antimodes at approximately 150 and 256 bp respectively. All intron sizes are from Ensembl version 98. [file 12864_2022_8760_MOESM1_ESM.pdf]

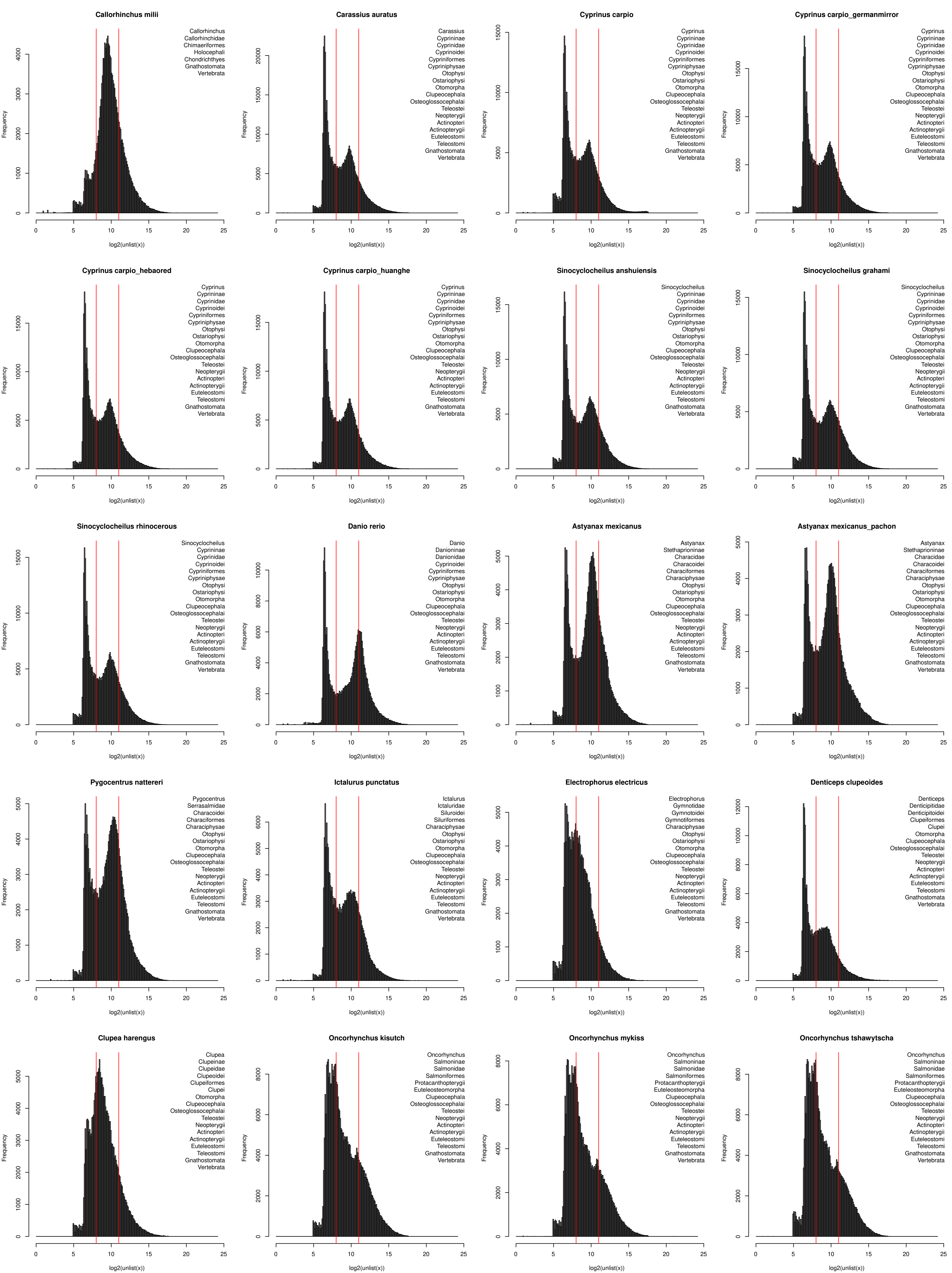

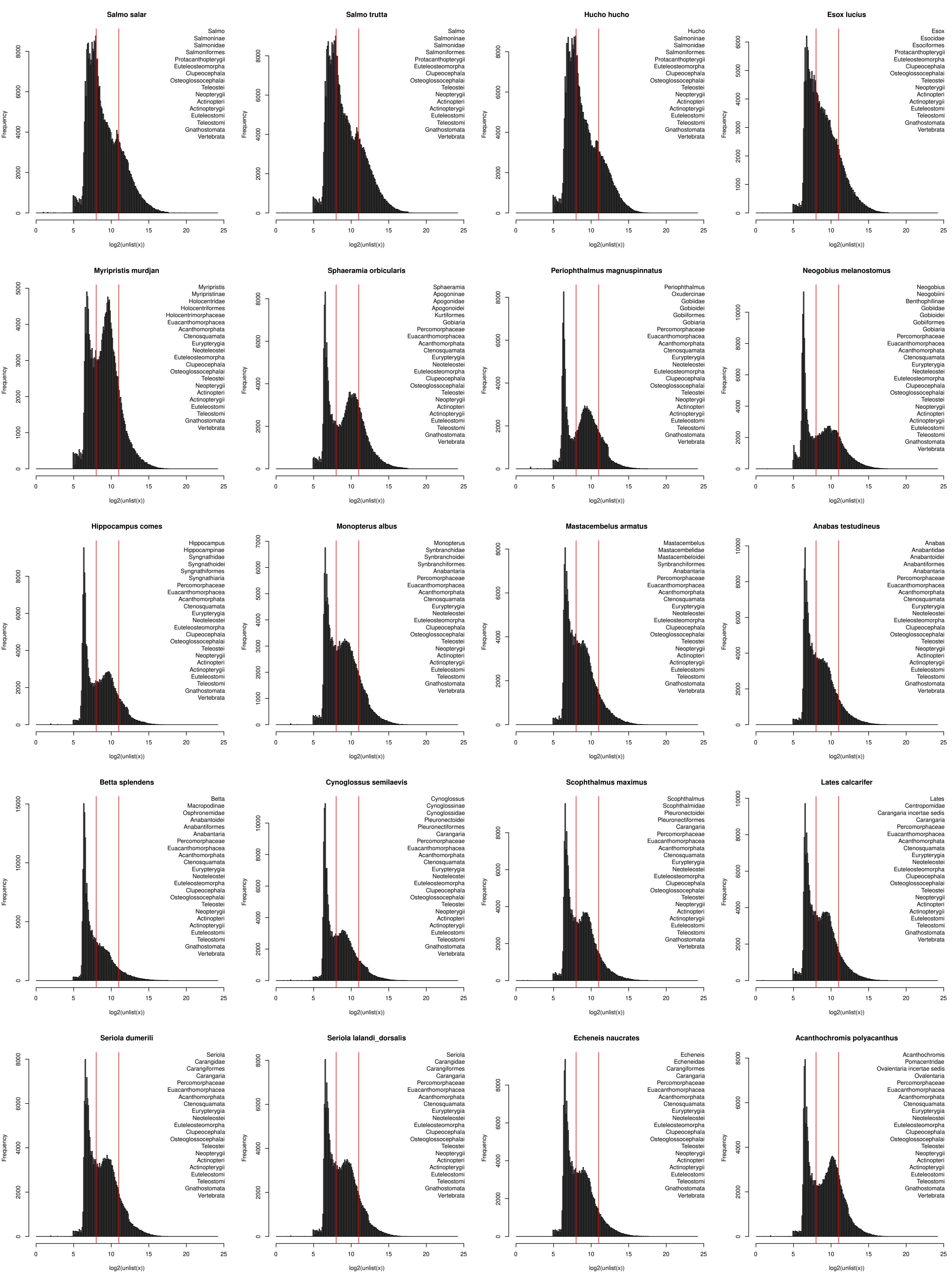

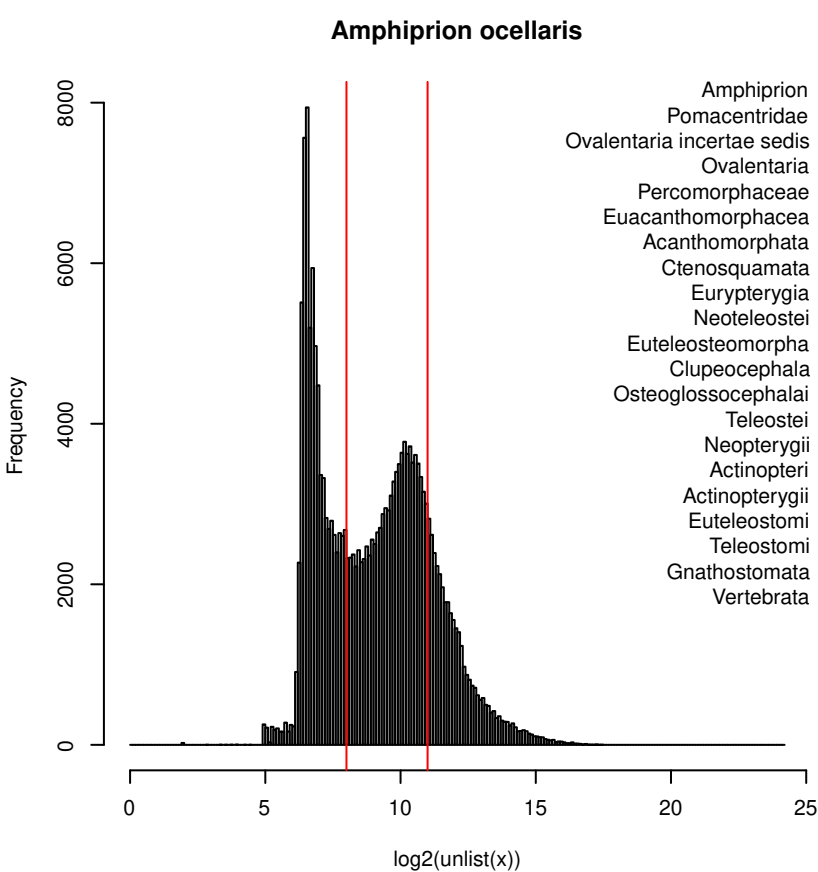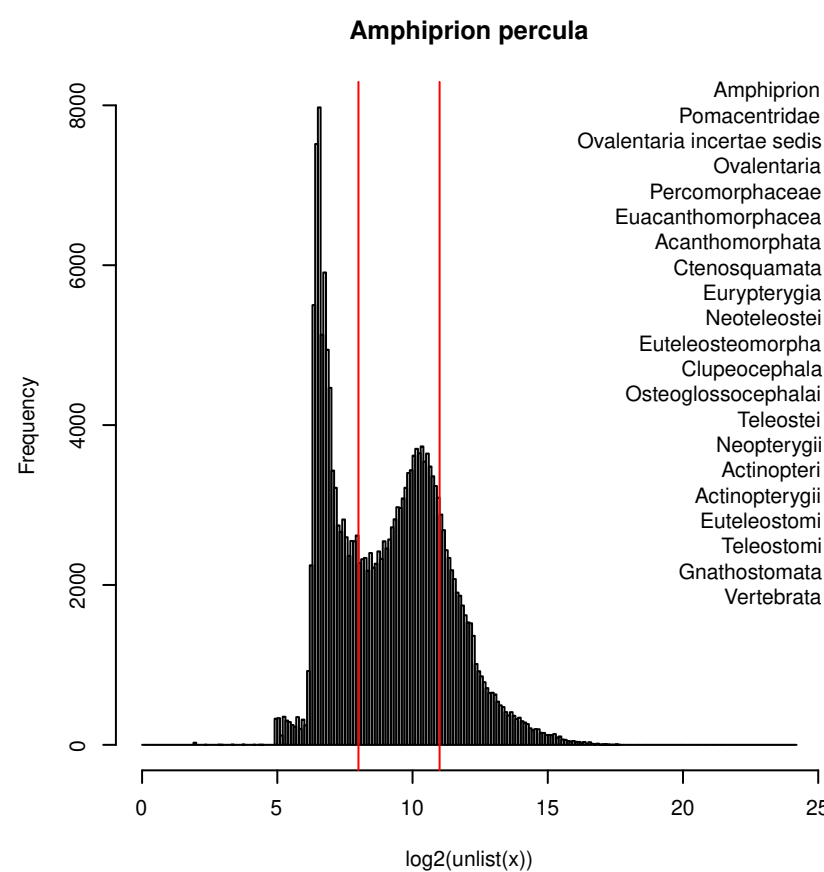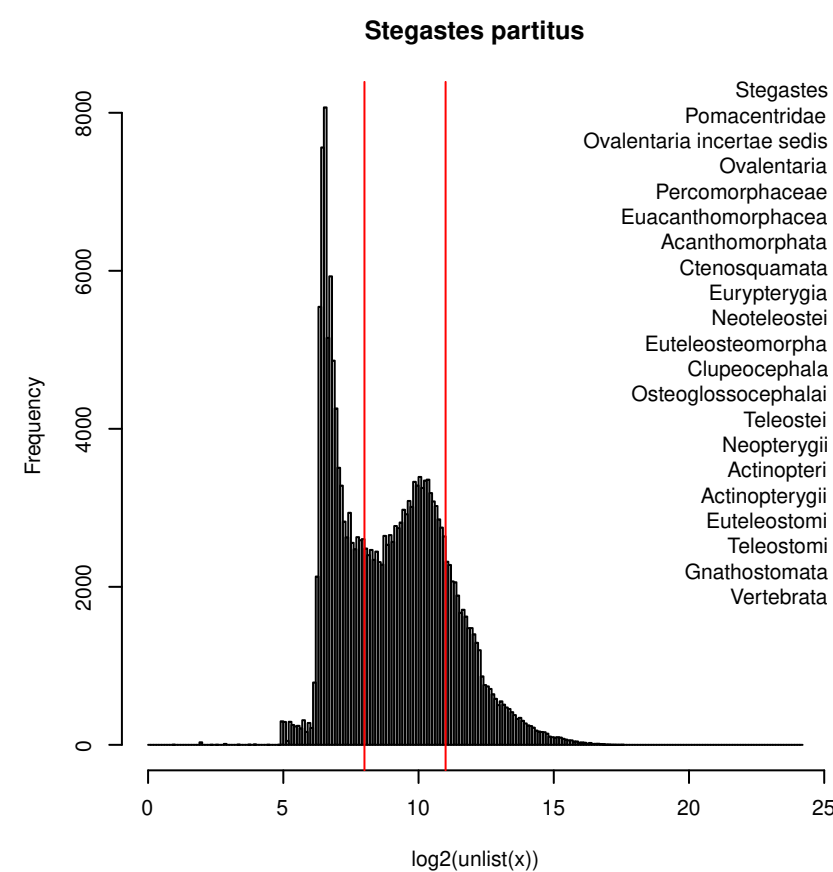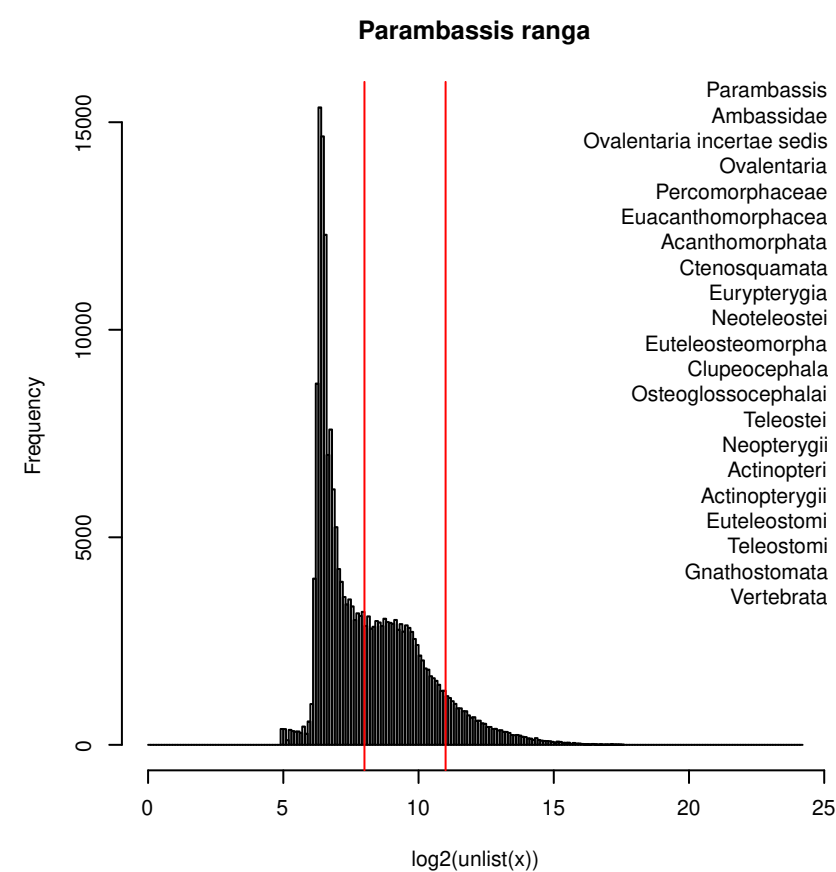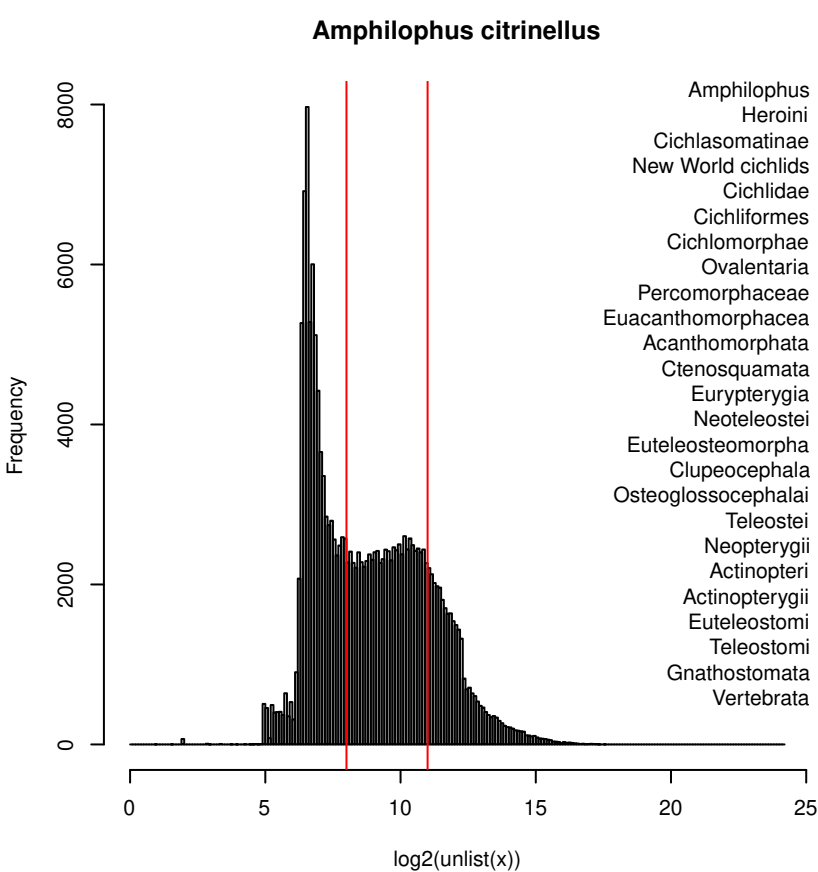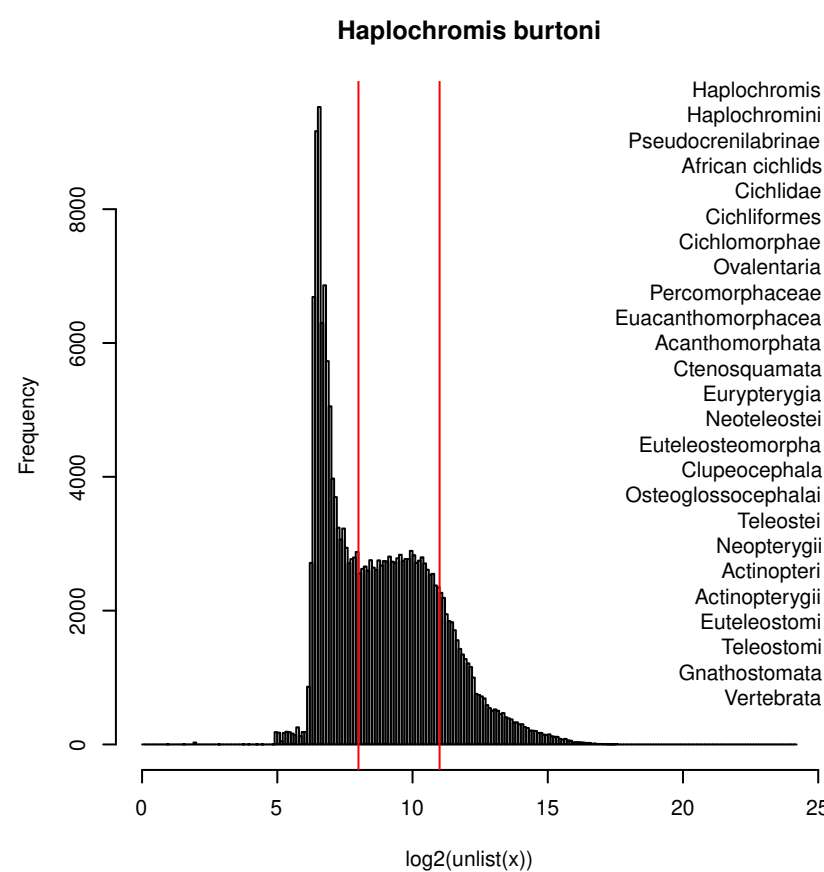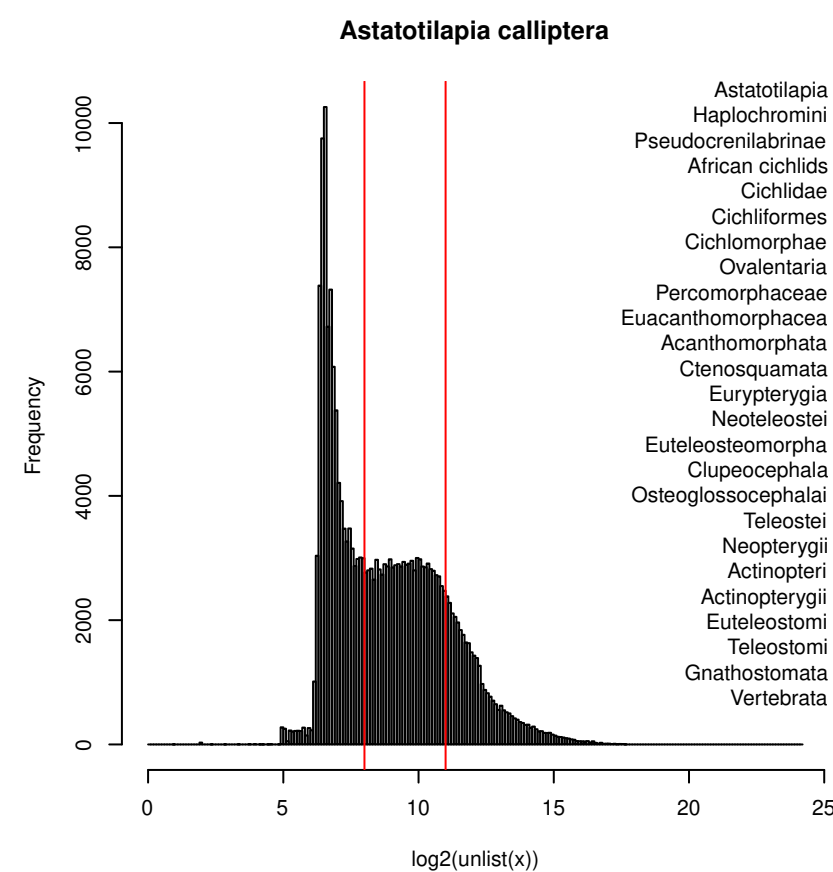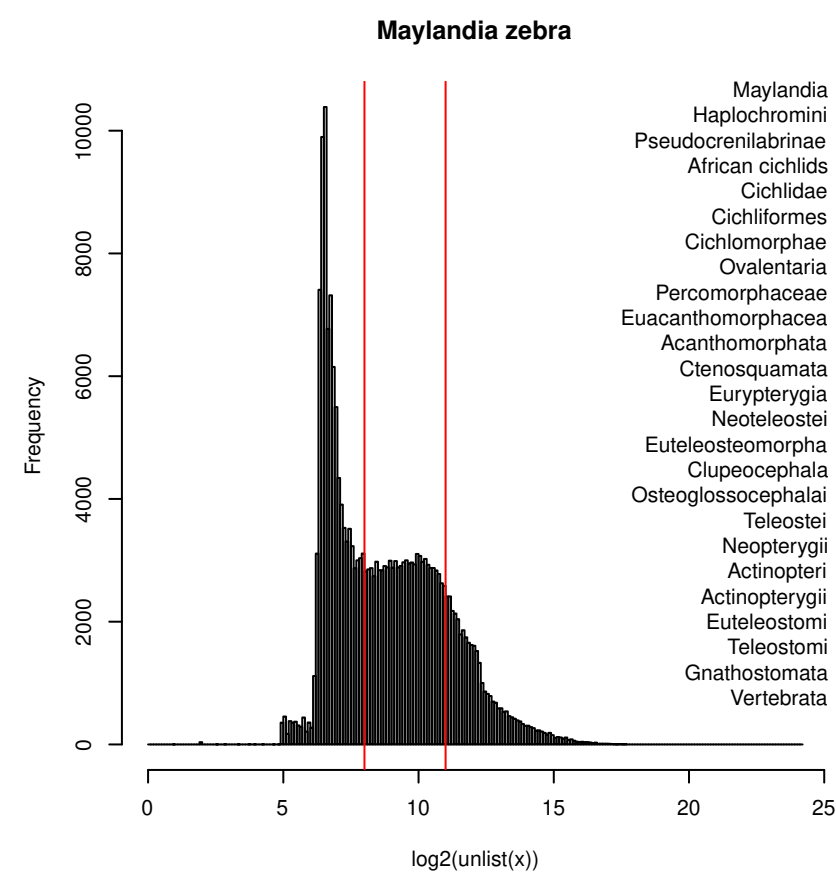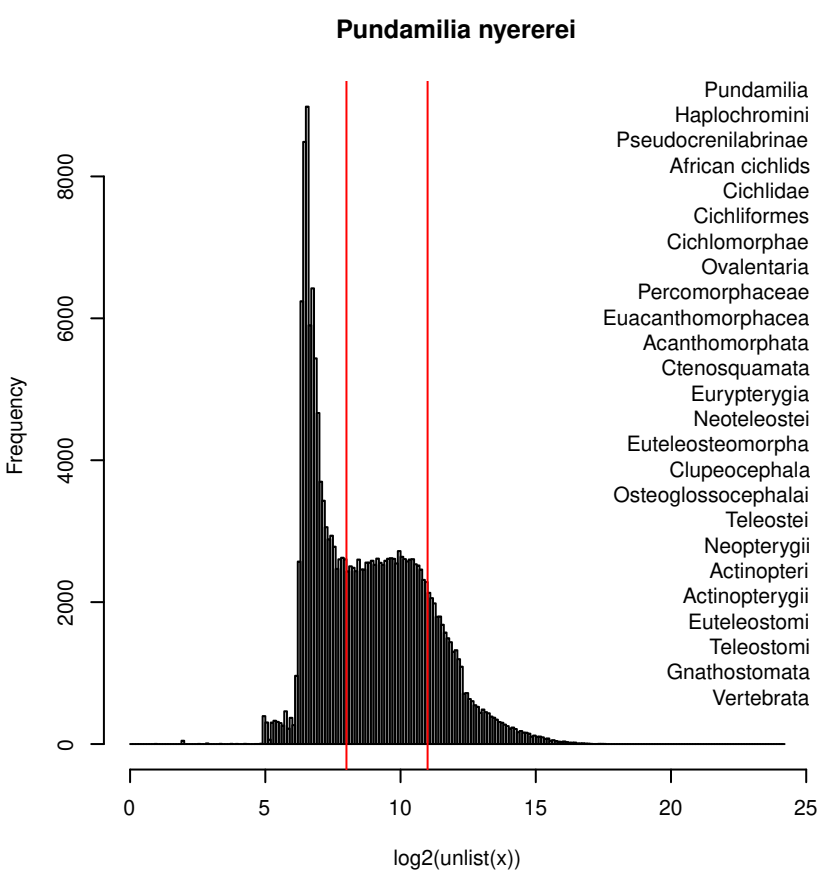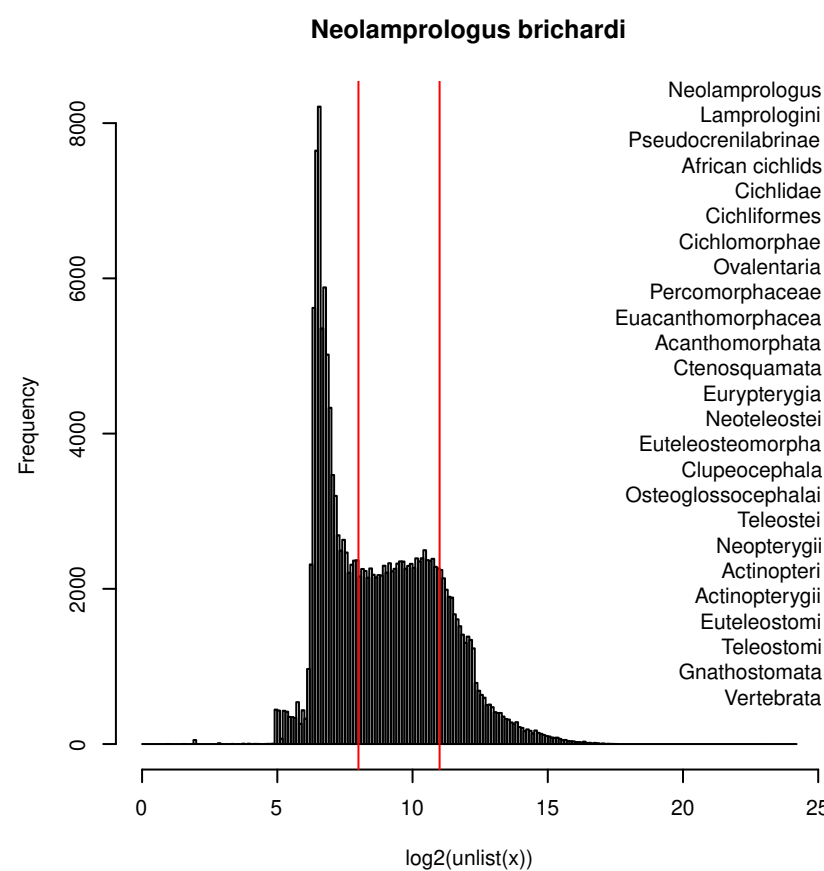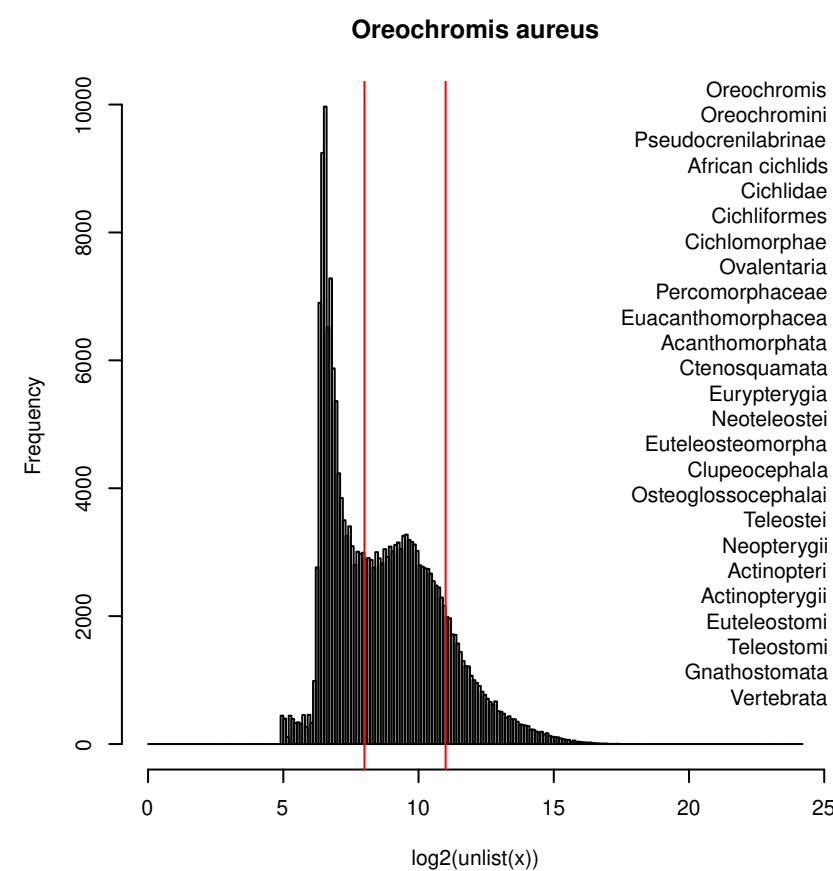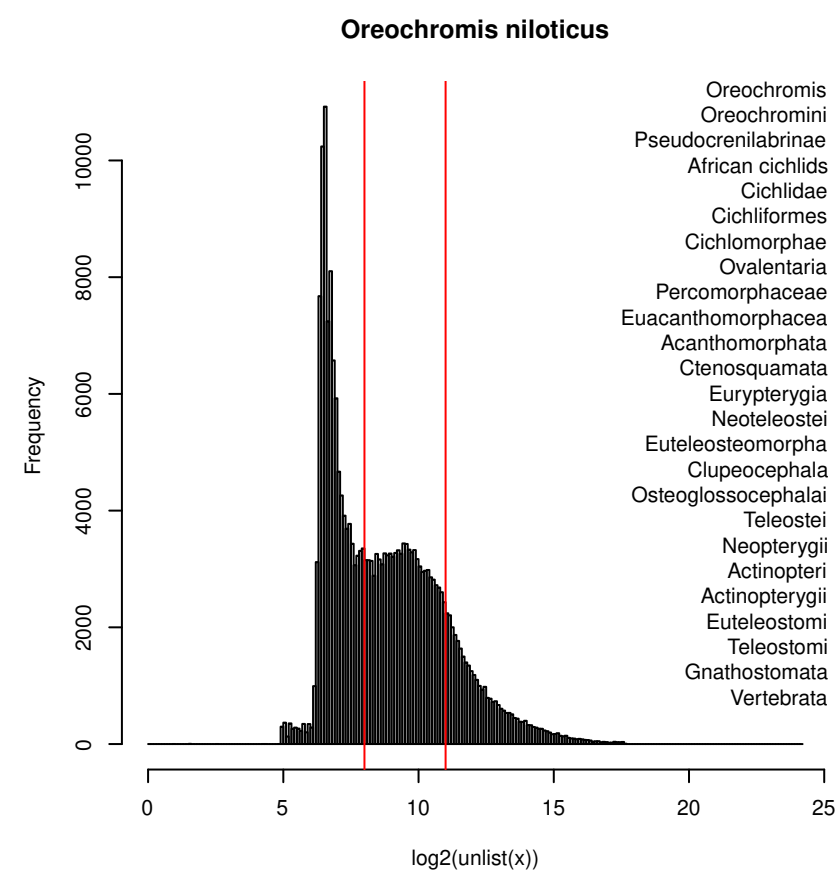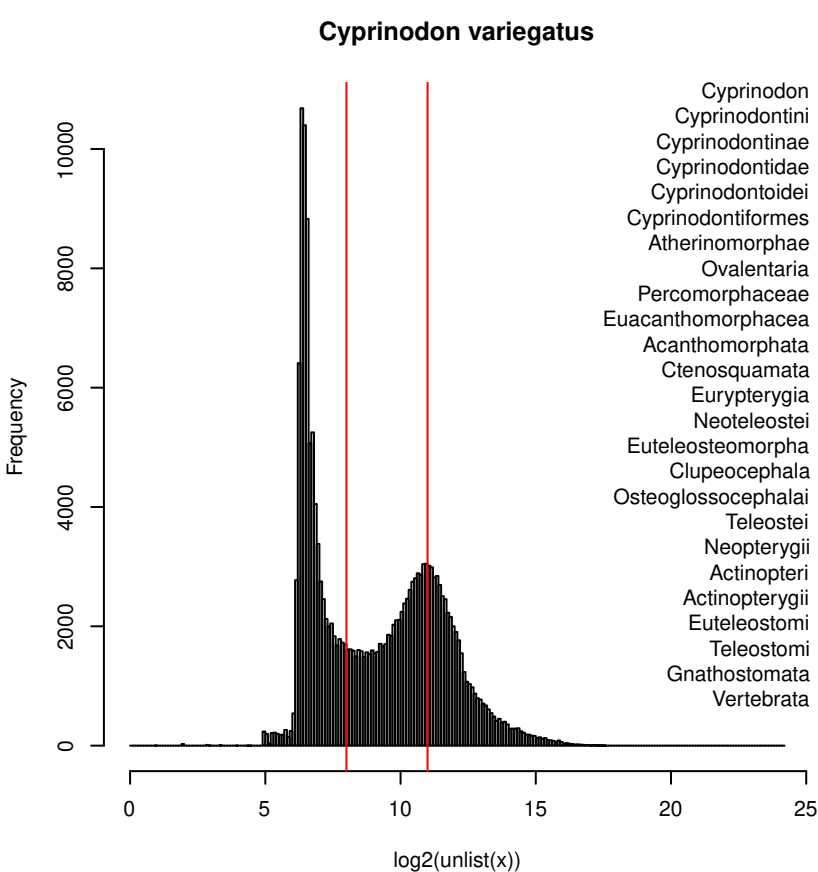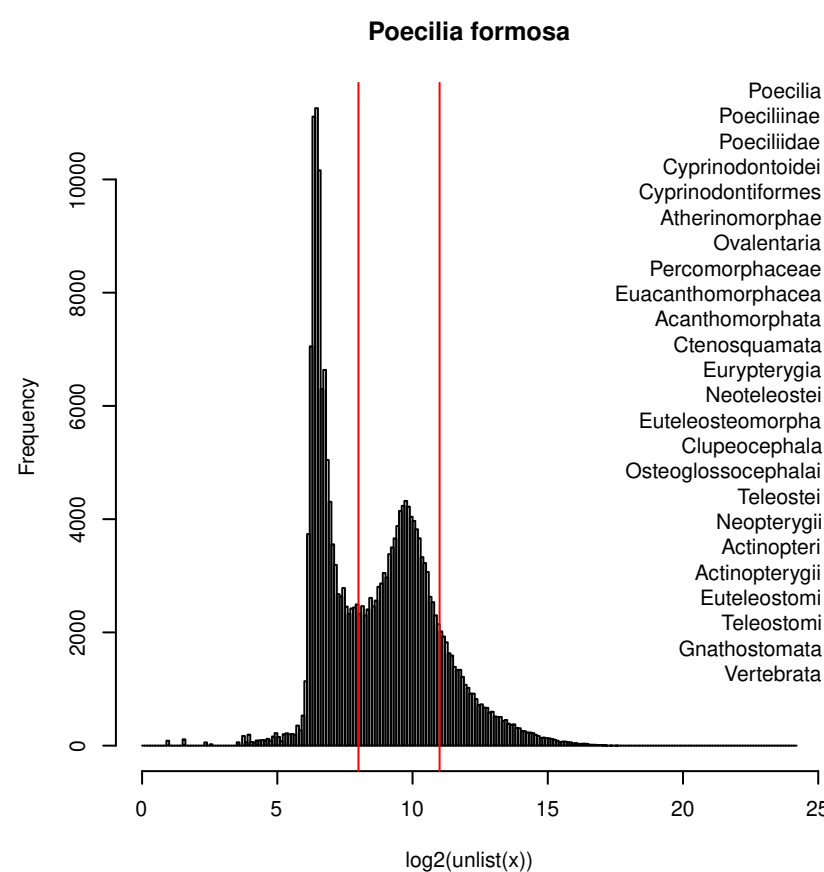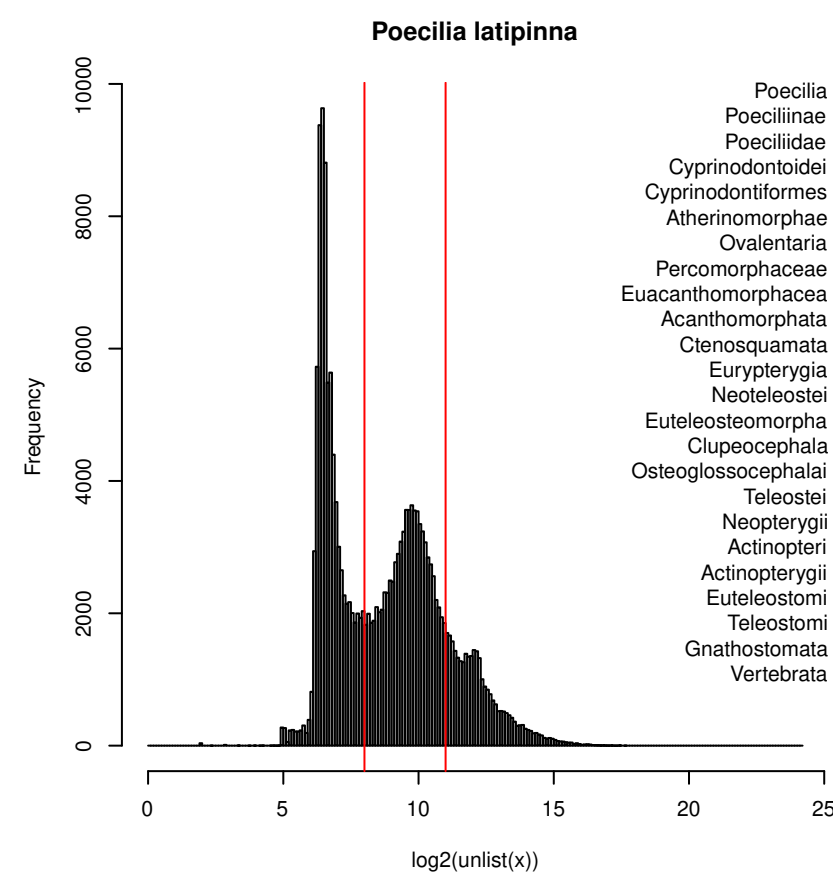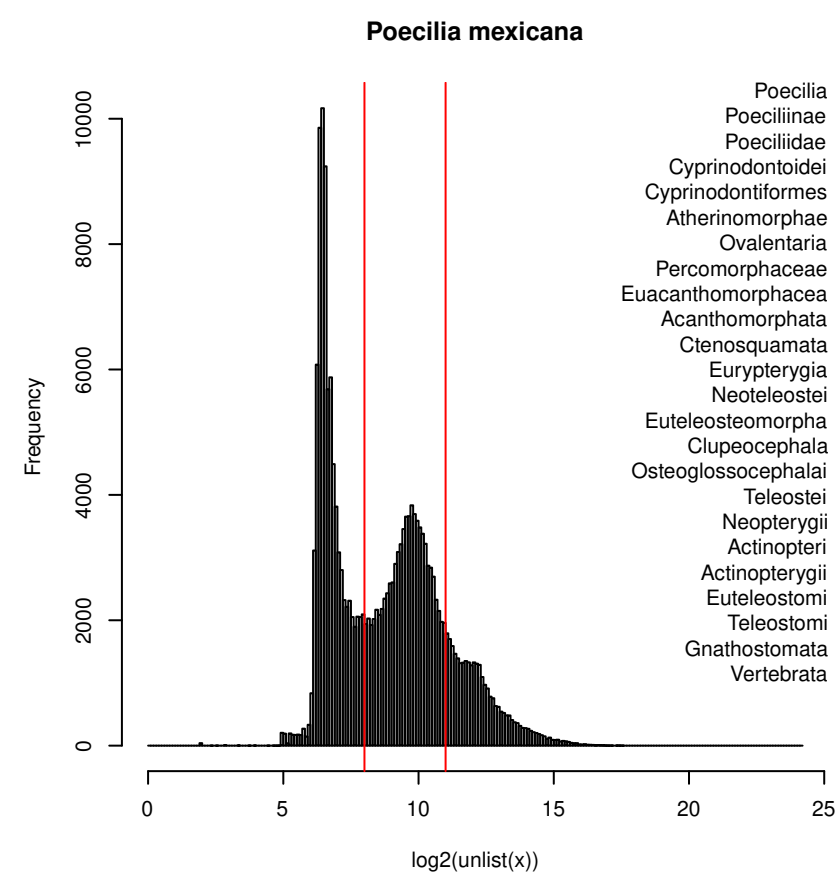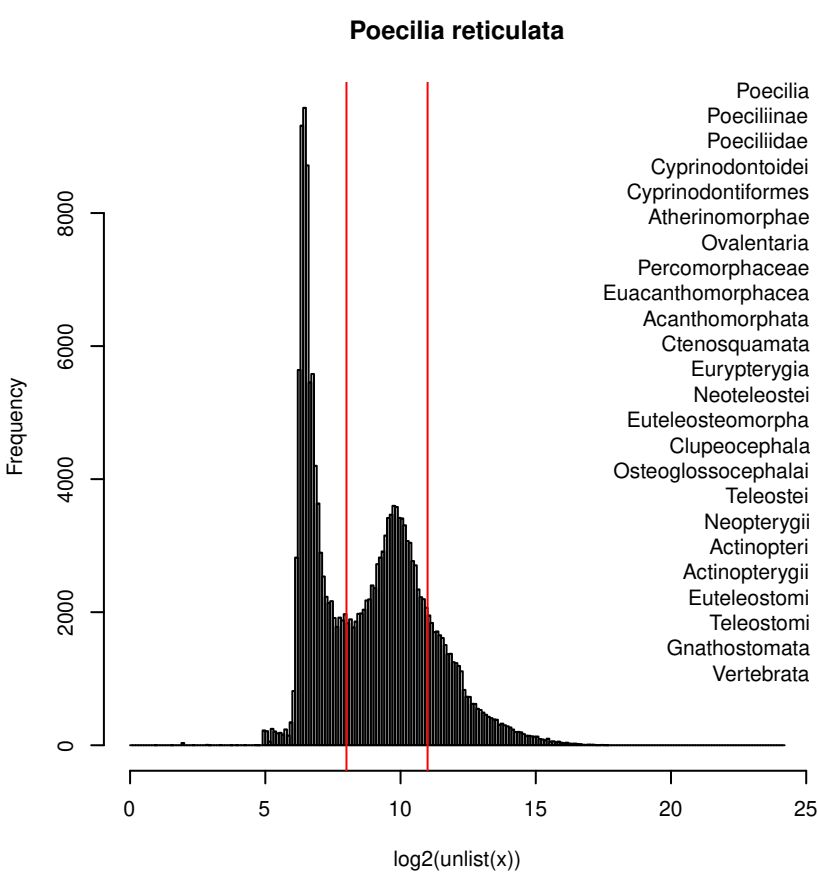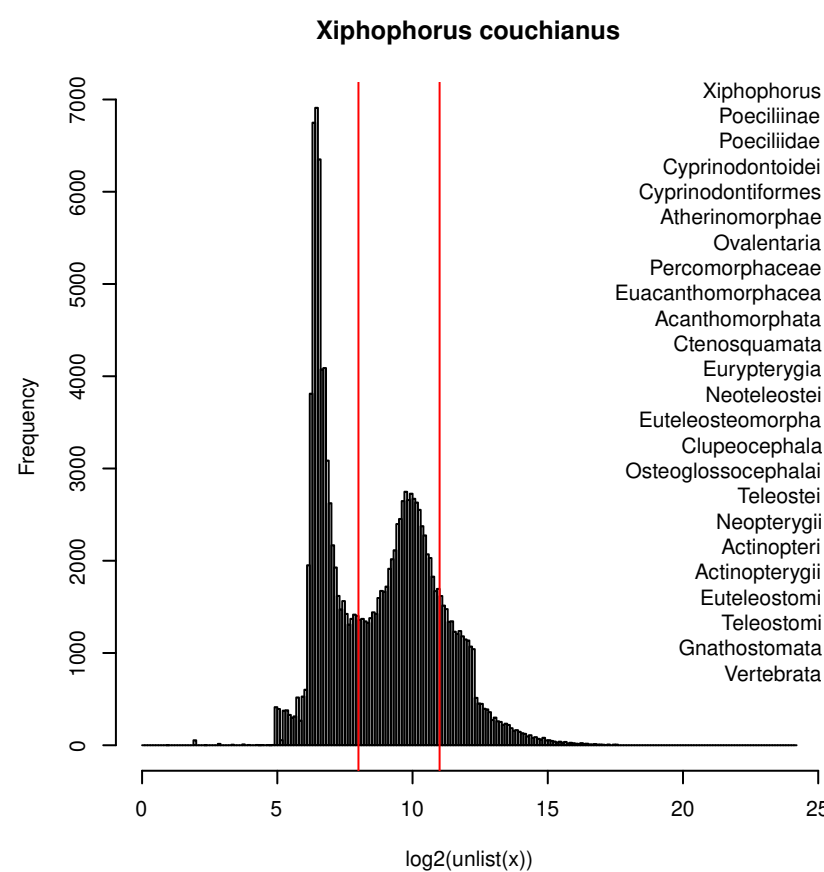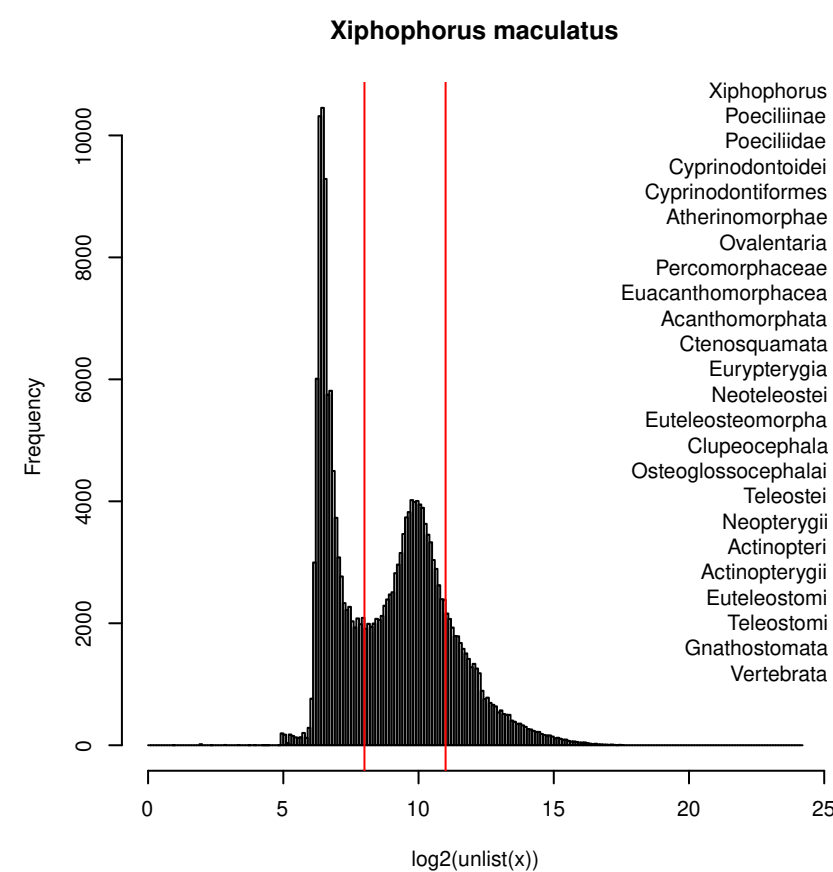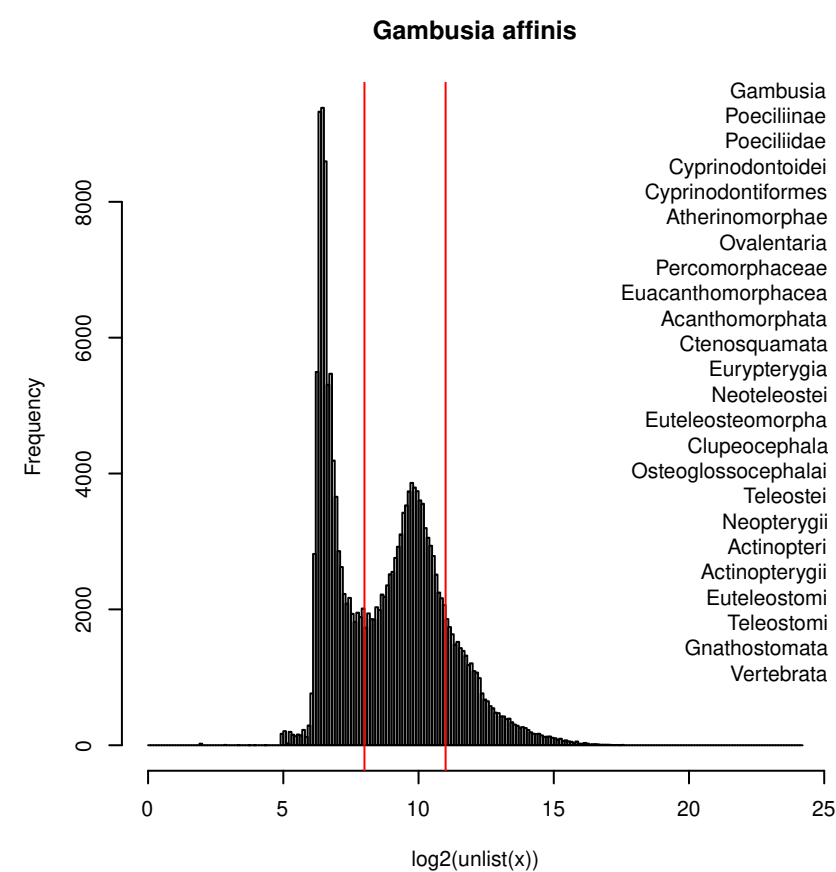

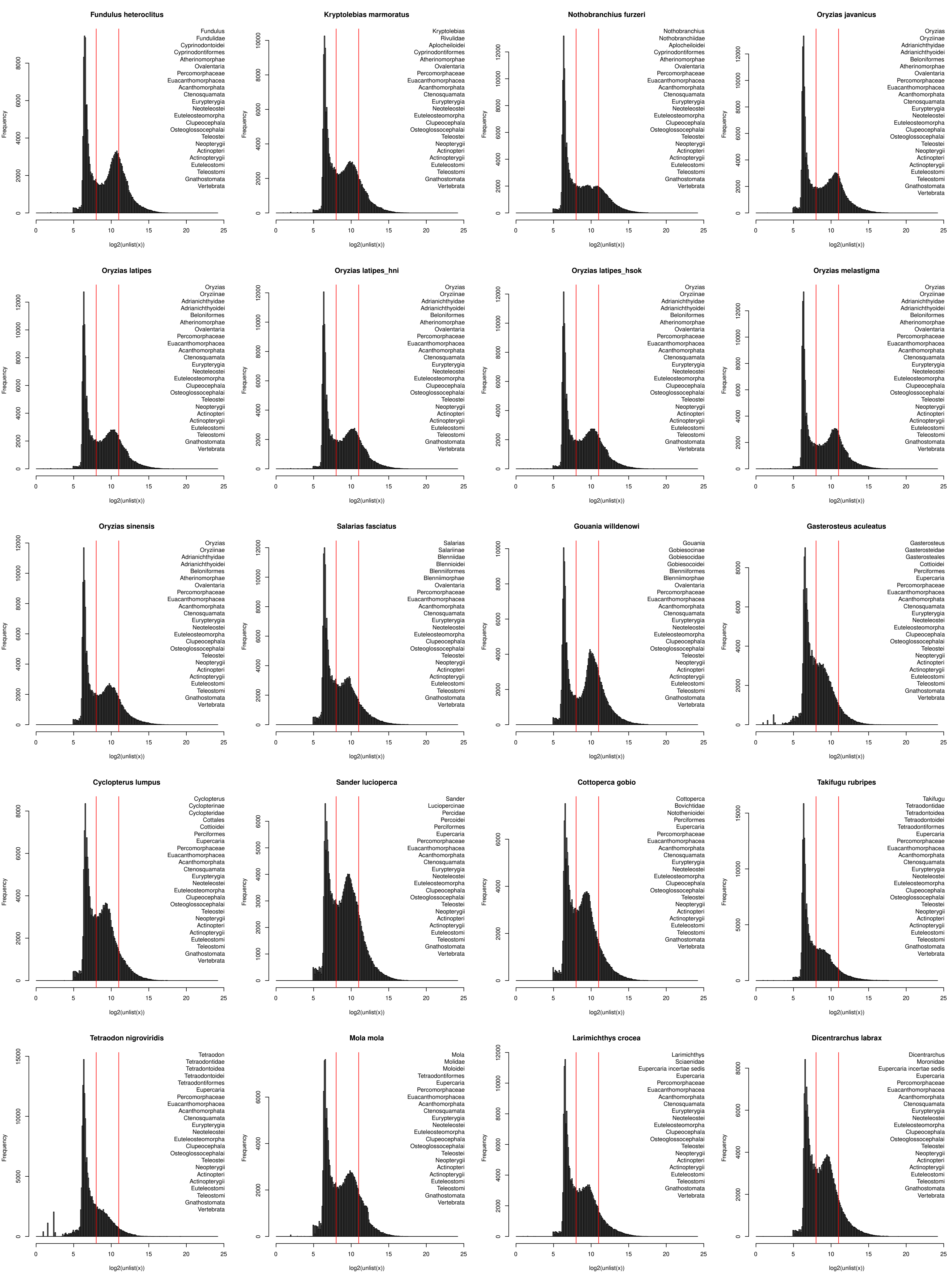

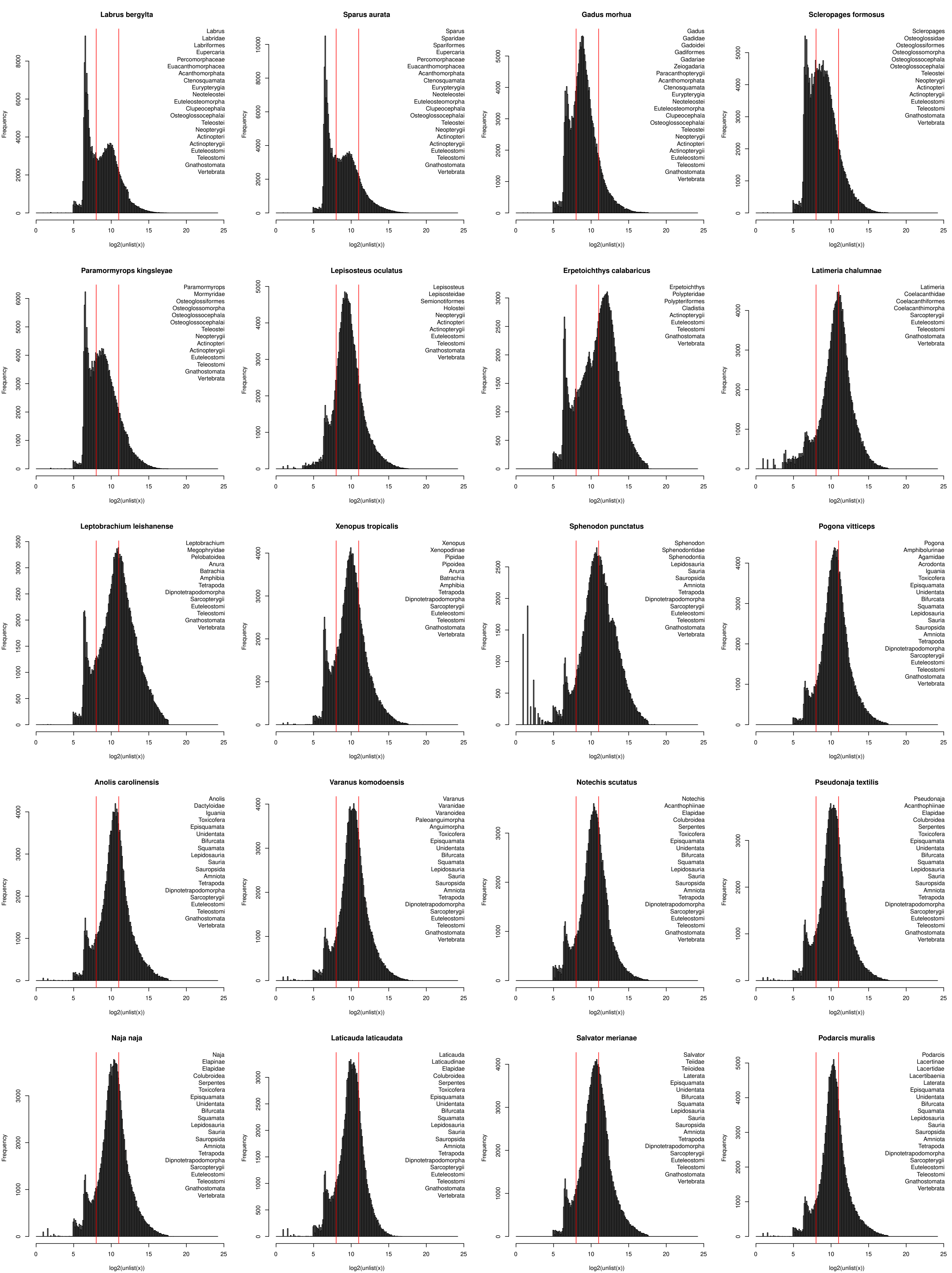

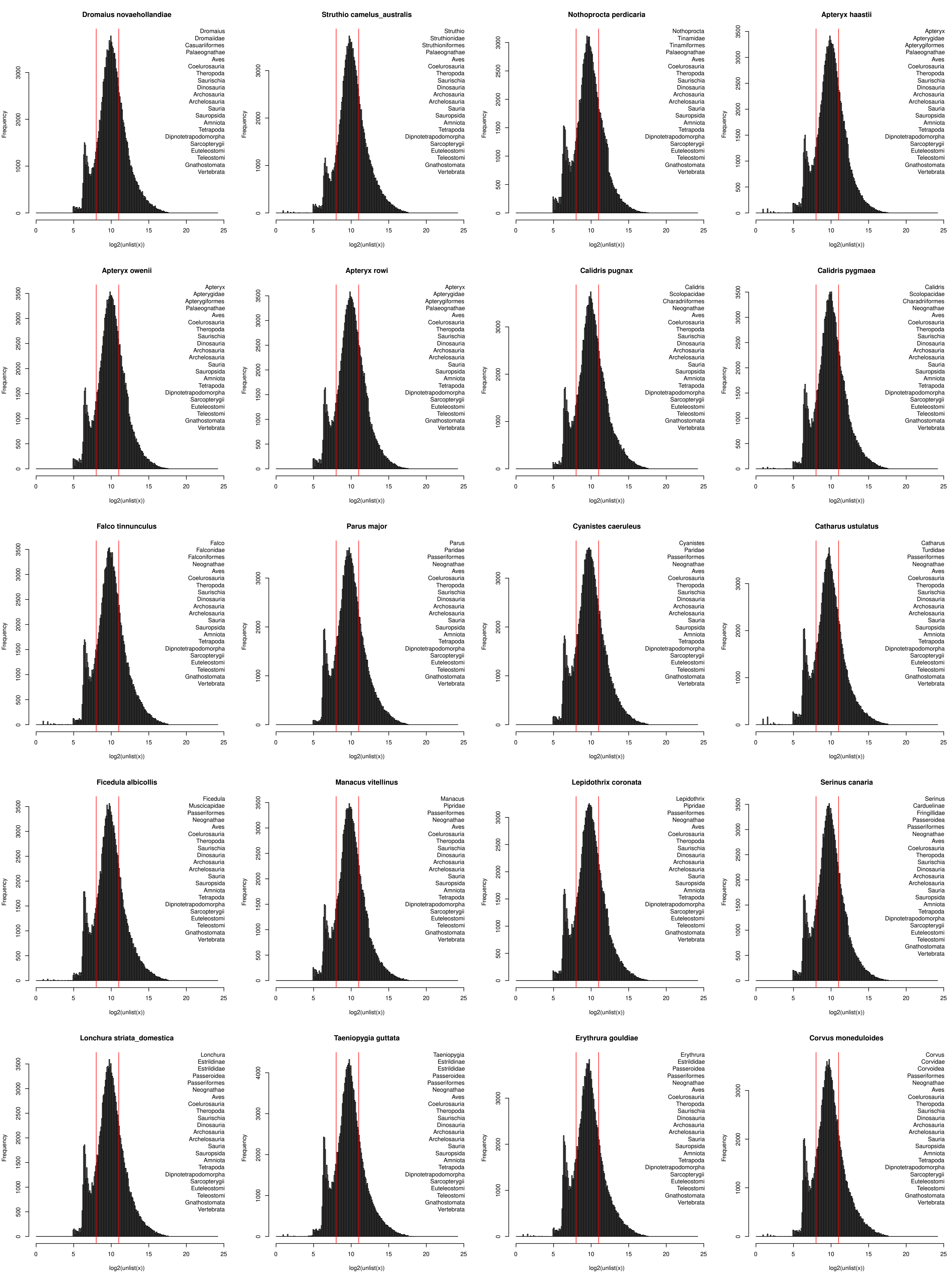

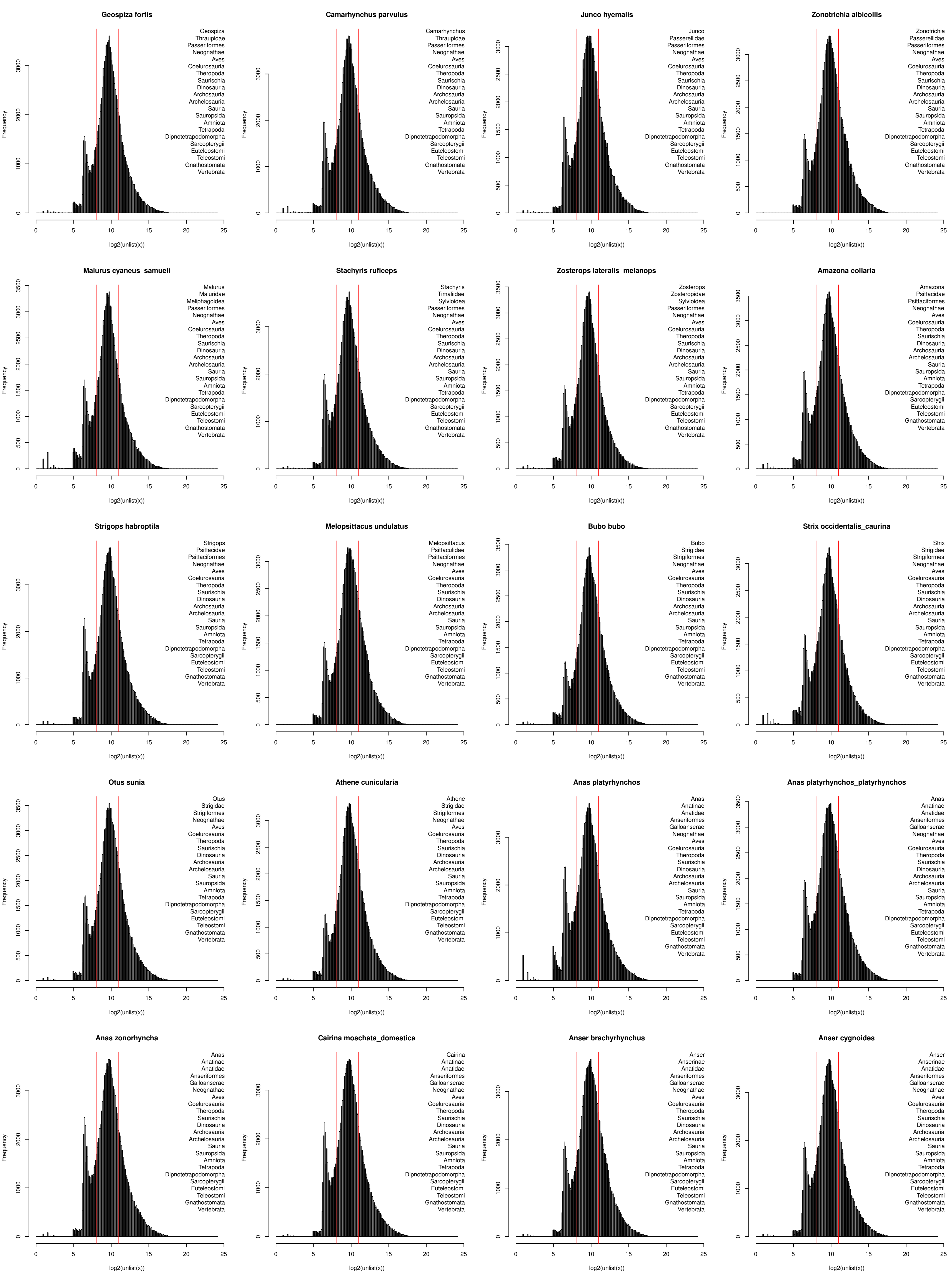

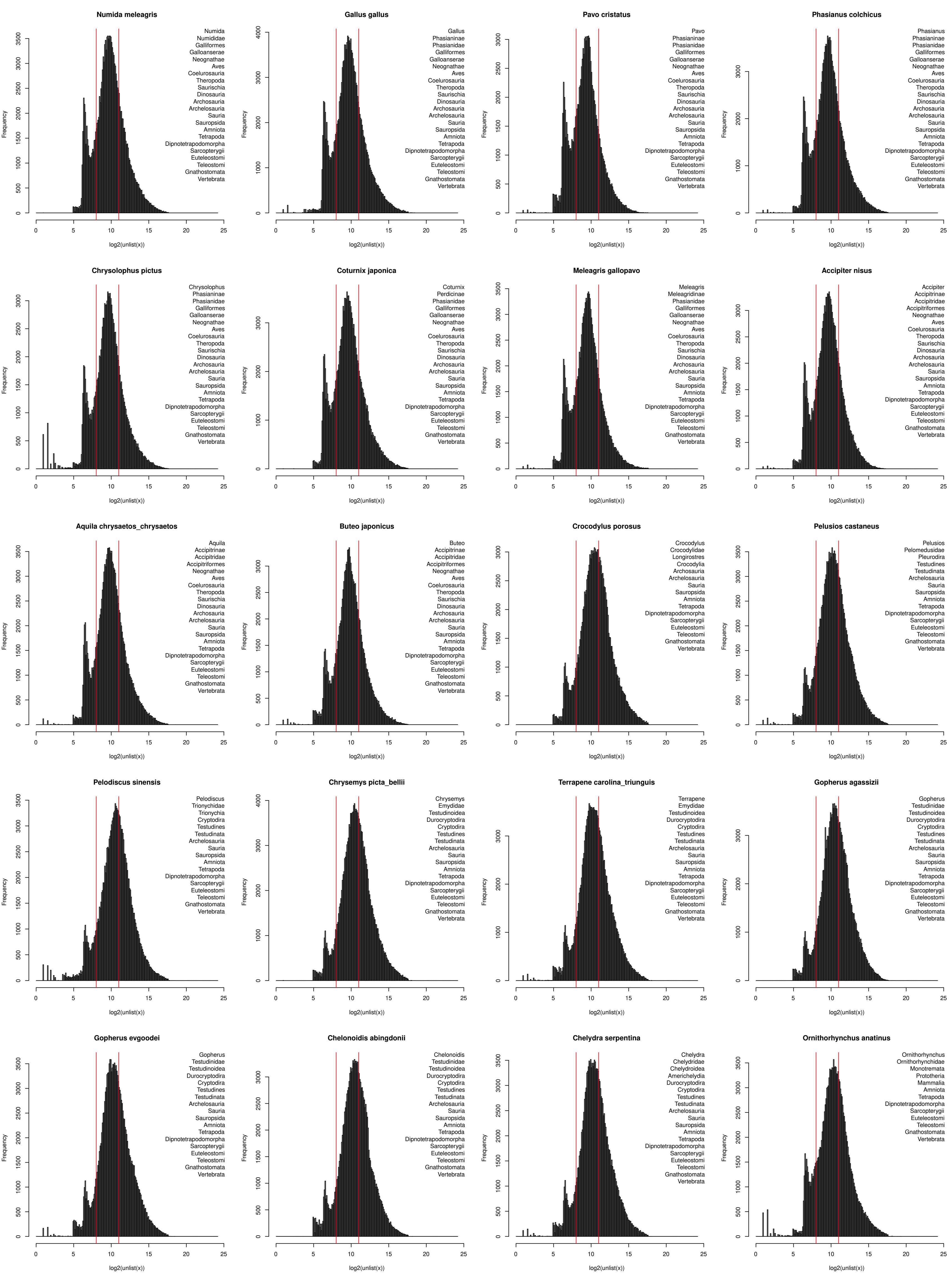

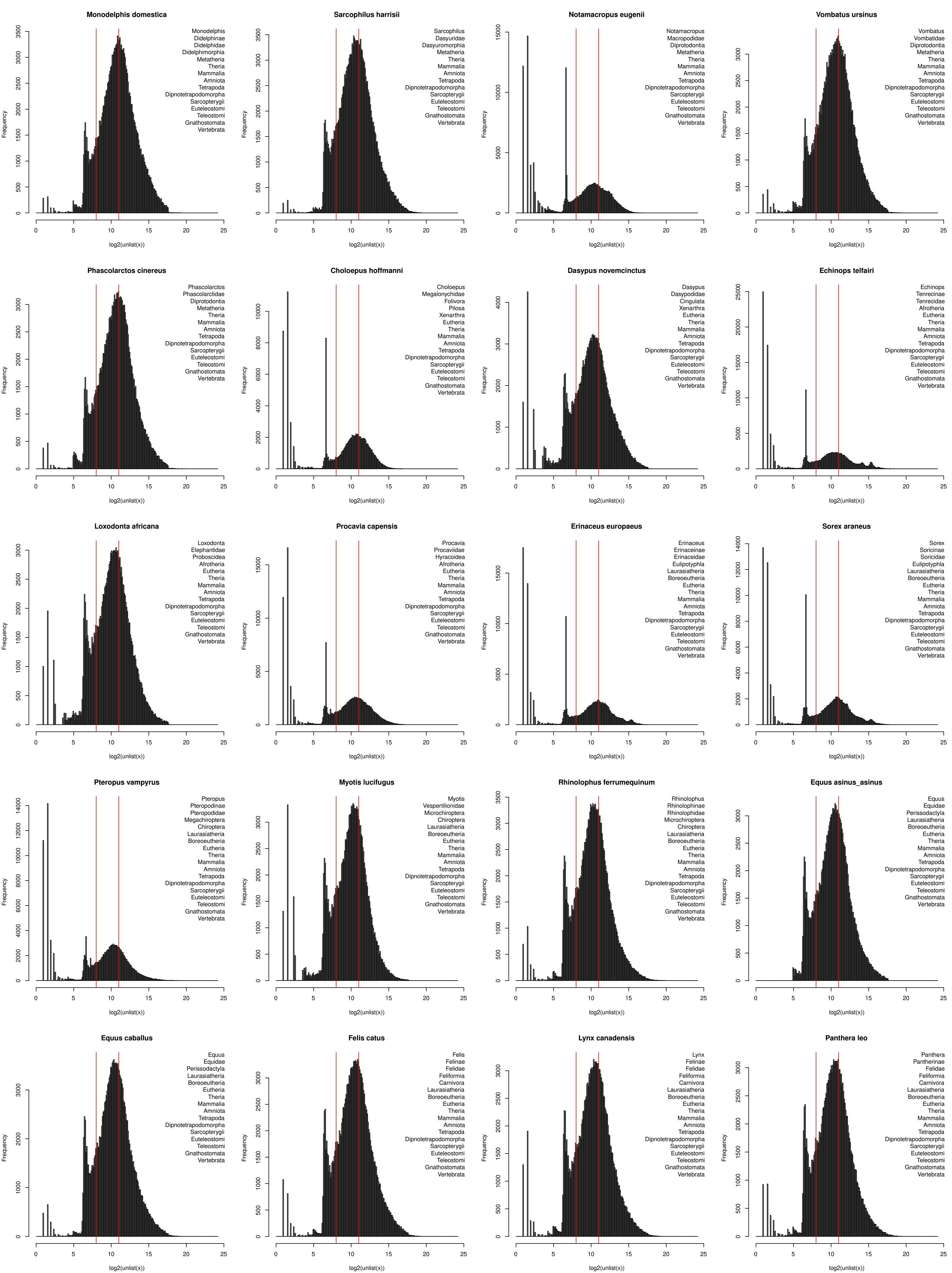

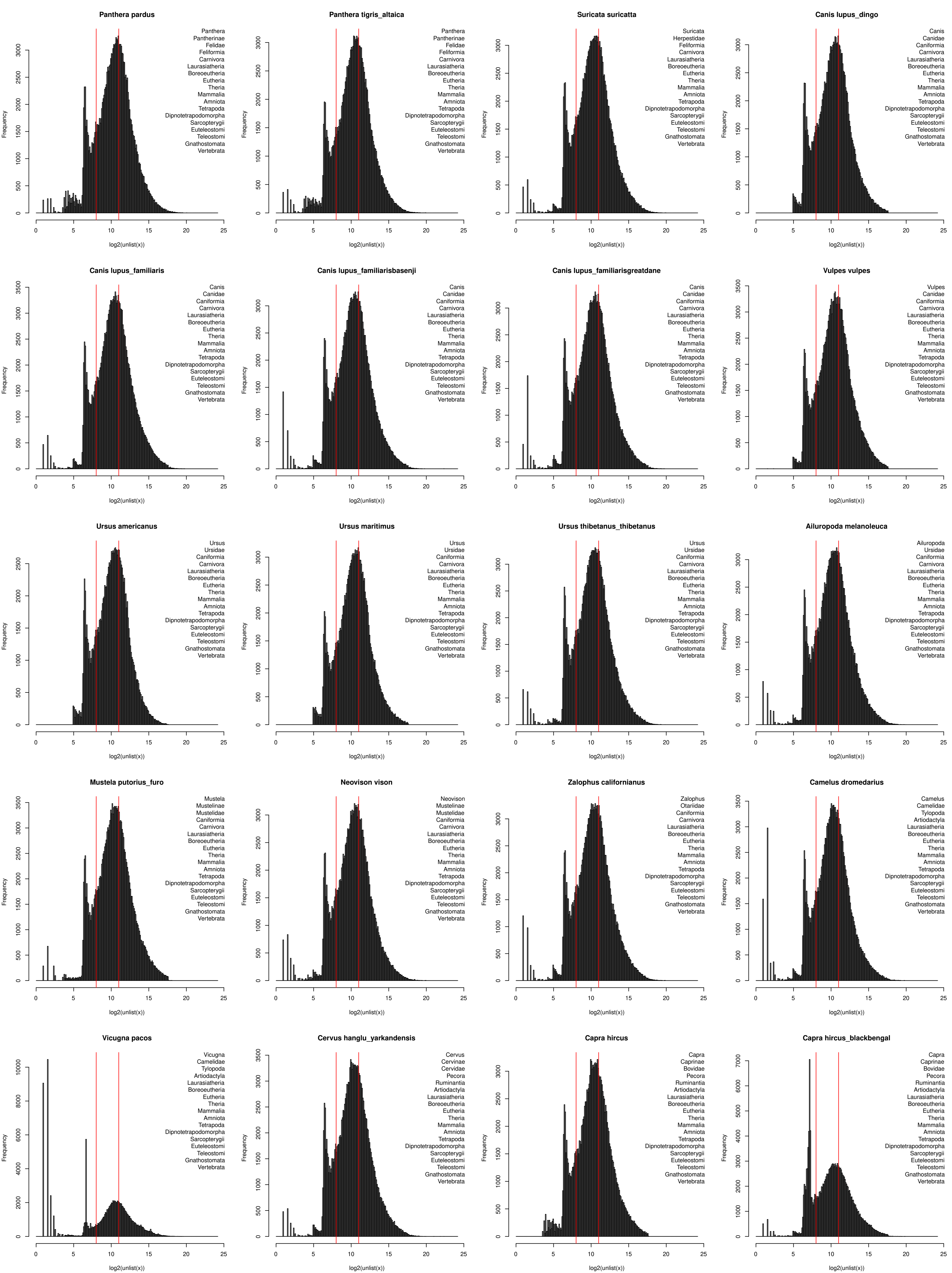

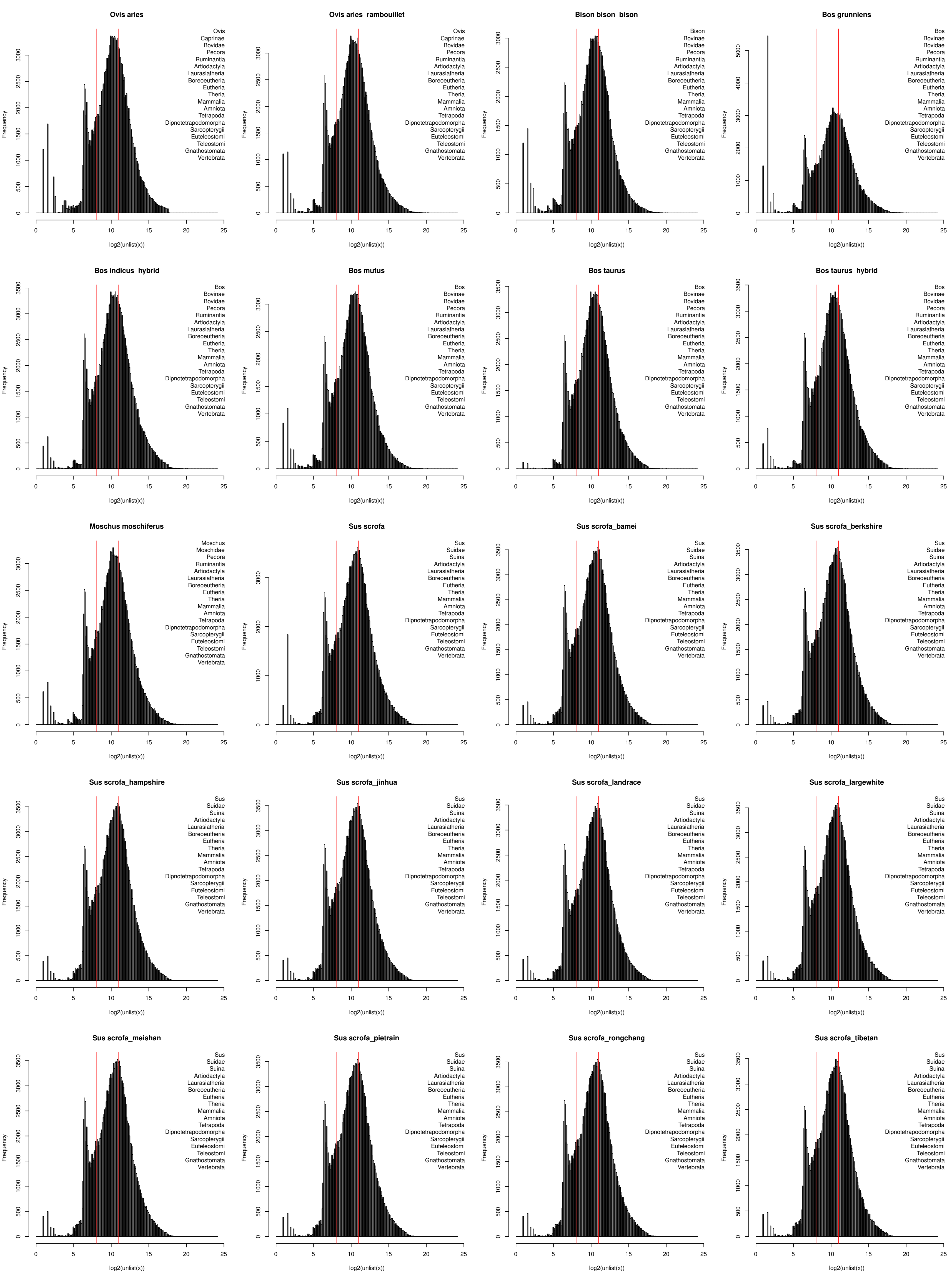

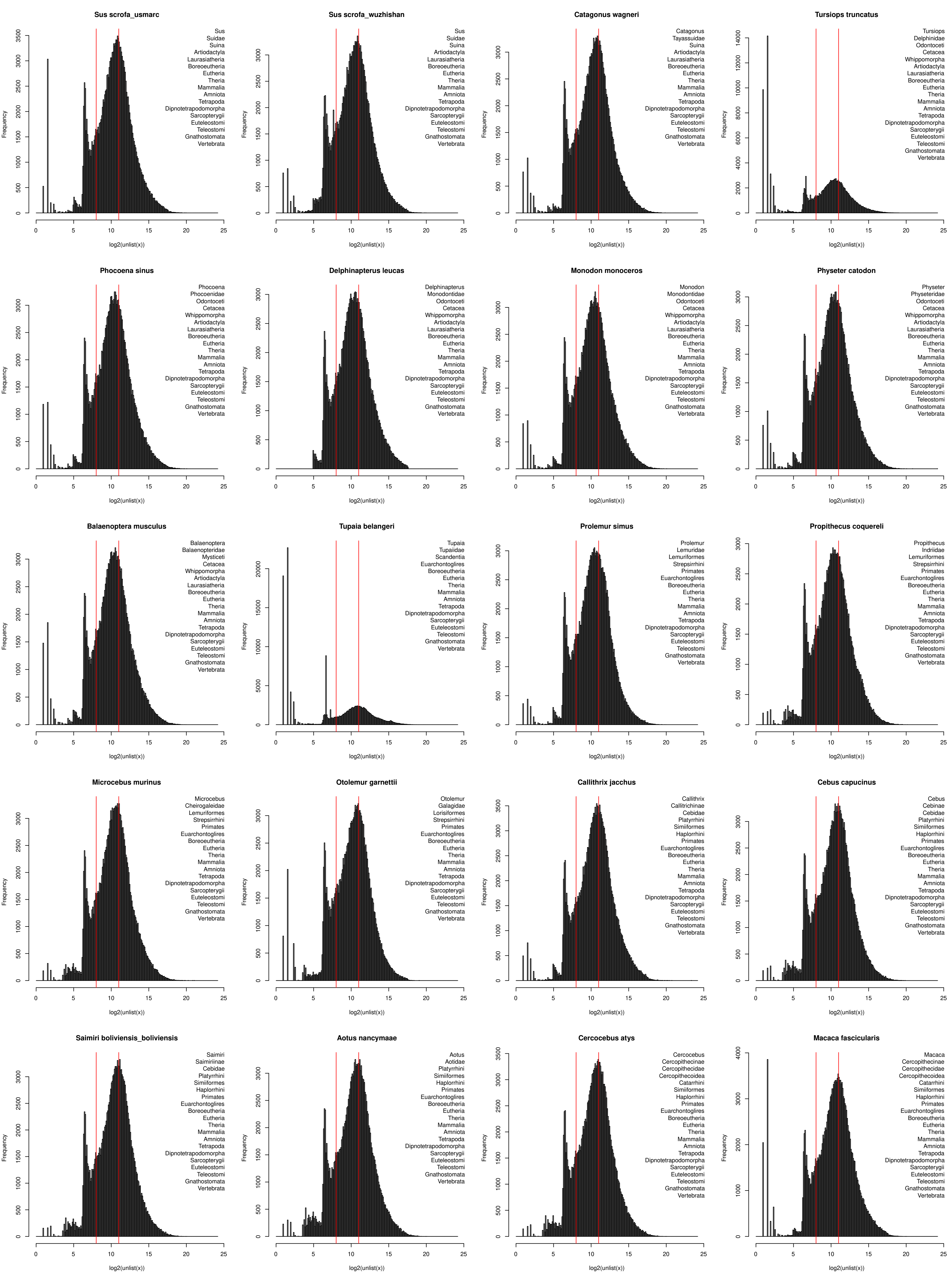

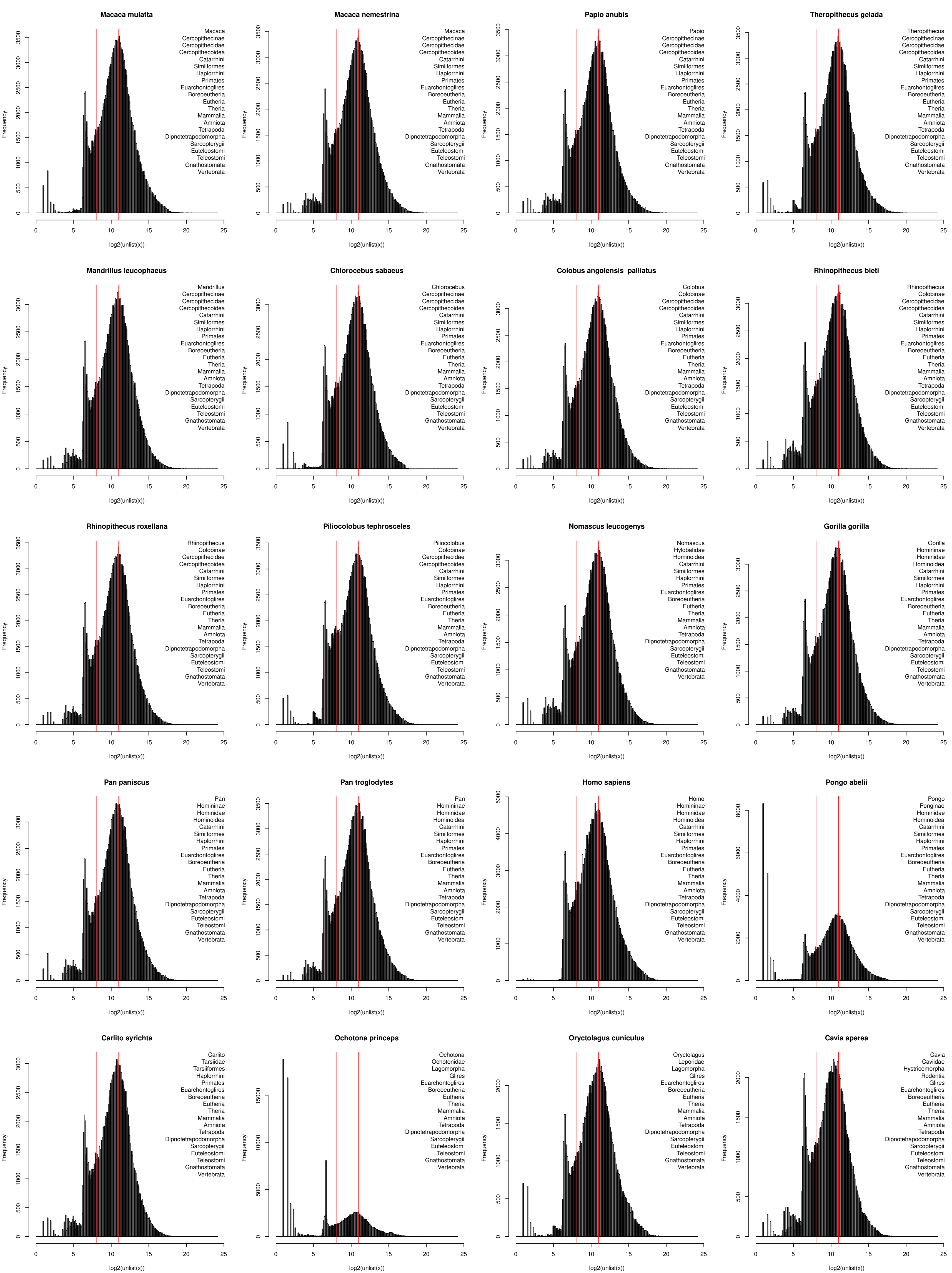

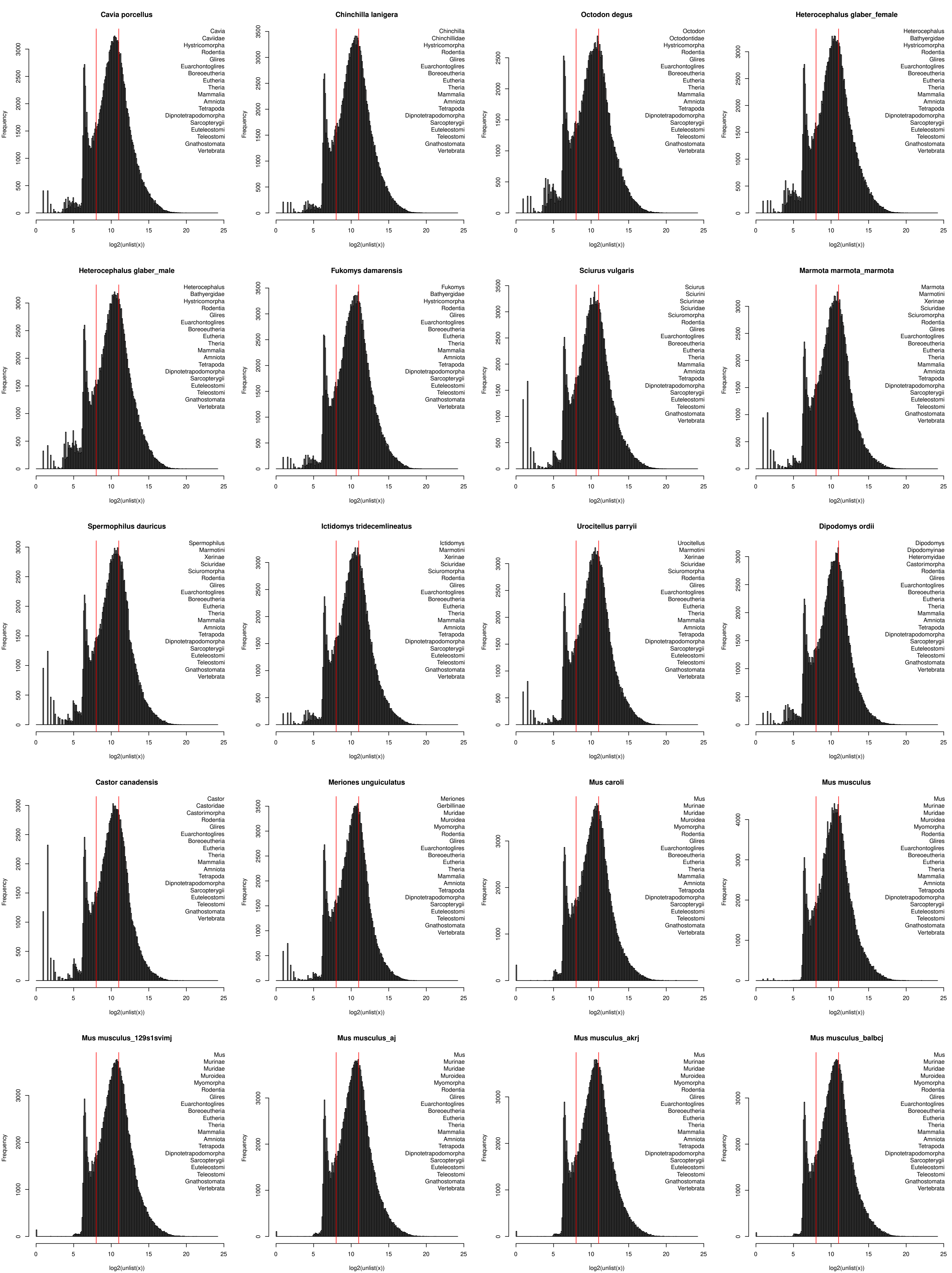

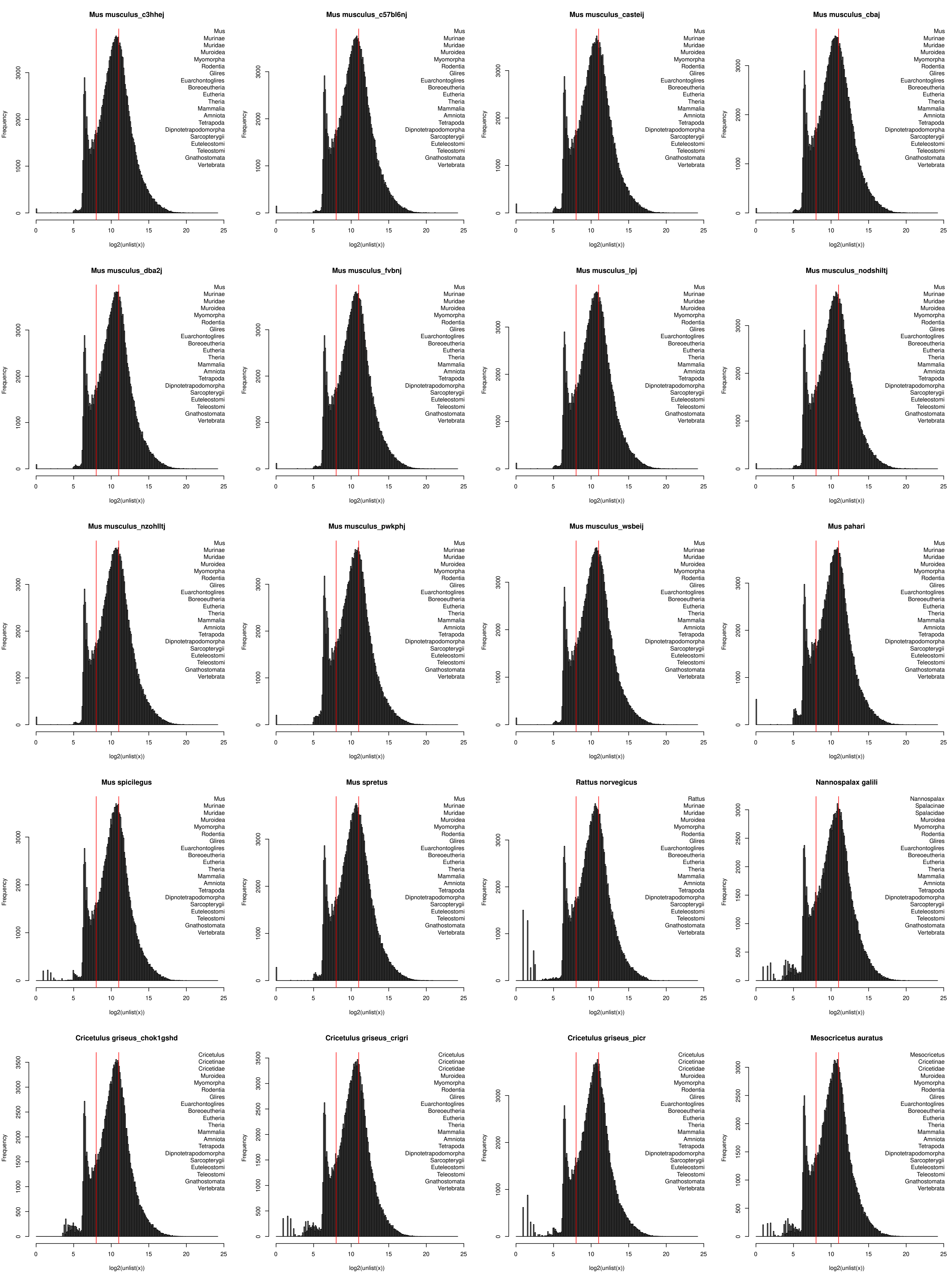

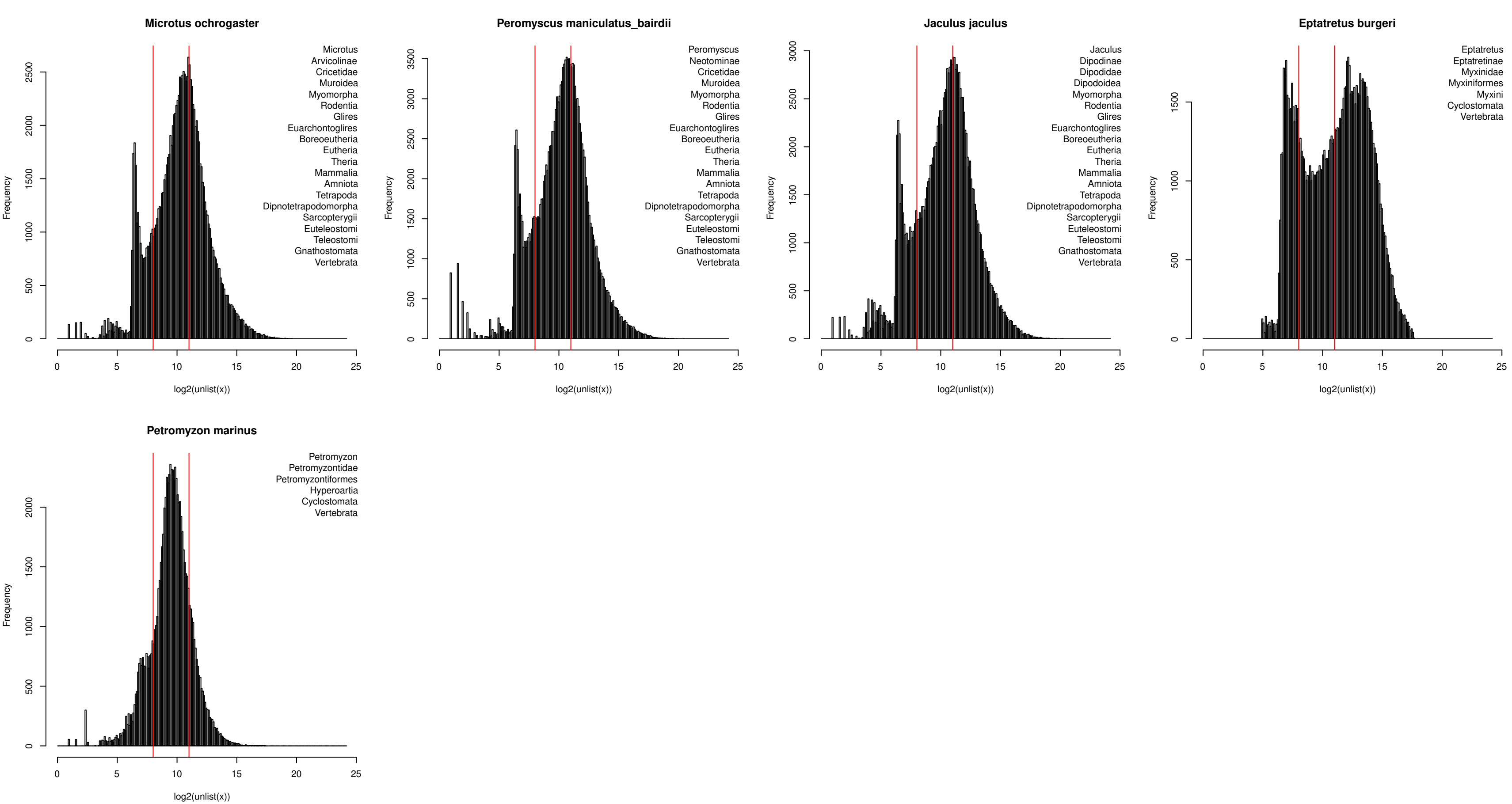

Supplement: Supplementary file 2 — Additional file 2 Vertebrate intron size distributions. log2 transformed intron size distributions for vertebrates taken from Ensembl version 104. The species taxonomic identifcation taken from the NCBI taxonomy is shown on the right for each species. The two vertical red lines indicate the positions of the antimode and peak of larger intron sizes in D. rerio. [file 12864_2022_8760_MOESM2_ESM.pdf]
