# Supplementary material for: Intron size minimisation in teleosts
Source: BMC Genomics. 2022 Sep 1;23:628. doi: 10.1186/s12864-022-08760-w (PMC9438311; doi:10.1186/s12864-022-08760-w)

GO:0048731 system development

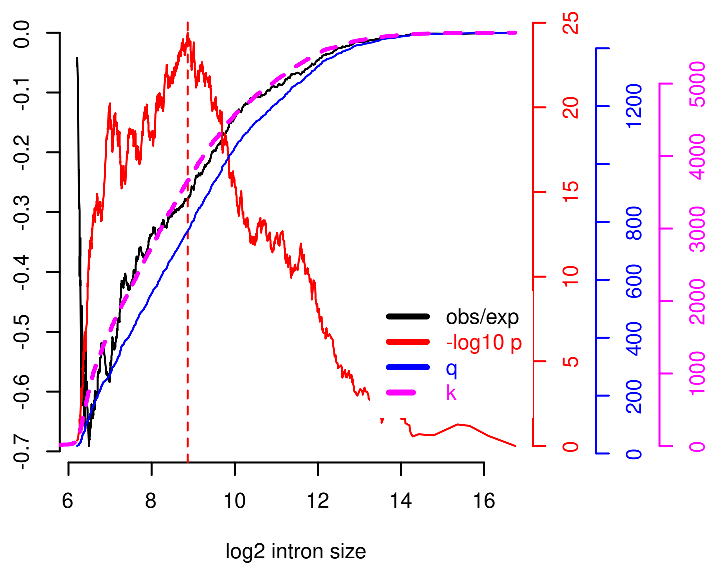

GO:0032501 multicellular organismal process

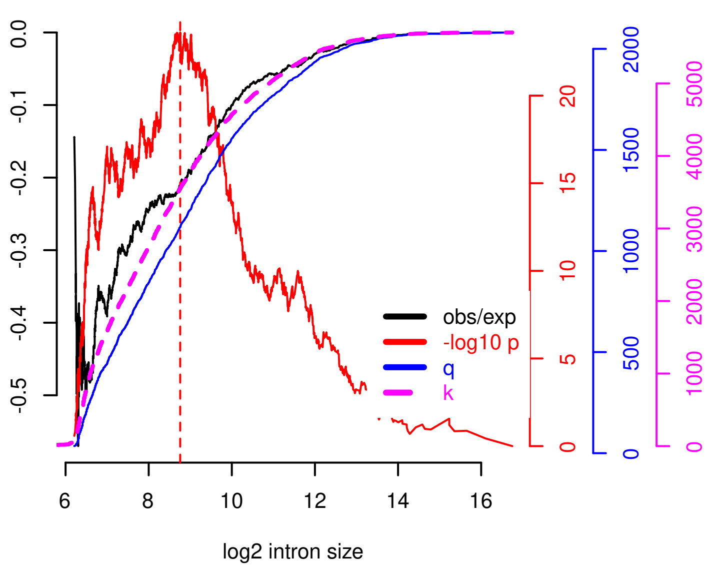

GO:0048856 anatomical structure development

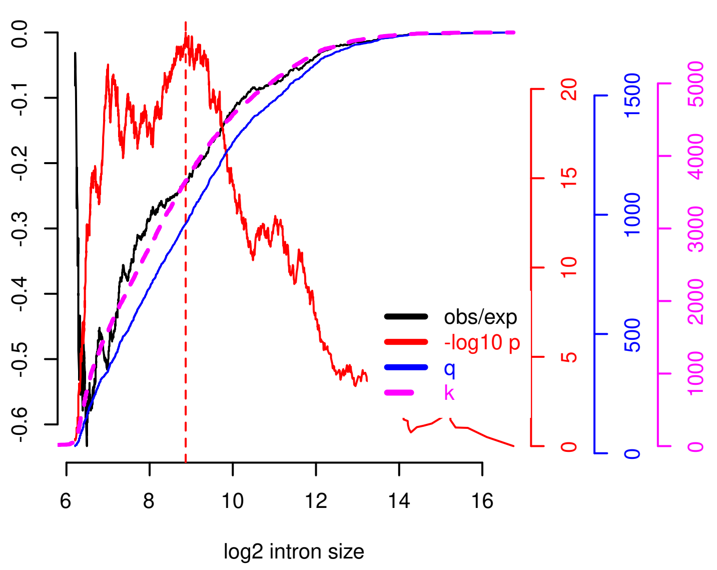

GO:0007275 multicellular organism development

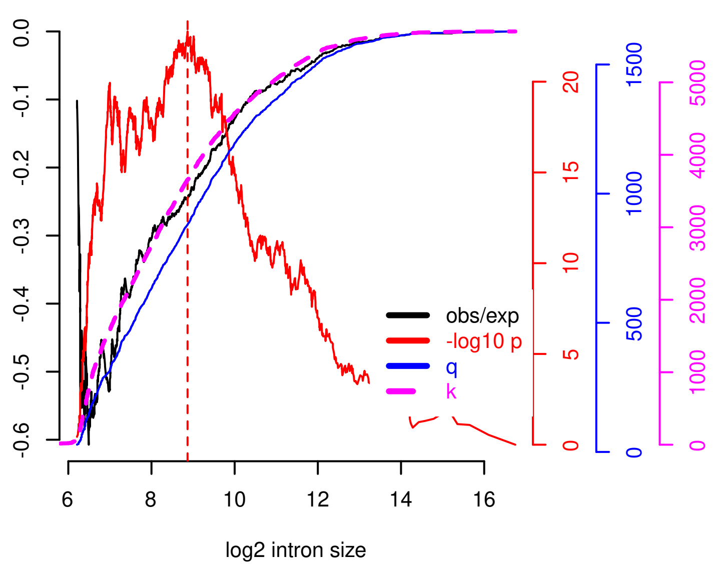

GO:0023052 signaling

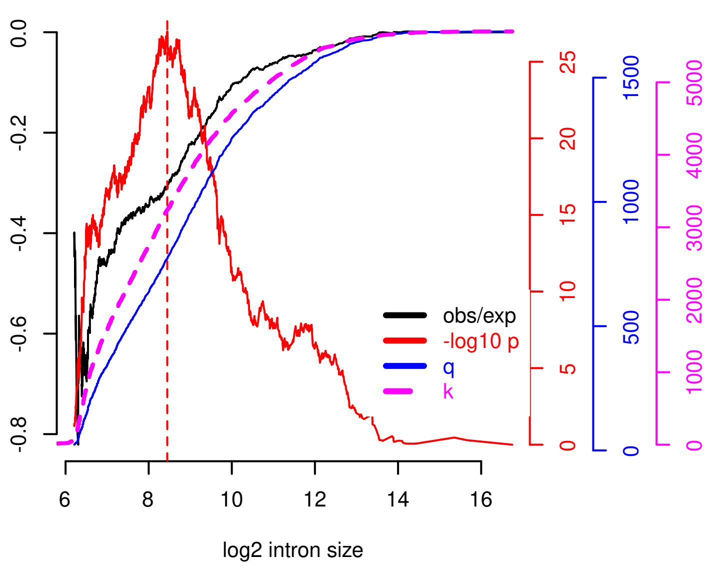

GO:0007154 cell communication

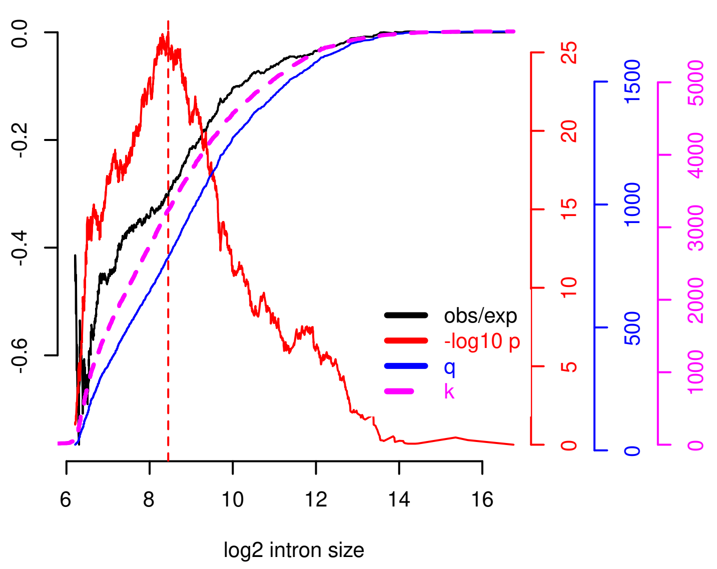

GO:0009653 anatomical structure morphogenesis

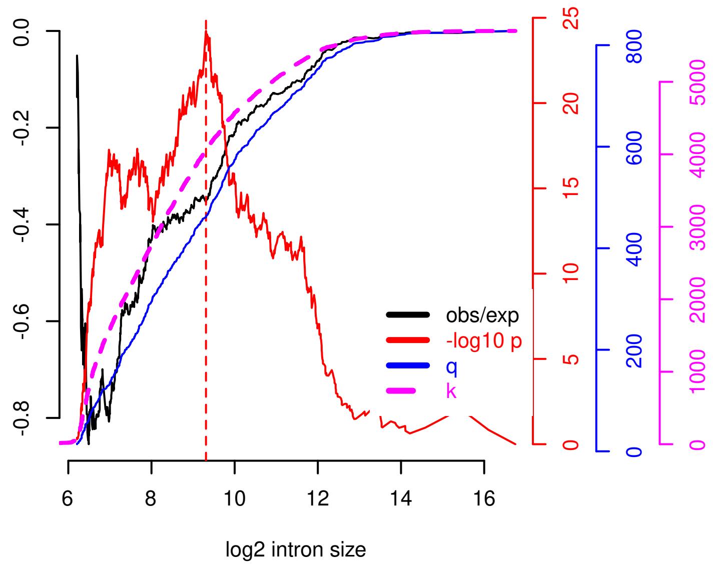

GO:0032502 developmental process

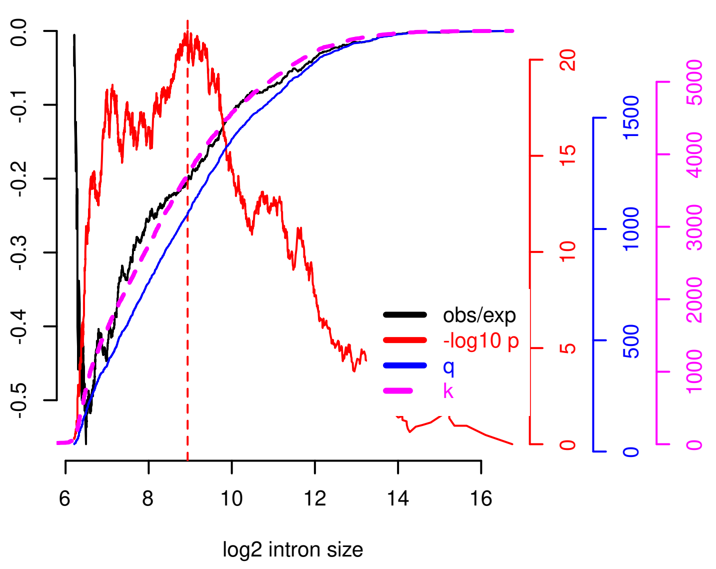

GO:0007165 signal transduction

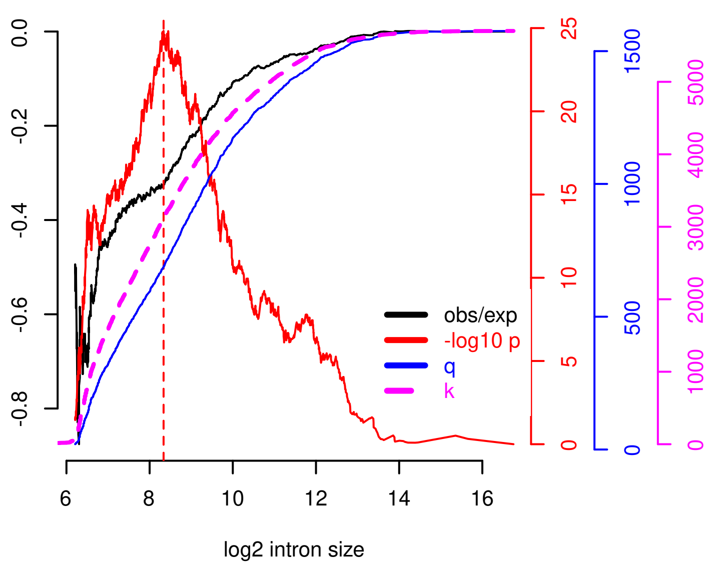

GO:0048513 animal organ development

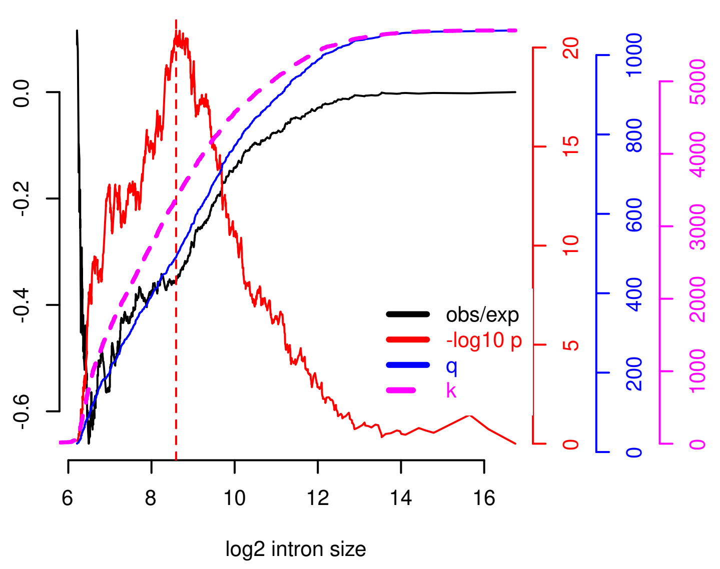

GO:0007155 cell adhesion

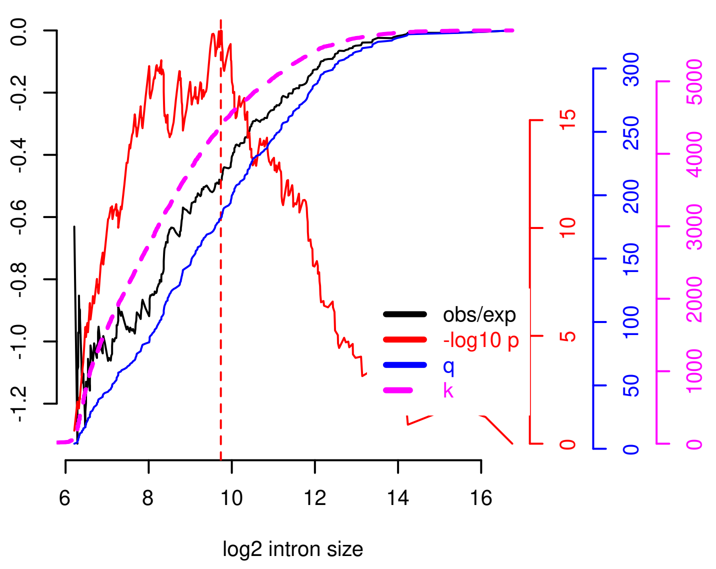

GO:0022610 biological adhesion

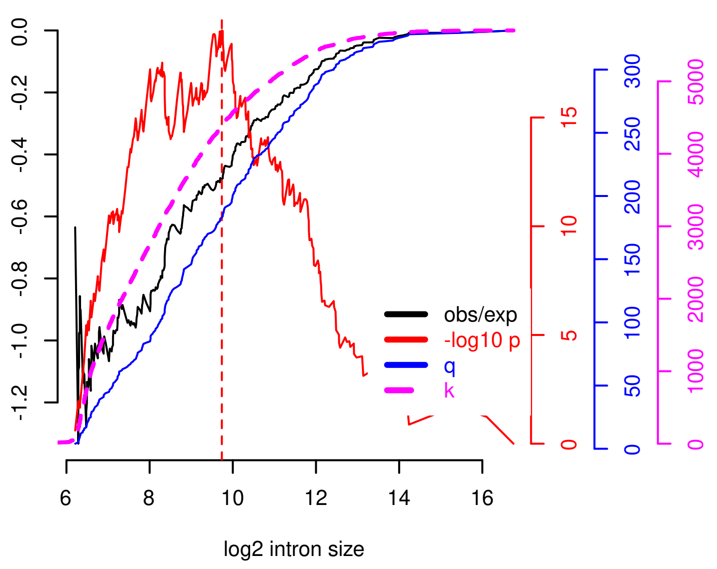

GO:0048869 cellular developmental process

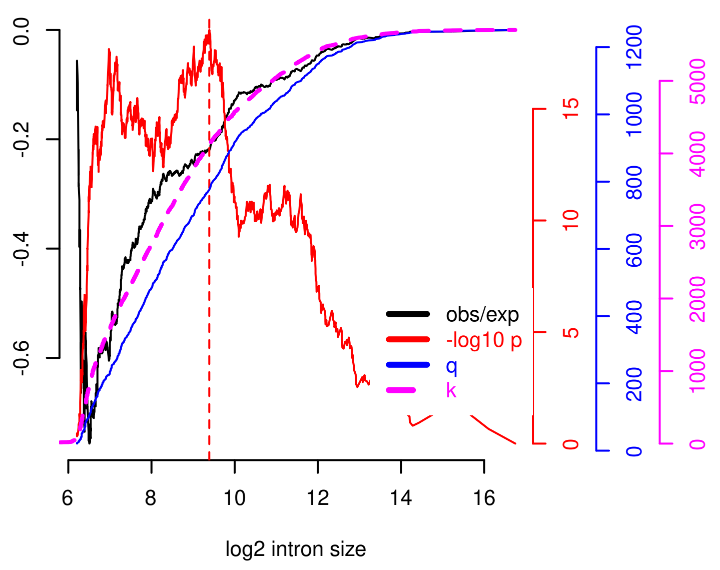

GO:0030154 cell differentiation

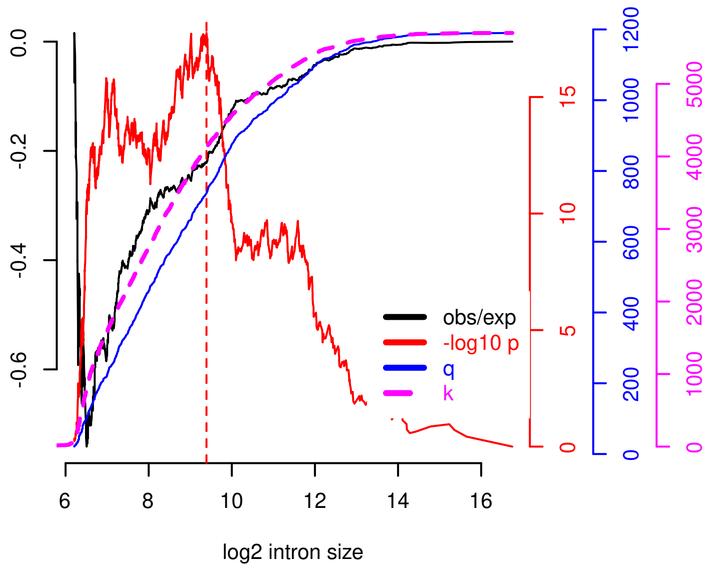

GO:0050794 regulation of cellular process

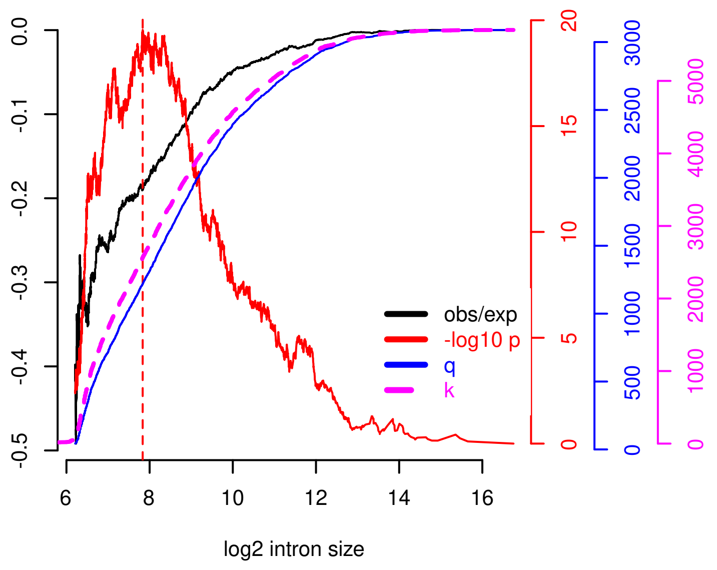

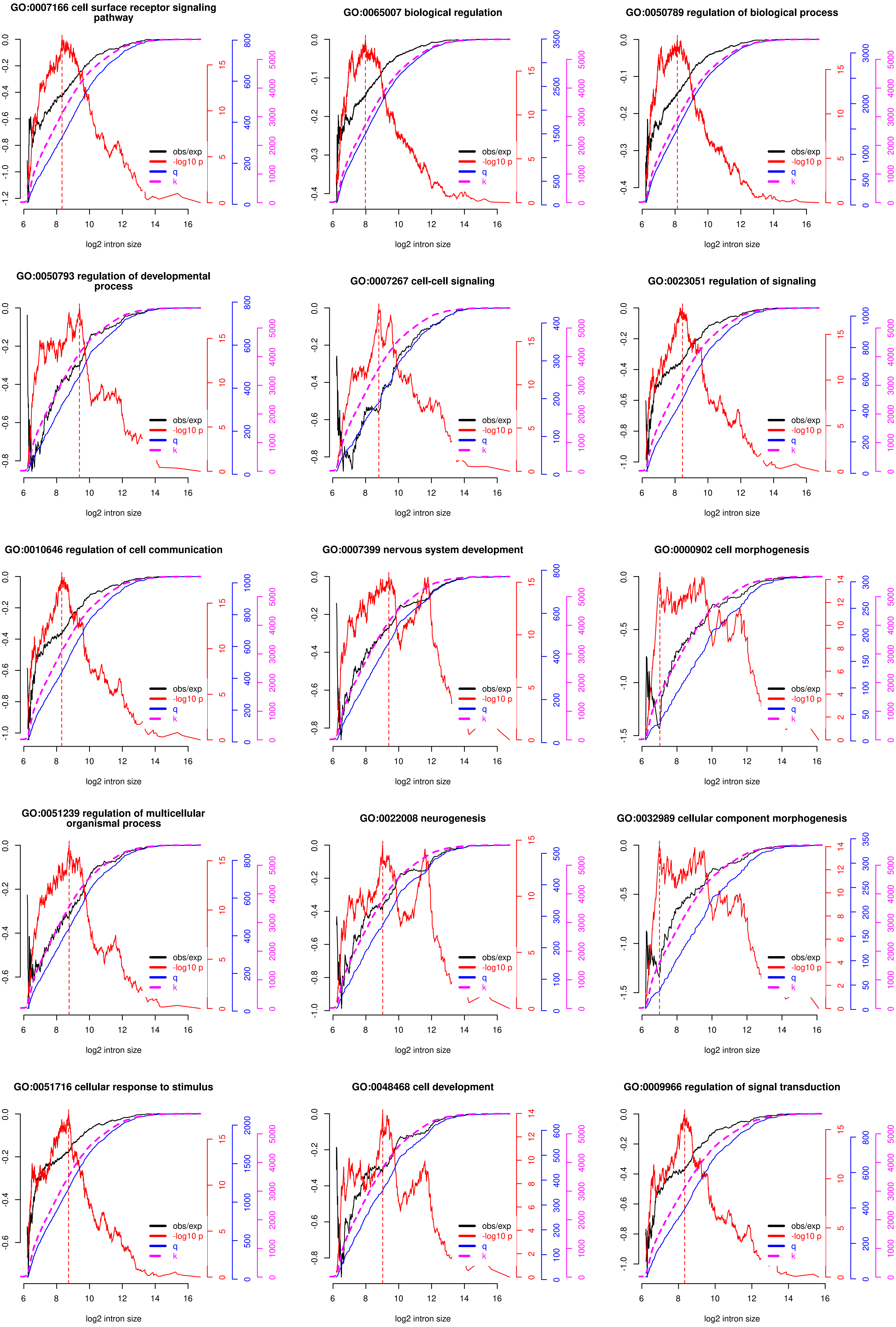
GO:0007166 cell surface receptor signaling pathway
obs/exp
-log10 p
q
k
log2 intron size
GO:0065007 biological regulation
obs/exp
-log10 p
q
k
log2 intron size
GO:0050789 regulation of biological process
obs/exp
-log10 p
q
k
log2 intron size
GO:0050793 regulation of developmental process
obs/exp
-log10 p
q
k
log2 intron size
GO:0007267 cell-cell signaling
obs/exp
-log10 p
q
k
log2 intron size
GO:0023051 regulation of signaling
obs/exp
-log10 p
q
k
log2 intron size
GO:0010646 regulation of cell communication
obs/exp
-log10 p
q
k
log2 intron size
GO:0007399 nervous system development
obs/exp
-log10 p
q
k
log2 intron size
GO:0000902 cell morphogenesis
obs/exp
-log10 p
q
k
log2 intron size
GO:0051239 regulation of multicellular organismal process
obs/exp
-log10 p
q
k
log2 intron size
GO:0022008 neurogenesis
obs/exp
-log10 p
q
k
log2 intron size
GO:0032989 cellular component morphogenesis
obs/exp
-log10 p
q
k
log2 intron size
GO:0051716 cellular response to stimulus
obs/exp
-log10 p
q
k
log2 intron size
GO:0048468 cell development
obs/exp
-log10 p
q
k
log2 intron size
GO:0009966 regulation of signal transduction
obs/exp
-log10 p
q
k
log2 intron size

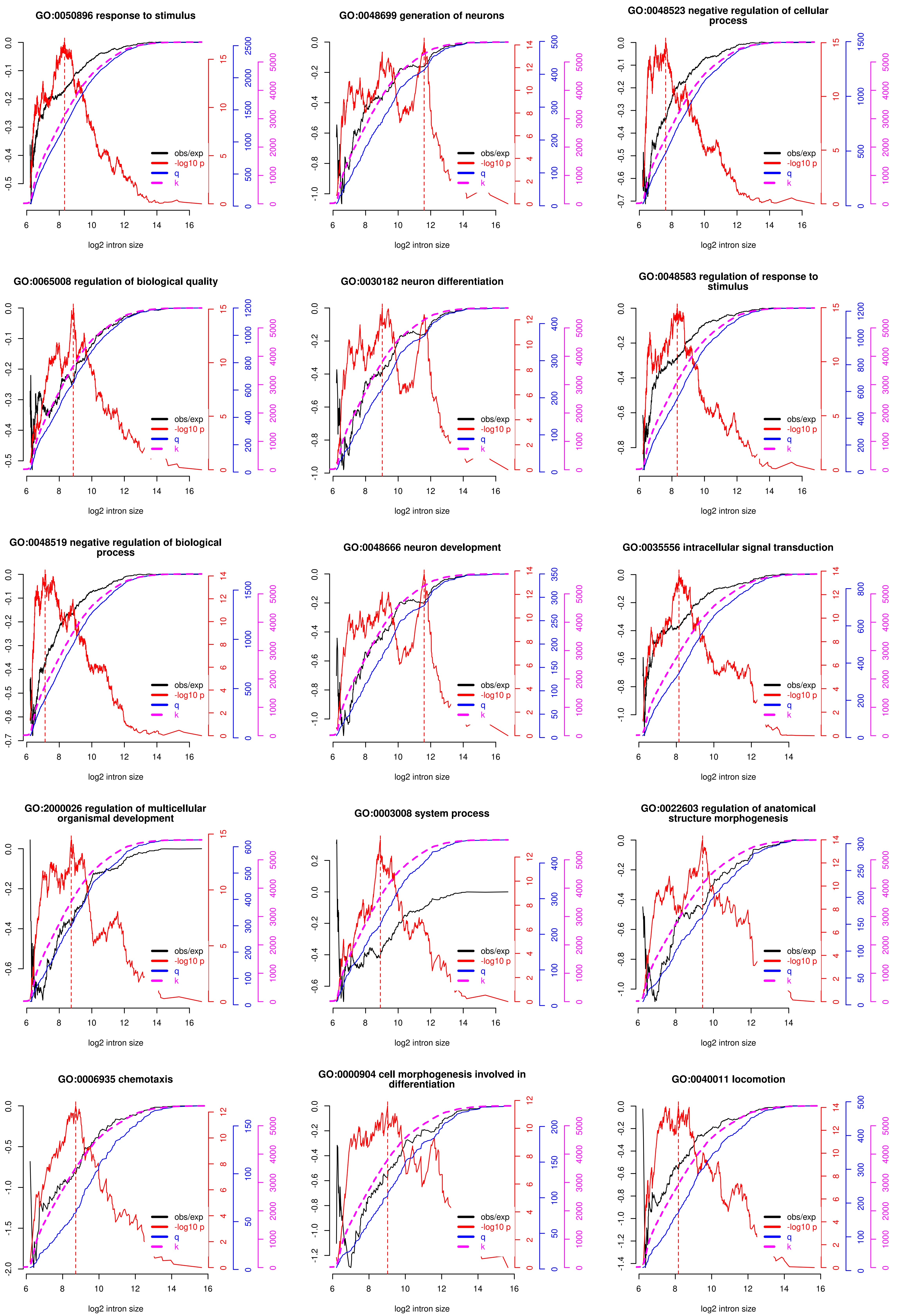
GO:0050896 response to stimulus
GO:0048699 generation of neurons
GO:0048523 negative regulation of cellular process
GO:0065008 regulation of biological quality
GO:0030182 neuron differentiation
GO:0048583 regulation of response to stimulus
GO:0048519 negative regulation of biological process
GO:0048666 neuron development
GO:0035556 intracellular signal transduction
GO:2000026 regulation of multicellular organismal development
GO:0003008 system process
GO:0022603 regulation of anatomical structure morphogenesis
GO:0006935 chemotaxis
GO:0000904 cell morphogenesis involved in differentiation
GO:0040011 locomotion
obs/exp
-log10 p
q
k
log2 intron size

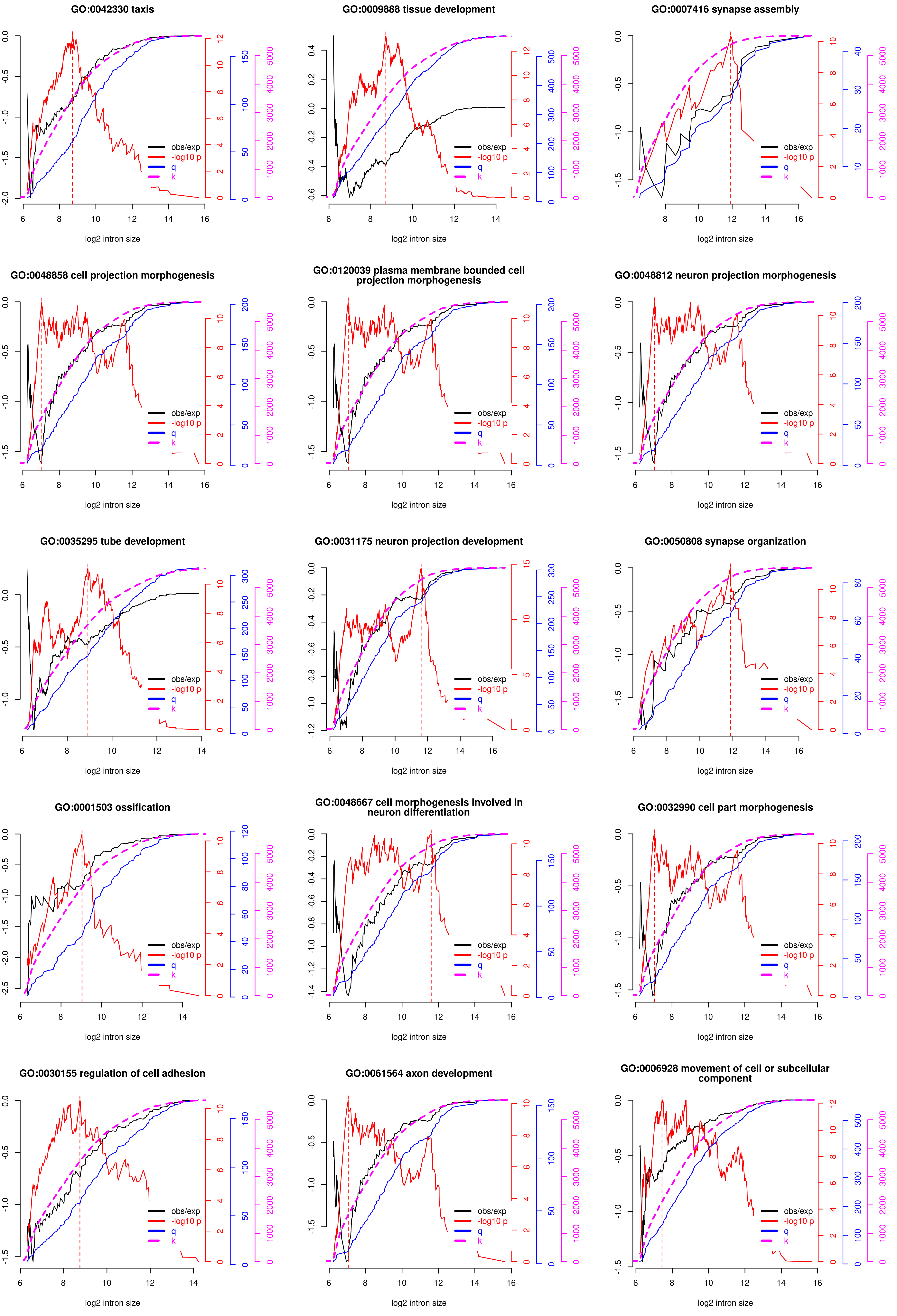
GO:0042330 taxis
GO:0009888 tissue development
GO:0007416 synapse assembly
GO:0048858 cell projection morphogenesis
GO:0120039 plasma membrane bounded cell projection morphogenesis
GO:0048812 neuron projection morphogenesis
GO:0035295 tube development
GO:0031175 neuron projection development
GO:0050808 synapse organization
GO:0001503 ossification
GO:0048667 cell morphogenesis involved in neuron differentiation
GO:0032990 cell part morphogenesis
GO:0030155 regulation of cell adhesion
GO:0061564 axon development
GO:0006928 movement of cell or subcellular component
log2 intron size
obs/exp
-log10 p
q
k

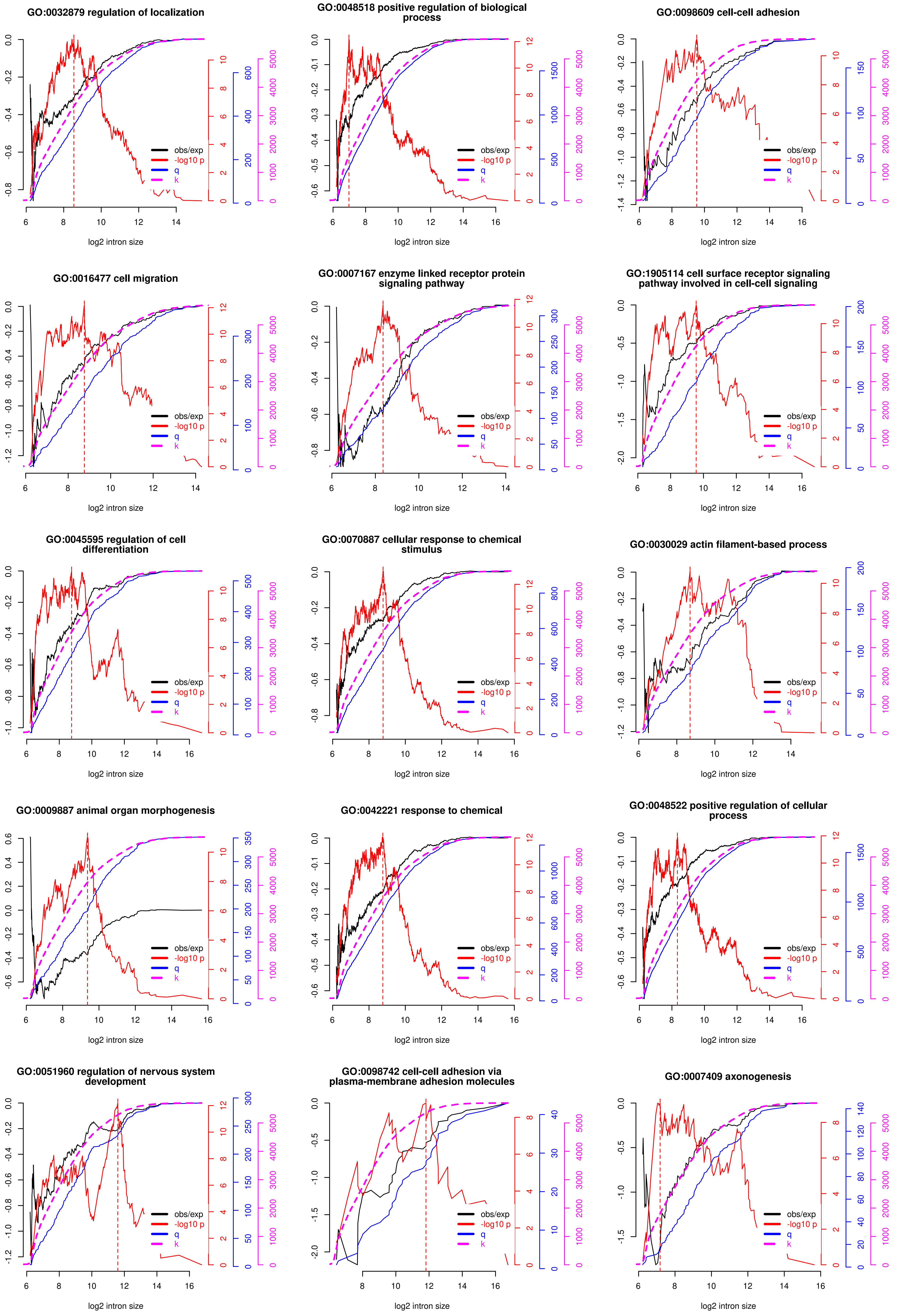
GO:0032879 regulation of localization
GO:0048518 positive regulation of biological process
GO:0098609 cell-cell adhesion
GO:0016477 cell migration
GO:0007167 enzyme linked receptor protein signaling pathway
GO:1905114 cell surface receptor signaling pathway involved in cell-cell signaling
GO:0045595 regulation of cell differentiation
GO:0070887 cellular response to chemical stimulus
GO:0030029 actin filament-based process
GO:0009887 animal organ morphogenesis
GO:0042221 response to chemical
GO:0048522 positive regulation of cellular process
GO:0051960 regulation of nervous system development
GO:0098742 cell-cell adhesion via plasma-membrane adhesion molecules
GO:0007409 axonogenesis
obs/exp
-log10 p
q
k
log2 intron size

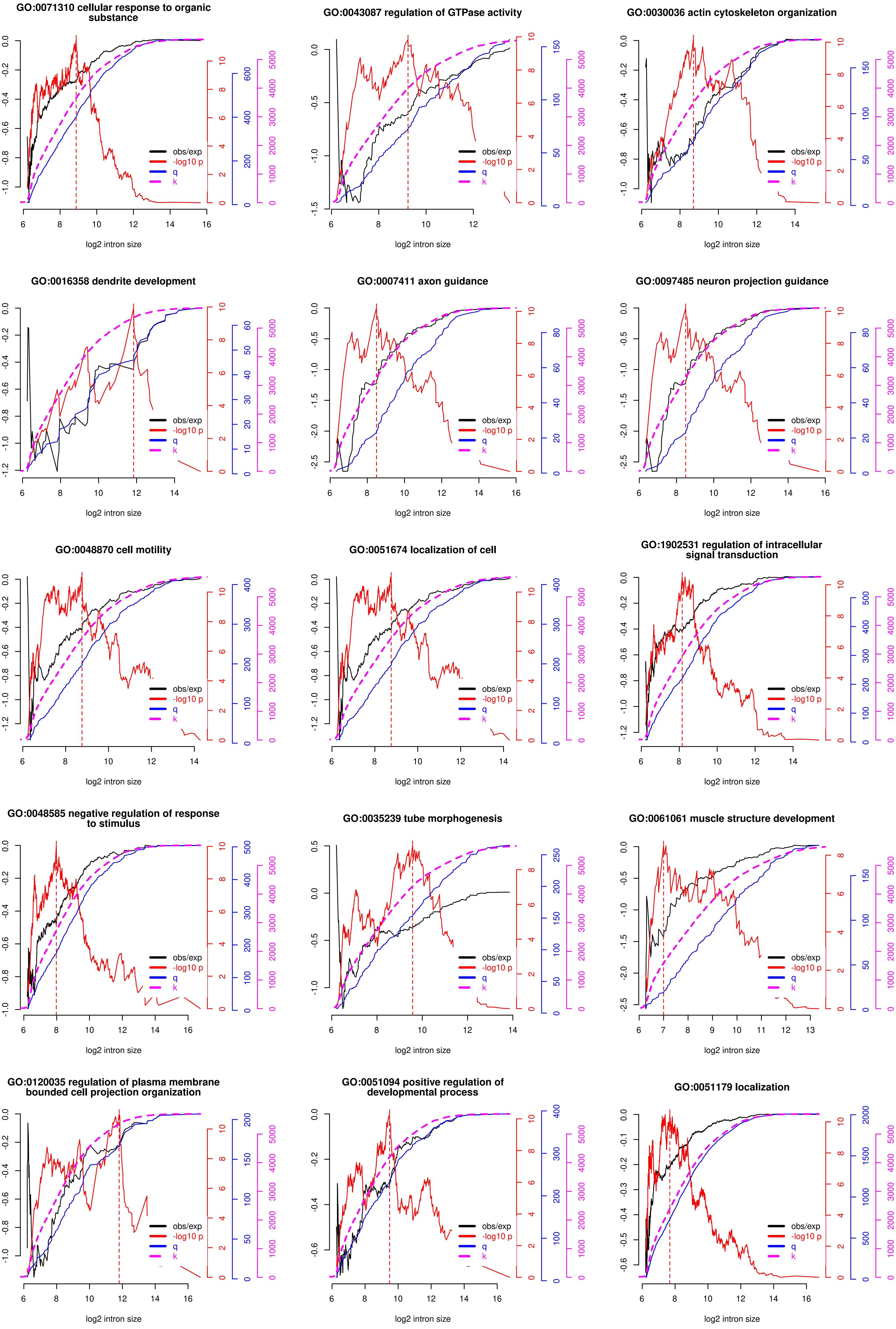
GO:0071310 cellular response to organic substance
GO:0043087 regulation of GTPase activity
GO:0030036 actin cytoskeleton organization
GO:0016358 dendrite development
GO:0007411 axon guidance
GO:0097485 neuron projection guidance
GO:0048870 cell motility
GO:0051674 localization of cell
GO:1902531 regulation of intracellular signal transduction
GO:0048585 negative regulation of response to stimulus
GO:0035239 tube morphogenesis
GO:0061061 muscle structure development
GO:0120035 regulation of plasma membrane bounded cell projection organization
GO:0051094 positive regulation of developmental process
GO:0051179 localization
obs/exp
-log10 p
q
k
log2 intron size

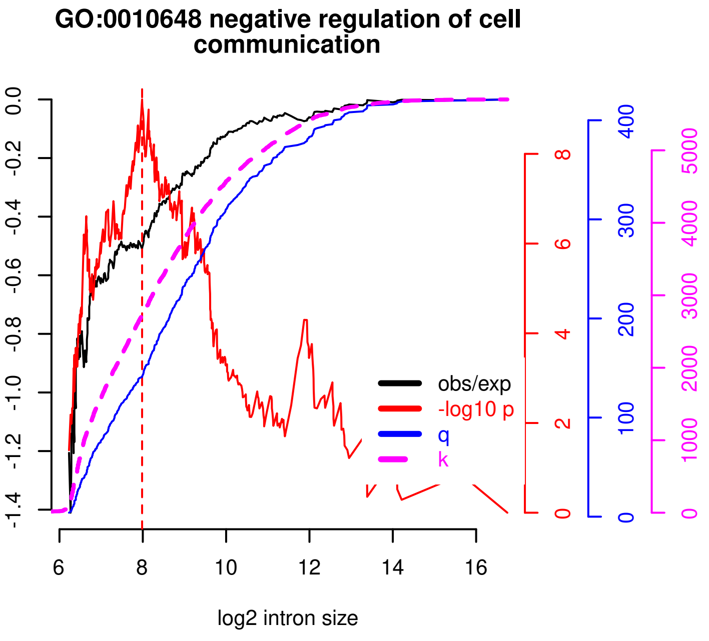
GO:0010648 negative regulation of cell communication
obs/exp
-log10 p
q
k
log2 intron size

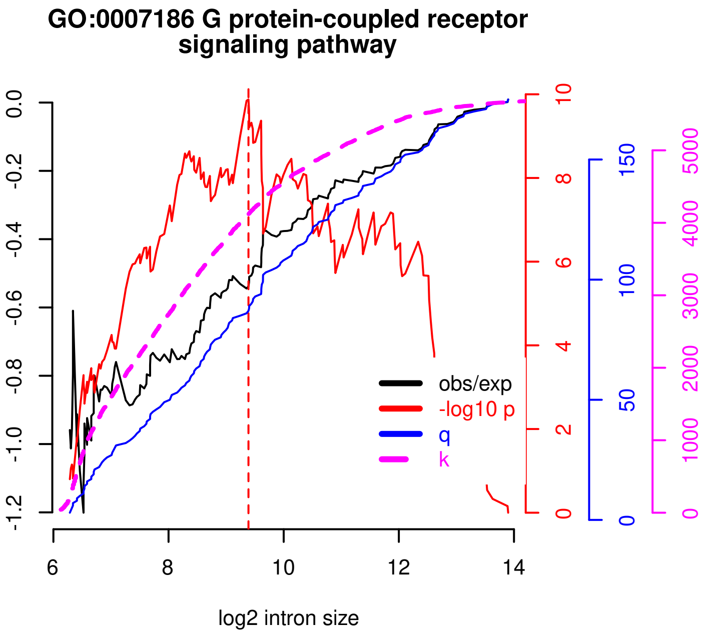
GO:0007186 G protein-coupled receptor signaling pathway
obs/exp
-log10 p
q
k
log2 intron size

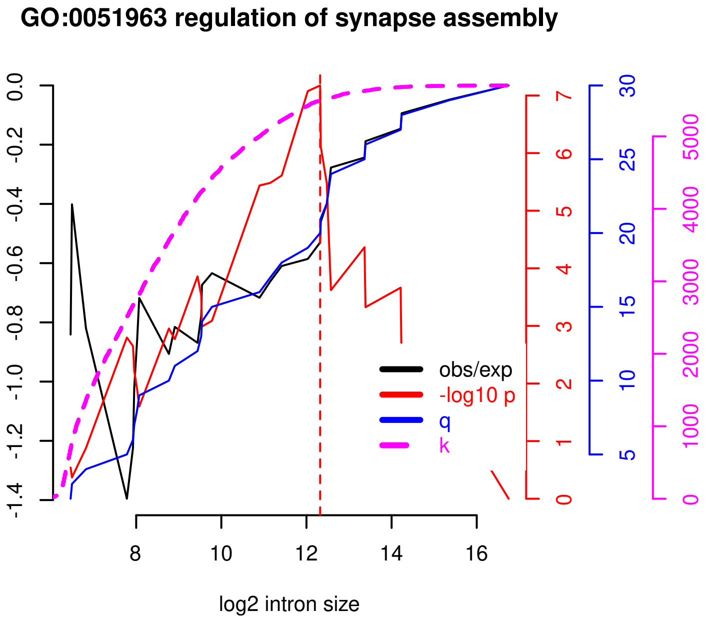
GO:0051963 regulation of synapse assembly
obs/exp
-log10 p
q
k
log2 intron size

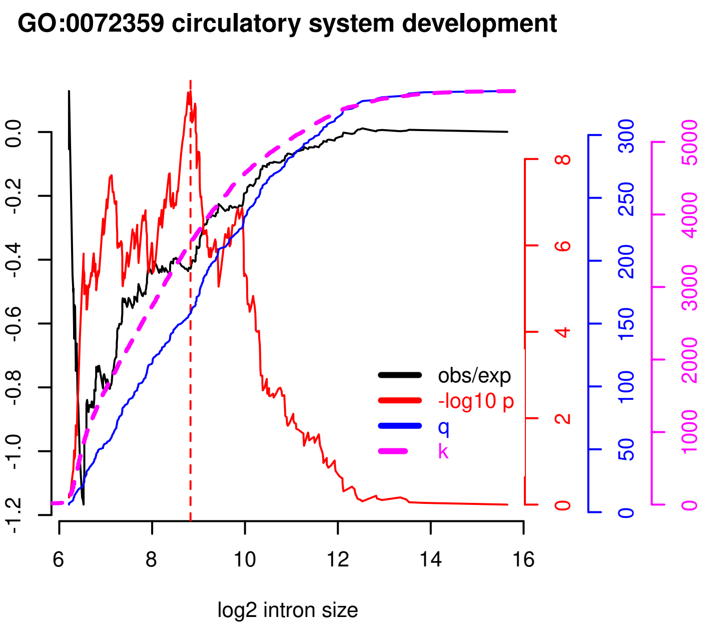
GO:0072359 circulatory system development
obs/exp
-log10 p
q
k
log2 intron size

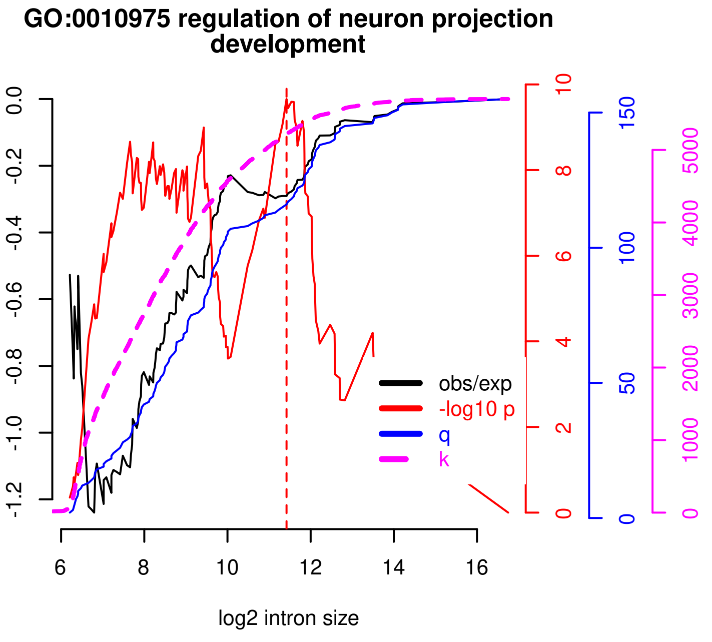
GO:0010975 regulation of neuron projection development
obs/exp
-log10 p
q
k
log2 intron size

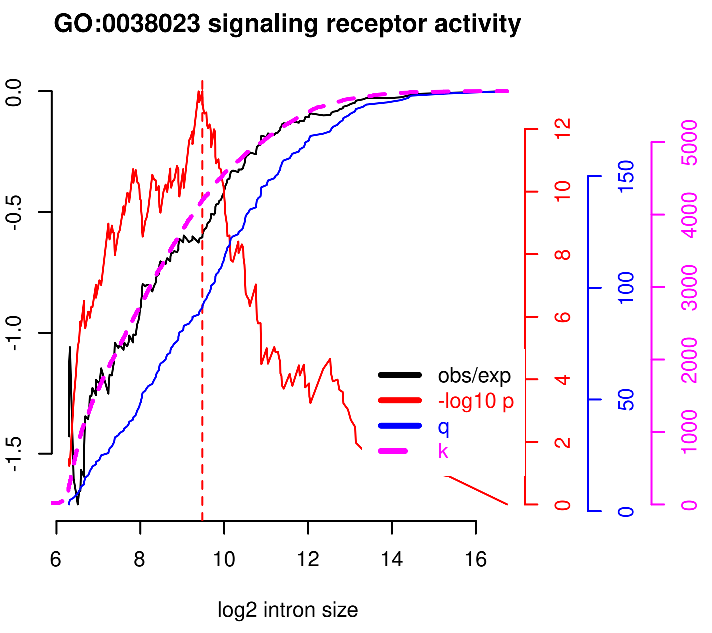
GO:0038023 signaling receptor activity
obs/exp
-log10 p
q
k
log2 intron size

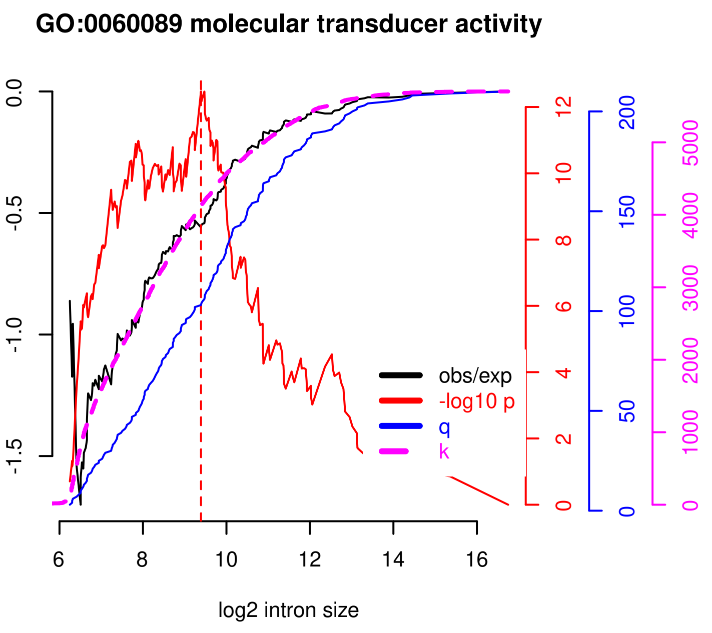
GO:0060089 molecular transducer activity
obs/exp
-log10 p
q
k
log2 intron size

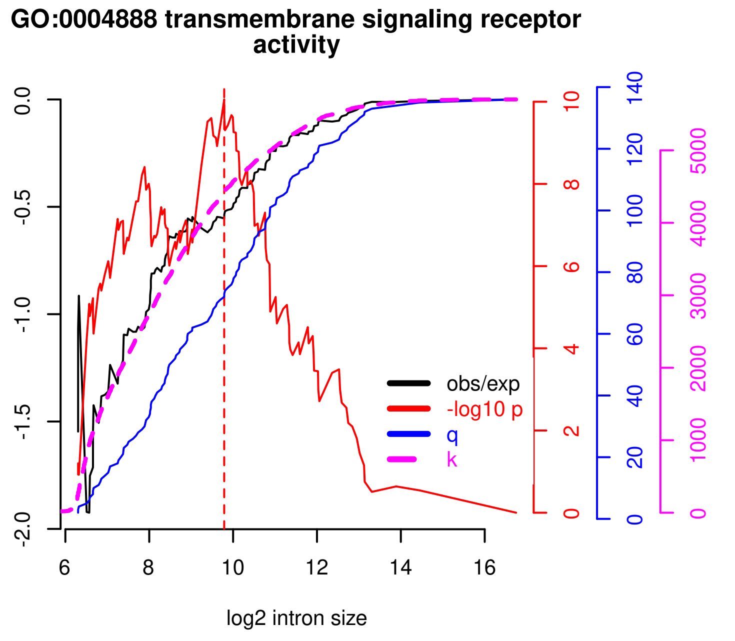
GO:0004888 transmembrane signaling receptor activity
obs/exp
-log10 p
q
k
log2 intron size

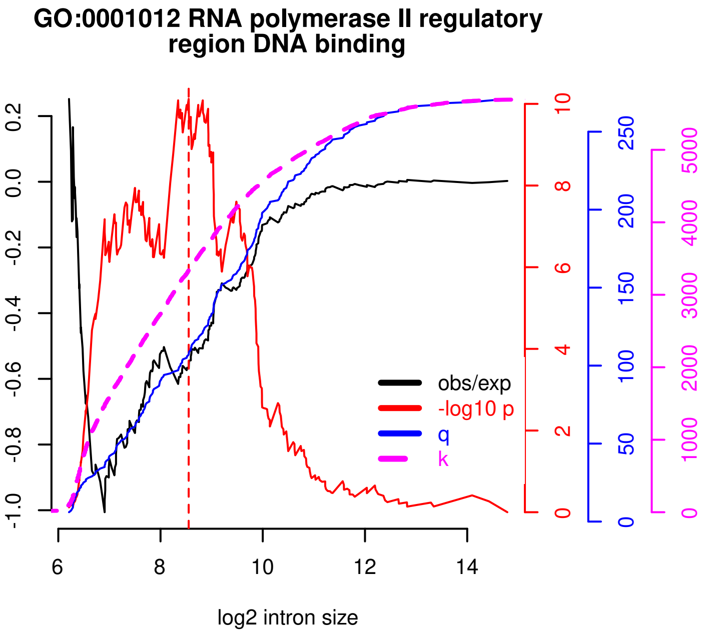
GO:0001012 RNA polymerase II regulatory region DNA binding
obs/exp
-log10 p
q
k
log2 intron size

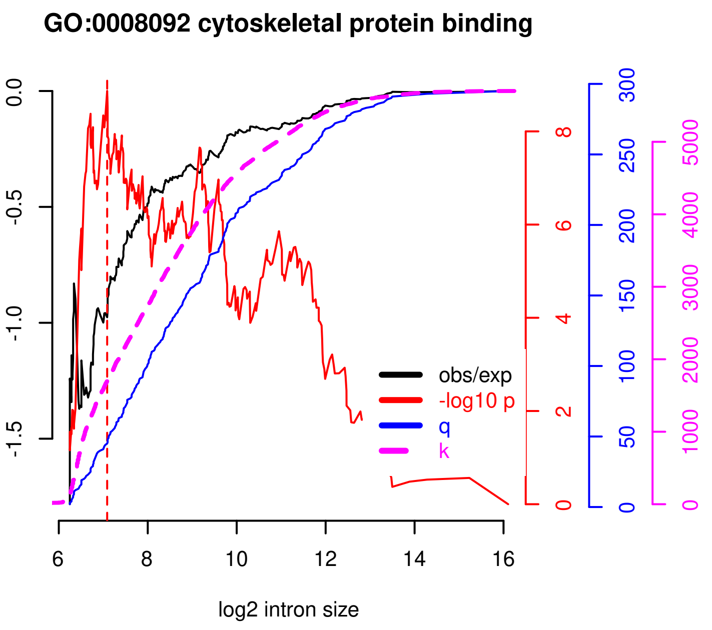
GO:0008092 cytoskeletal protein binding
obs/exp
-log10 p
q
k
log2 intron size

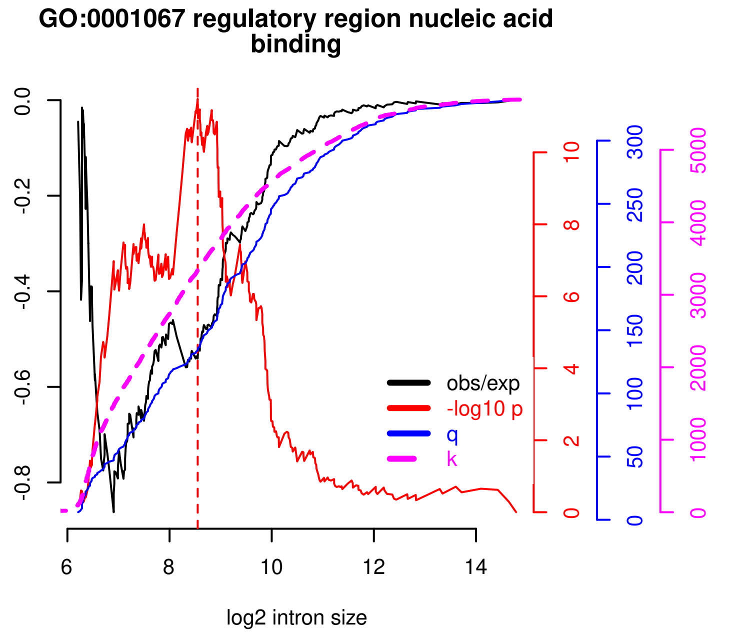
GO:0001067 regulatory region nucleic acid binding
obs/exp
-log10 p
q
k
log2 intron size

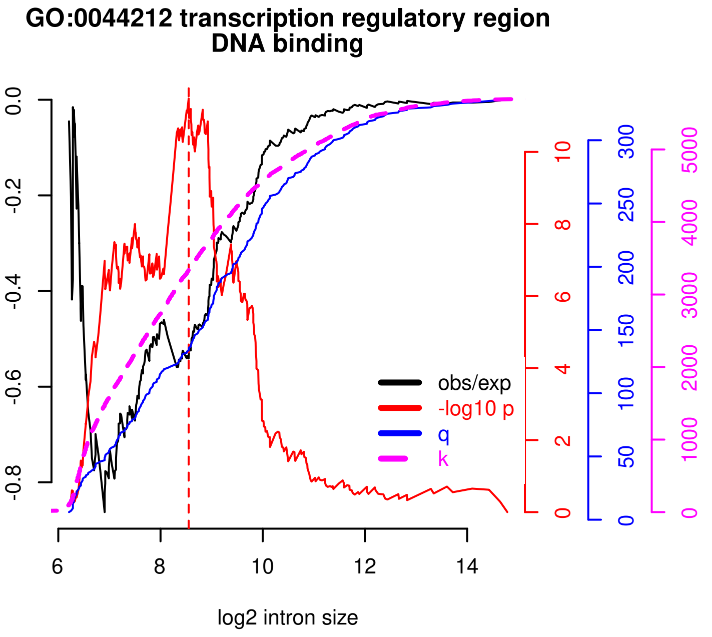
GO:0044212 transcription regulatory region DNA binding
obs/exp
-log10 p
q
k
log2 intron size

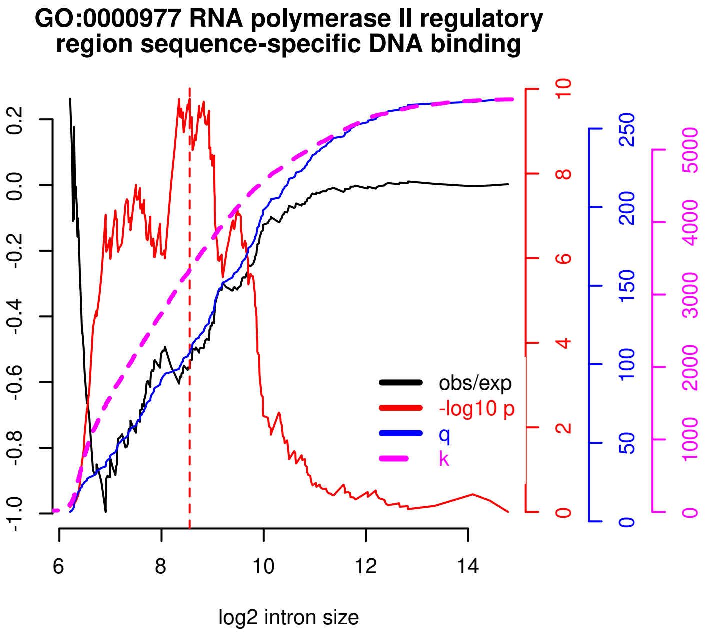
GO:0000977 RNA polymerase II regulatory region sequence-specific DNA binding
obs/exp
-log10 p
q
k
log2 intron size

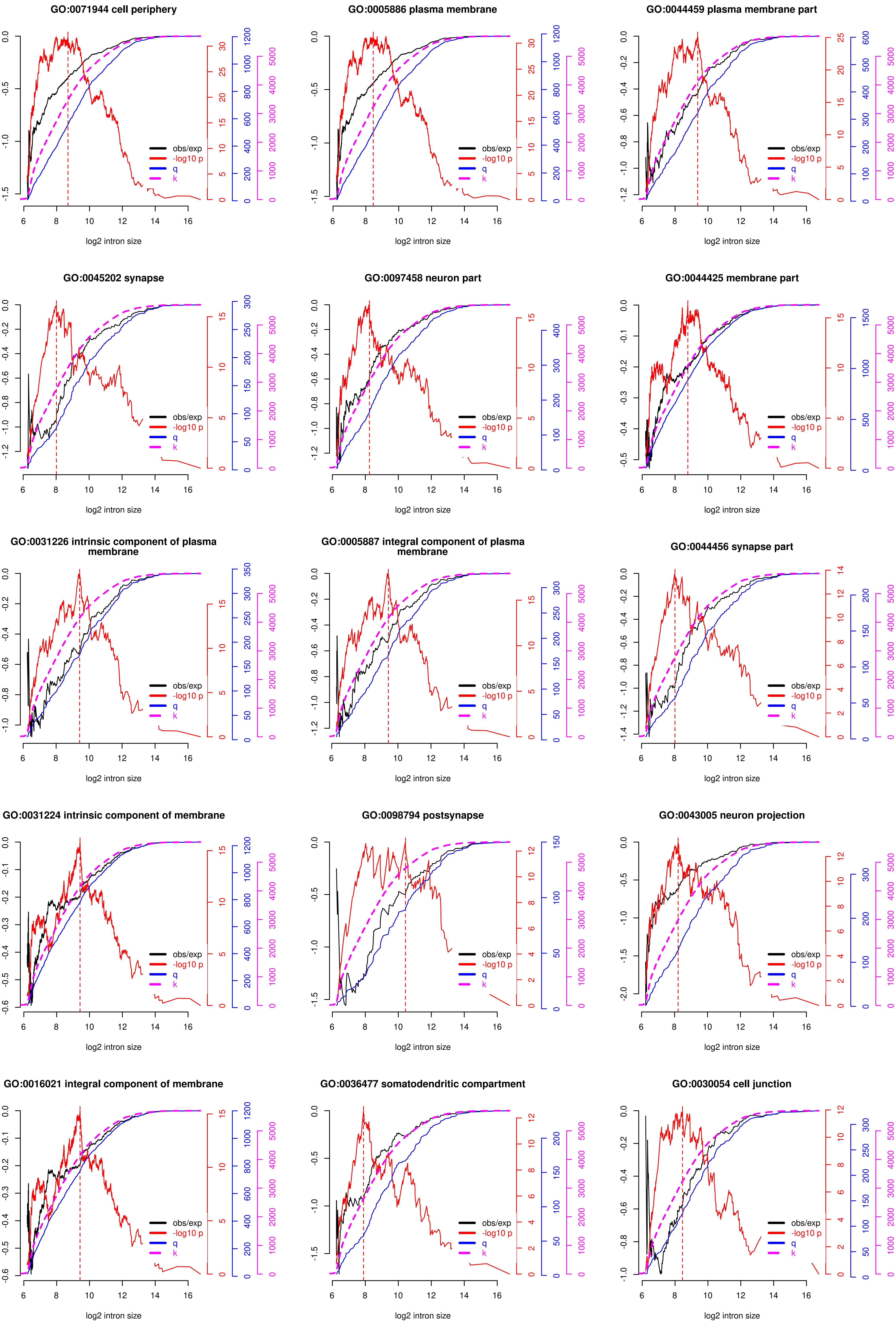
GO:0071944 cell periphery
GO:0005886 plasma membrane
GO:0044459 plasma membrane part
GO:0045202 synapse
GO:0097458 neuron part
GO:0044425 membrane part
GO:0031226 intrinsic component of plasma membrane
GO:0005887 integral component of plasma membrane
GO:0044456 synapse part
GO:0031224 intrinsic component of membrane
GO:0098794 postsynapse
GO:0043005 neuron projection
GO:0016021 integral component of membrane
GO:0036477 somatodendritic compartment
GO:0030054 cell junction
obs/exp
-log10 p
q
k
log2 intron size

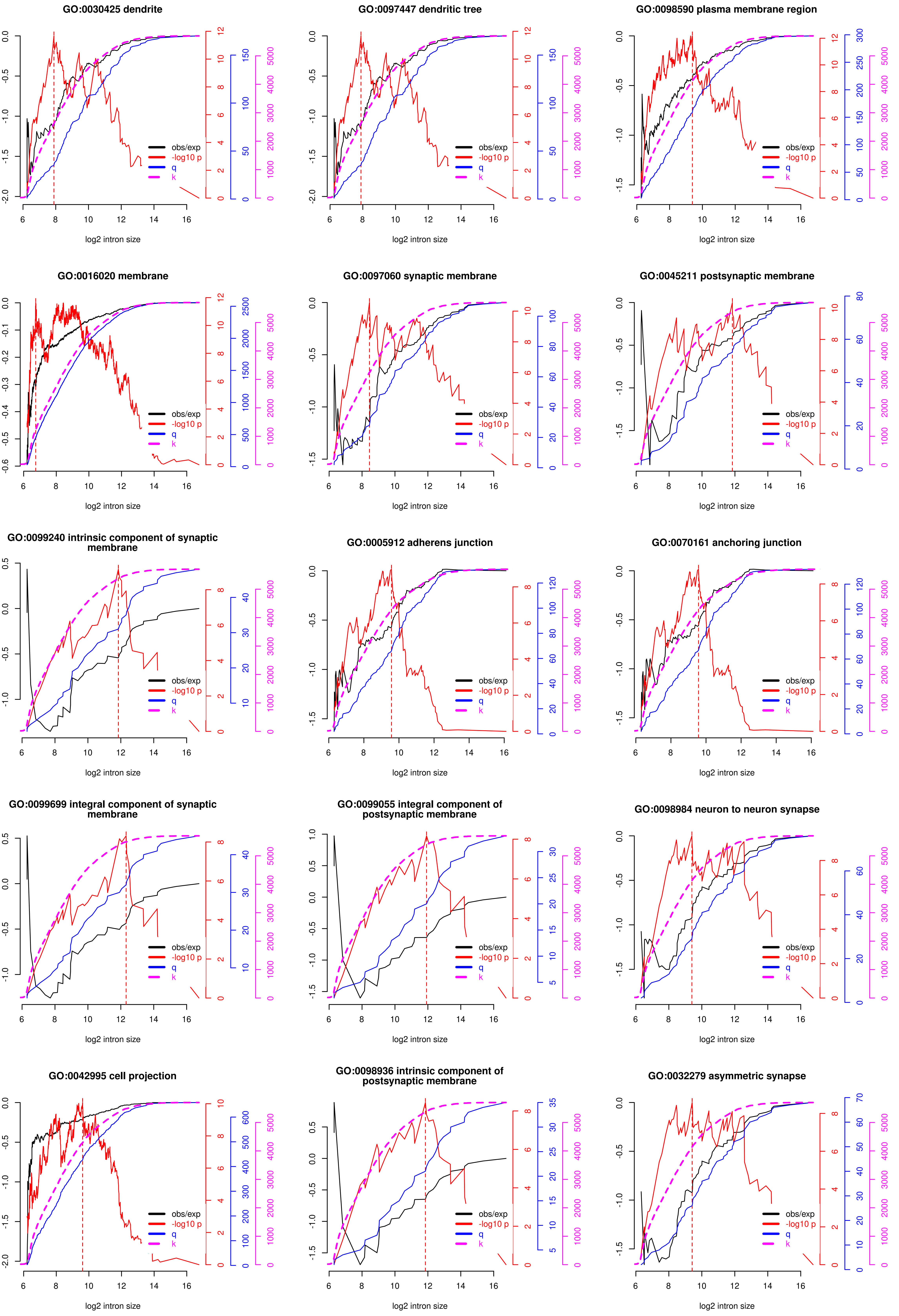

GO:0030425 dendrite
GO:0097447 dendritic tree
GO:0098590 plasma membrane region
GO:0016020 membrane
GO:0097060 synaptic membrane
GO:0045211 postsynaptic membrane
GO:0099240 intrinsic component of synaptic membrane
GO:0005912 adherens junction
GO:0070161 anchoring junction
GO:0099699 integral component of synaptic membrane
GO:0099055 integral component of postsynaptic membrane
GO:0098984 neuron to neuron synapse
GO:0042995 cell projection
GO:0098936 intrinsic component of postsynaptic membrane
GO:0032279 asymmetric synapse
obs/exp
-log10 p
q
k
log2 intron size

GO:0099572 postsynaptic specialization

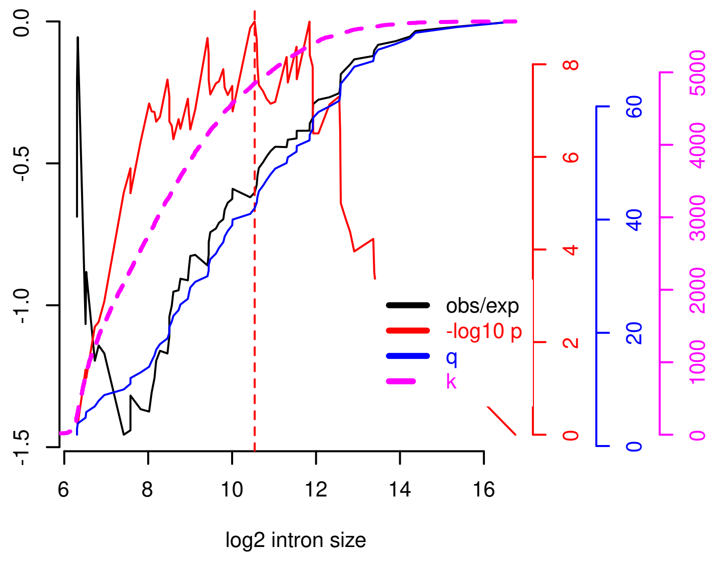

Supplement: Supplementary file 3 — Additional file 3 Gene ontology depletion statistics. Individual panels show statistics associated with an under-representation of members of gene ontology groups in nested sets of human genes ordered by the minimum size of their largest orthologous teleost intron. The statistics are shown plotted against the size (log2 transformed) of the largest teleost intron of the nested set (x-axis). The statistics shown are: log2(observed/expected) (black), −log10 hypergeometric probabilities associated with the observations (red), q the number of members of the gene ontology set (blue) and k, the total number of genes in the nested set (purple). The dashed vertical lines show the position of the minimal hypergeometric p-values. The minimal p-values are associated with an inflexion in the observed / expected ratios and occur close to the antimode of the typical teleost intron length distribution. Pages 1-7: Biological Process (BP), 8: Molecular Function (MF), 9-11 Cellular Compartment (CC). [file 12864_2022_8760_MOESM3_ESM.pdf]
