# Supplementary material for: Intron size minimisation in teleosts
Source: BMC Genomics. 2022 Sep 1;23:628. doi: 10.1186/s12864-022-08760-w (PMC9438311; doi:10.1186/s12864-022-08760-w)
Supplement: Supplementary file 4 — Additional file 4 Local alignments of D. rerio intron sequences to vertebrate orthologues for a set of introns that are long (>1024 bp) across the teleosts. Alignments were performed recursively to identify all non-overlapping alignments to the D. rerio sequence. [file 12864_2022_8760_MOESM4_ESM.pdf]

ENSDARG00000105243 : ENSDART00000159101 intron no: 4 length: 3192

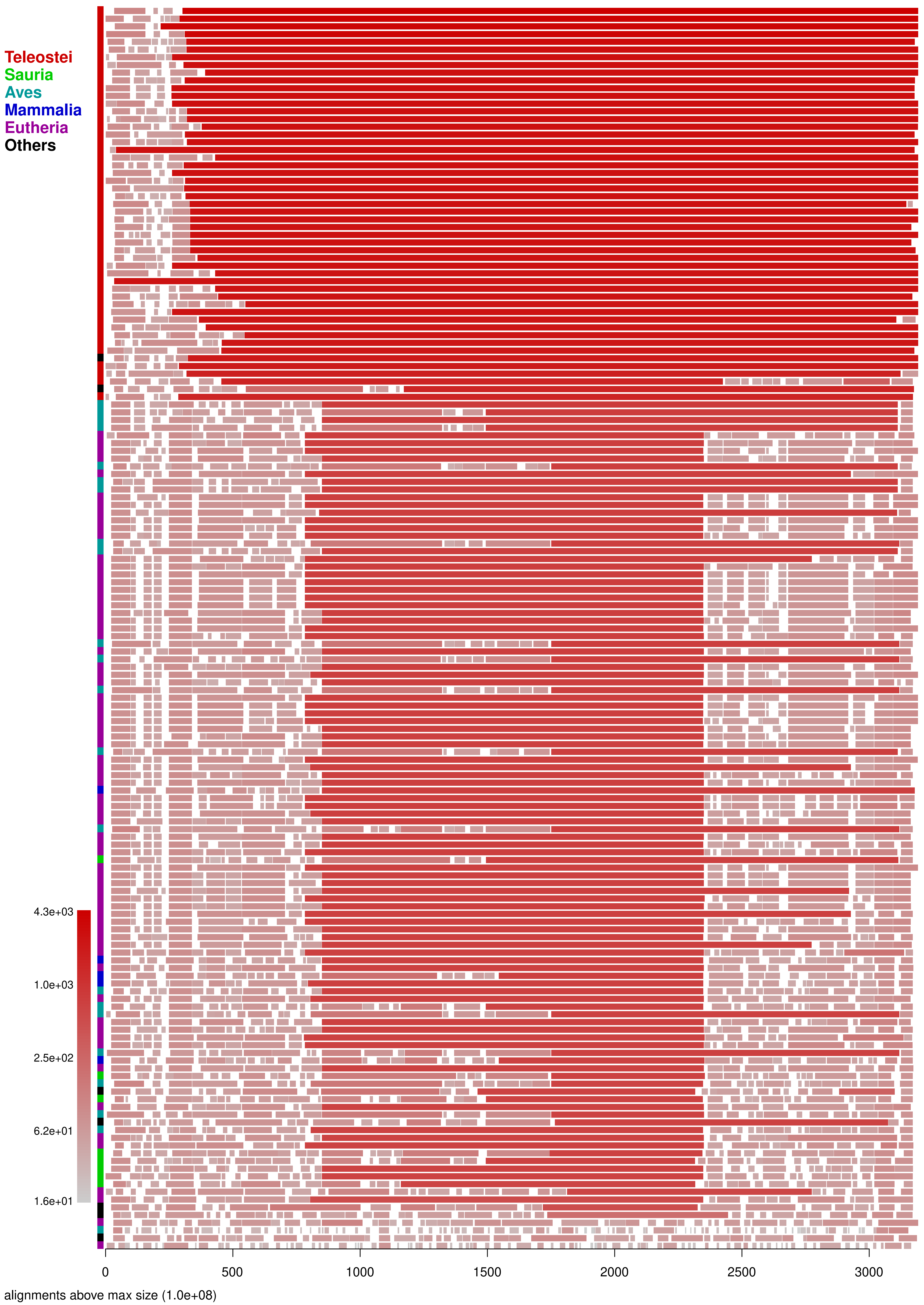

0 alignments above max size (1.0e+08)

ENSDARG00000077052 : ENSDART00000155112 intron no: 1 length: 1372

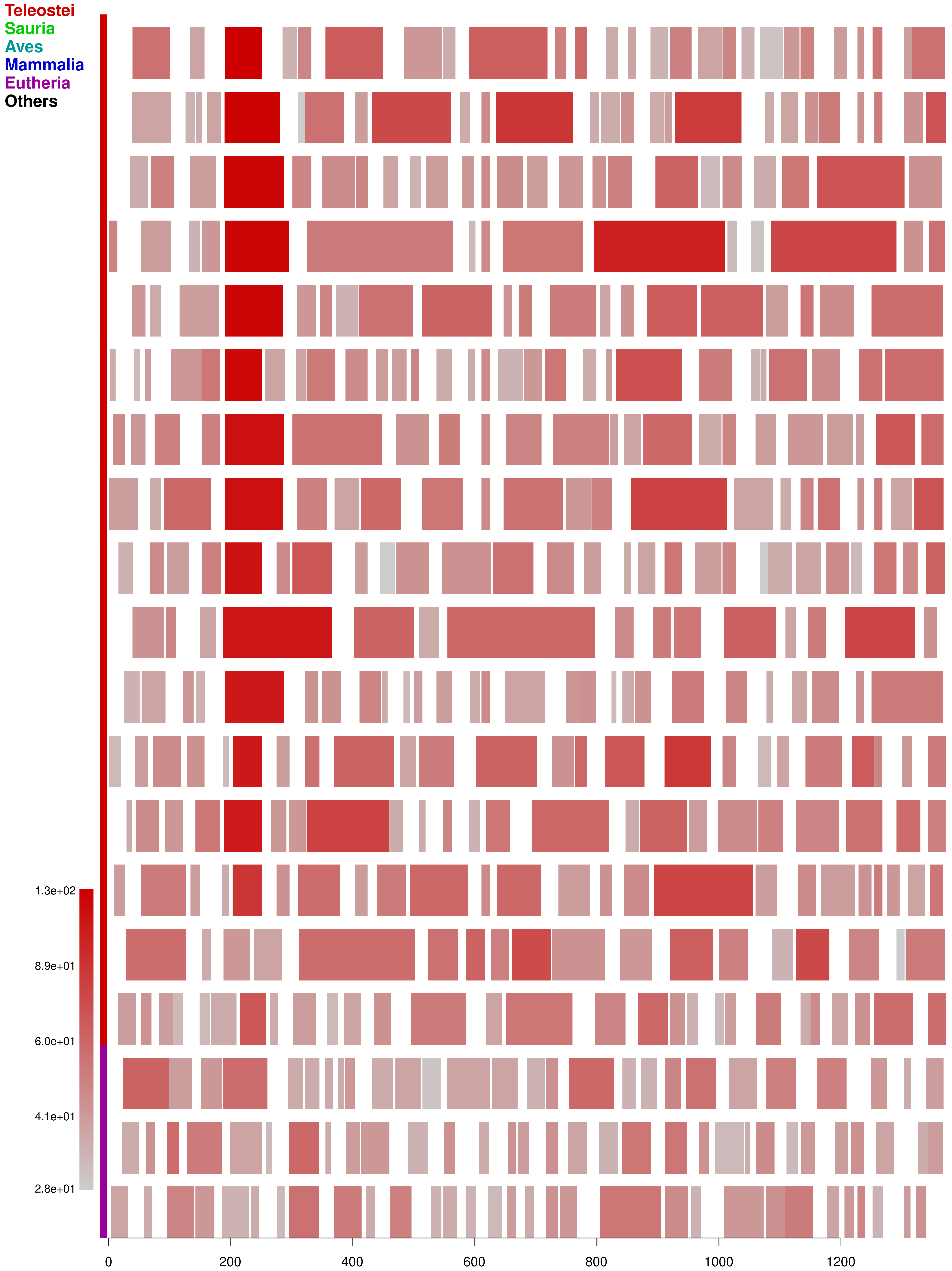

0 alignments above max size (1.0e+08)

ENSDARG00000055124 : ENSDART00000077435 intron no: 3 length: 2151

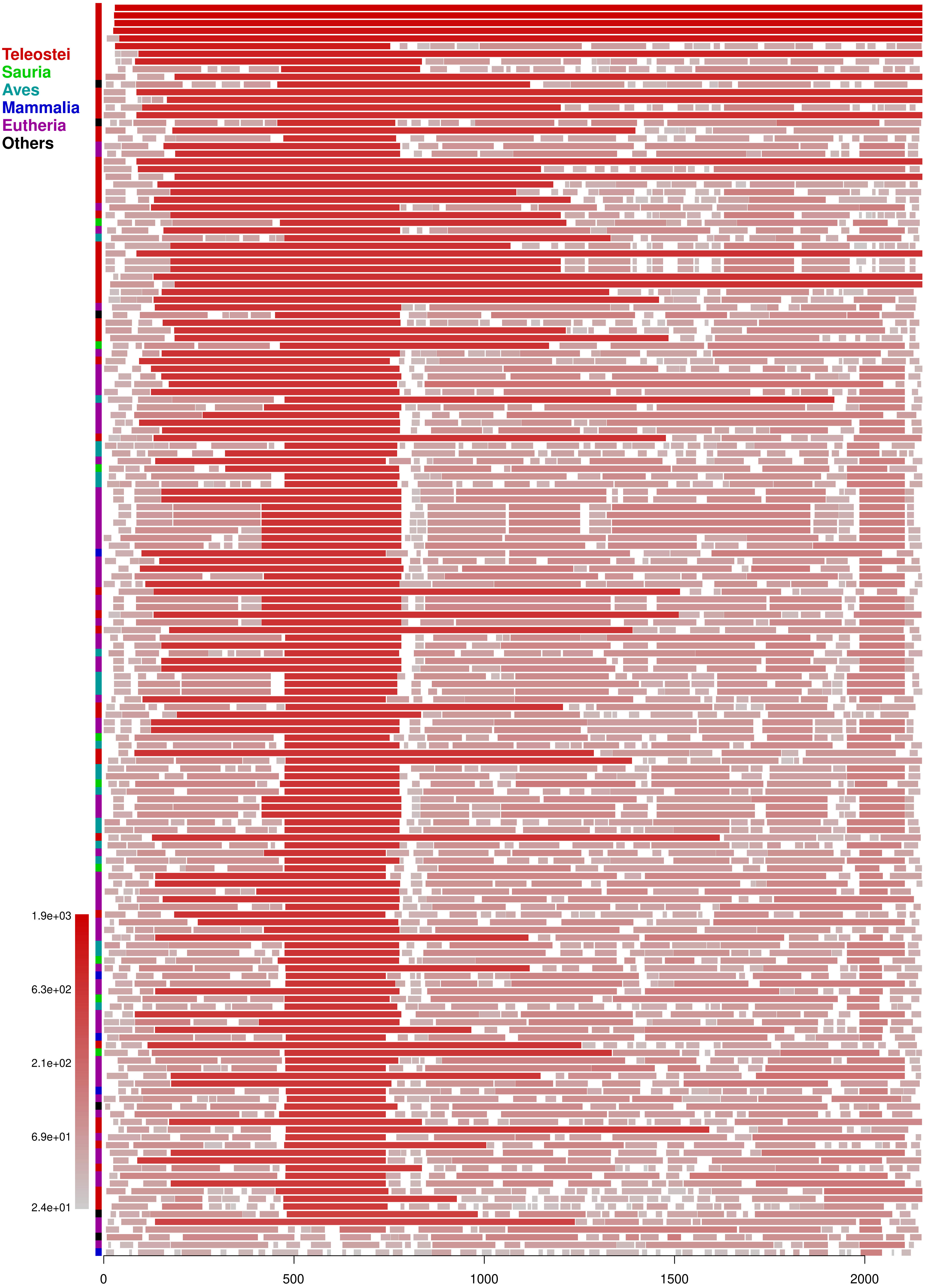

0 alignments above max size (1.0e+08)

ENSDARG00000055026 : ENSDART00000077304 intron no: 2 length: 7581

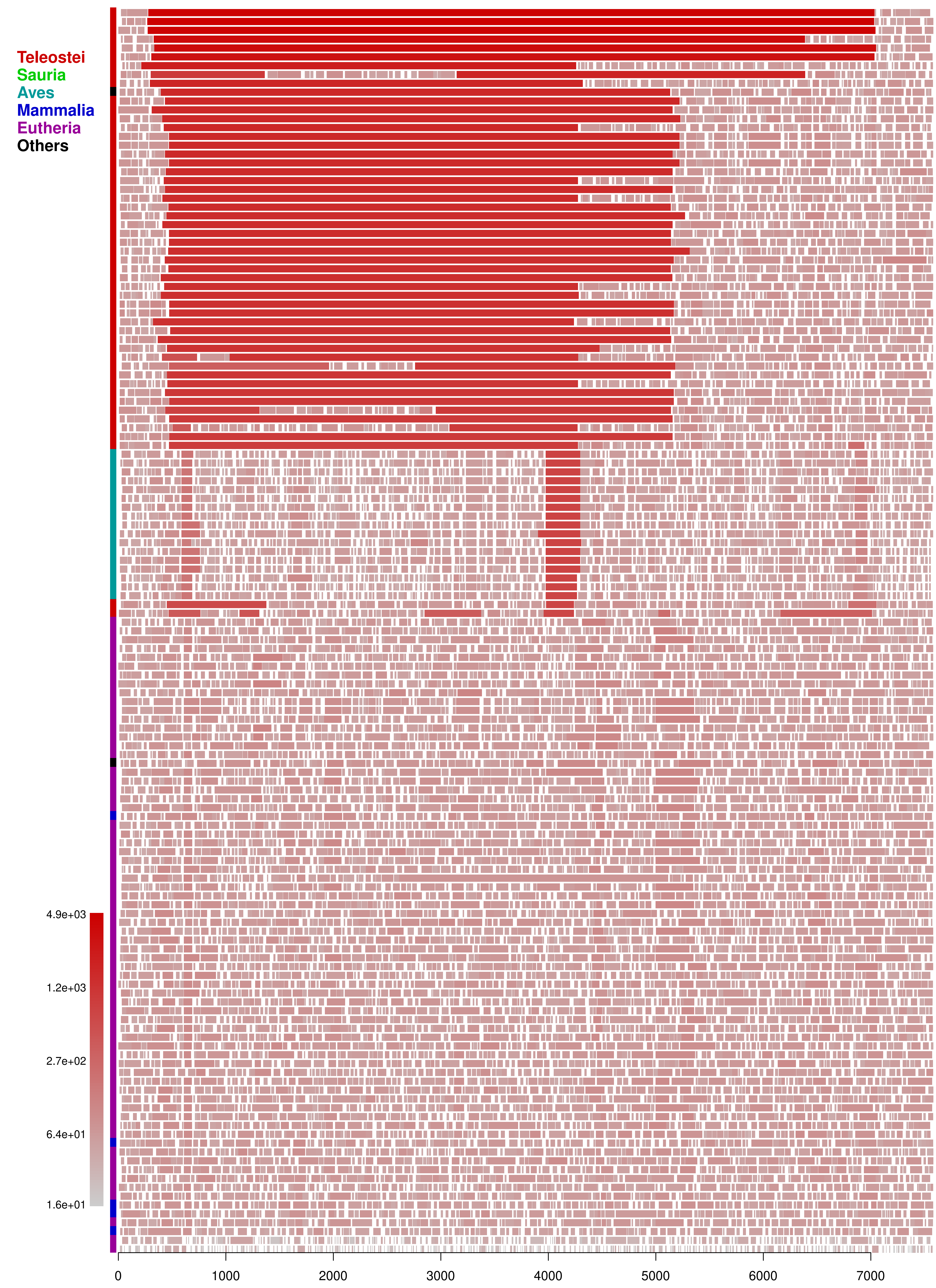

11 alignments above max size (1.0e+08)

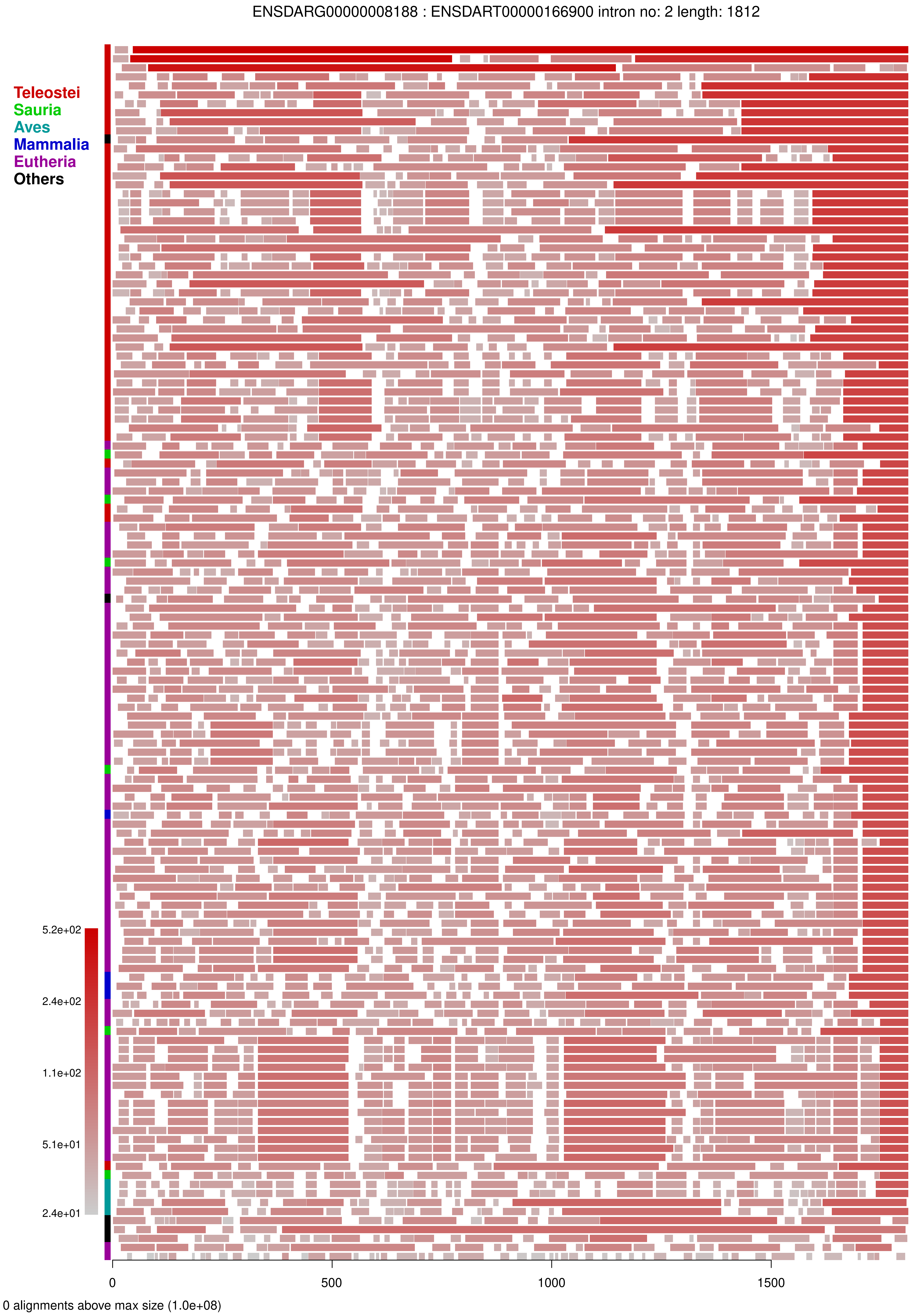
ENSDARG00000008188 : ENSDART00000166900 intron no: 2 length: 1812
Teleostei
Sauria
Aves
Mammalia
Eutheria
Others
5.2e+02
2.4e+02
1.1e+02
5.1e+01
2.4e+01
0
500
1000
1500
0 alignments above max size (1.0e+08)

ENSDARG00000089156 : ENSDART00000127062 intron no: 1 length: 3031

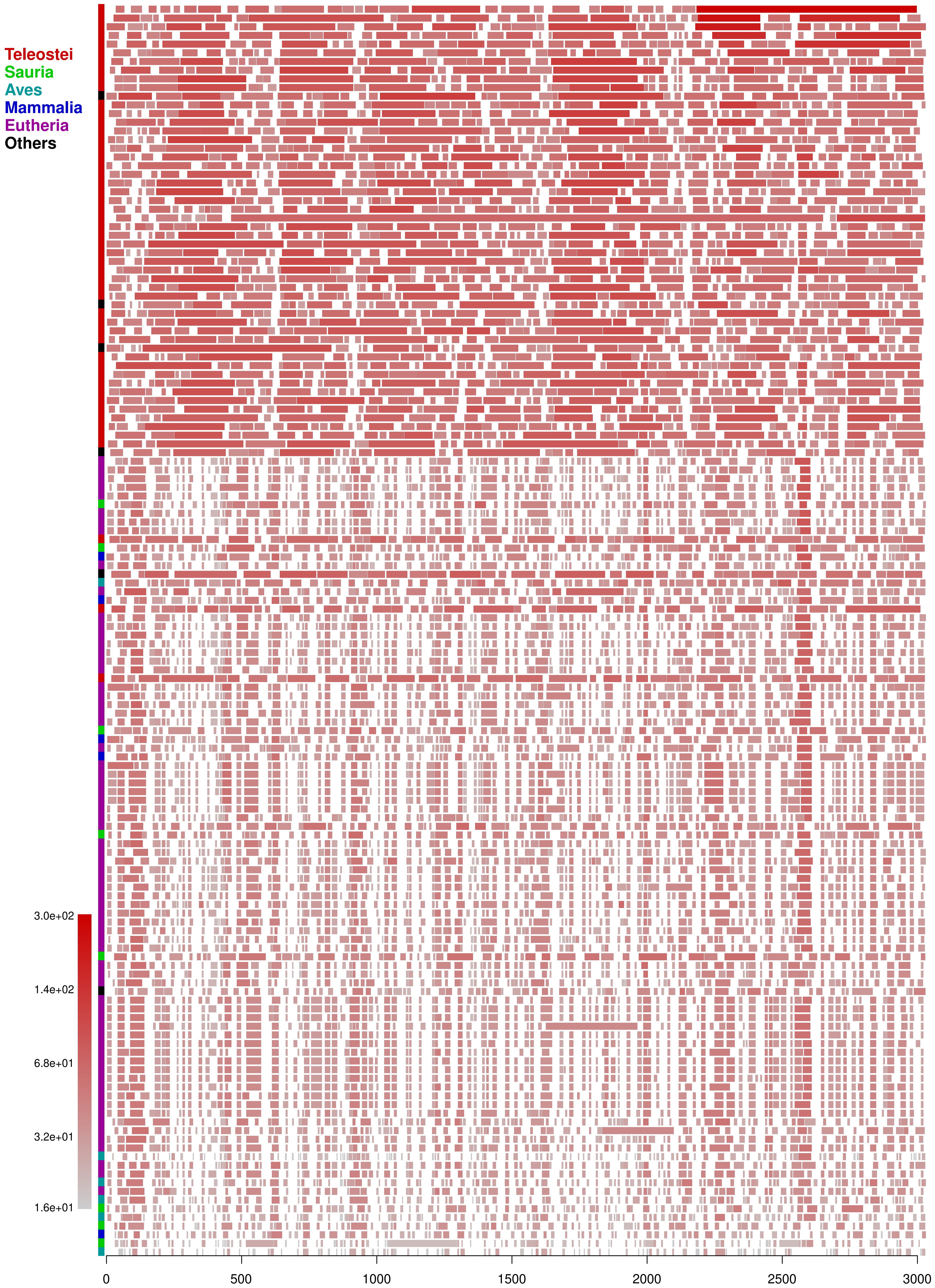

0 alignments above max size (1.0e+08)

ENSDARG00000045401 : ENSDART00000149744 intron no: 8 length: 3927

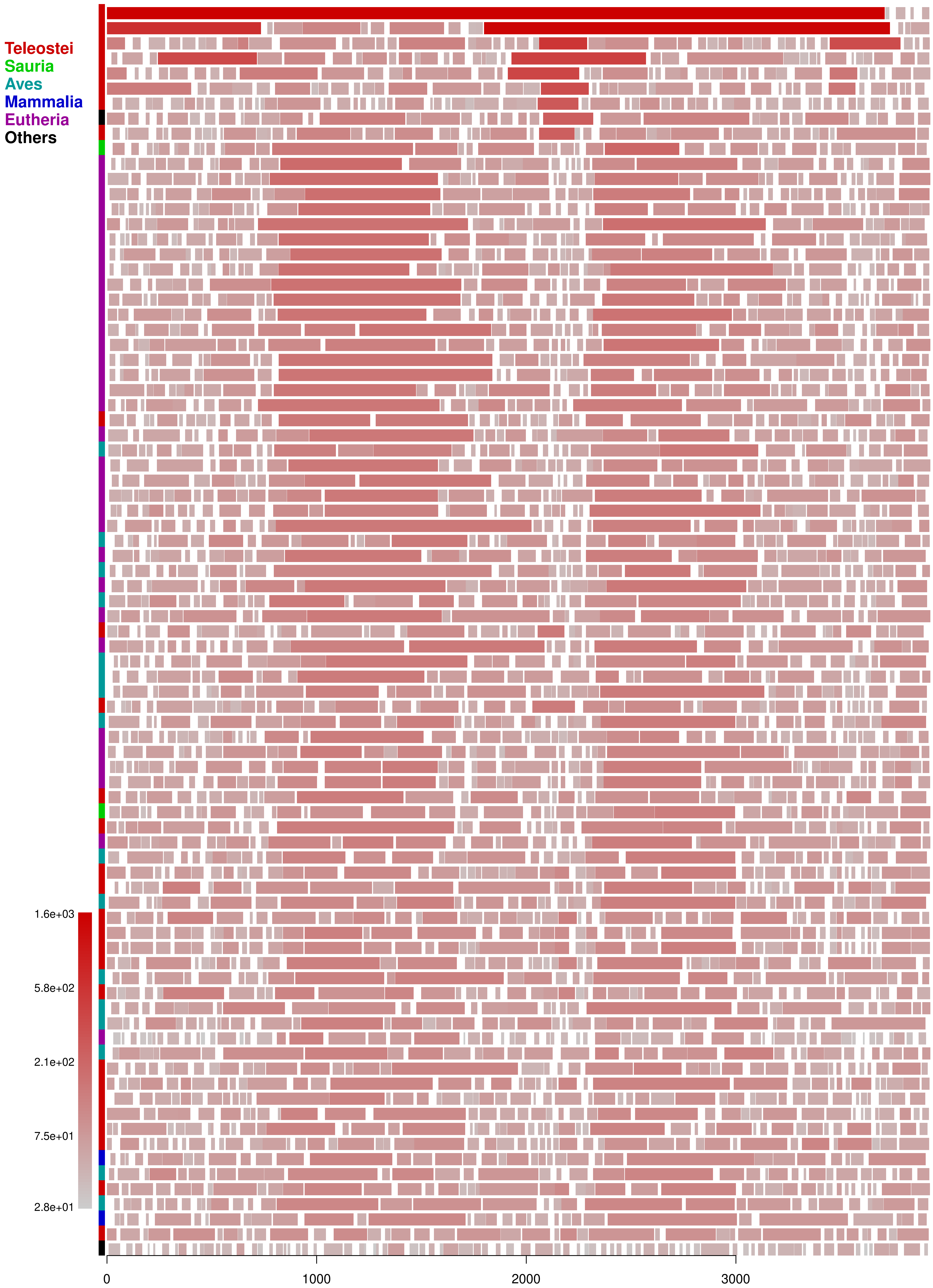

43 alignments above max size (1.0e+08)

ENSDARG00000098749 : ENSDART00000161419 intron no: 13 length: 7313

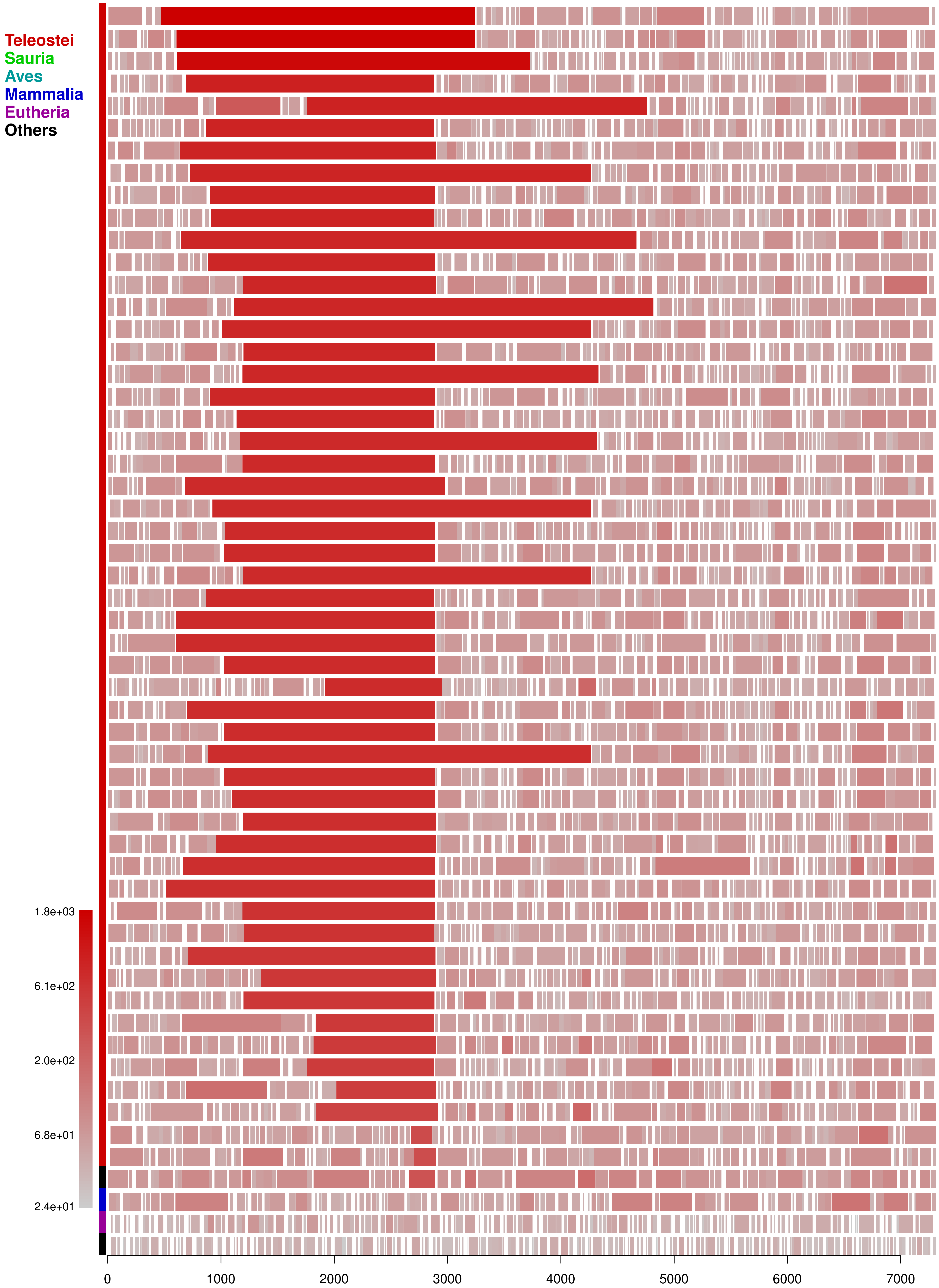

105 alignments above max size (1.0e+08)

ENSDARG00000113877 : ENSDART00000182896 intron no: 1 length: 1656

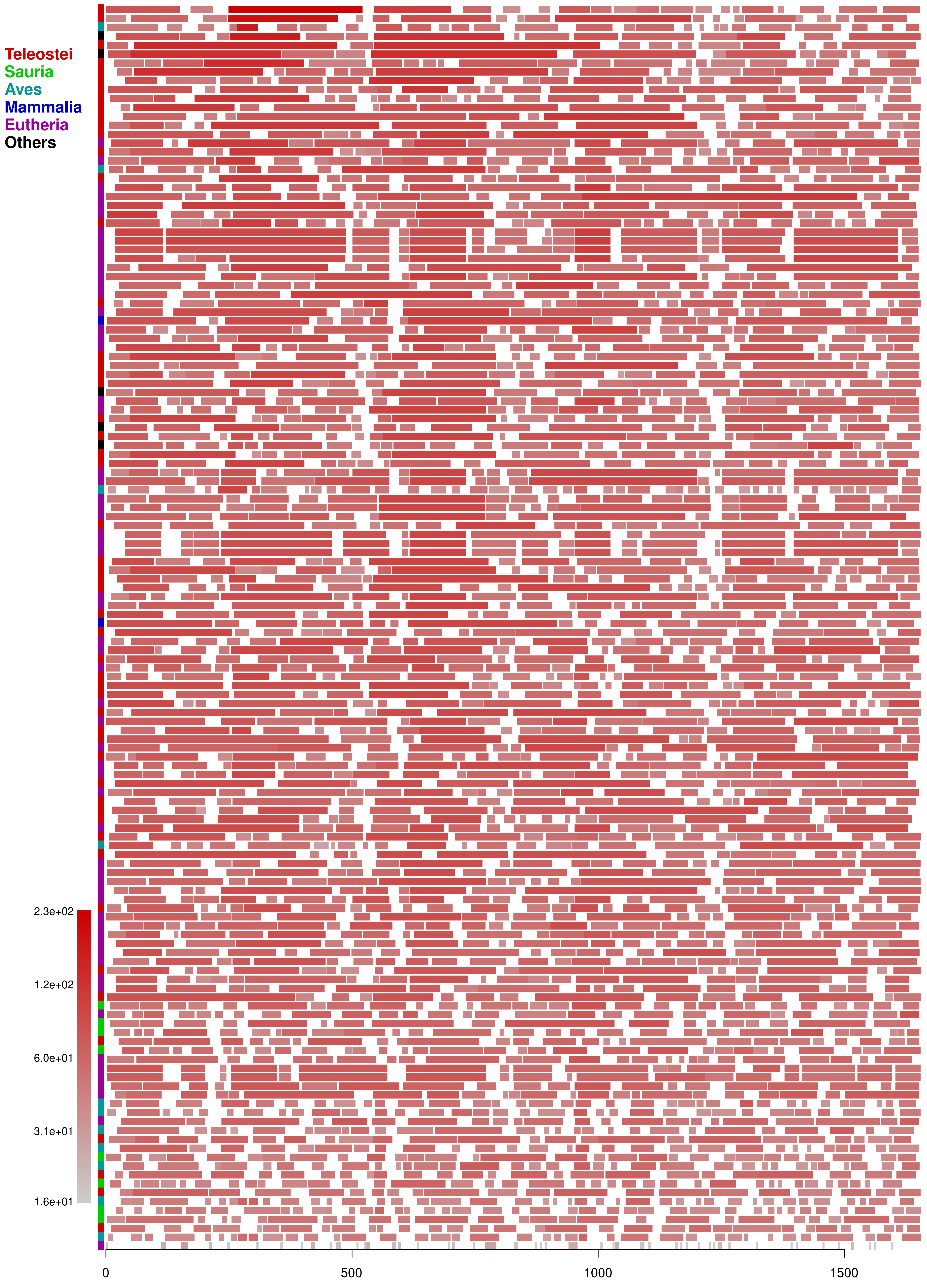

0 alignments above max size (1.0e+08)

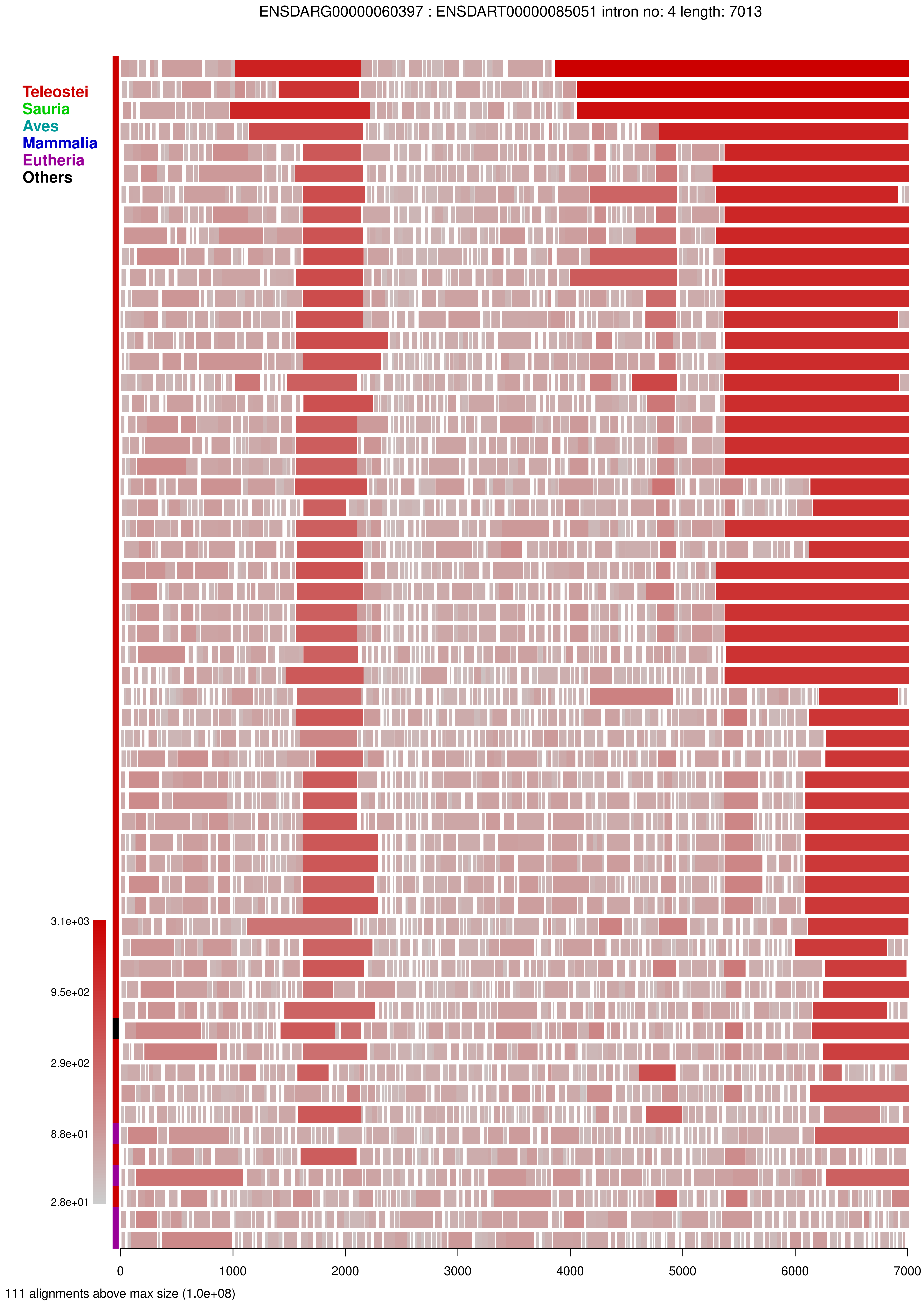
ENSDARG00000060397 : ENSDART00000085051 intron no: 4 length: 7013
Teleostei
Sauria
Aves
Mammalia
Eutheria
Others
3.1e+03
9.5e+02
2.9e+02
8.8e+01
2.8e+01
0
1000
2000
3000
4000
5000
6000
7000
111 alignments above max size (1.0e+08)

ENSDARG00000059768 : ENSDART00000151193 intron no: 3 length: 8449

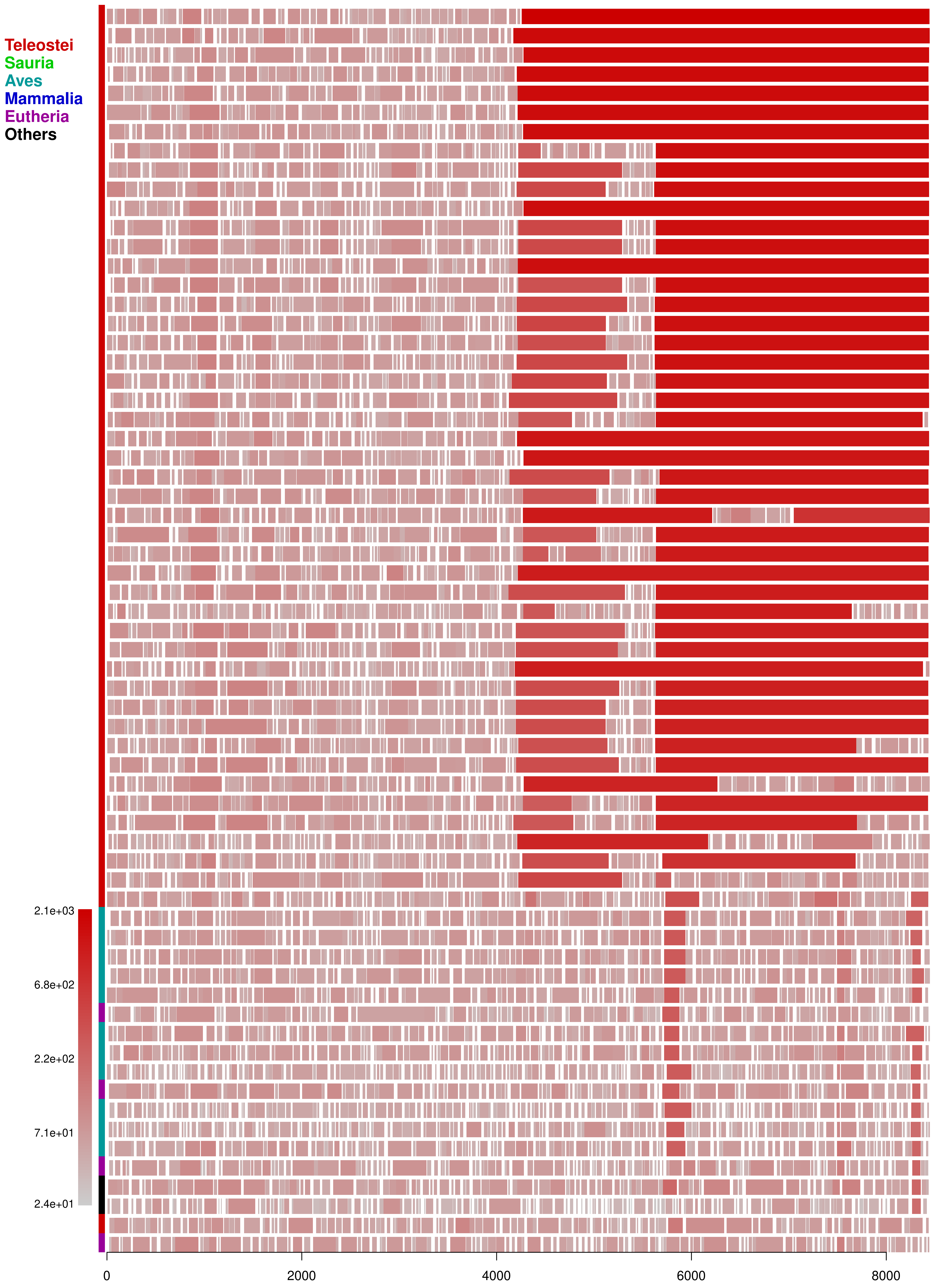

94 alignments above max size (1.0e+08)

ENSDARG00000059768 : ENSDART00000151193 intron no: 2 length: 11383

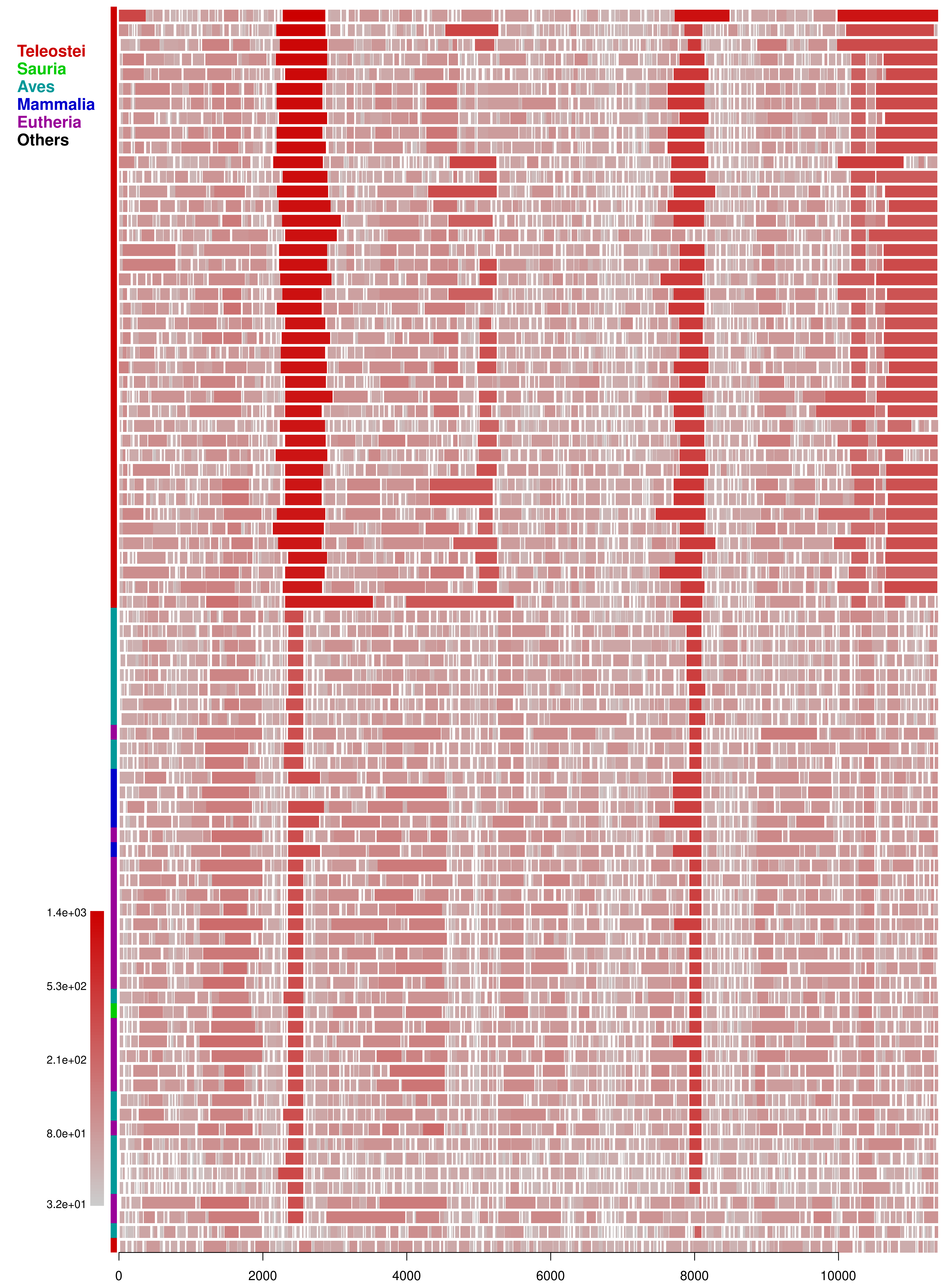

67 alignments above max size (1.0e+08)

ENSDARG00000034268 : ENSDART00000146299 intron no: 4 length: 121948

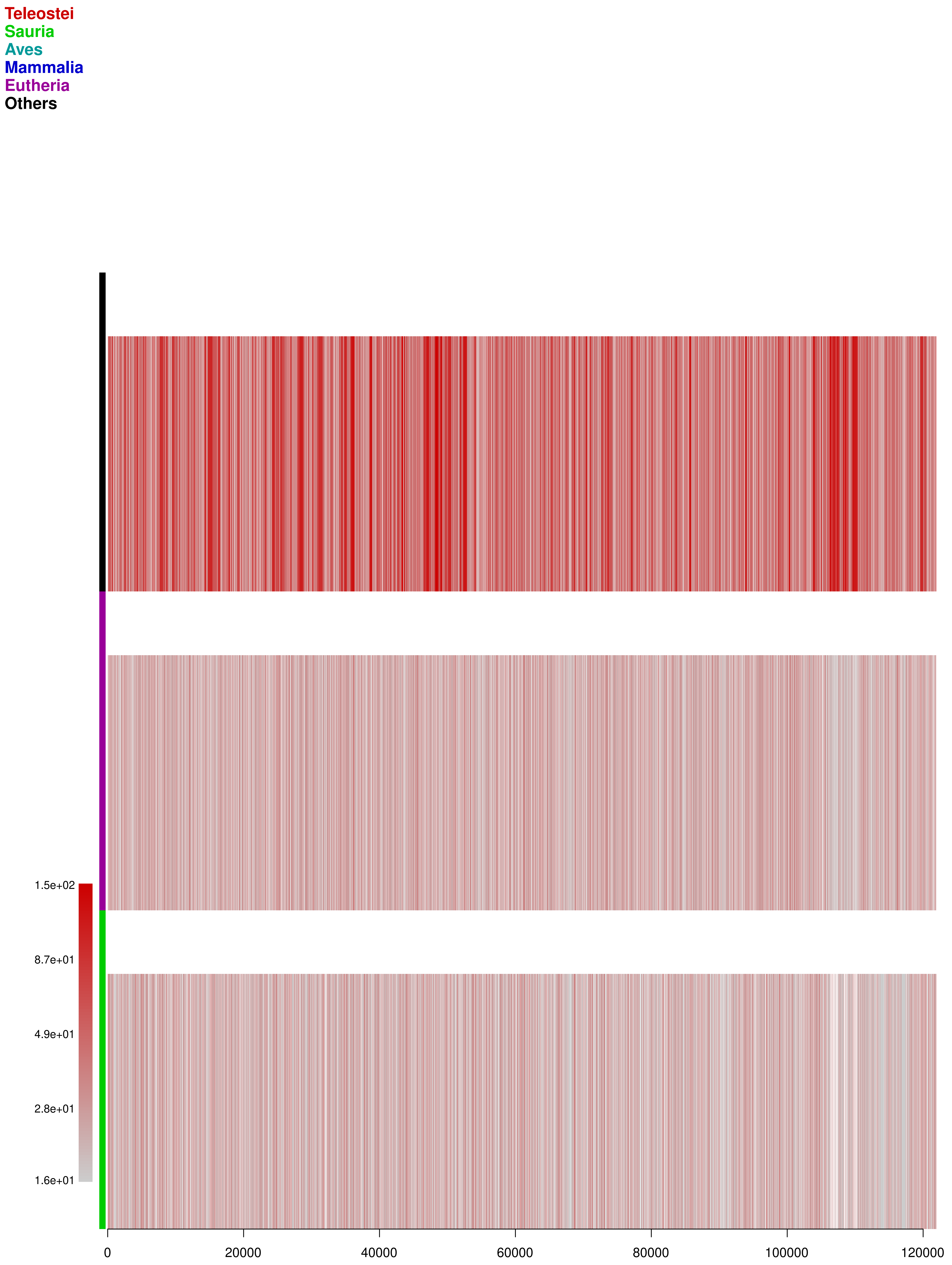

115 alignments above max size (1.0e+08)

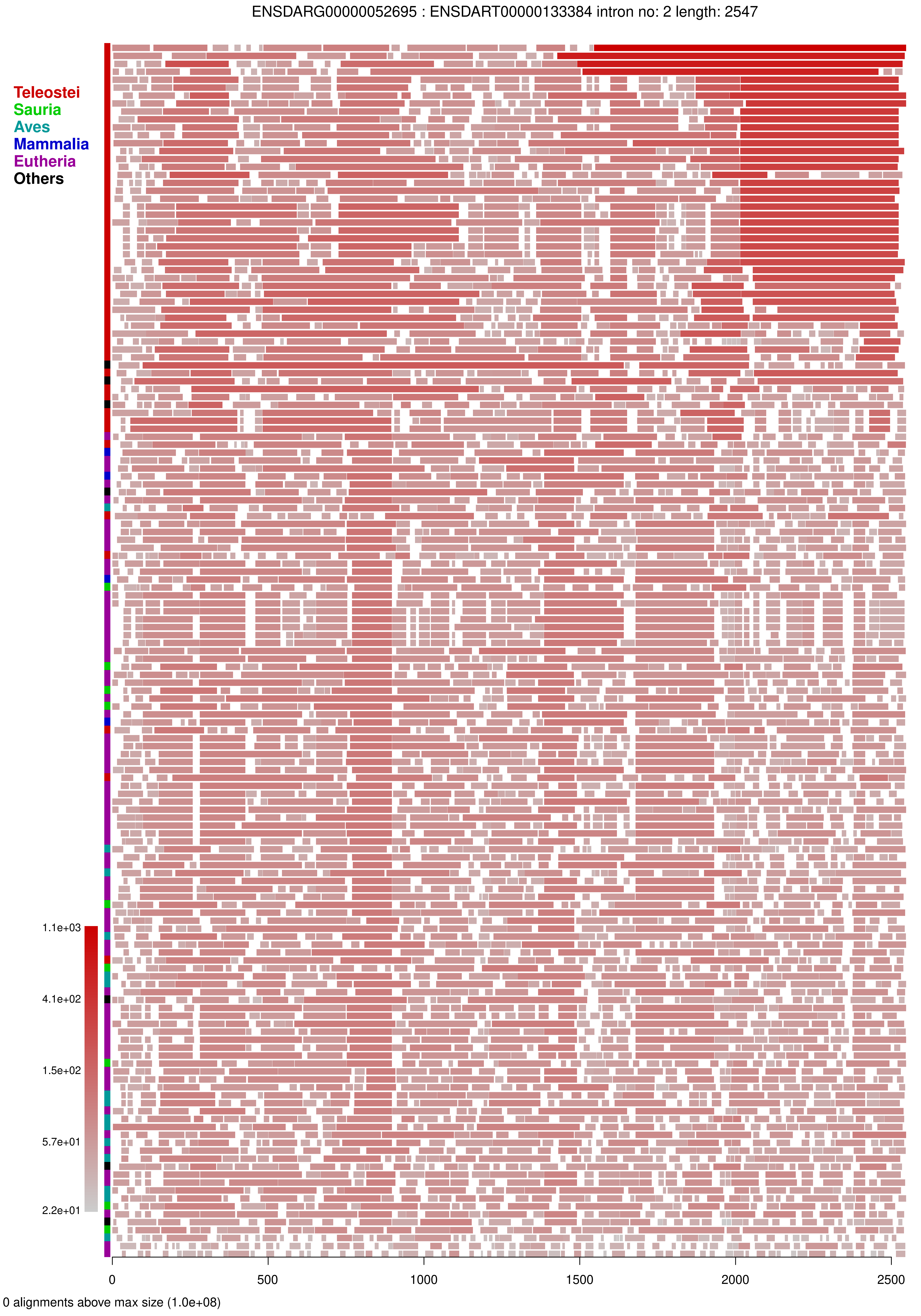
ENSDARG00000052695 : ENSDART00000133384 intron no: 2 length: 2547
Teleostei
Sauria
Aves
Mammalia
Eutheria
Others
1.1e+03
4.1e+02
1.5e+02
5.7e+01
2.2e+01
0
500
1000
1500
2000
2500
0 alignments above max size (1.0e+08)

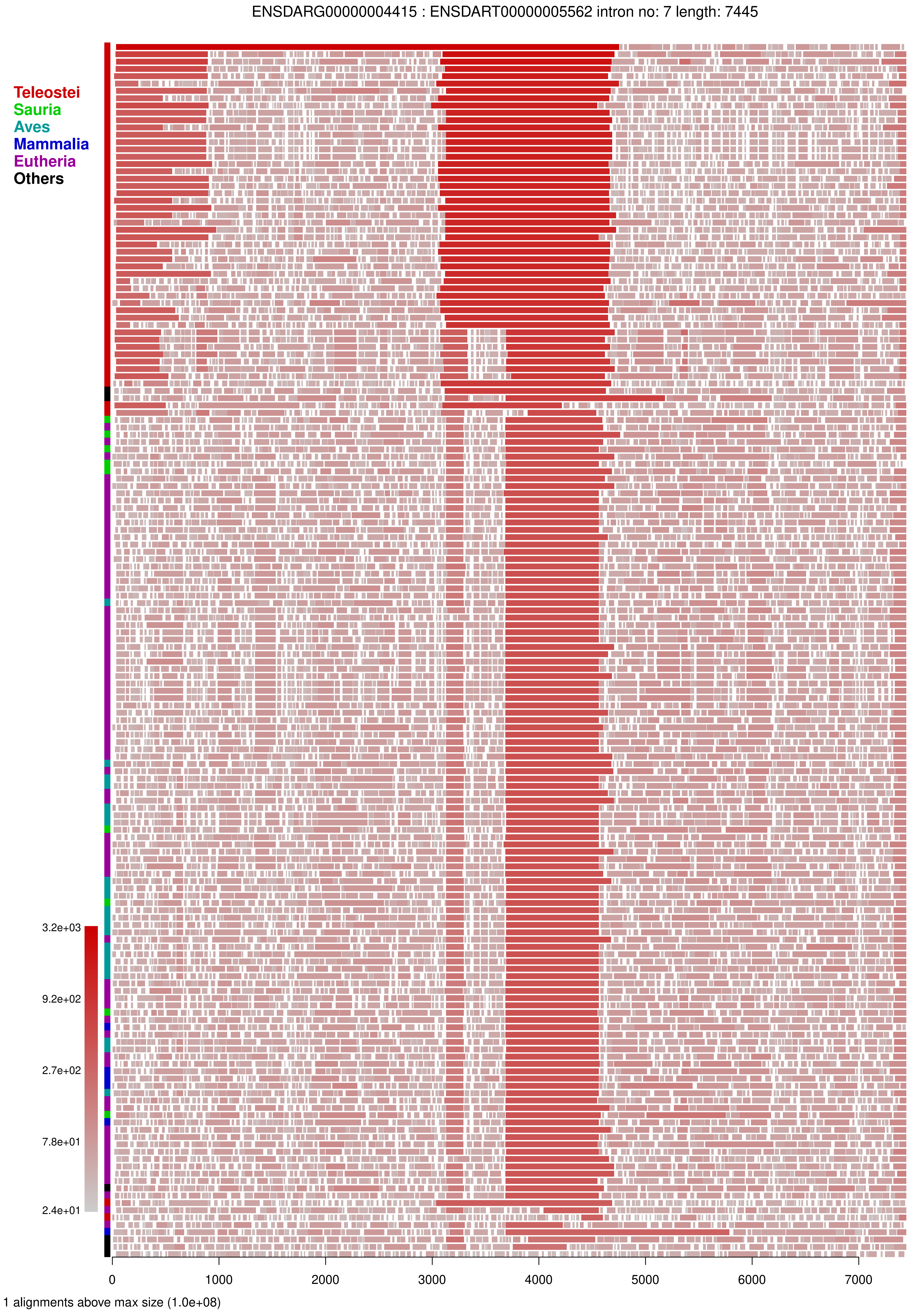

ENSDARG00000004415 : ENSDART00000005562 intron no: 7 length: 7445
Teleostei
Sauria
Aves
Mammalia
Eutheria
Others
3.2e+03
9.2e+02
2.7e+02
7.8e+01
2.4e+01
0
1000
2000
3000
4000
5000
6000
7000
1 alignments above max size (1.0e+08)

ENSDARG00000078355 : ENSDART00000109318 intron no: 1 length: 2792

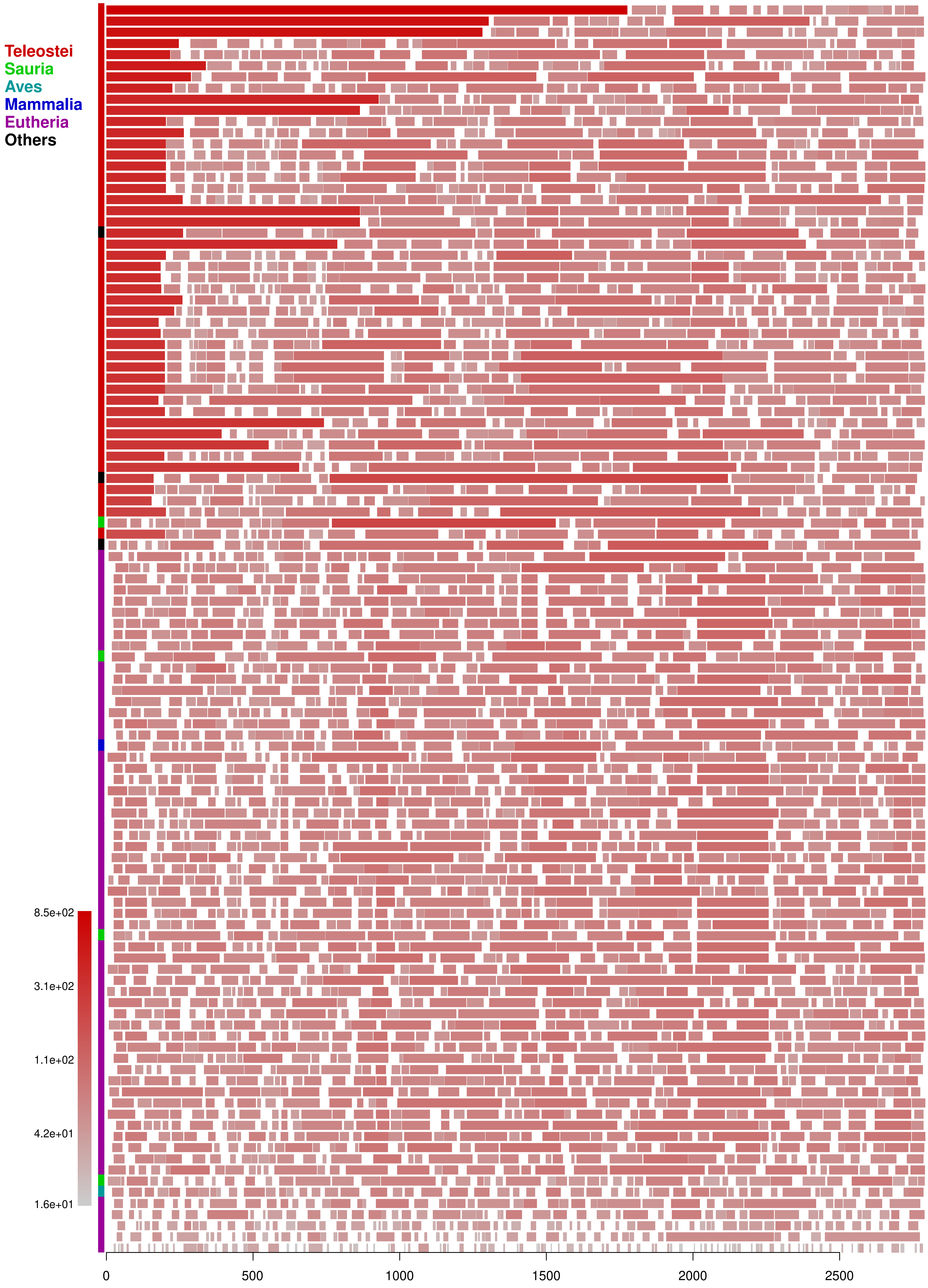

0 alignments above max size (1.0e+08)

ENSDARG00000099353 : ENSDART00000170343 intron no: 14 length: 7646

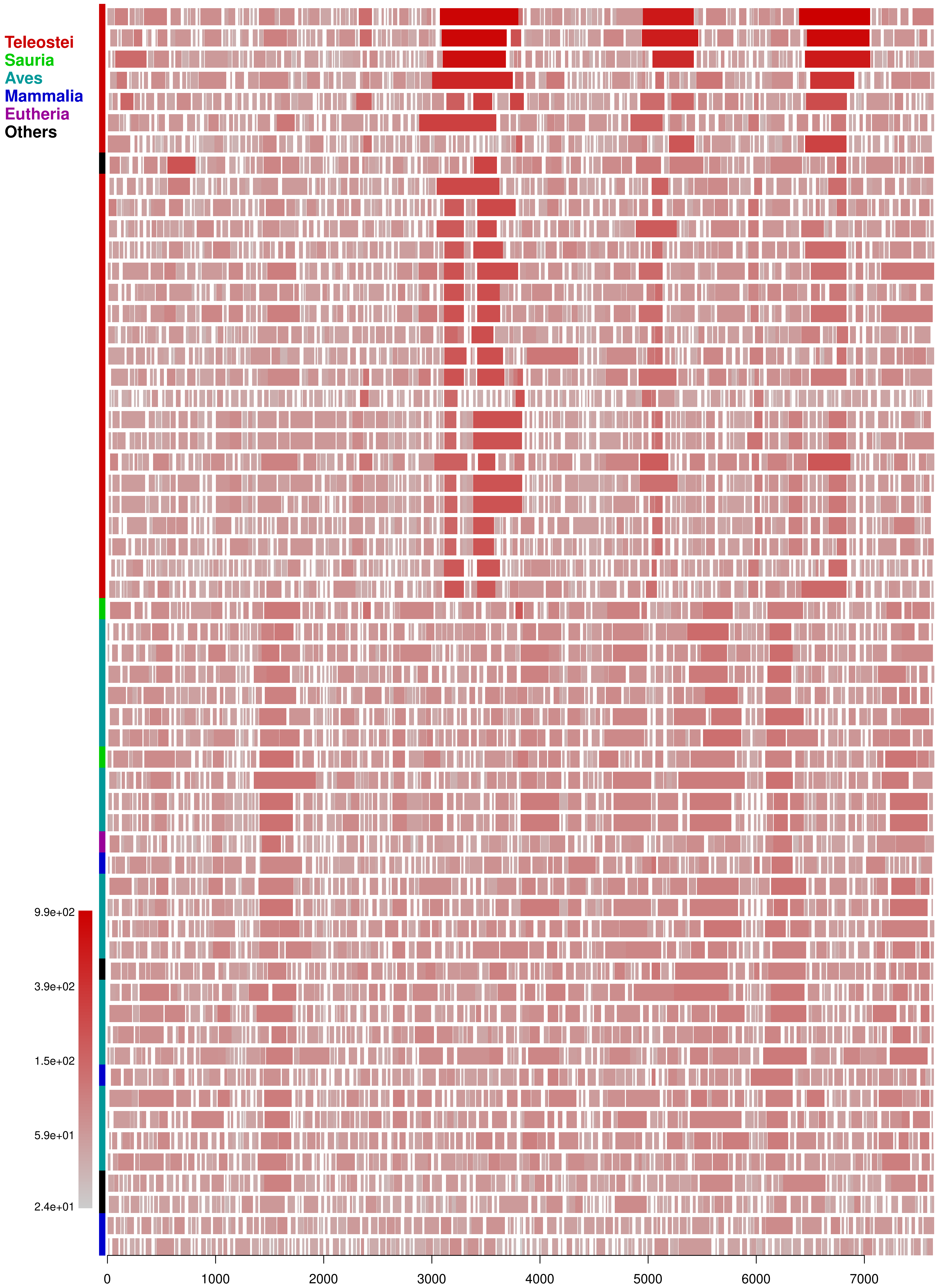

82 alignments above max size (1.0e+08)

ENSDARG00000037030 : ENSDART00000148257 intron no: 7 length: 4579

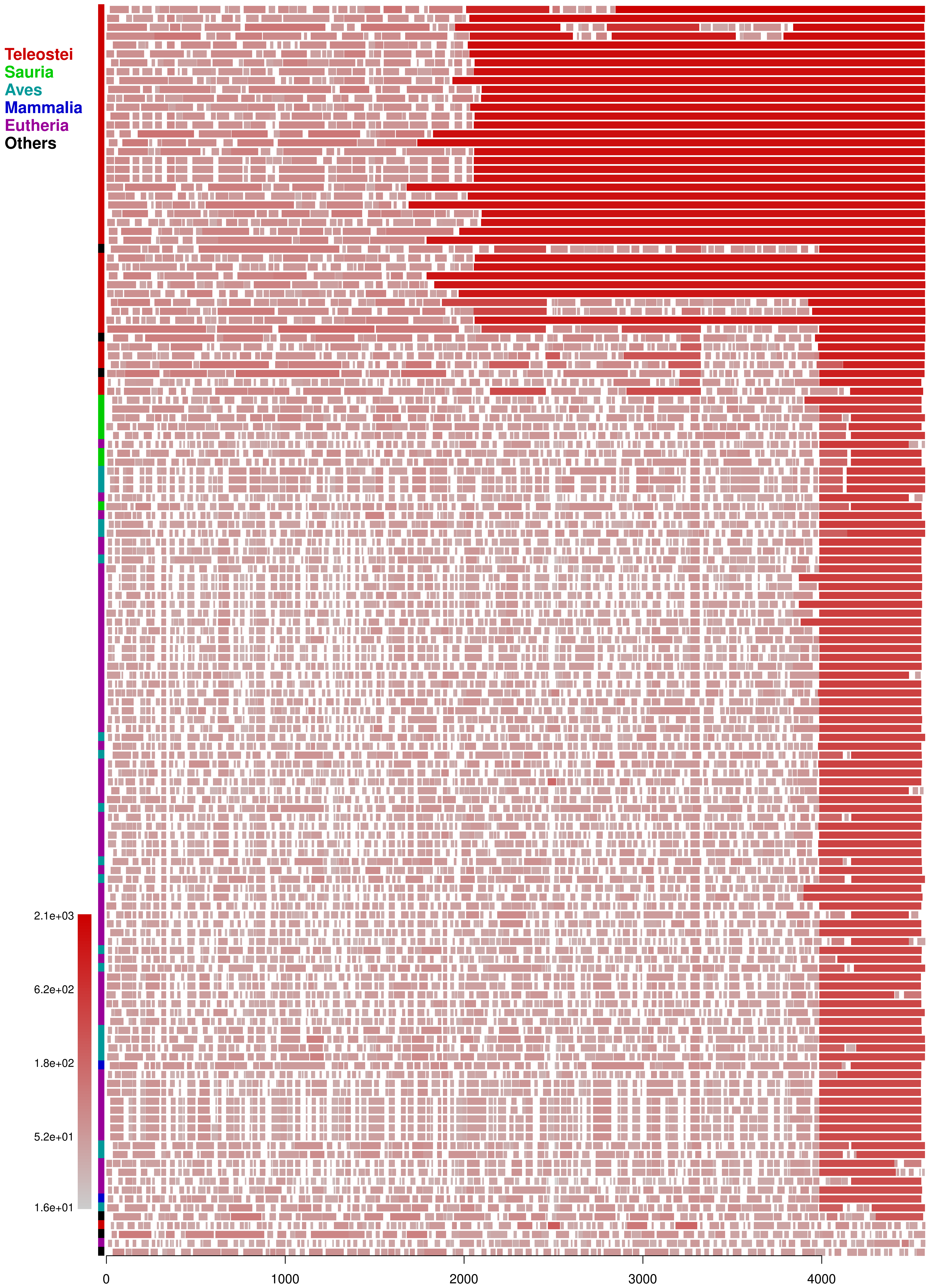

0 alignments above max size (1.0e+08)

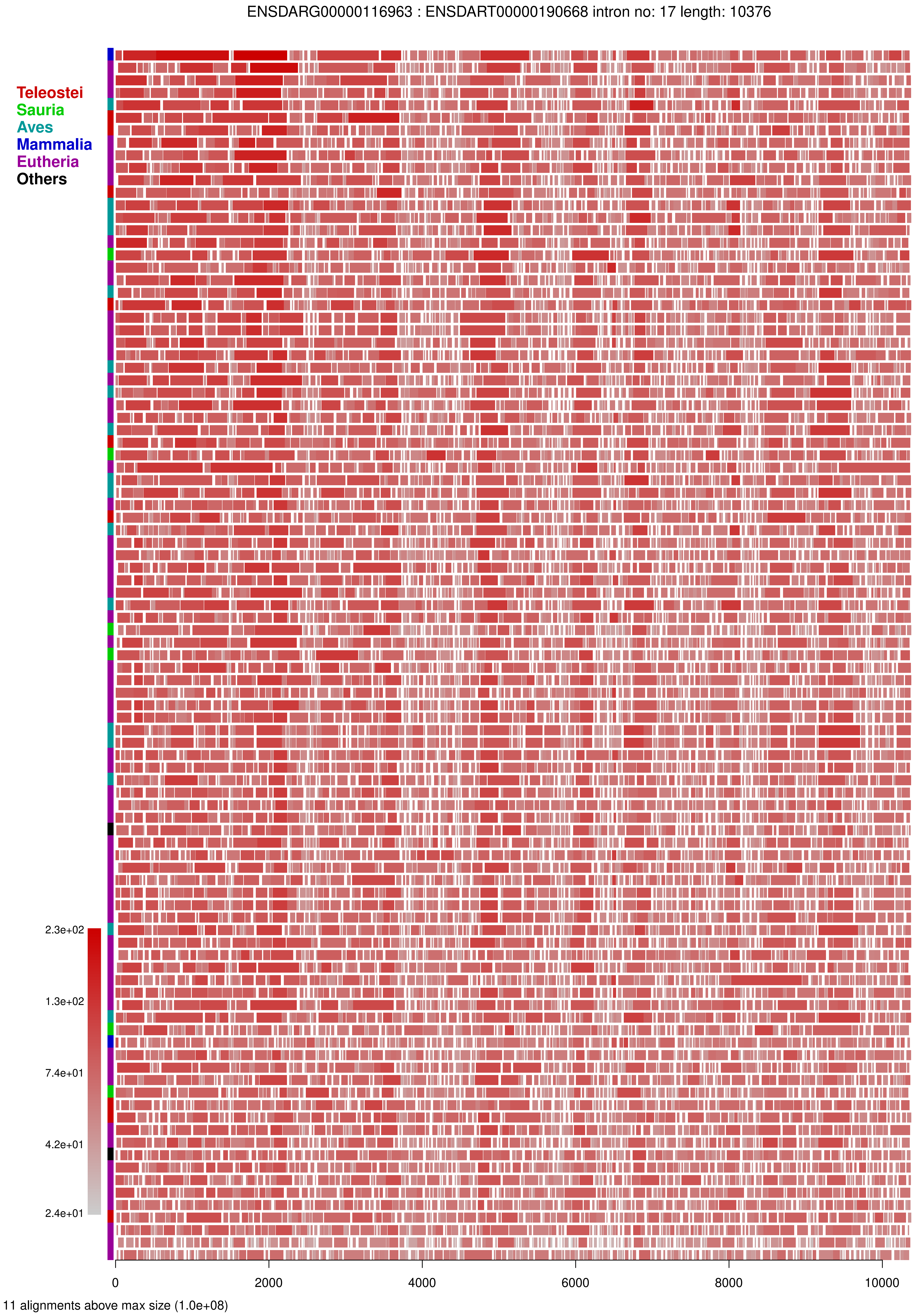
ENSDARG00000116963 : ENSDART00000190668 intron no: 17 length: 10376
Teleostei
Sauria
Aves
Mammalia
Eutheria
Others
2.3e+02
1.3e+02
7.4e+01
4.2e+01
2.4e+01
0
2000
4000
6000
8000
10000
11 alignments above max size (1.0e+08)

ENSDARG00000060323 : ENSDART00000148786 intron no: 4 length: 2164

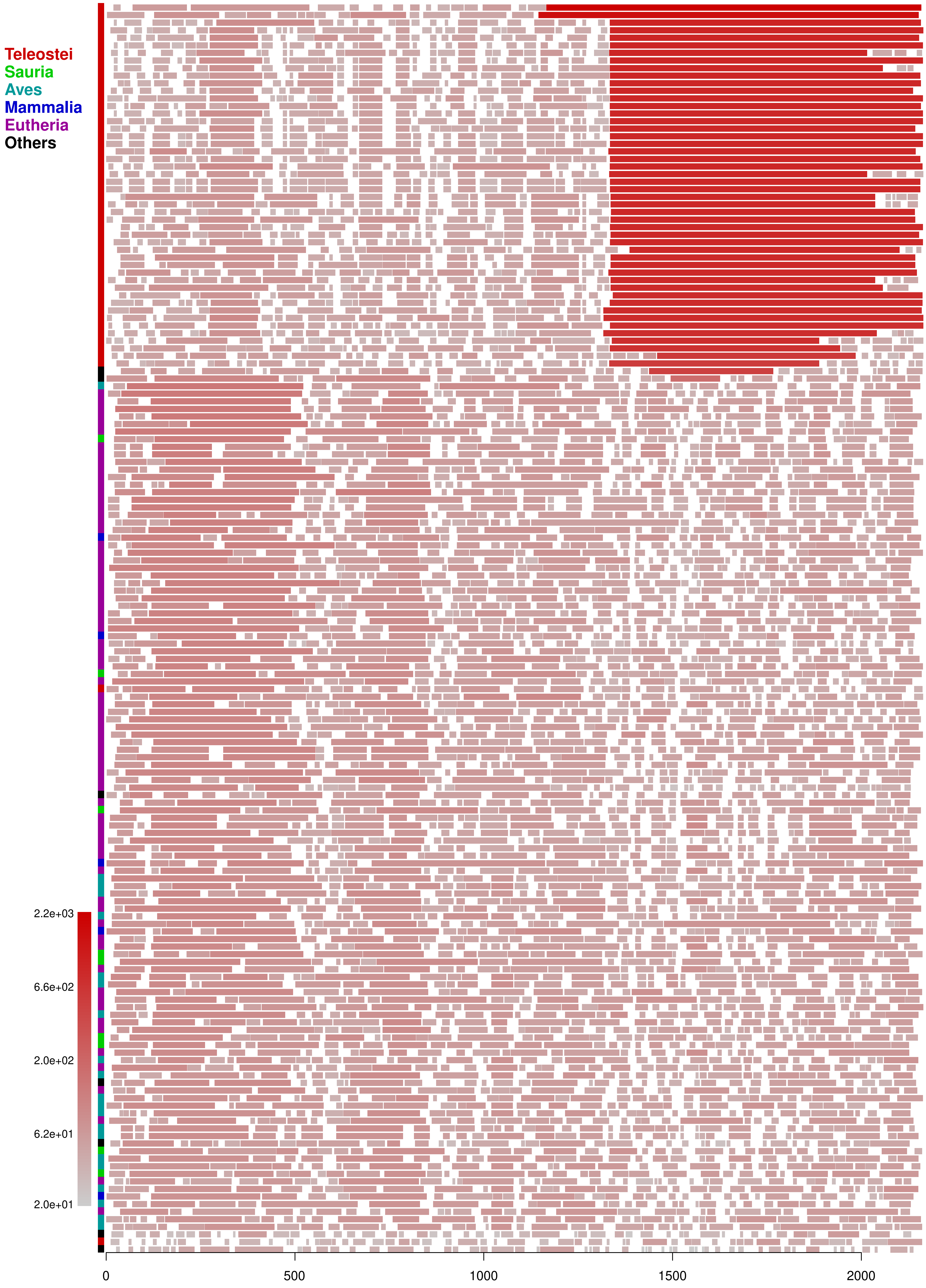

0 alignments above max size (1.0e+08)

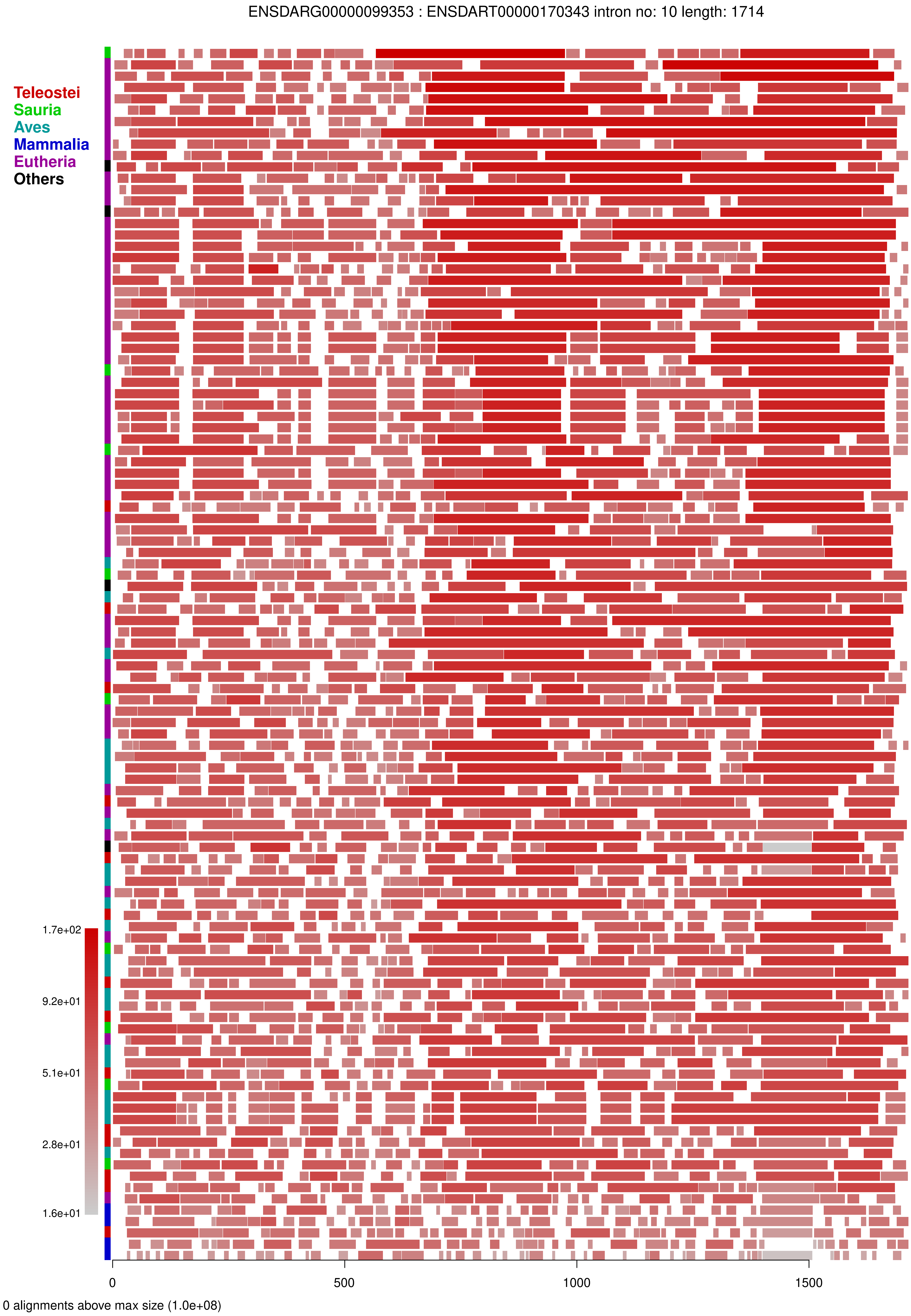
ENSDARG00000099353 : ENSDART00000170343 intron no: 10 length: 1714
Teleostei
Sauria
Aves
Mammalia
Eutheria
Others
1.7e+02
9.2e+01
5.1e+01
2.8e+01
1.6e+01
0
500
1000
1500
0 alignments above max size (1.0e+08)

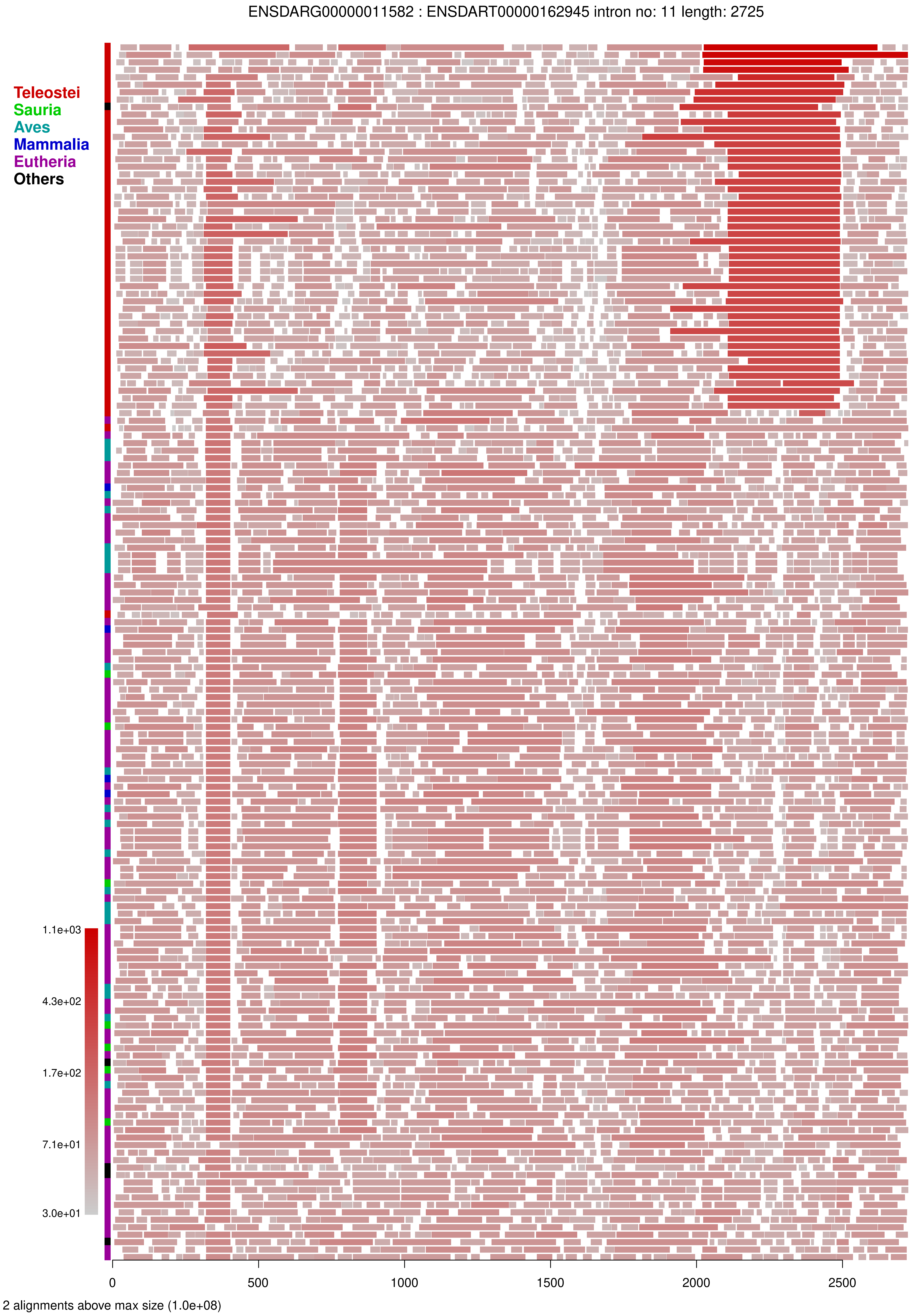

ENSDARG00000011582 : ENSDART00000162945 intron no: 11 length: 2725
Teleostei
Sauria
Aves
Mammalia
Eutheria
Others
1.1e+03
4.3e+02
1.7e+02
7.1e+01
3.0e+01
0
500
1000
1500
2000
2500
2 alignments above max size (1.0e+08)

ENSDARG00000073957 : ENSDART00000113342 intron no: 1 length: 39191

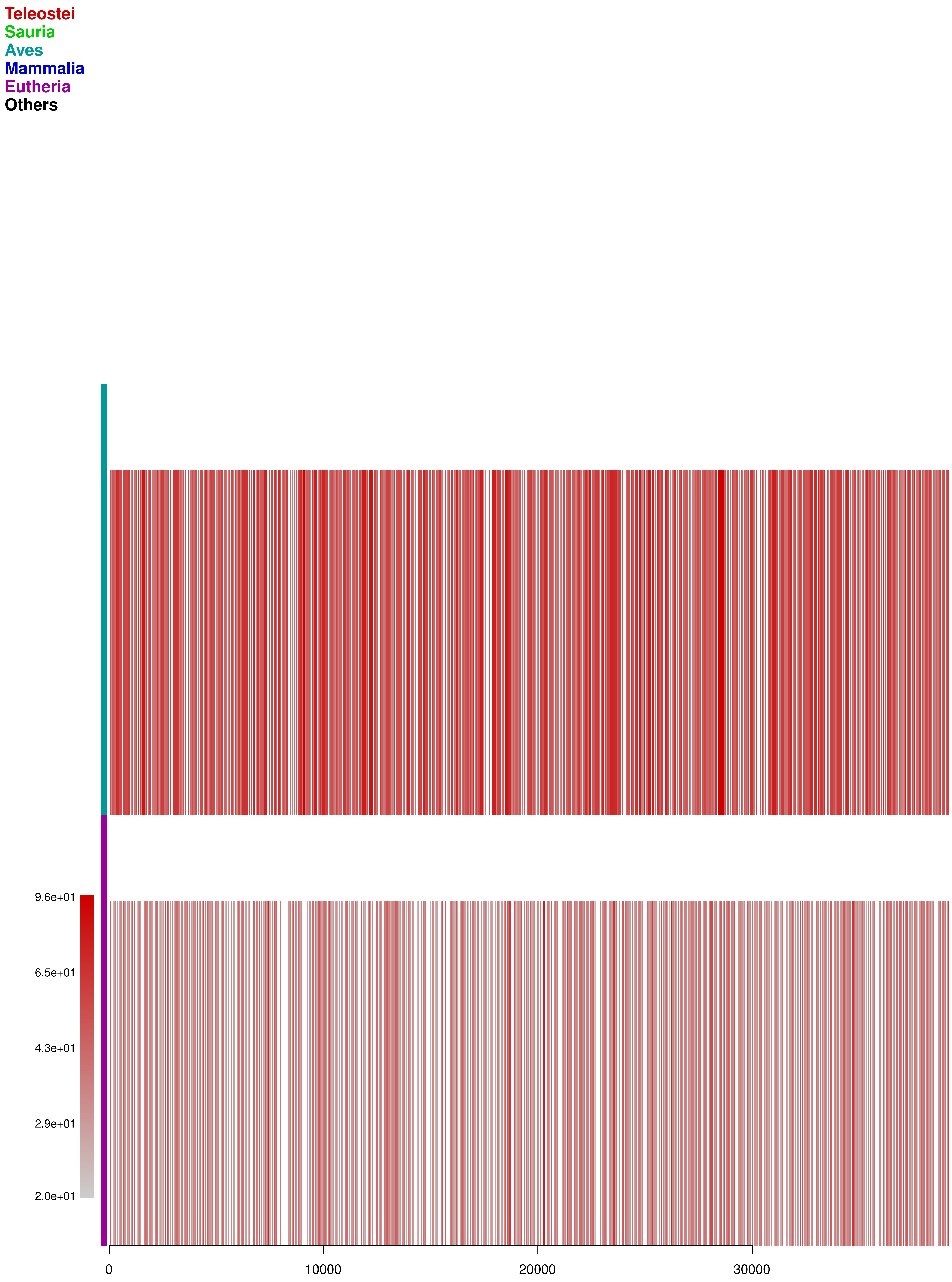

119 alignments above max size (1.0e+08)

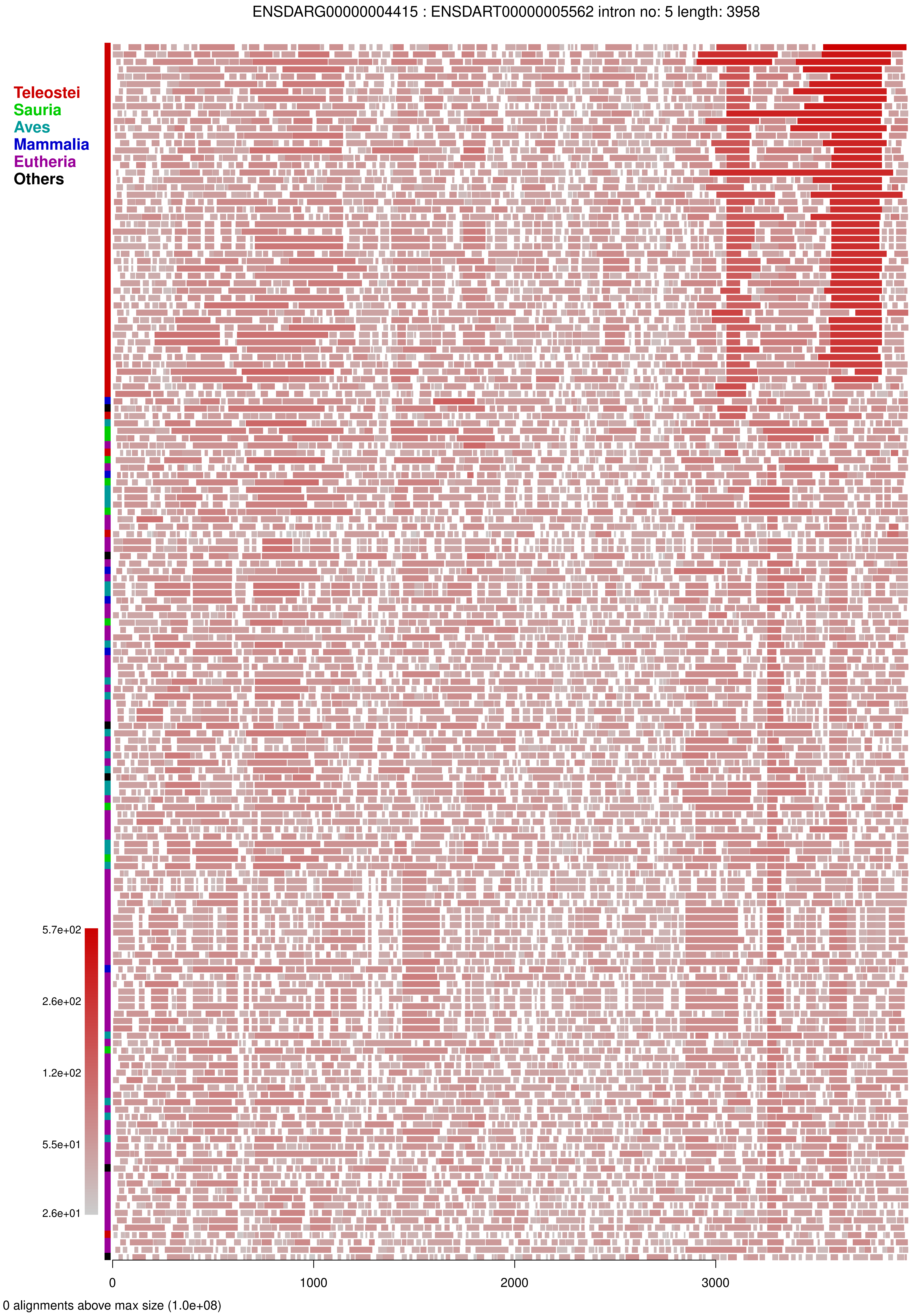
ENSDARG00000004415 : ENSDART00000005562 intron no: 5 length: 3958
Teleostei
Sauria
Aves
Mammalia
Eutheria
Others
5.7e+02
2.6e+02
1.2e+02
5.5e+01
2.6e+01
0
1000
2000
3000
0 alignments above max size (1.0e+08)

ENSDARG00000037030 : ENSDART00000148257 intron no: 4 length: 5366

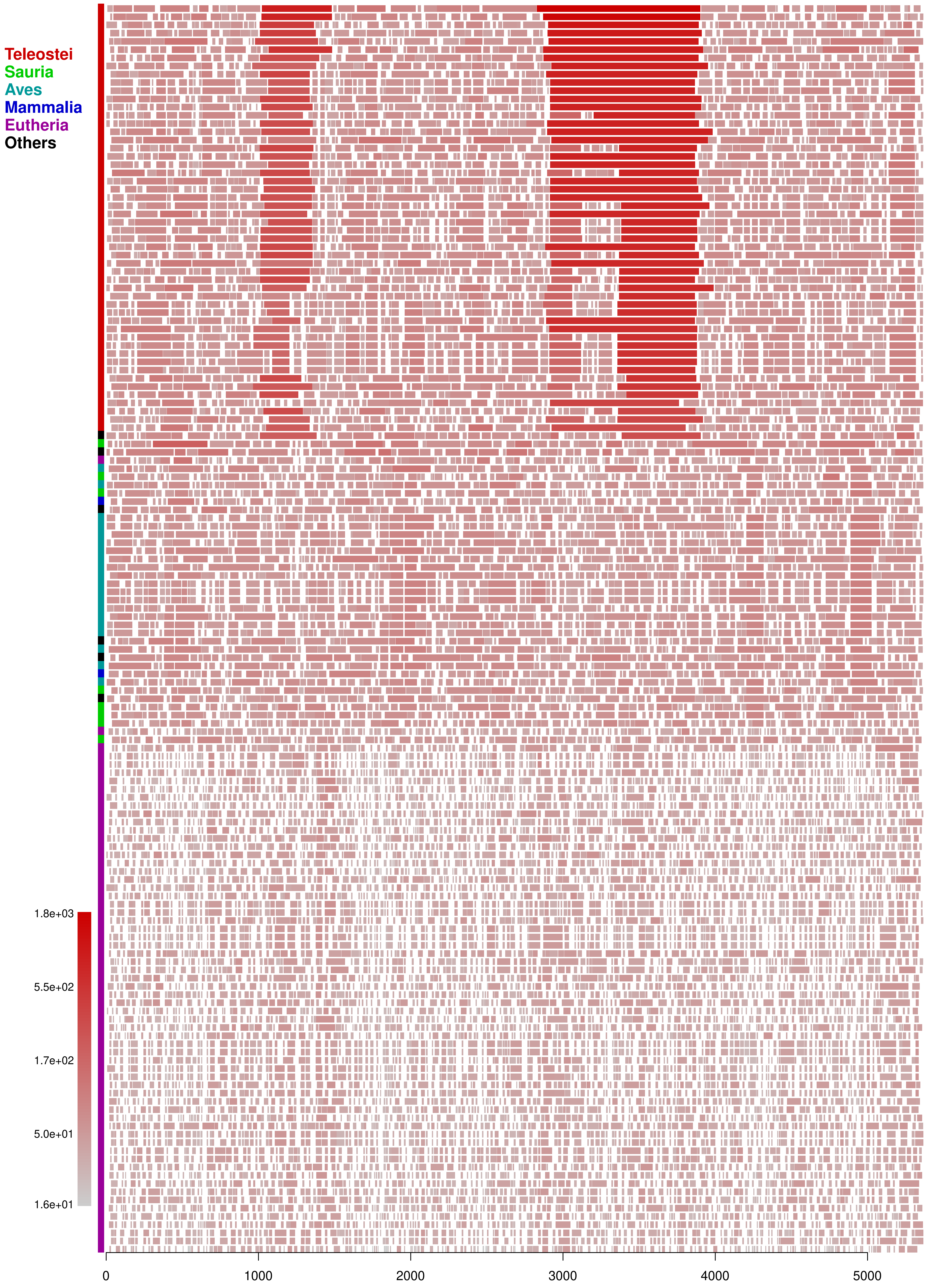

0 alignments above max size (1.0e+08)

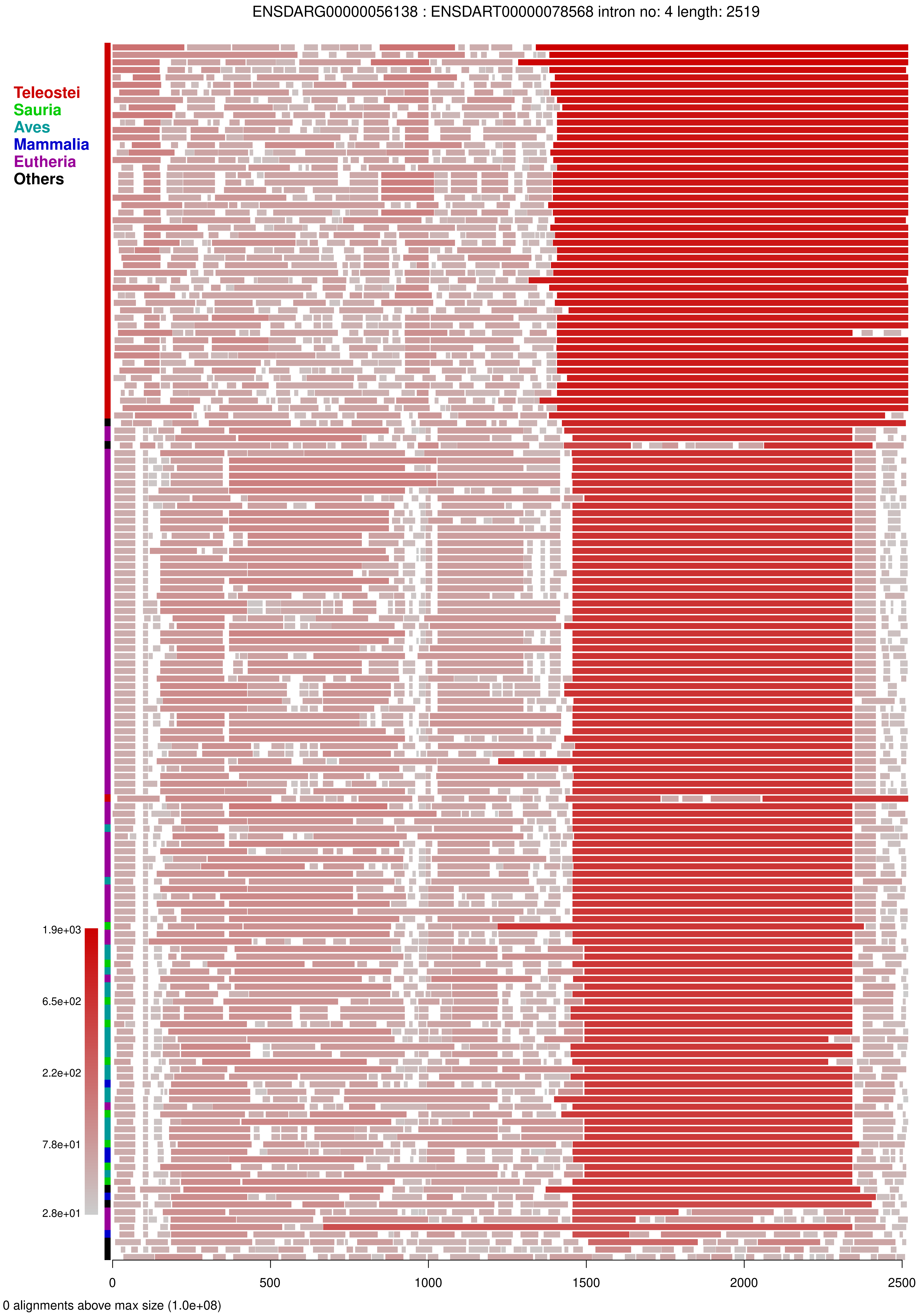

ENSDARG00000056138 : ENSDART00000078568 intron no: 4 length: 2519
Teleostei
Sauria
Aves
Mammalia
Eutheria
Others
1.9e+03
6.5e+02
2.2e+02
7.8e+01
2.8e+01
0
500
1000
1500
2000
2500
0 alignments above max size (1.0e+08)

ENSDARG00000018904 : ENSDART00000025423 intron no: 3 length: 5247

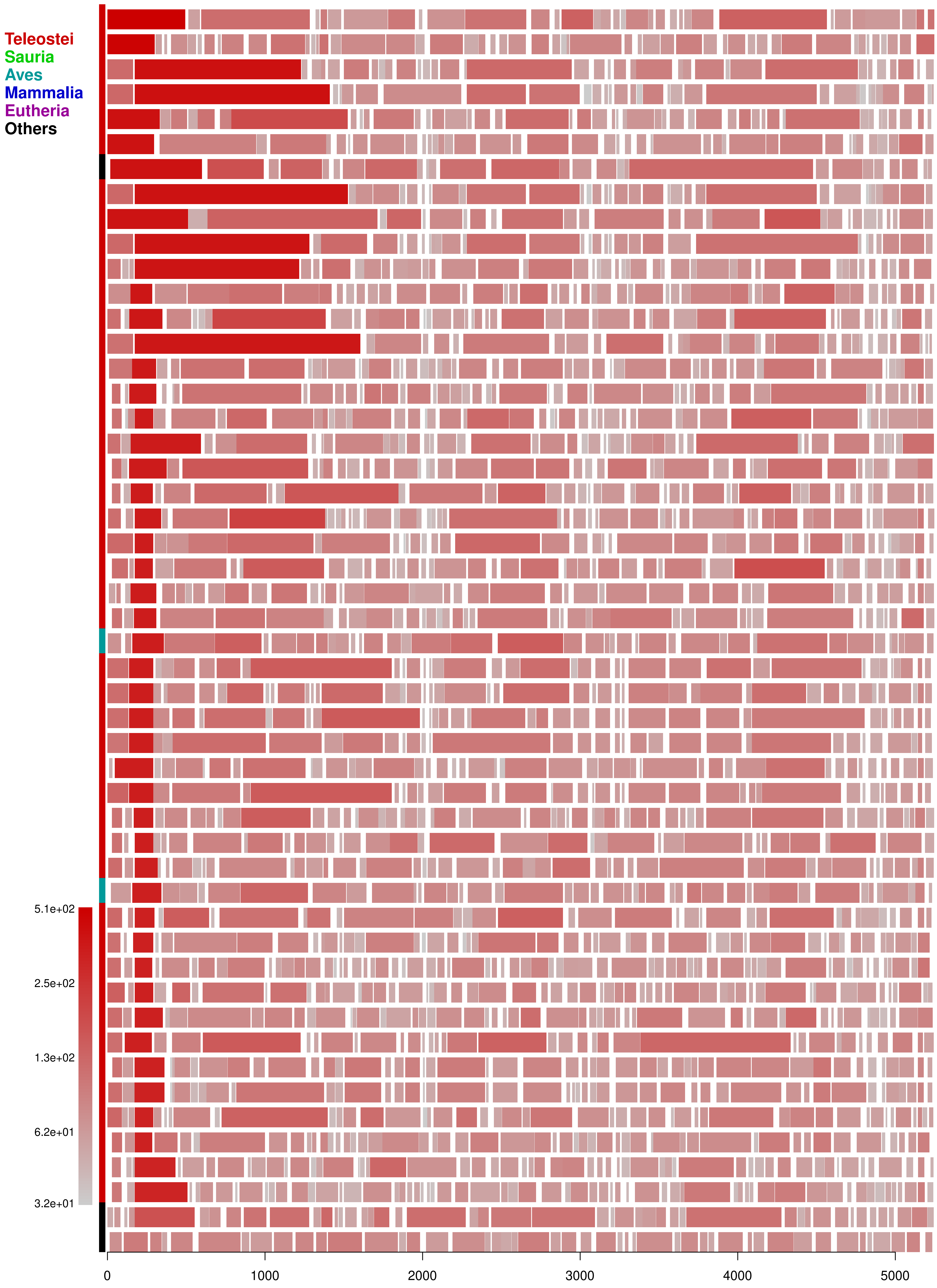

104 alignments above max size (1.0e+08)

ENSDARG00000058702 : ENSDART00000081620 intron no: 1 length: 3995

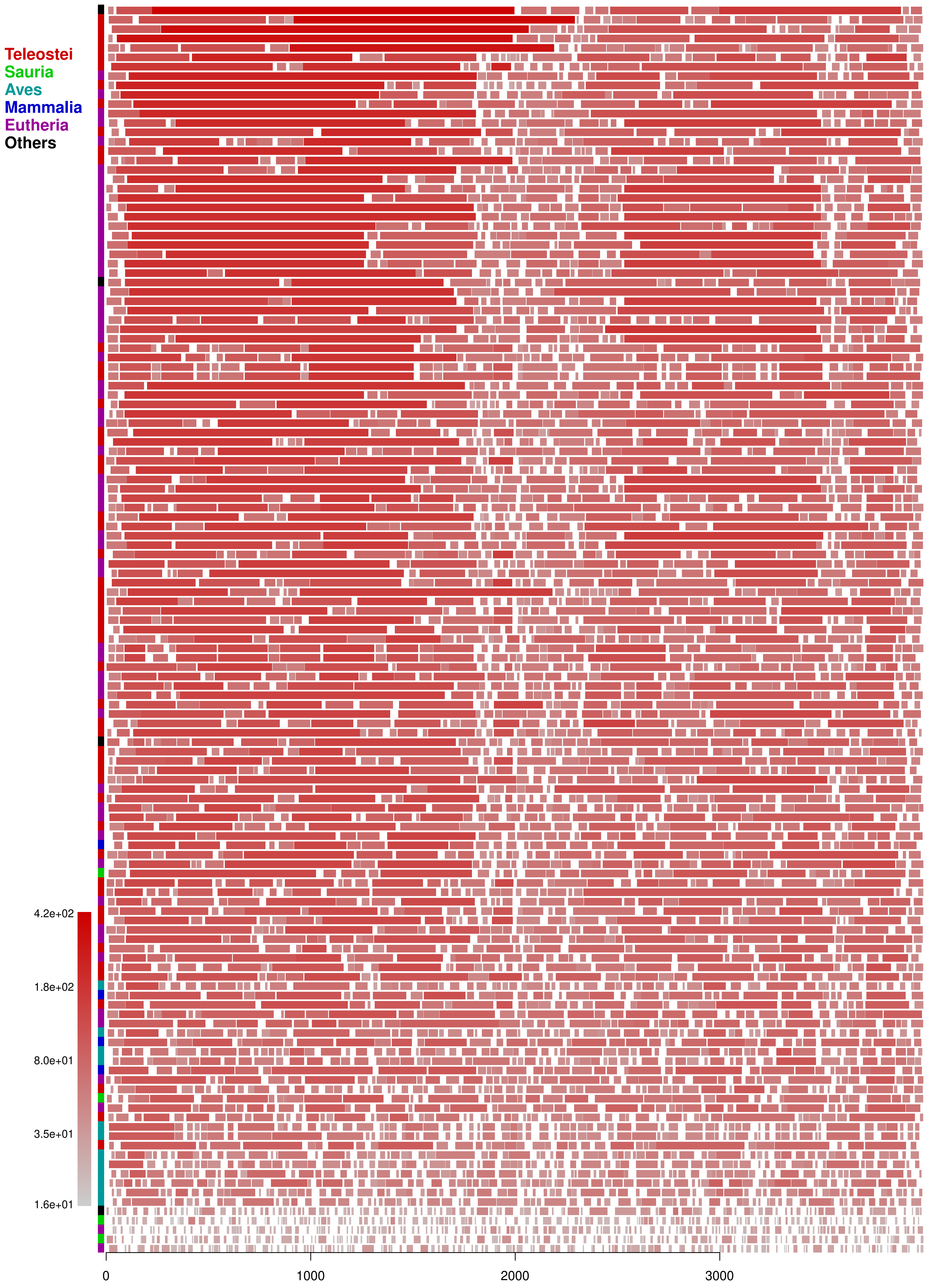

3 alignments above max size (1.0e+08)

ENSDARG00000031108 : ENSDART00000177989 intron no: 35 length: 24239

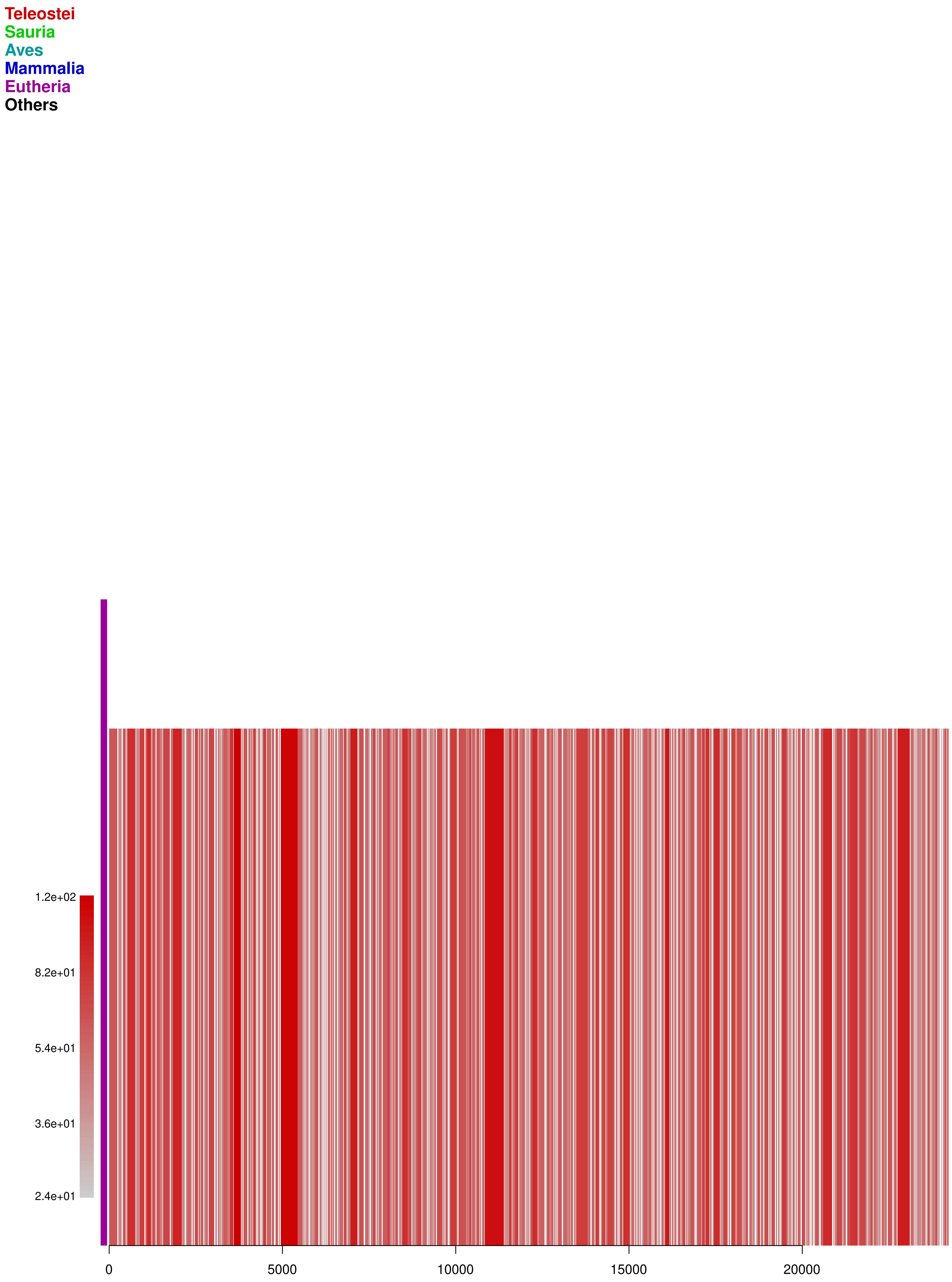

155 alignments above max size (1.0e+08)

ENSDARG00000037964 : ENSDART00000134360 intron no: 3 length: 1893

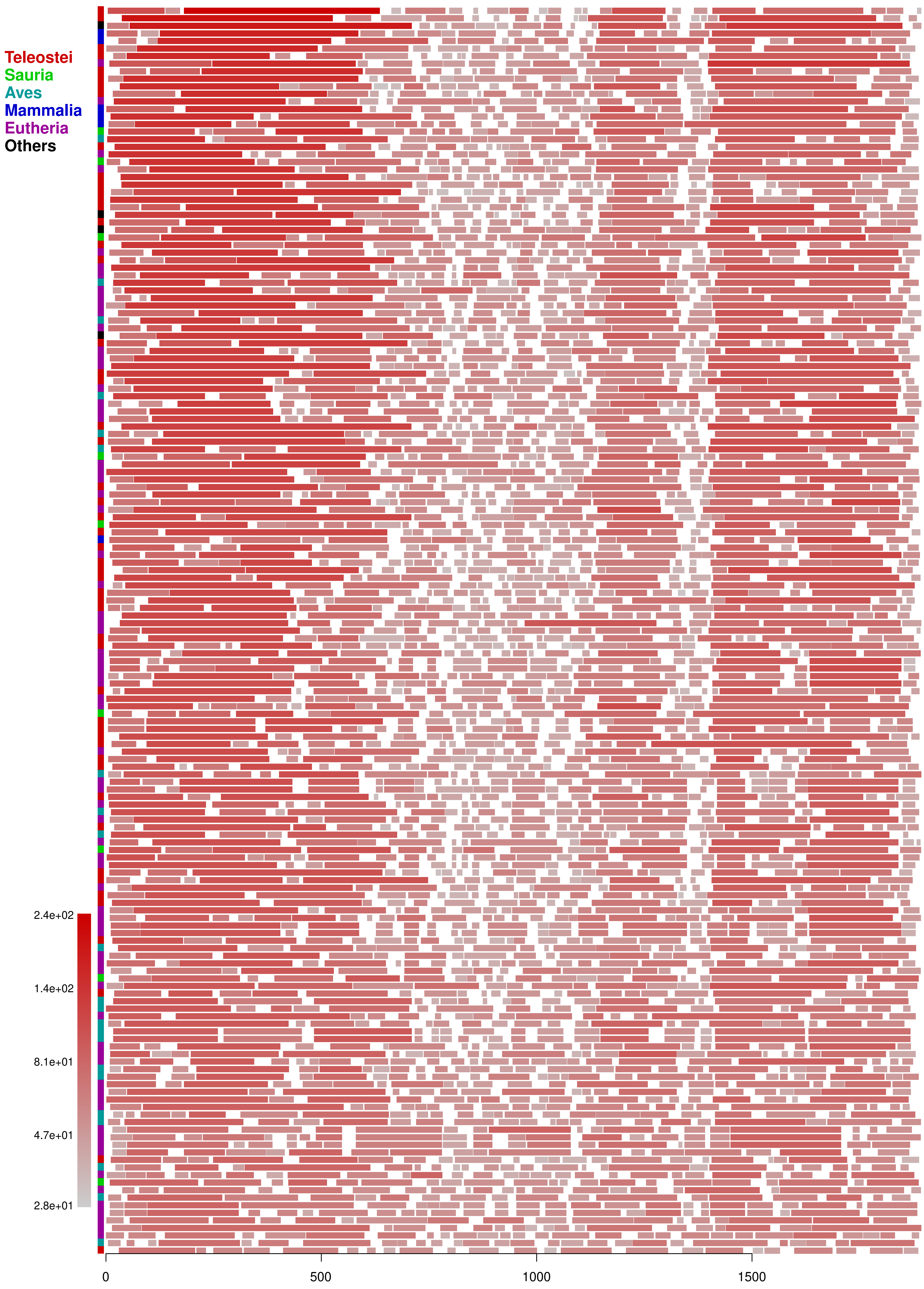

0 alignments above max size (1.0e+08)

ENSDARG00000024694 : ENSDART00000138204 intron no: 13 length: 3114

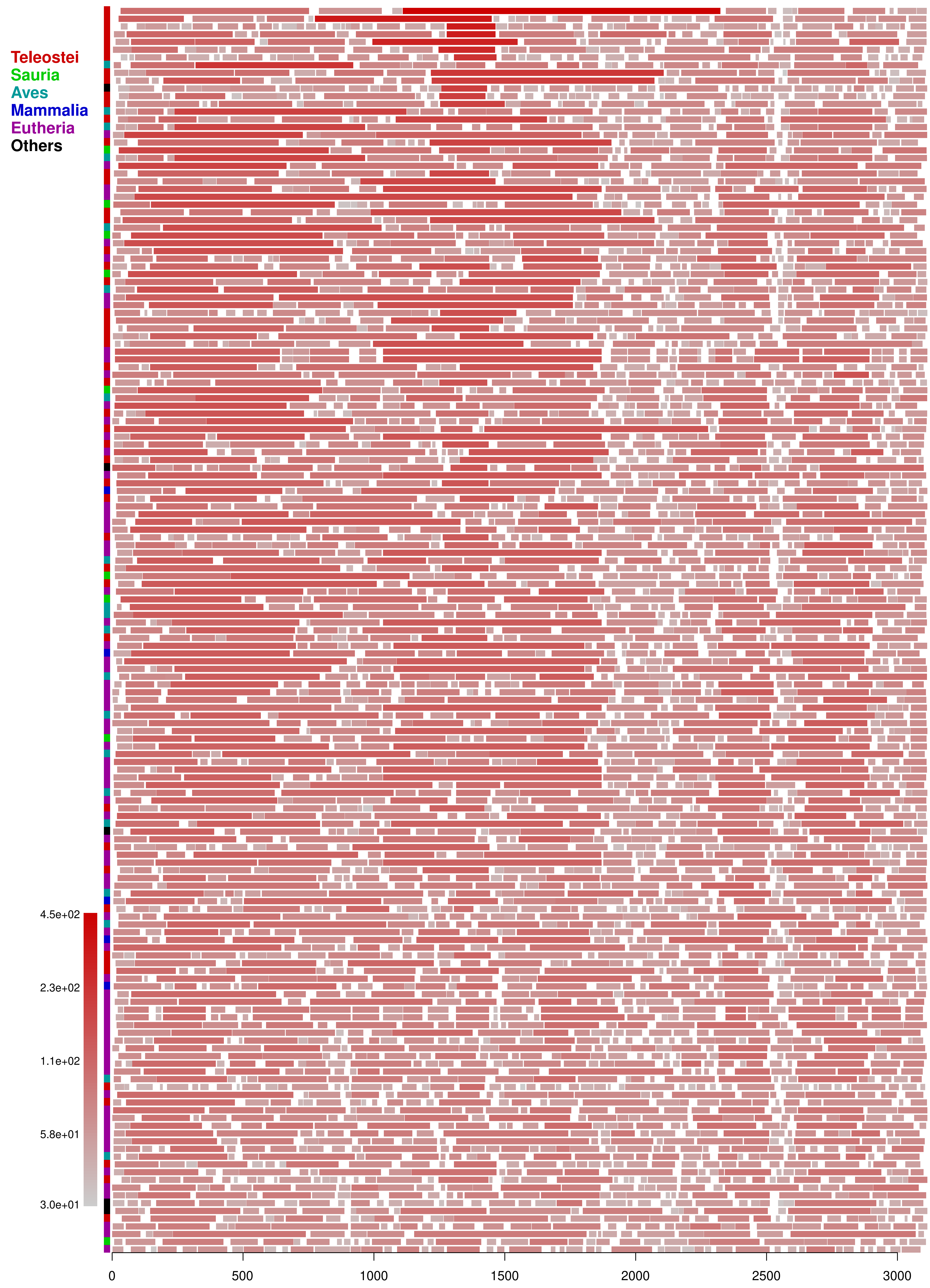

0 alignments above max size (1.0e+08)

ENSDARG00000014420 : ENSDART00000028610 intron no: 3 length: 4257

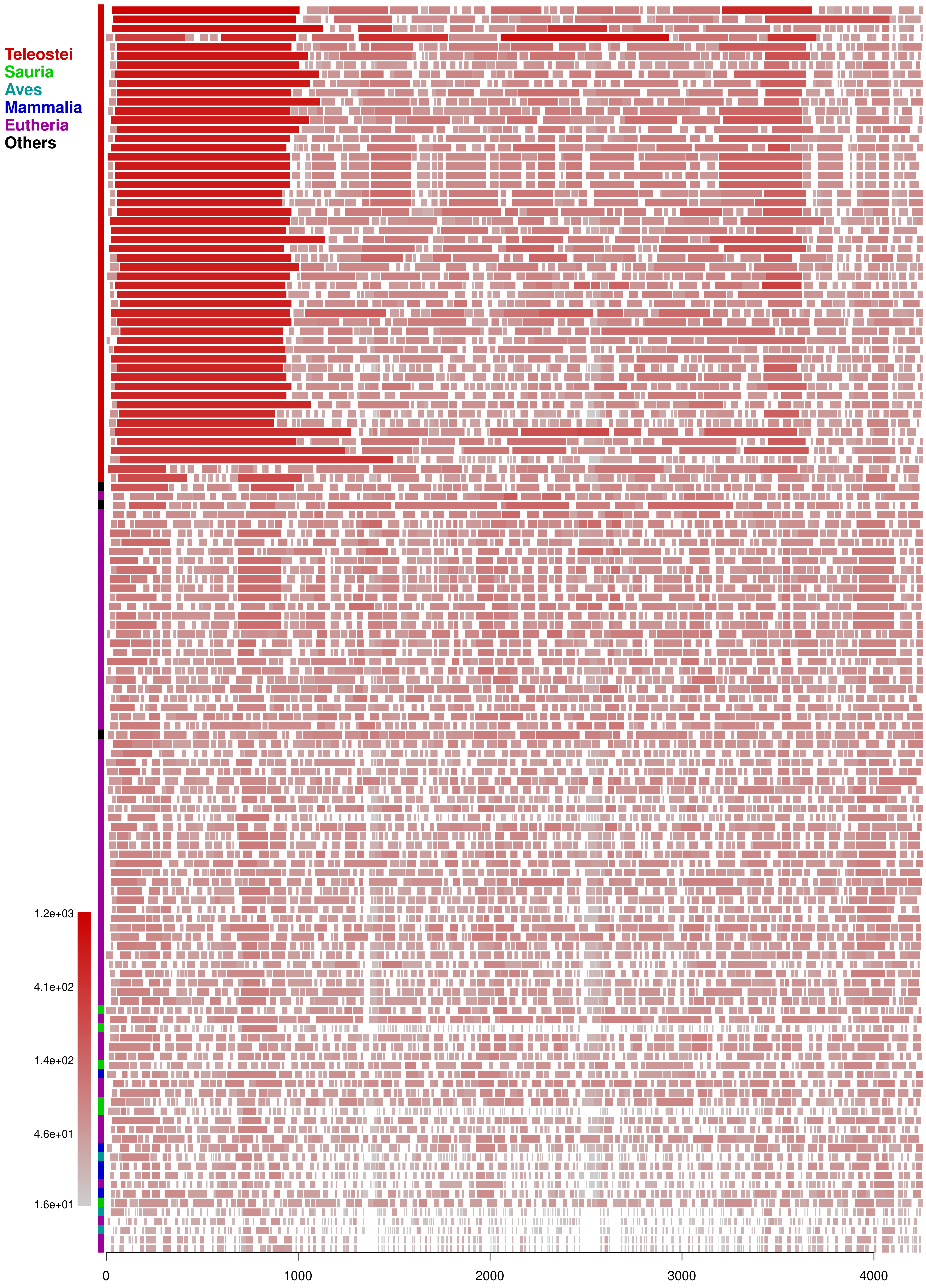

1 alignments above max size (1.0e+08)

ENSDARG00000079570 : ENSDART00000109124 intron no: 2 length: 18923

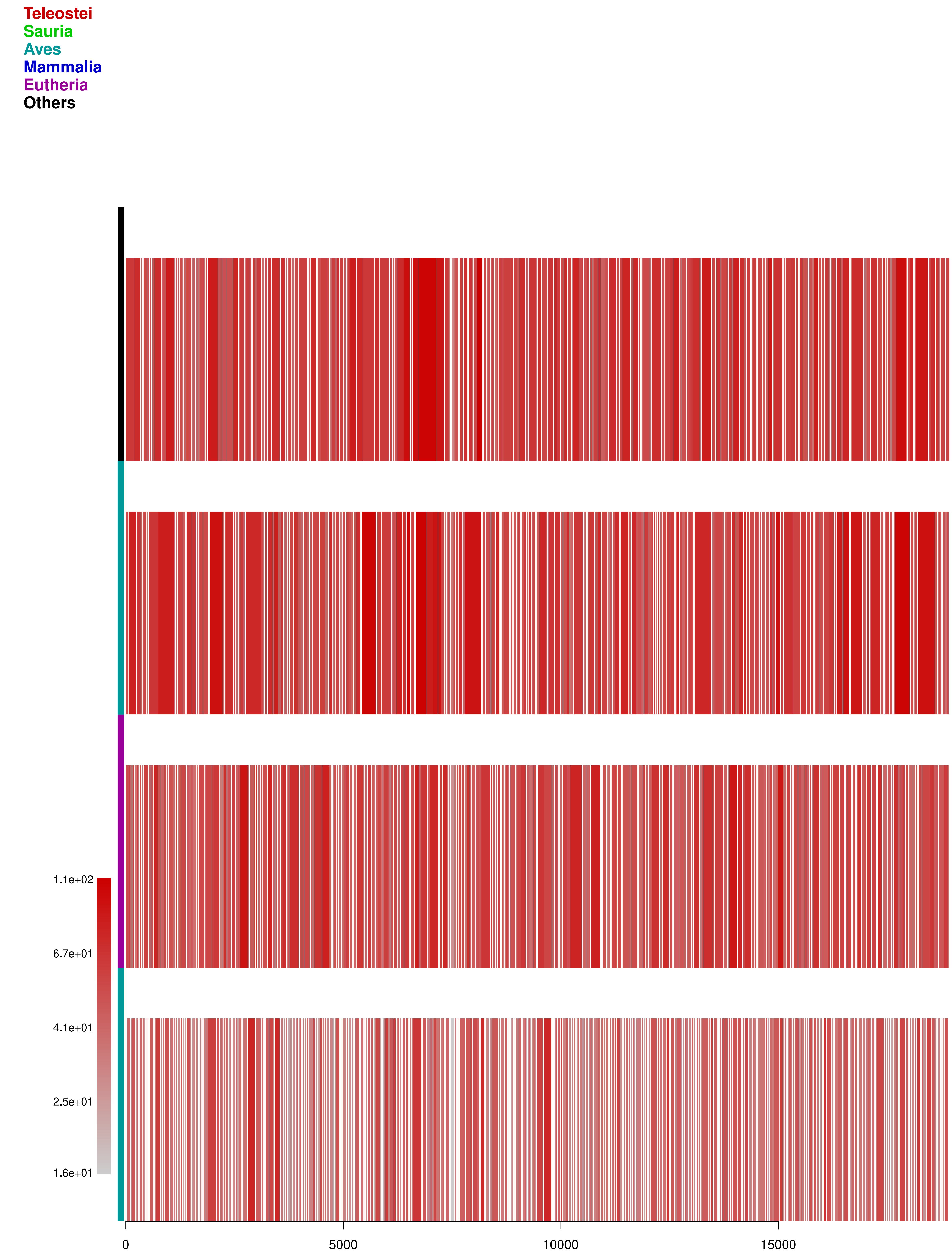

161 alignments above max size (1.0e+08)

ENSDARG00000075625 : ENSDART00000115085 intron no: 3 length: 9027

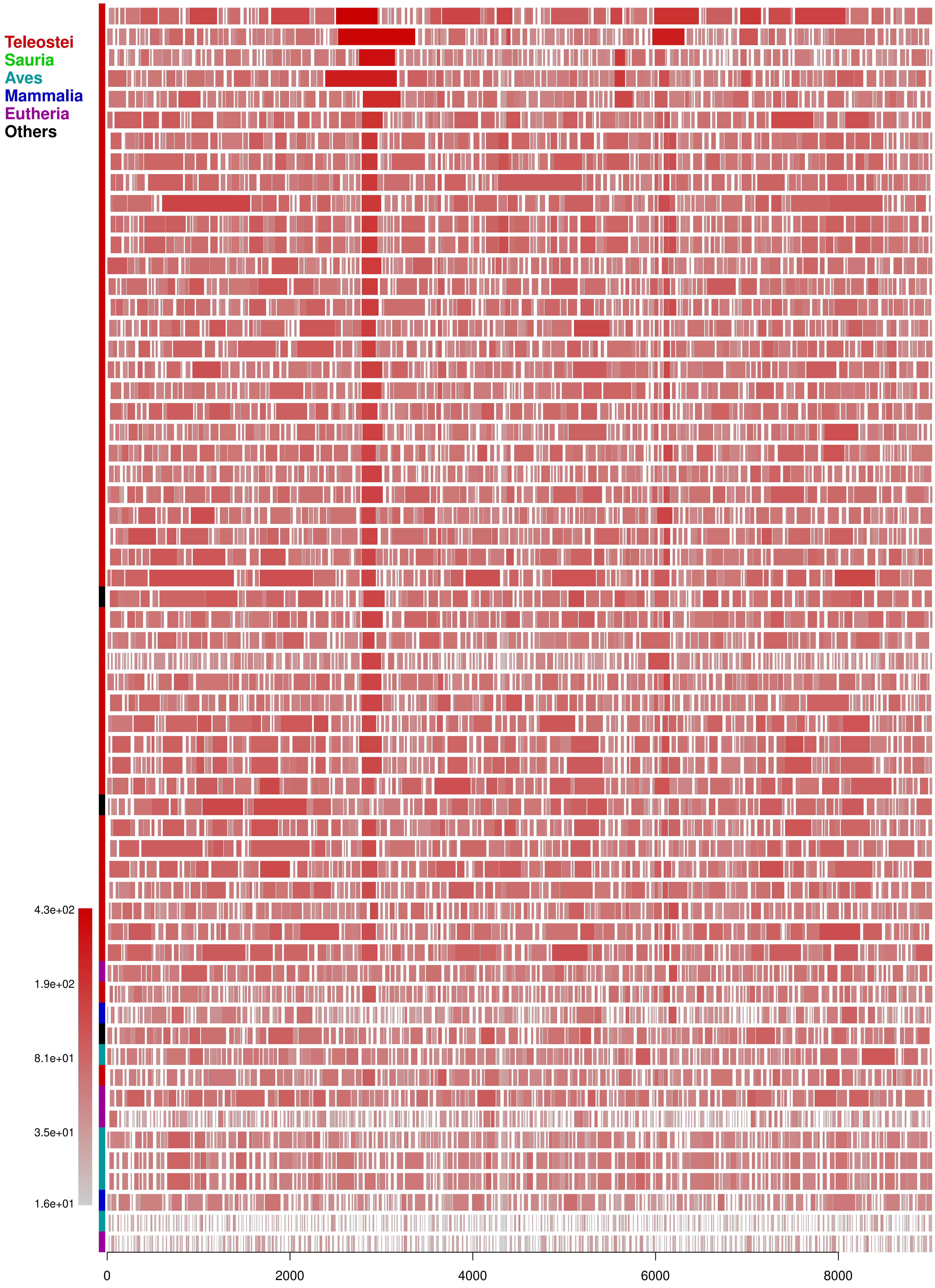

89 alignments above max size (1.0e+08)

ENSDARG00000061590 : ENSDART00000087964 intron no: 1 length: 57337

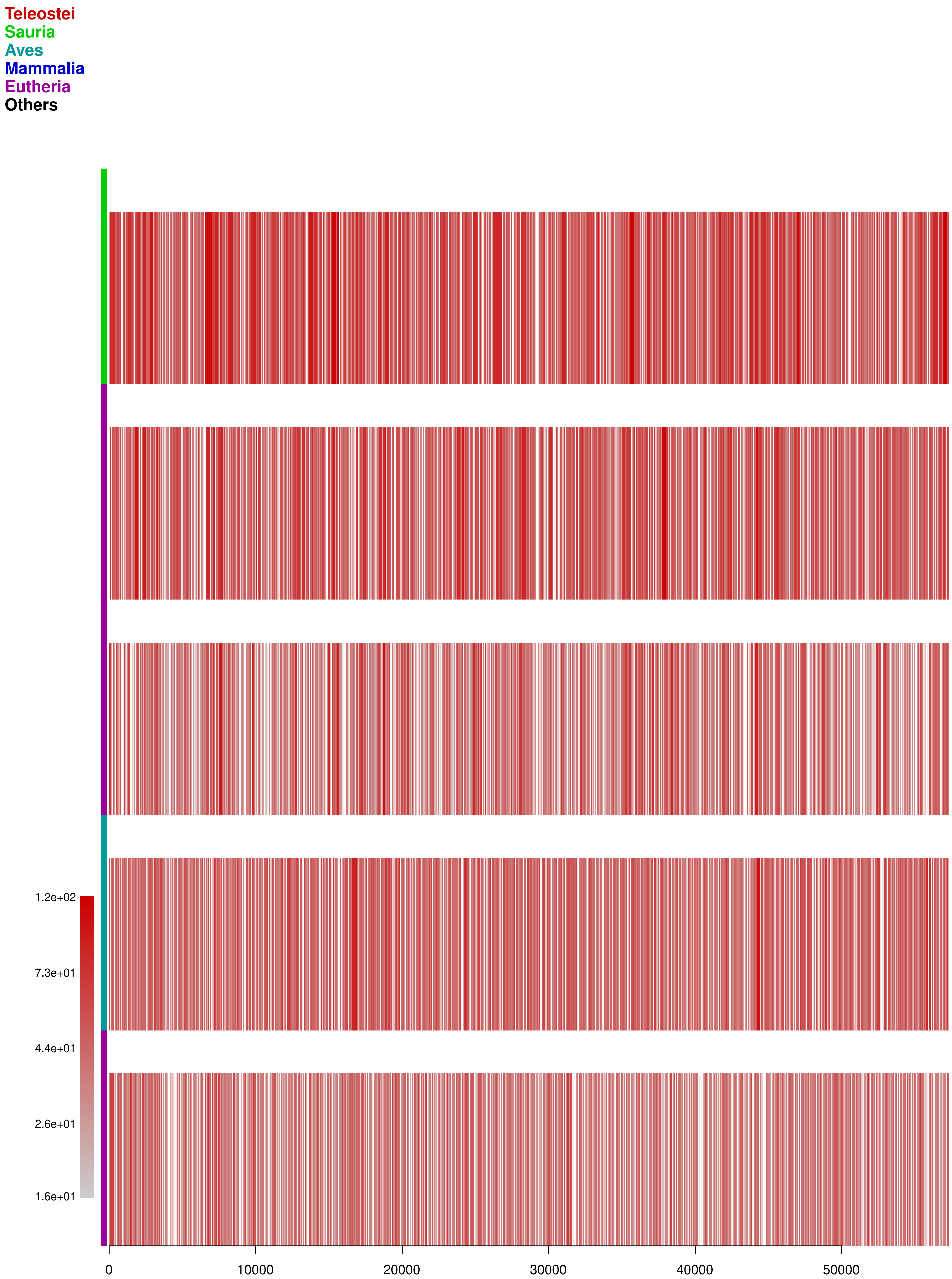

137 alignments above max size (1.0e+08)

ENSDARG00000100013 : ENSDART00000167562 intron no: 26 length: 3586

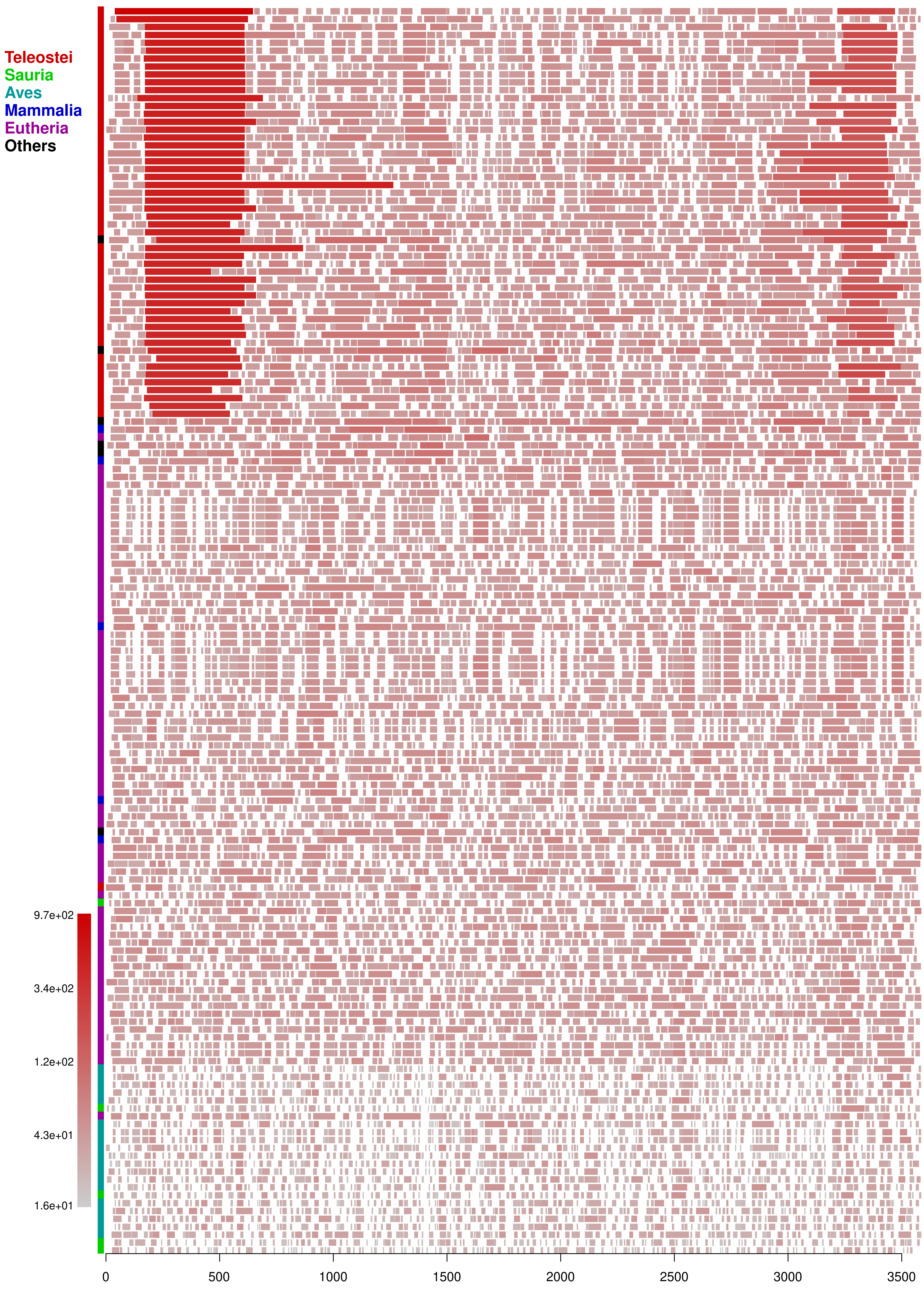

0 alignments above max size (1.0e+08)

ENSDARG00000042904 : ENSDART00000062982 intron no: 2 length: 39322

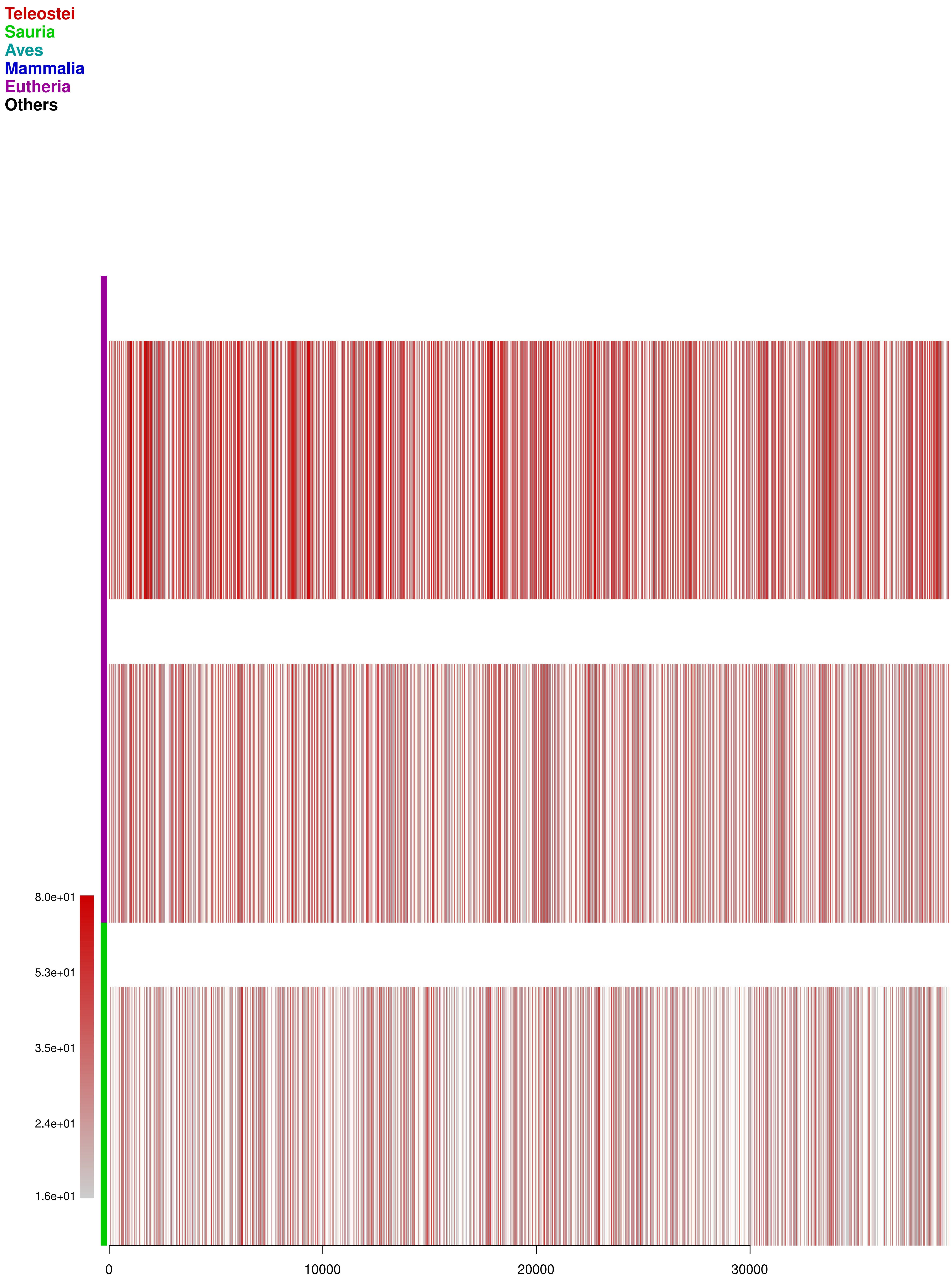

127 alignments above max size (1.0e+08)

ENSDARG00000076060 : ENSDART00000147443 intron no: 17 length: 131384

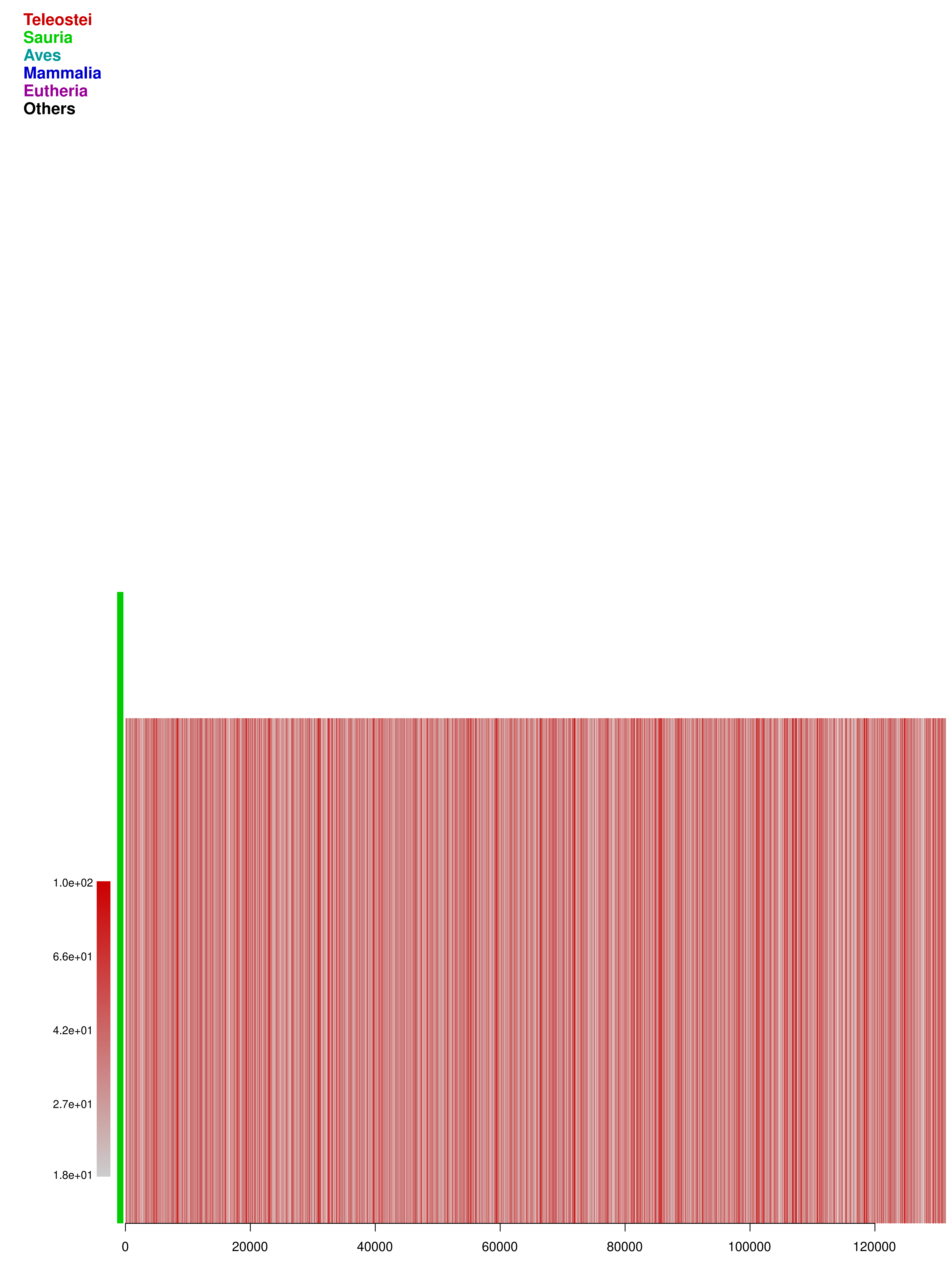

145 alignments above max size (1.0e+08)

ENSDARG00000078675 : ENSDART00000042134 intron no: 14 length: 15099

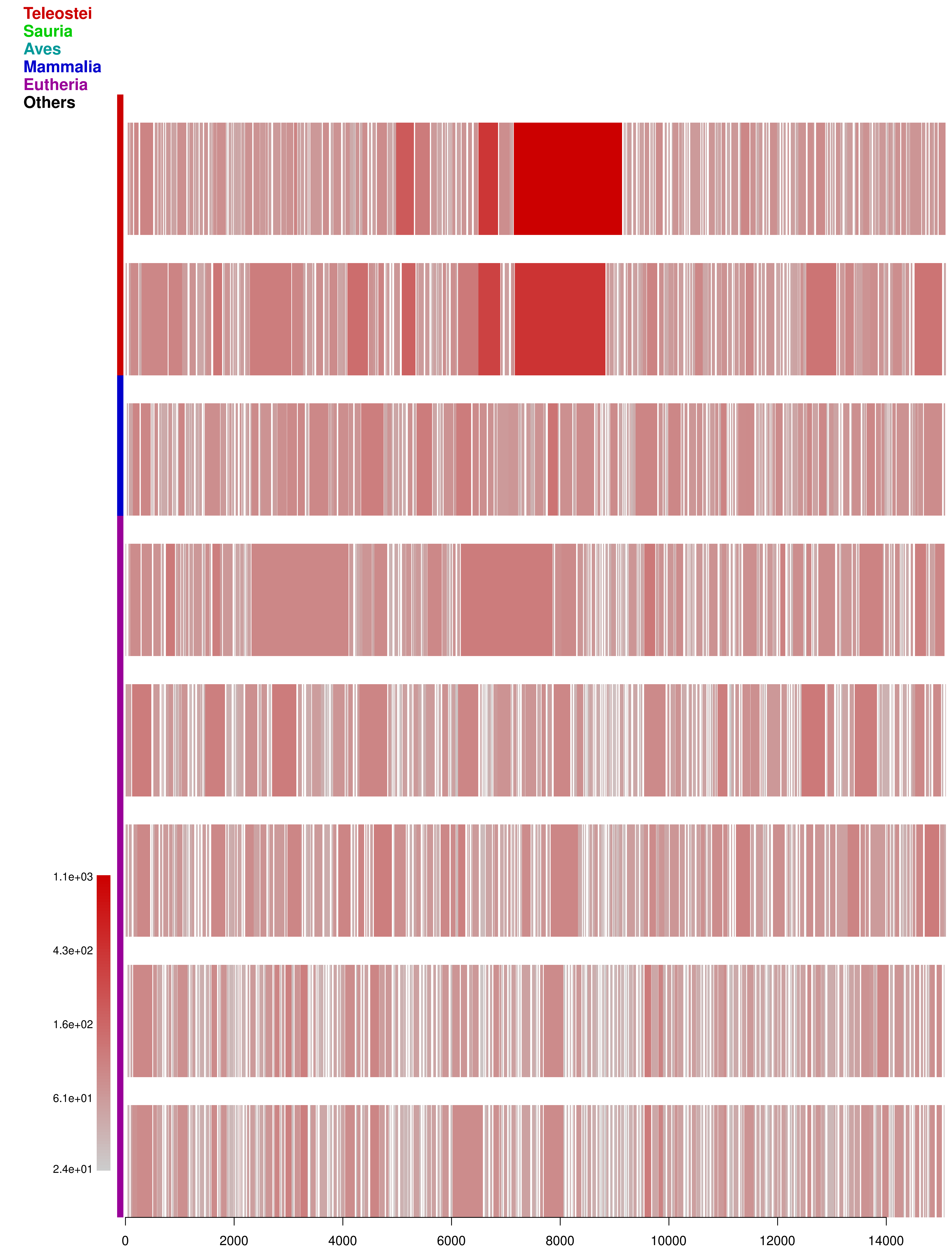

159 alignments above max size (1.0e+08)

ENSDARG00000076060 : ENSDART00000147443 intron no: 19 length: 31815

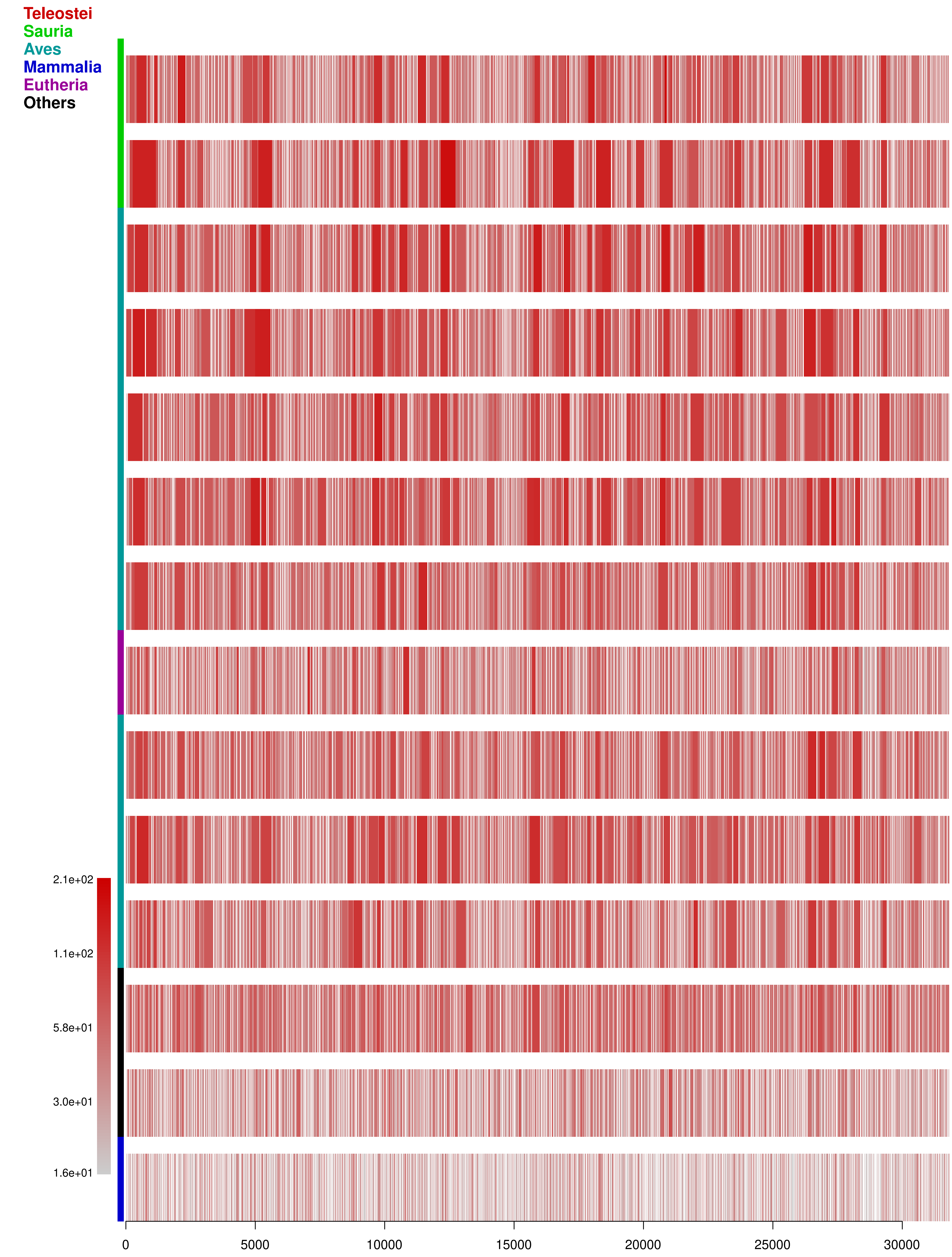

136 alignments above max size (1.0e+08)

ENSDARG00000076120 : ENSDART00000114288 intron no: 11 length: 2758

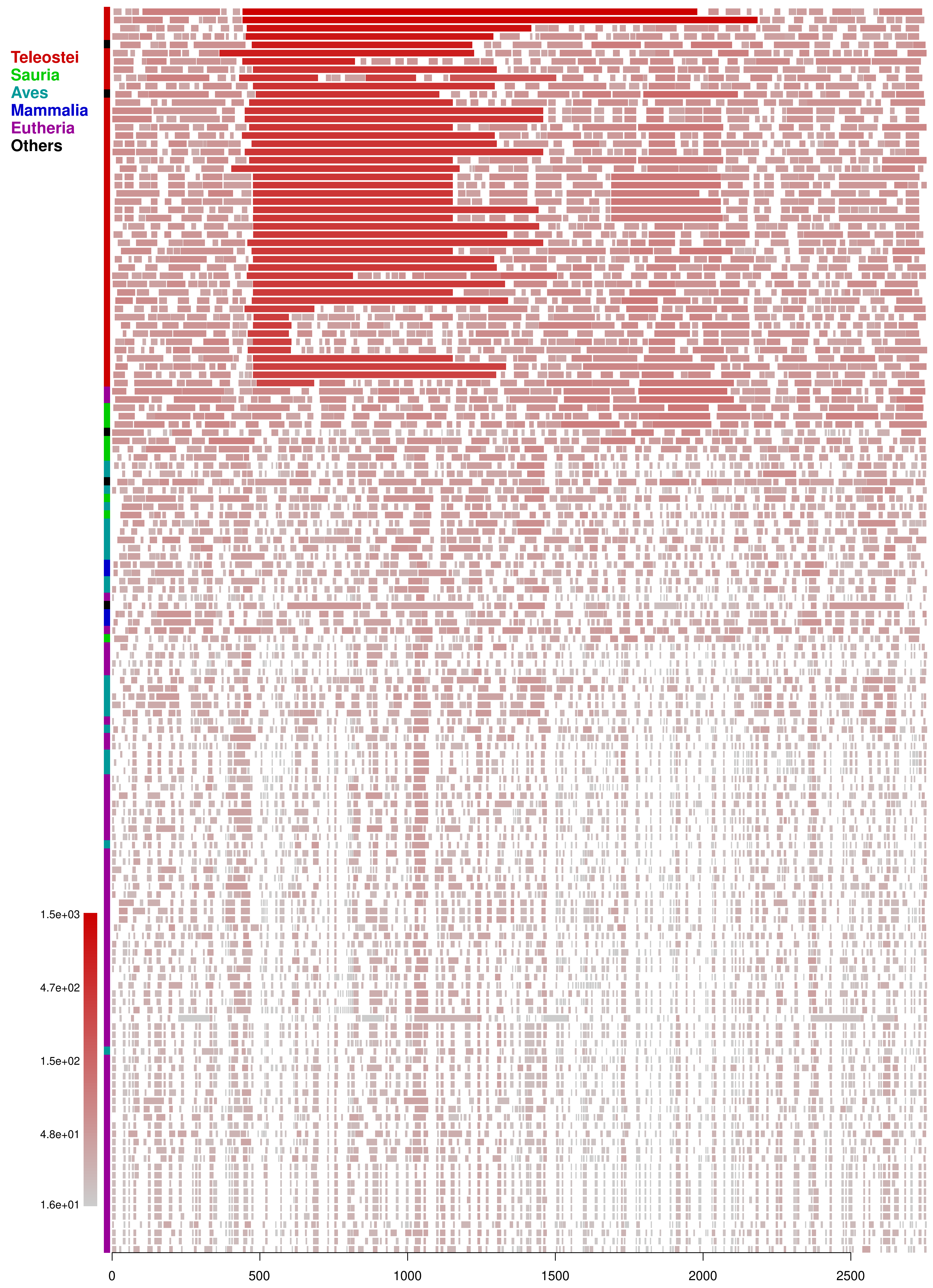

0 alignments above max size (1.0e+08)

ENSDARG00000018303 : ENSDART00000013033 intron no: 4 length: 12194

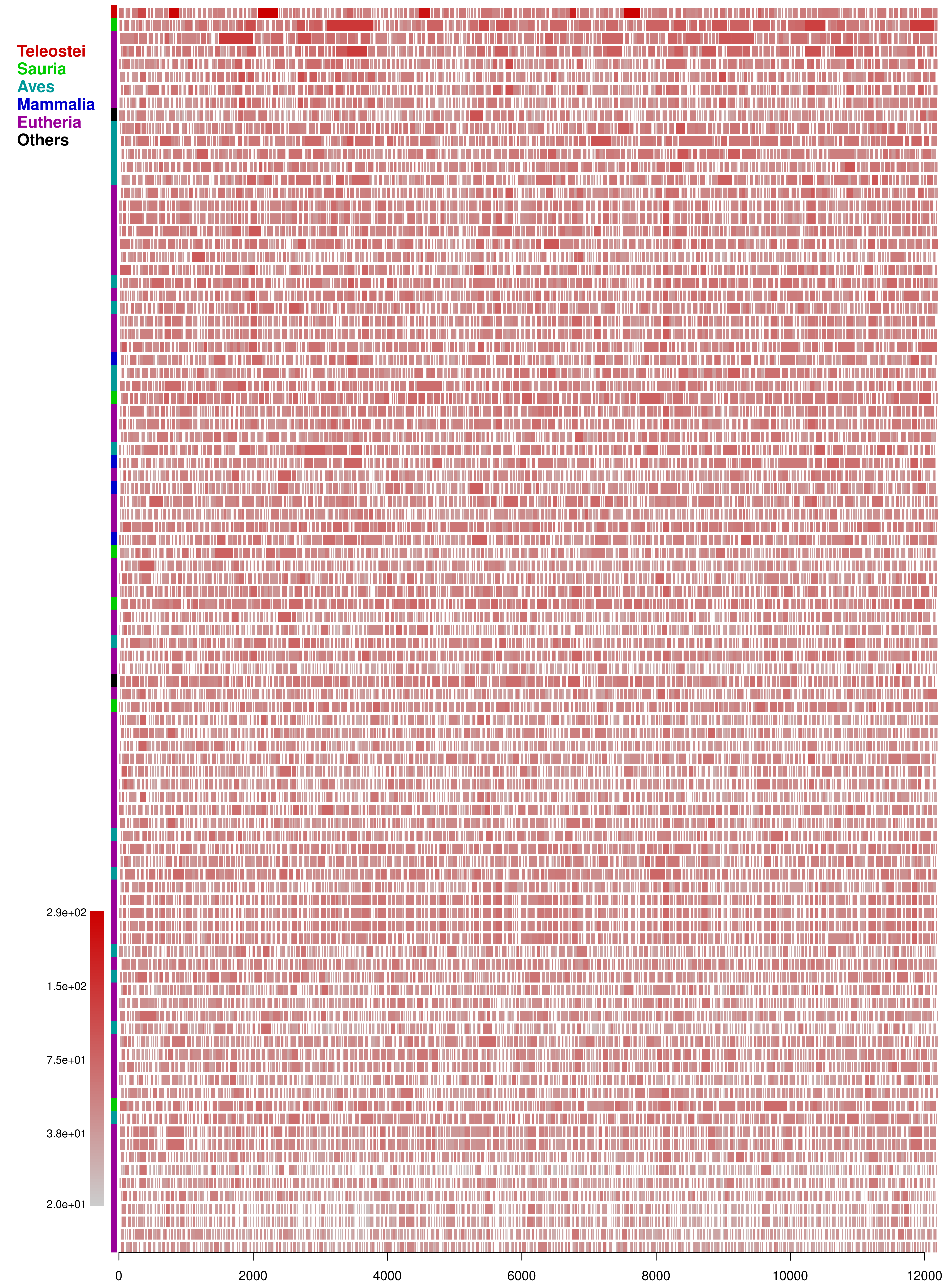

54 alignments above max size (1.0e+08)

ENSDARG00000055026 : ENSDART00000077304 intron no: 19 length: 3351

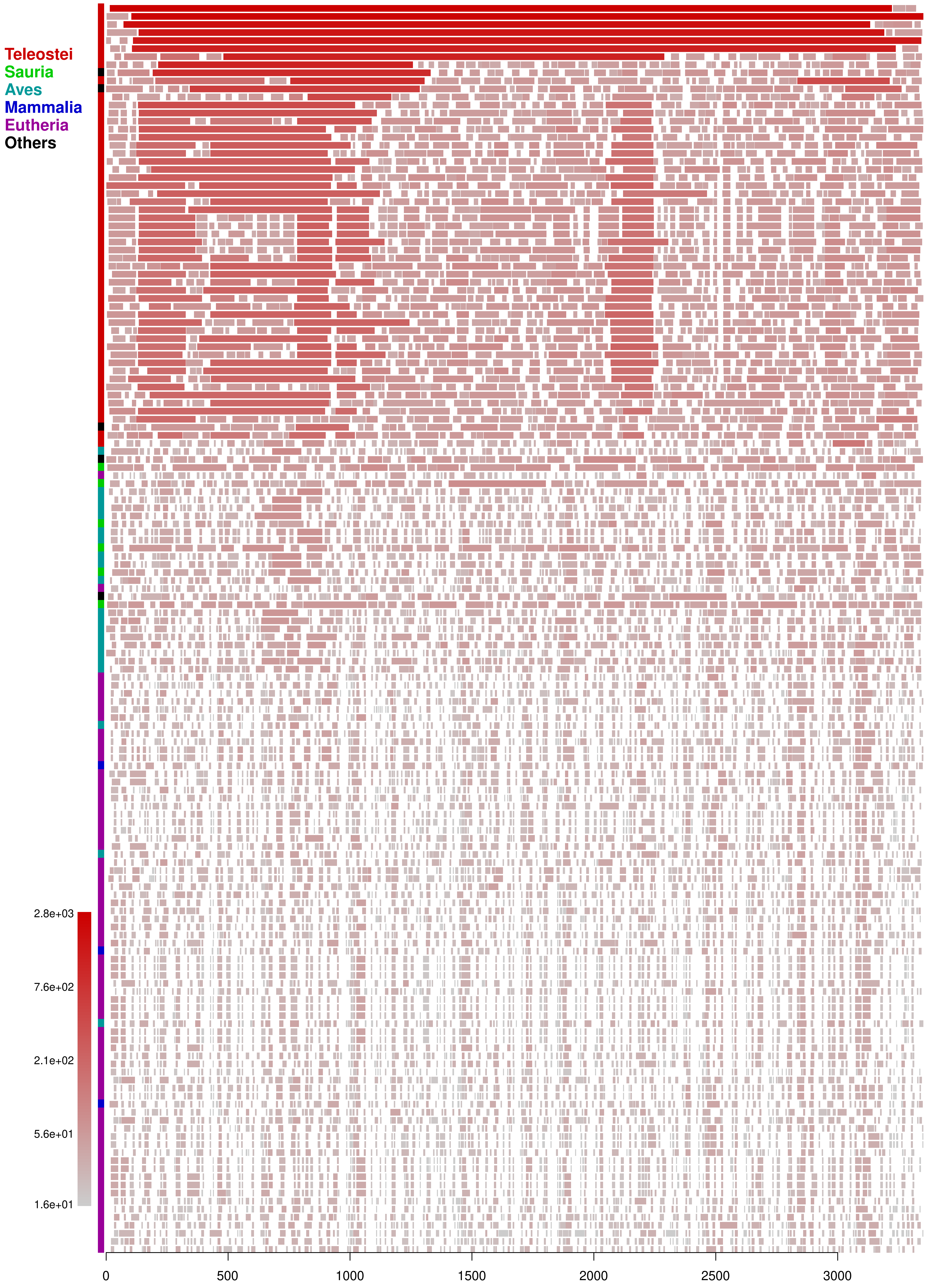

0 alignments above max size (1.0e+08)

ENSDARG00000022418 : ENSDART00000034004 intron no: 8 length: 17978

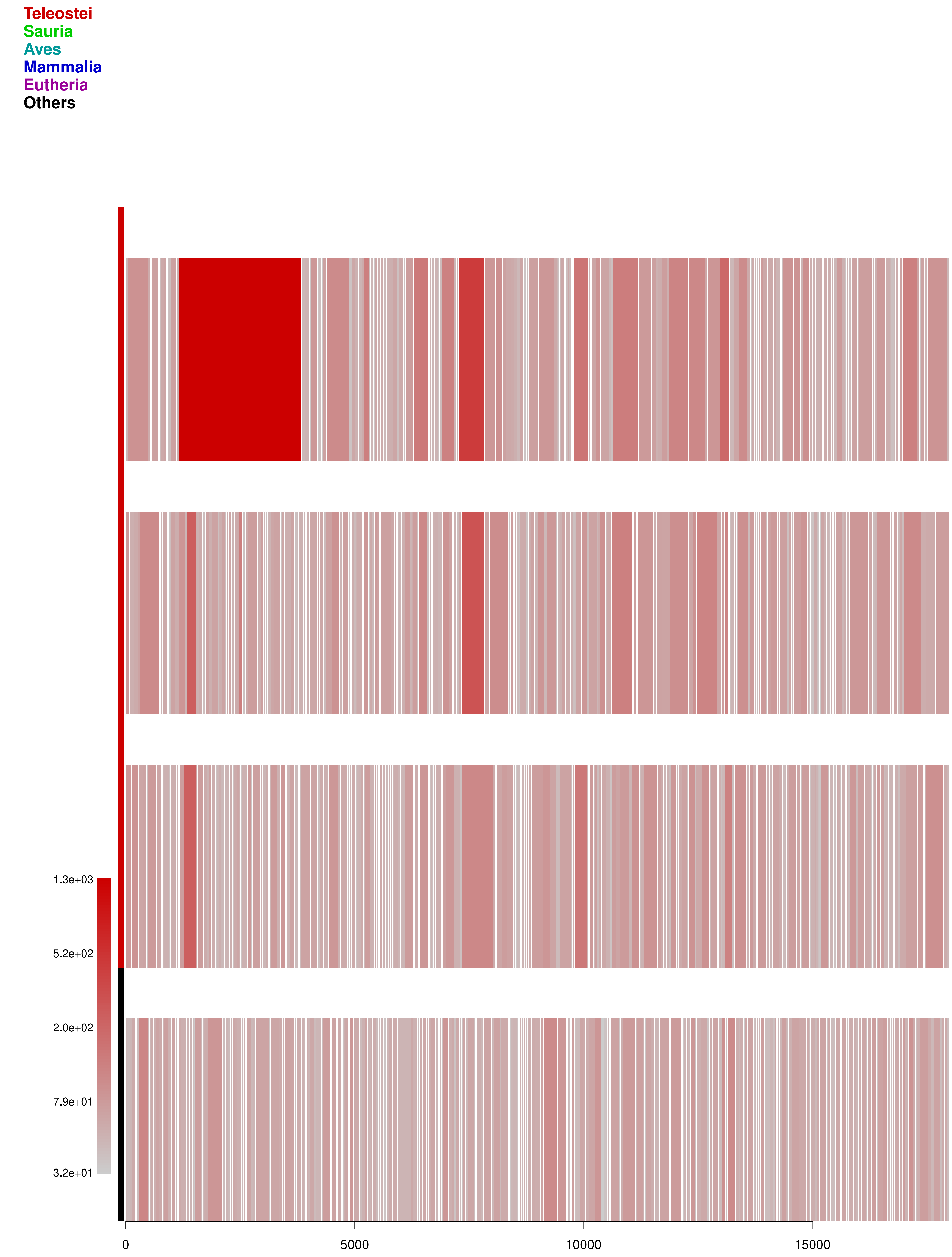

162 alignments above max size (1.0e+08)

ENSDARG00000074498 : ENSDART00000173682 intron no: 2 length: 17809

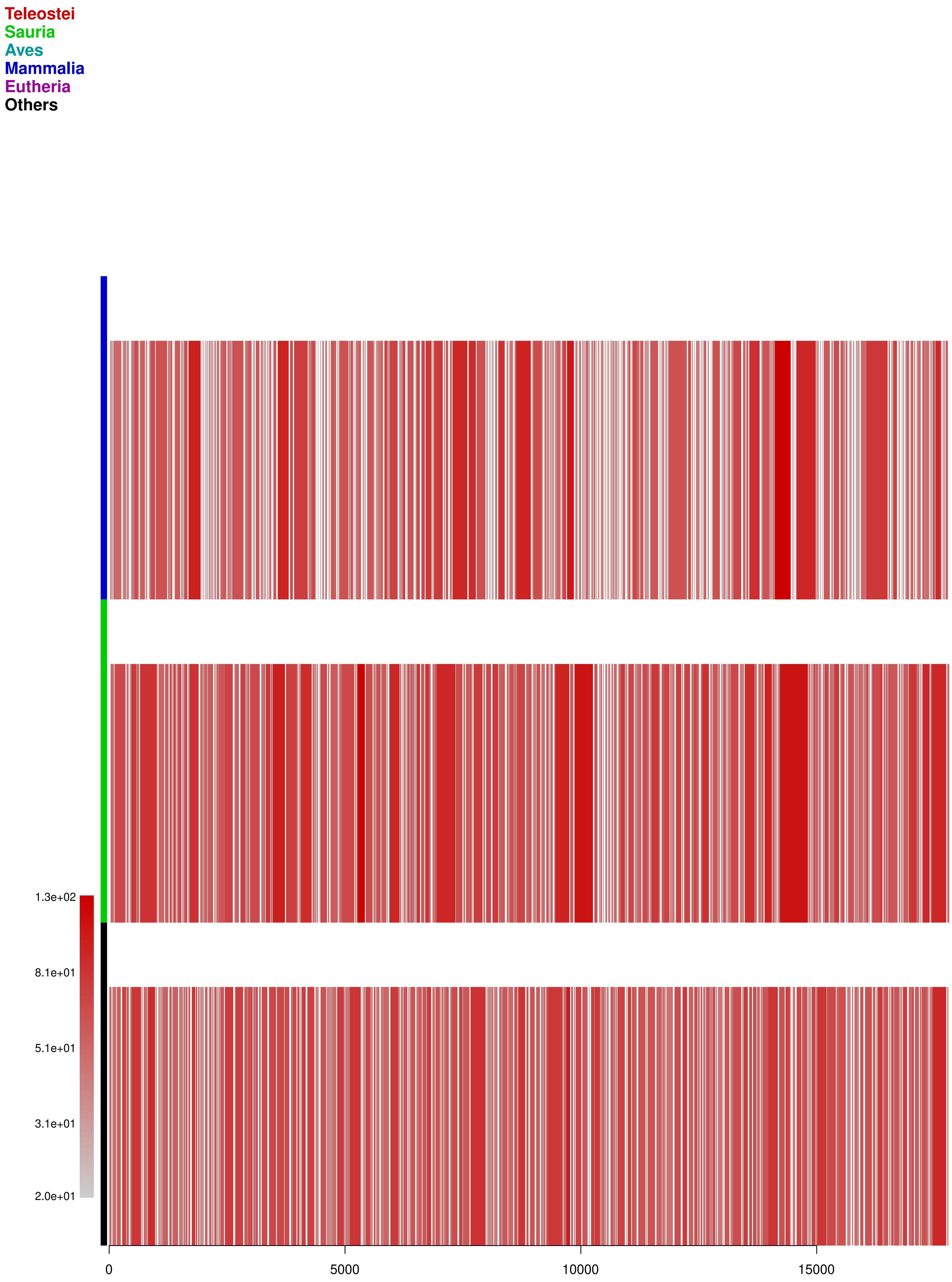

153 alignments above max size (1.0e+08)

ENSDARG00000031108 : ENSDART00000177989 intron no: 36 length: 40229

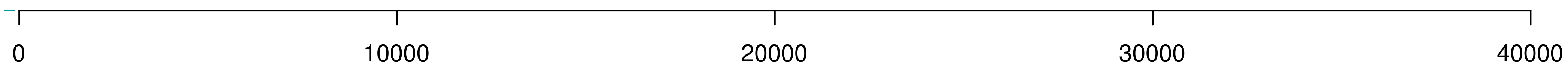

159 alignments above max size (1.0e+08)

ENSDARG00000007723 : ENSDART00000011399 intron no: 2 length: 20723

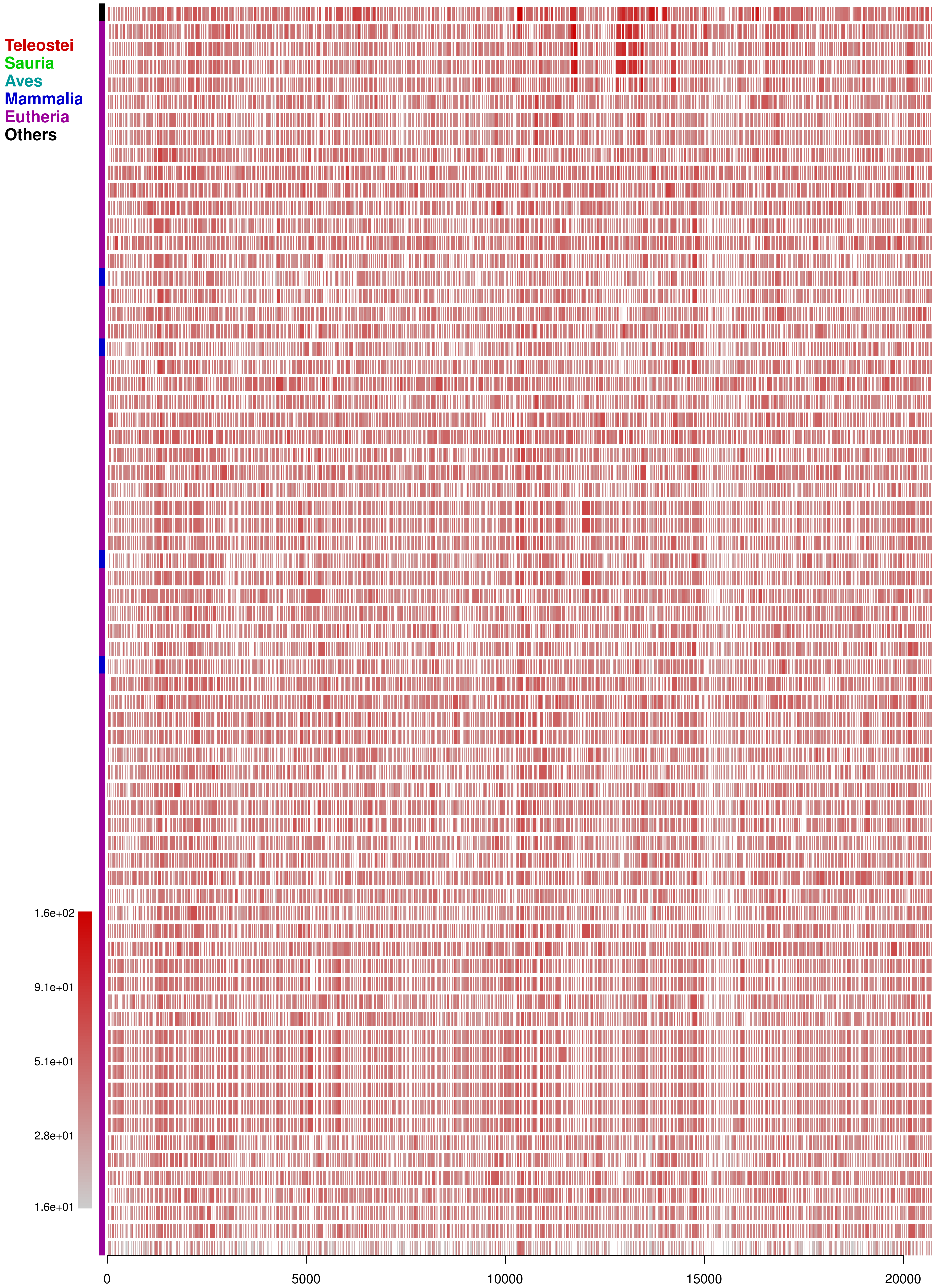

84 alignments above max size (1.0e+08)

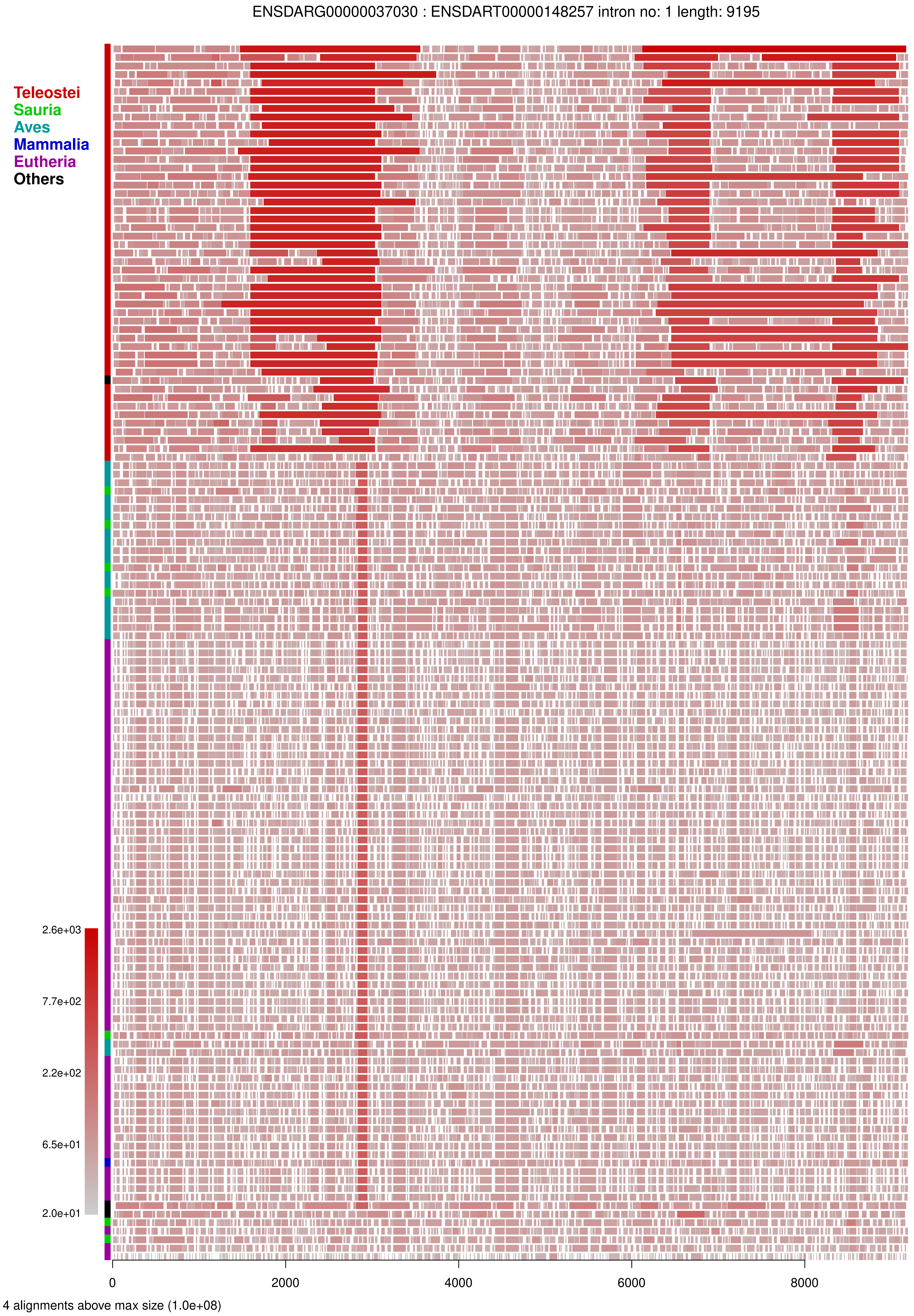
ENSDARG00000037030 : ENSDART00000148257 intron no: 1 length: 9195
Teleostei
Sauria
Aves
Mammalia
Eutheria
Others
2.6e+03
7.7e+02
2.2e+02
6.5e+01
2.0e+01
0
2000
4000
6000
8000
4 alignments above max size (1.0e+08)

ENSDARG00000105177 : ENSDART00000162317 intron no: 5 length: 2043

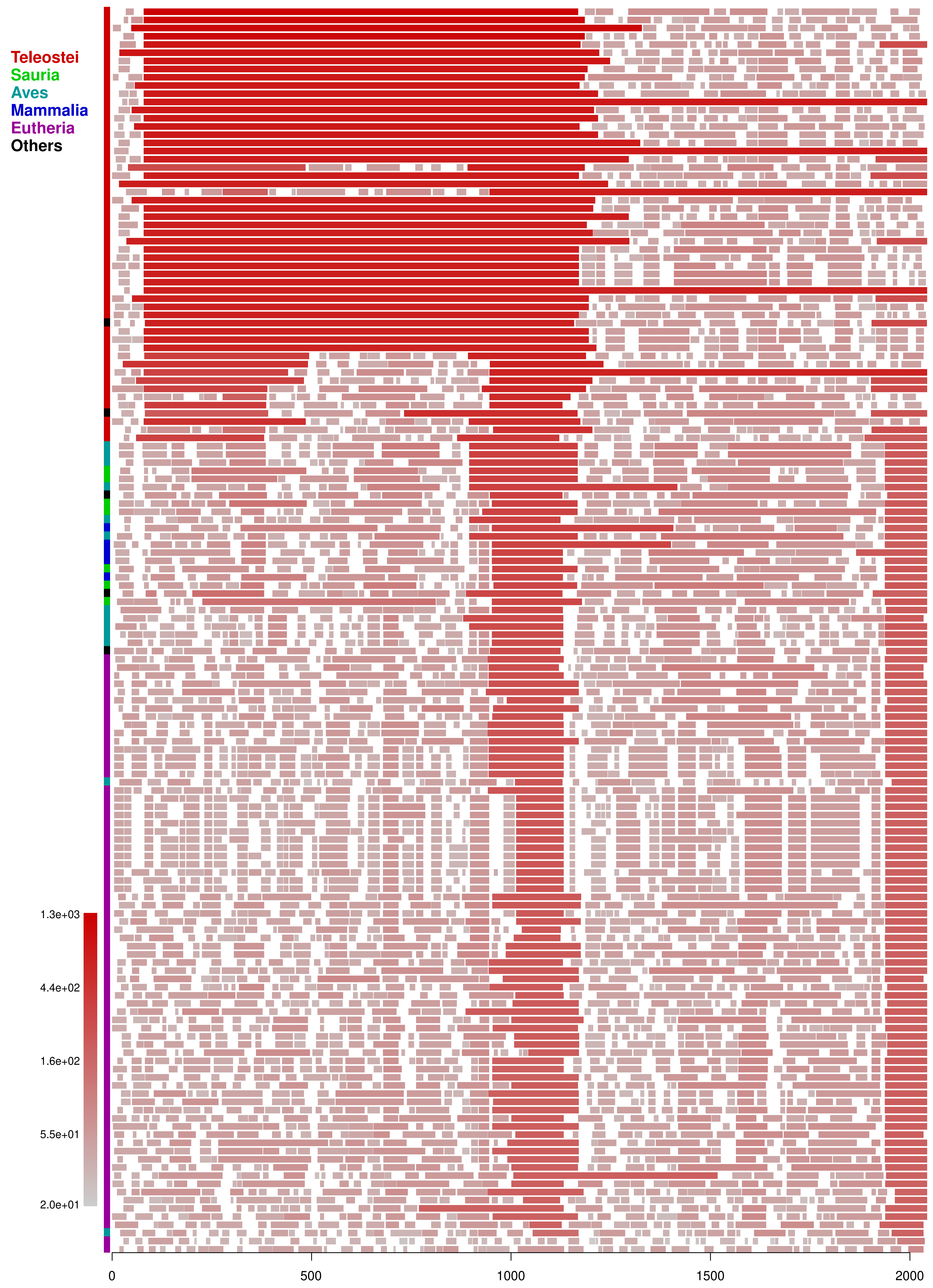

0 alignments above max size (1.0e+08)

ENSDARG00000014946 : ENSDART00000163855 intron no: 1 length: 3768

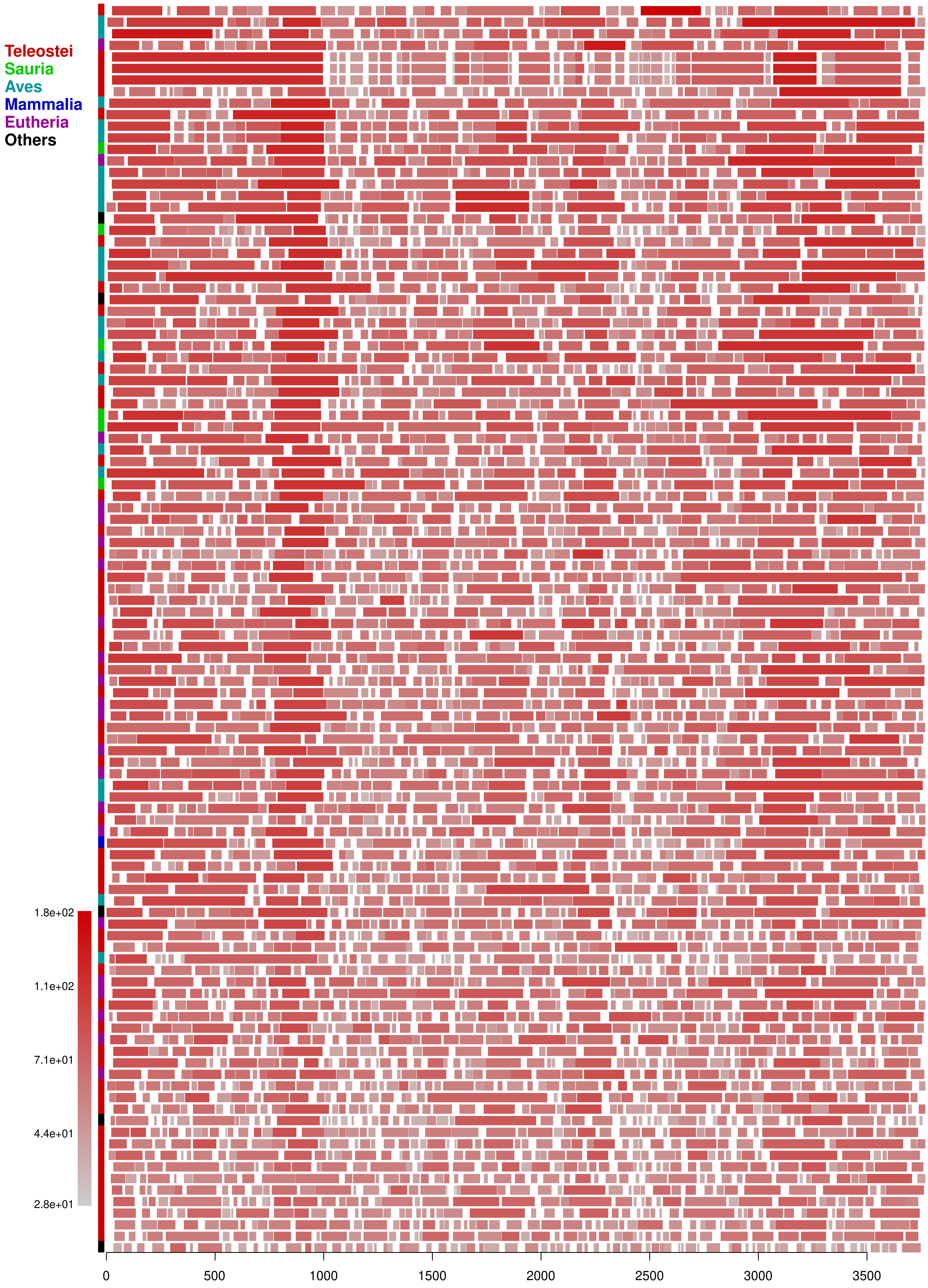

58 alignments above max size (1.0e+08)
