# Supplementary material for: Intron size minimisation in teleosts
Source: BMC Genomics. 2022 Sep 1;23:628. doi: 10.1186/s12864-022-08760-w (PMC9438311; doi:10.1186/s12864-022-08760-w)
Supplement: Supplementary file 5 — Additional file 5 Local alignments of D. rerio intron sequences to vertebrate orthologues for a set of introns with variable teleost lengths; introns were long (>1024 bp) in D. rerio but had a median teleost length less than 256 bp. Alignments were performed recursively to identify all non-overlapping alignments to the D. rerio sequence. [file 12864_2022_8760_MOESM5_ESM.pdf]

ENSDARG00000074695 : ENSDART00000109266 intron no: 16 length: 2629

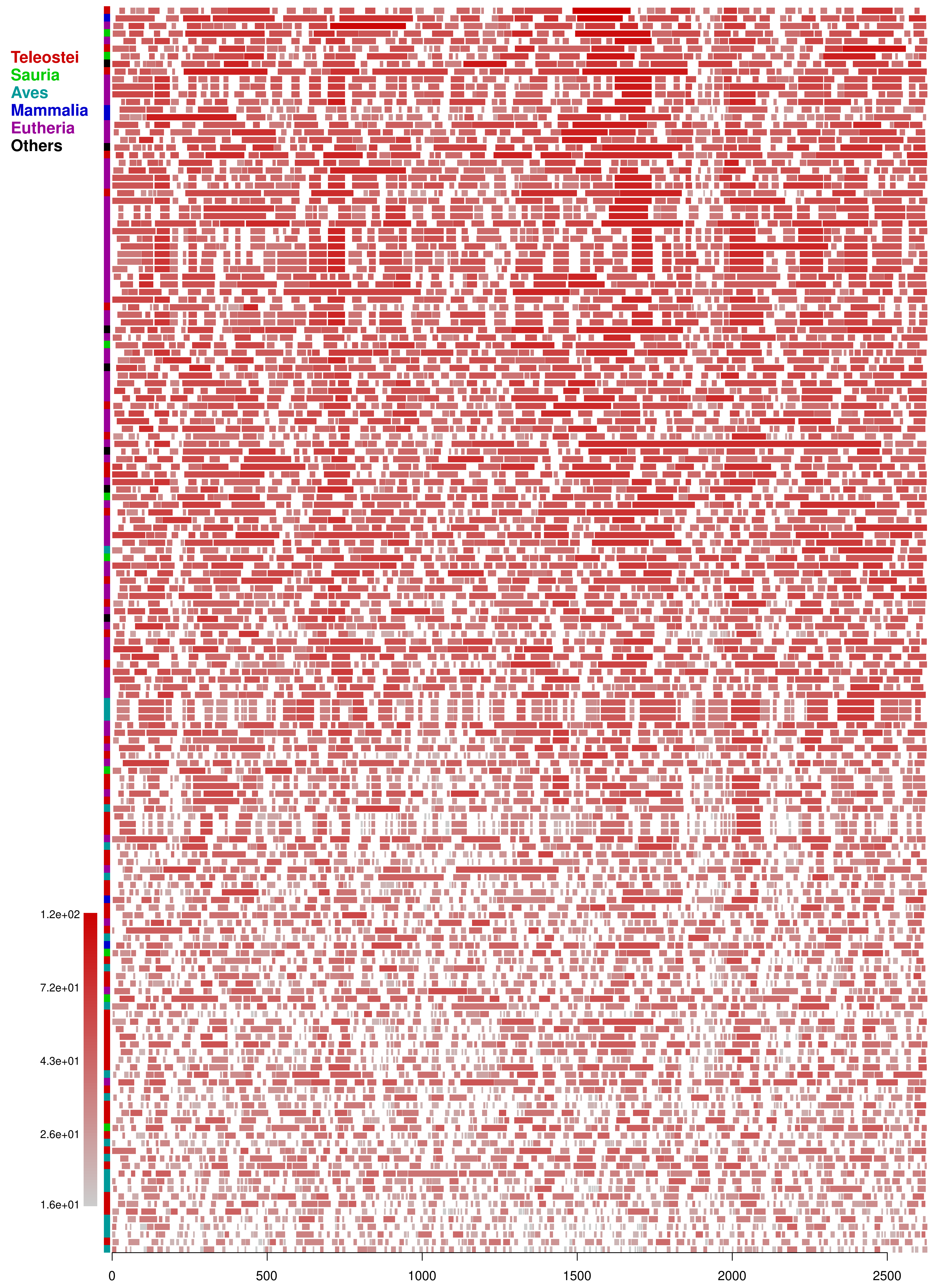

0 alignments above max size (1.0e+08)

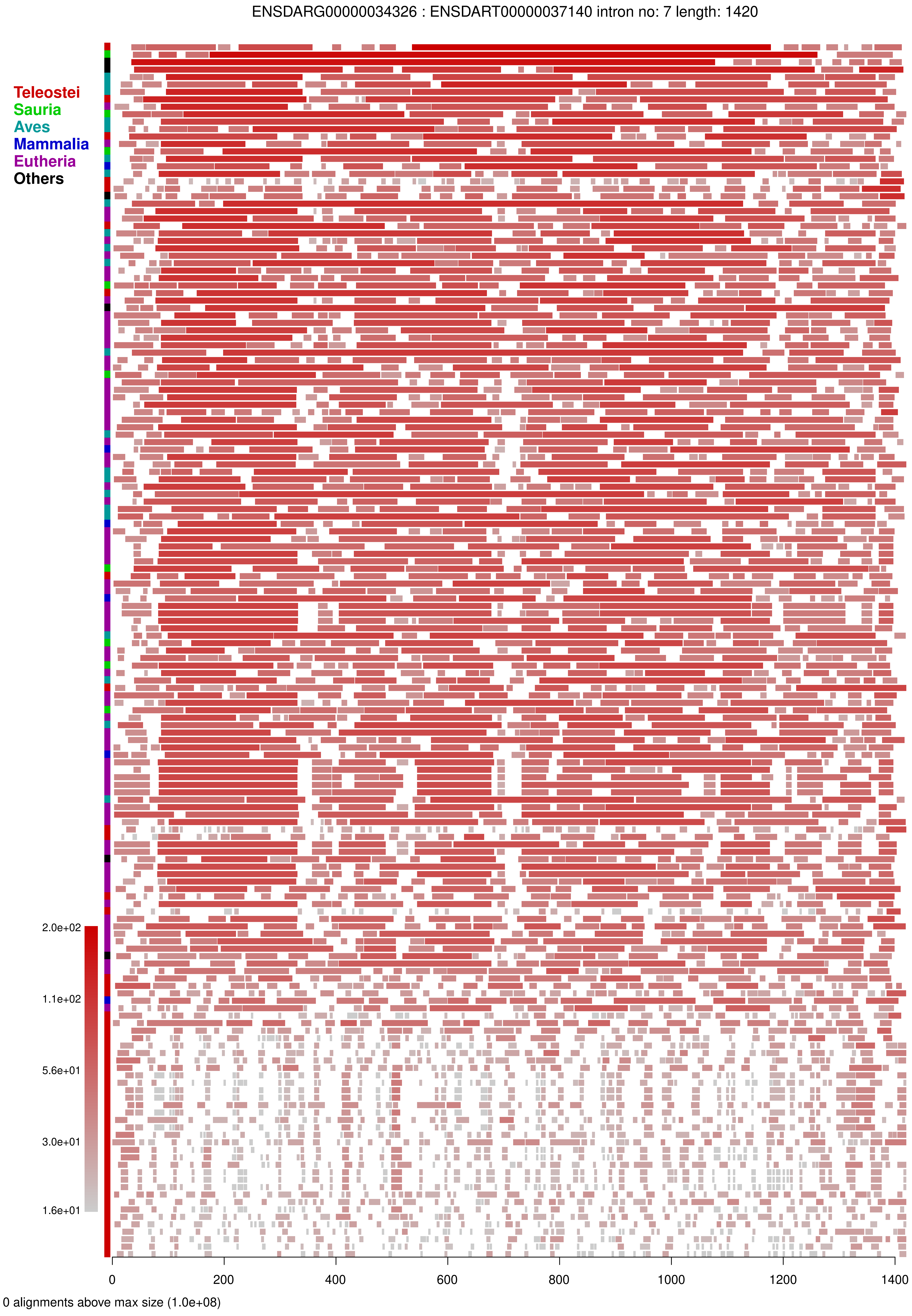

ENSDARG00000034326 : ENSDART00000037140 intron no: 7 length: 1420
Teleostei
Sauria
Aves
Mammalia
Eutheria
Others
2.0e+02
1.1e+02
5.6e+01
3.0e+01
1.6e+01
0
200
400
600
800
1000
1200
1400
0 alignments above max size (1.0e+08)

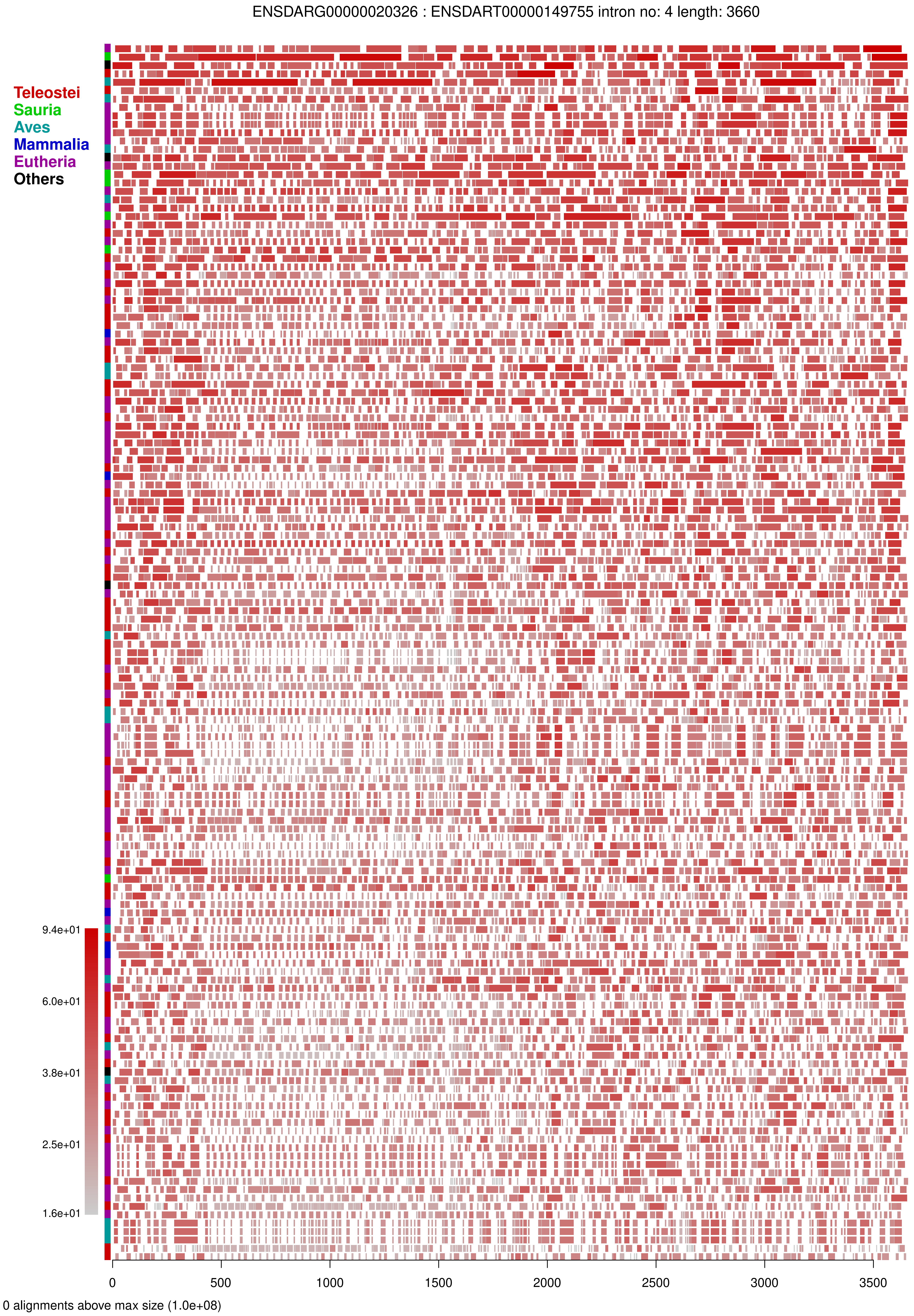
ENSDARG00000020326 : ENSDART00000149755 intron no: 4 length: 3660
Teleostei
Sauria
Aves
Mammalia
Eutheria
Others
9.4e+01
6.0e+01
3.8e+01
2.5e+01
1.6e+01
0
500
1000
1500
2000
2500
3000
3500
0 alignments above max size (1.0e+08)

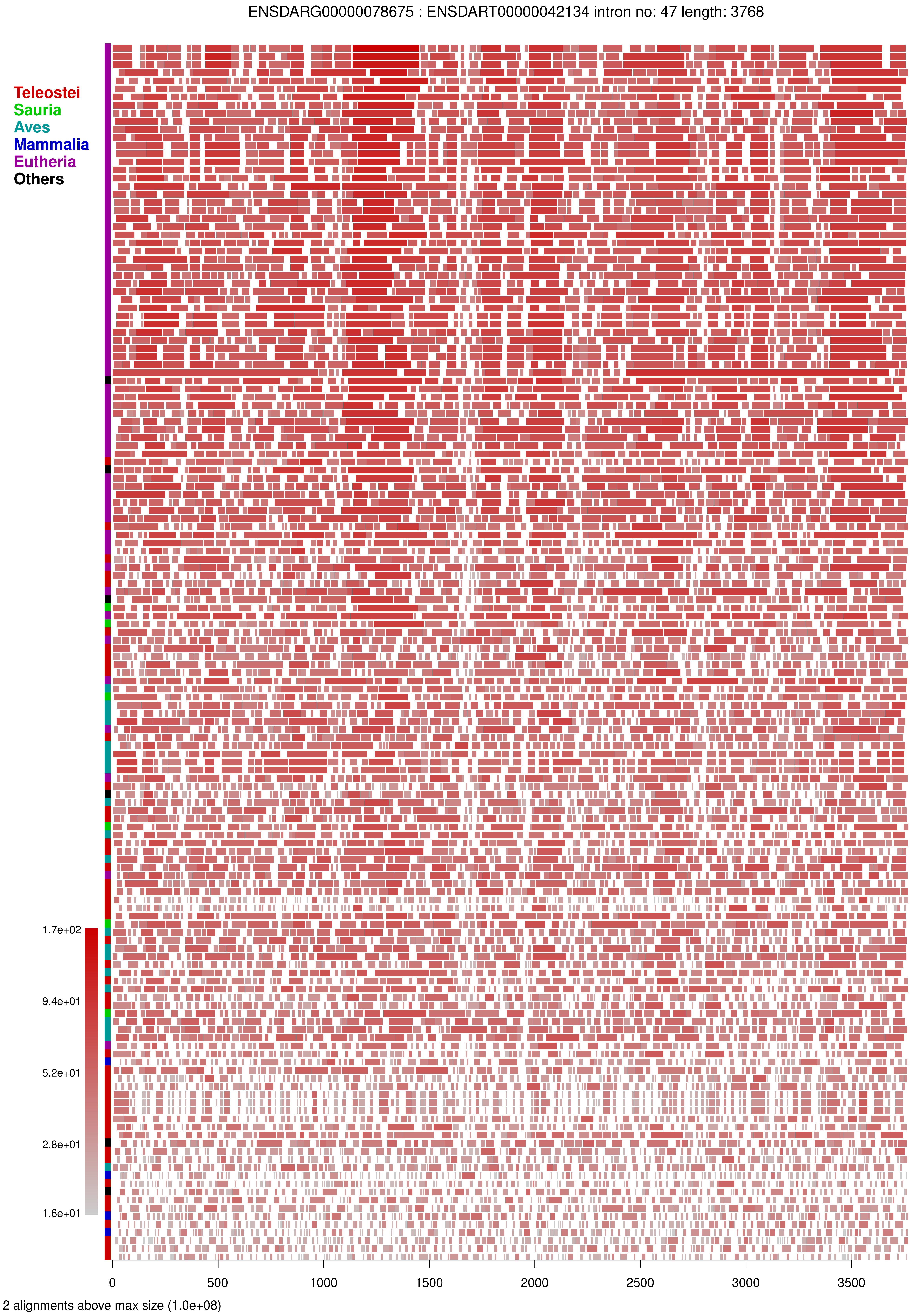

ENSDARG00000078675 : ENSDART00000042134 intron no: 47 length: 3768
Teleostei
Sauria
Aves
Mammalia
Eutheria
Others
1.7e+02
9.4e+01
5.2e+01
2.8e+01
1.6e+01
0
500
1000
1500
2000
2500
3000
3500
2 alignments above max size (1.0e+08)

ENSDARG00000006093 : ENSDART00000081129 intron no: 3 length: 1904

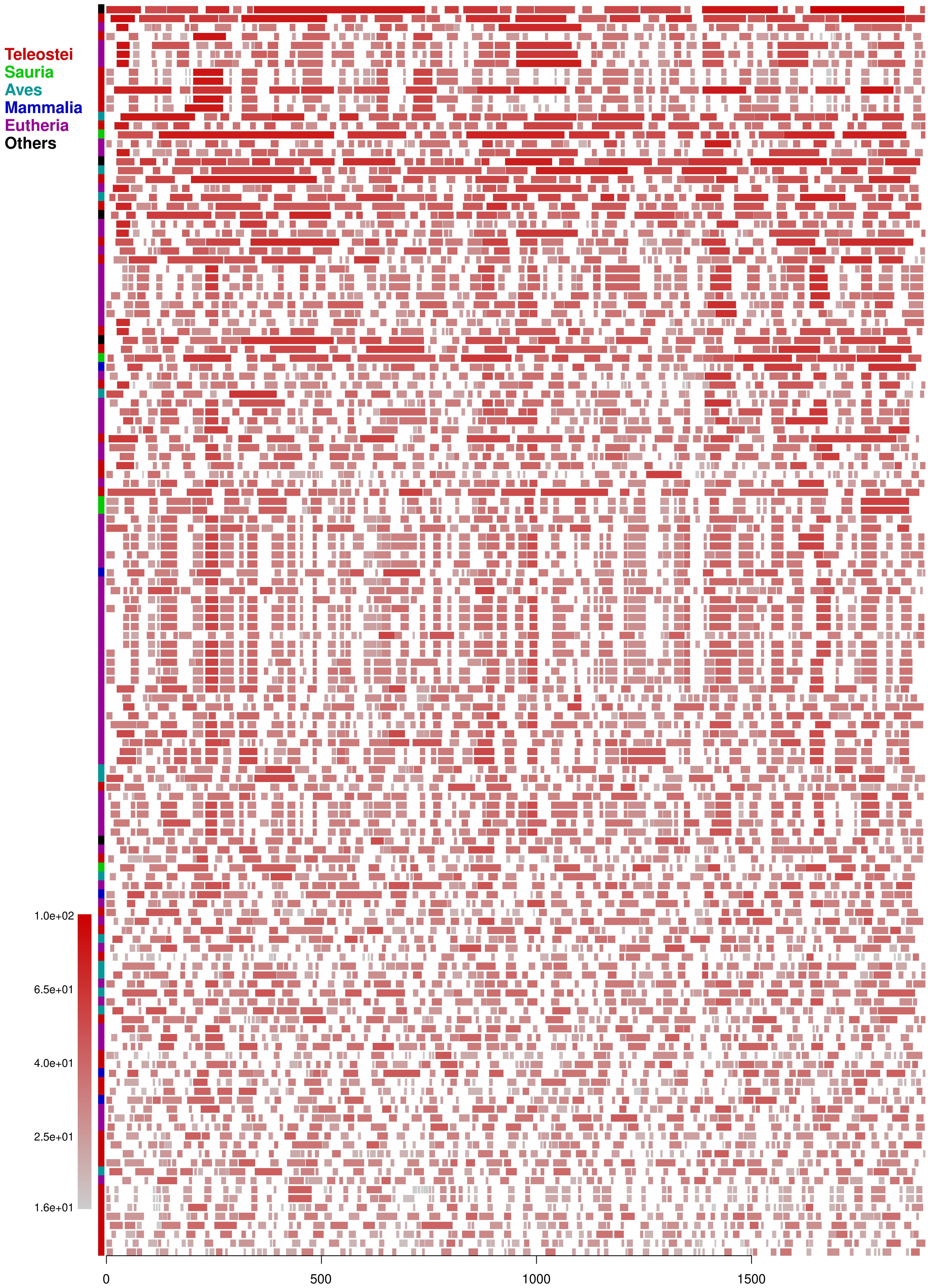

0 alignments above max size (1.0e+08)

ENSDARG00000020201 : ENSDART00000157155 intron no: 2 length: 3133

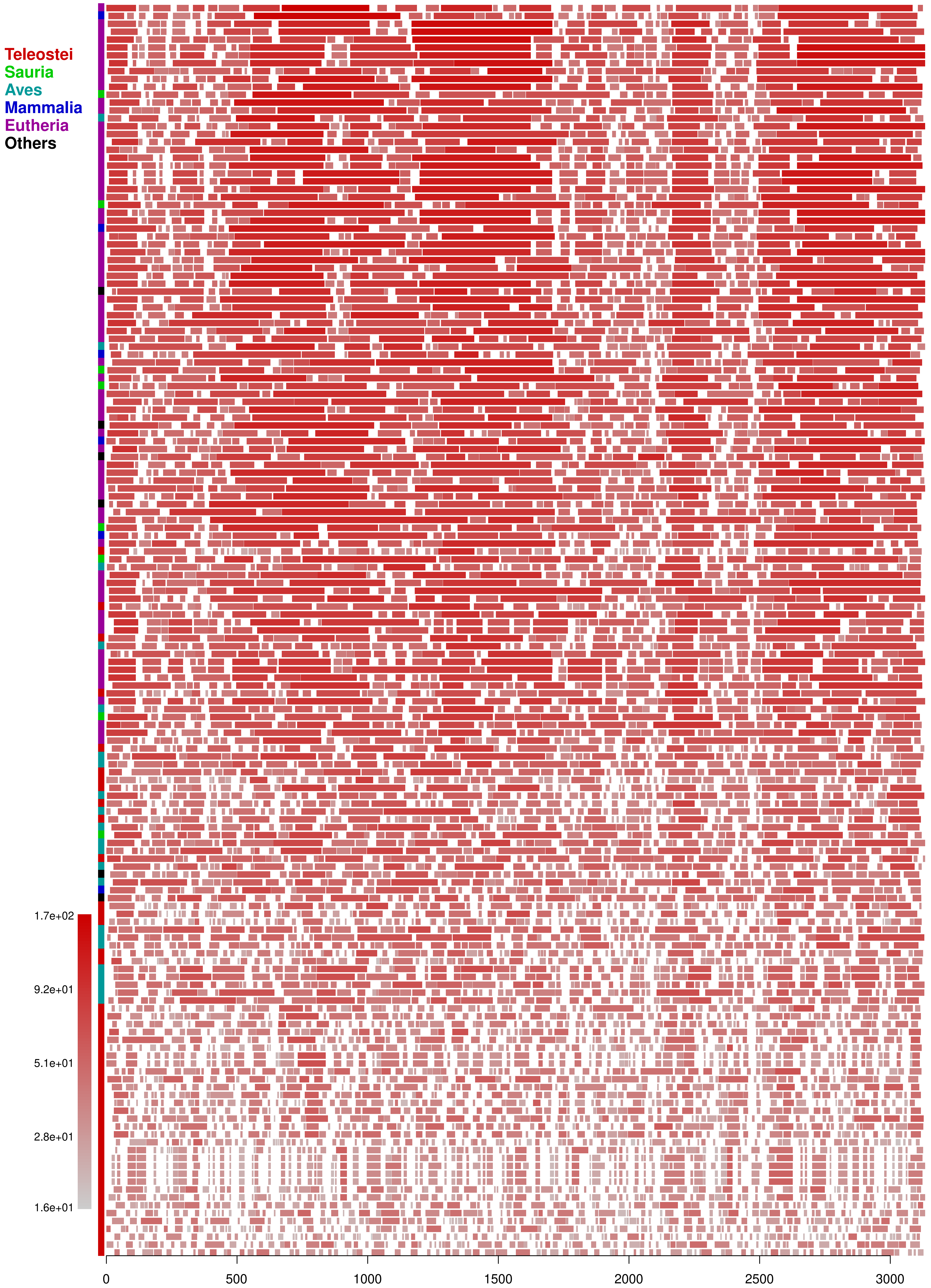

1 alignments above max size (1.0e+08)

ENSDARG00000079848 : ENSDART00000109535 intron no: 10 length: 1105

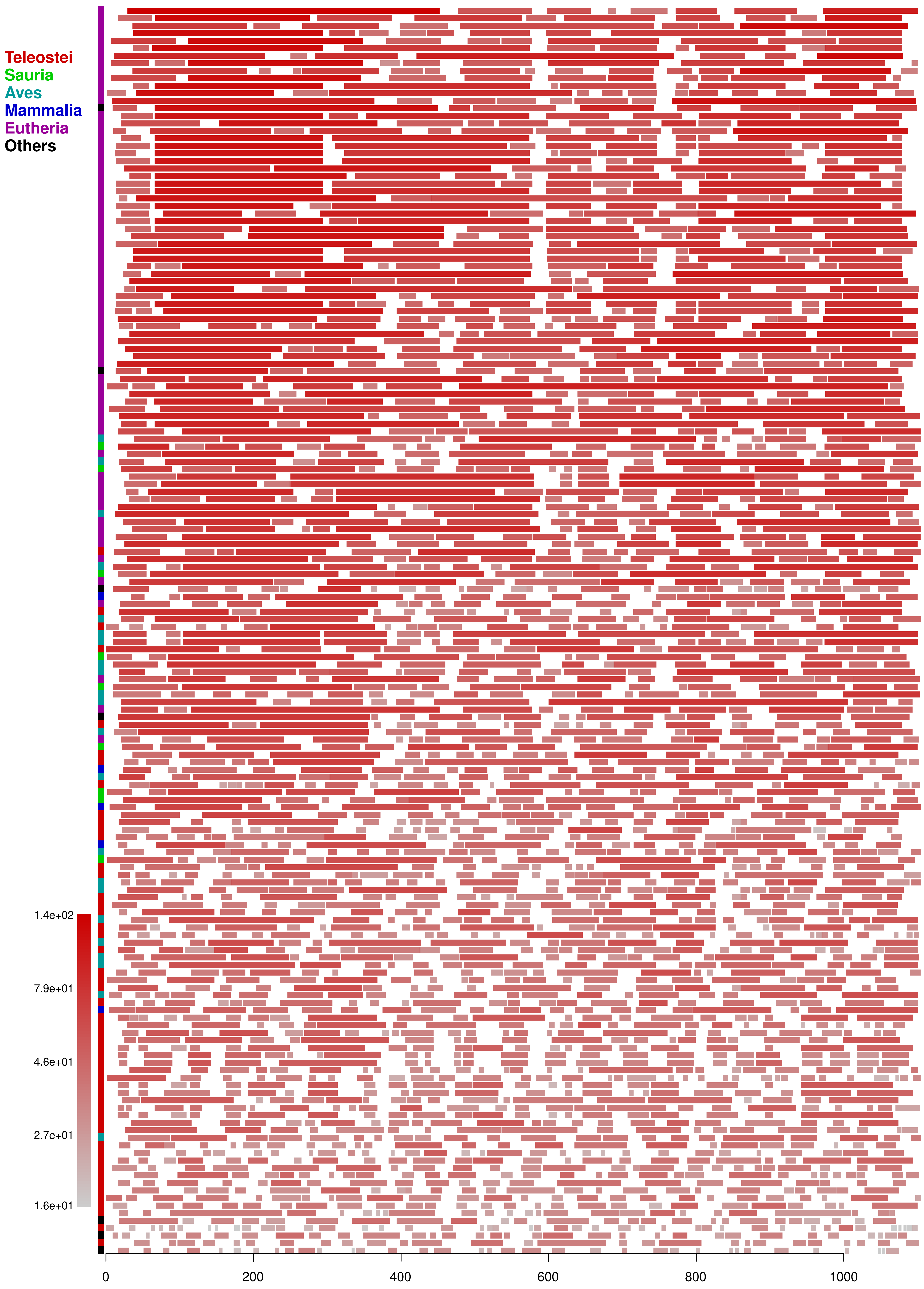

0 alignments above max size (1.0e+08)

ENSDARG00000100420 : ENSDART00000170637 intron no: 16 length: 2853

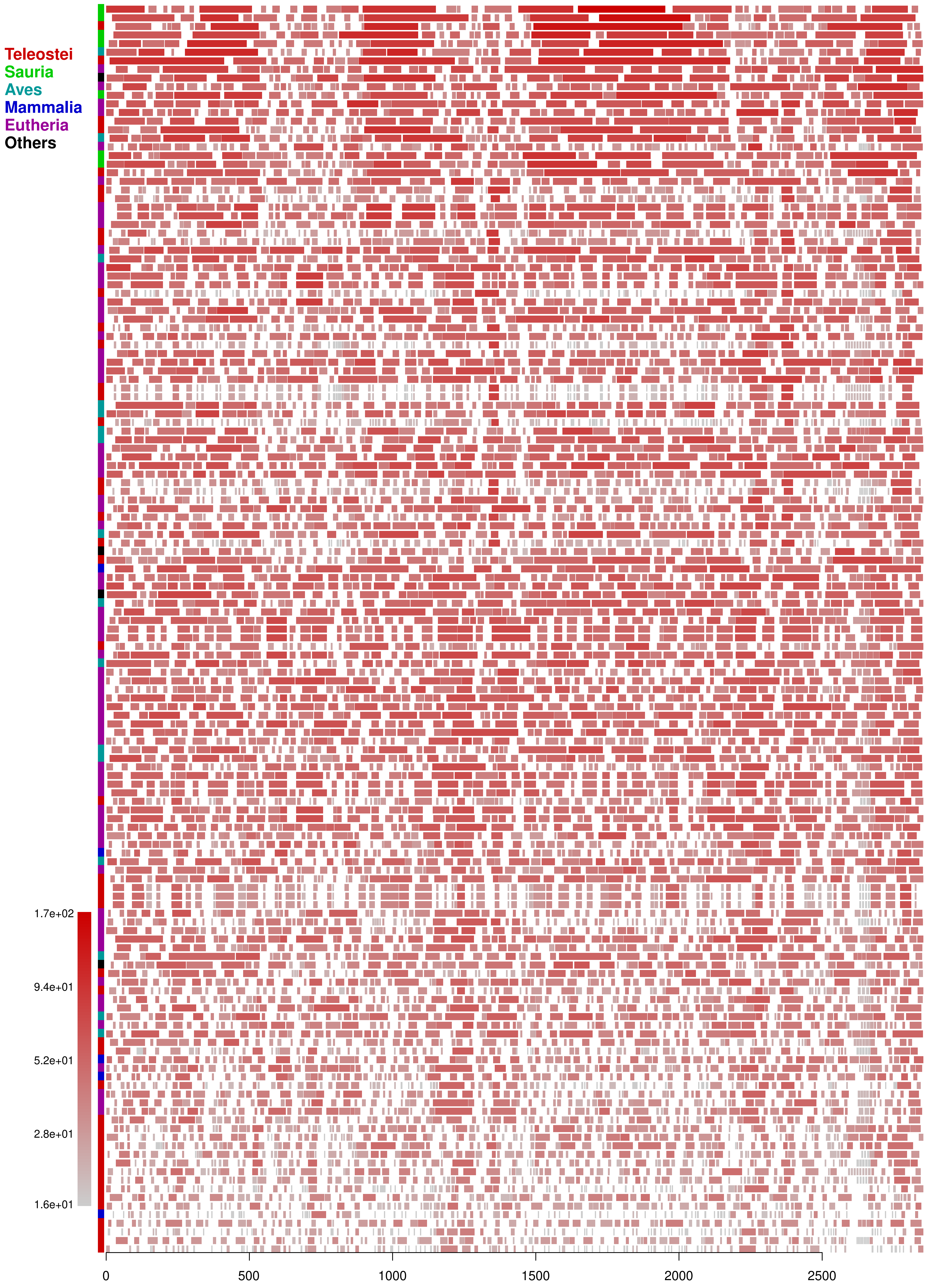

0 alignments above max size (1.0e+08)

ENSDARG00000039770 : ENSDART00000058147 intron no: 10 length: 2056

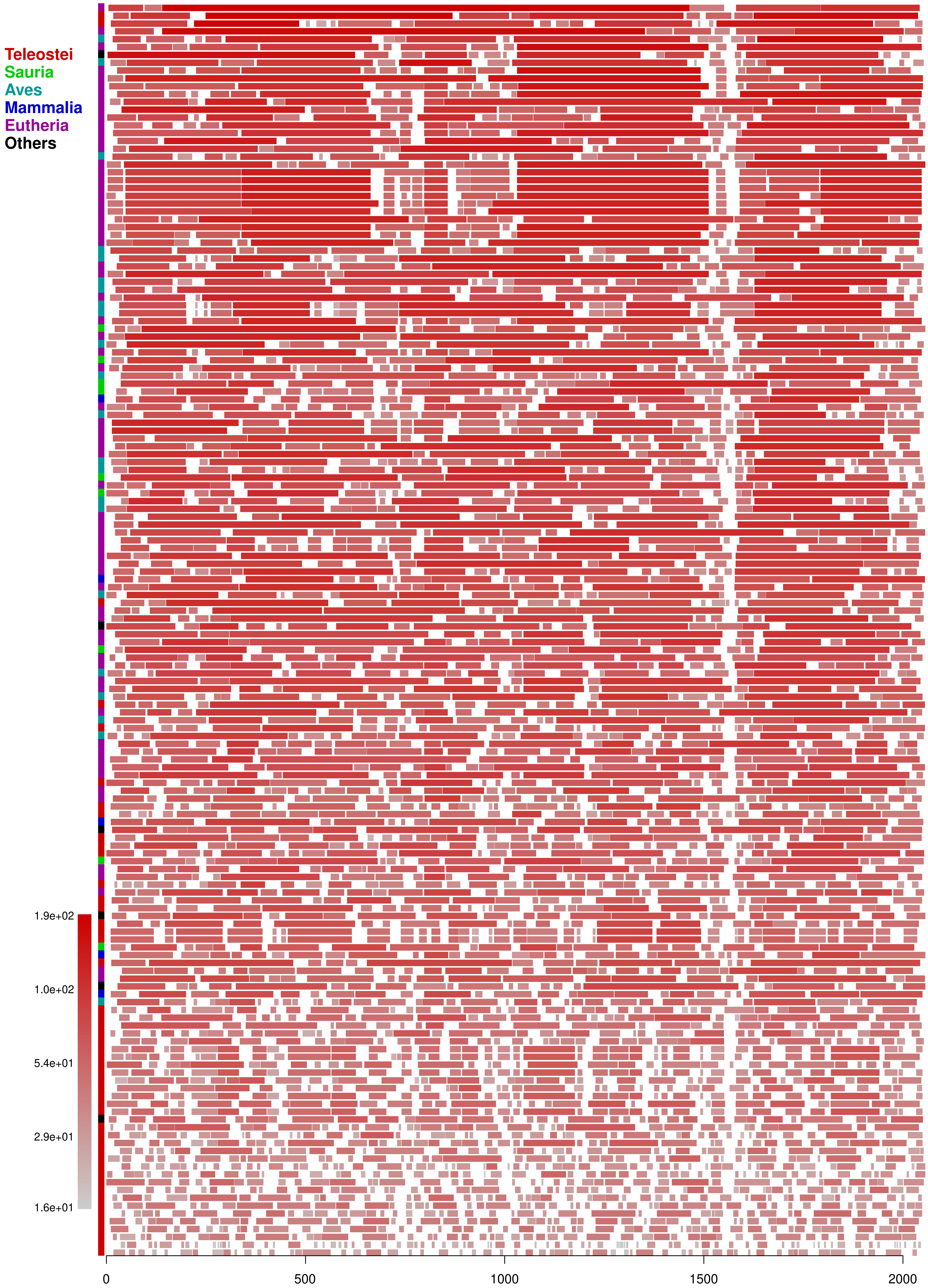

0 alignments above max size (1.0e+08)

ENSDARG00000076056 : ENSDART00000110282 intron no: 2 length: 2398

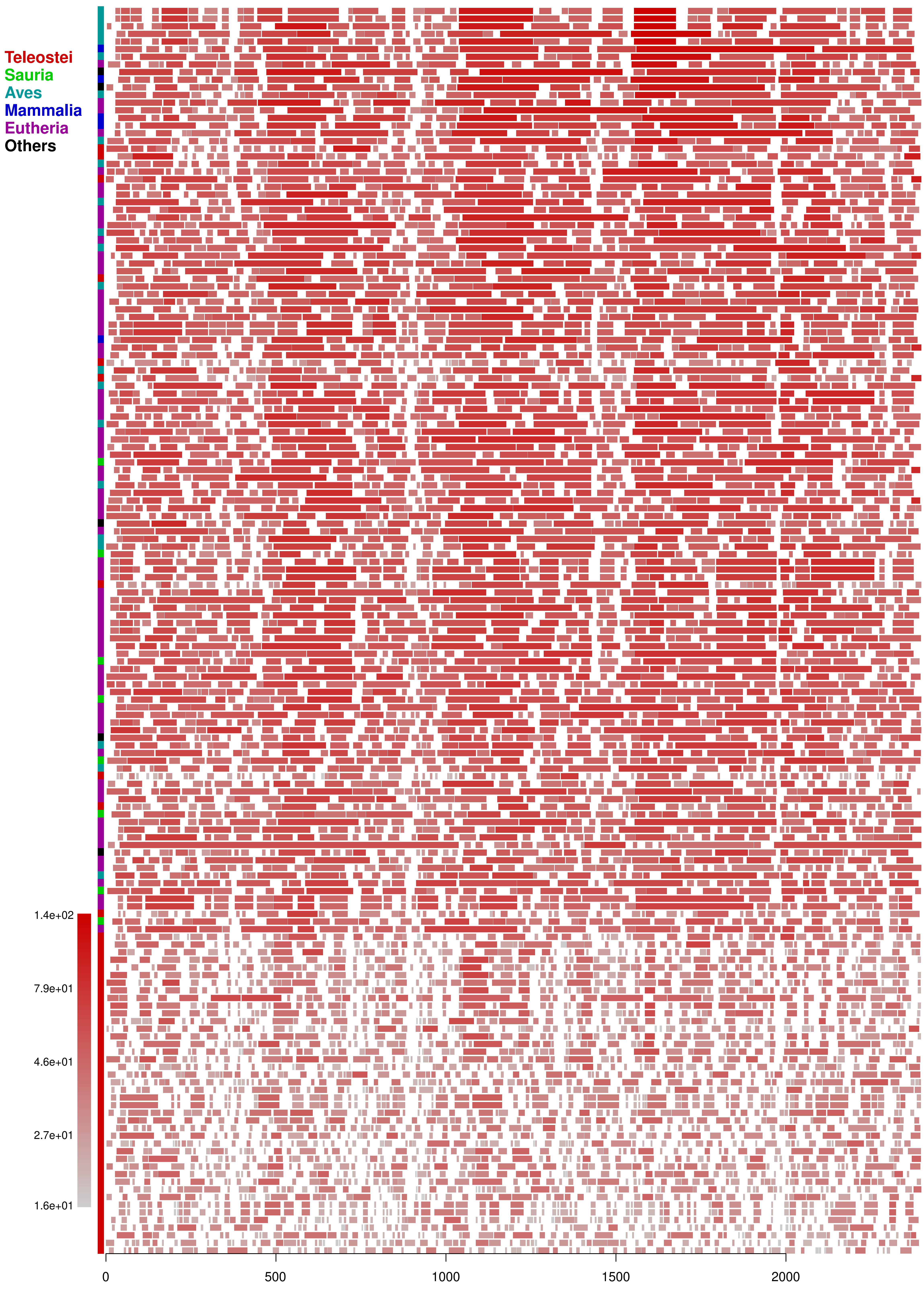

0 alignments above max size (1.0e+08)

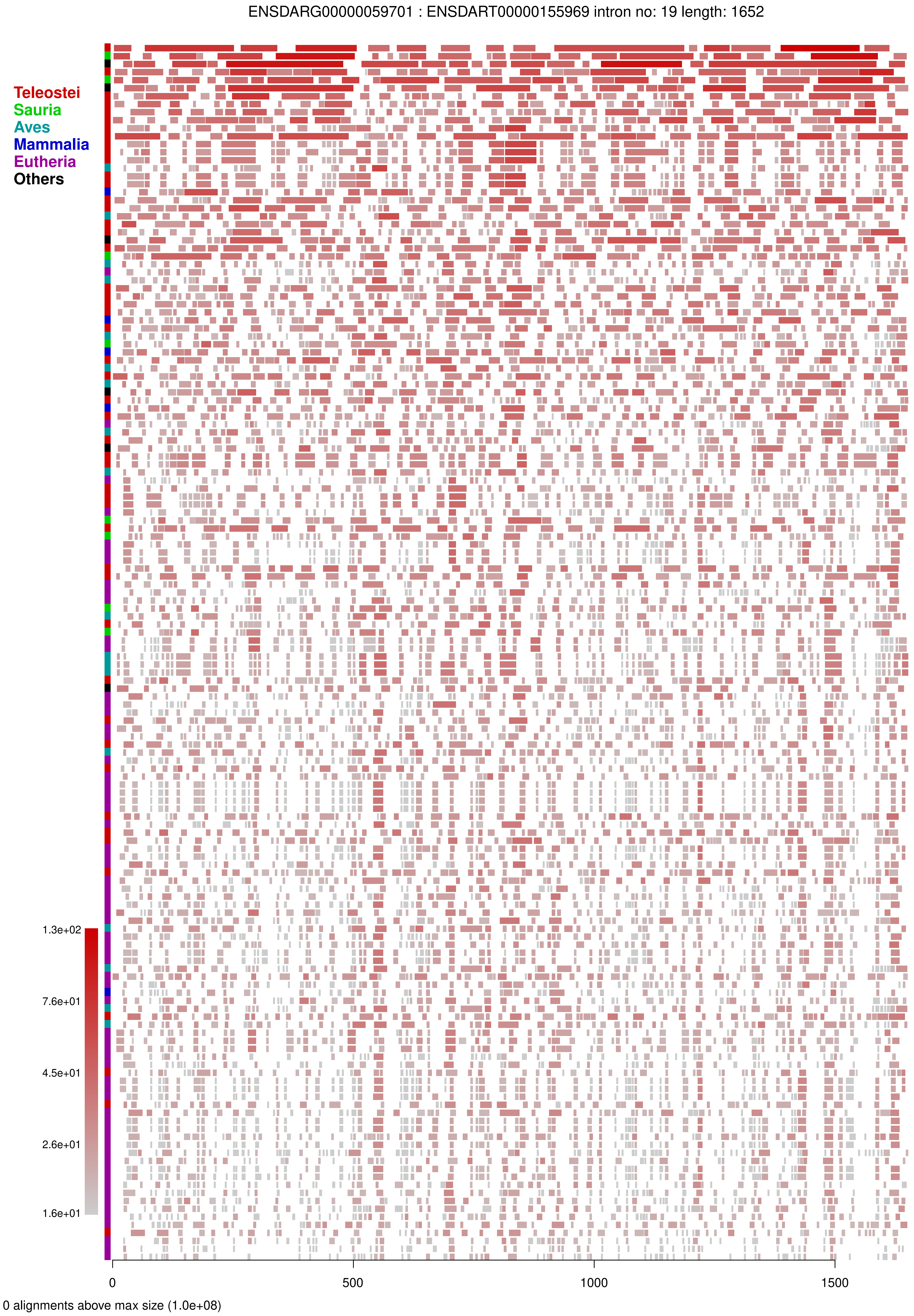

ENSDARG00000059701 : ENSDART00000155969 intron no: 19 length: 1652
Teleostei
Sauria
Aves
Mammalia
Eutheria
Others
1.3e+02
7.6e+01
4.5e+01
2.6e+01
1.6e+01
0
500
1000
1500
0 alignments above max size (1.0e+08)

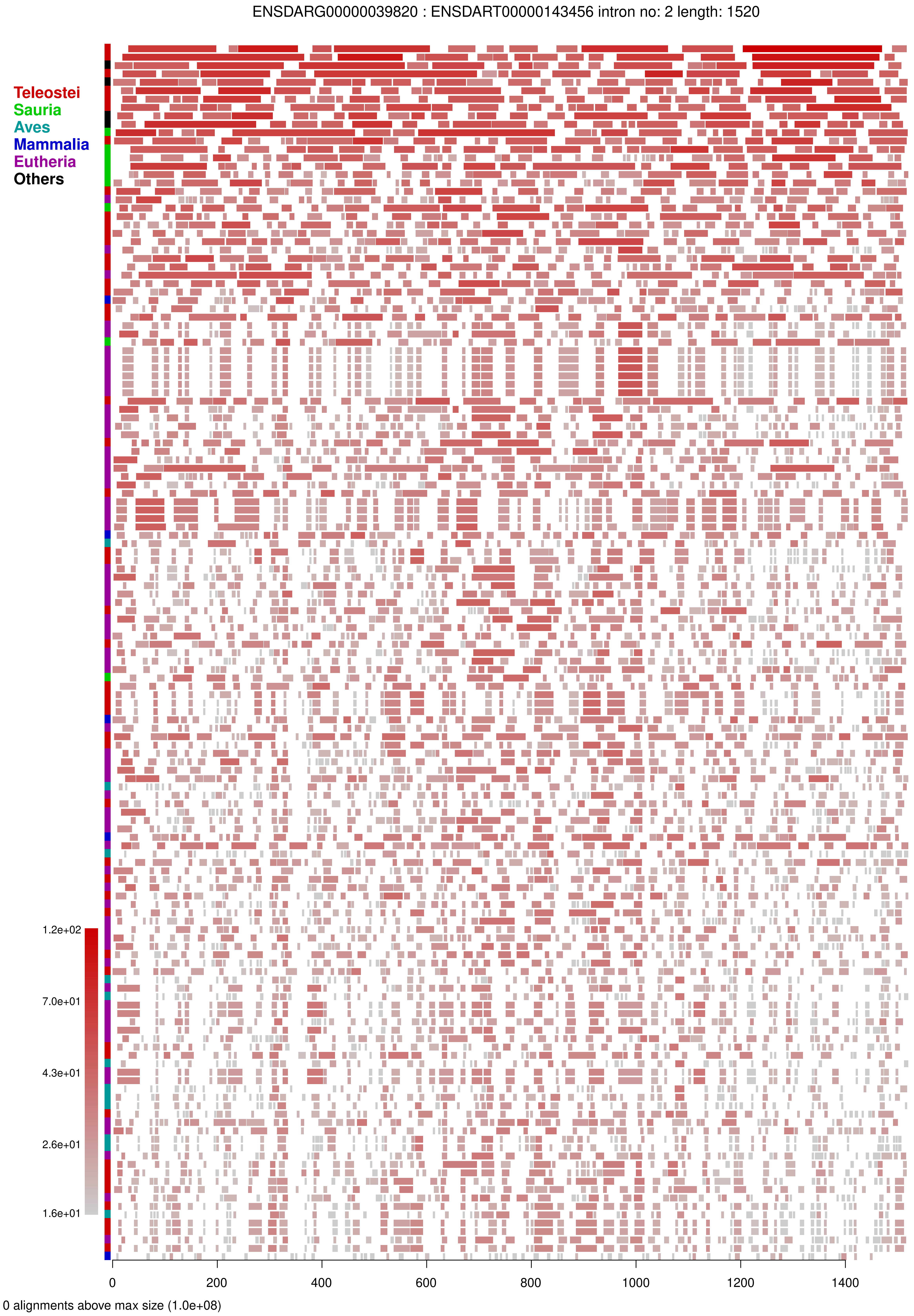

ENSDARG00000039820 : ENSDART00000143456 intron no: 2 length: 1520
Teleostei
Sauria
Aves
Mammalia
Eutheria
Others
1.2e+02
7.0e+01
4.3e+01
2.6e+01
1.6e+01
0
200
400
600
800
1000
1200
1400
0 alignments above max size (1.0e+08)

ENSDARG00000008188 : ENSDART00000166900 intron no: 9 length: 1298

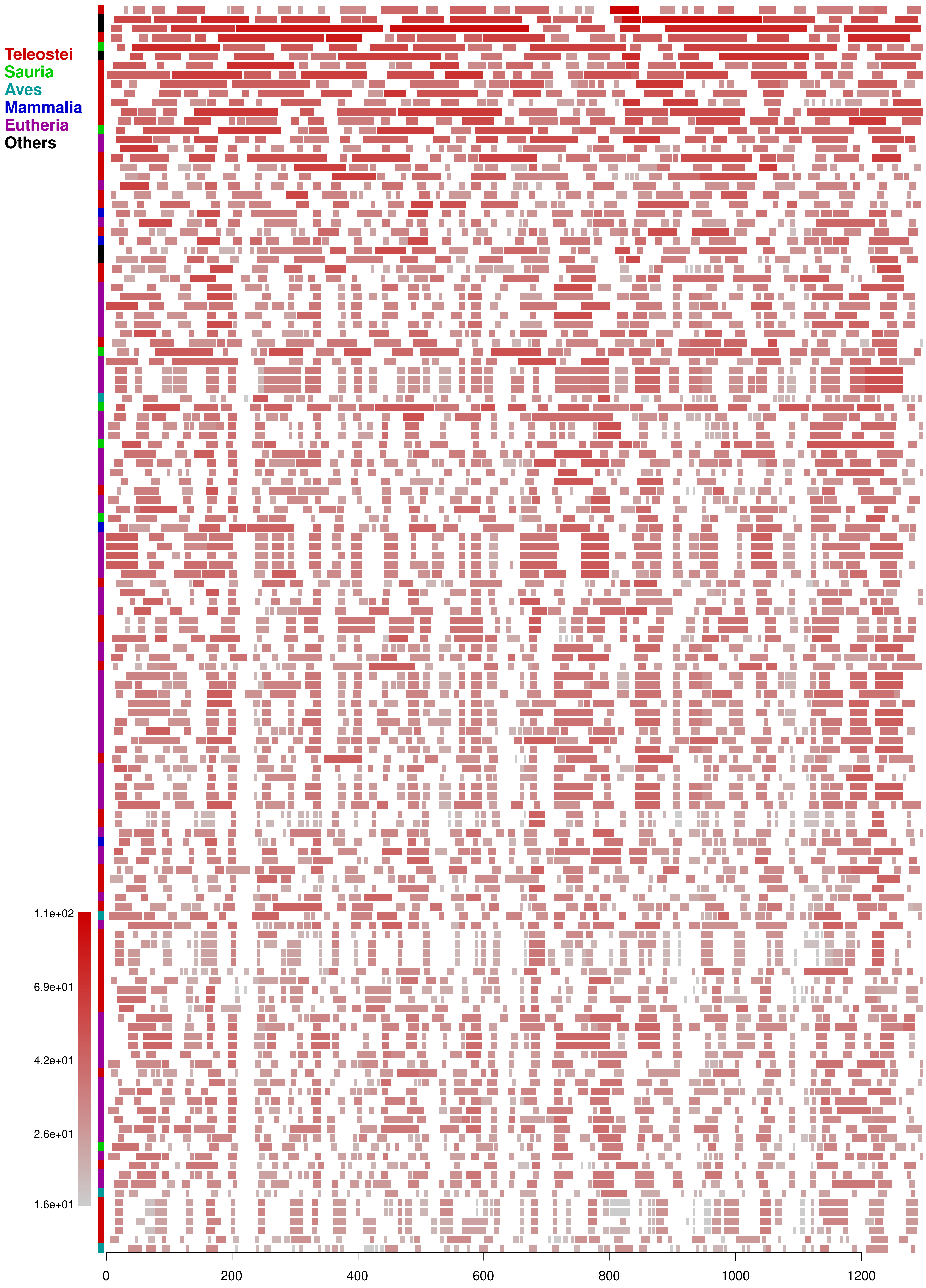

0 alignments above max size (1.0e+08)

ENSDARG00000102277 : ENSDART00000160069 intron no: 58 length: 2002

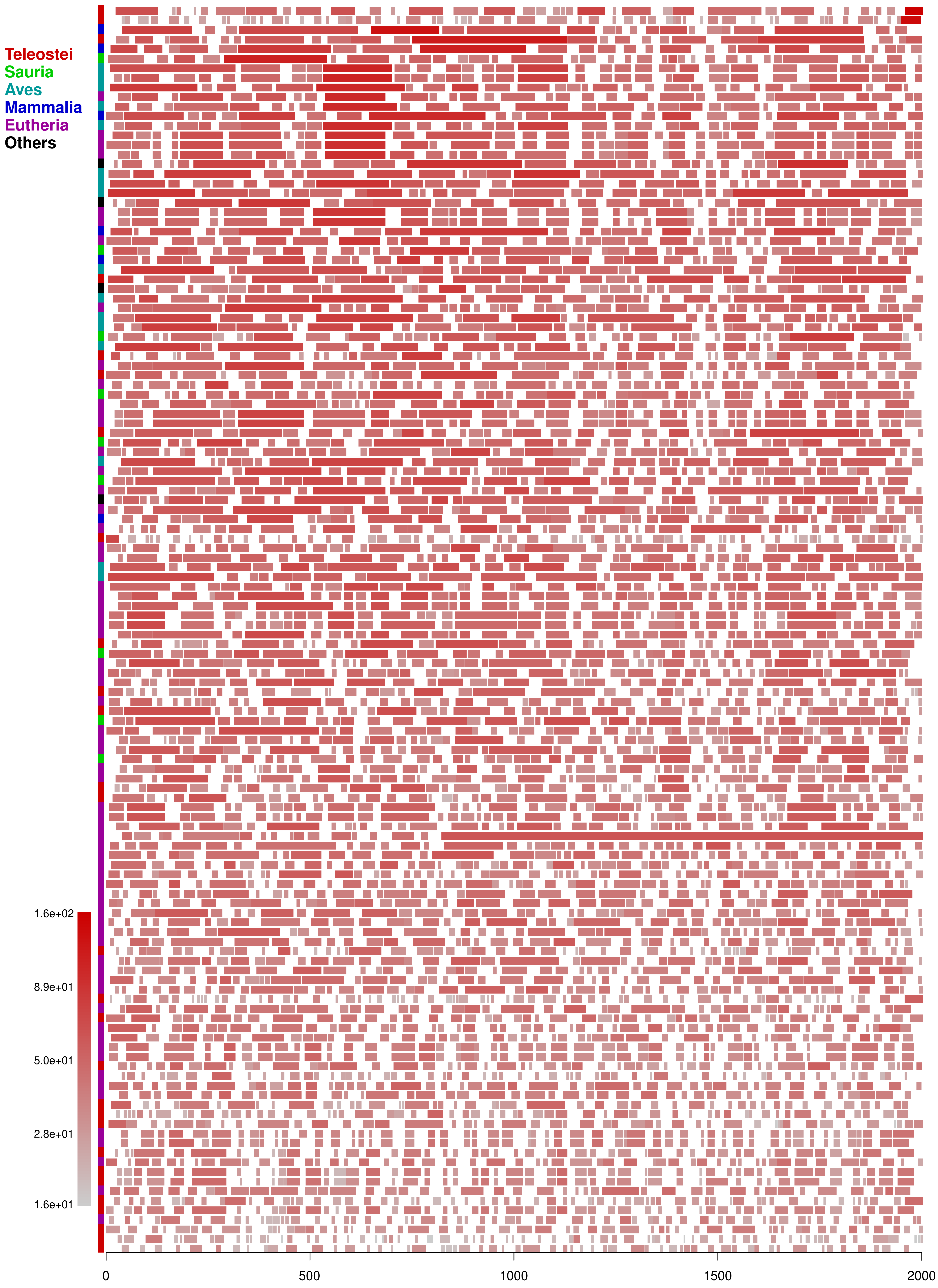

0 alignments above max size (1.0e+08)

ENSDARG00000036179 : ENSDART00000052539 intron no: 12 length: 1771

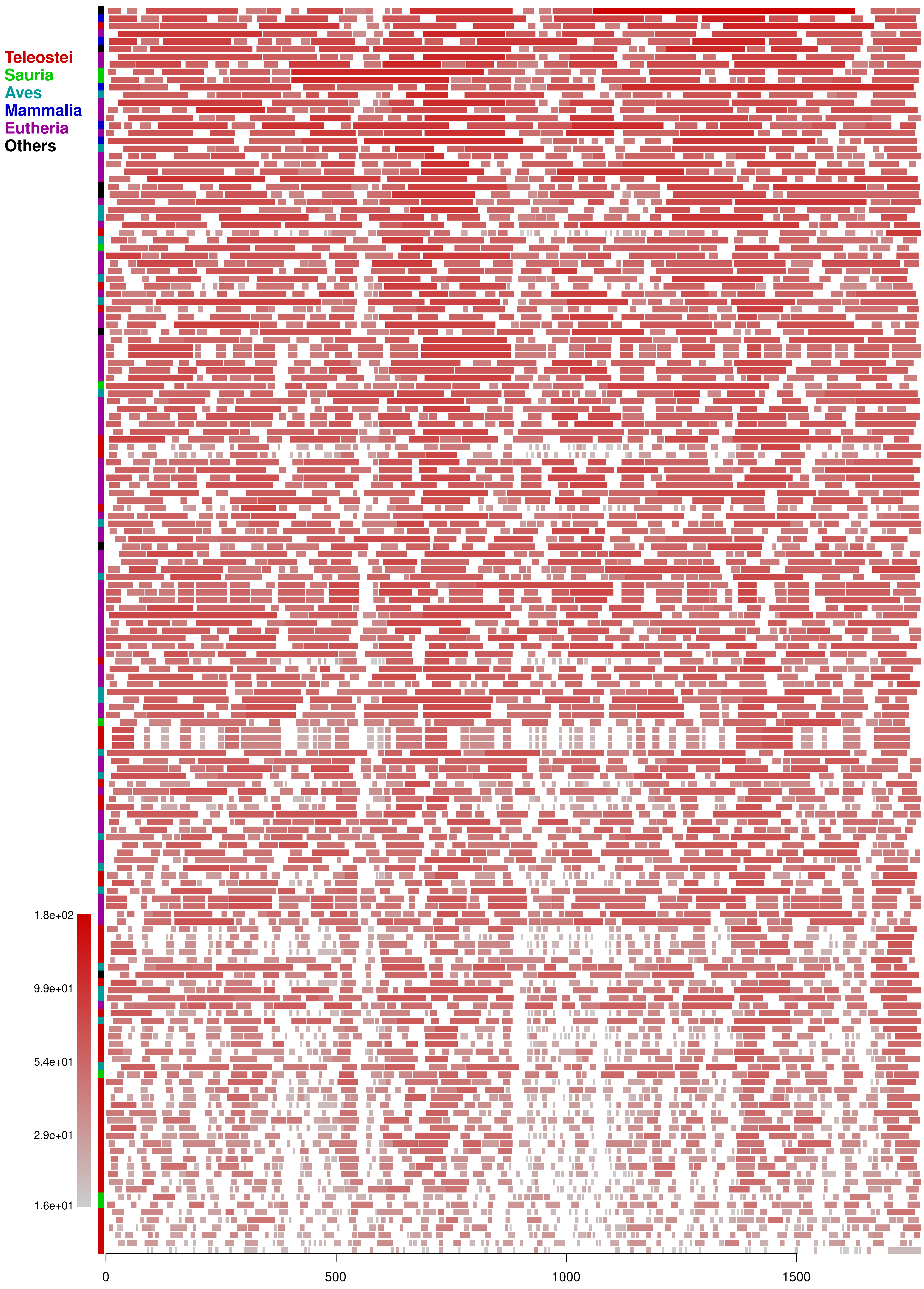

0 alignments above max size (1.0e+08)

ENSDARG00000076050 : ENSDART00000115111 intron no: 5 length: 2673

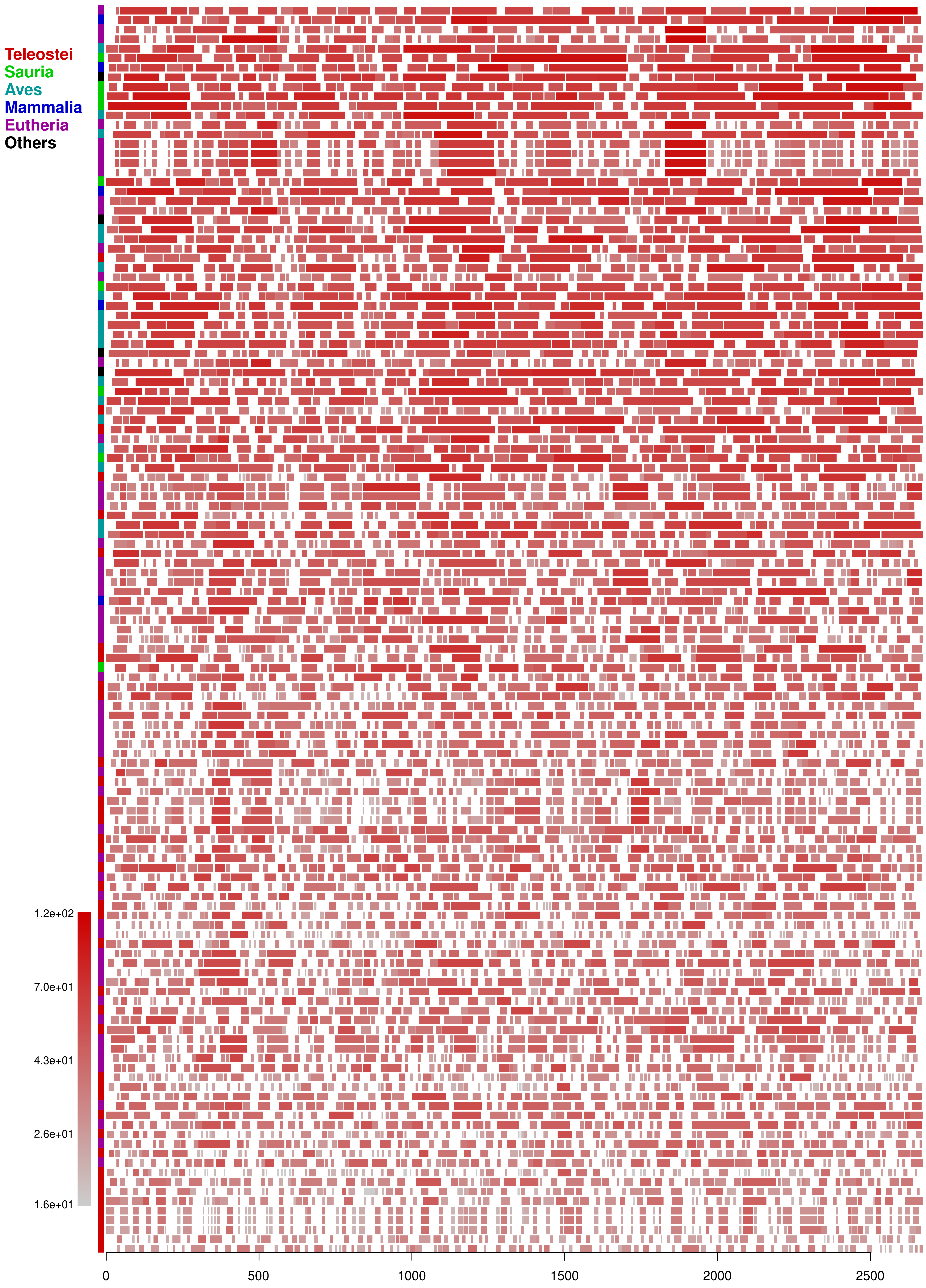

0 alignments above max size (1.0e+08)

ENSDARG00000035706 : ENSDART00000082944 intron no: 18 length: 2302

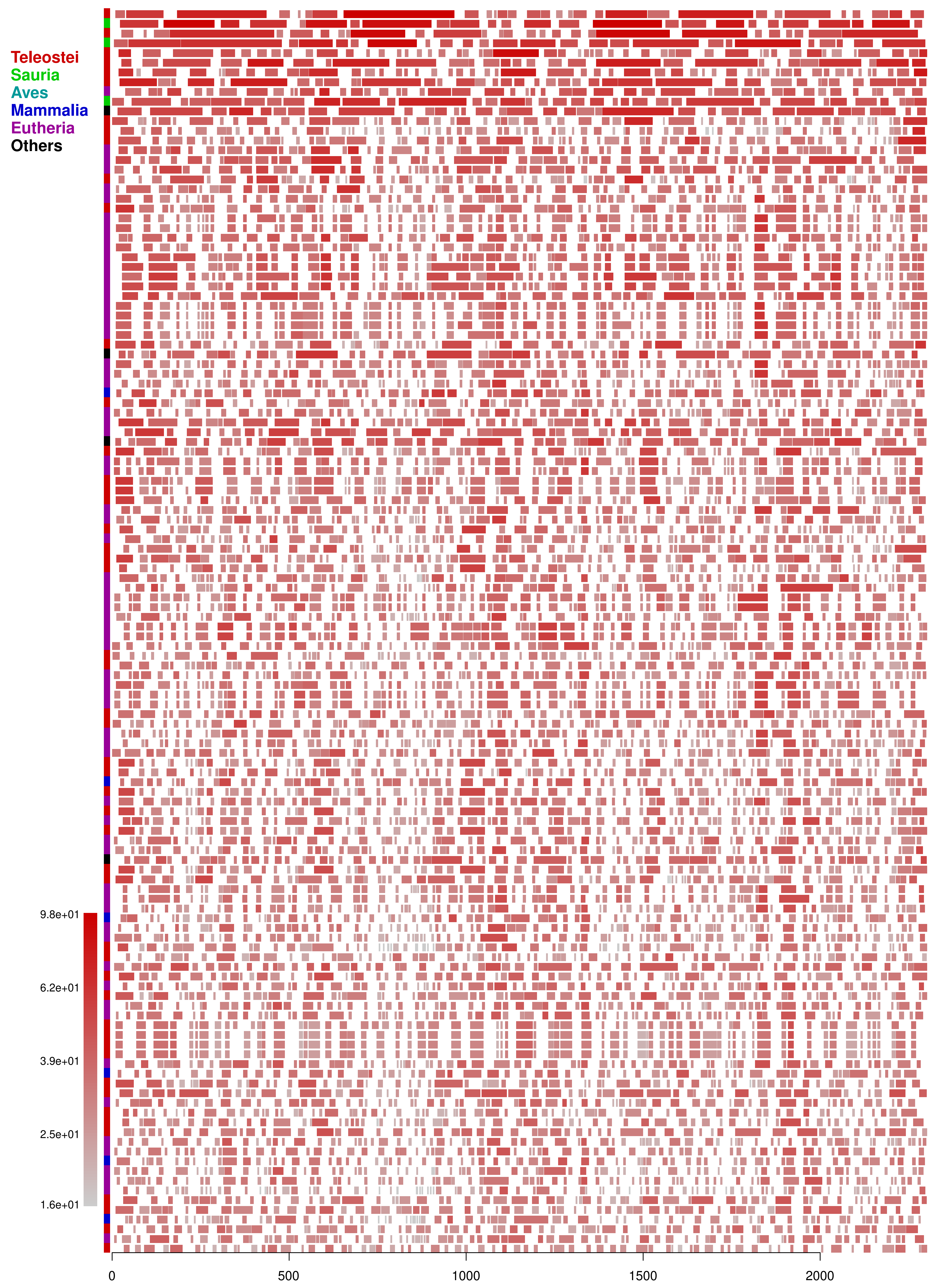

0 alignments above max size (1.0e+08)

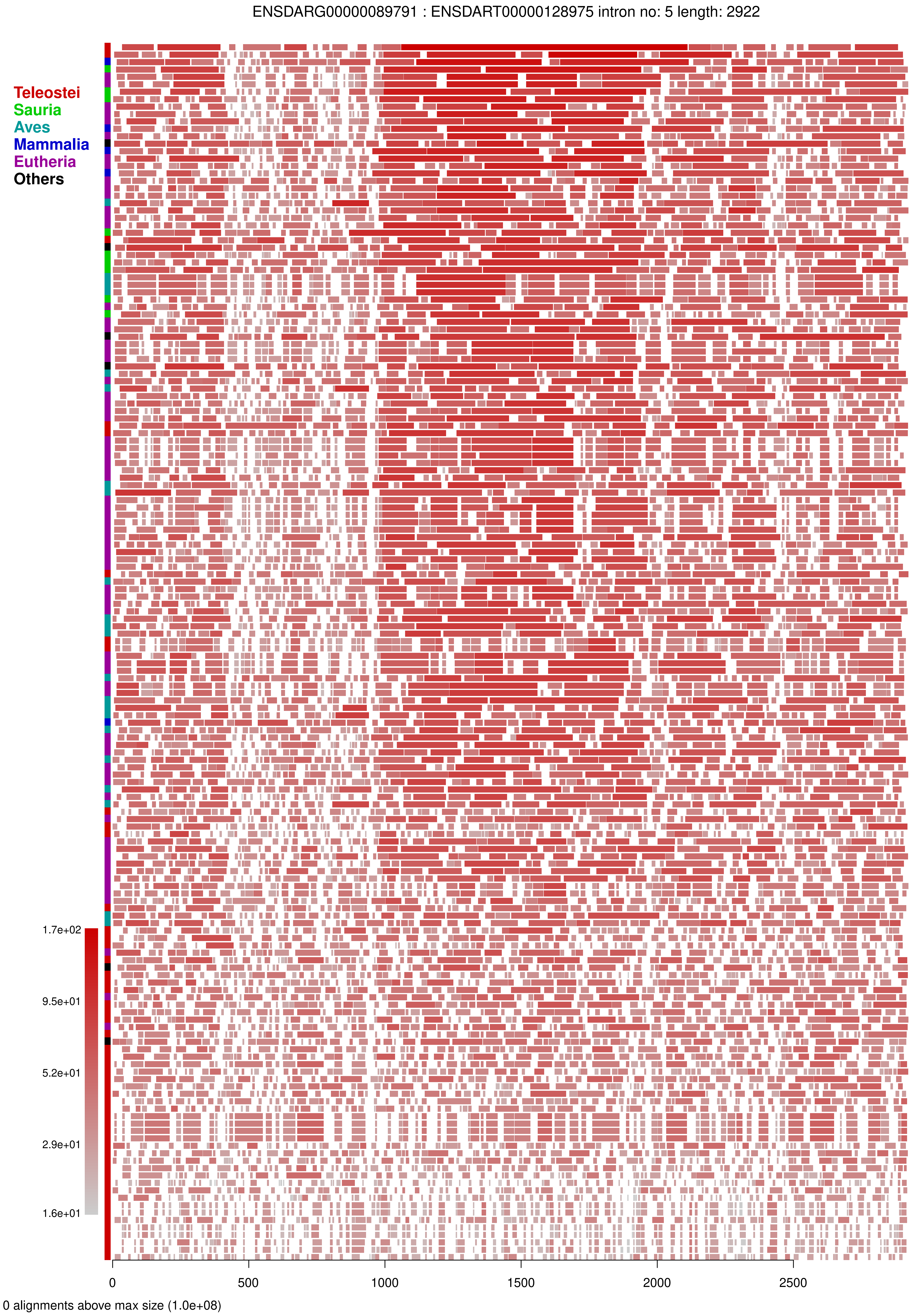

ENSDARG00000089791 : ENSDART00000128975 intron no: 5 length: 2922
Teleostei
Sauria
Aves
Mammalia
Eutheria
Others
1.7e+02
9.5e+01
5.2e+01
2.9e+01
1.6e+01
0
500
1000
1500
2000
2500
0 alignments above max size (1.0e+08)

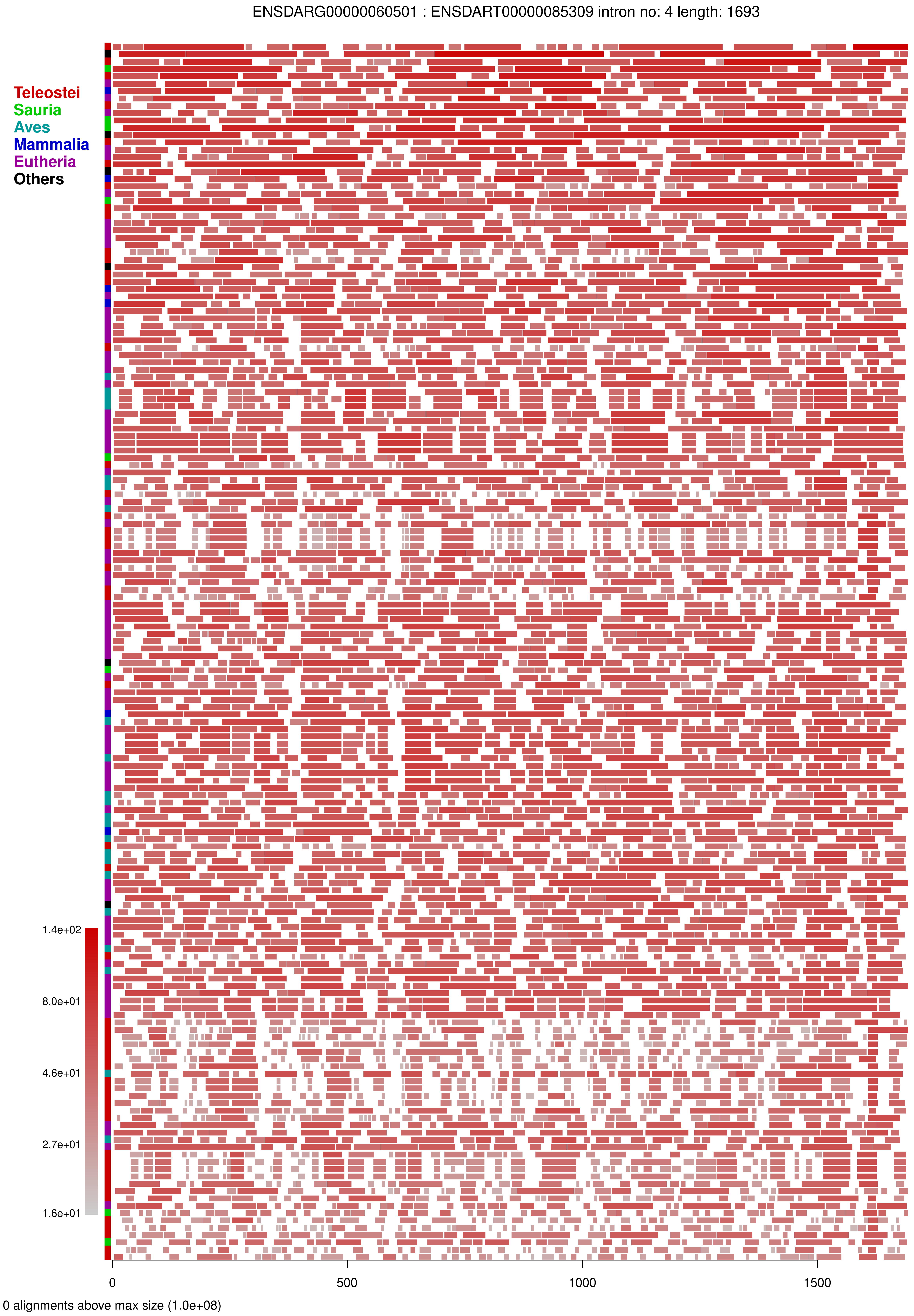

ENSDARG00000060501 : ENSDART00000085309 intron no: 4 length: 1693
Teleostei
Sauria
Aves
Mammalia
Eutheria
Others
1.4e+02
8.0e+01
4.6e+01
2.7e+01
1.6e+01
0
500
1000
1500
0 alignments above max size (1.0e+08)

ENSDARG00000062267 : ENSDART00000161713 intron no: 13 length: 4803

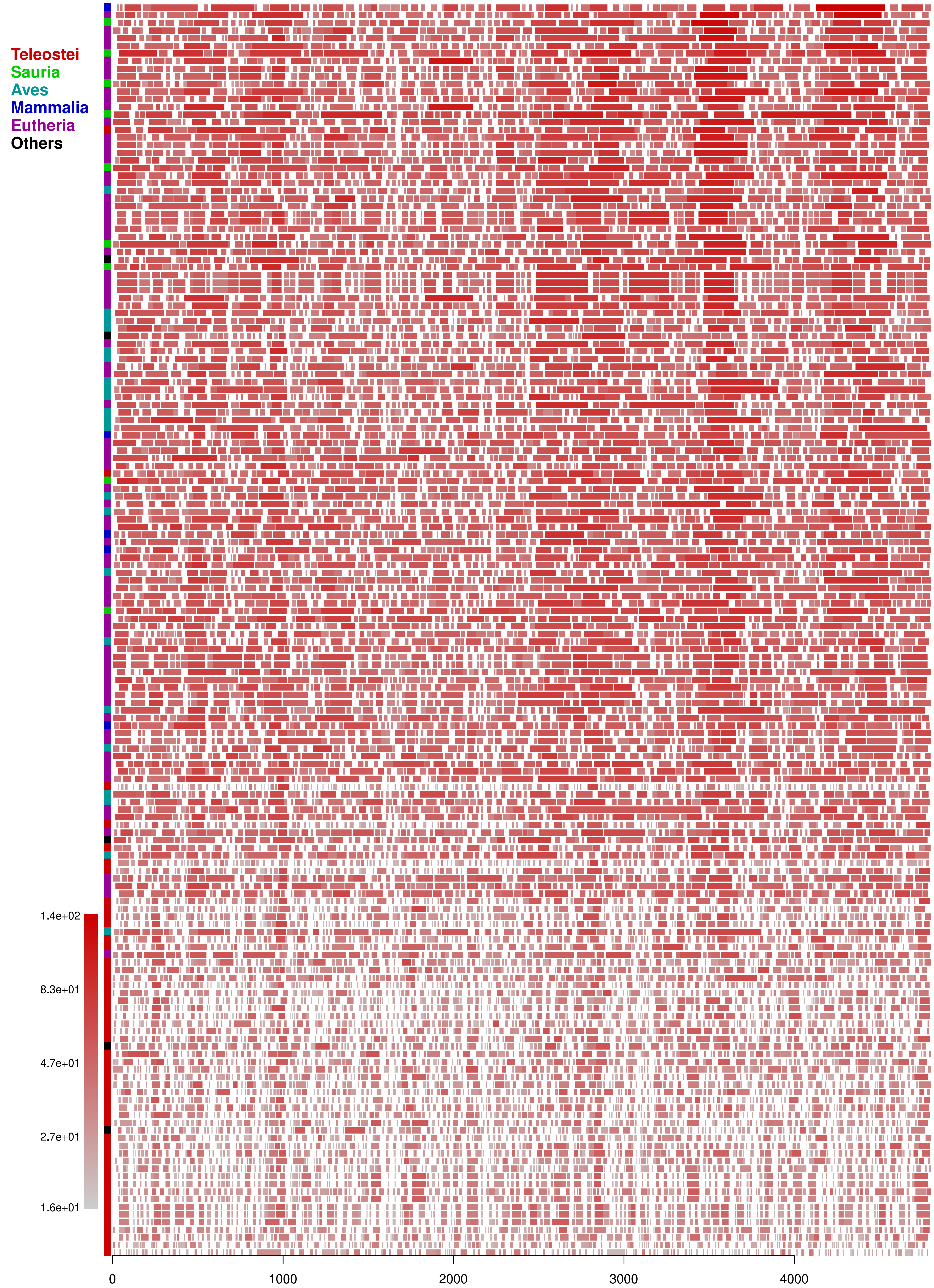

1 alignments above max size (1.0e+08)

ENSDARG00000017612 : ENSDART00000022724 intron no: 5 length: 3238

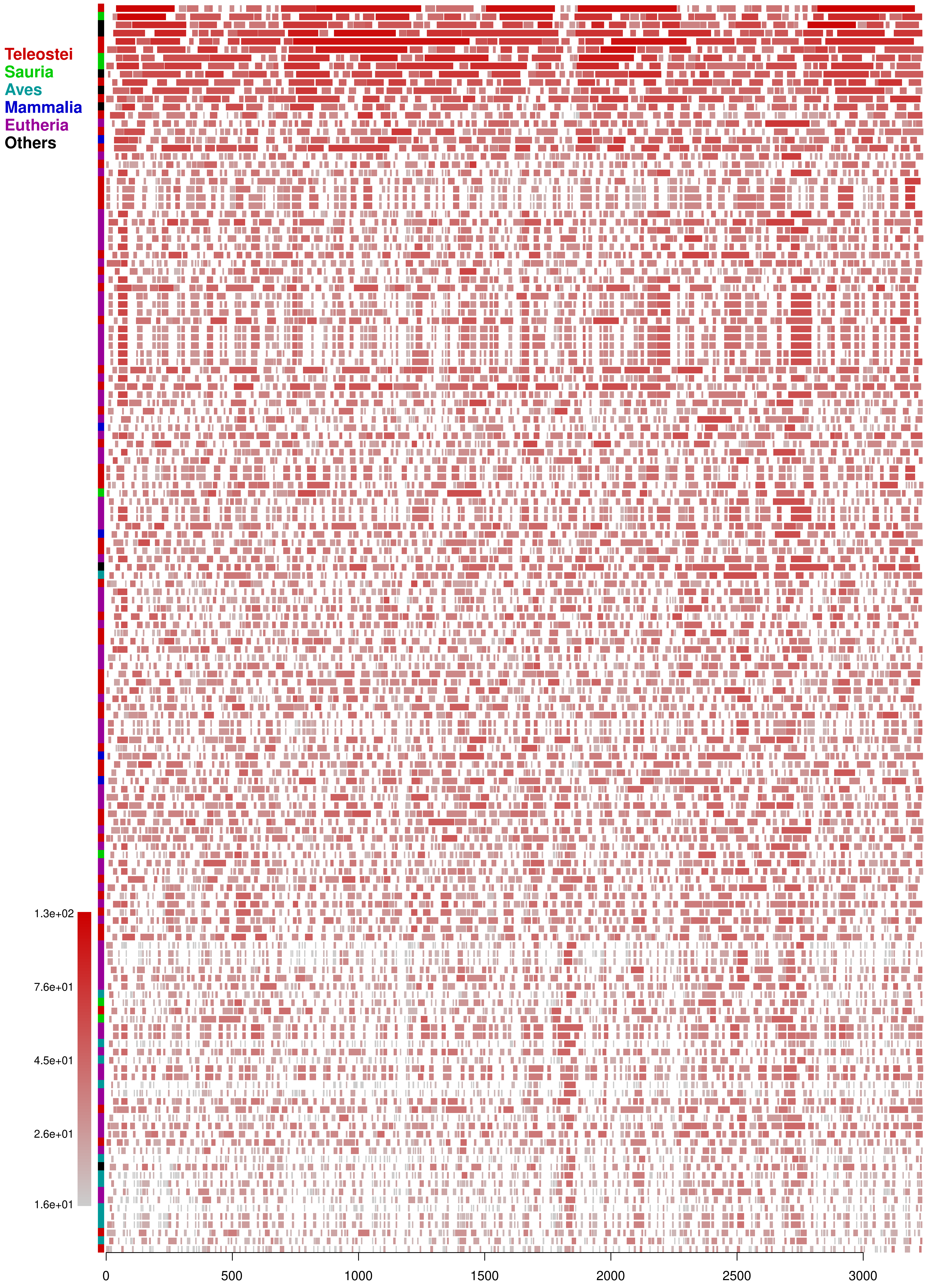

0 alignments above max size (1.0e+08)

ENSDARG00000074842 : ENSDART00000109392 intron no: 4 length: 6015

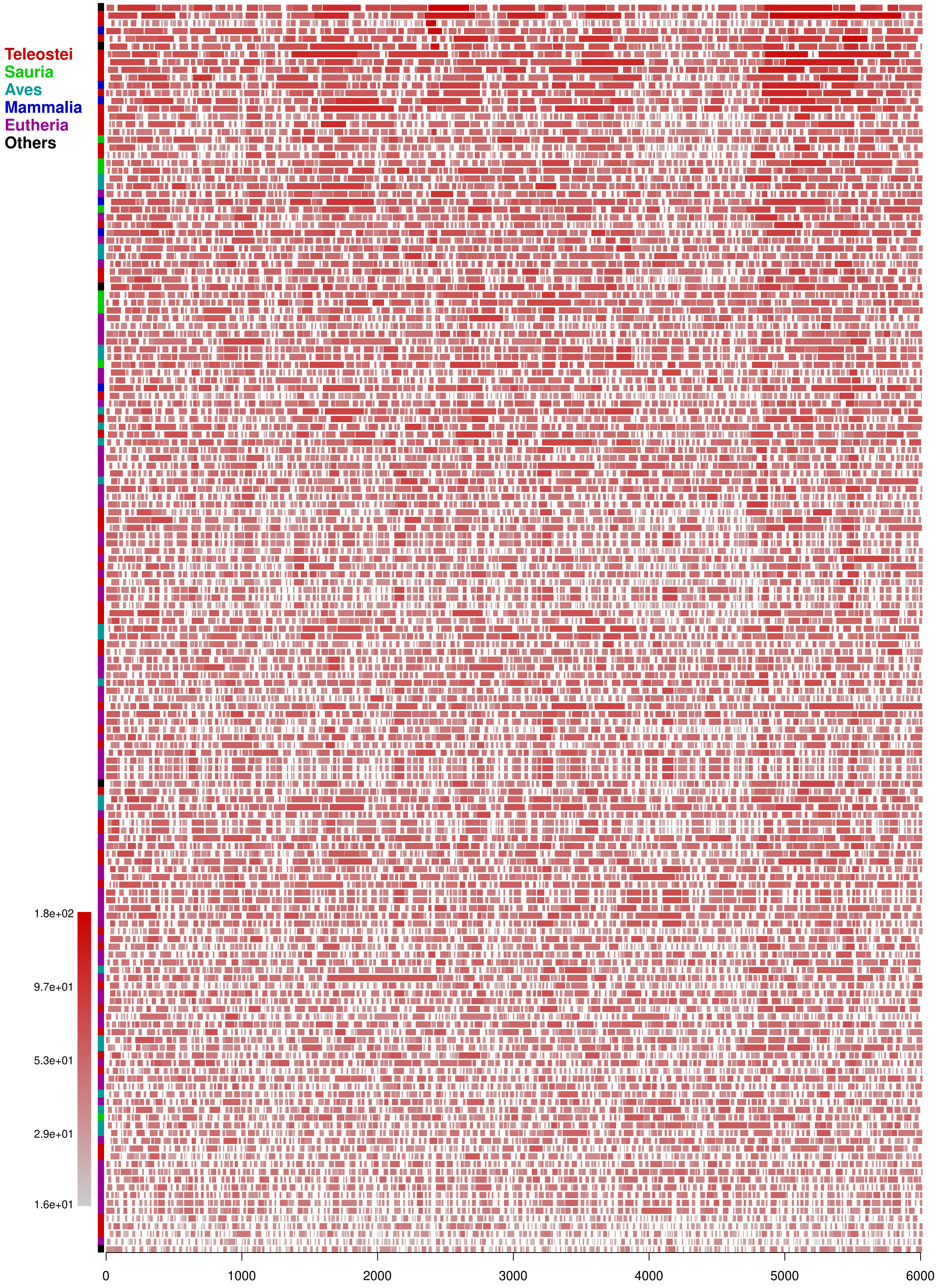

0 alignments above max size (1.0e+08)

ENSDARG00000011125 : ENSDART00000011663 intron no: 4 length: 1300

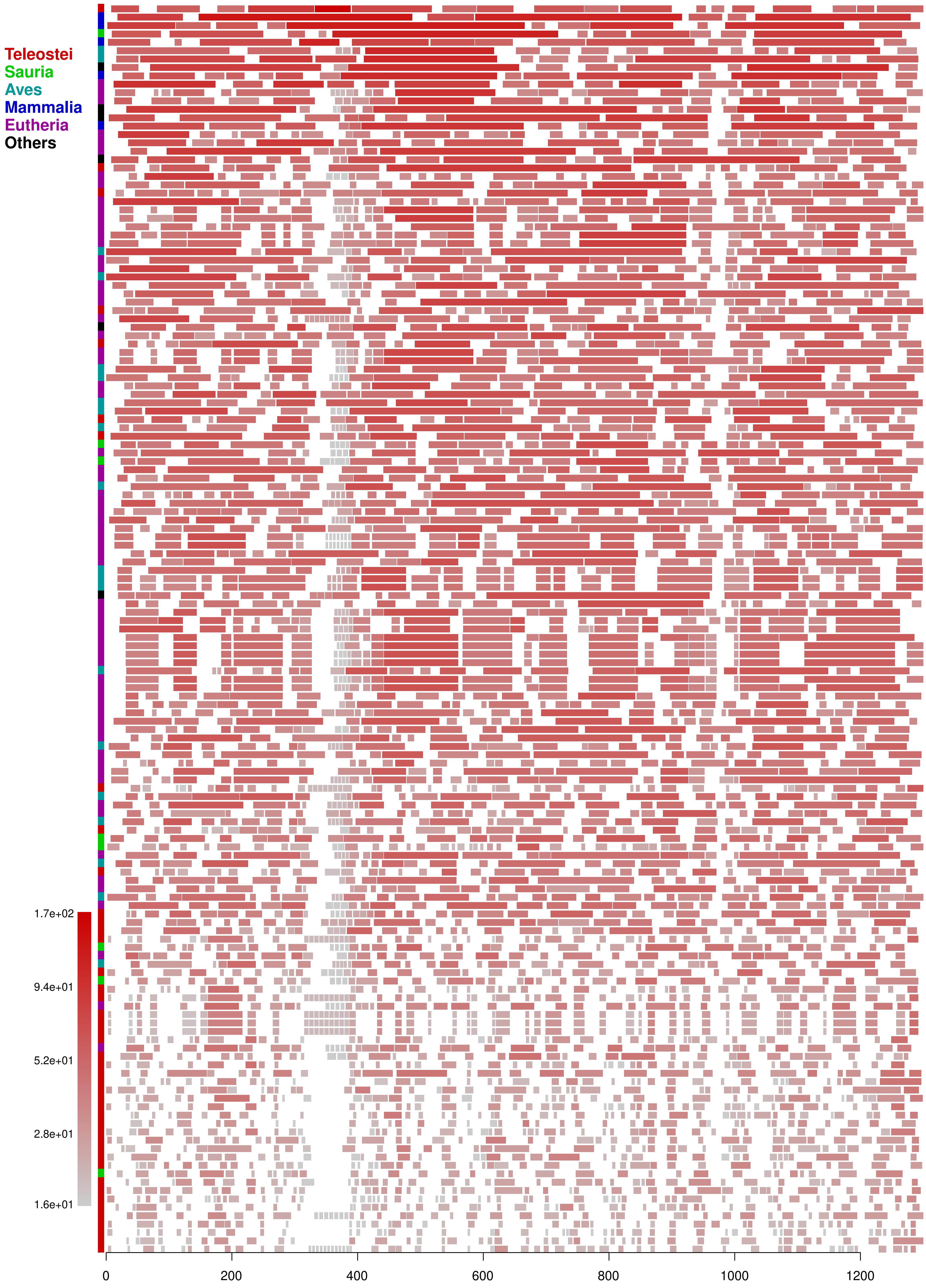

0 alignments above max size (1.0e+08)

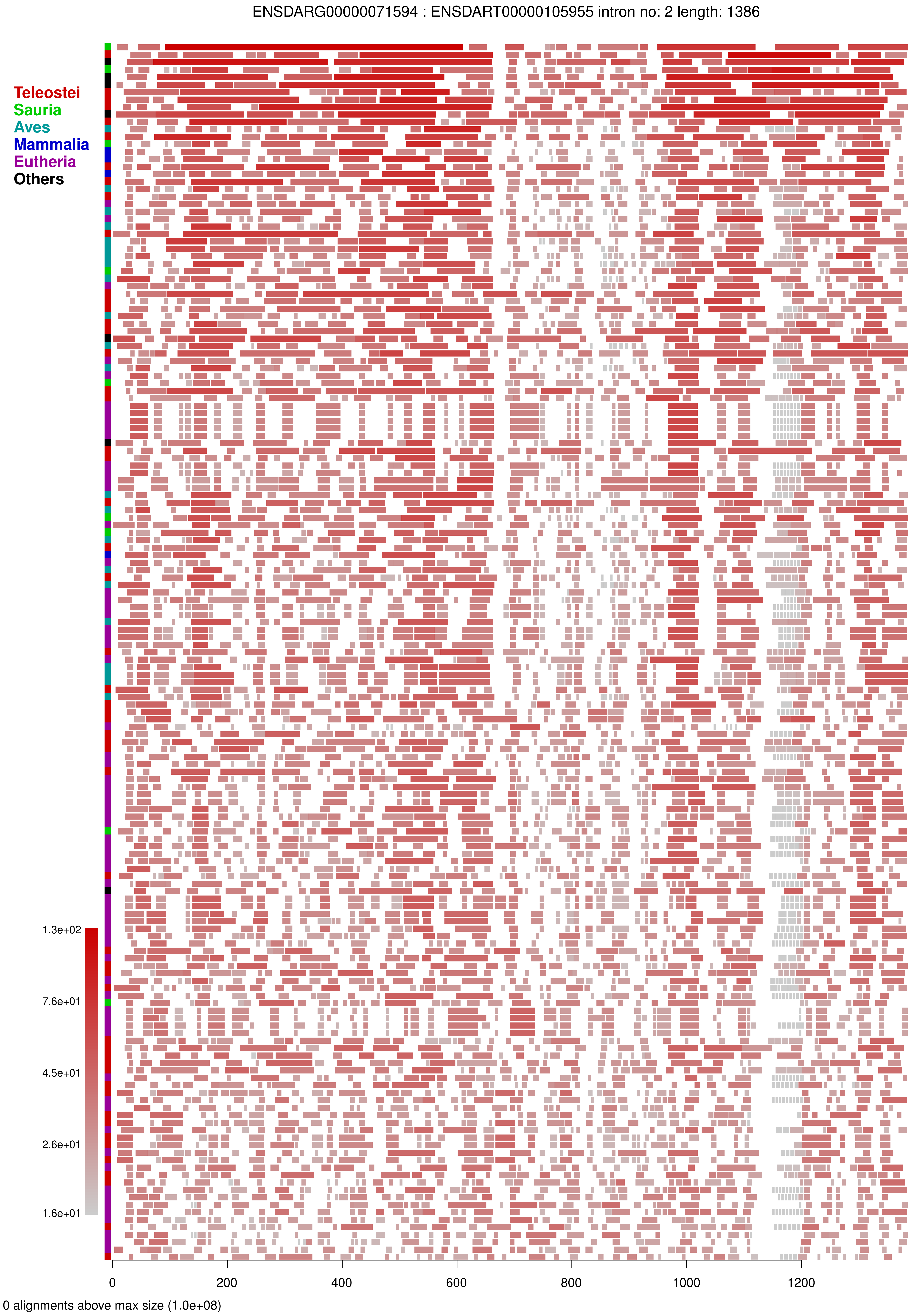
ENSDARG00000071594 : ENSDART00000105955 intron no: 2 length: 1386
Teleostei
Sauria
Aves
Mammalia
Eutheria
Others
1.3e+02
7.6e+01
4.5e+01
2.6e+01
1.6e+01
0
200
400
600
800
1000
1200
0 alignments above max size (1.0e+08)

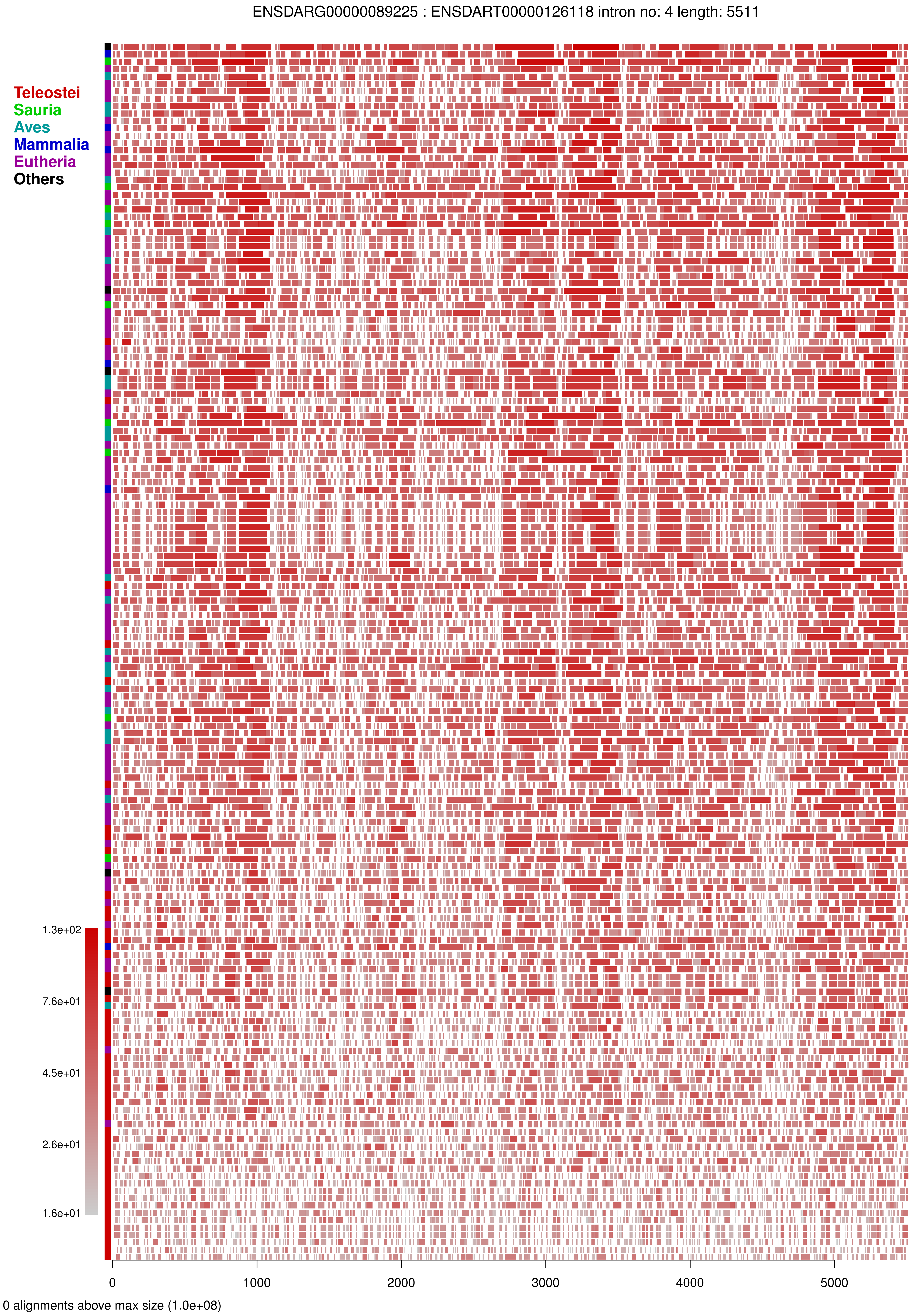

ENSDARG00000089225 : ENSDART00000126118 intron no: 4 length: 5511
Teleostei
Sauria
Aves
Mammalia
Eutheria
Others
1.3e+02
7.6e+01
4.5e+01
2.6e+01
1.6e+01
0
1000
2000
3000
4000
5000
0 alignments above max size (1.0e+08)

ENSDARG00000069326 : ENSDART00000100782 intron no: 8 length: 2810

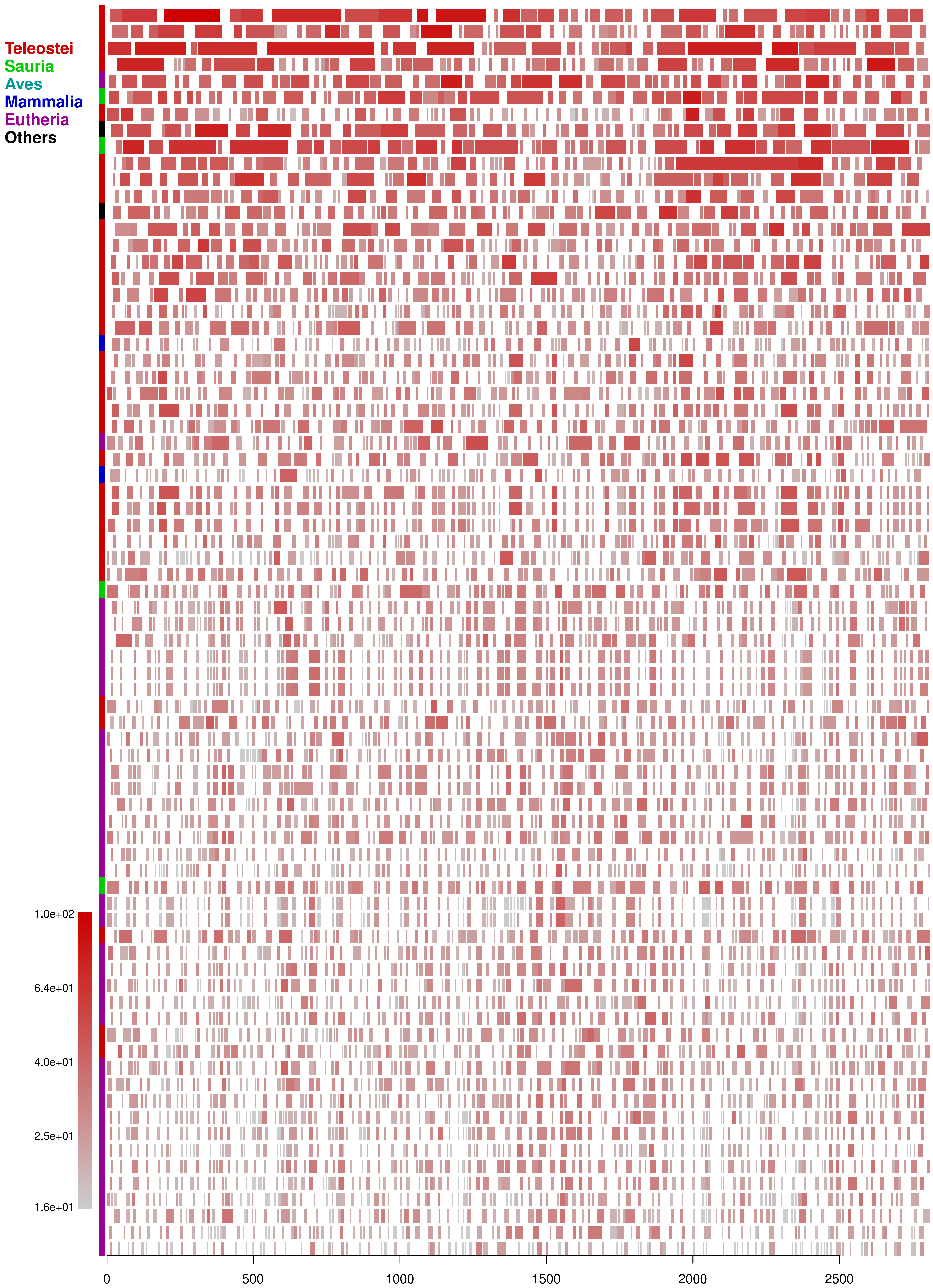

0 alignments above max size (1.0e+08)

ENSDARG00000004343 : ENSDART00000148877 intron no: 5 length: 2270

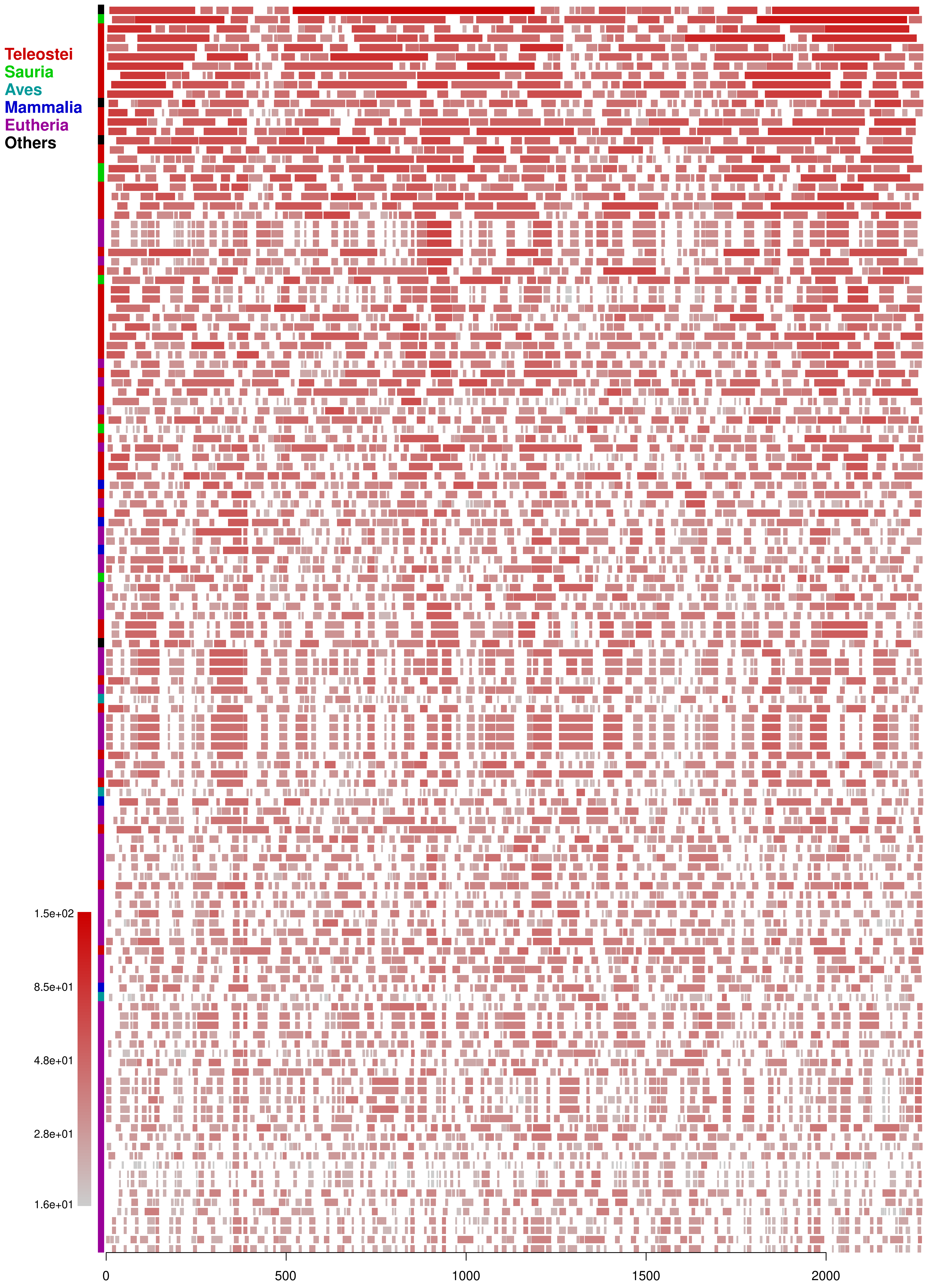

0 alignments above max size (1.0e+08)

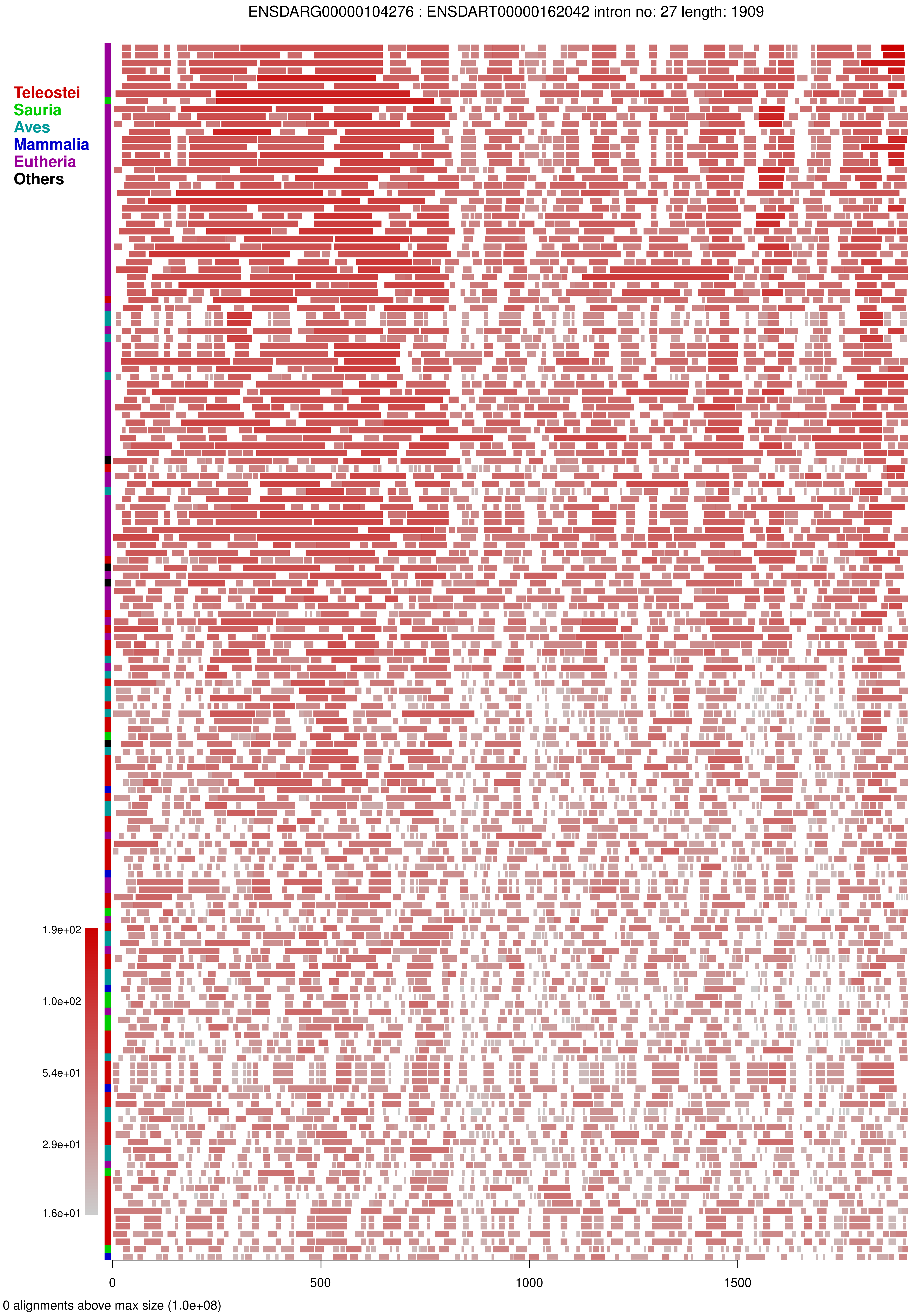

ENSDARG00000104276 : ENSDART00000162042 intron no: 27 length: 1909
Teleostei
Sauria
Aves
Mammalia
Eutheria
Others
1.9e+02
1.0e+02
5.4e+01
2.9e+01
1.6e+01
0
500
1000
1500
0 alignments above max size (1.0e+08)

ENSDARG00000061397 : ENSDART00000087566 intron no: 31 length: 1713

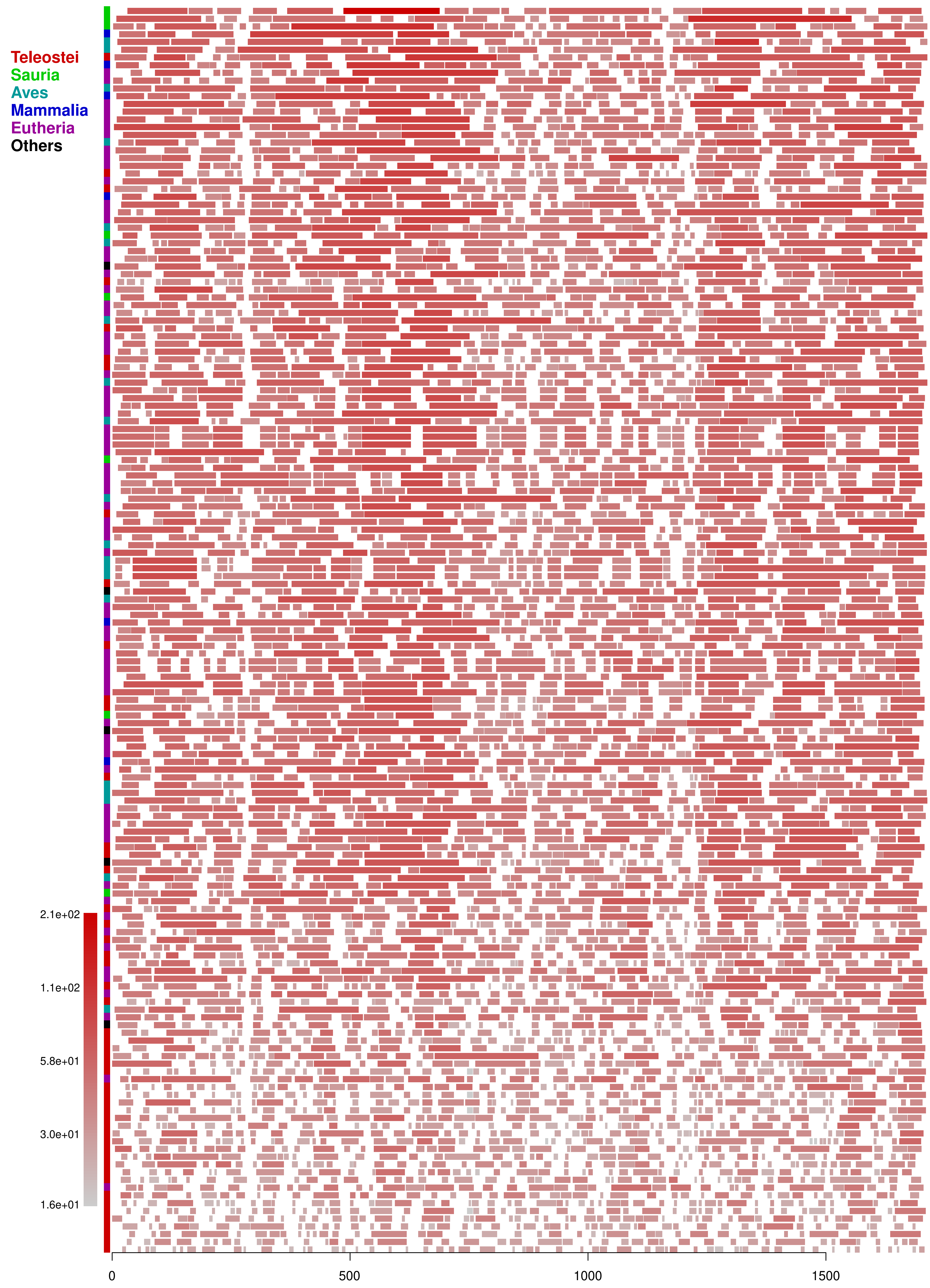

0 alignments above max size (1.0e+08)

ENSDARG00000027749 : ENSDART00000037906 intron no: 4 length: 1540

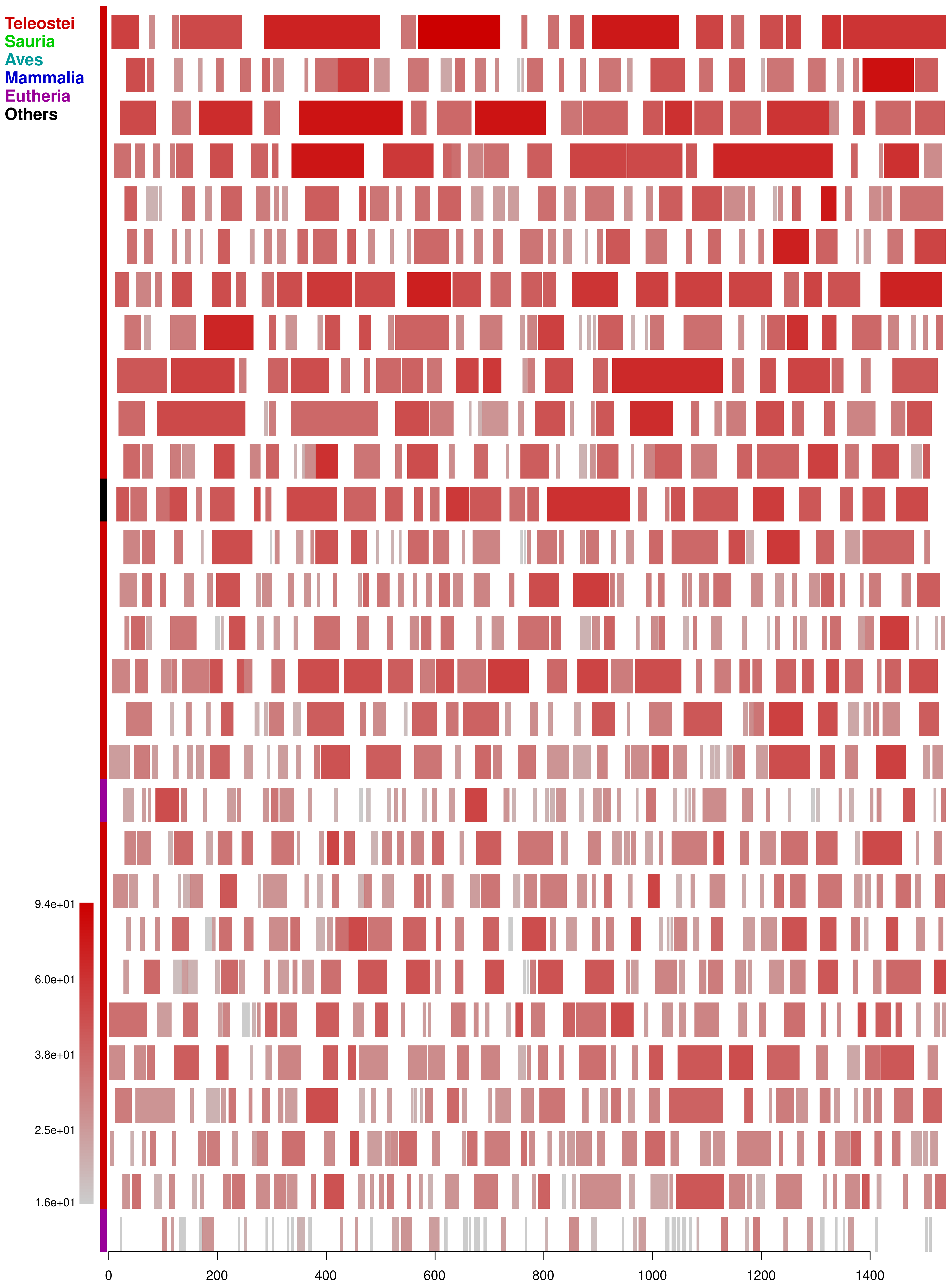

0 alignments above max size (1.0e+08)

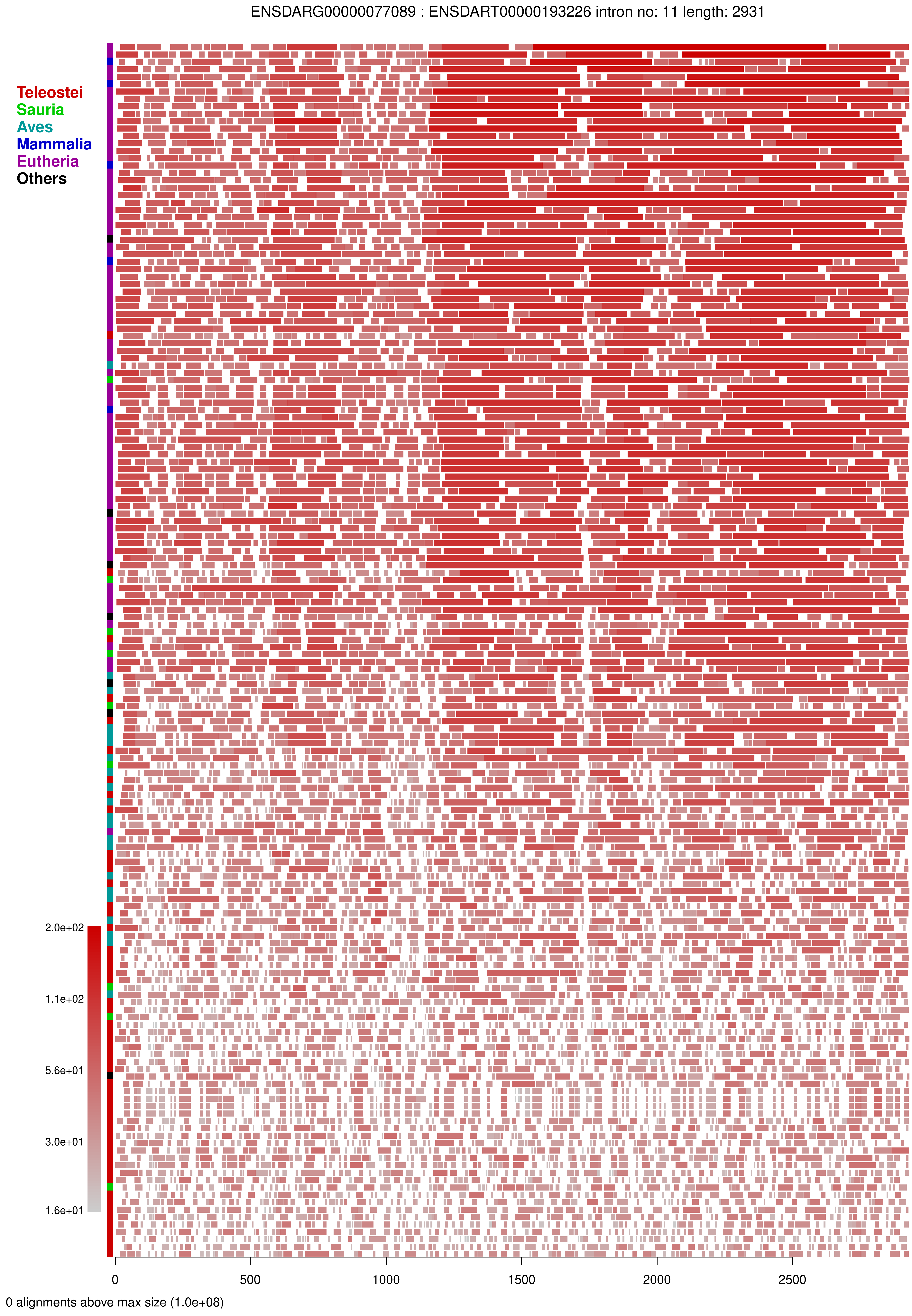
ENSDARG00000077089 : ENSDART00000193226 intron no: 11 length: 2931
Teleostei
Sauria
Aves
Mammalia
Eutheria
Others
2.0e+02
1.1e+02
5.6e+01
3.0e+01
1.6e+01
0
500
1000
1500
2000
2500
0 alignments above max size (1.0e+08)

ENSDARG00000060506 : ENSDART00000085319 intron no: 15 length: 2544

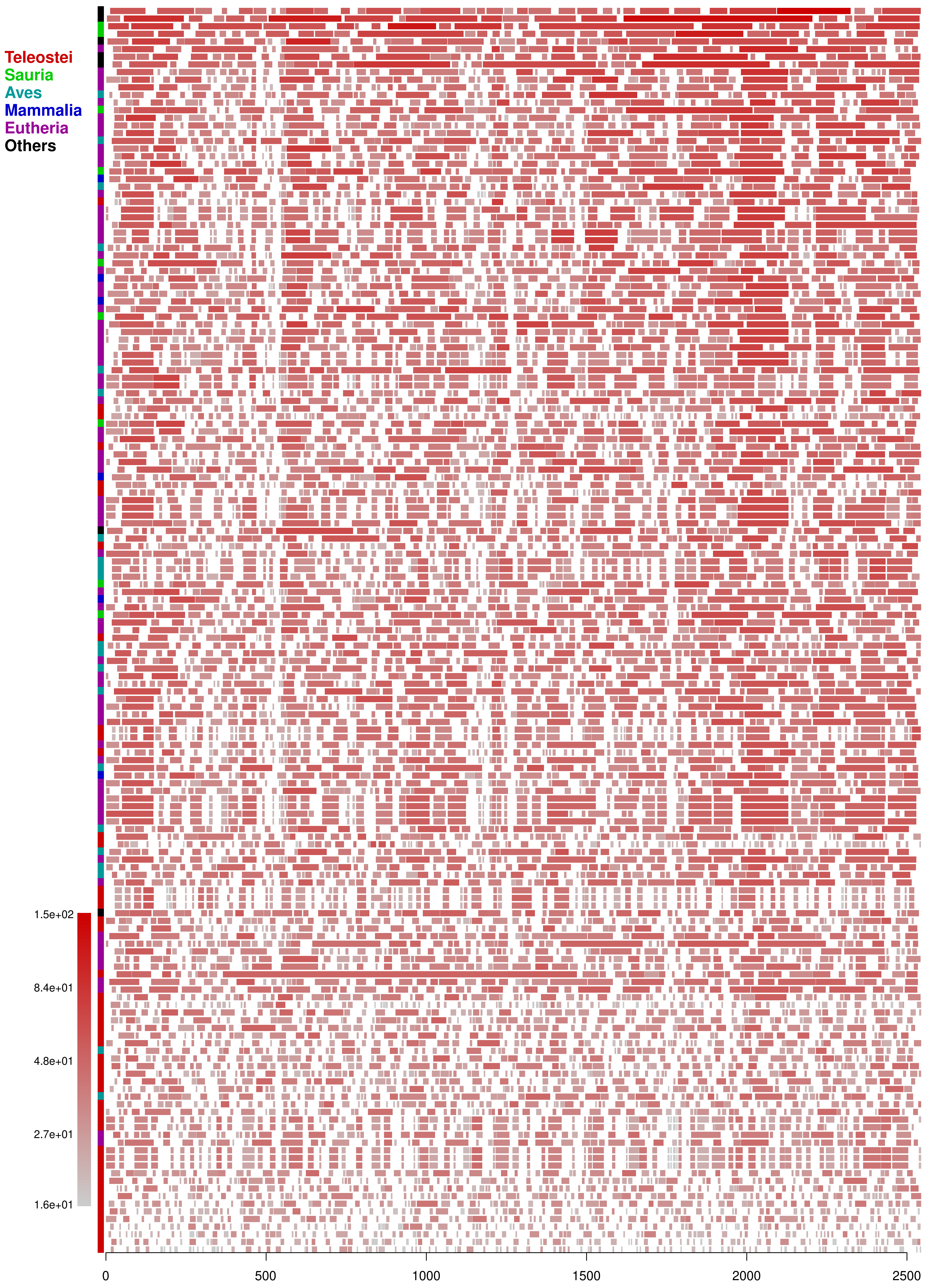

0 alignments above max size (1.0e+08)

ENSDARG00000003069 : ENSDART00000149024 intron no: 6 length: 1466

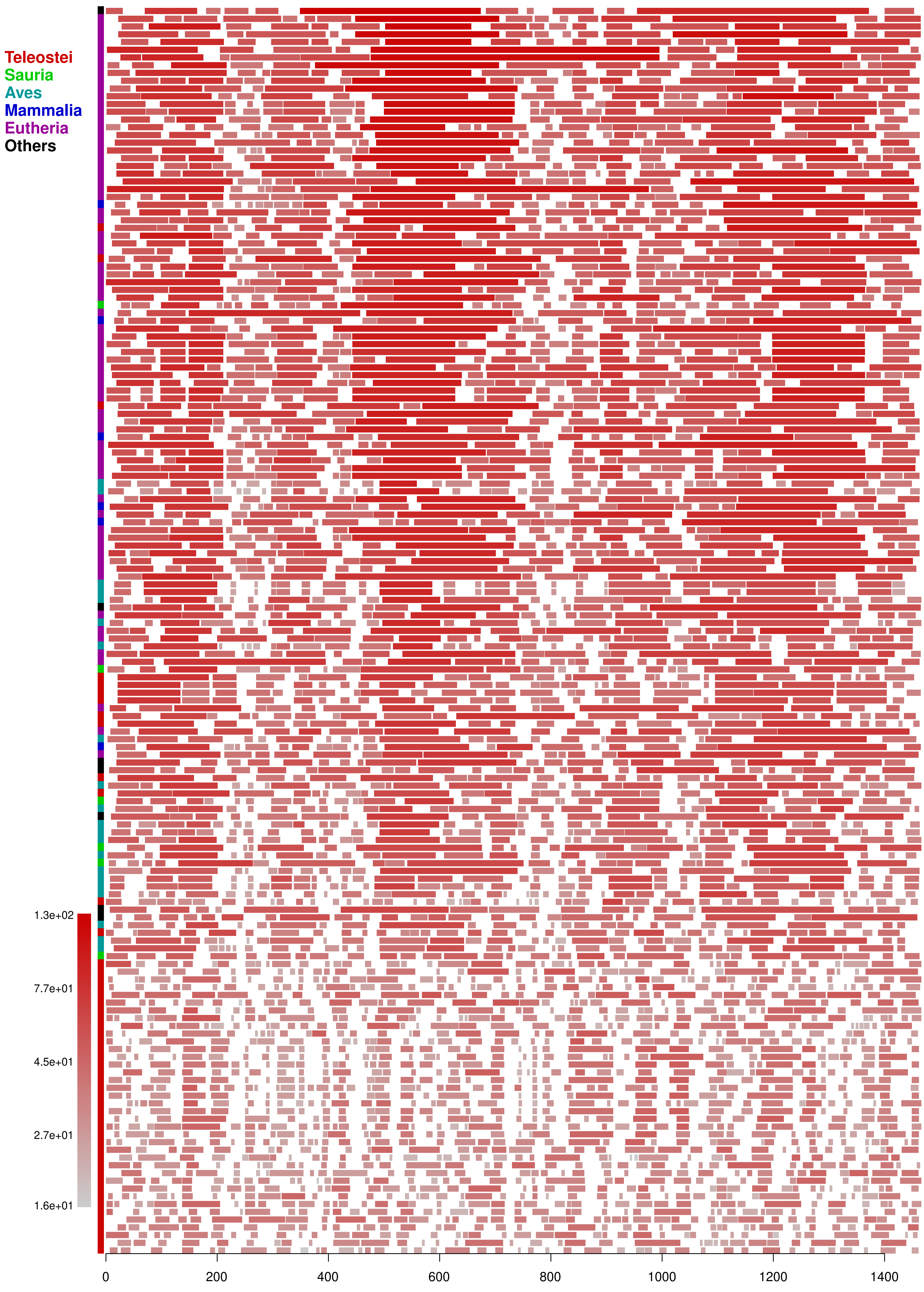

0 alignments above max size (1.0e+08)

ENSDARG00000074331 : ENSDART00000145172 intron no: 30 length: 2418

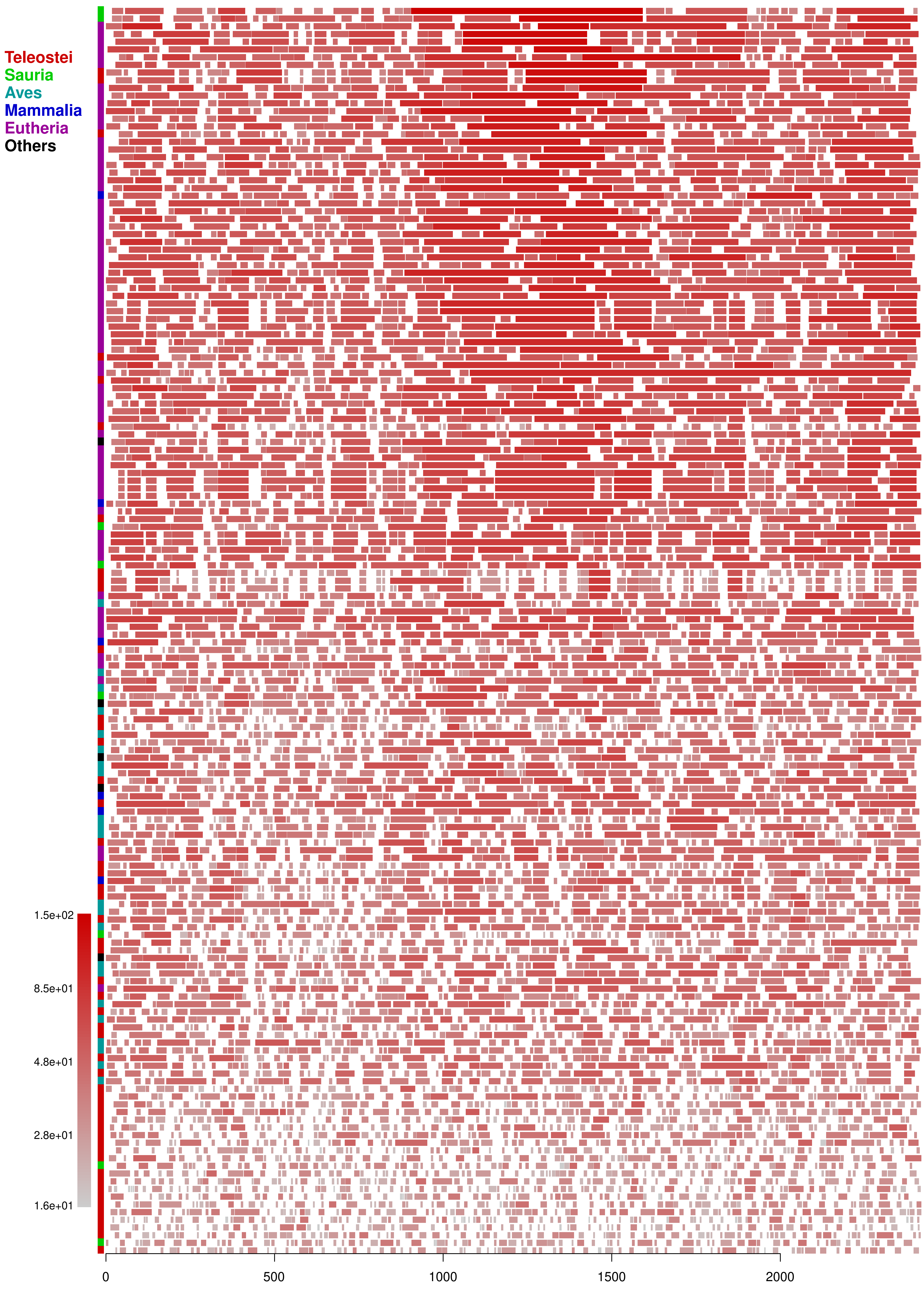

1 alignments above max size (1.0e+08)

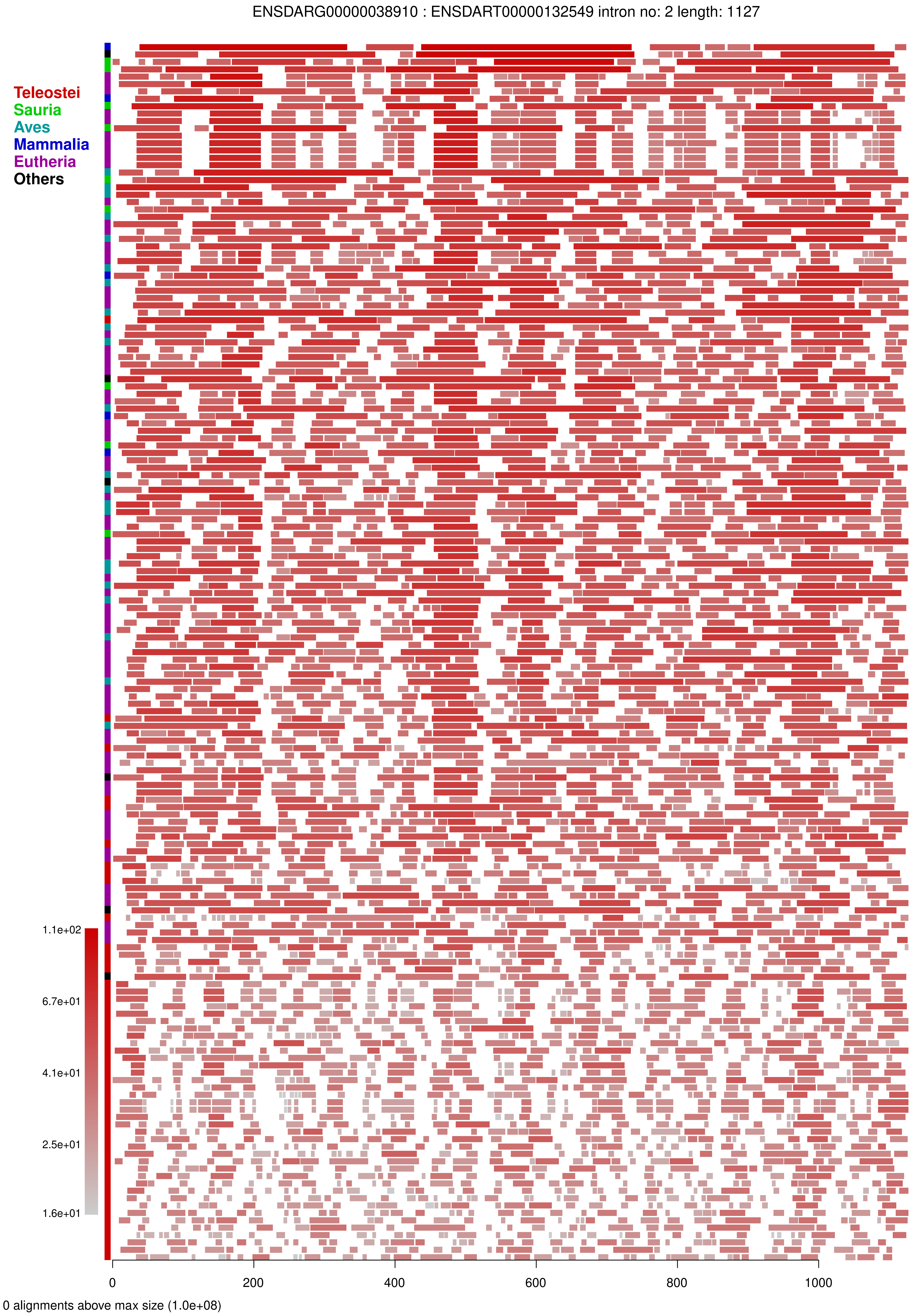

ENSDARG00000038910 : ENSDART00000132549 intron no: 2 length: 1127
Teleostei
Sauria
Aves
Mammalia
Eutheria
Others
1.1e+02
6.7e+01
4.1e+01
2.5e+01
1.6e+01
0
200
400
600
800
1000
0 alignments above max size (1.0e+08)

ENSDARG00000003984 : ENSDART00000022170 intron no: 15 length: 1166

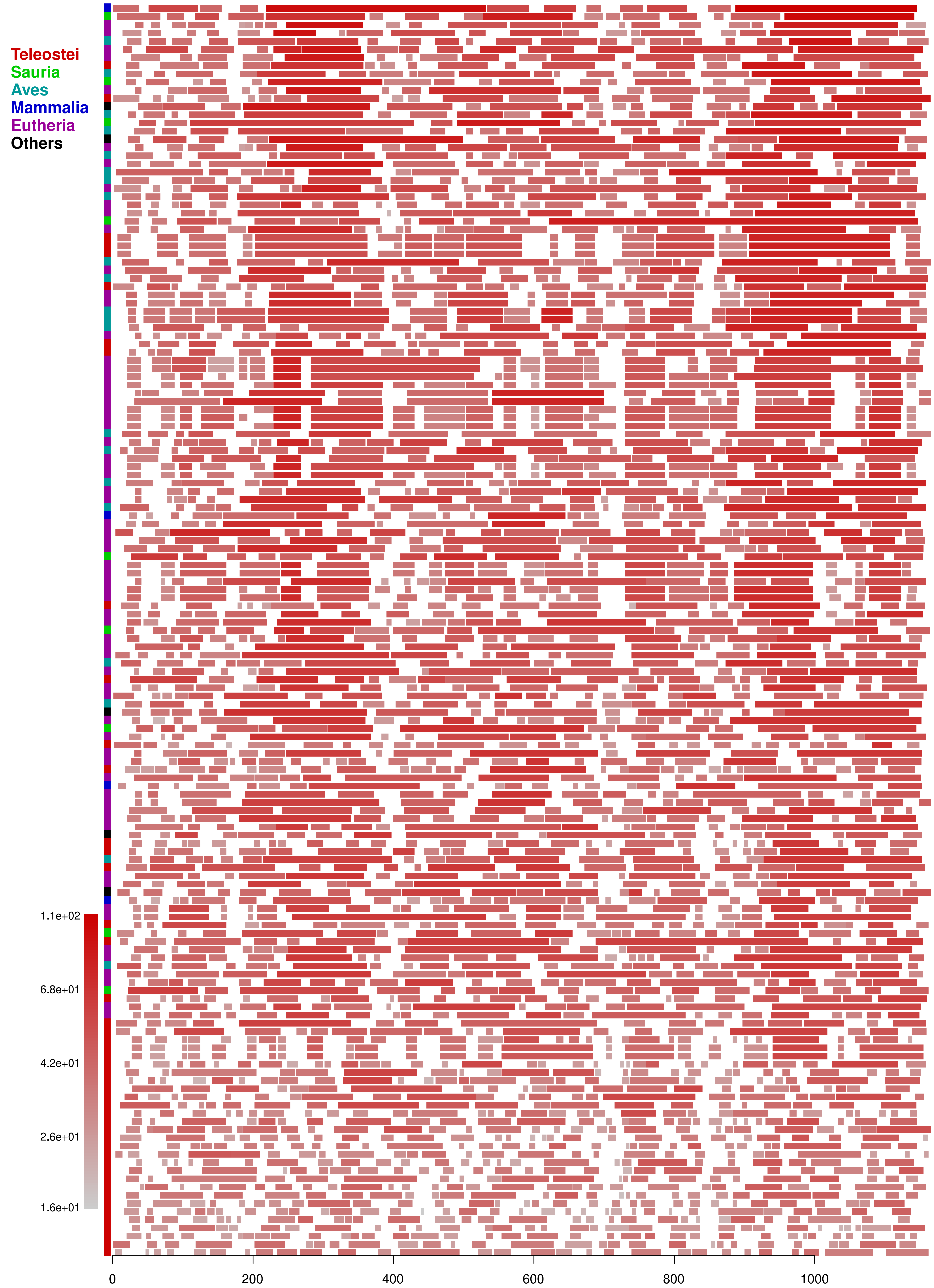

1 alignments above max size (1.0e+08)

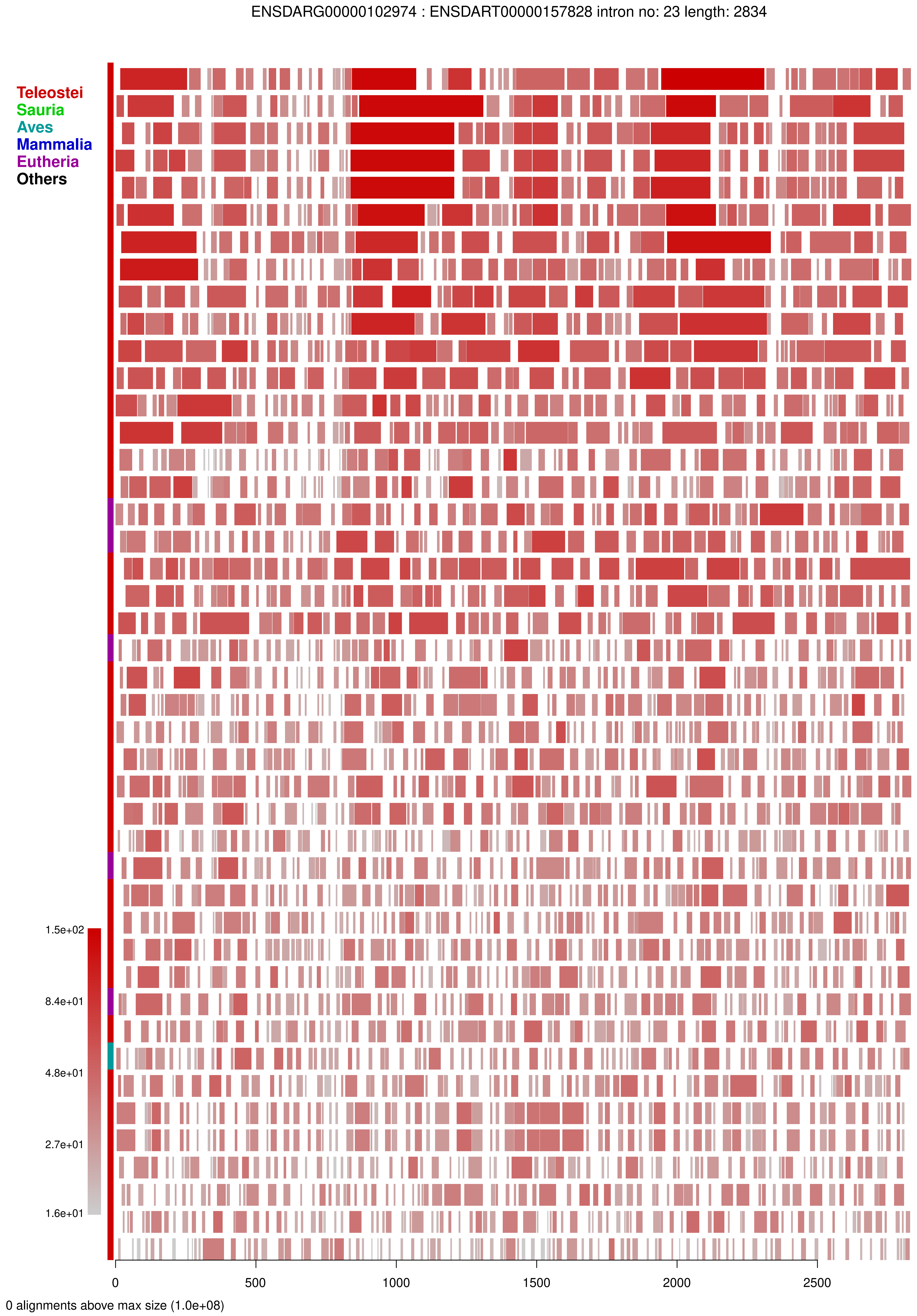

ENSDARG00000102974 : ENSDART00000157828 intron no: 23 length: 2834
Teleostei
Sauria
Aves
Mammalia
Eutheria
Others
1.5e+02
8.4e+01
4.8e+01
2.7e+01
1.6e+01
0
500
1000
1500
2000
2500
0 alignments above max size (1.0e+08)

ENSDARG00000104789 : ENSDART00000169624 intron no: 3 length: 2802

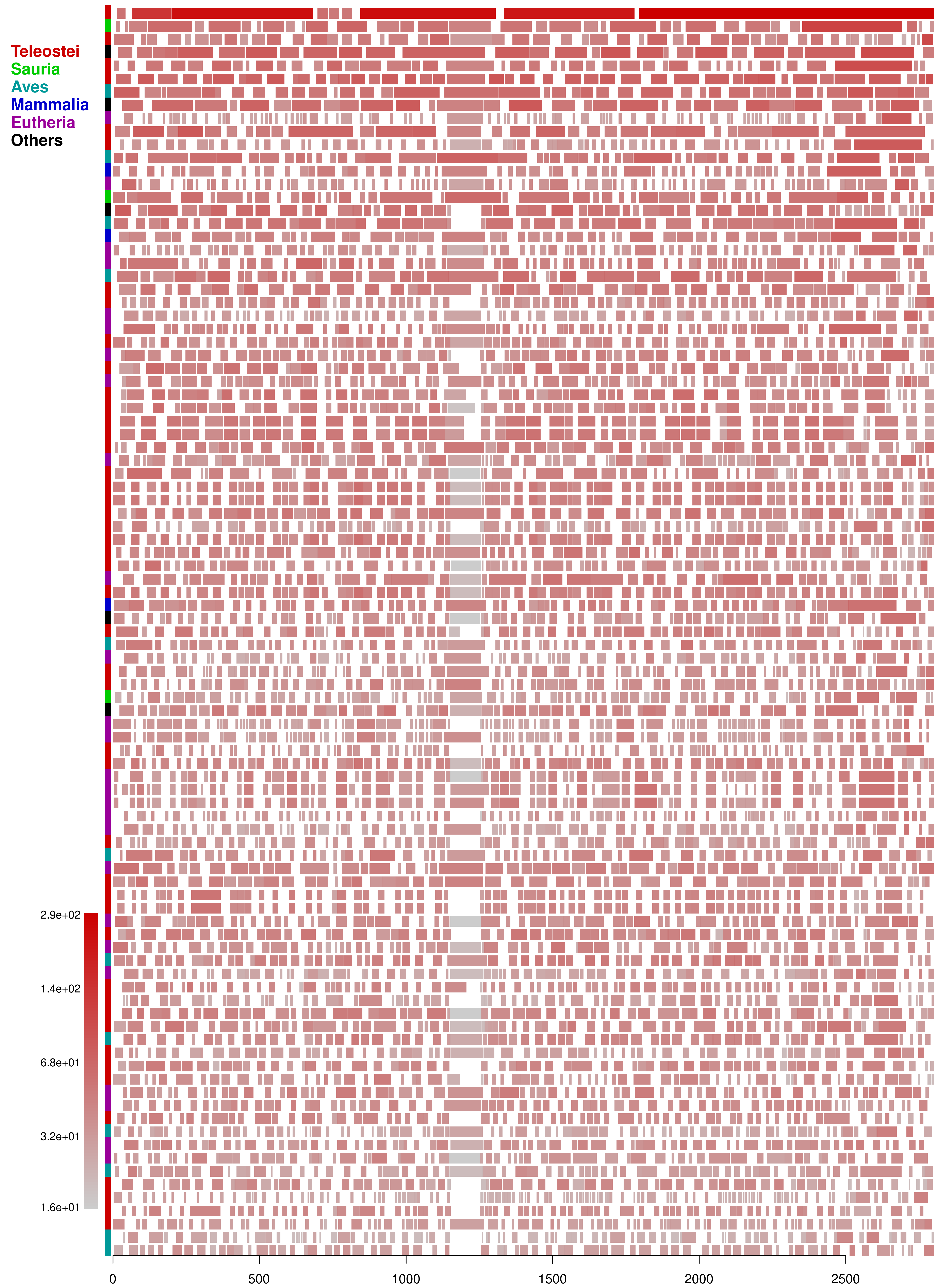

0 alignments above max size (1.0e+08)

ENSDARG00000075217 : ENSDART00000191647 intron no: 15 length: 1414

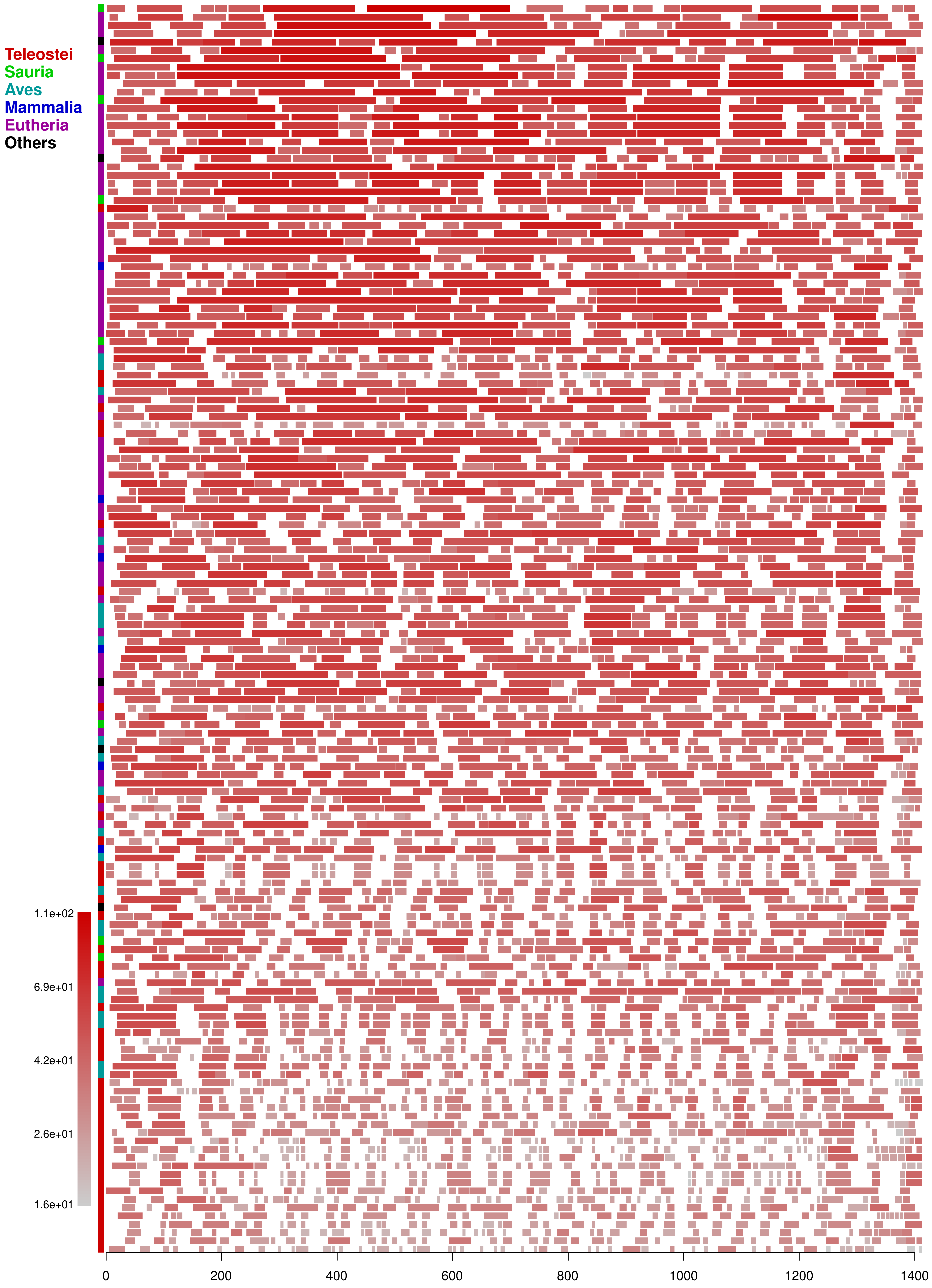

0 alignments above max size (1.0e+08)

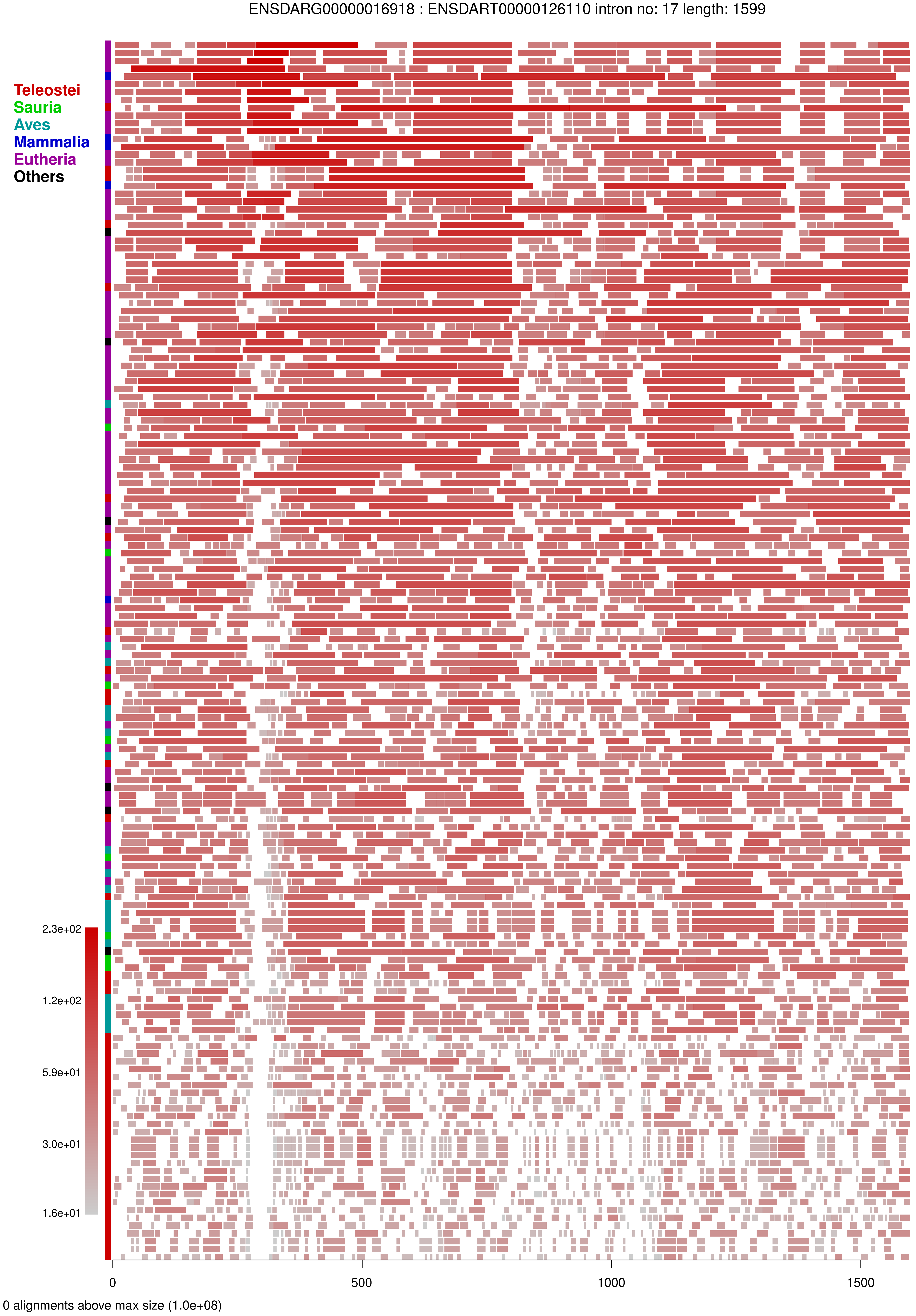

ENSDARG00000016918 : ENSDART00000126110 intron no: 17 length: 1599
Teleostei
Sauria
Aves
Mammalia
Eutheria
Others
2.3e+02
1.2e+02
5.9e+01
3.0e+01
1.6e+01
0
500
1000
1500
0 alignments above max size (1.0e+08)

ENSDARG00000070958 : ENSDART00000104613 intron no: 7 length: 2201

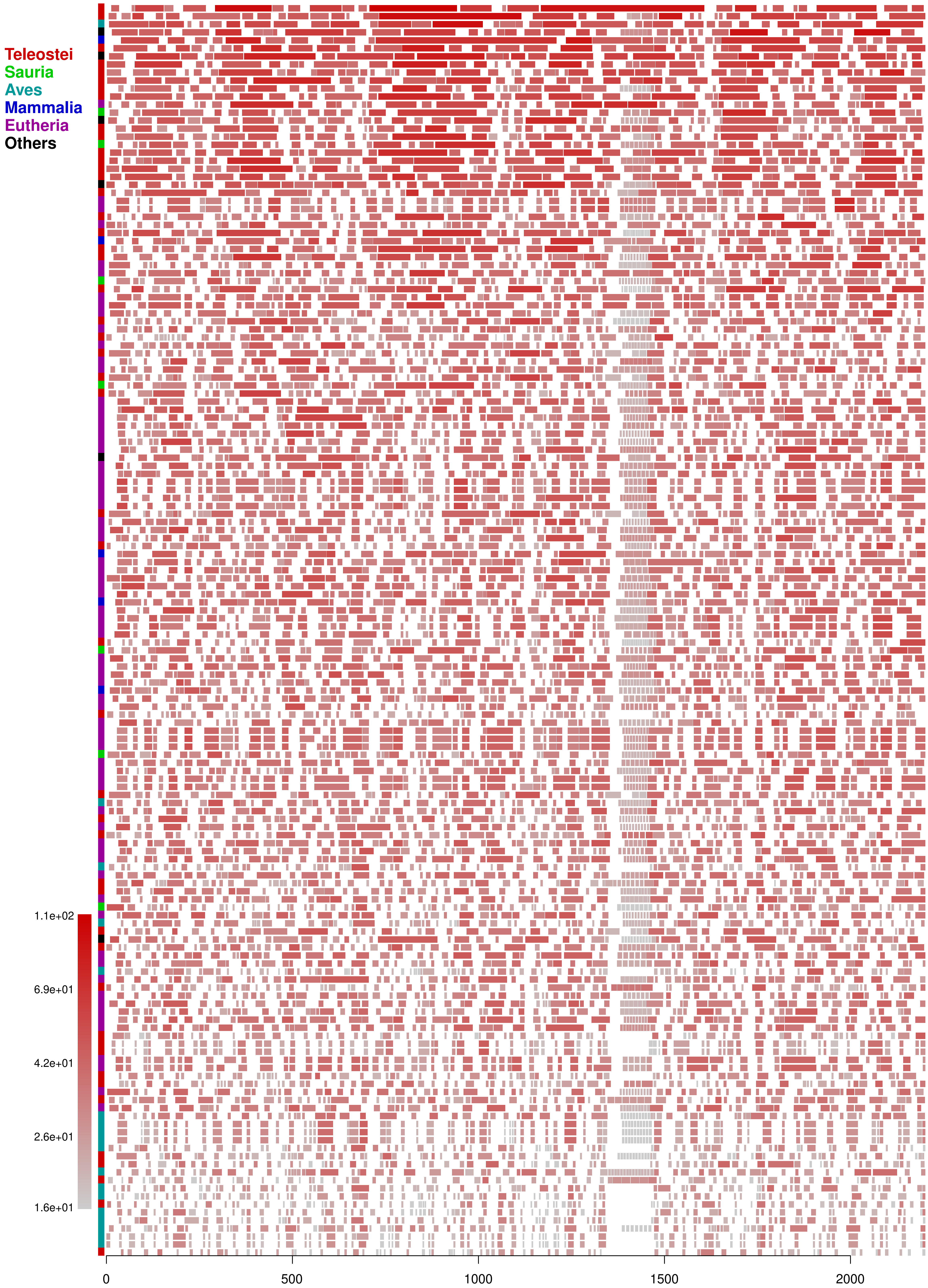

0 alignments above max size (1.0e+08)

ENSDARG00000075824 : ENSDART00000159064 intron no: 22 length: 2836

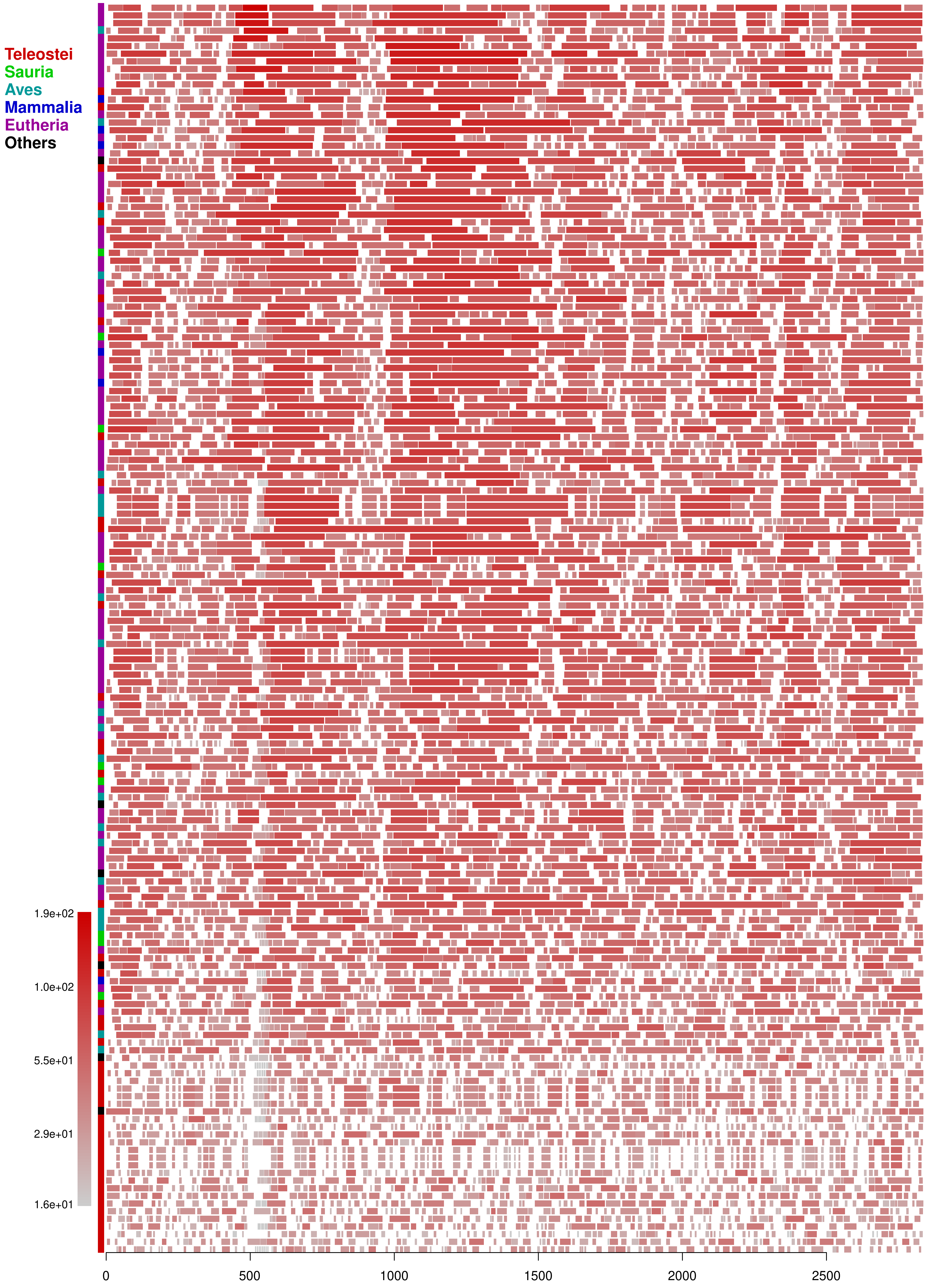

0 alignments above max size (1.0e+08)

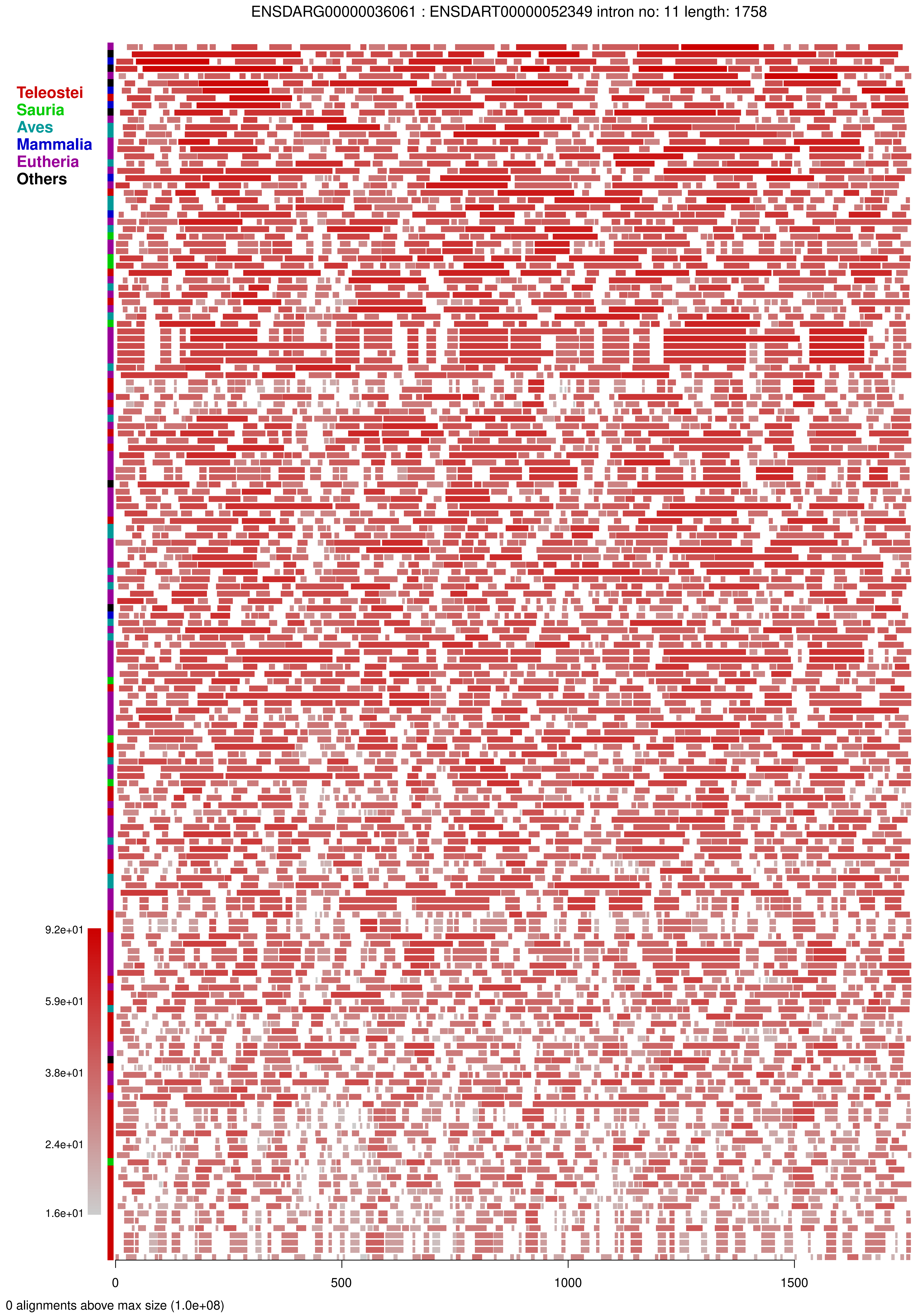

ENSDARG00000036061 : ENSDART00000052349 intron no: 11 length: 1758
Teleostei
Sauria
Aves
Mammalia
Eutheria
Others
9.2e+01
5.9e+01
3.8e+01
2.4e+01
1.6e+01
0
500
1000
1500
0 alignments above max size (1.0e+08)

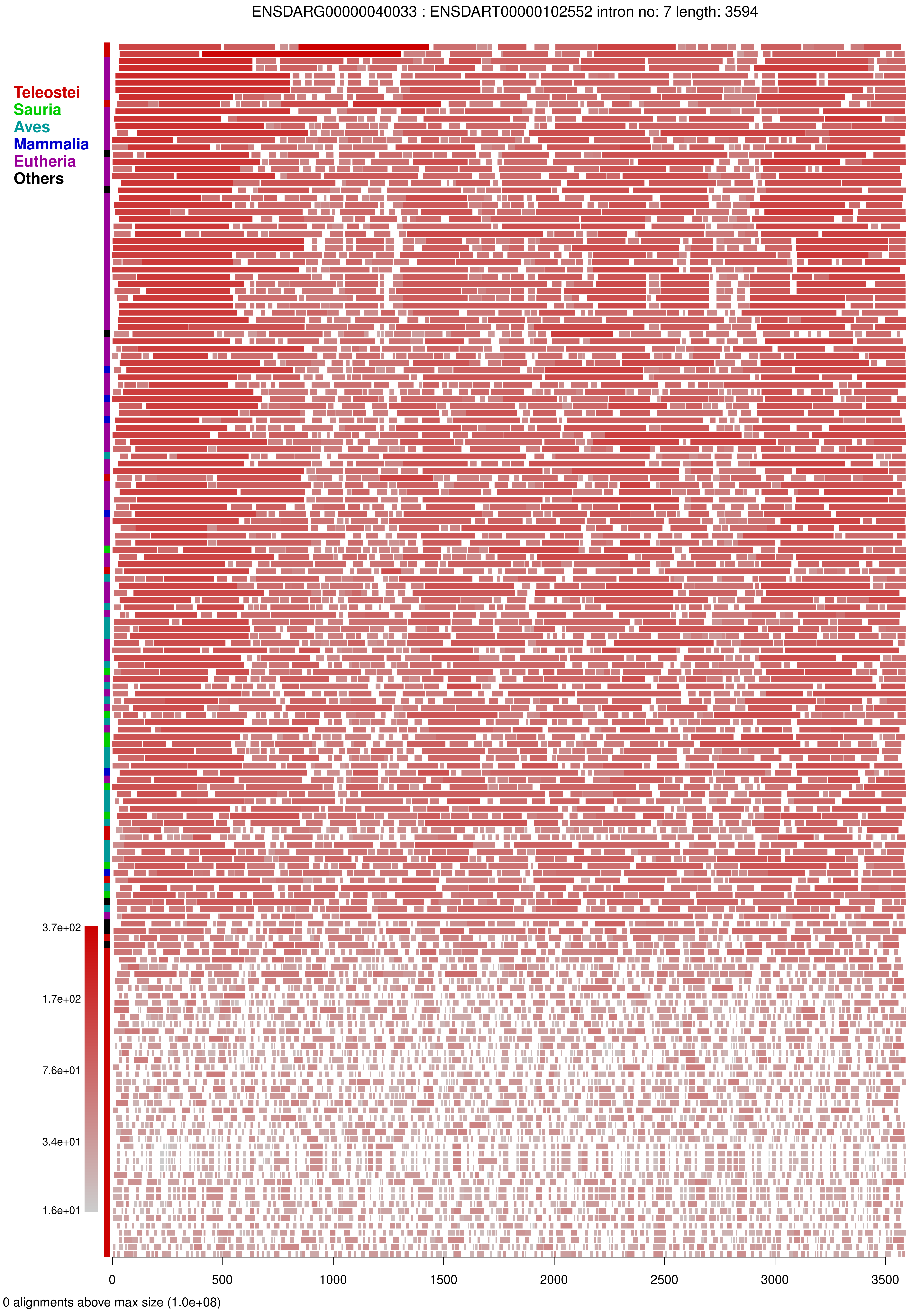
ENSDARG00000040033 : ENSDART00000102552 intron no: 7 length: 3594
Teleostei
Sauria
Aves
Mammalia
Eutheria
Others
3.7e+02
1.7e+02
7.6e+01
3.4e+01
1.6e+01
0
500
1000
1500
2000
2500
3000
3500
0 alignments above max size (1.0e+08)

ENSDARG00000040255 : ENSDART00000058890 intron no: 14 length: 4592

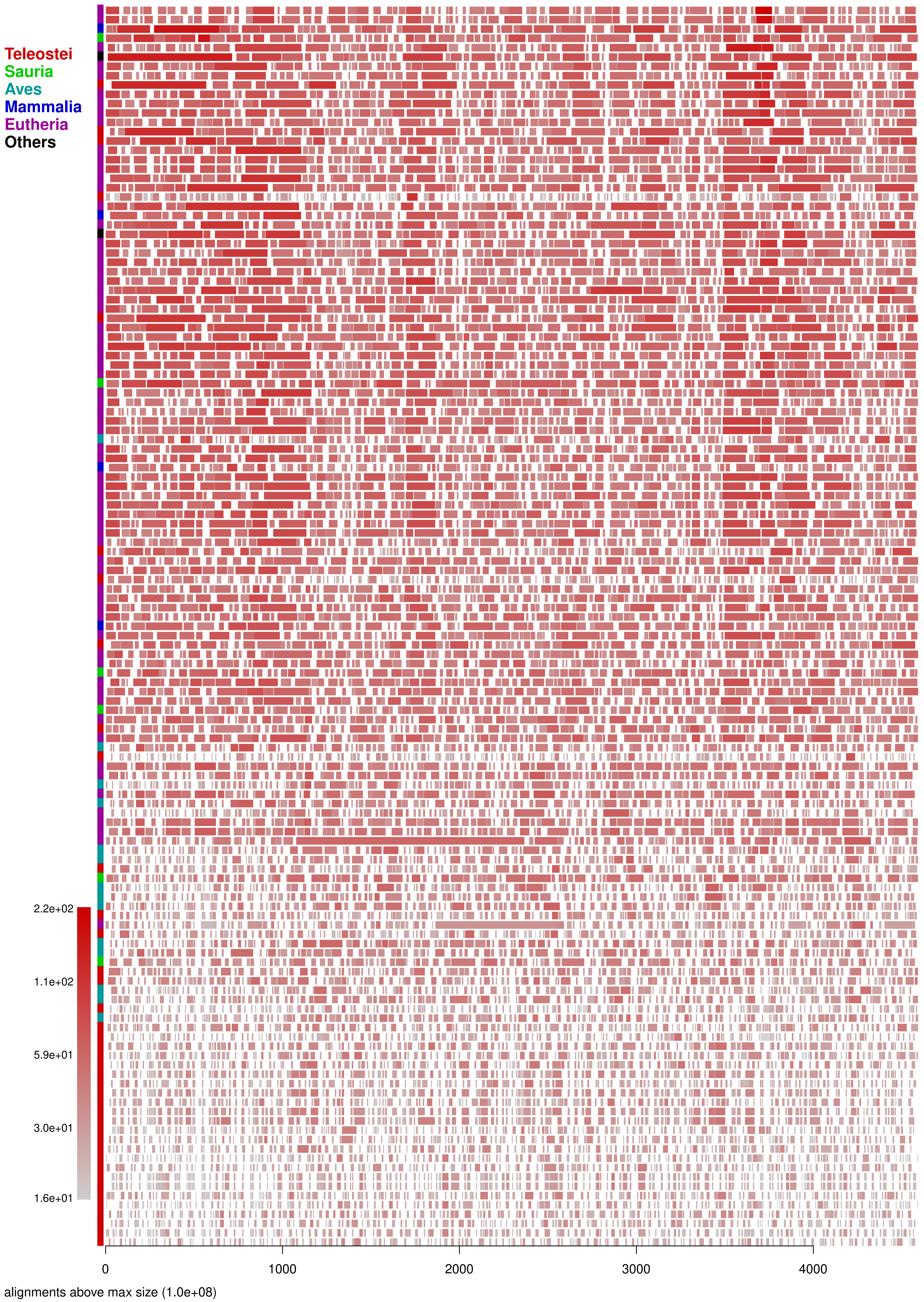

0 alignments above max size (1.0e+08)

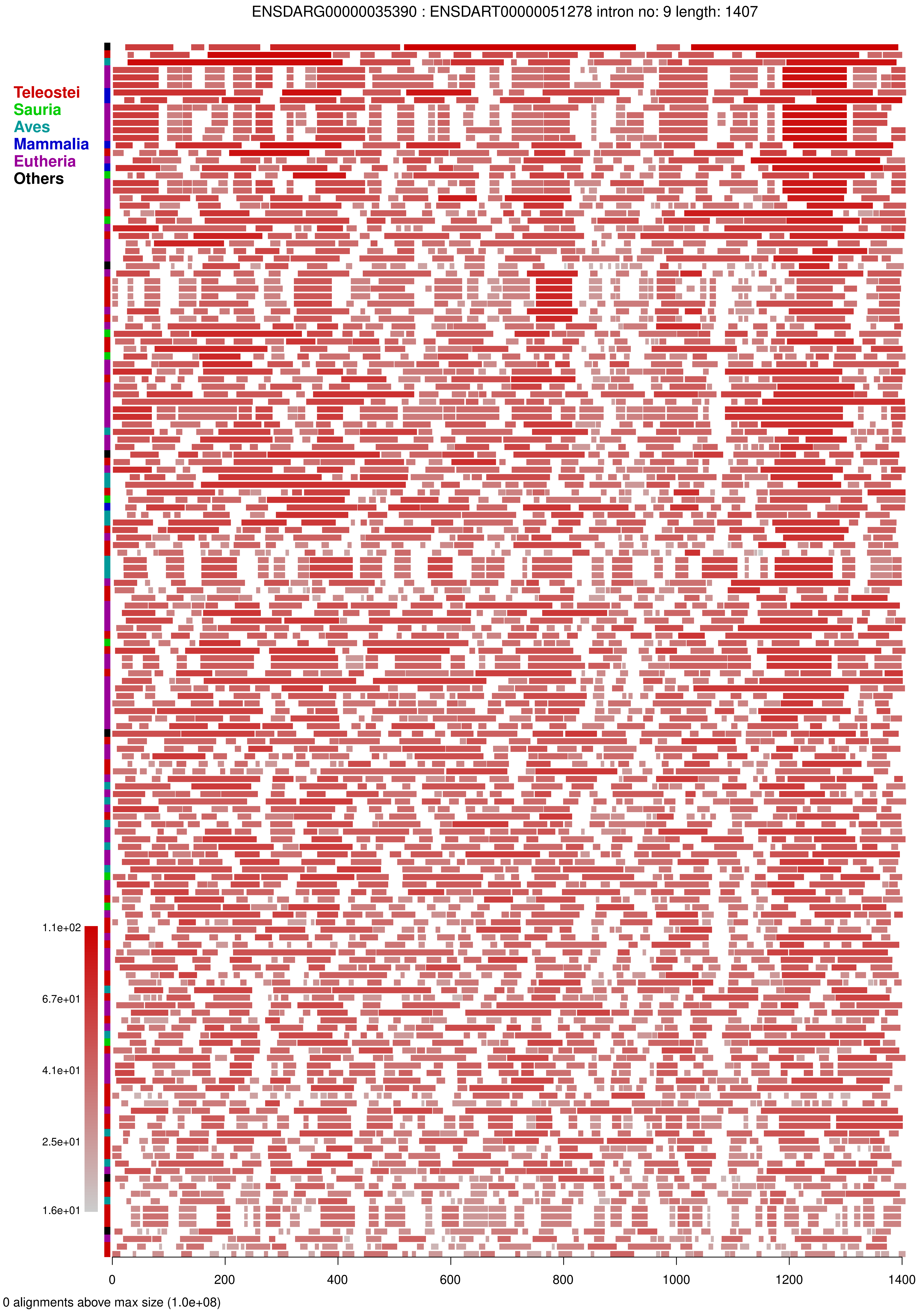

ENSDARG00000035390 : ENSDART00000051278 intron no: 9 length: 1407
Teleostei
Sauria
Aves
Mammalia
Eutheria
Others
1.1e+02
6.7e+01
4.1e+01
2.5e+01
1.6e+01
0
200
400
600
800
1000
1200
1400
0 alignments above max size (1.0e+08)

ENSDARG00000016706 : ENSDART00000022307 intron no: 7 length: 1771

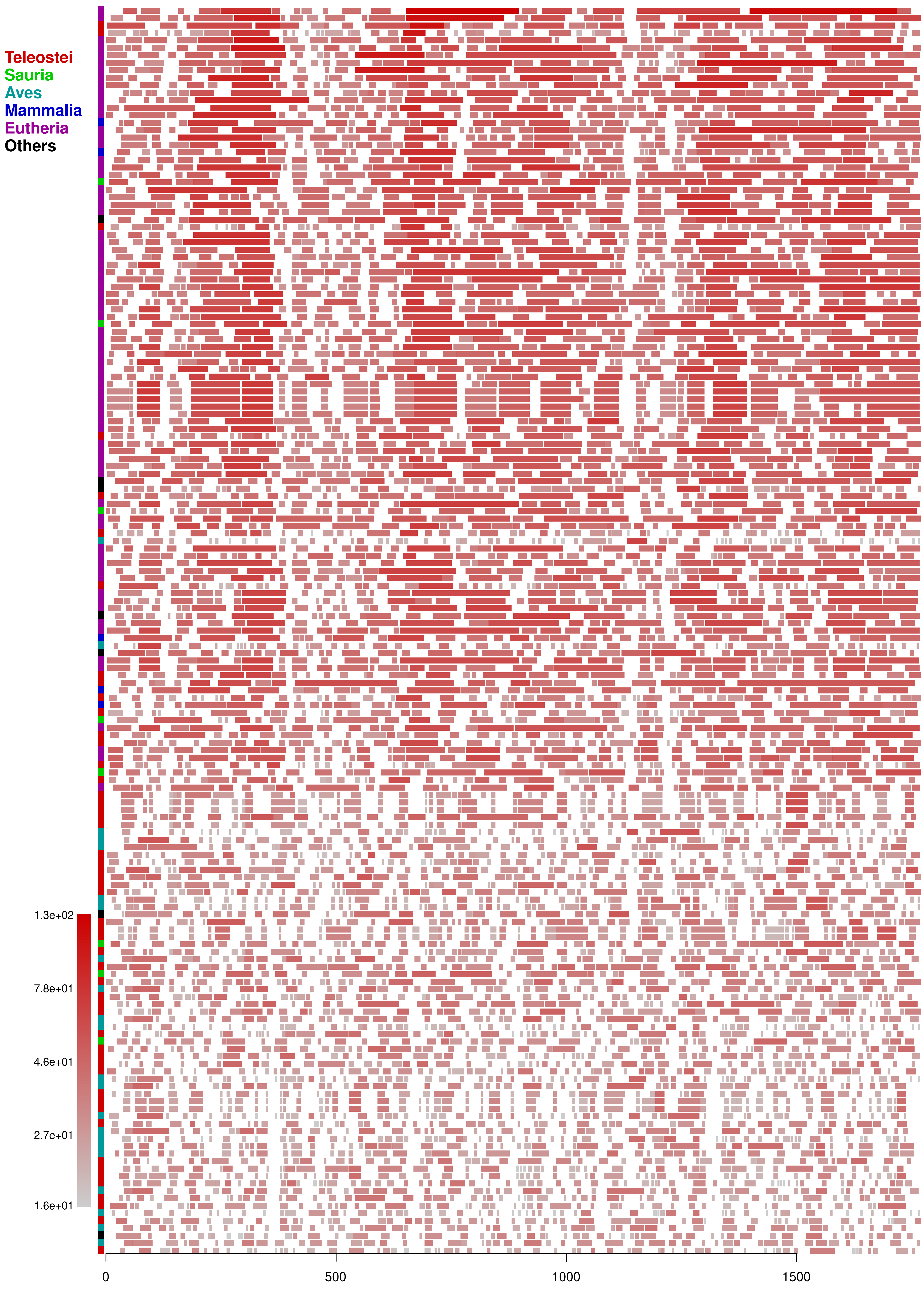

0 alignments above max size (1.0e+08)

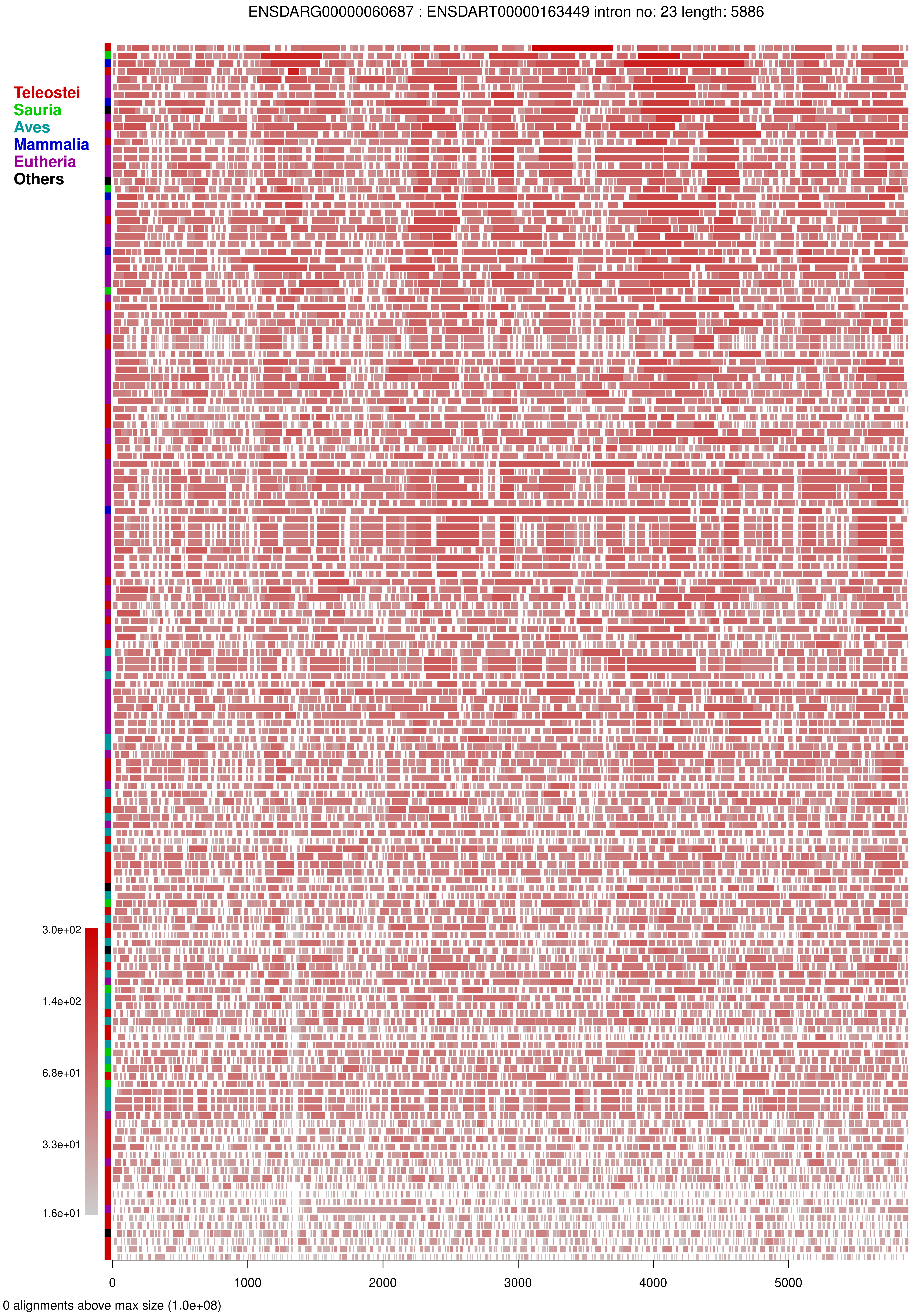

ENSDARG00000060687 : ENSDART00000163449 intron no: 23 length: 5886
Teleostei
Sauria
Aves
Mammalia
Eutheria
Others
3.0e+02
1.4e+02
6.8e+01
3.3e+01
1.6e+01
0
1000
2000
3000
4000
5000
0 alignments above max size (1.0e+08)

ENSDARG00000079024 : ENSDART00000156806 intron no: 7 length: 1049

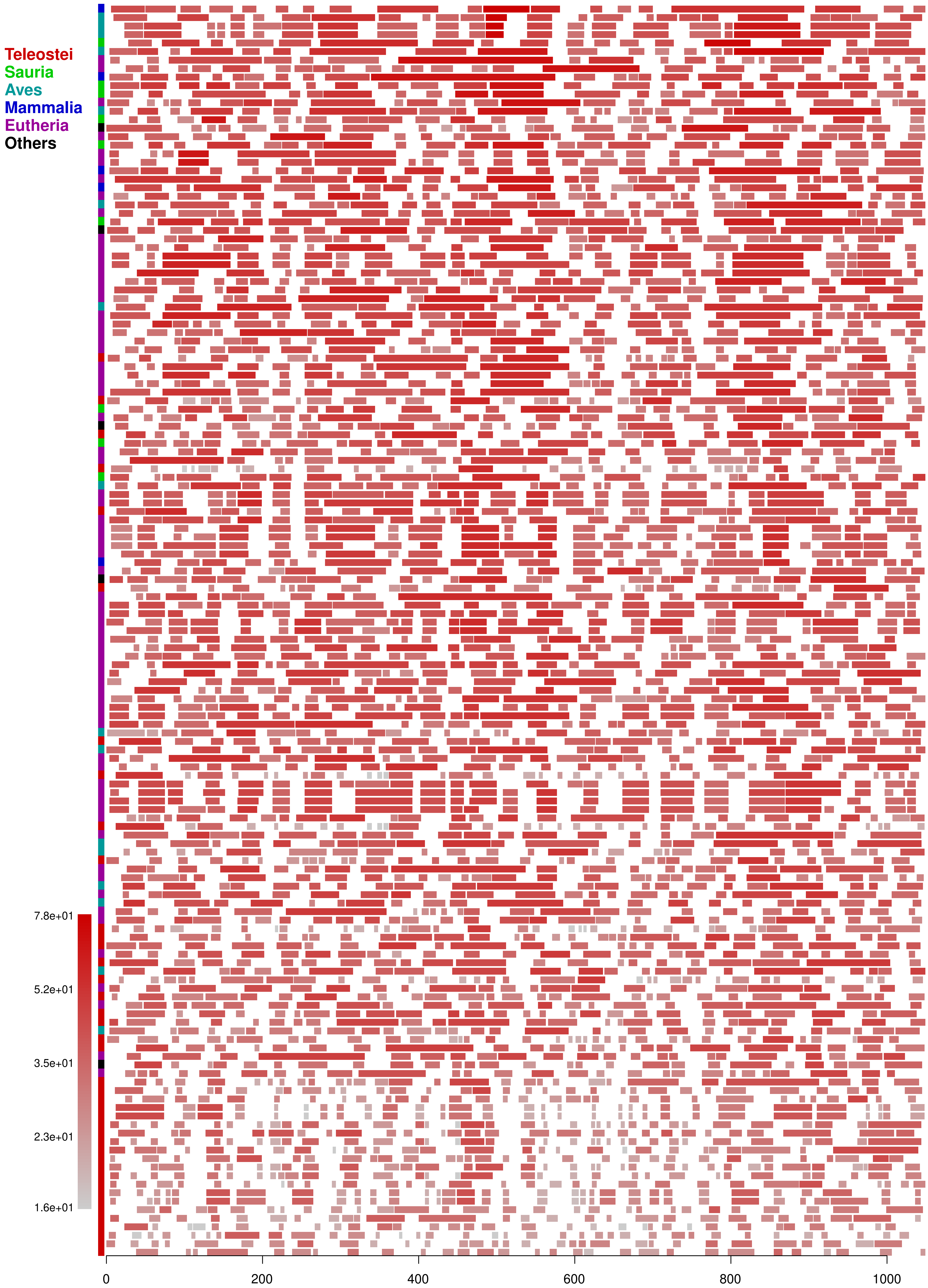

0 alignments above max size (1.0e+08)

ENSDARG00000069271 : ENSDART00000100668 intron no: 4 length: 4668

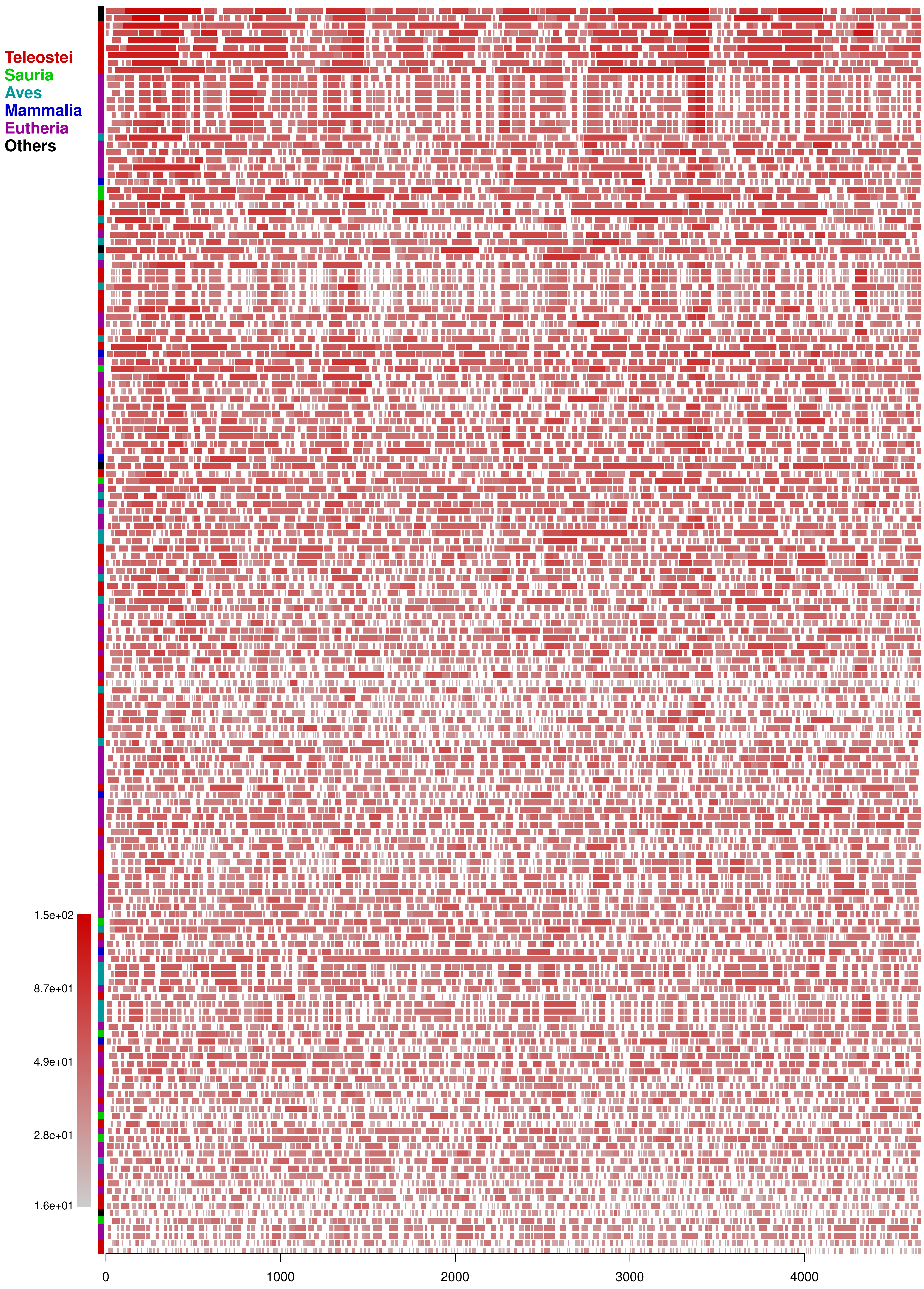

0 alignments above max size (1.0e+08)
