# Supplementary material for: Intron size minimisation in teleosts
Source: BMC Genomics. 2022 Sep 1;23:628. doi: 10.1186/s12864-022-08760-w (PMC9438311; doi:10.1186/s12864-022-08760-w)

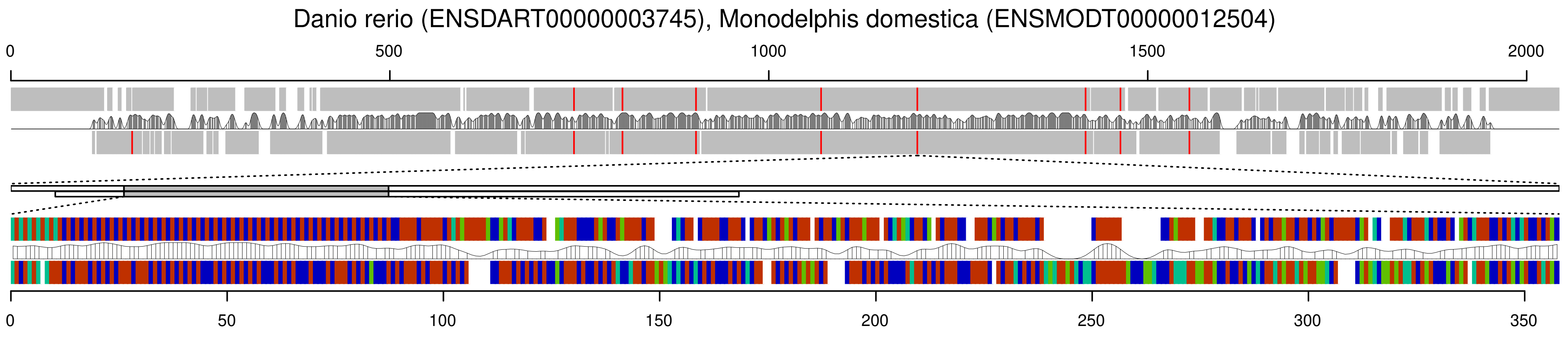
Danio rerio (ENSDART00000003745), Monodelphis domestica (ENSMODT00000012504)
0
500
1000
1500
2000
0
50
100
150
200
250
300
350

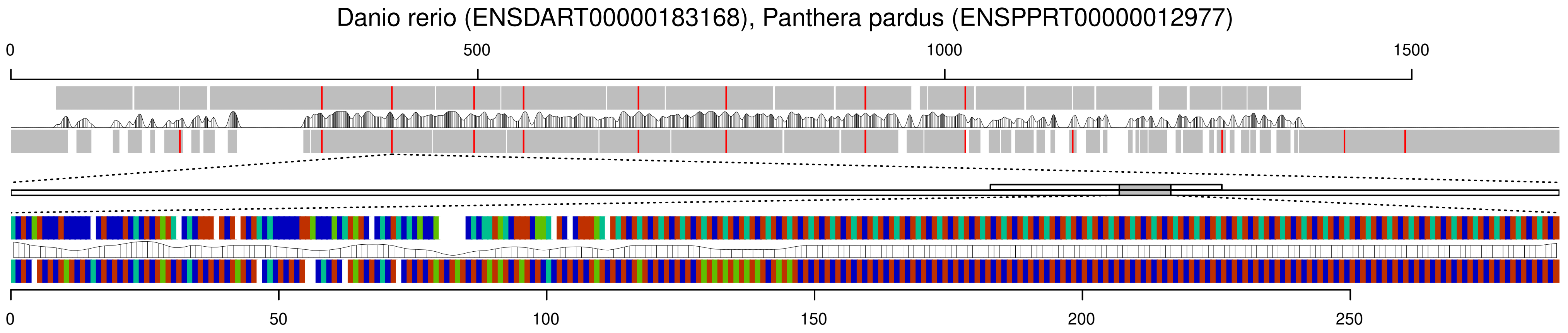
Danio rerio (ENSDART00000183168), Panthera pardus (ENSPPRT00000012977)
0
500
1000
1500
0
50
100
150
200
250

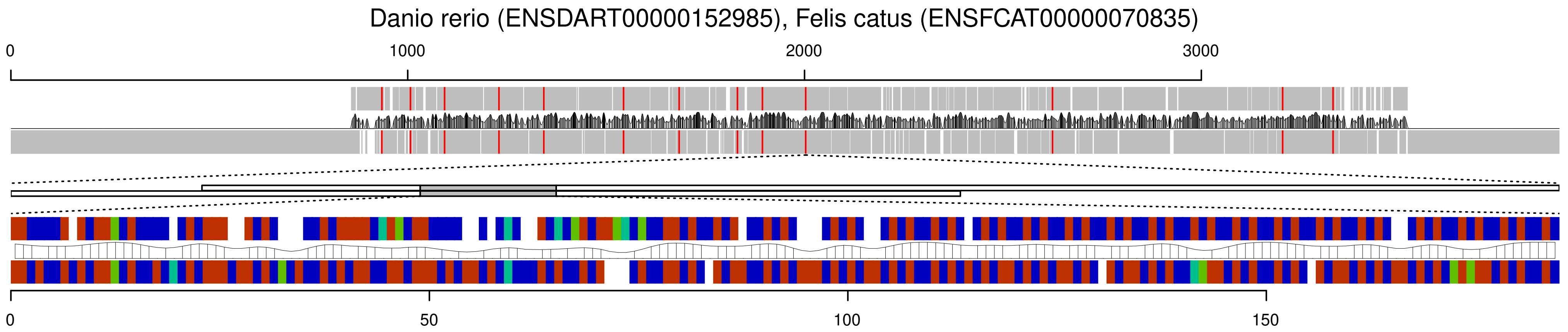
Danio rerio (ENSDART00000152985), Felis catus (ENSFCAT00000070835)
0
1000
2000
3000
0
50
100
150

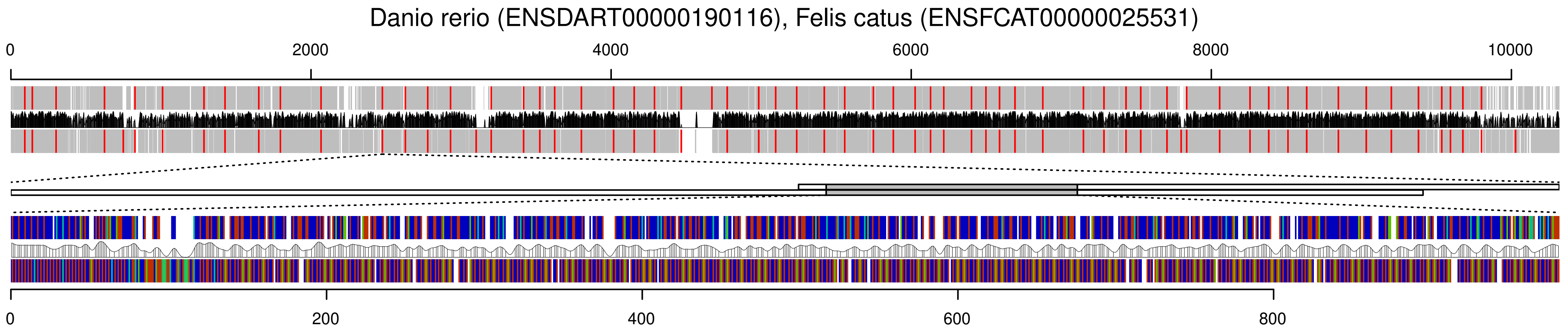
Danio rerio (ENSDART00000190116), Felis catus (ENSFCAT00000025531)
0
2000
4000
6000
8000
10000
0
200
400
600
800

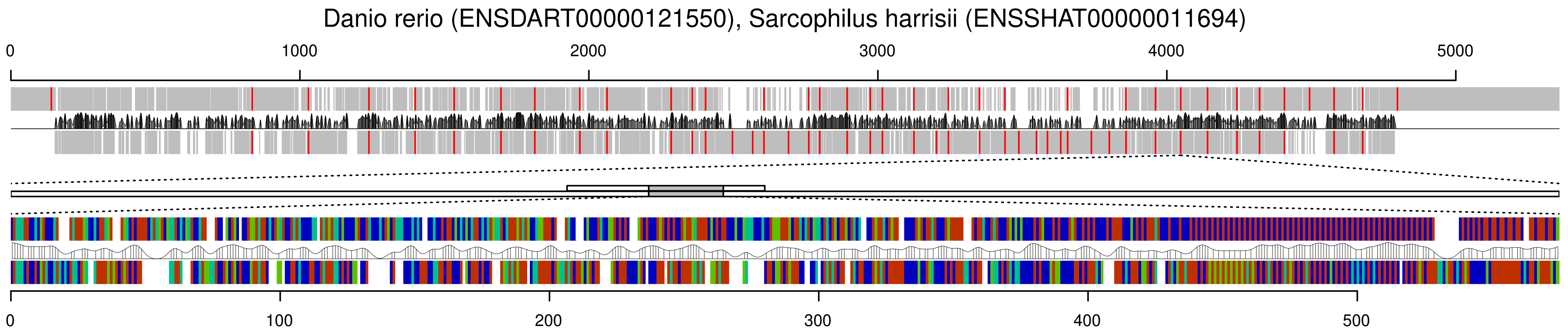
Danio rerio (ENSDART00000121550), Sarcophilus harrisii (ENSSHAT00000011694)
0
1000
2000
3000
4000
5000
0
100
200
300
400
500

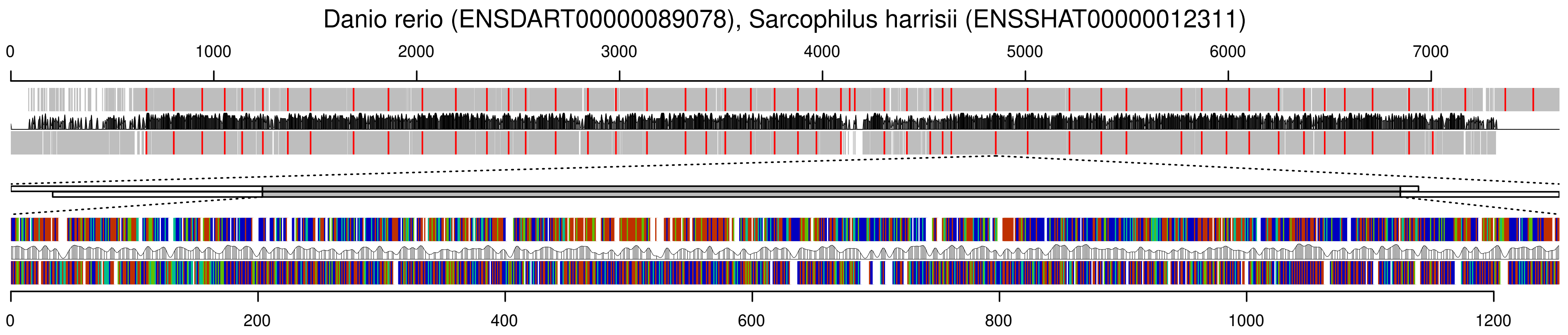
Danio rerio (ENSDART00000089078), Sarcophilus harrisii (ENSSHAT00000012311)
0
1000
2000
3000
4000
5000
6000
7000
0
200
400
600
800
1000
1200

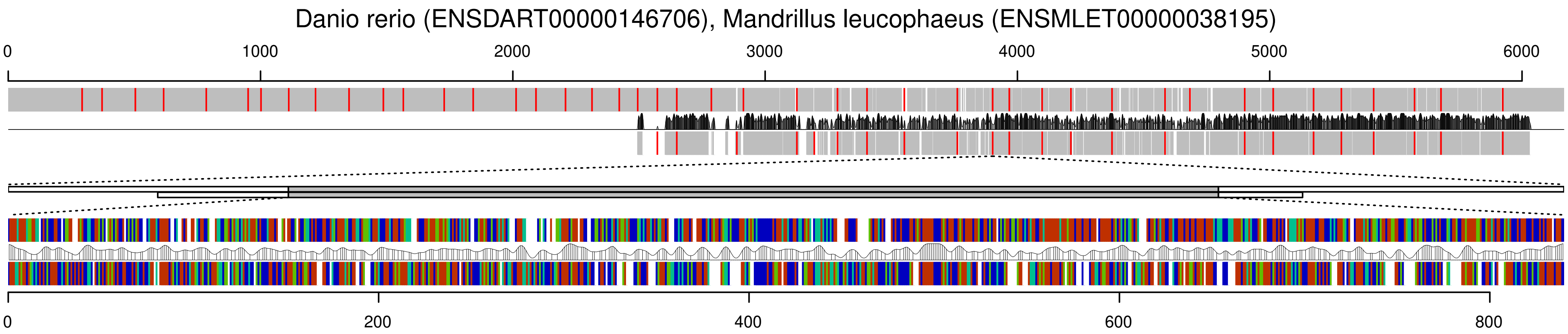
Danio rerio (ENSDART00000146706), Mandrillus leucophaeus (ENSMLET00000038195)
0 1000 2000 3000 4000 5000 6000
0 200 400 600 800

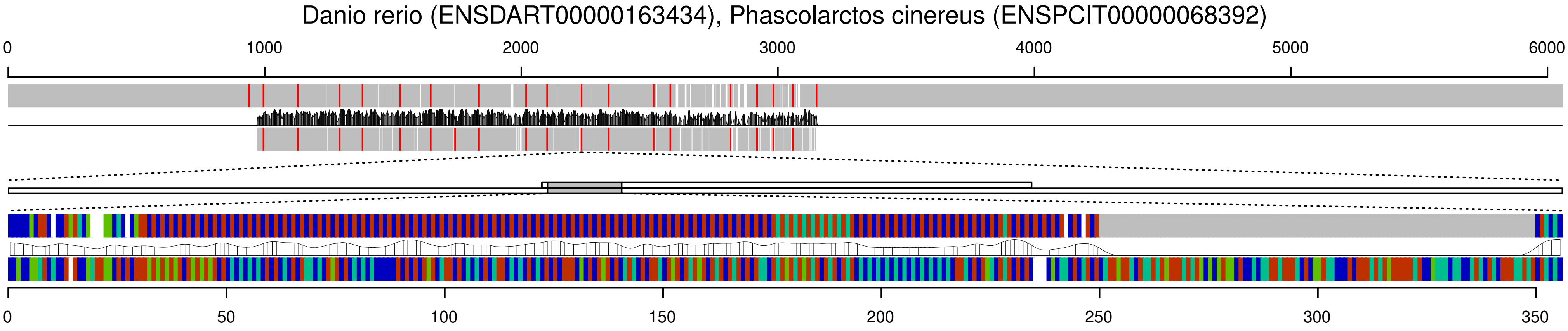
Danio rerio (ENSDART00000163434), Phascolarctos cinereus (ENSPCIT00000068392)
0 1000 2000 3000 4000 5000 6000
0 50 100 150 200 250 300 350

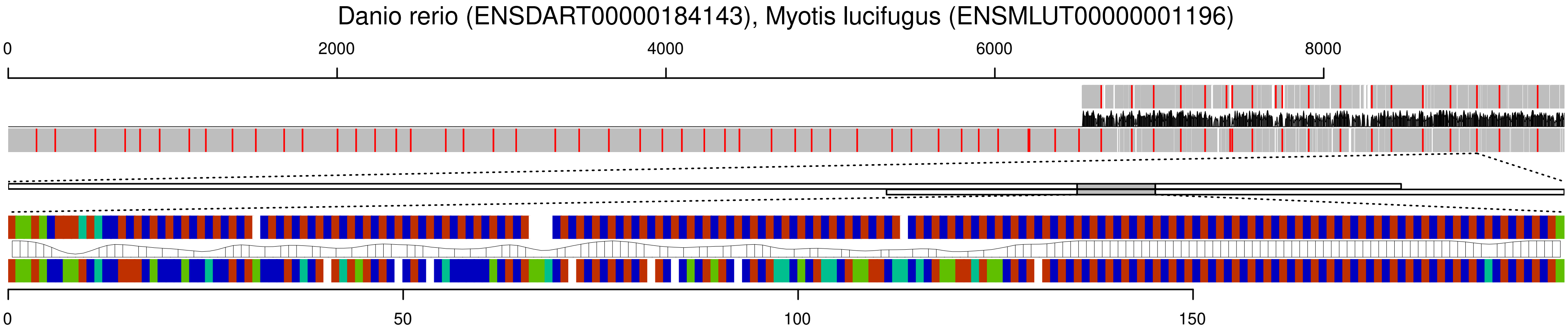
Danio rerio (ENSDART00000184143), Myotis lucifugus (ENSMLUT00000001196)
0 2000 4000 6000 8000
0 50 100 150

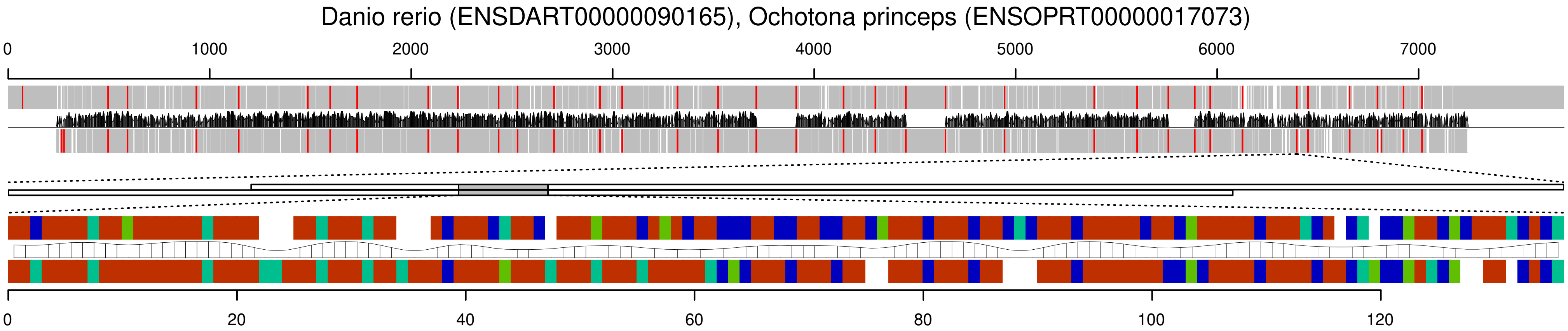
Danio rerio (ENSDART00000090165), Ochotona princeps (ENSOPRT00000017073)
0 1000 2000 3000 4000 5000 6000 7000
0 20 40 60 80 100 120

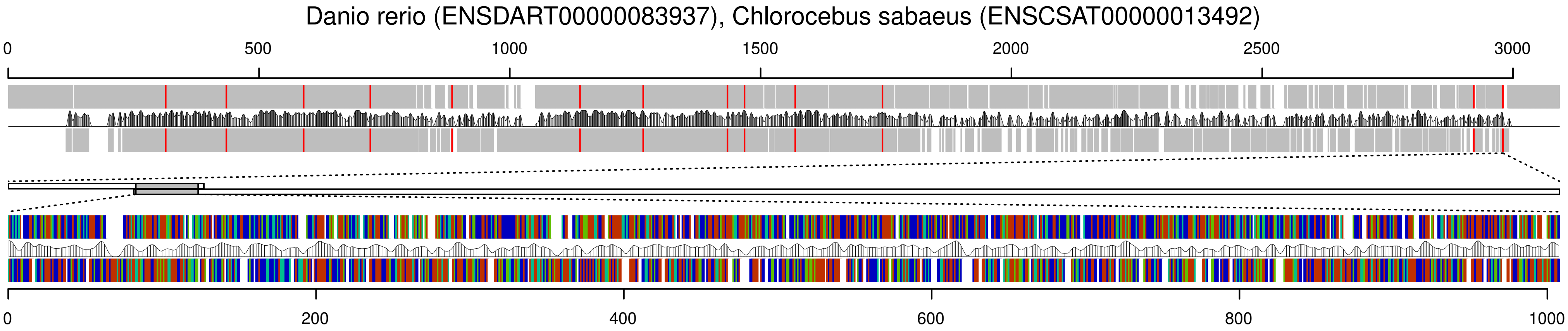
Danio rerio (ENSDART00000083937), Chlorocebus sabaeus (ENSCSAT00000013492)
0 500 1000 1500 2000 2500 3000
0 200 400 600 800 1000

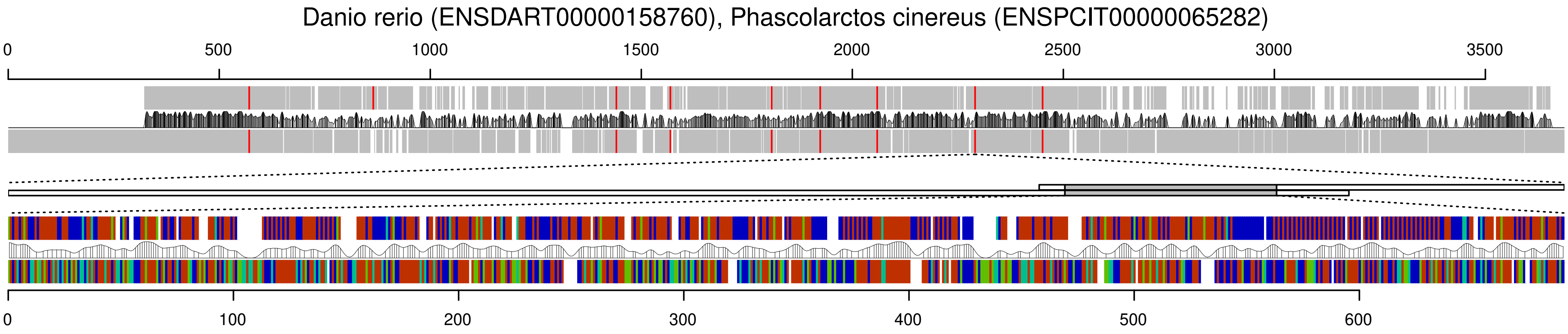
Danio rerio (ENSDART00000158760), Phascolarctos cinereus (ENSPCIT00000065282)
0 500 1000 1500 2000 2500 3000 3500
0 100 200 300 400 500 600

Danio rerio (ENSDART00000184238), Castor canadensis (ENSCCNT00000040843)

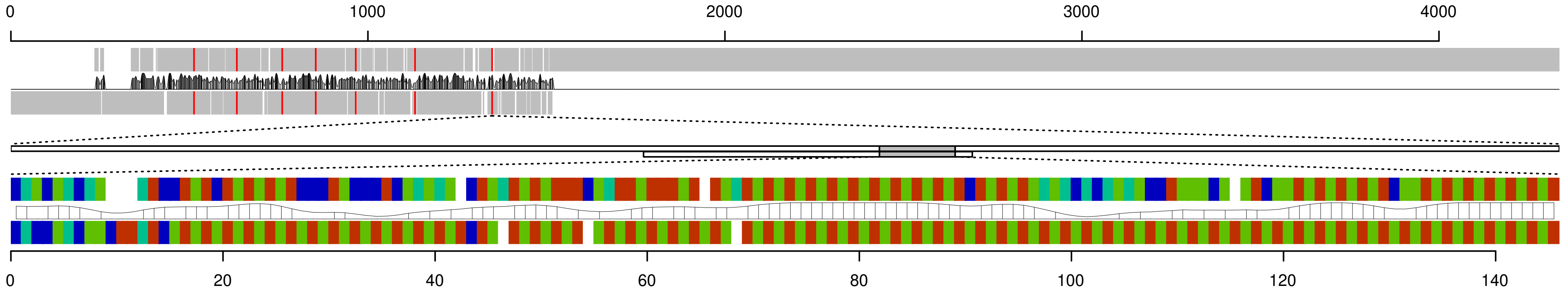

Danio rerio (ENSDART00000035761), Vombatus ursinus (ENSVURT00010010503)

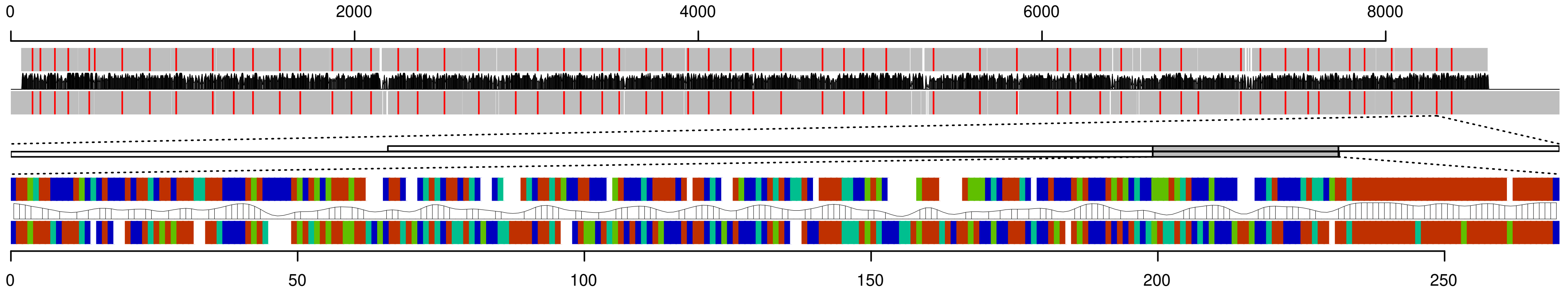

Danio rerio (ENSDART00000115296), Ursus maritimus (ENSUMAT00000037820)

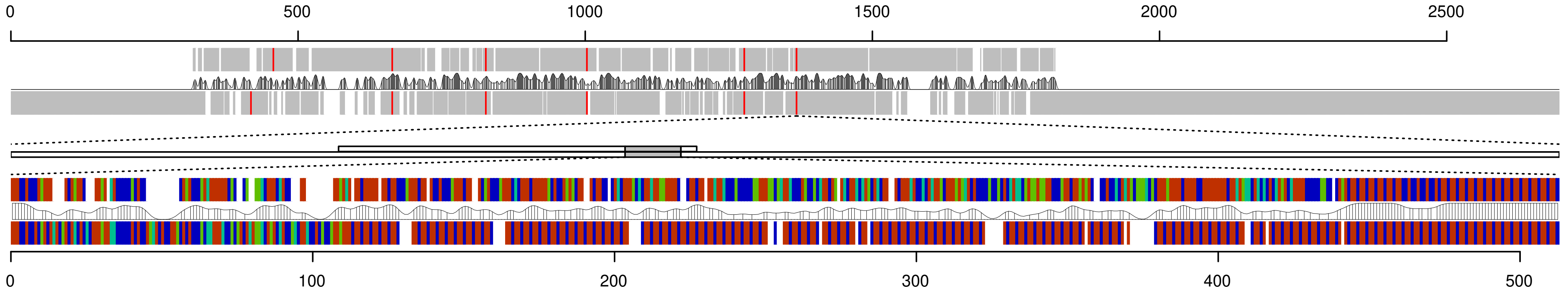

Danio rerio (ENSDART00000113609), Cavia porcellus (ENSCPOT00000033404)

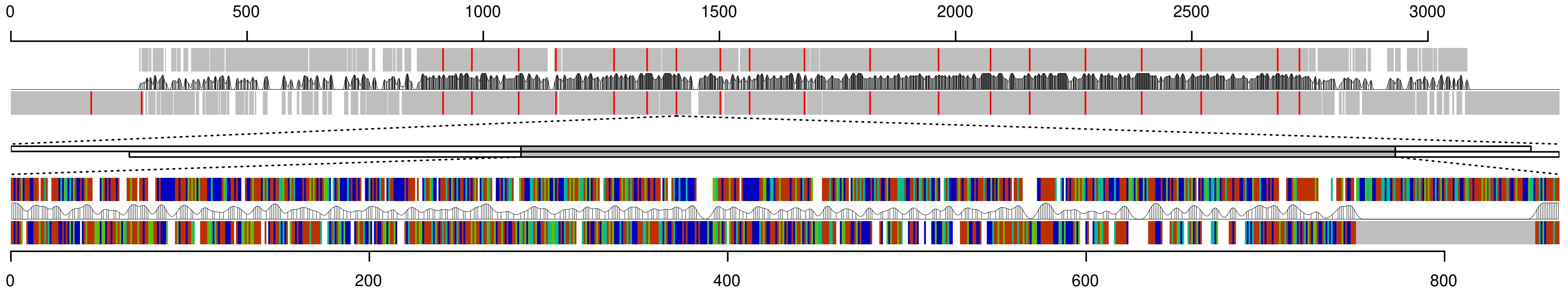

Danio rerio (ENSDART00000192981), Sus scrofa (ENSSSCT00000080217)

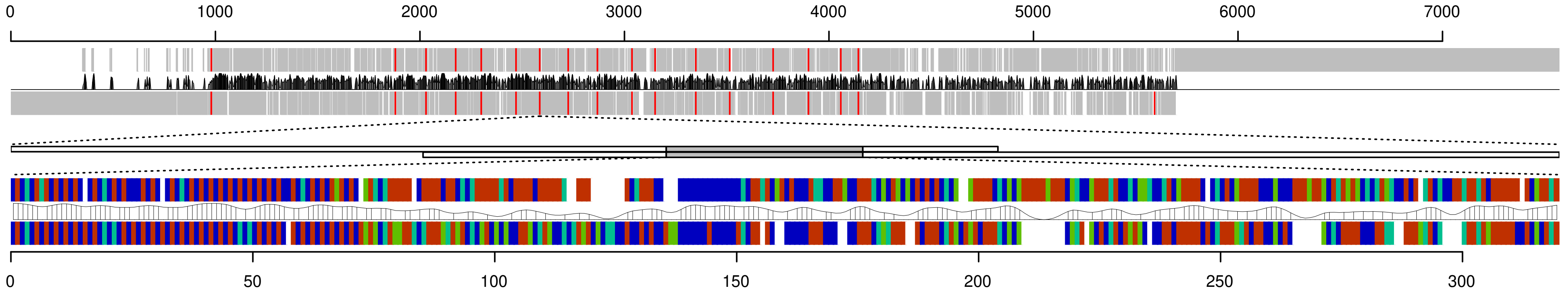

Danio rerio (ENSDART00000020680), Sorex araneus (ENSSART00000002526)

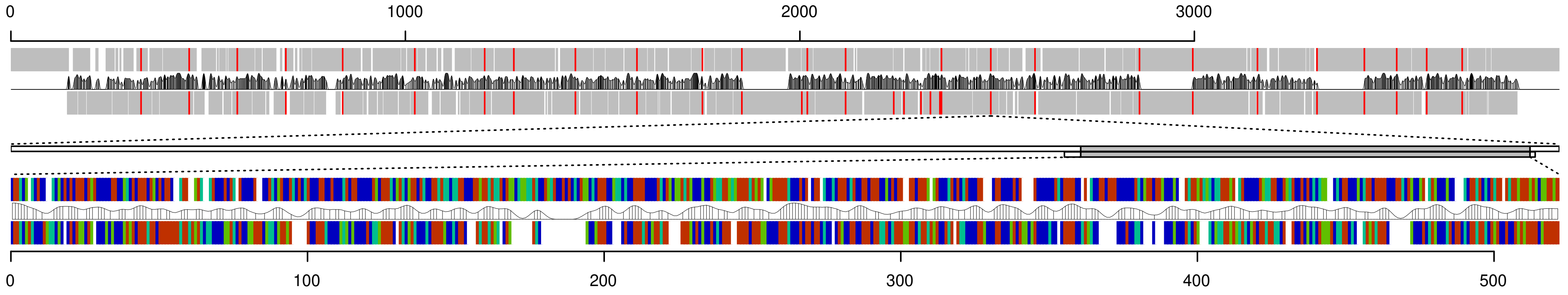

Danio rerio (ENSDART00000008532), Sarcophilus harrisii (ENSSHAT00000007152)

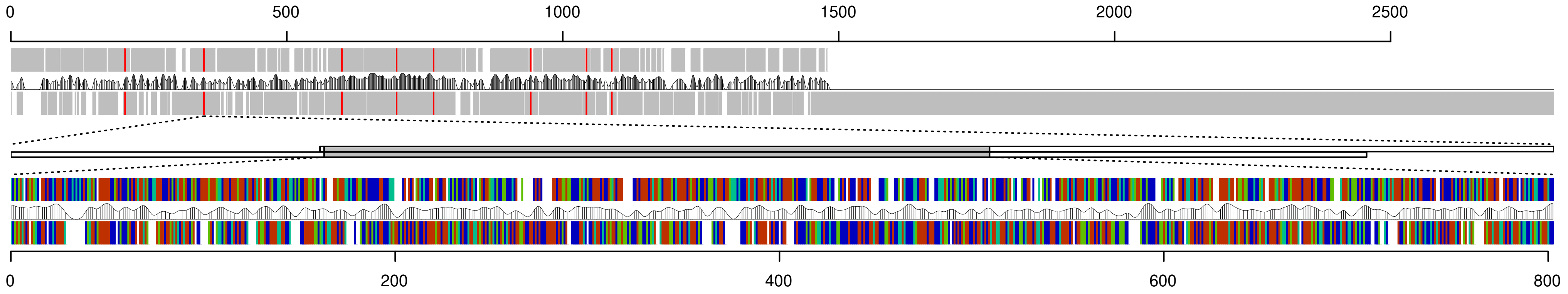

Danio rerio (ENSDART00000009164), Vulpes vulpes (ENSVVUT00000006026)

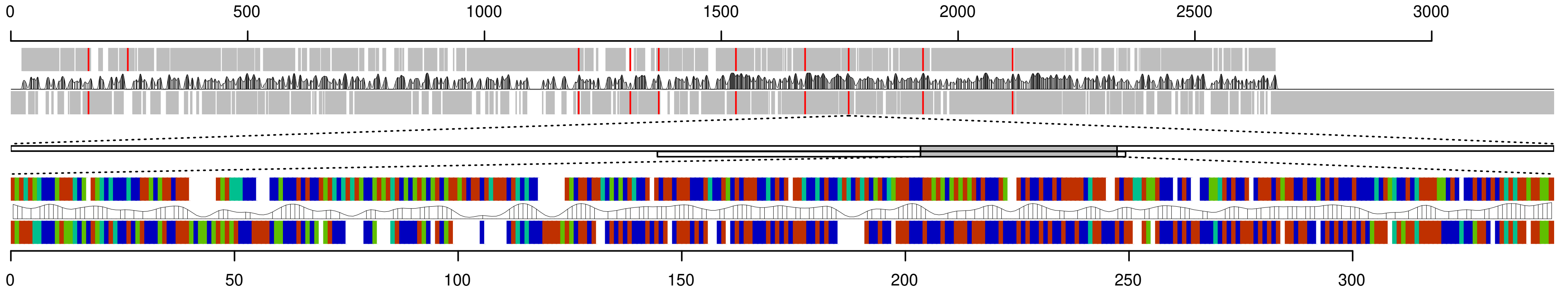

Danio rerio (ENSDART00000081772), Phascolarctos cinereus (ENSPCIT00000040211)

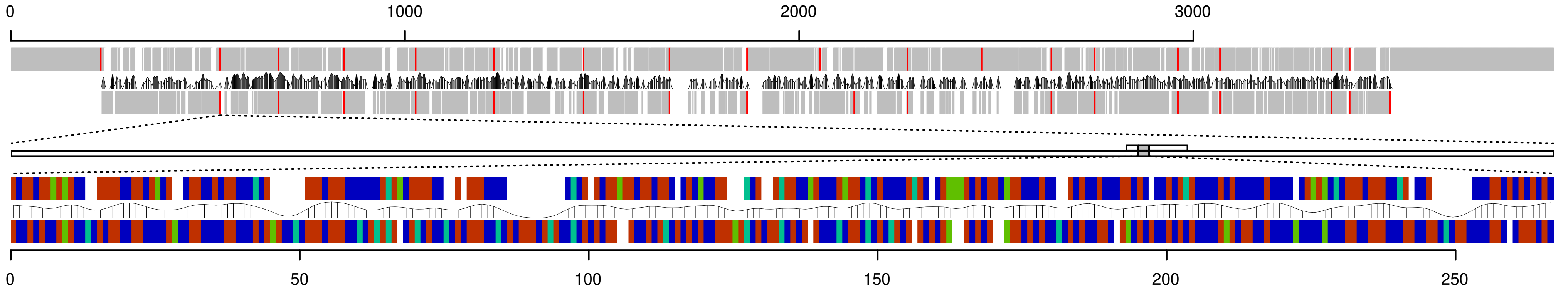

Danio rerio (ENSDART00000083317), Tursiops truncatus (ENSTTRT00000013065)

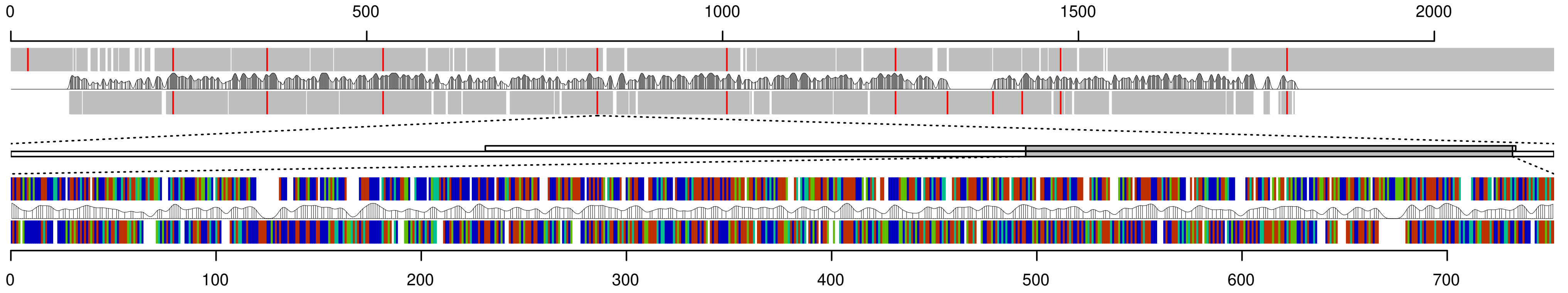

Danio rerio (ENSDART00000123935), Phascolarctos cinereus (ENSPCIT00000028881)

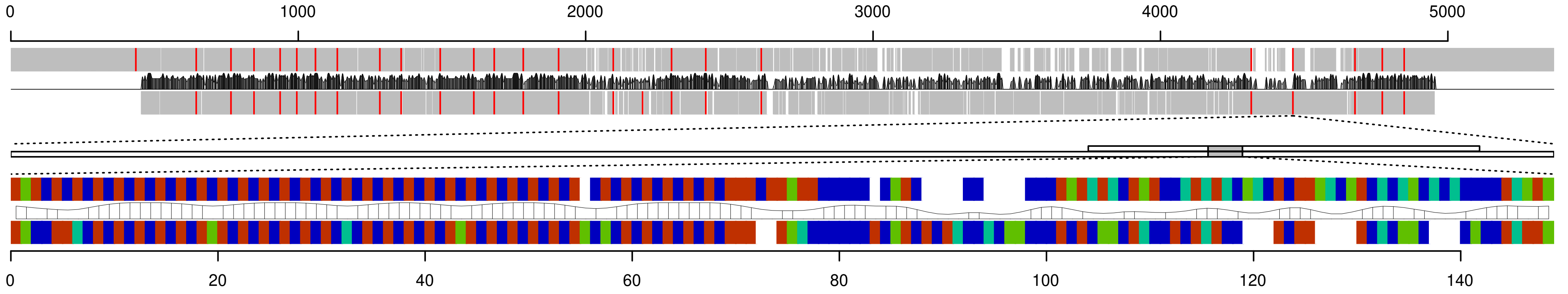

Danio rerio (ENSDART00000026800), Jaculus jaculus (ENSJJAT00000022237)

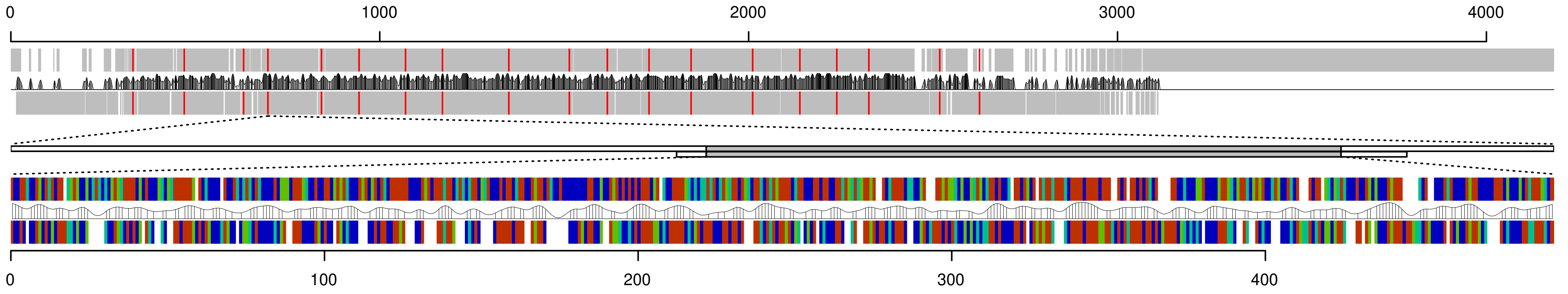

Danio rerio (ENSDART00000062699), Notamacropus eugenii (ENSMEUT00000007817)

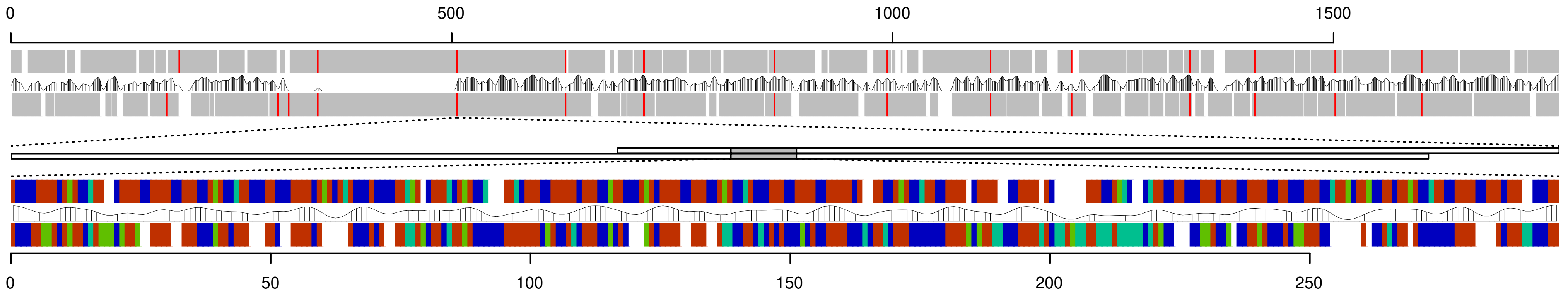

Danio rerio (ENSDART00000085319), Felis catus (ENSFCAT00000025255)

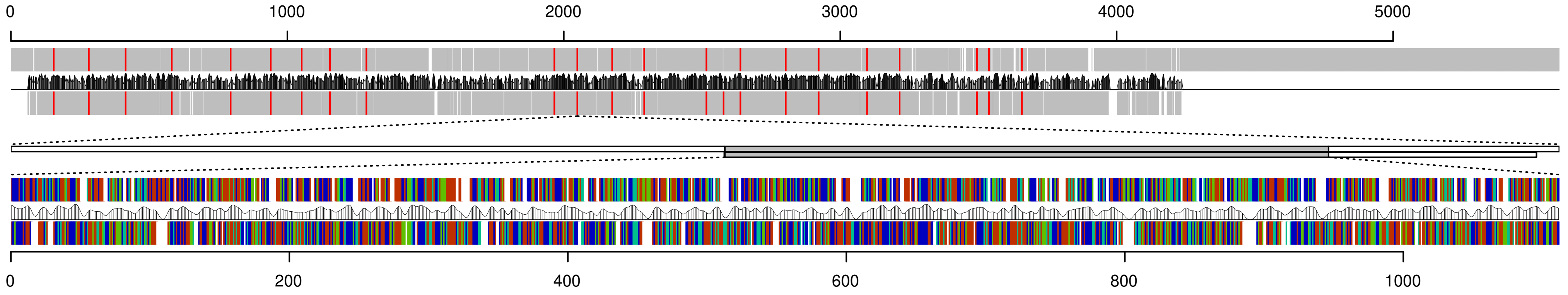

Danio rerio (ENSDART00000042064), Canis familiaris (ENSCAFT00000065164)

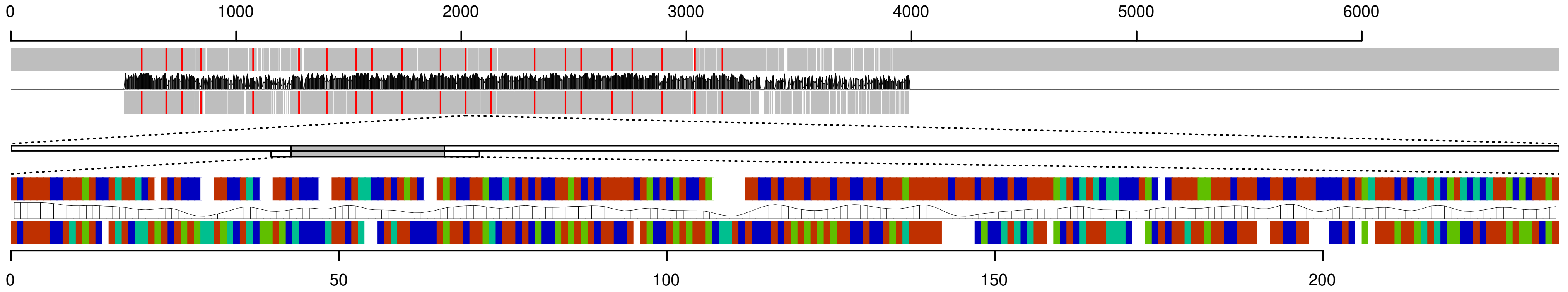

Danio rerio (ENSDART00000105590), Prolemur simus (ENSPSMT00000034782)

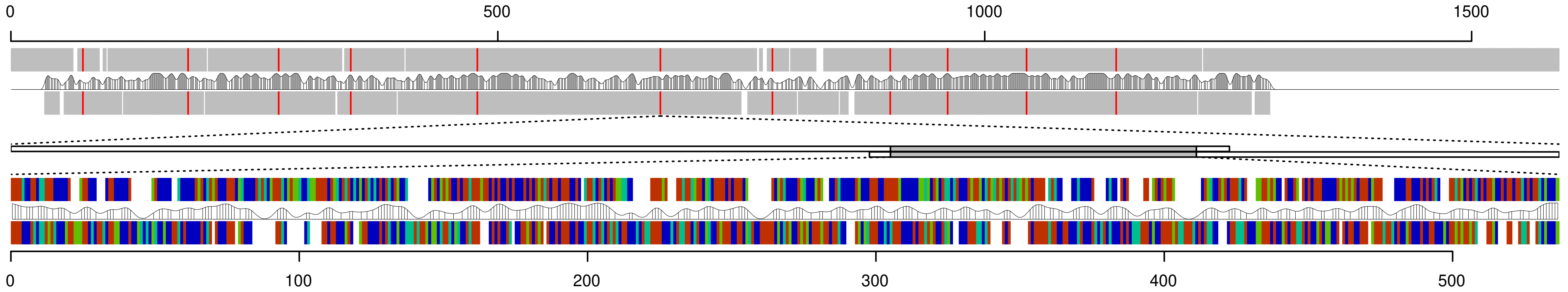

Danio rerio (ENSDART00000137844), Octodon degus (ENSODET00000025527)

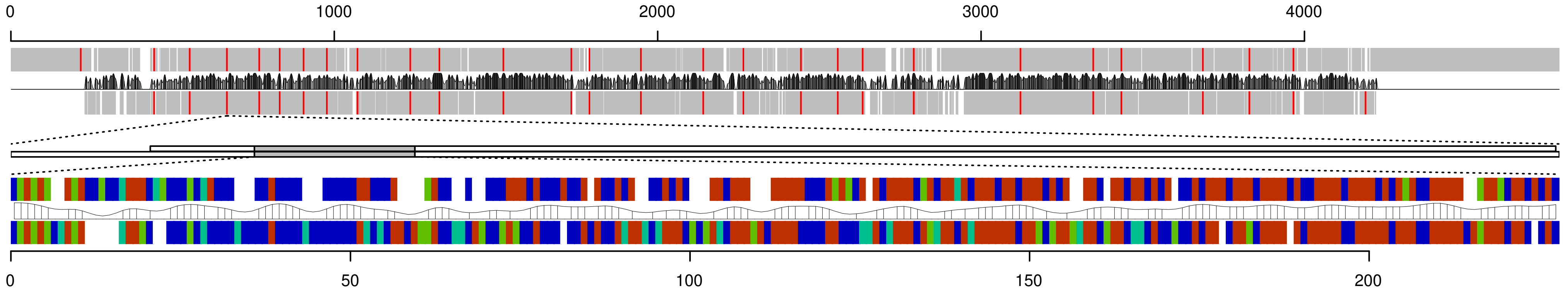

Danio rerio (ENSDART00000038120), Propithecus coquereli (ENSPCOT00000028367)

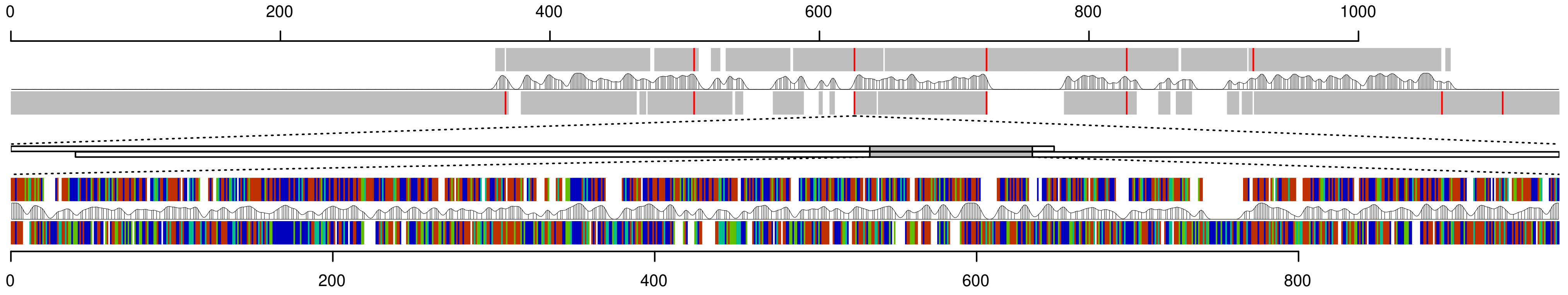

Danio rerio (ENSDART00000091901), Equus caballus (ENSECAT00000044602)

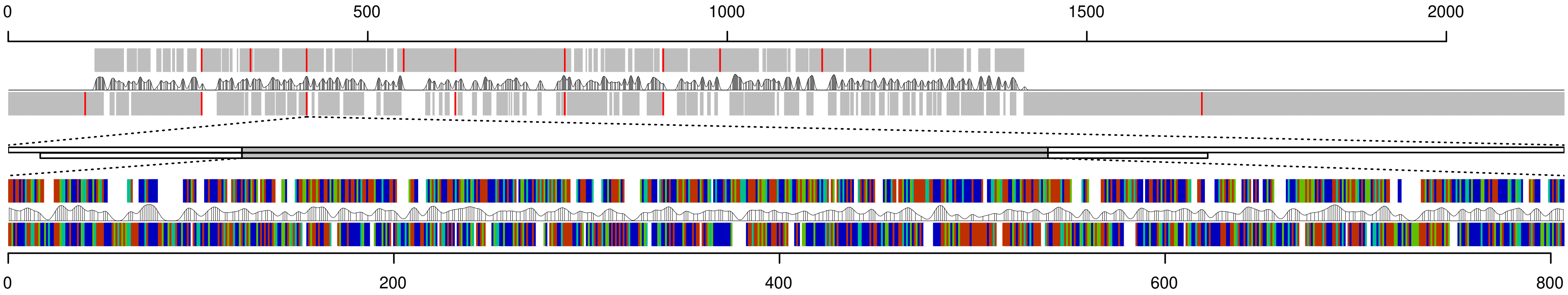

Danio rerio (ENSDART00000111454), Tursiops truncatus (ENSTTRT00000007785)

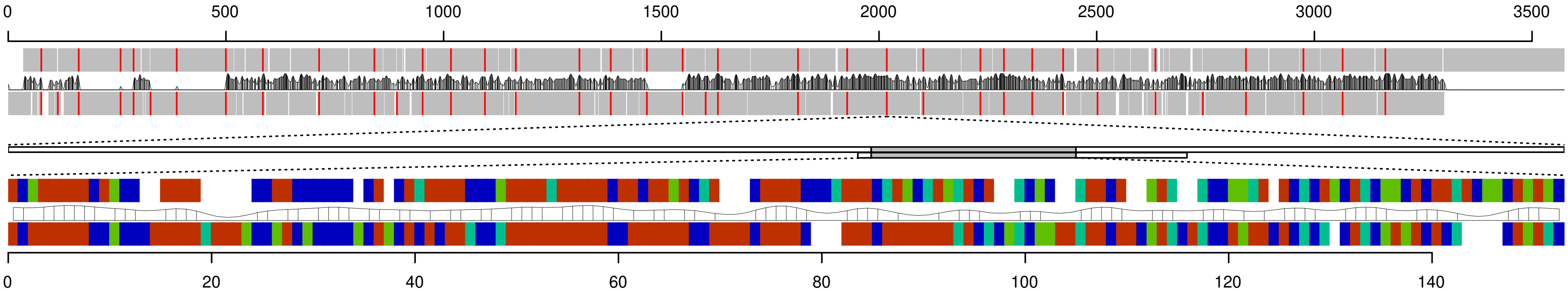

Danio rerio (ENSDART00000160547), Nannospalax galili (ENSNGAT00000031716)

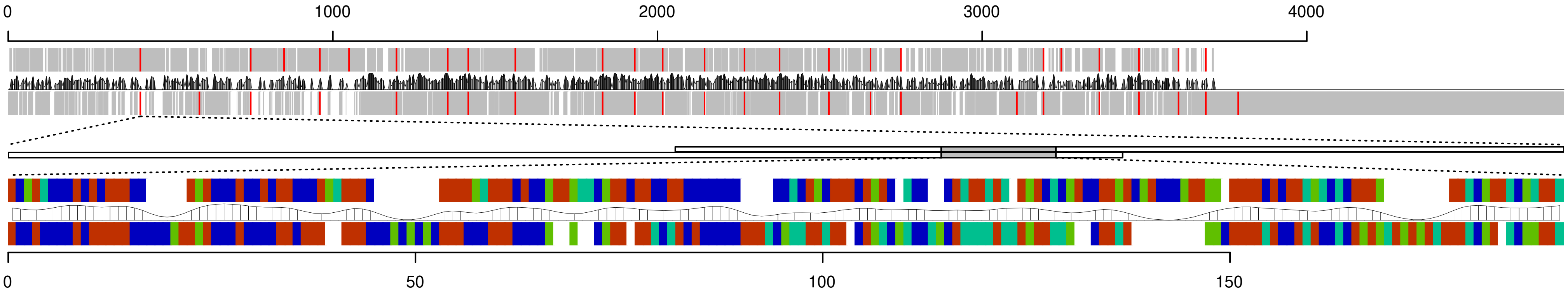

Danio rerio (ENSDART00000058876), Myotis lucifugus (ENSMLUT00000006506)

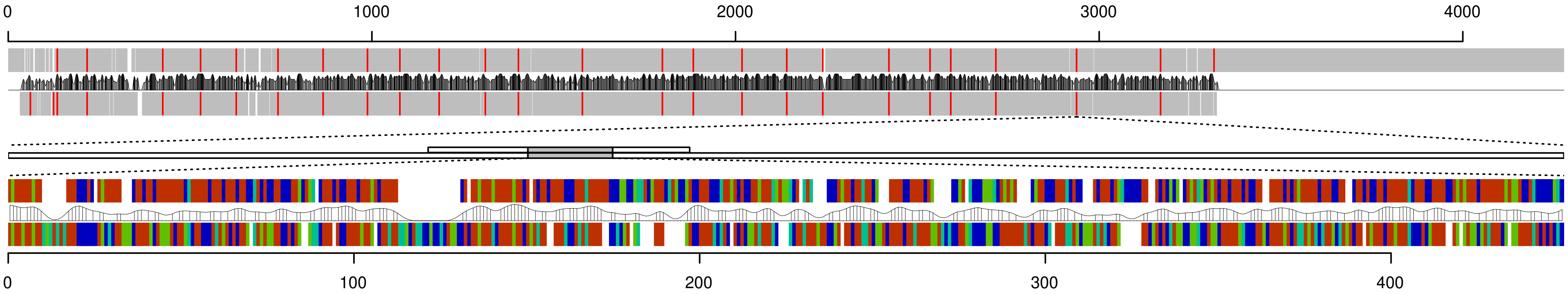

Danio rerio (ENSDART00000125362), Felis catus (ENSFCAT00000041198)

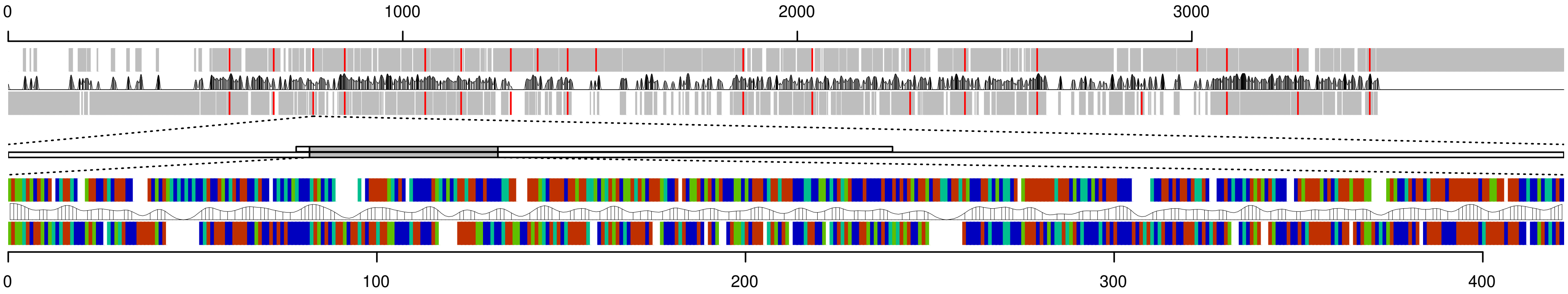

Danio rerio (ENSDART00000140673), Microtus ochrogaster (ENSMOCT00000021674)

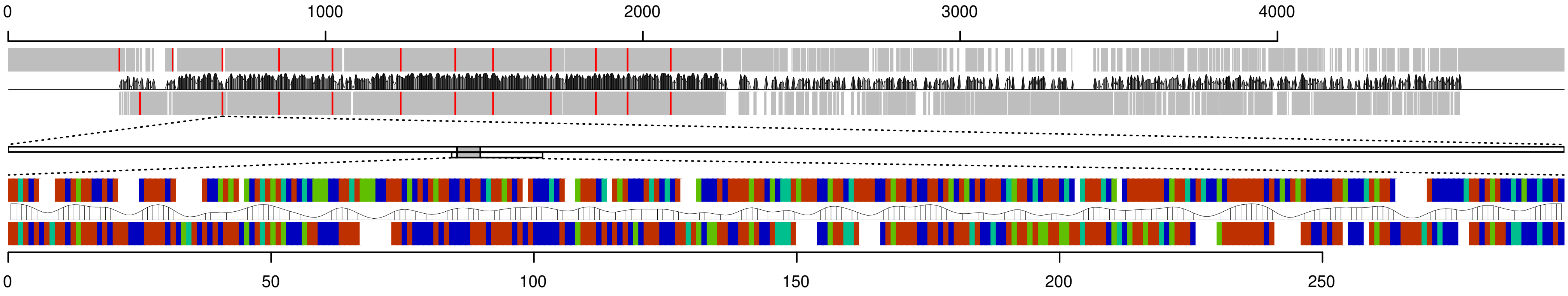

Danio rerio (ENSDART00000085165), Sarcophilus harrisii (ENSSHAT00000022267)

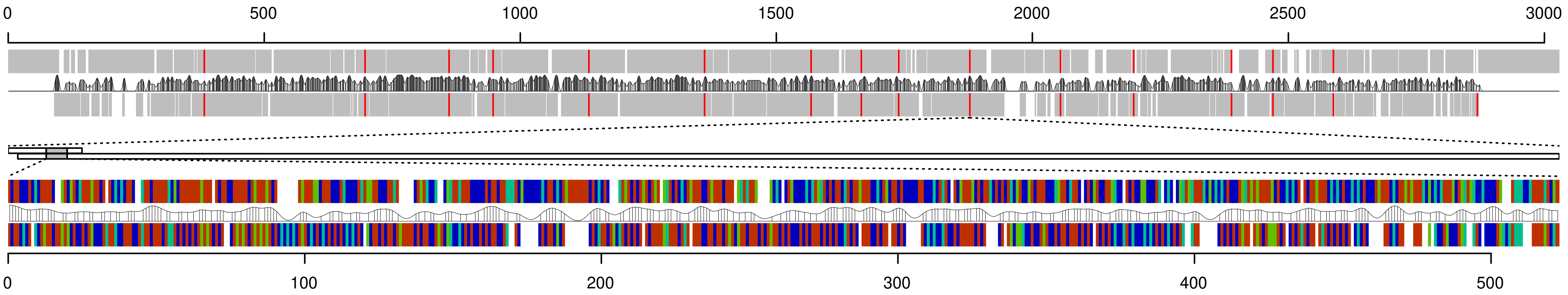

Danio rerio (ENSDART00000137575), Pteropus vampyrus (ENSPVAT00000005427)

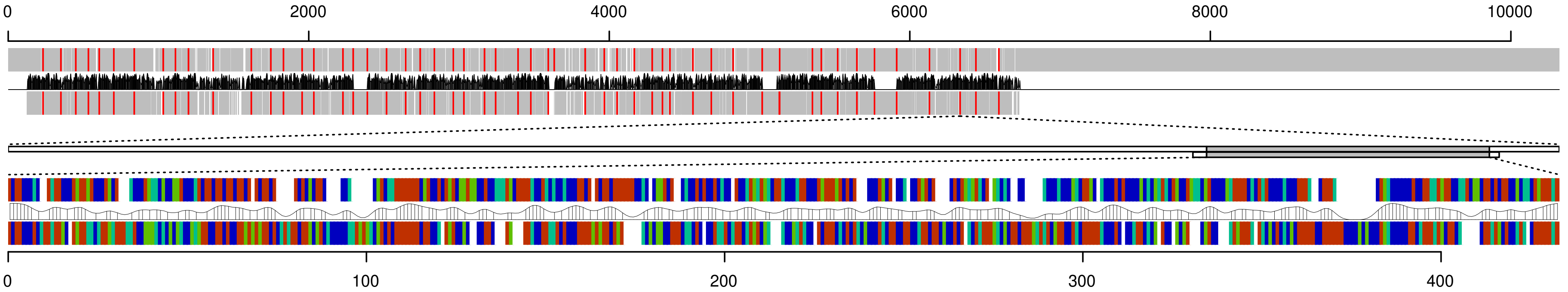

Danio rerio (ENSDART00000154639), Ictidomys tridecemlineatus (ENSSTOT00000036727)

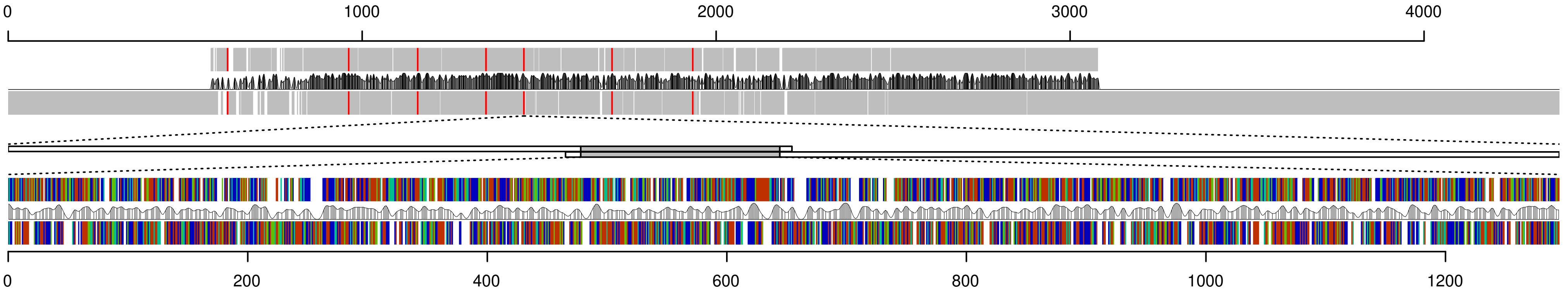

Danio rerio (ENSDART00000141799), Rattus norvegicus (ENSRNOT00000020925)

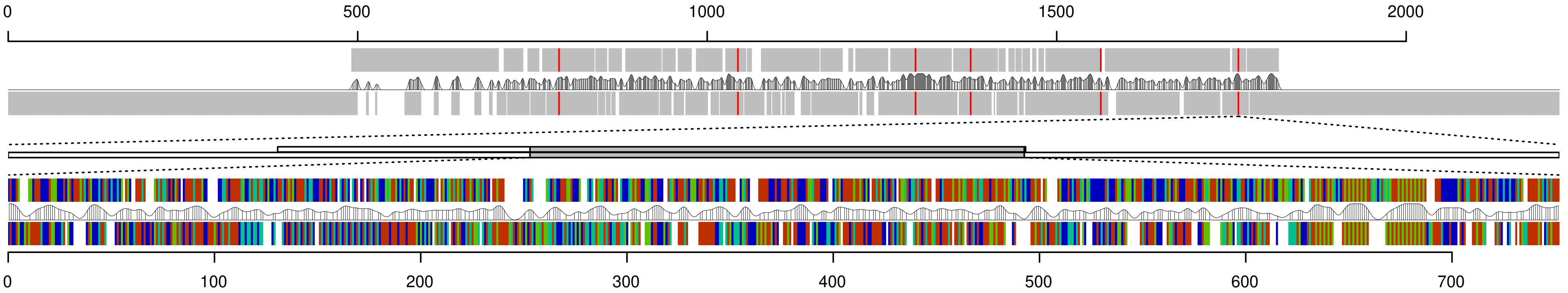

Danio rerio (ENSDART00000042134), Urocitellus parryii (ENSUPAT00010023009)

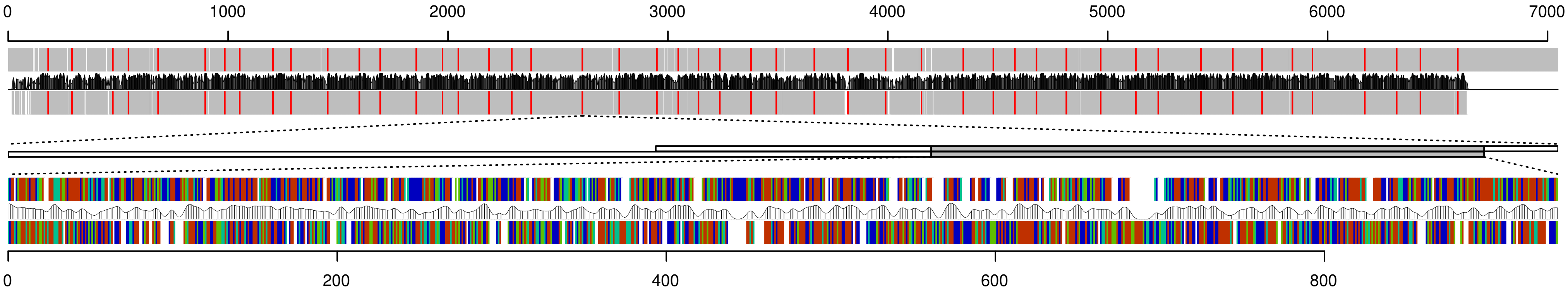

Danio rerio (ENSDART00000123916), Dasypus novemcinctus (ENSDNOT00000016877)

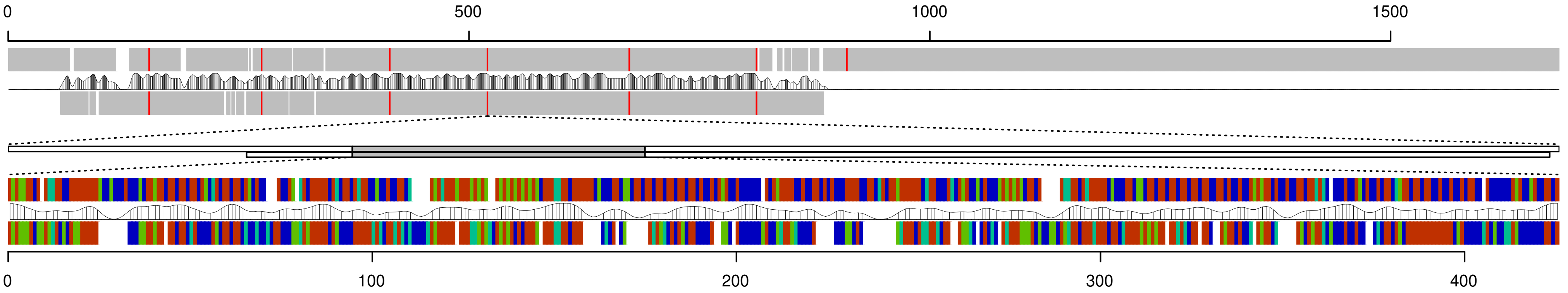

Danio rerio (ENSDART00000179701), Papio anubis (ENSPANT00000018612)

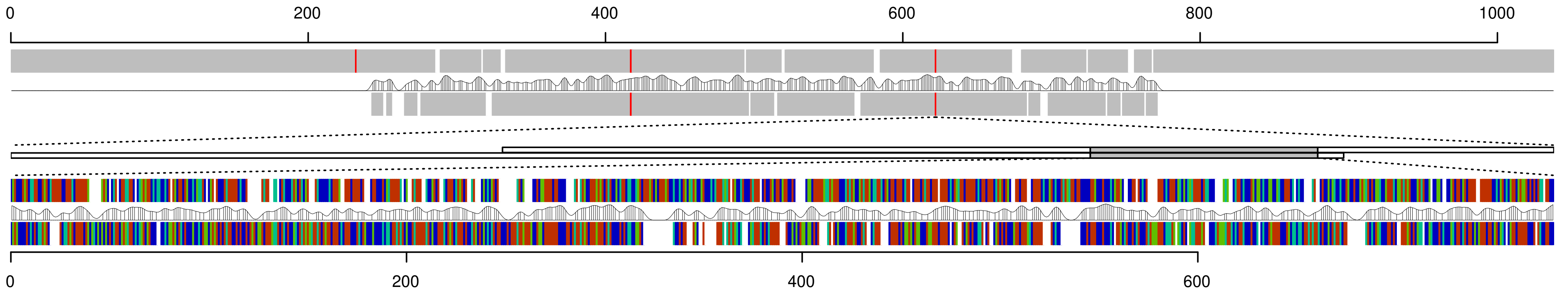

Danio rerio (ENSDART00000110824), Spermophilus dauricus (ENSSDAT00000009227)

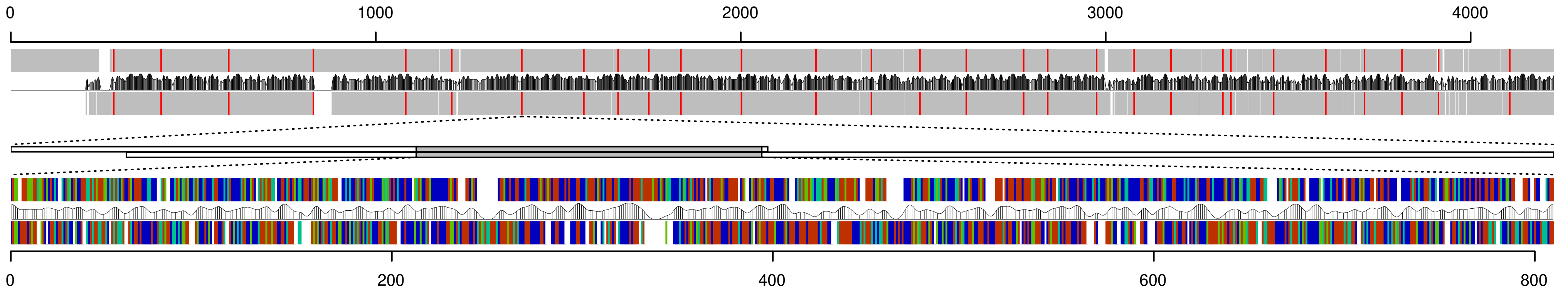

Danio rerio (ENSDART00000058876), Mus pahari (MGP_PahariEiJ_T0085731)

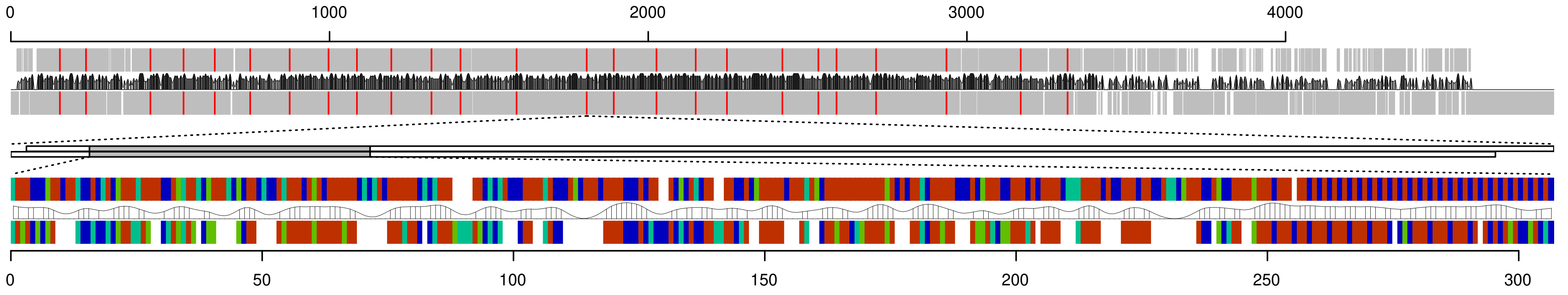

Danio rerio (ENSDART00000051546), Castor canadensis (ENSCCNT00000027489)

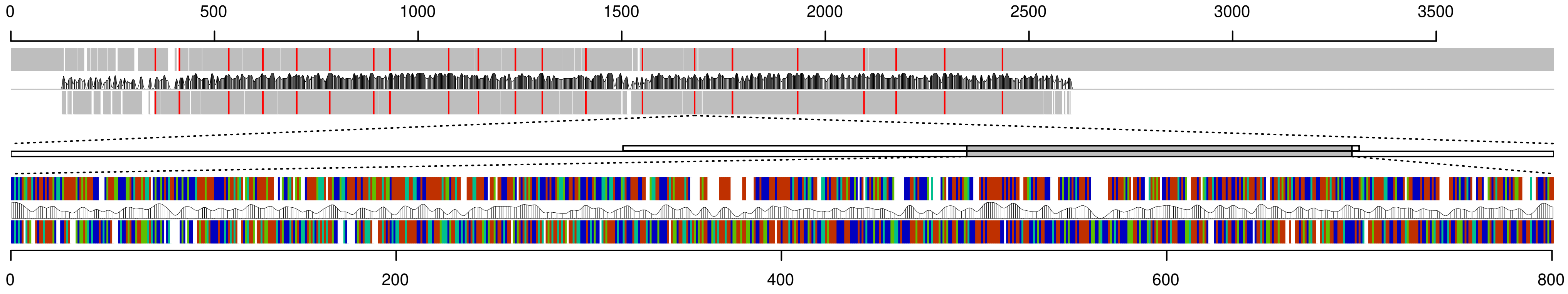

Danio rerio (ENSDART00000100651), Mus spretus (MGP_SPRETEiJ_T0063622)

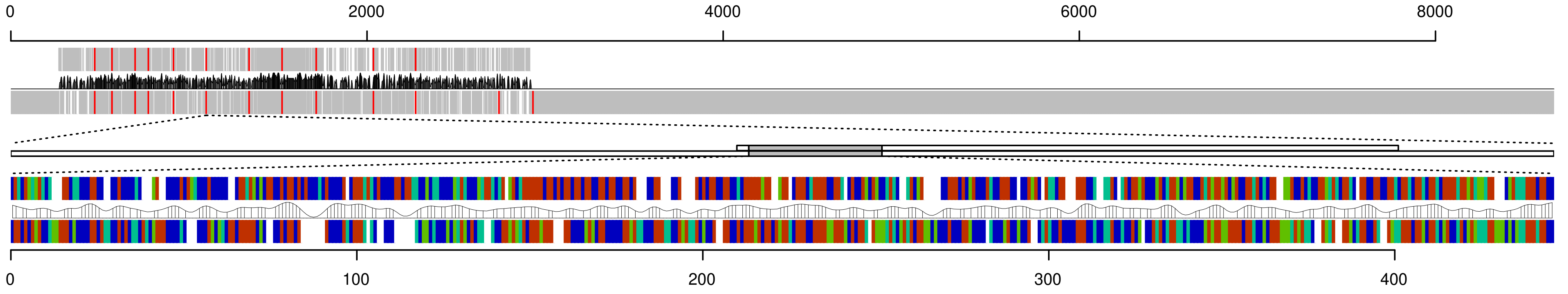

Danio rerio (ENSDART00000104072), Cavia porcellus (ENSCPOT00000012861)

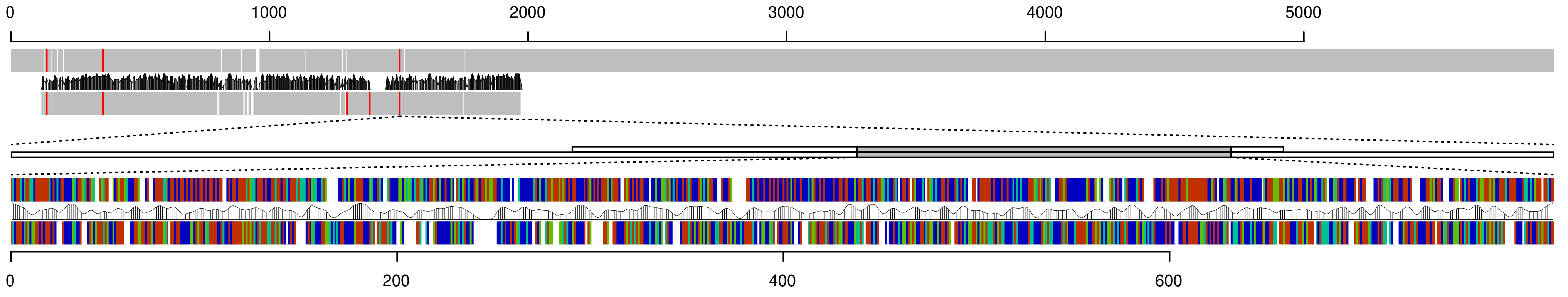

Danio rerio (ENSDART00000016488), Rhinopithecus bieti (ENSRBIT00000063494)

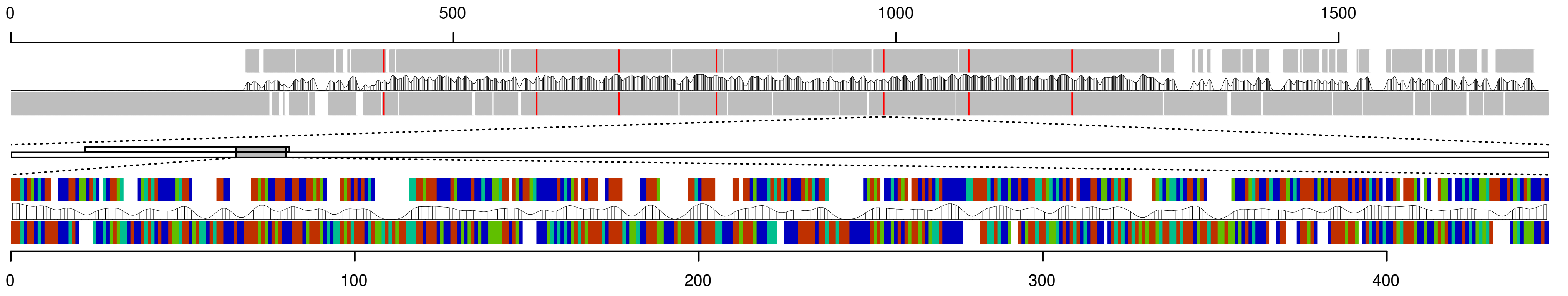

Danio rerio (ENSDART00000127329), Ailuropoda melanoleuca (ENSAMET00000015297)

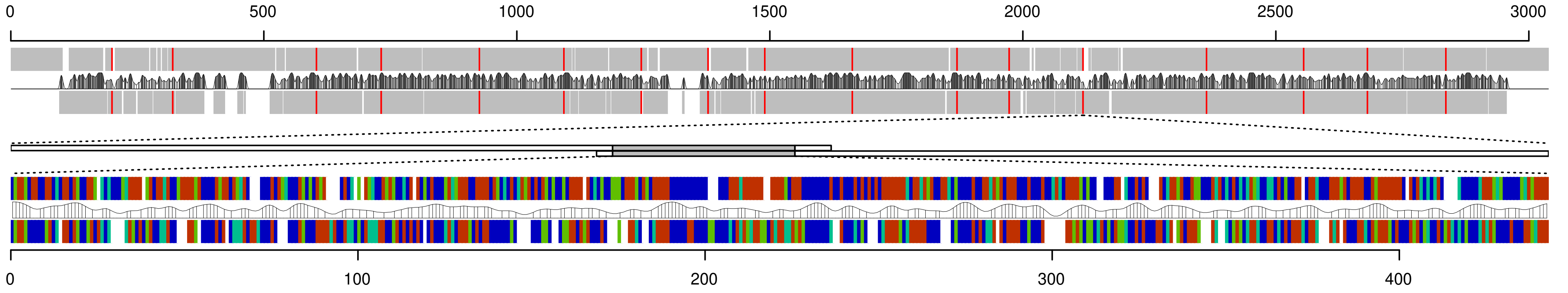

Danio rerio (ENSDART00000075601), Tupaia belangeri (ENSTBET00000007890)

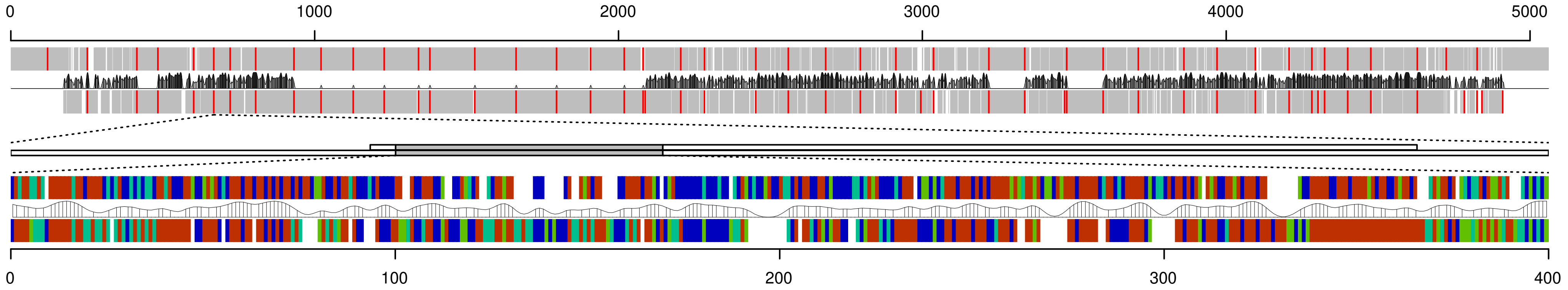

Danio rerio (ENSDART00000055845), Urocitellus parryii (ENSUPAT00010003600)

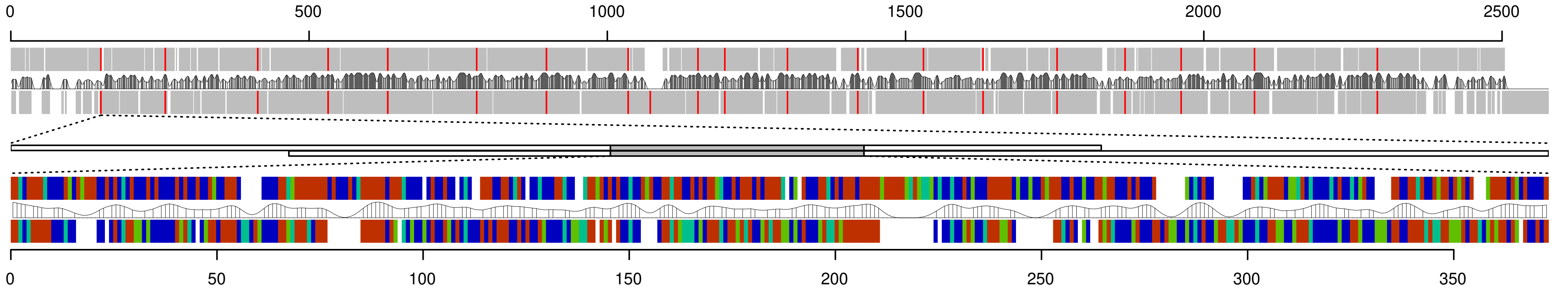

Danio rerio (ENSDART00000046211), Carlito syrichta (ENSTSYT00000012962)

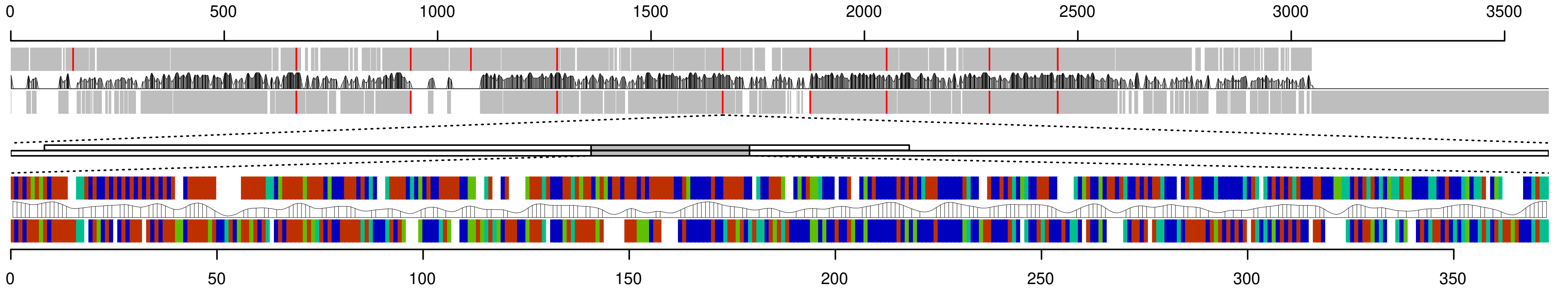

Danio rerio (ENSDART00000180753), Cavia porcellus (ENSCPOT00000010675)

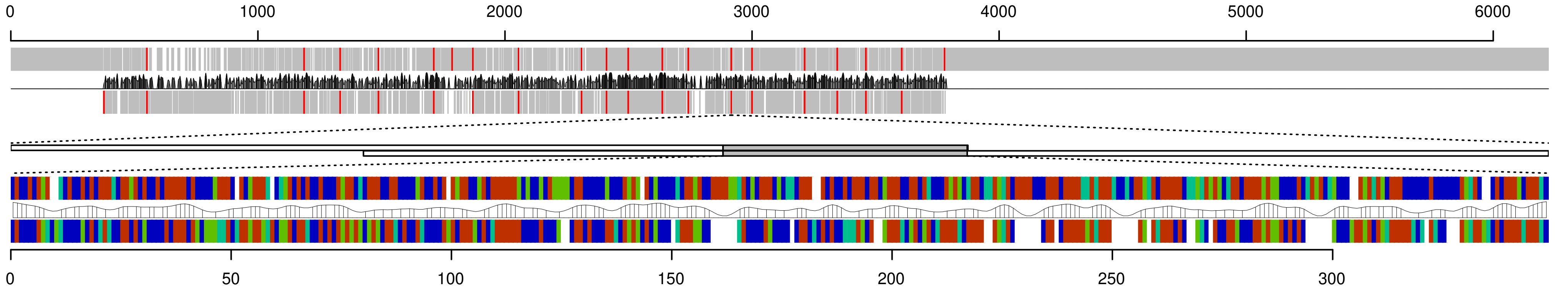

Danio rerio (ENSDART00000014536), Erinaceus europaeus (ENSEEUT00000005440)

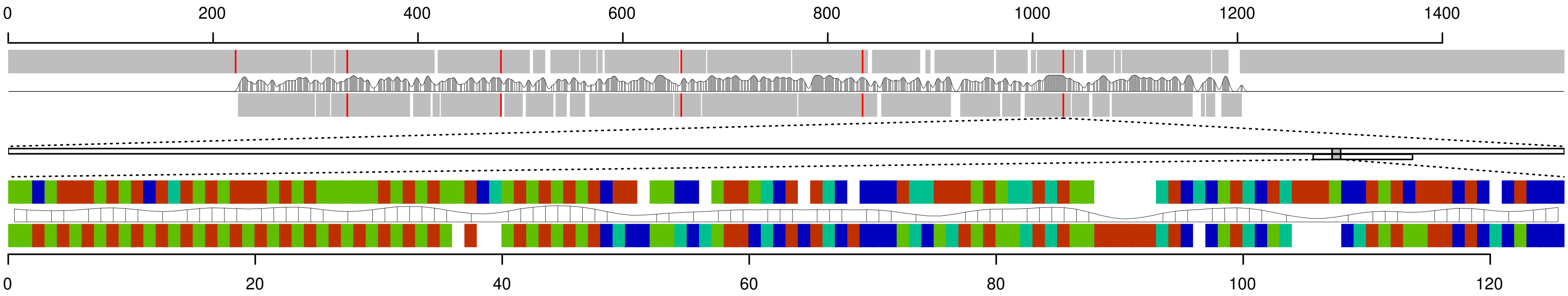

Danio rerio (ENSDART00000166649), Ursus americanus (ENSUAMT00000017340)

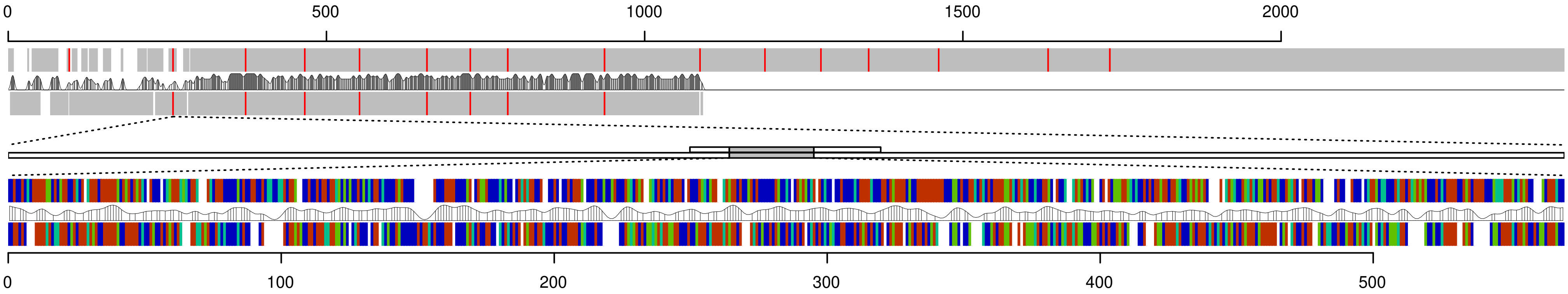

Danio rerio (ENSDART00000105286), Ovis aries (ENSOART00000011280)

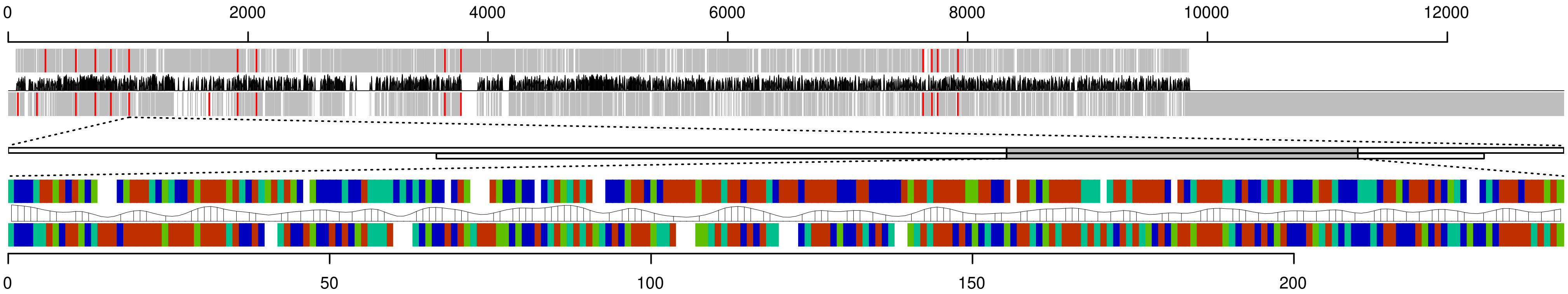

Danio rerio (ENSDART00000153726), Procavia capensis (ENSPCAT00000010832)

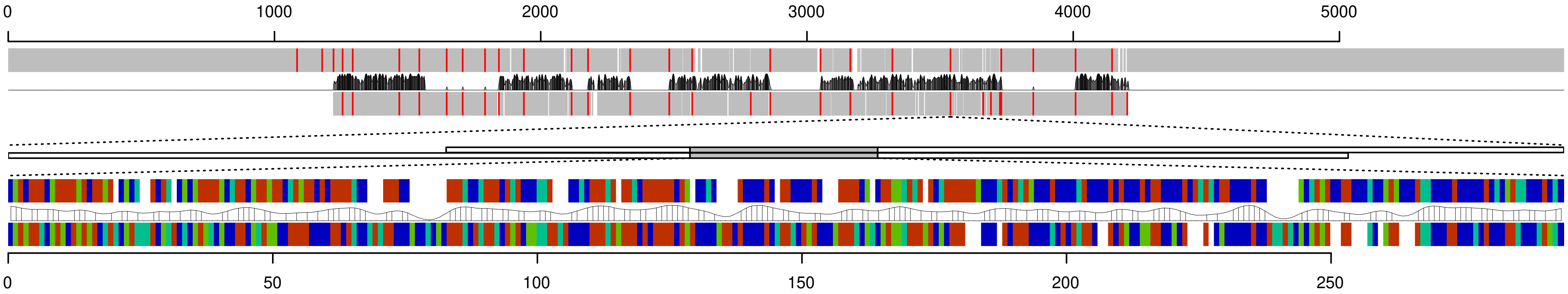

Danio rerio (ENSDART00000000887), Sorex araneus (ENSSART00000001582)

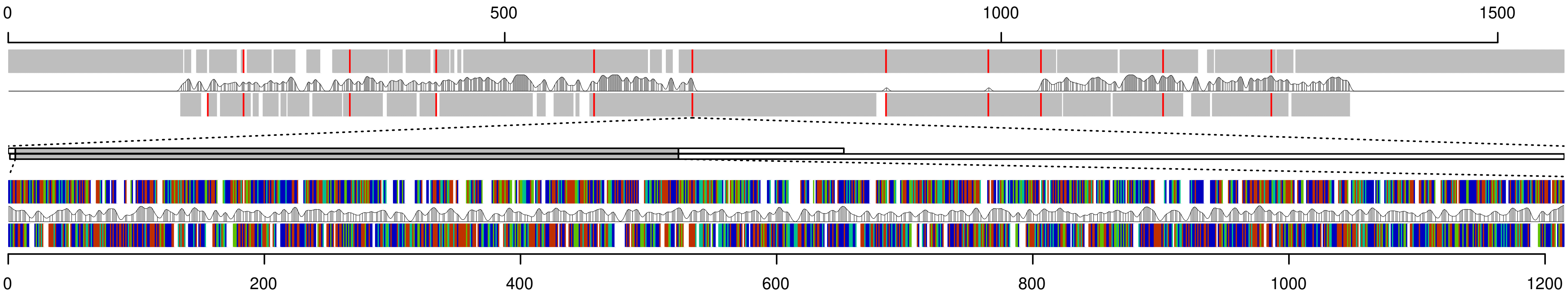

Danio rerio (ENSDART00000109412), Choloepus hoffmanni (ENSCHOT00000000668)

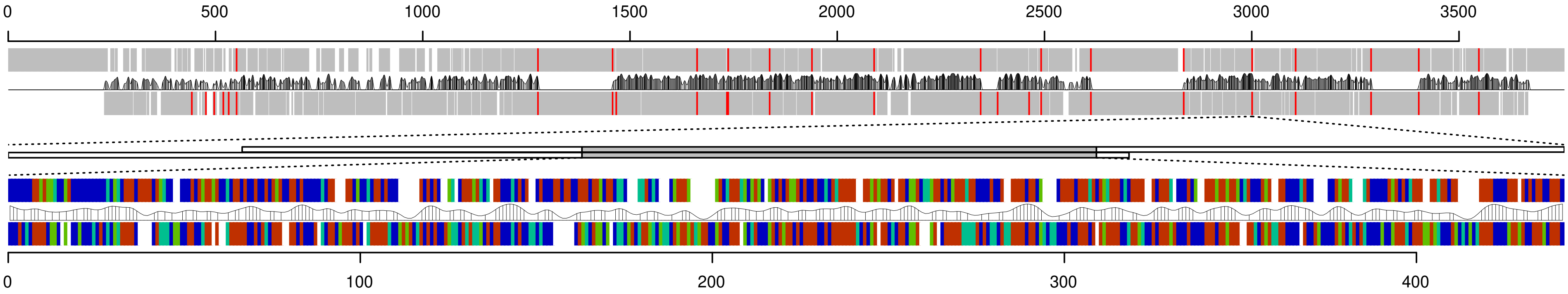

Supplement: Supplementary file 6 — Transcript and intron alignments for points in Fig. S14. Each panel shows the maximally scoring alignment between D. rerio and teleost intron orthologues (lower) and transcript alignment (upper) used to establish the intron orthology. Grey, white and red parts indicate aligned exonic sequence, gaps and positions of intron meta-characters respectively. Colours in intron alignment represent bases (A blue, C cyan, G green, T brown, N grey, gap white). Curves lying between sequence representations show a normal kernel density smoothed estimate of local similarity (9 bp window, standard deviation two); vertical lines indicate matches. Region between exon and intron alignments indicates the location of the maximally scoring alignment in the introns. Upper sequence D. rerio. Files 6–10 and 11–15 contain alignments to teleost and mammalian sequences respectively. Each file corresponds to one panel in Fig. S17 and to one specific teleost size class: Files 6,11: long (E,J), 7,12: medium (D,I), 8,13: short.2 (C,H), 9,14 short (B, G) and 10,15 ctl (A,F). [file 12864_2022_8760_MOESM6_ESM.zip › 12864_2022_8760_MOESM15_ESM.pdf]
