# Supplementary material for: Intron size minimisation in teleosts
Source: BMC Genomics. 2022 Sep 1;23:628. doi: 10.1186/s12864-022-08760-w (PMC9438311; doi:10.1186/s12864-022-08760-w)

Danio rerio (ENSDART00000124112), Nomascus leucogenys (ENSNLET00000007138)

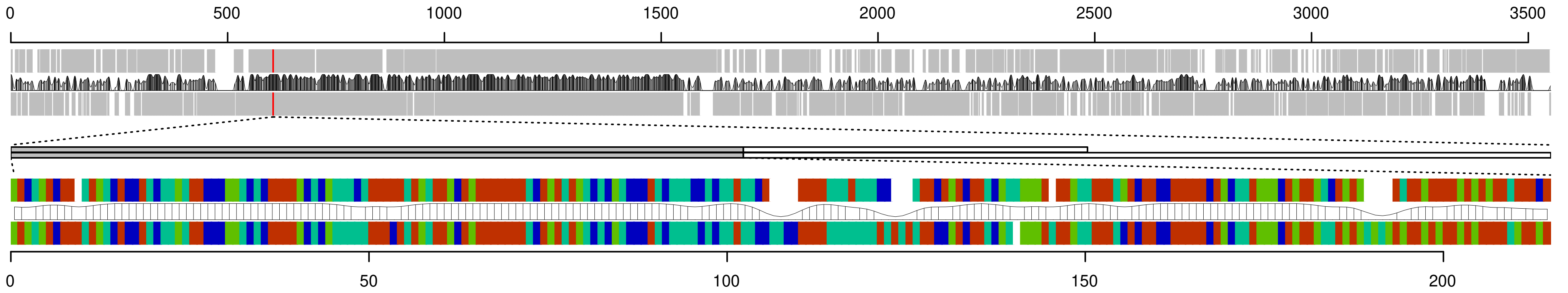

Danio rerio (ENSDART00000187248), Monodelphis domestica (ENSMODT00000018922)

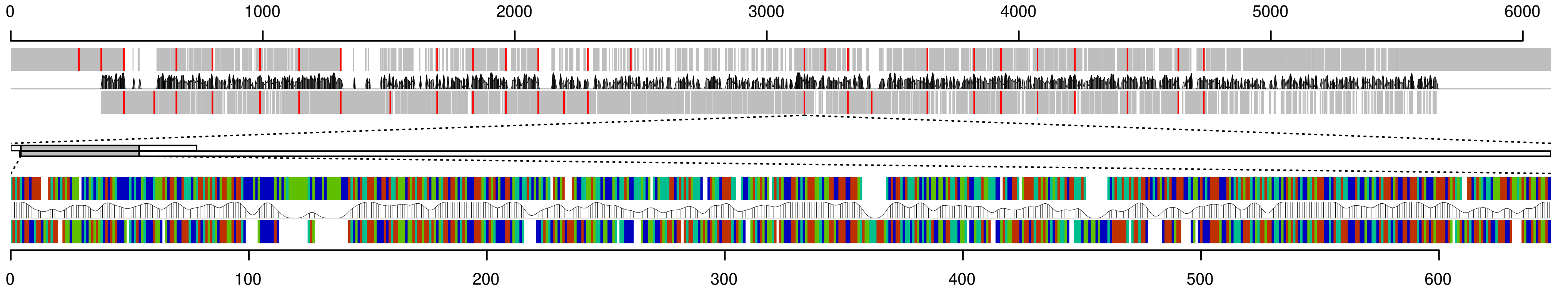

Danio rerio (ENSDART00000019910), Sarcophilus harrisii (ENSSHAT00000019603)

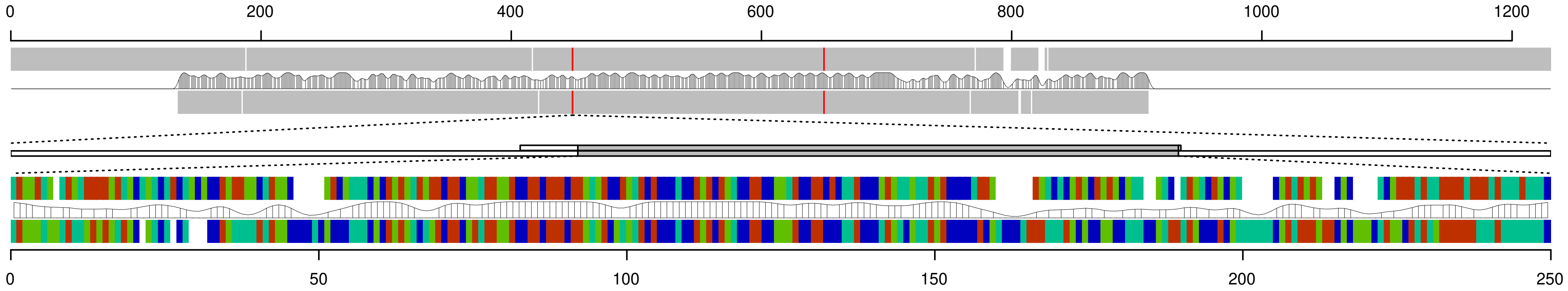

Danio rerio (ENSDART00000045991), Ovis aries (ENSOART00000000112)

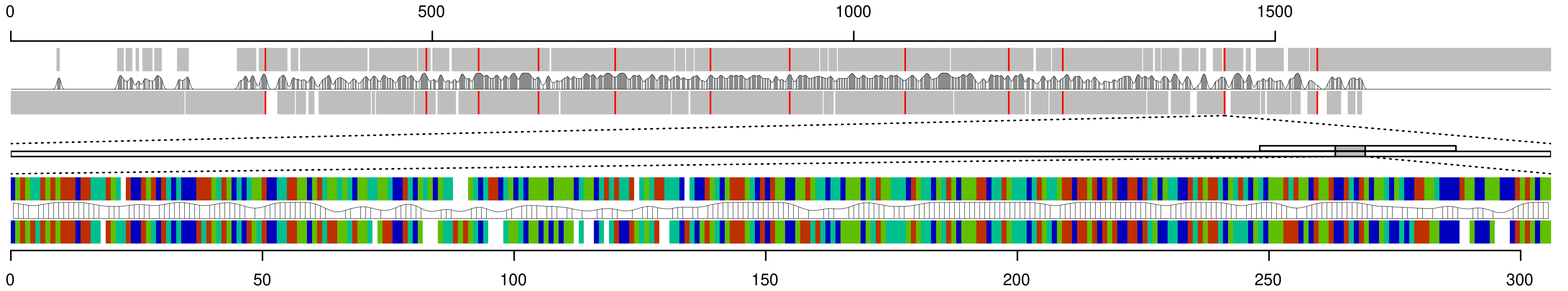

Danio rerio (ENSDART00000017176), Ornithorhynchus anatinus (ENSOANT00000013884)

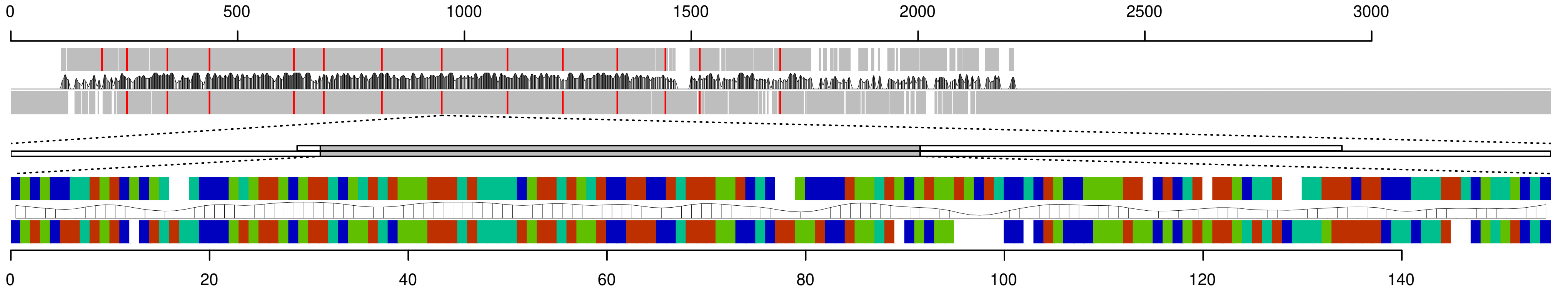

Danio rerio (ENSDART00000050915), Sarcophilus harrisii (ENSSHAT00000007458)

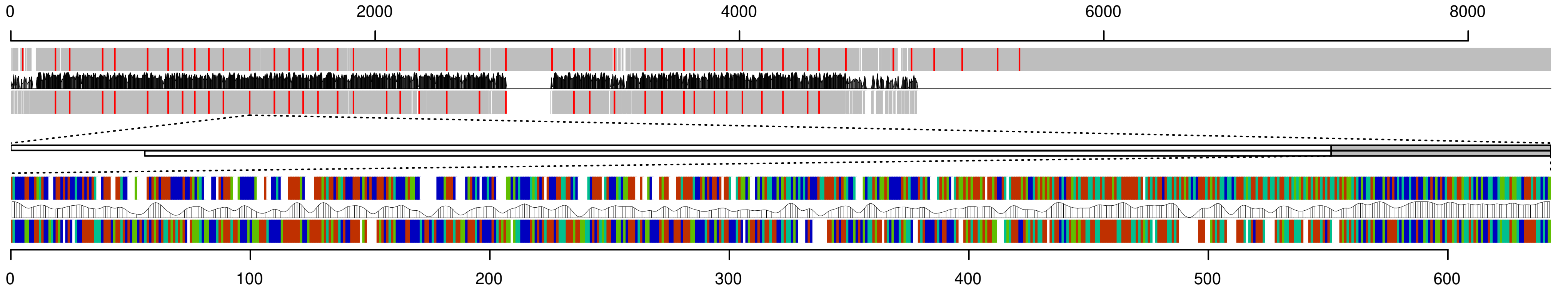

Danio rerio (ENSDART00000168537), Notamacropus eugenii (ENSMEUT00000009262)

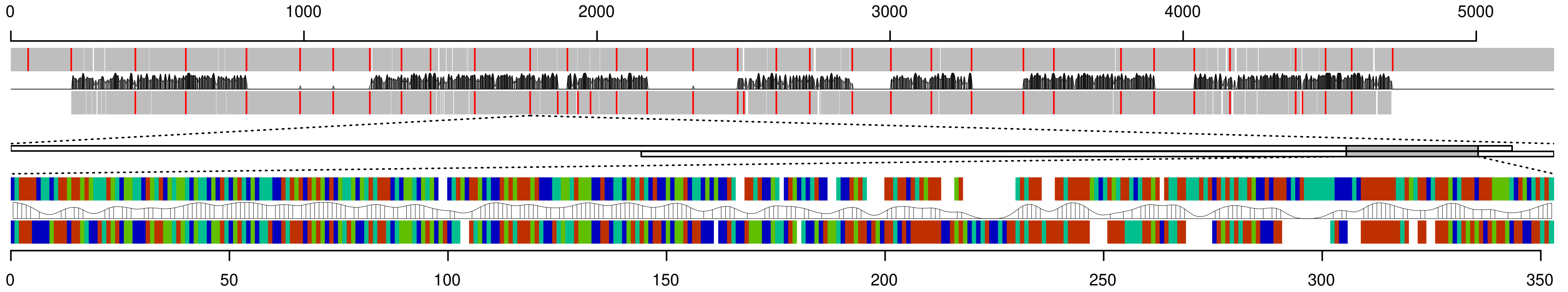

Danio rerio (ENSDART00000161988), Sarcophilus harrisii (ENSSHAT00000019778)

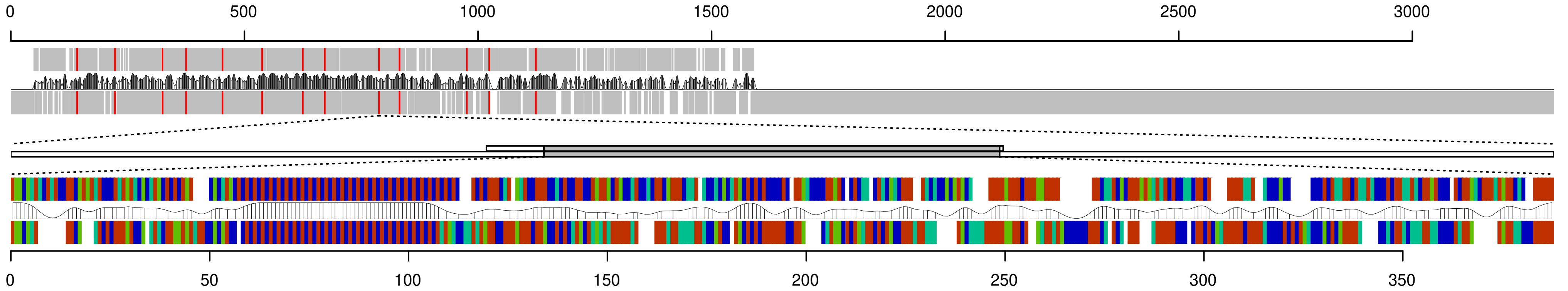

Danio rerio (ENSDART00000010148), Phascolarctos cinereus (ENSPCIT00000021891)

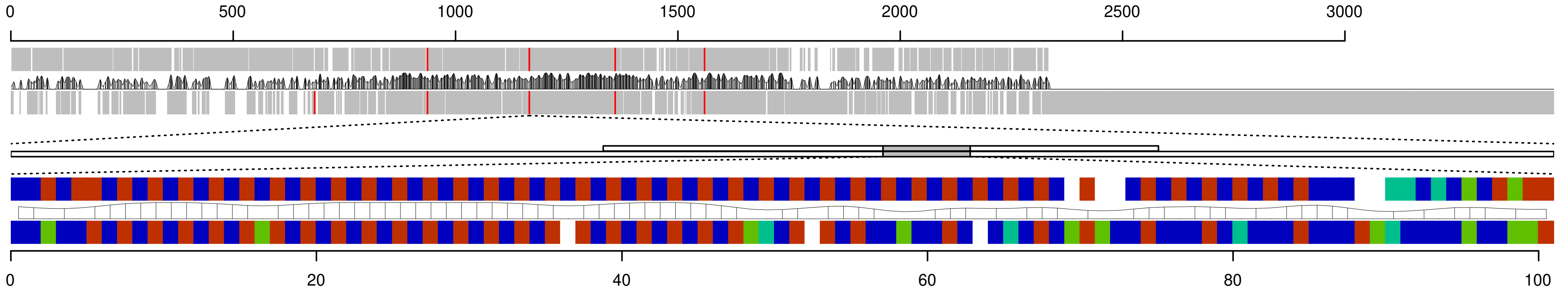

Danio rerio (ENSDART00000102335), Ovis aries (ENSOART00000015428)

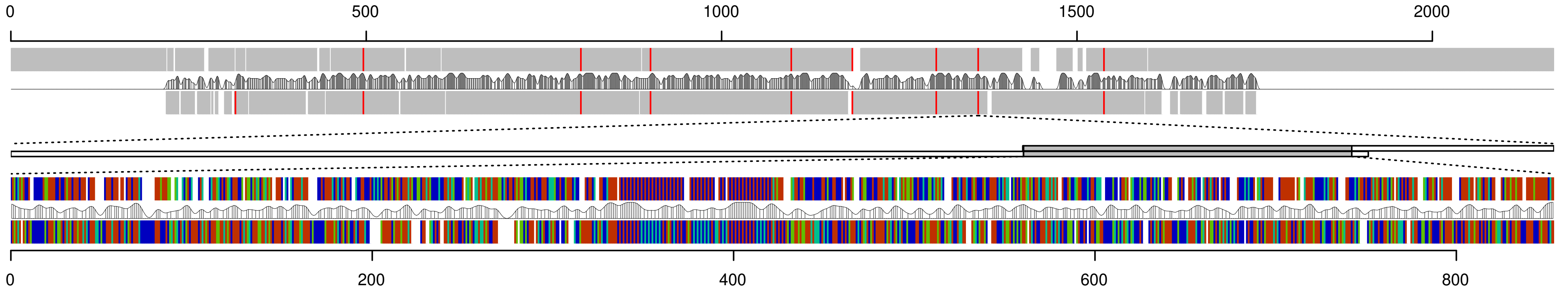

Danio rerio (ENSDART00000090165), Nomascus leucogenys (ENSNLET00000022467)

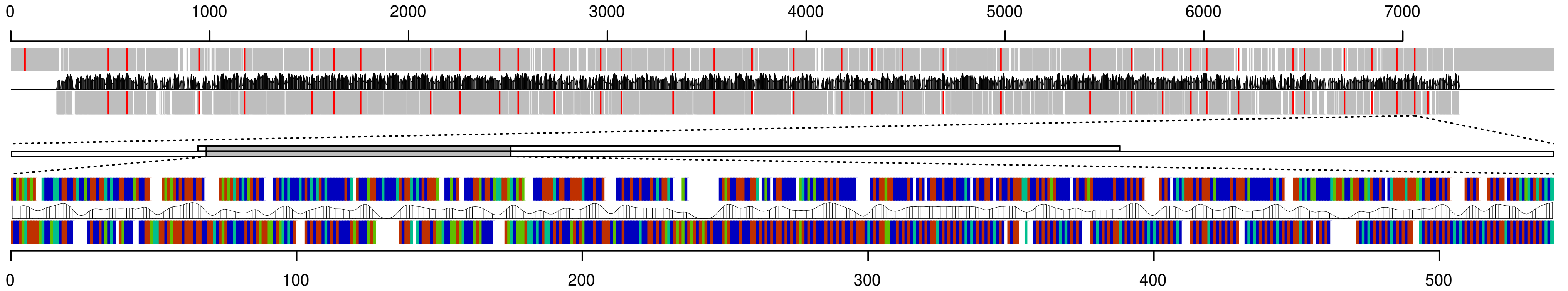

Danio rerio (ENSDART00000153595), Oryctolagus cuniculus (ENSOCUT00000009068)

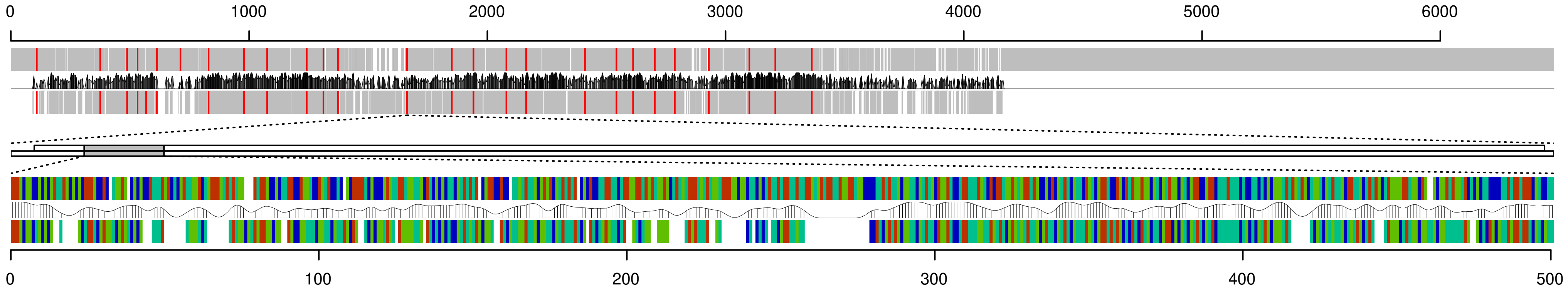

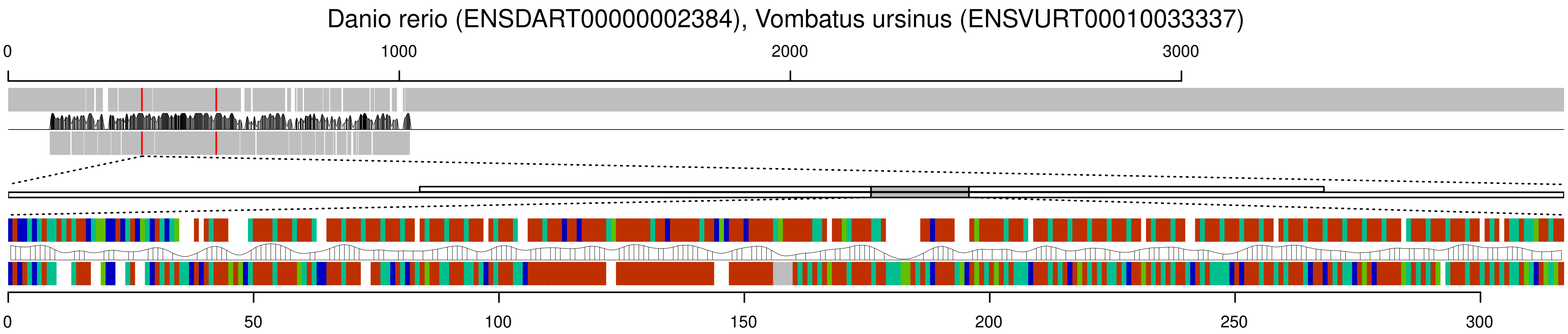
Danio rerio (ENSDART00000002384), Vombatus ursinus (ENSVURT00010033337)
0
1000
2000
3000
0
50
100
150
200
250
300

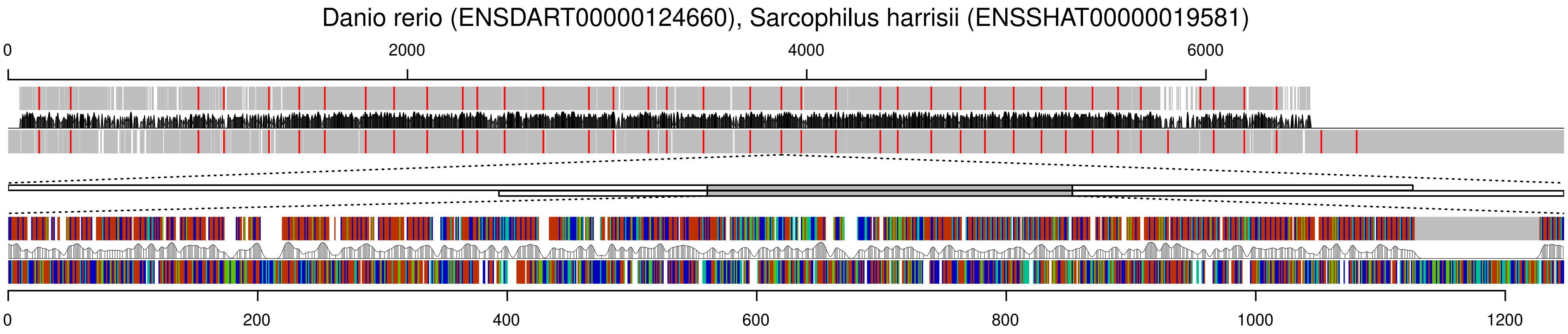
Danio rerio (ENSDART00000124660), Sarcophilus harrisii (ENSSHAT00000019581)
0
2000
4000
6000
0
200
400
600
800
1000
1200

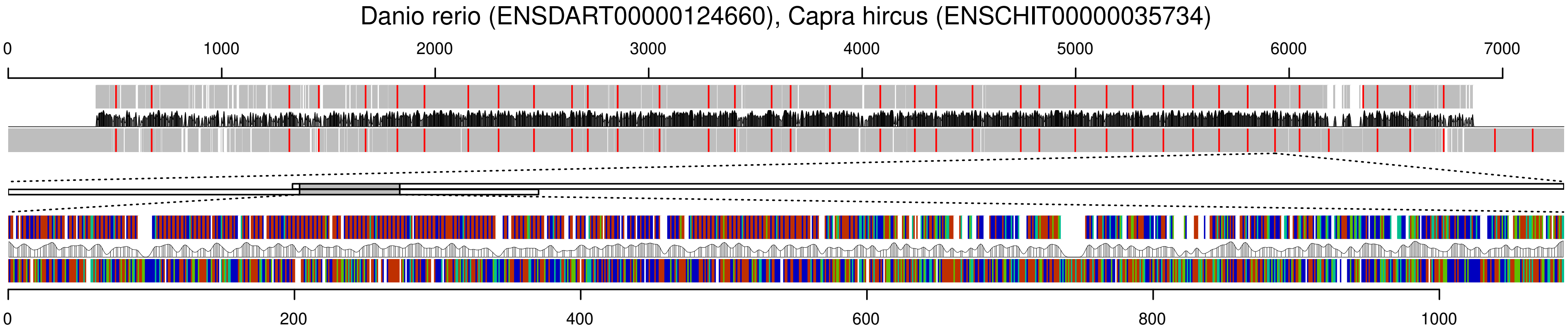
Danio rerio (ENSDART00000124660), Capra hircus (ENSCHIT00000035734)
0
1000
2000
3000
4000
5000
6000
7000
0
200
400
600
800
1000

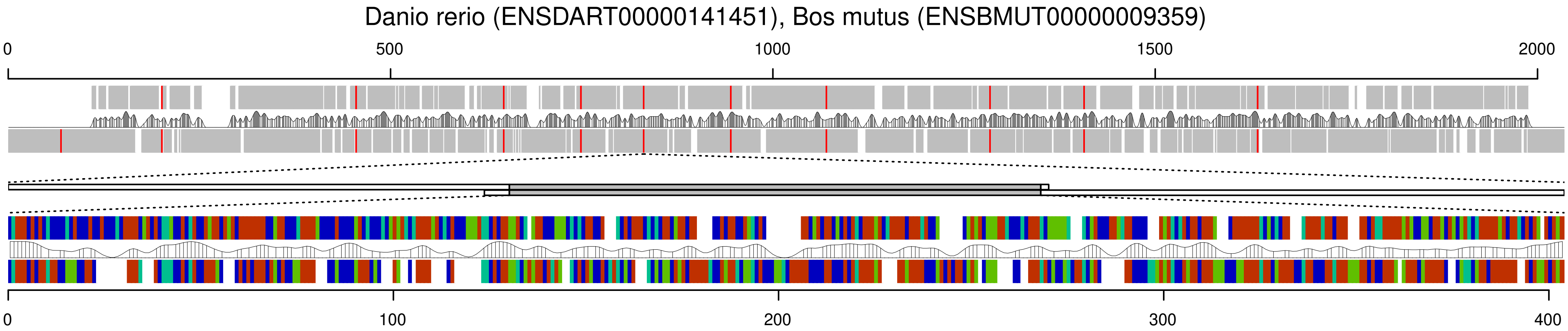
Danio rerio (ENSDART00000141451), Bos mutus (ENSBMUT00000009359)
0
500
1000
1500
2000
0
100
200
300
400

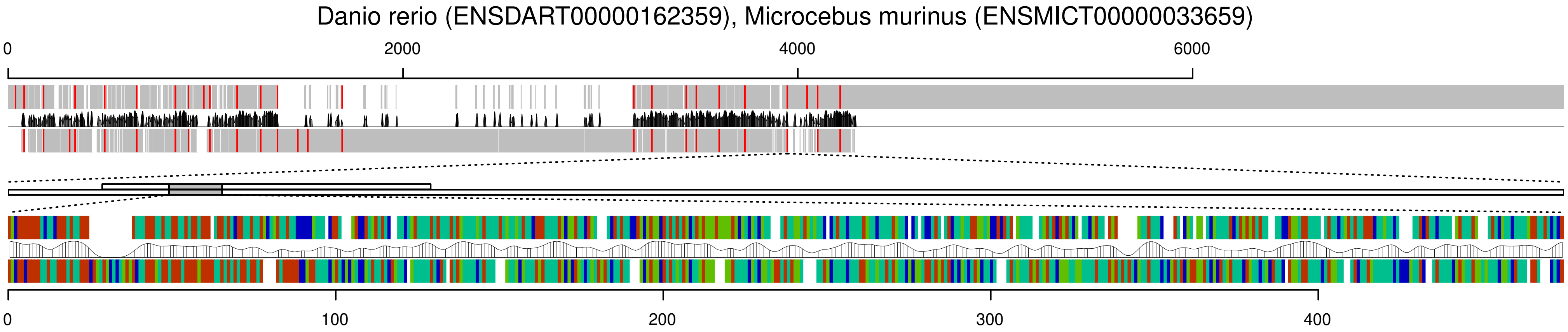
Danio rerio (ENSDART00000162359), Microcebus murinus (ENSMICT00000033659)
0
2000
4000
6000
0
100
200
300
400

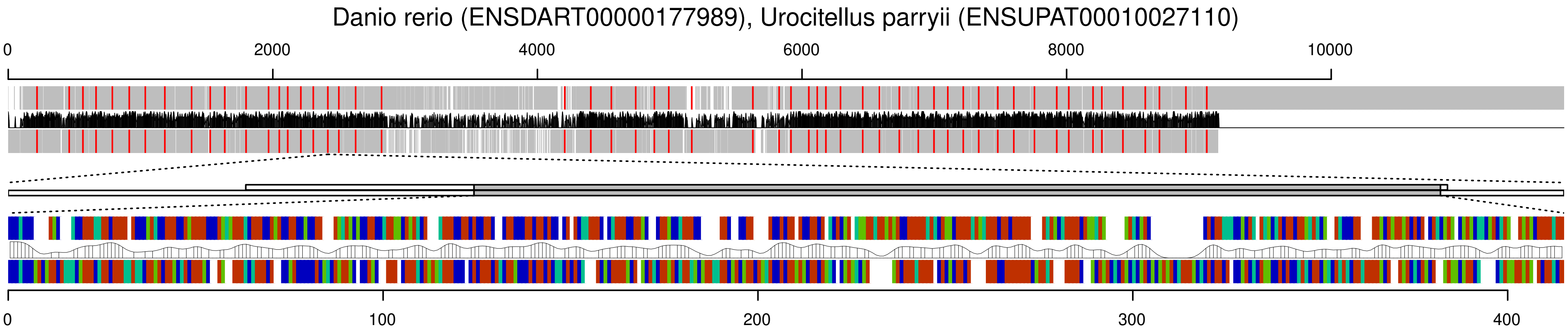
Danio rerio (ENSDART00000177989), Urocitellus parryii (ENSUPAT00010027110)
0
2000
4000
6000
8000
10000
0
100
200
300
400

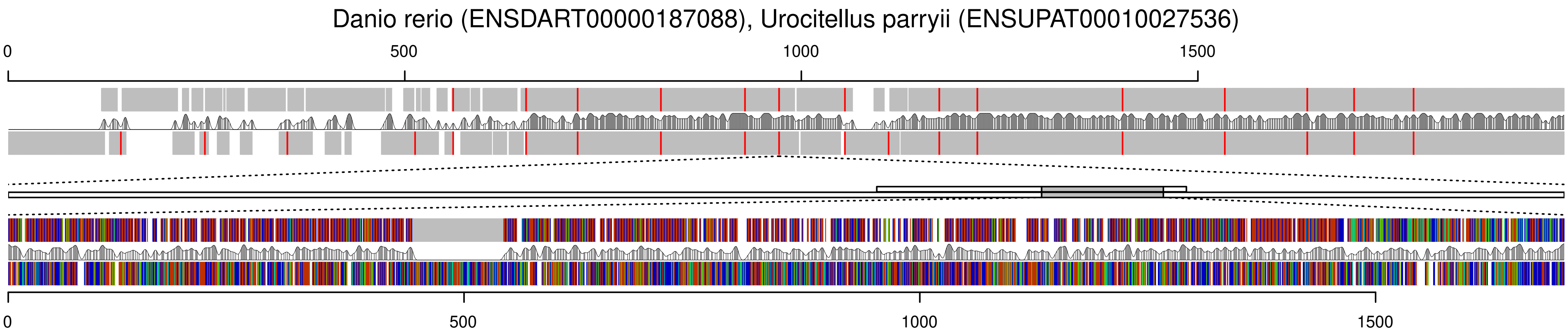
Danio rerio (ENSDART00000187088), Urocitellus parryii (ENSUPAT00010027536)
0 500 1000 1500
0 500 1000 1500

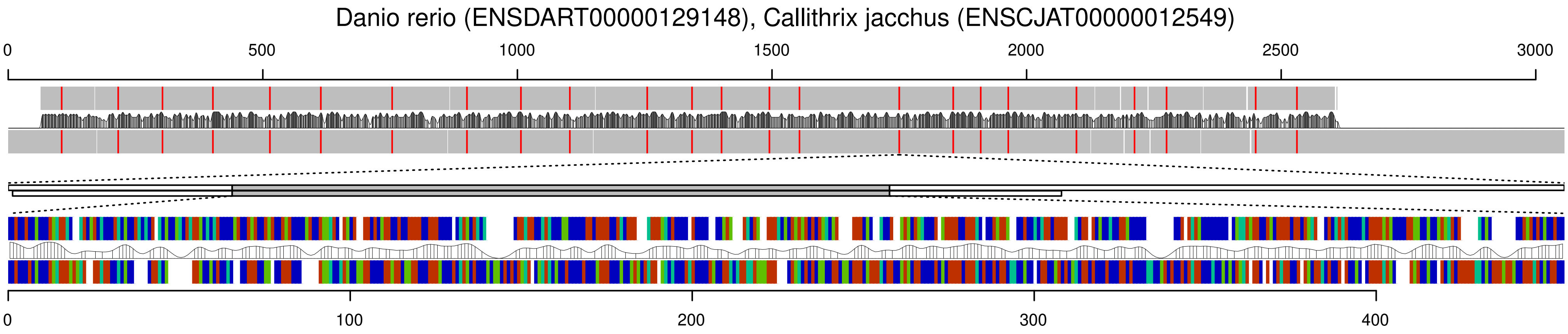
Danio rerio (ENSDART00000129148), Callithrix jacchus (ENSCJAT00000012549)
0 500 1000 1500 2000 2500 3000
0 100 200 300 400

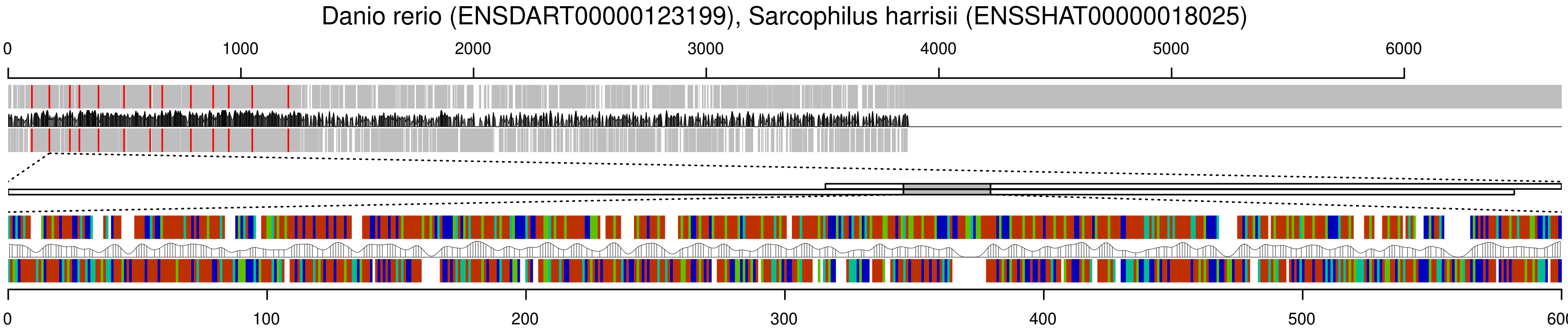
Danio rerio (ENSDART00000123199), Sarcophilus harrisii (ENSSHAT00000018025)
0 1000 2000 3000 4000 5000 6000
0 100 200 300 400 500 600

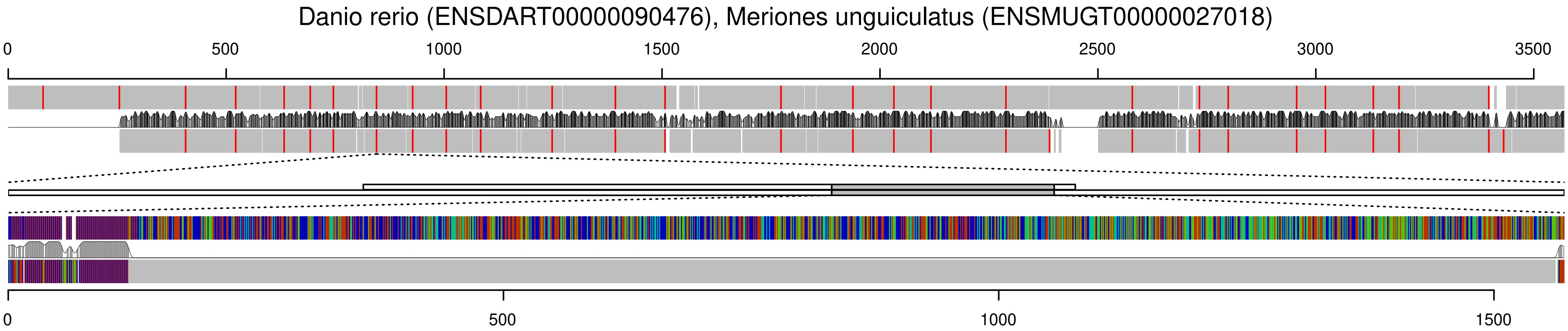
Danio rerio (ENSDART00000090476), Meriones unguiculatus (ENSMUGT00000027018)
0 500 1000 1500 2000 2500 3000 3500
0 500 1000 1500

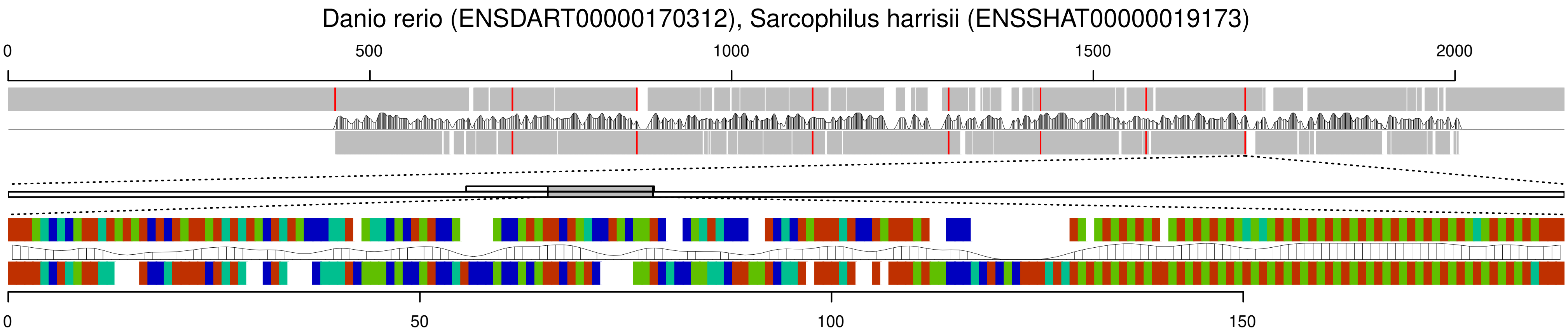
Danio rerio (ENSDART00000170312), Sarcophilus harrisii (ENSSHAT00000019173)
0 500 1000 1500 2000
0 50 100 150

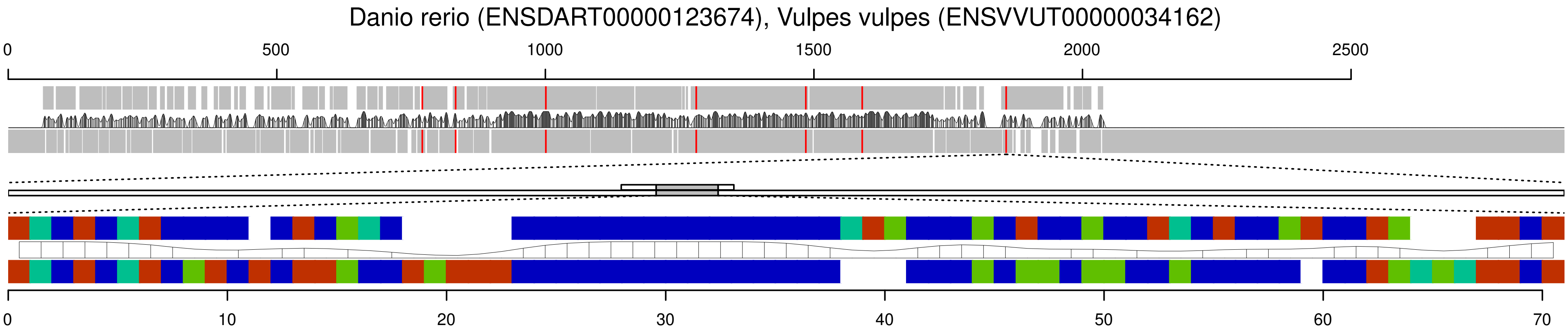
Danio rerio (ENSDART00000123674), Vulpes vulpes (ENSVVUT00000034162)
0 500 1000 1500 2000 2500
0 10 20 30 40 50 60 70

Danio rerio (ENSDART00000089622), Phascolarctos cinereus (ENSPCIT00000051240)

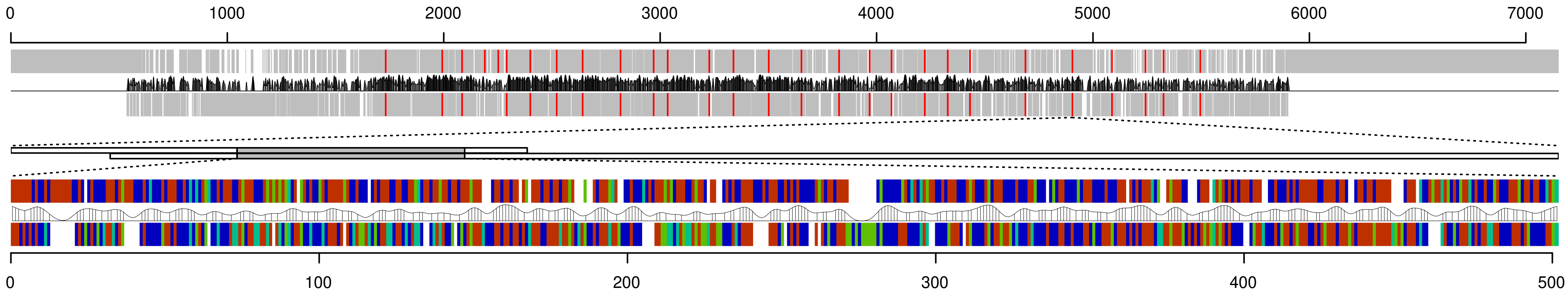

Danio rerio (ENSDART00000026635), Pan paniscus (ENSPPAT00000055016)

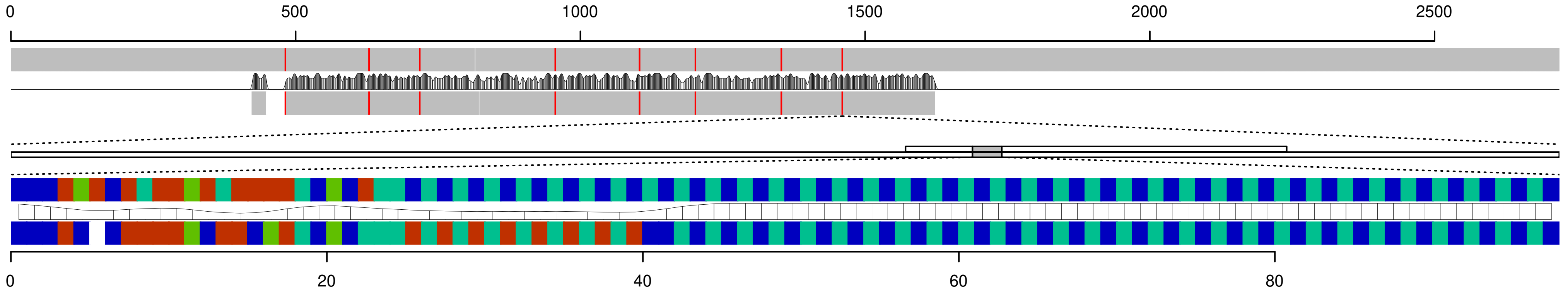

Danio rerio (ENSDART00000153364), Sarcophilus harrisii (ENSSHAT00000017290)

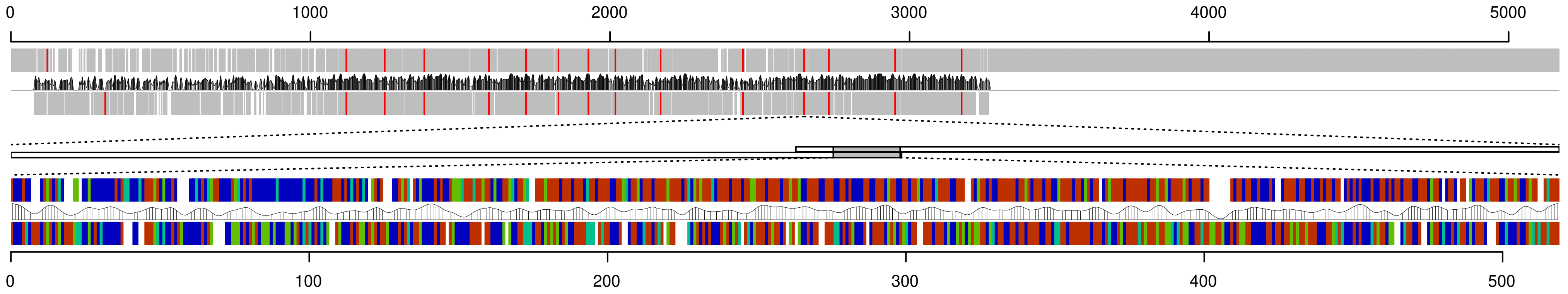

Danio rerio (ENSDART00000046274), Notamacropus eugenii (ENSMEUT00000011721)

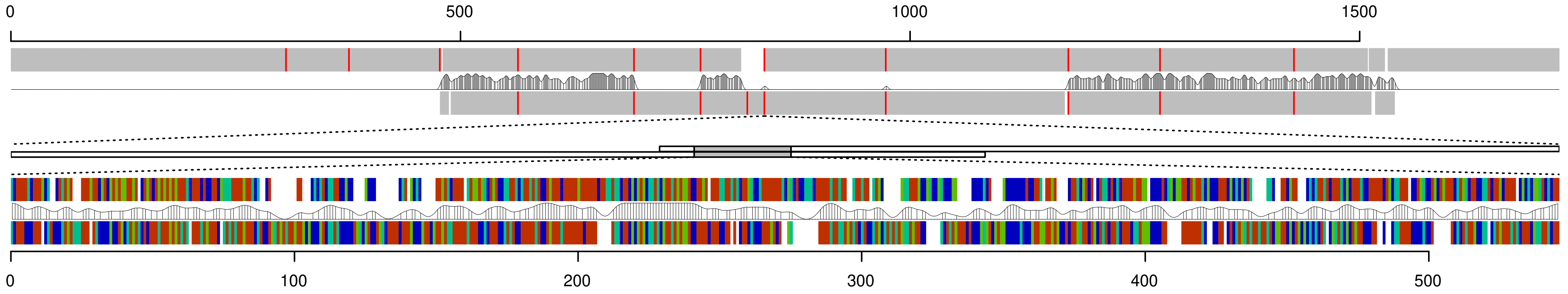

Danio rerio (ENSDART00000014232), Piliocolobus tephrosceles (ENSPTET00000056724)

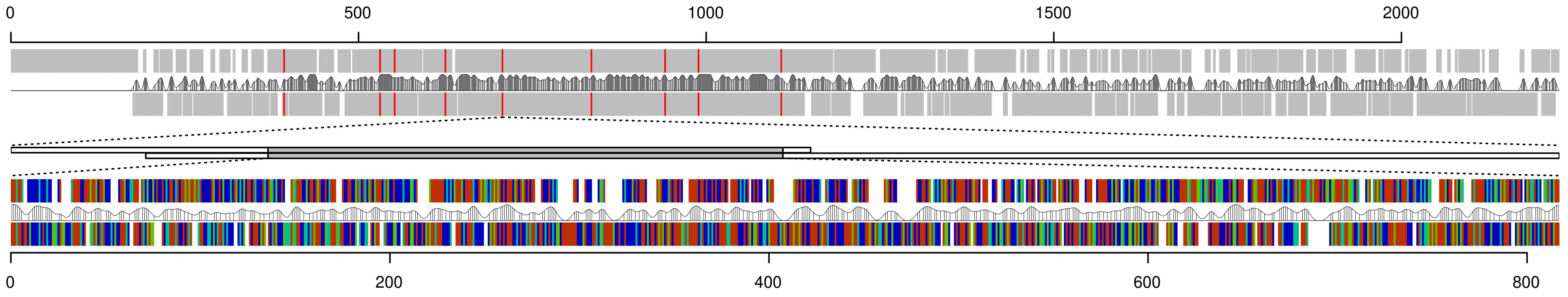

Danio rerio (ENSDART00000148363), Cebus capucinus (ENSCCAT00000026070)

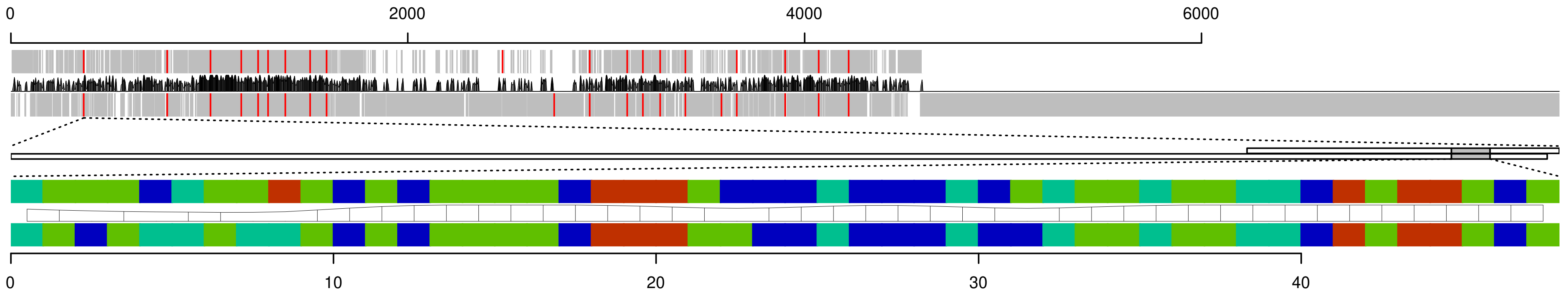

Danio rerio (ENSDART00000135985), Vicugna pacos (ENSVPAT00000003433)

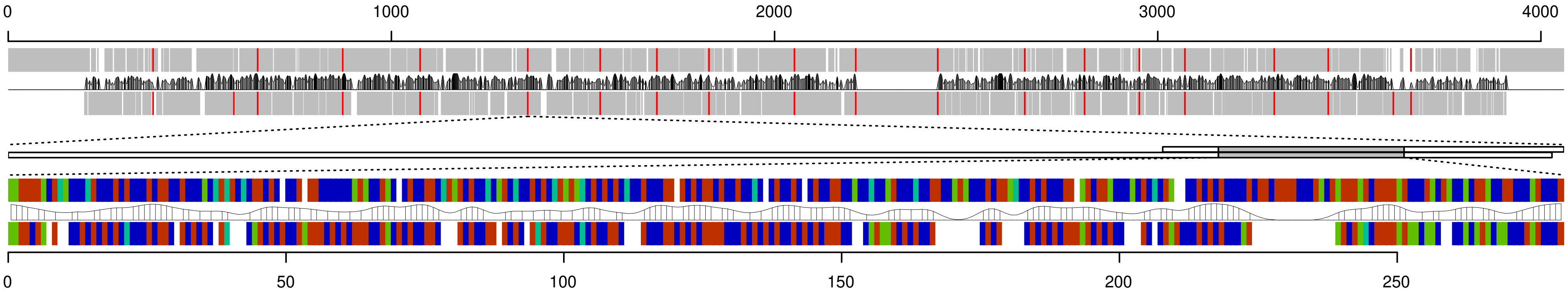

Danio rerio (ENSDART00000125423), Pan paniscus (ENSPPAT00000008956)

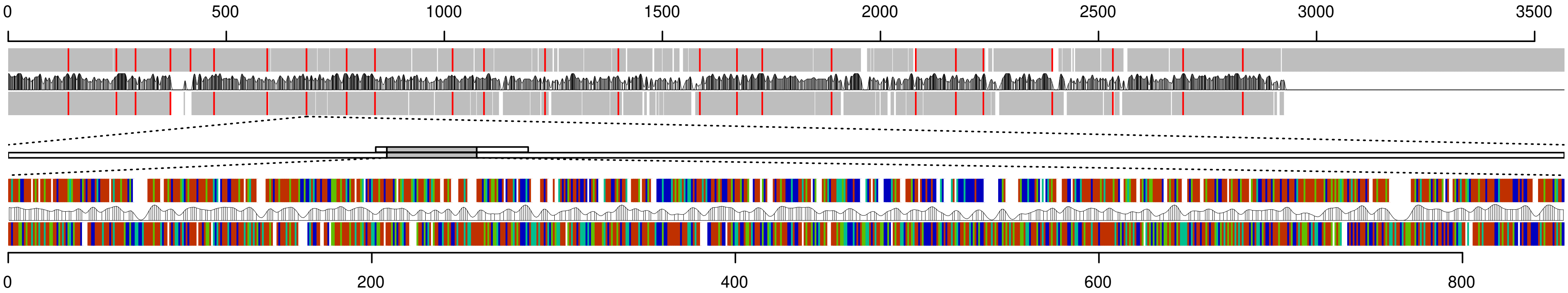

Danio rerio (ENSDART00000009464), Vombatus ursinus (ENSVURT00010002274)

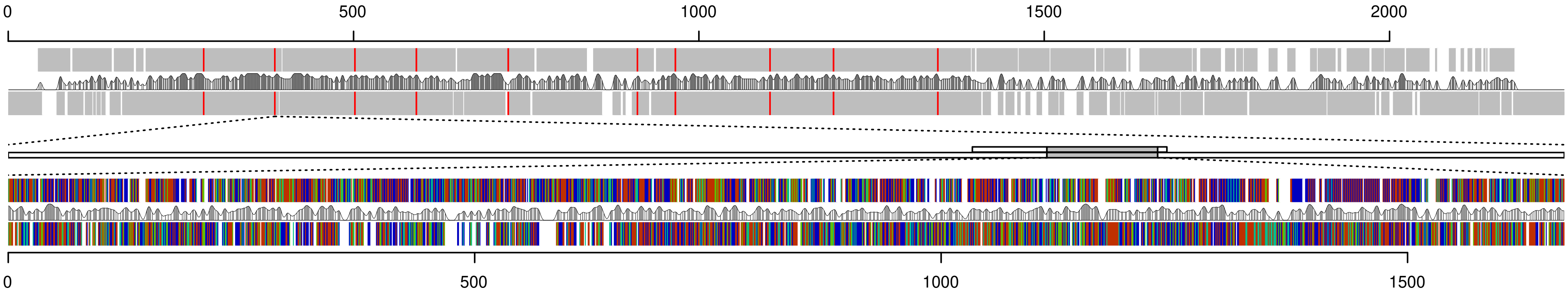

Danio rerio (ENSDART00000110411), Felis catus (ENSFCAT00000055766)

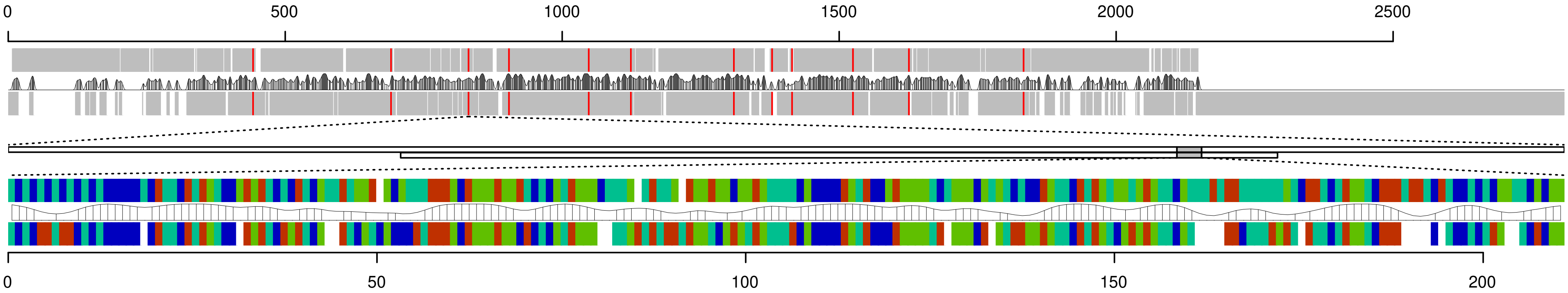

Danio rerio (ENSDART00000085387), Monodelphis domestica (ENSMODT00000003255)

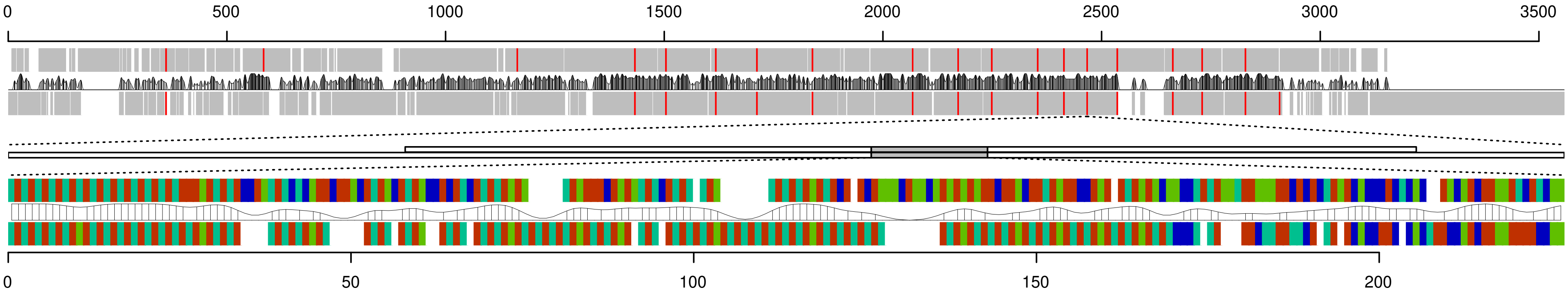

Danio rerio (ENSDART00000077612), Pteropus vampyrus (ENSPVAT00000000364)

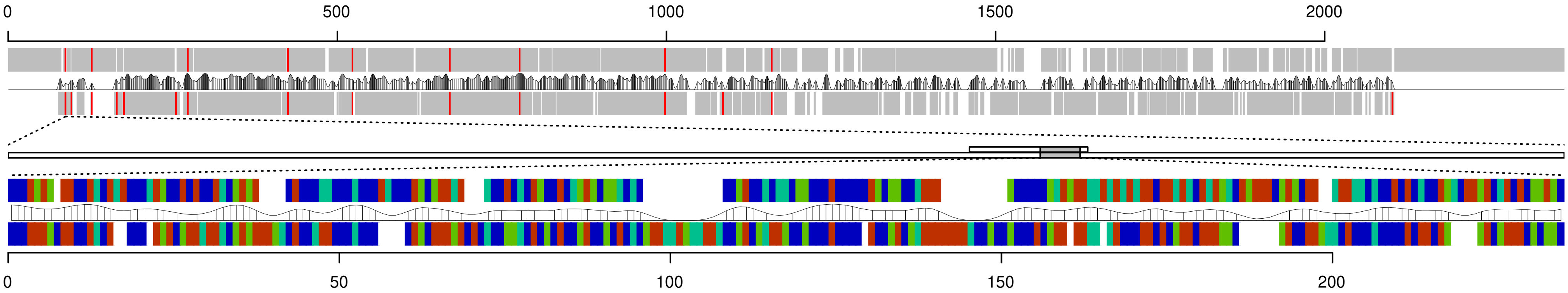

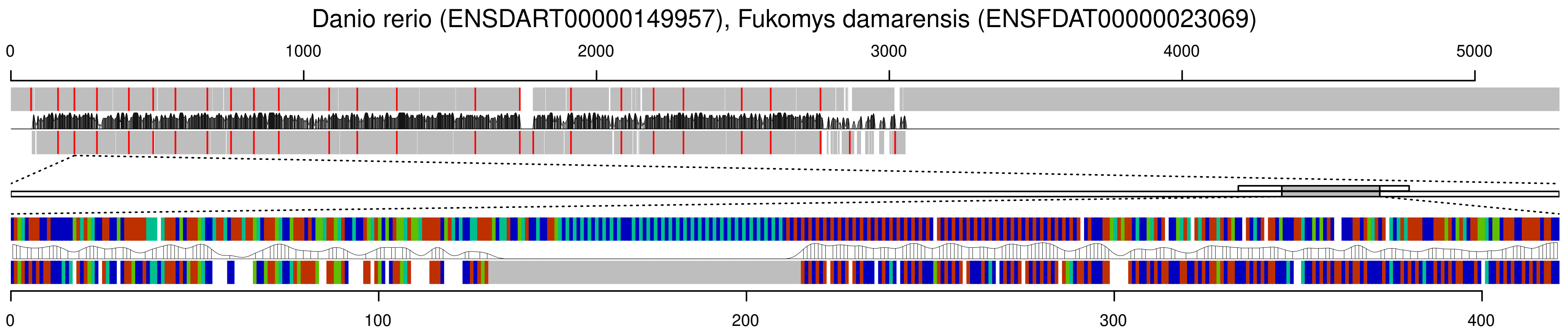
Danio rerio (ENSDART00000149957), Fukomys damarensis (ENSFDAT00000023069)
0
1000
2000
3000
4000
5000
0
100
200
300
400

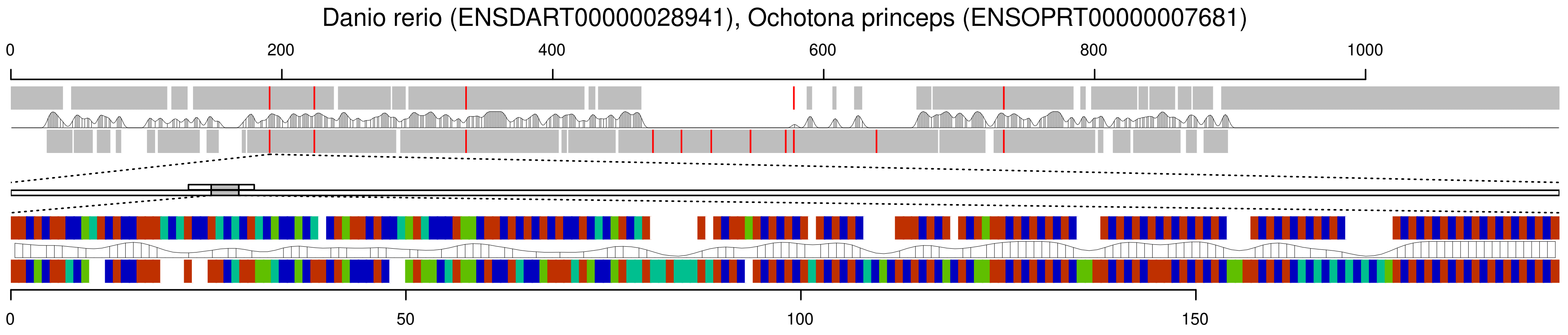
Danio rerio (ENSDART00000028941), Ochotona princeps (ENSOPRT00000007681)
0
200
400
600
800
1000
0
50
100
150

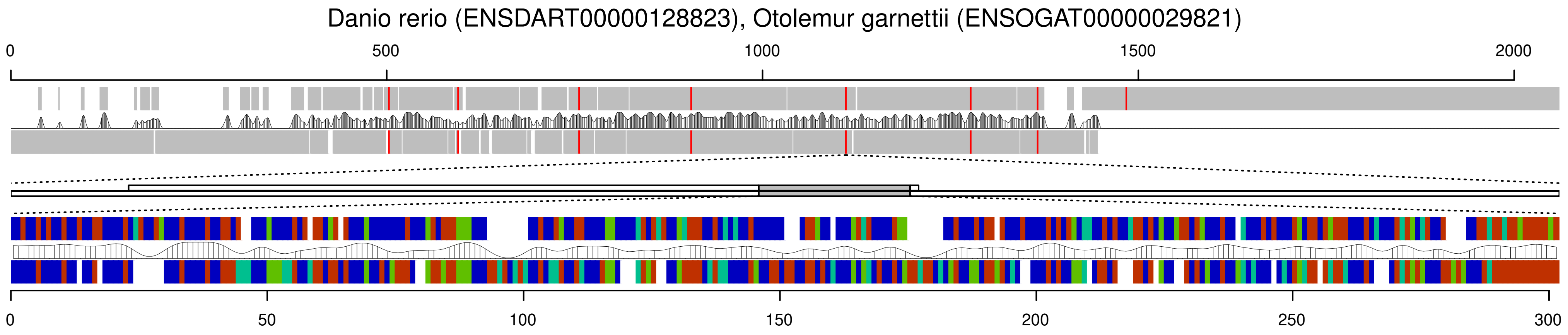
Danio rerio (ENSDART00000128823), Otolemur garnettii (ENSOGAT00000029821)
0
500
1000
1500
2000
0
50
100
150
200
250
300

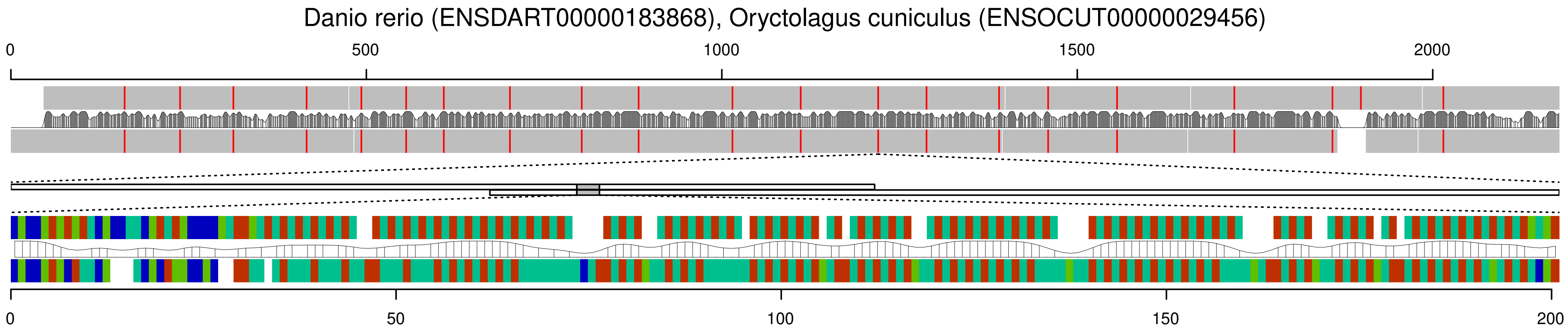
Danio rerio (ENSDART00000183868), Oryctolagus cuniculus (ENSOCUT00000029456)
0
500
1000
1500
2000
0
50
100
150
200

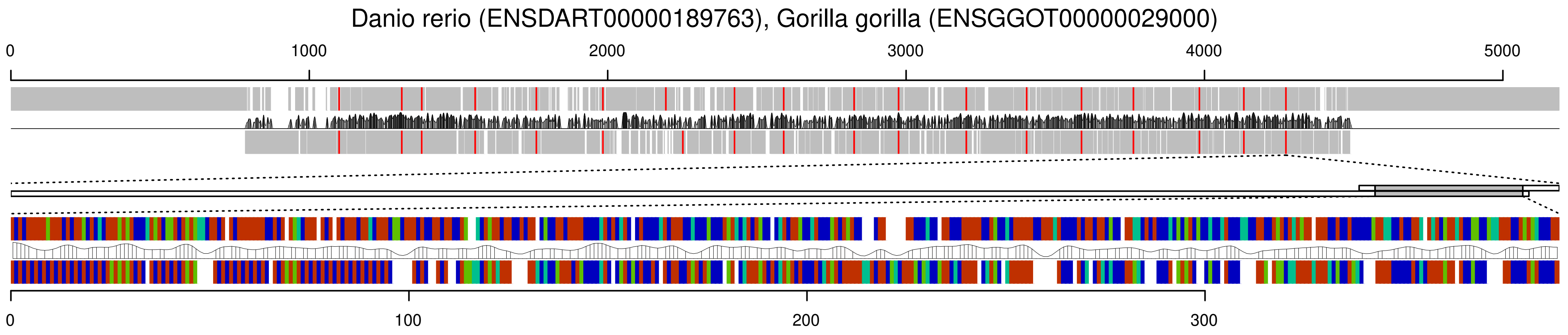
Danio rerio (ENSDART00000189763), Gorilla gorilla (ENSGGOT00000029000)
0
1000
2000
3000
4000
5000
0
100
200
300

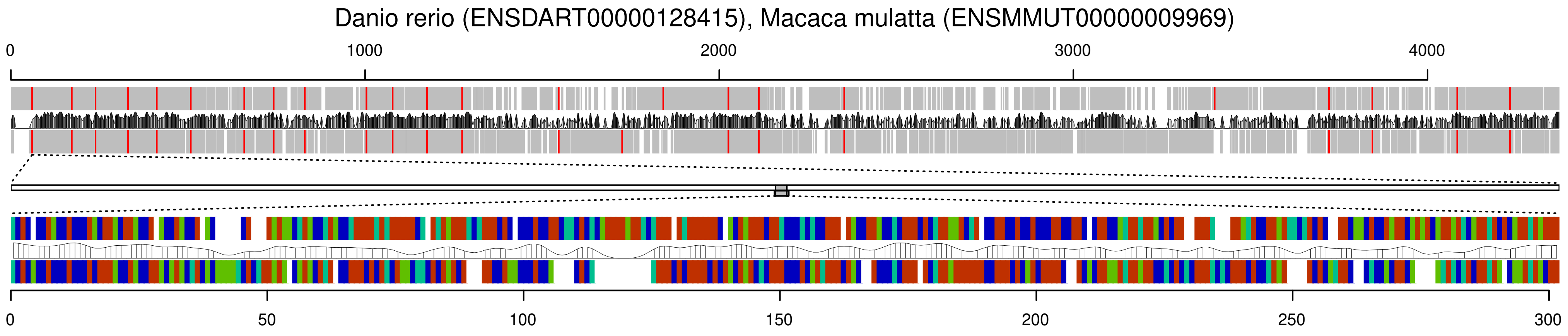
Danio rerio (ENSDART00000128415), Macaca mulatta (ENSMMUT00000009969)
0
1000
2000
3000
4000
0
50
100
150
200
250
300

Danio rerio (ENSDART00000086495), Equus caballus (ENSECAT00000042153)

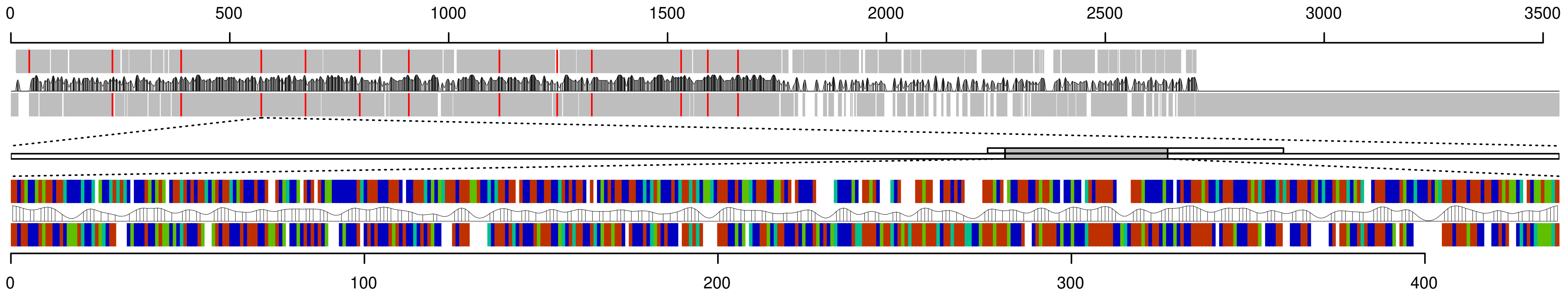

Danio rerio (ENSDART00000158495), Panthera pardus (ENSPPRT00000001891)

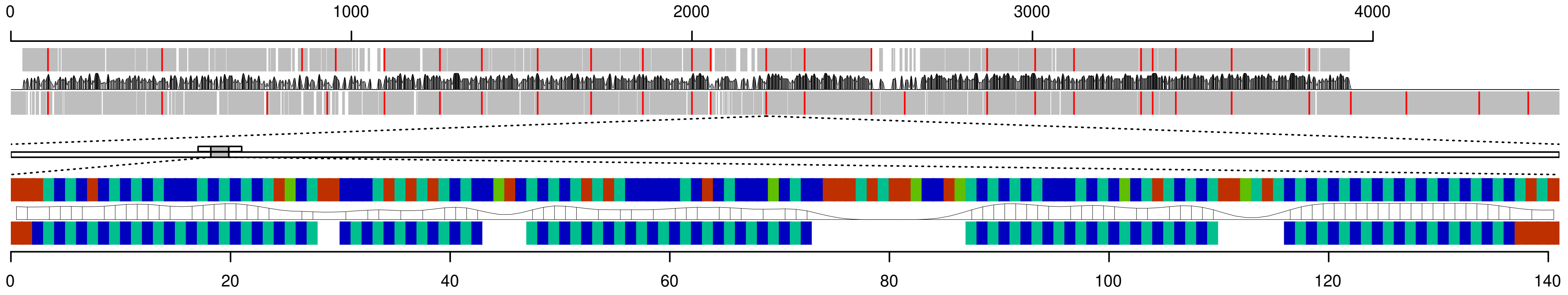

Danio rerio (ENSDART00000165318), Cebus capucinus (ENSCCAT00000026008)

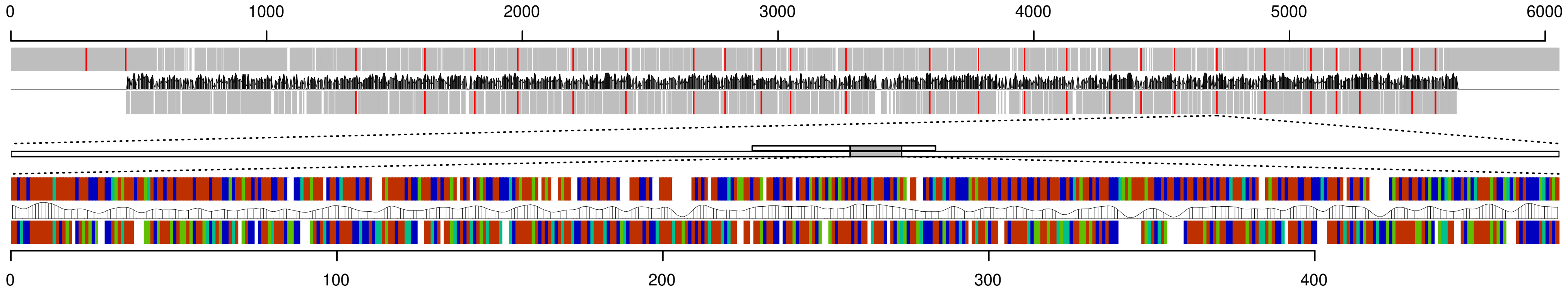

Danio rerio (ENSDART00000145596), Mus spicilegus (ENSMSIT00000040300)

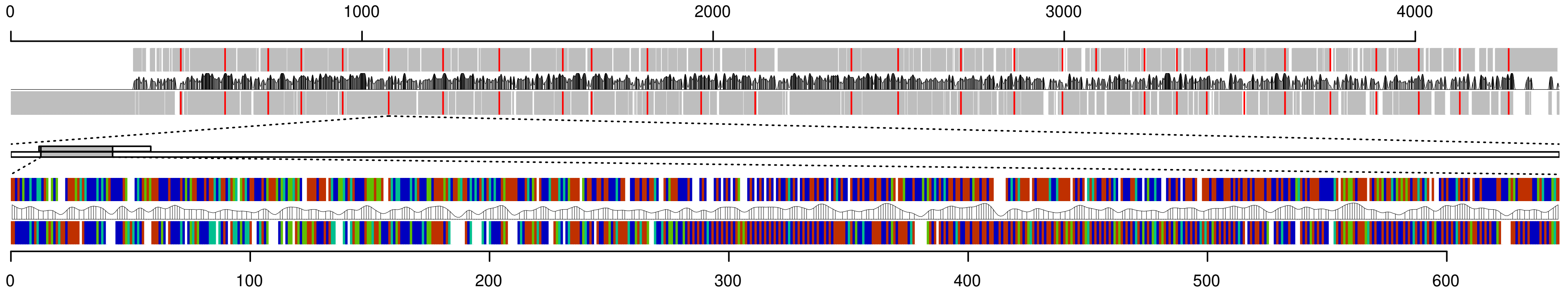

Danio rerio (ENSDART00000135384), Bos taurus (ENSBTAT00000050390)

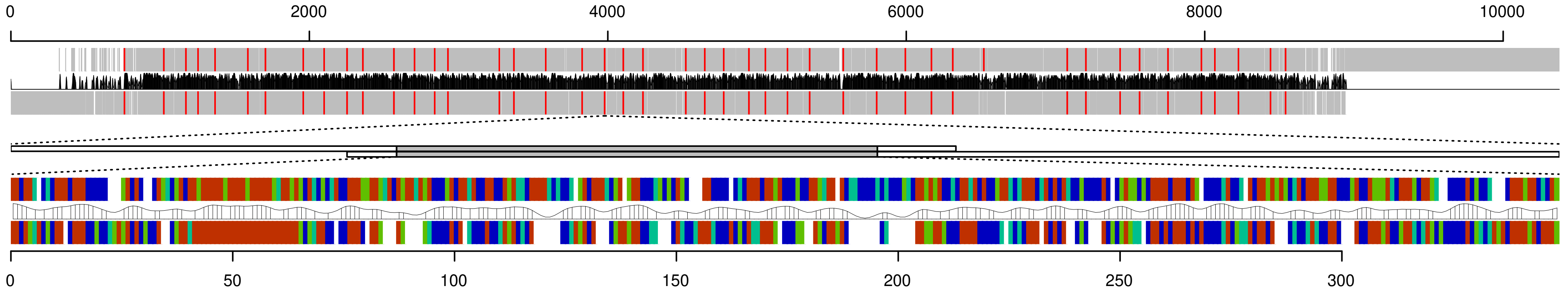

Danio rerio (ENSDART00000084282), Monodelphis domestica (ENSMODT00000016589)

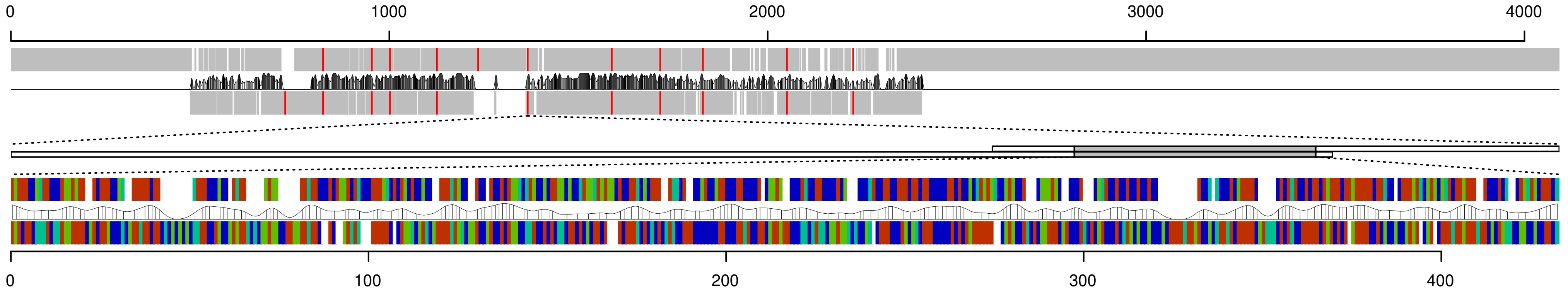

Danio rerio (ENSDART00000089589), Octodon degus (ENSODET00000019016)

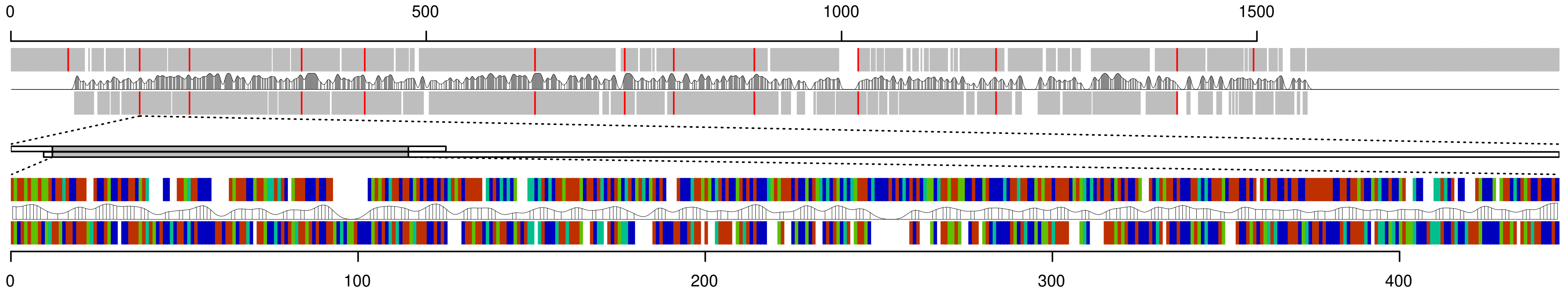

Danio rerio (ENSDART00000125173), Bos taurus (ENSBTAT00000027486)

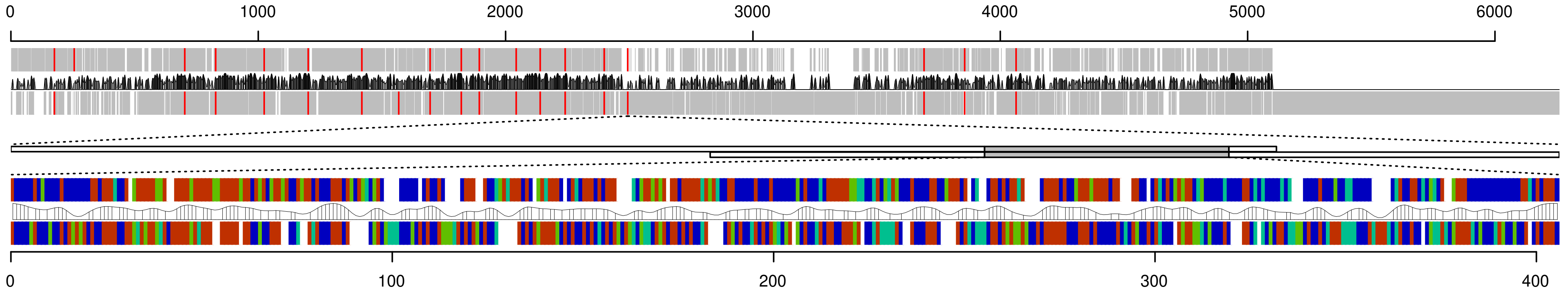

Danio rerio (ENSDART00000191475), Myotis lucifugus (ENSMLUT00000013379)

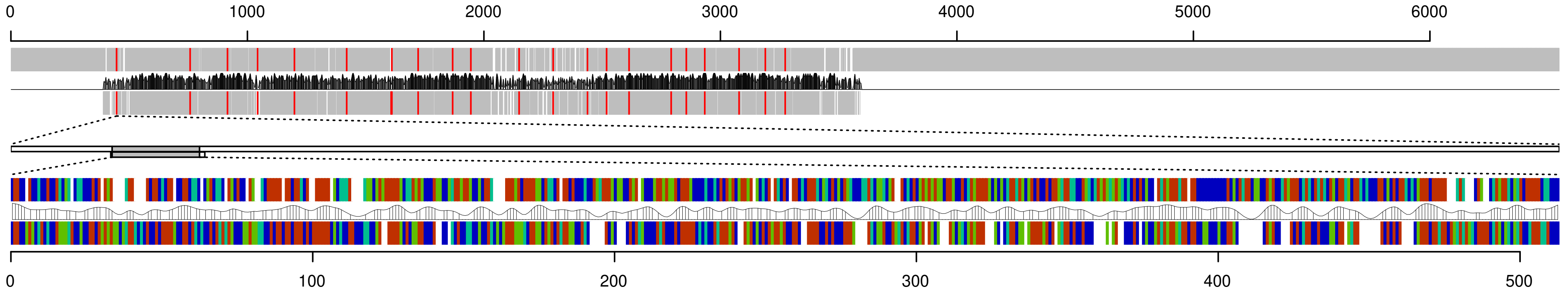

Danio rerio (ENSDART00000021575), Phascolarctos cinereus (ENSPCIT00000017637)

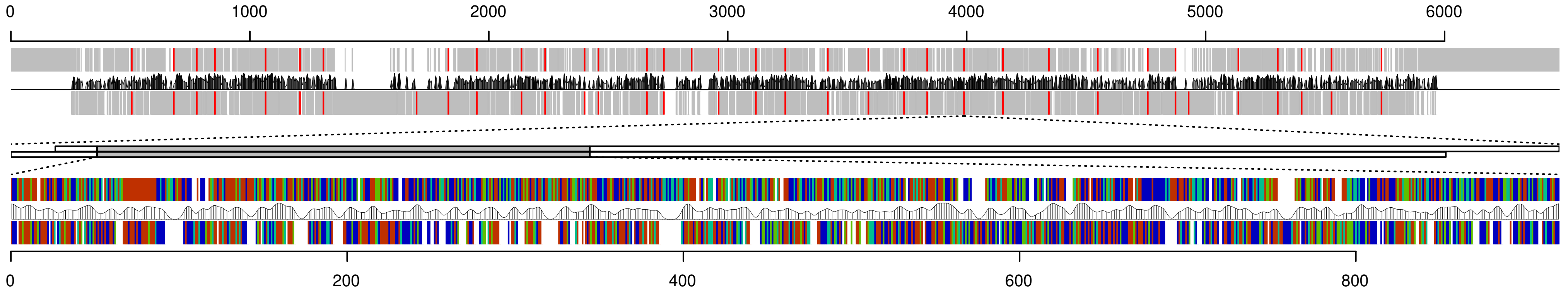

Danio rerio (ENSDART00000079341), Mus musculus (ENSMUST00000084105)

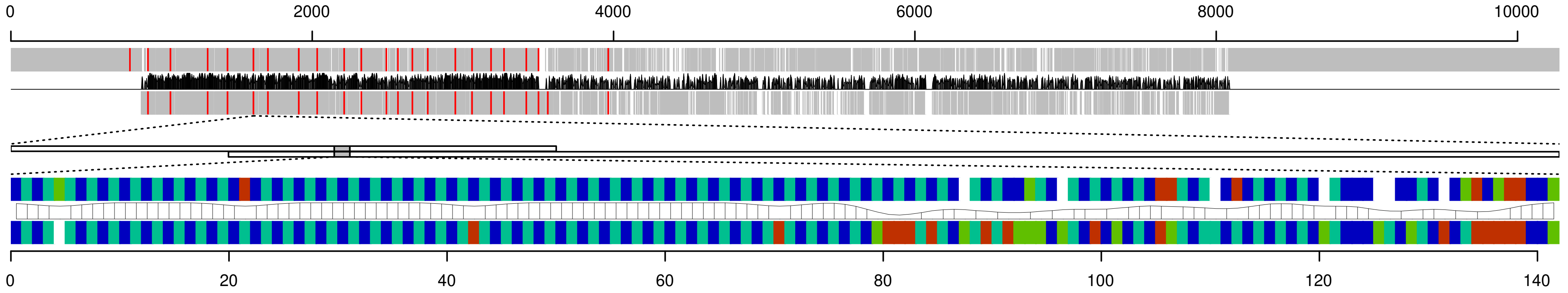

Danio rerio (ENSDART00000121885), Myotis lucifugus (ENSMLUT00000015004)

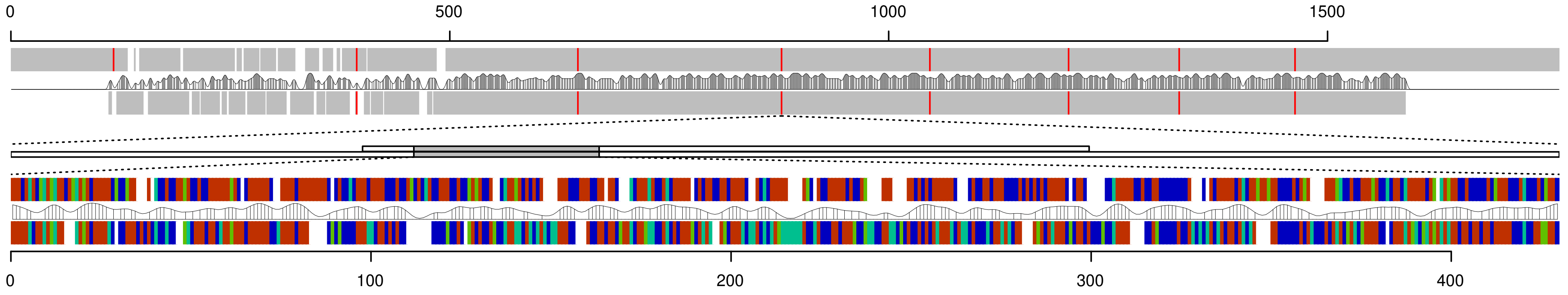

Danio rerio (ENSDART00000186891), Sarcophilus harrisii (ENSSHAT00000006402)

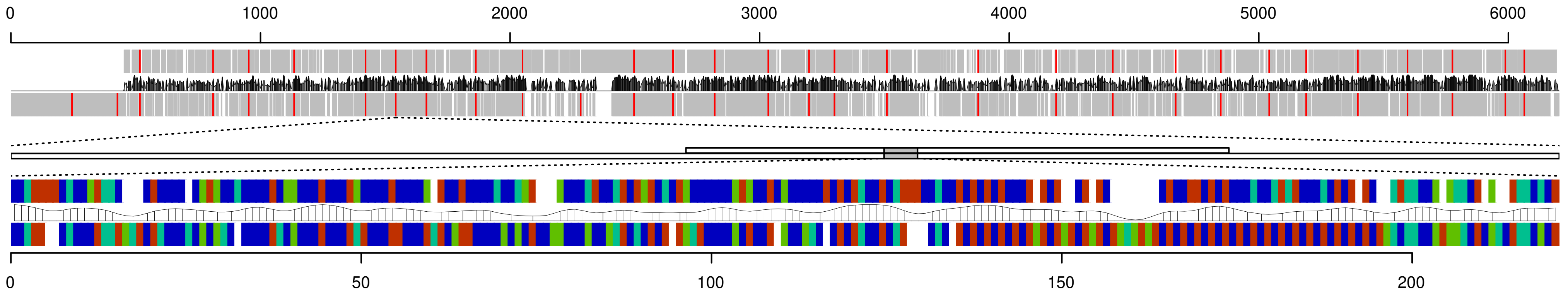

Danio rerio (ENSDART00000111856), Mesocricetus auratus (ENSMAUT00000023686)

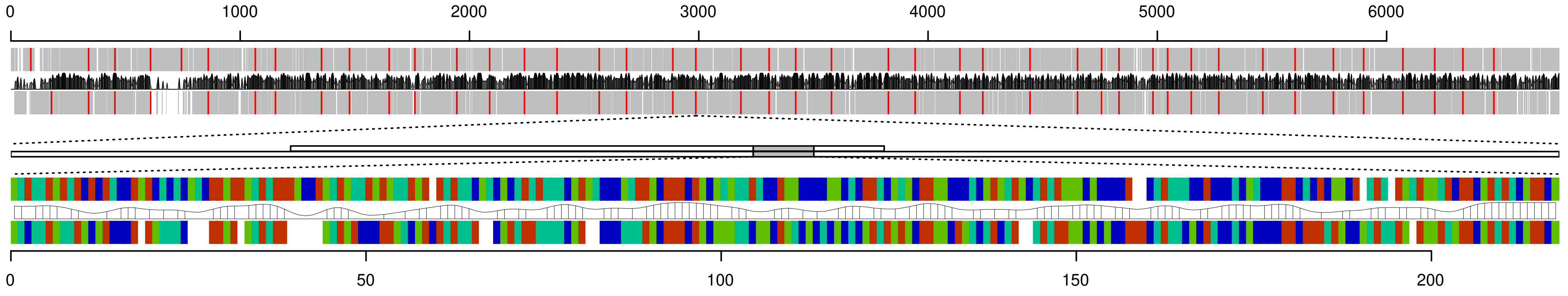

Danio rerio (ENSDART00000092773), Octodon degus (ENSODET00000022325)

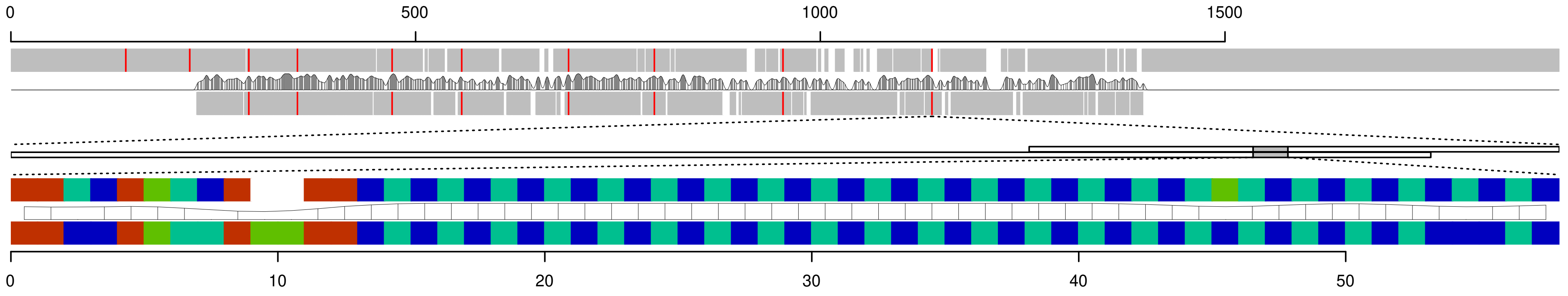

Danio rerio (ENSDART00000135074), Felis catus (ENSFCAT00000042485)

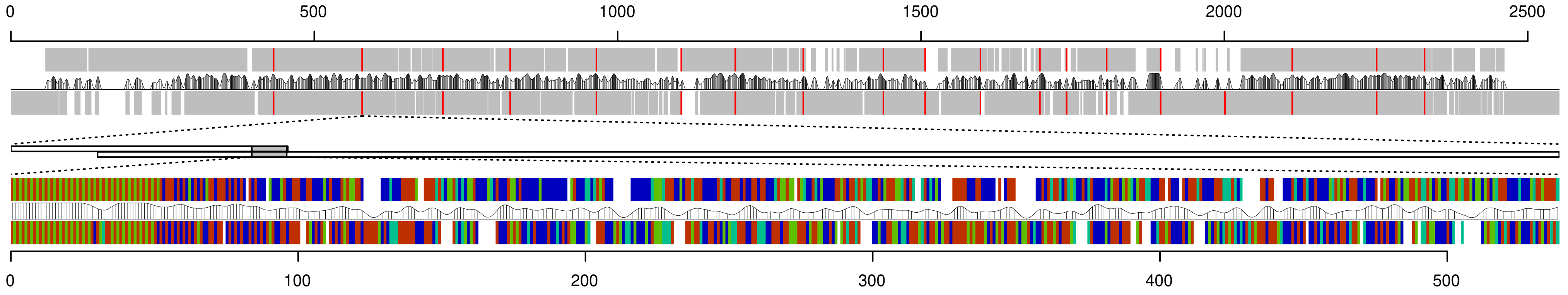

Danio rerio (ENSDART00000104762), Ictidomys tridecemlineatus (ENSSTOT00000026993)

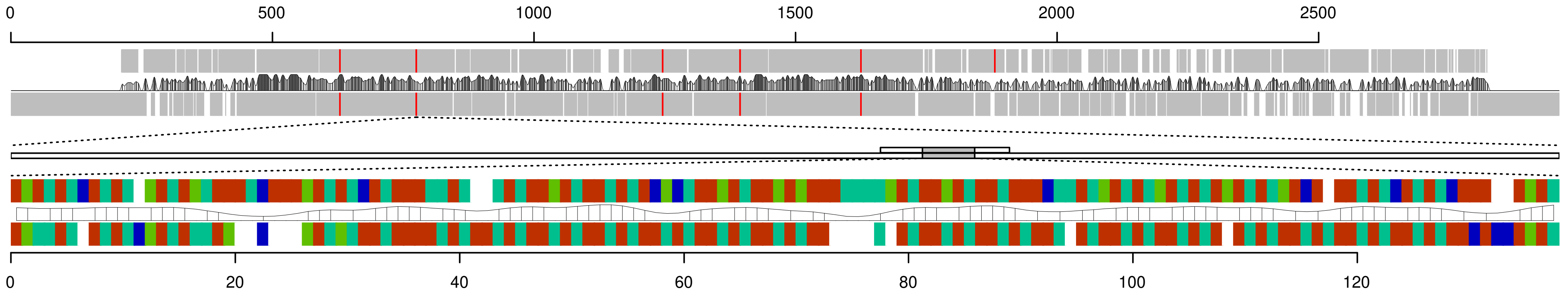

Danio rerio (ENSDART00000100622), Neovison vison (ENSNVIT00000021869)

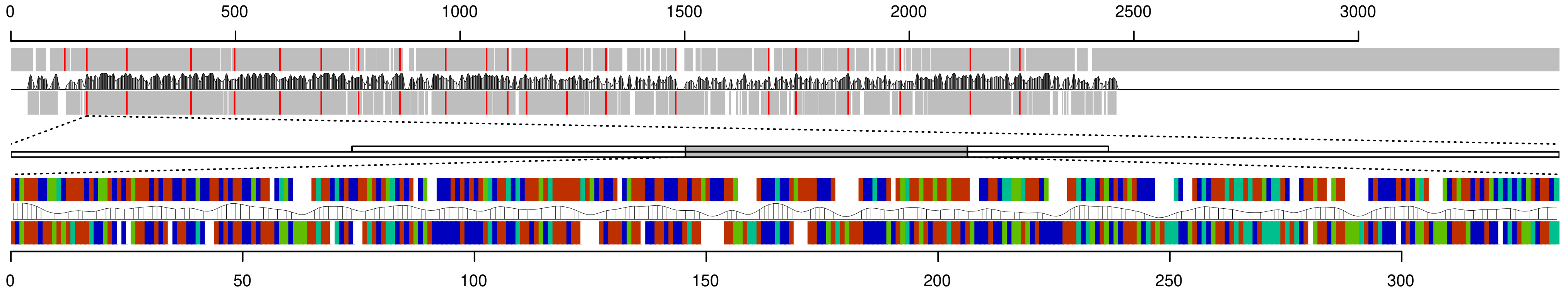

Supplement: Supplementary file 6 — Transcript and intron alignments for points in Fig. S14. Each panel shows the maximally scoring alignment between D. rerio and teleost intron orthologues (lower) and transcript alignment (upper) used to establish the intron orthology. Grey, white and red parts indicate aligned exonic sequence, gaps and positions of intron meta-characters respectively. Colours in intron alignment represent bases (A blue, C cyan, G green, T brown, N grey, gap white). Curves lying between sequence representations show a normal kernel density smoothed estimate of local similarity (9 bp window, standard deviation two); vertical lines indicate matches. Region between exon and intron alignments indicates the location of the maximally scoring alignment in the introns. Upper sequence D. rerio. Files 6–10 and 11–15 contain alignments to teleost and mammalian sequences respectively. Each file corresponds to one panel in Fig. S17 and to one specific teleost size class: Files 6,11: long (E,J), 7,12: medium (D,I), 8,13: short.2 (C,H), 9,14 short (B, G) and 10,15 ctl (A,F). [file 12864_2022_8760_MOESM6_ESM.zip › 12864_2022_8760_MOESM14_ESM.pdf]
