# Supplementary material for: Intron size minimisation in teleosts
Source: BMC Genomics. 2022 Sep 1;23:628. doi: 10.1186/s12864-022-08760-w (PMC9438311; doi:10.1186/s12864-022-08760-w)
Supplement: Supplementary file 6 — Transcript and intron alignments for points in Fig. S14. Each panel shows the maximally scoring alignment between D. rerio and teleost intron orthologues (lower) and transcript alignment (upper) used to establish the intron orthology. Grey, white and red parts indicate aligned exonic sequence, gaps and positions of intron meta-characters respectively. Colours in intron alignment represent bases (A blue, C cyan, G green, T brown, N grey, gap white). Curves lying between sequence representations show a normal kernel density smoothed estimate of local similarity (9 bp window, standard deviation two); vertical lines indicate matches. Region between exon and intron alignments indicates the location of the maximally scoring alignment in the introns. Upper sequence D. rerio. Files 6–10 and 11–15 contain alignments to teleost and mammalian sequences respectively. Each file corresponds to one panel in Fig. S17 and to one specific teleost size class: Files 6,11: long (E,J), 7,12: medium (D,I), 8,13: short.2 (C,H), 9,14 short (B, G) and 10,15 ctl (A,F). [file 12864_2022_8760_MOESM6_ESM.zip › 12864_2022_8760_MOESM13_ESM.pdf]

Danio rerio (ENSDART00000010378), Dipodomys ordii (ENSDORT00000012605)

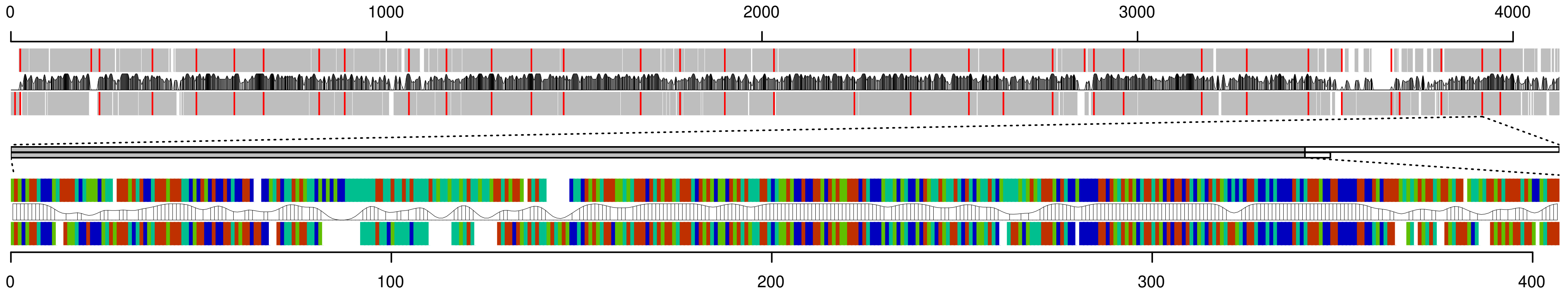

Danio rerio (ENSDART00000025031), Phascolarctos cinereus (ENSPCIT00000060434)

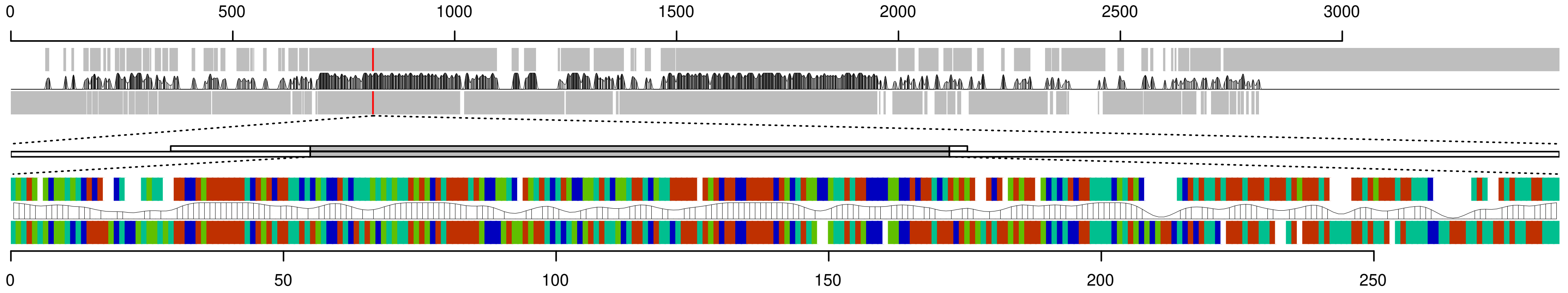

Danio rerio (ENSDART00000134547), Gorilla gorilla (ENSGGOT00000005206)

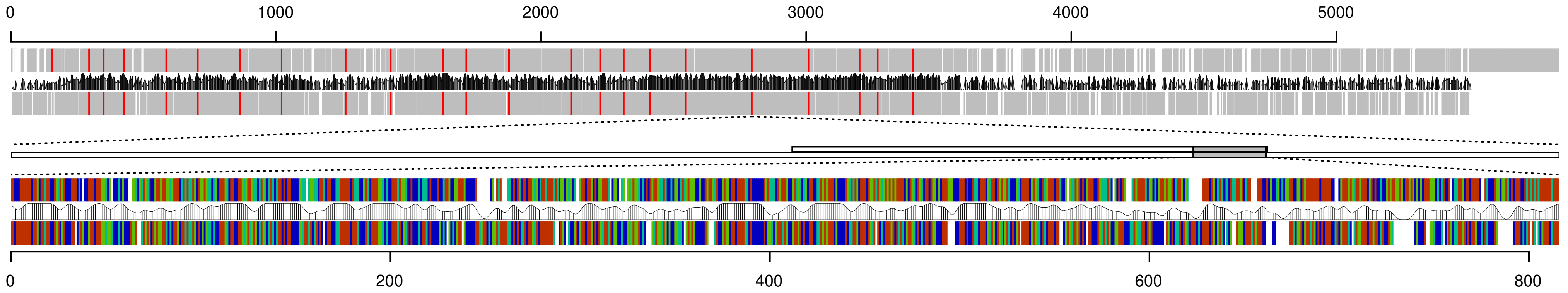

Danio rerio (ENSDART00000149175), Monodelphis domestica (ENSMODT00000012920)

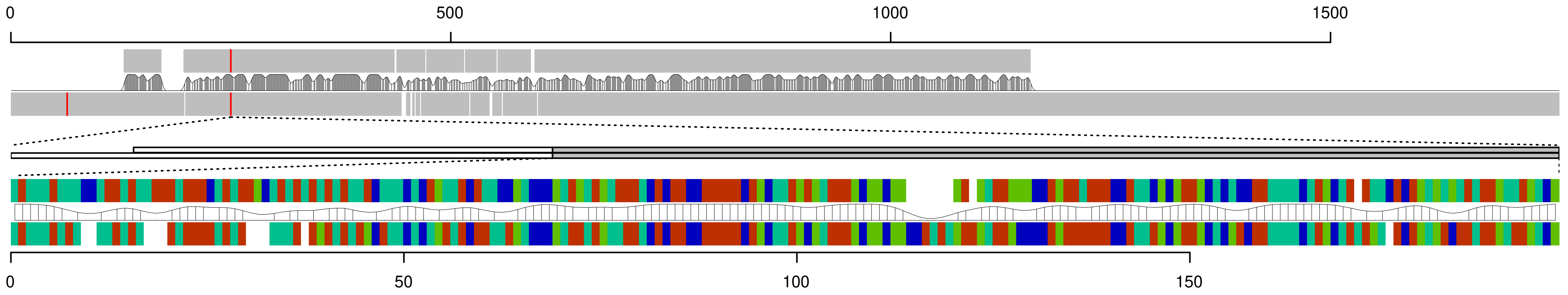

Danio rerio (ENSDART00000170482), Erinaceus europaeus (ENSEEUT00000008321)

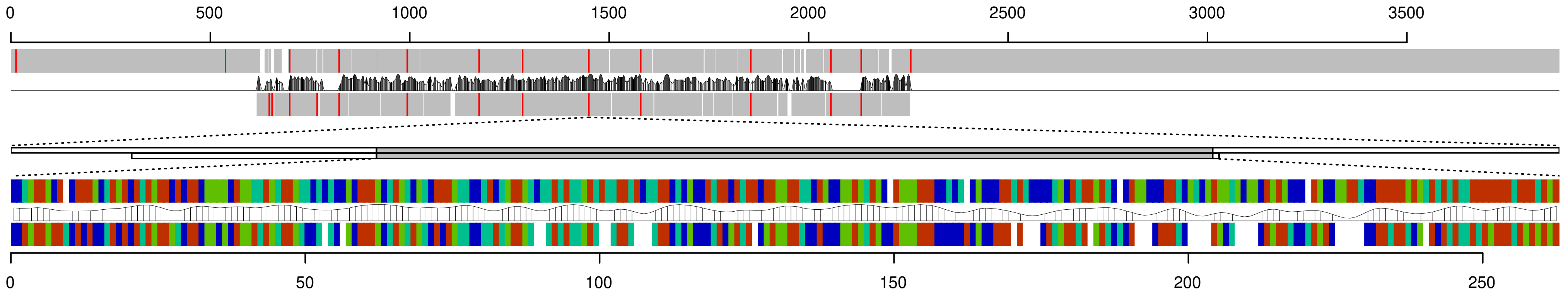

Danio rerio (ENSDART00000055395), Fukomys damarensis (ENSFDAT00000007673)

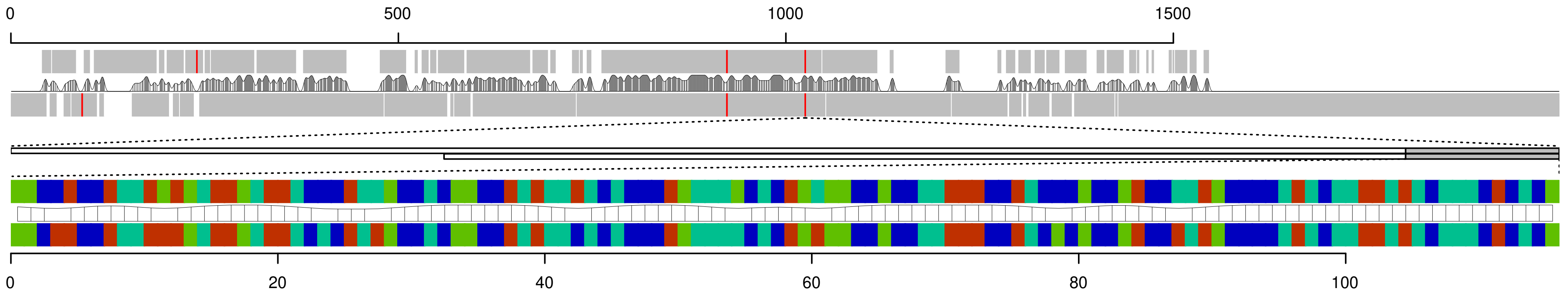

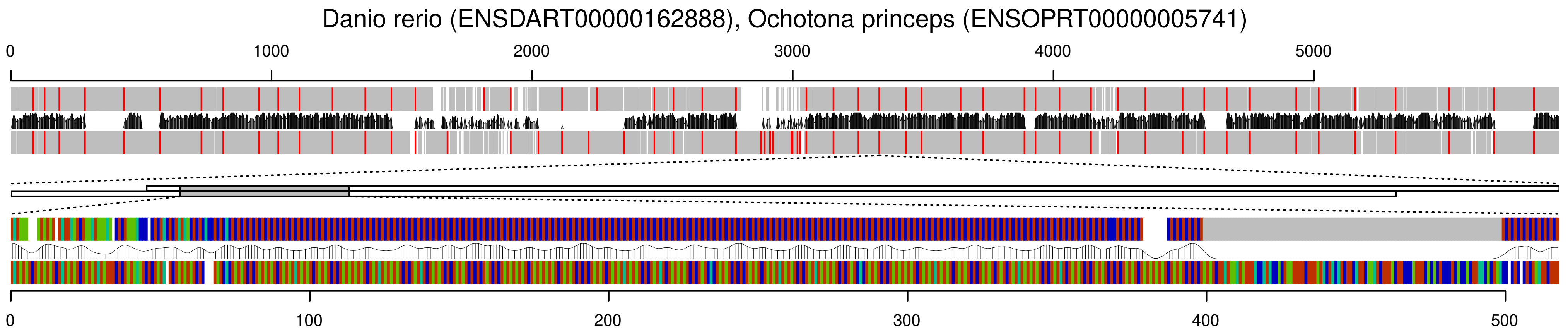
Danio rerio (ENSDART00000162888), Ochotona princeps (ENSOPRT00000005741)
0 1000 2000 3000 4000 5000
0 100 200 300 400 500

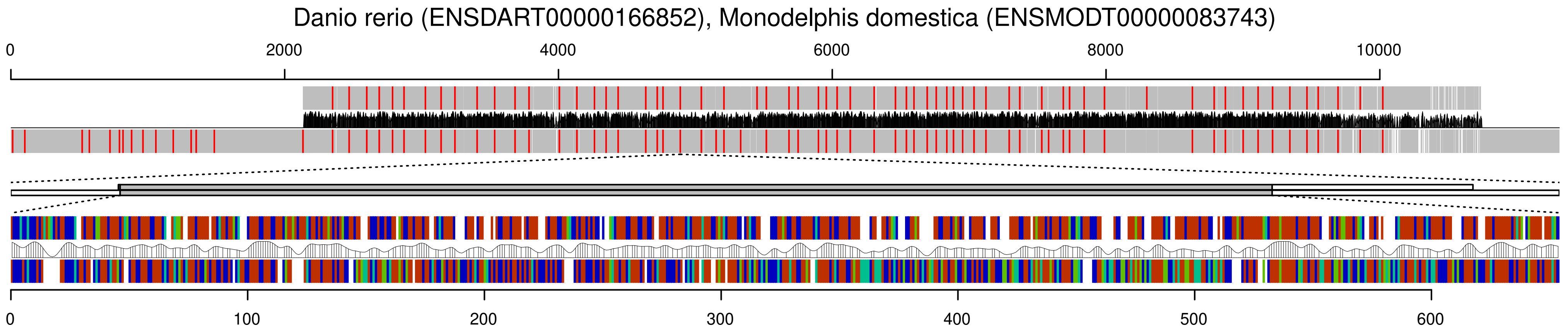
Danio rerio (ENSDART00000166852), Monodelphis domestica (ENSMODT00000083743)
0 2000 4000 6000 8000 10000
0 100 200 300 400 500 600

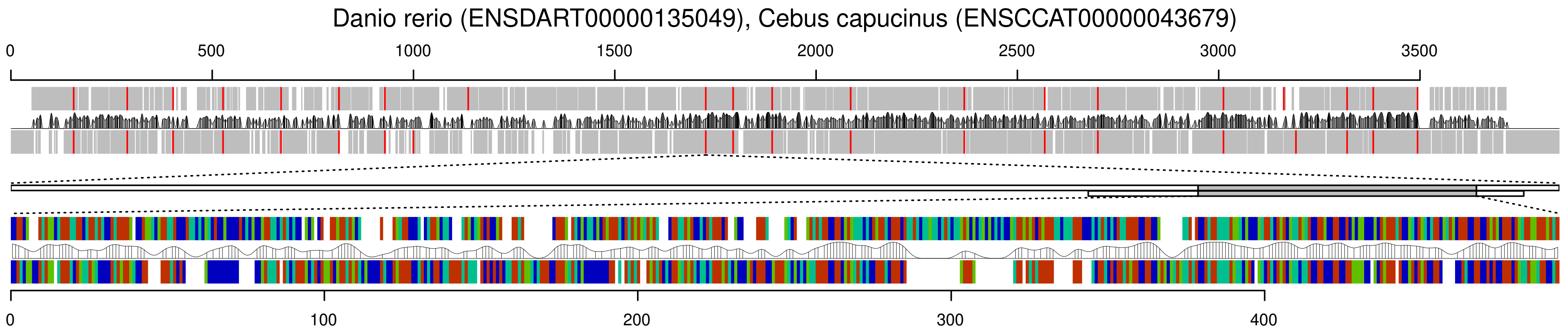
Danio rerio (ENSDART00000135049), Cebus capucinus (ENSCCAT00000043679)
0 500 1000 1500 2000 2500 3000 3500
0 100 200 300 400

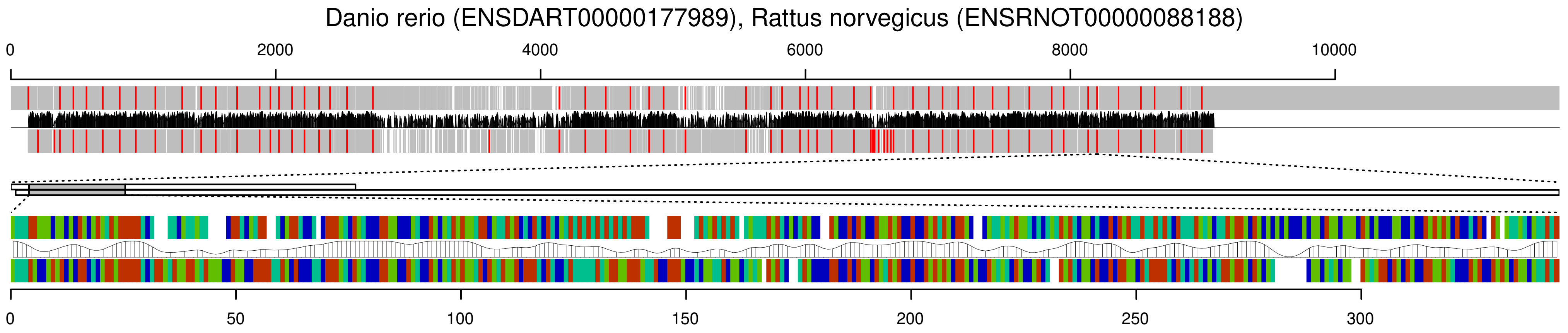
Danio rerio (ENSDART00000177989), Rattus norvegicus (ENSRNOT00000088188)
0 2000 4000 6000 8000 10000
0 50 100 150 200 250 300

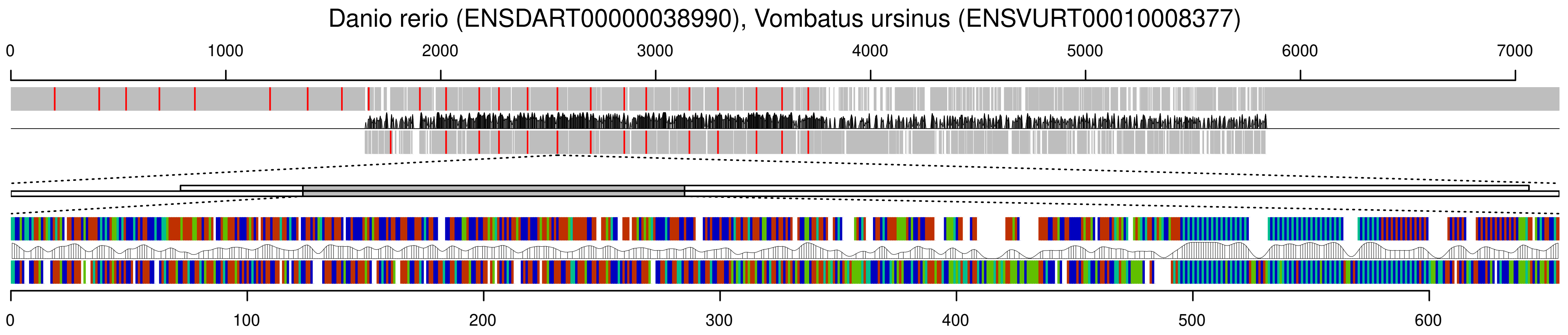
Danio rerio (ENSDART00000038990), Vombatus ursinus (ENSVURT00010008377)
0 1000 2000 3000 4000 5000 6000 7000
0 100 200 300 400 500 600

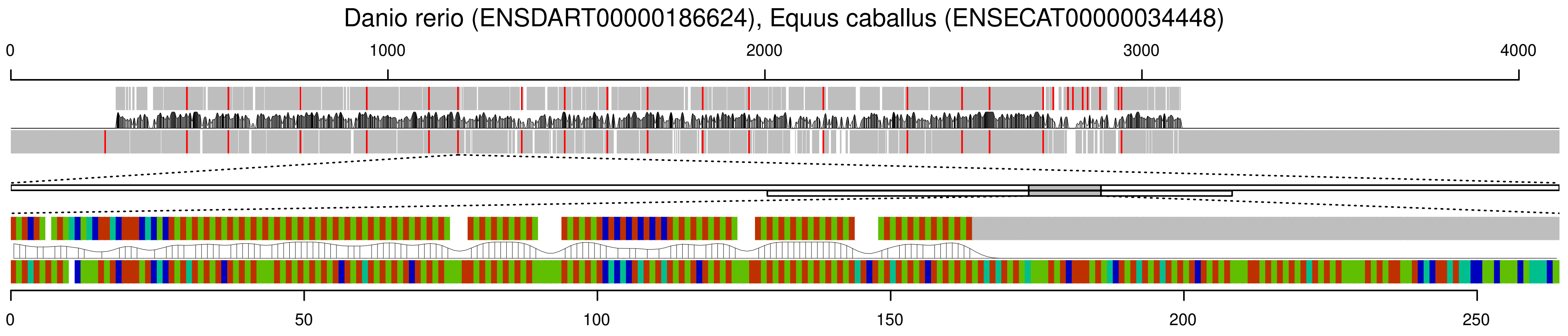
Danio rerio (ENSDART00000186624), Equus caballus (ENSECAT00000034448)
0 1000 2000 3000 4000
0 50 100 150 200 250

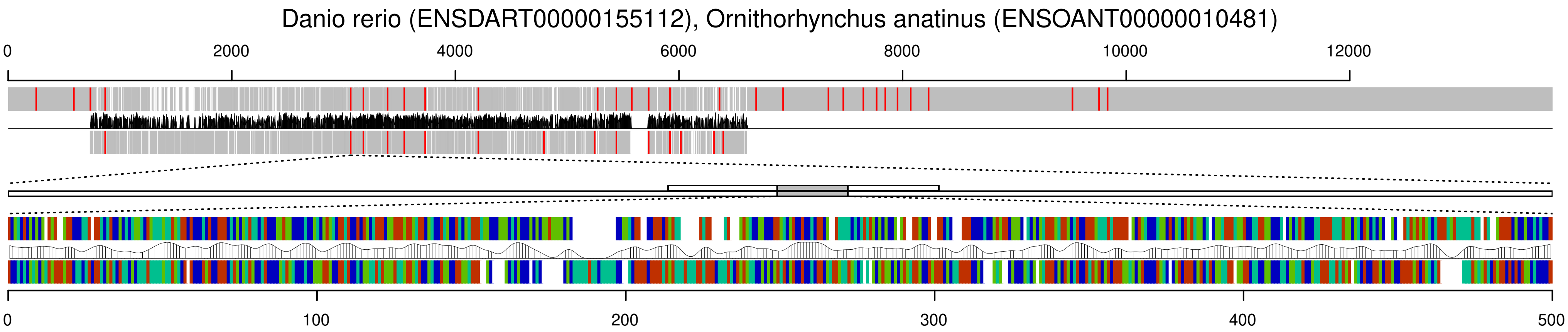
Danio rerio (ENSDART00000155112), Ornithorhynchus anatinus (ENSOANT00000010481)
0 2000 4000 6000 8000 10000 12000
0 100 200 300 400 500

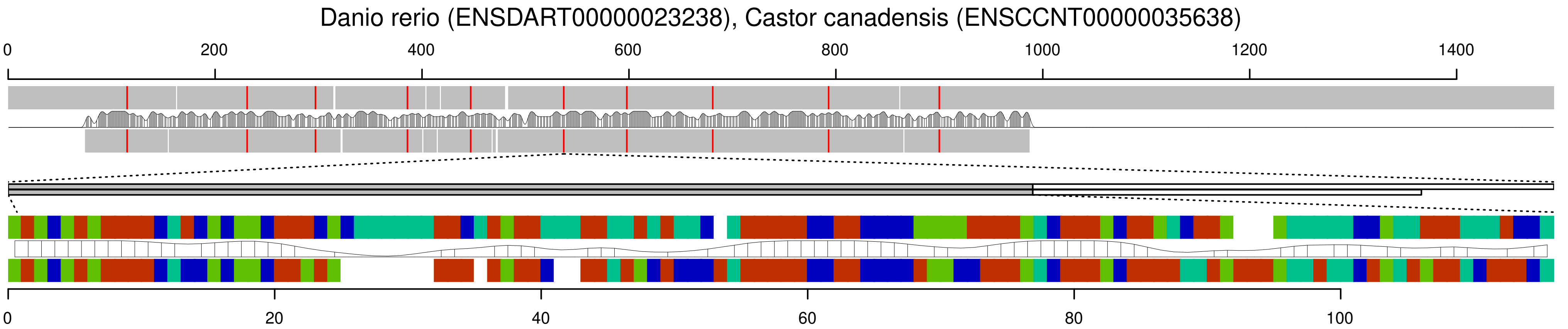
Danio rerio (ENSDART00000023238), Castor canadensis (ENSCCNT00000035638)
0 200 400 600 800 1000 1200 1400
0 20 40 60 80 100

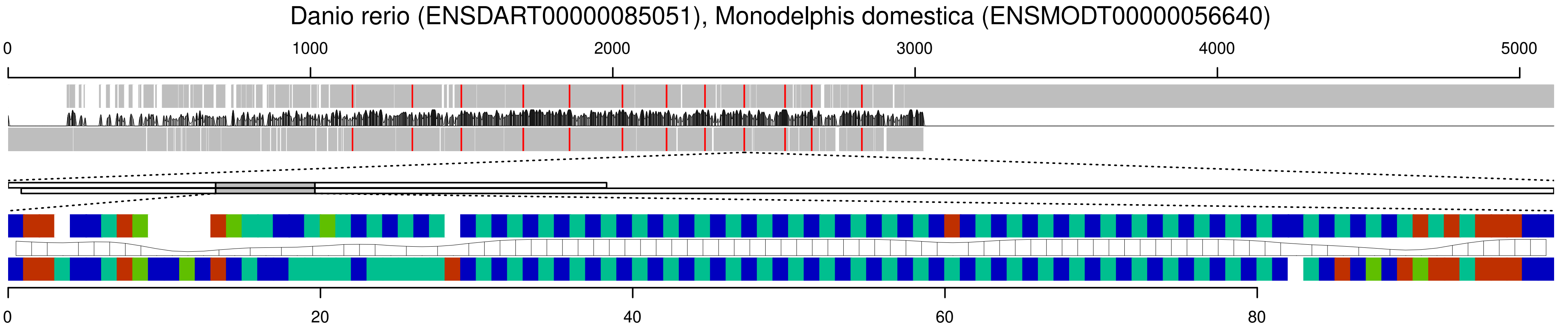
Danio rerio (ENSDART00000085051), Monodelphis domestica (ENSMODT00000056640)
0 1000 2000 3000 4000 5000
0 20 40 60 80

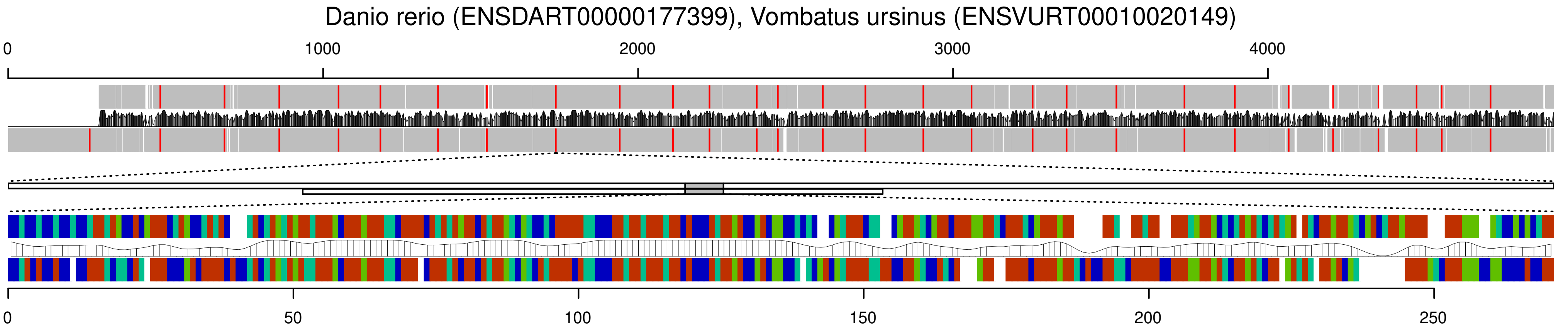
Danio rerio (ENSDART00000177399), Vombatus ursinus (ENSVURT00010020149)
0 1000 2000 3000 4000
0 50 100 150 200 250

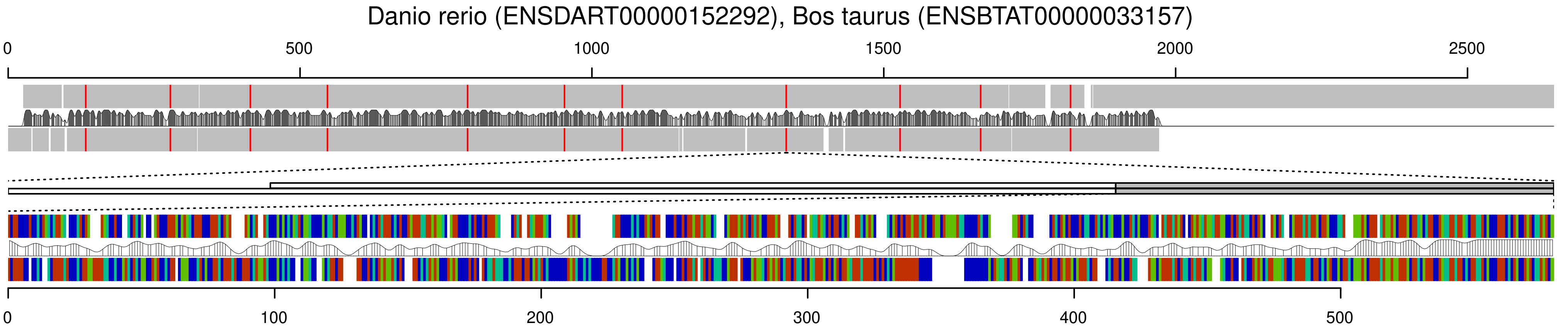
Danio rerio (ENSDART00000152292), Bos taurus (ENSBTAT00000033157)
0 500 1000 1500 2000 2500
0 100 200 300 400 500

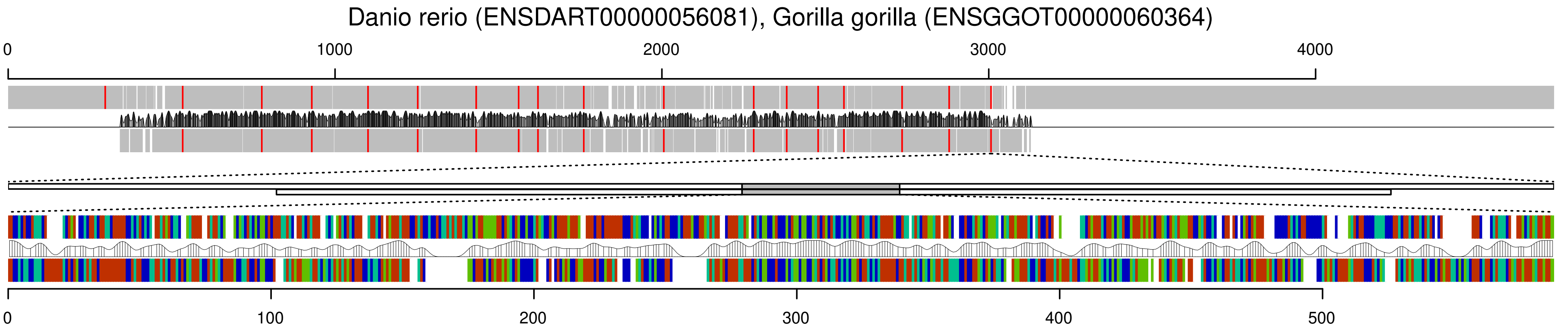
Danio rerio (ENSDART00000056081), Gorilla gorilla (ENSGGOT00000060364)
0 1000 2000 3000 4000
0 100 200 300 400 500

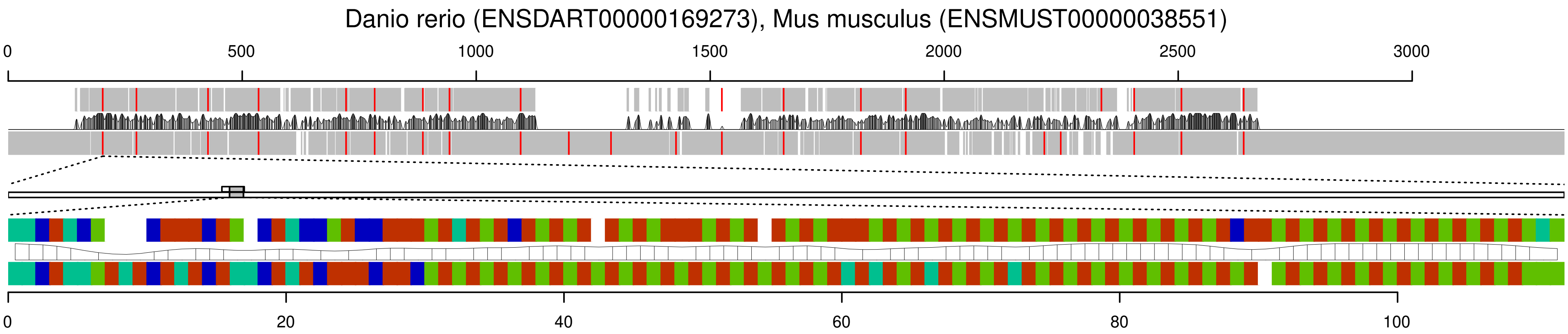
Danio rerio (ENSDART00000169273), Mus musculus (ENSMUST00000038551)
0 500 1000 1500 2000 2500 3000
0 20 40 60 80 100

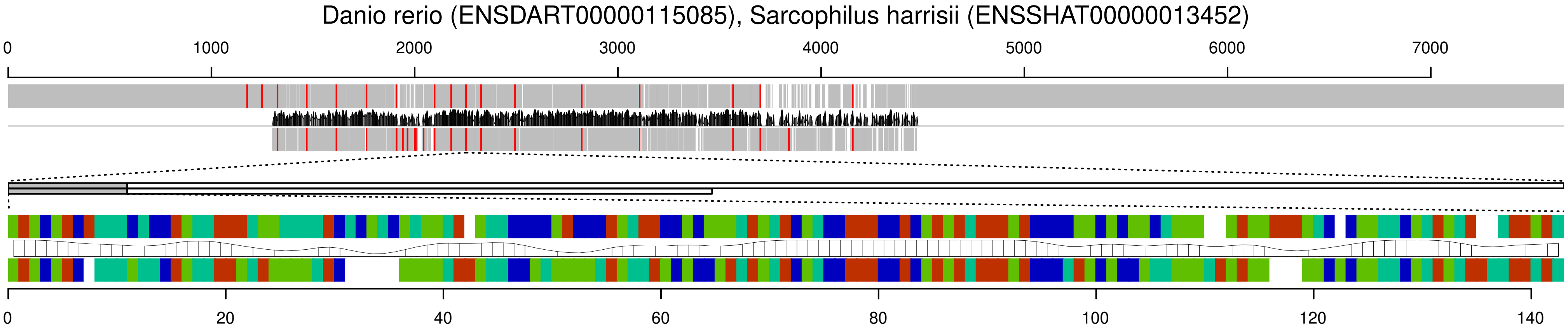
Danio rerio (ENSDART00000115085), Sarcophilus harrisii (ENSSHAT00000013452)
0 1000 2000 3000 4000 5000 6000 7000
0 20 40 60 80 100 120 140

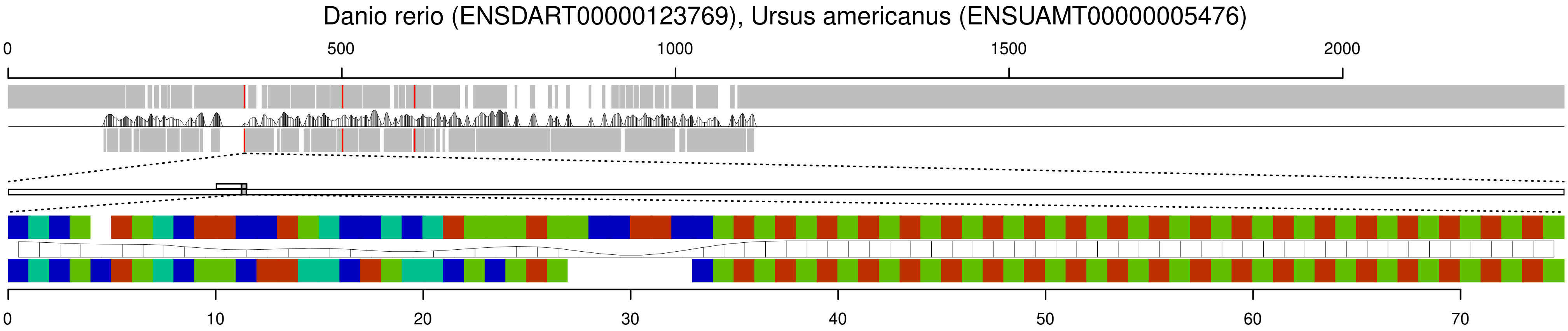
Danio rerio (ENSDART00000123769), Ursus americanus (ENSUAMT00000005476)
0 500 1000 1500 2000
0 10 20 30 40 50 60 70

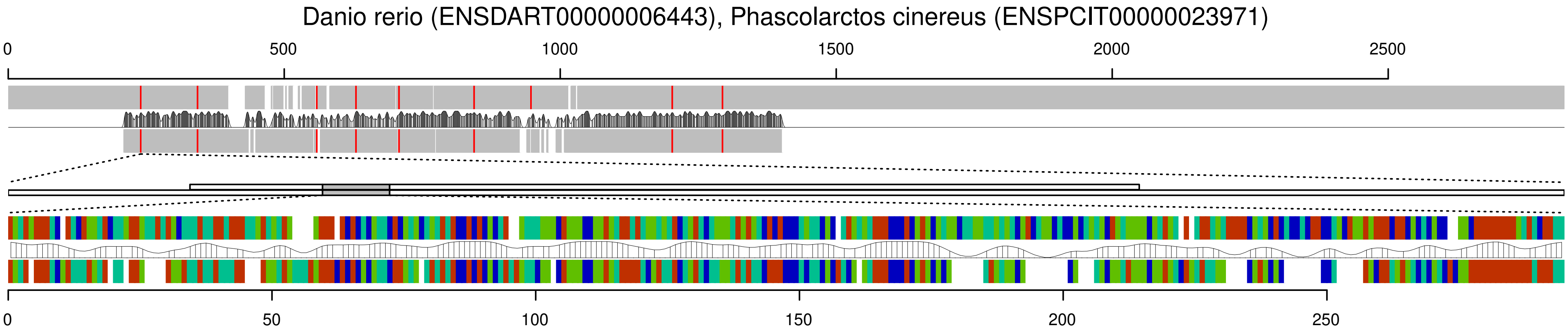
Danio rerio (ENSDART00000006443), Phascolarctos cinereus (ENSPCIT00000023971)
0 500 1000 1500 2000 2500
0 50 100 150 200 250

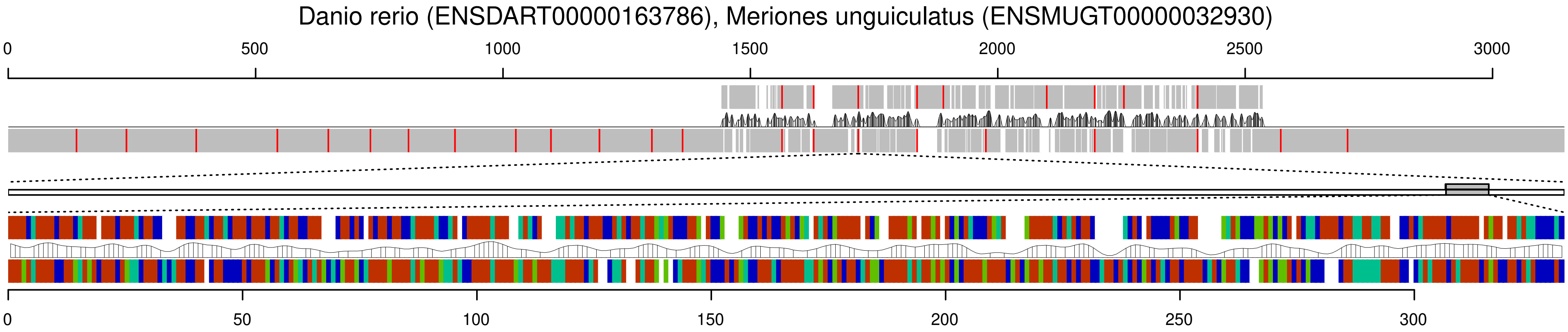
Danio rerio (ENSDART00000163786), Meriones unguiculatus (ENSMUGT00000032930)
0 500 1000 1500 2000 2500 3000
0 50 100 150 200 250 300

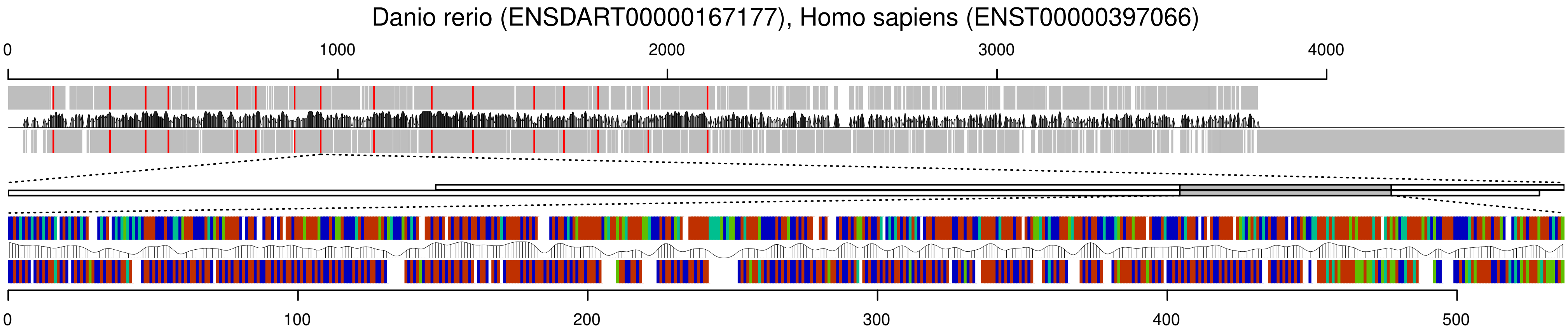
Danio rerio (ENSDART00000167177), Homo sapiens (ENST00000397066)
0 1000 2000 3000 4000
0 100 200 300 400 500

Danio rerio (ENSDART00000063154), Castor canadensis (ENSCCNT00000025027)

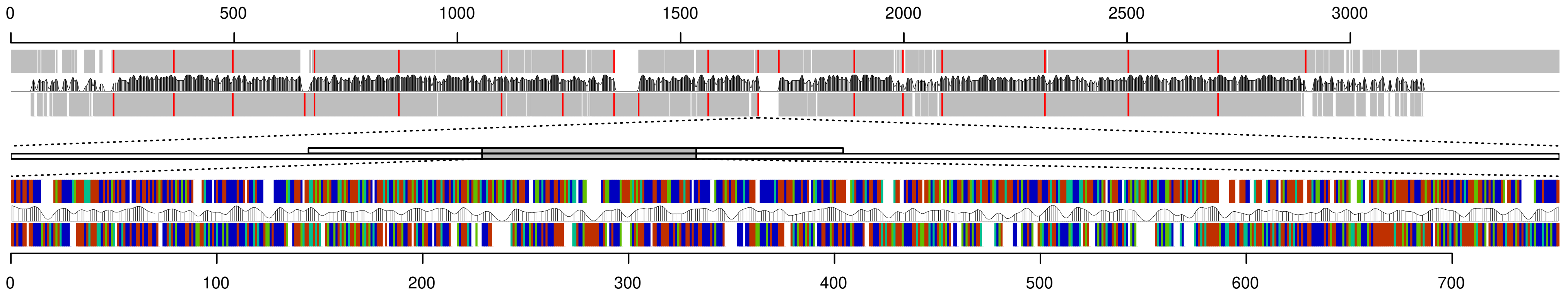

Danio rerio (ENSDART00000104496), Notamacropus eugenii (ENSMEUT00000012134)

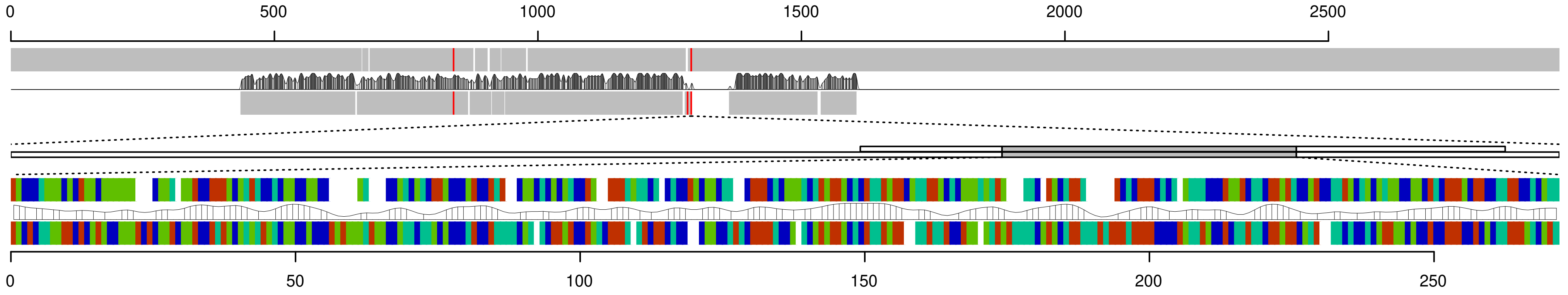

Danio rerio (ENSDART00000029387), Mandrillus leucophaeus (ENSMLET00000061438)

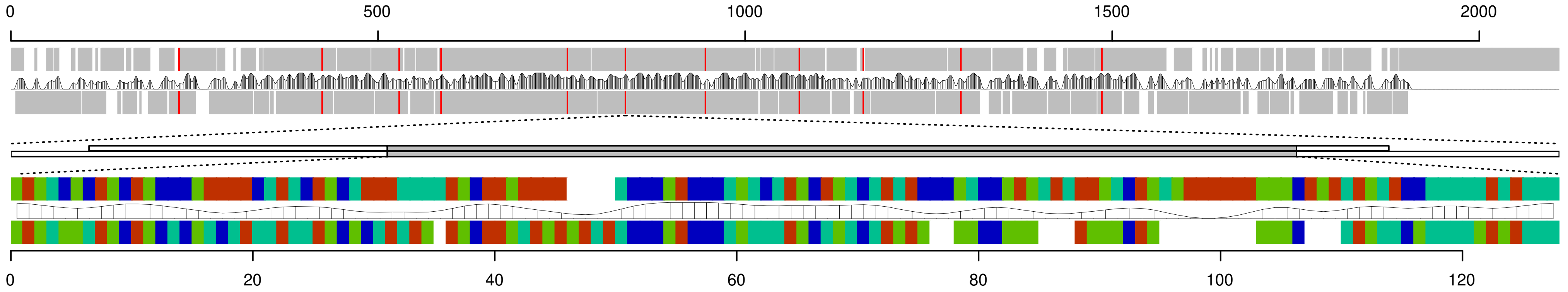

Danio rerio (ENSDART00000025019), Loxodonta africana (ENSLAFT00000021679)

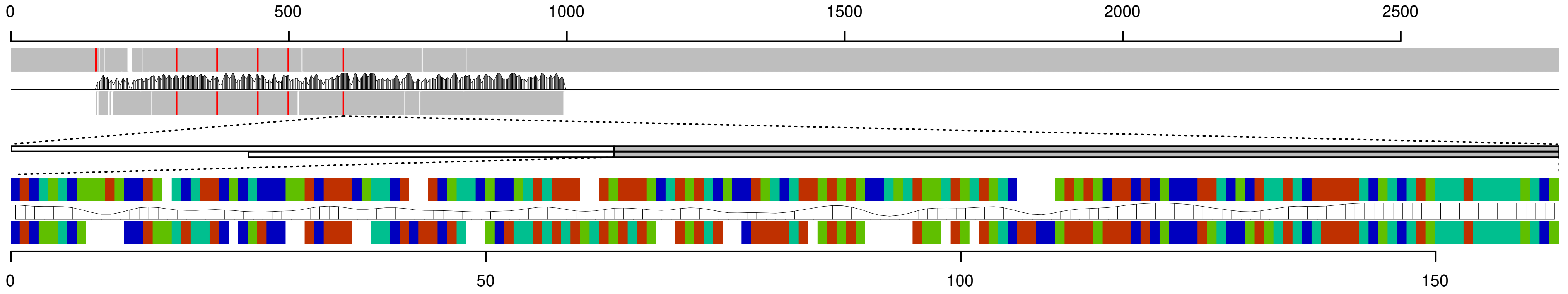

Danio rerio (ENSDART00000093343), Microtus ochrogaster (ENSMOCT00000009135)

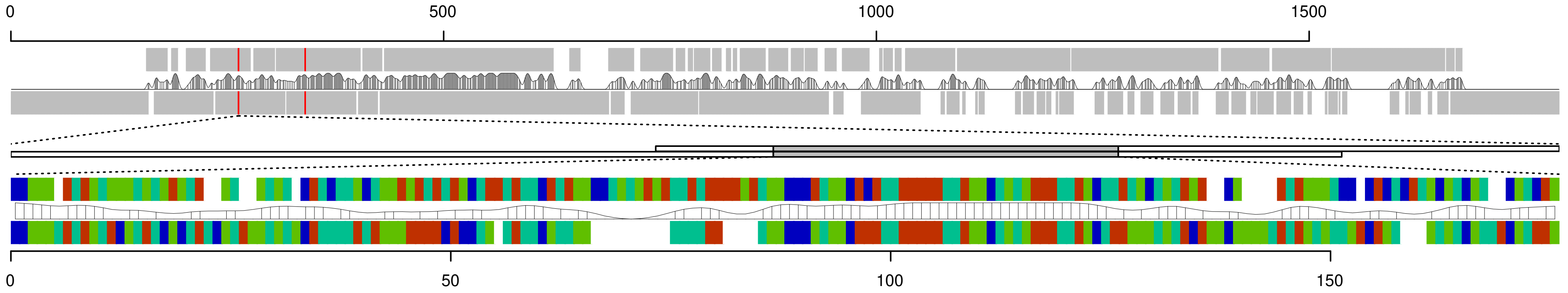

Danio rerio (ENSDART00000021299), Choloepus hoffmanni (ENSCHOT00000001245)

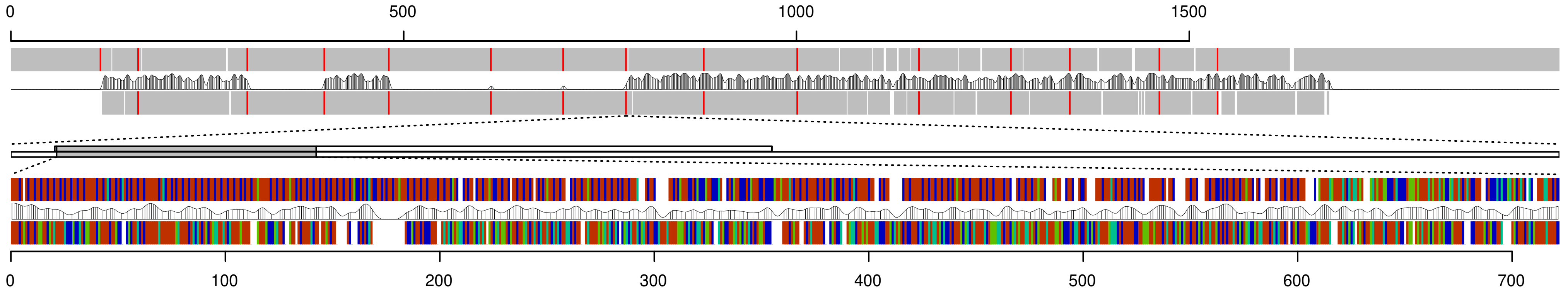

Danio rerio (ENSDART00000129125), Microtus ochrogaster (ENSMOCT00000006875)

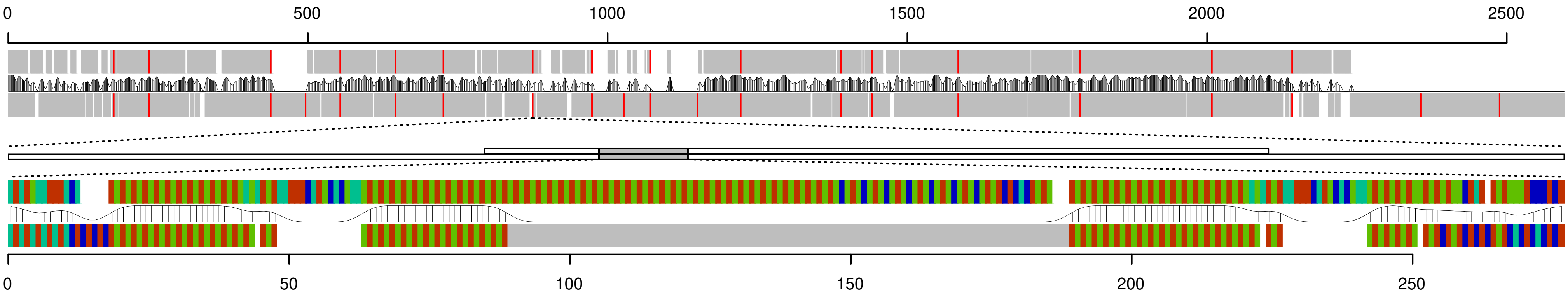

Danio rerio (ENSDART00000091409), Sarcophilus harrisii (ENSSHAT00000001452)

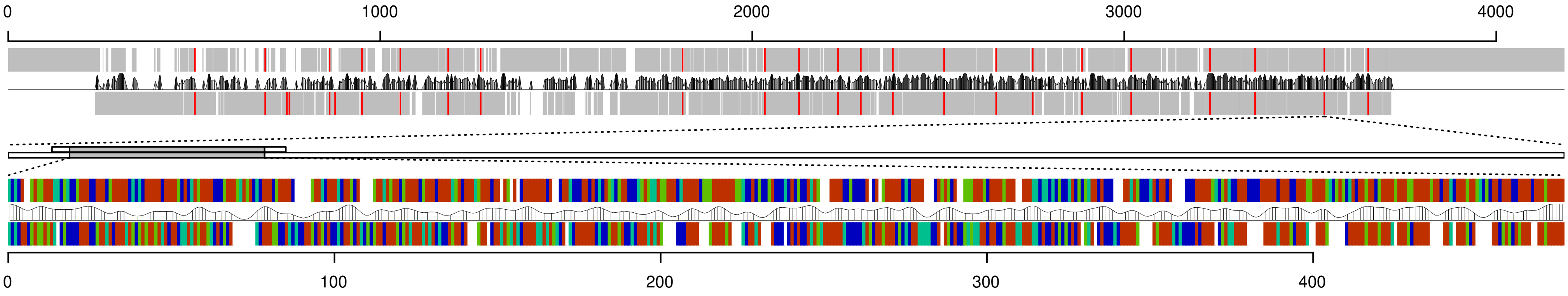

Danio rerio (ENSDART00000140243), Dipodomys ordii (ENSDORT00000015767)

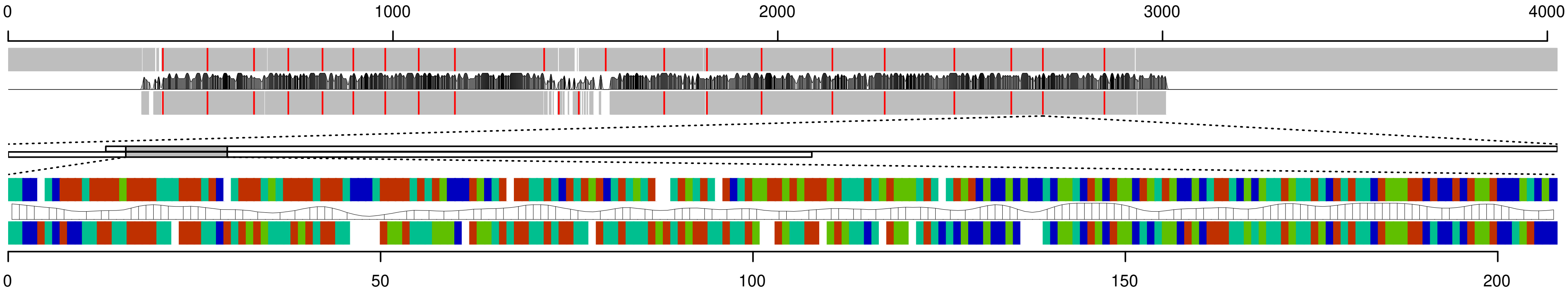

Danio rerio (ENSDART00000051139), Aotus nancymaae (ENSANAT00000050931)

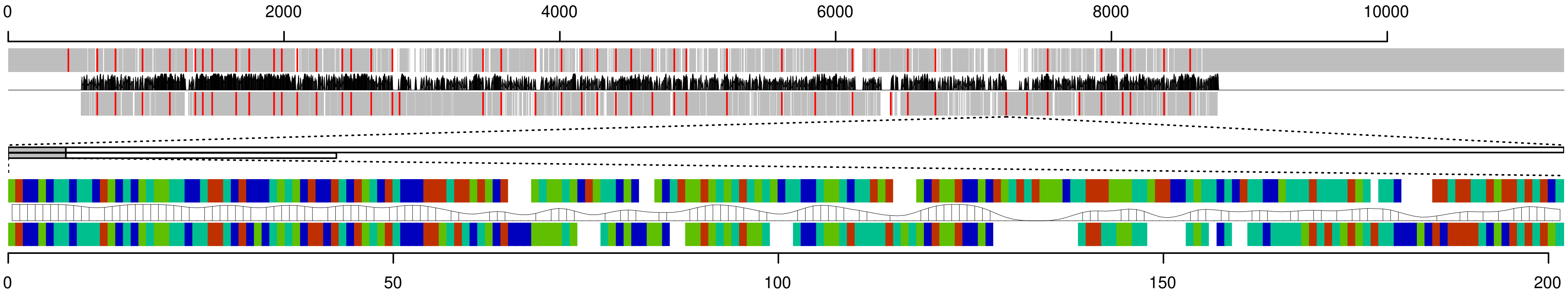

Danio rerio (ENSDART00000162962), Monodelphis domestica (ENSMODT00000078792)

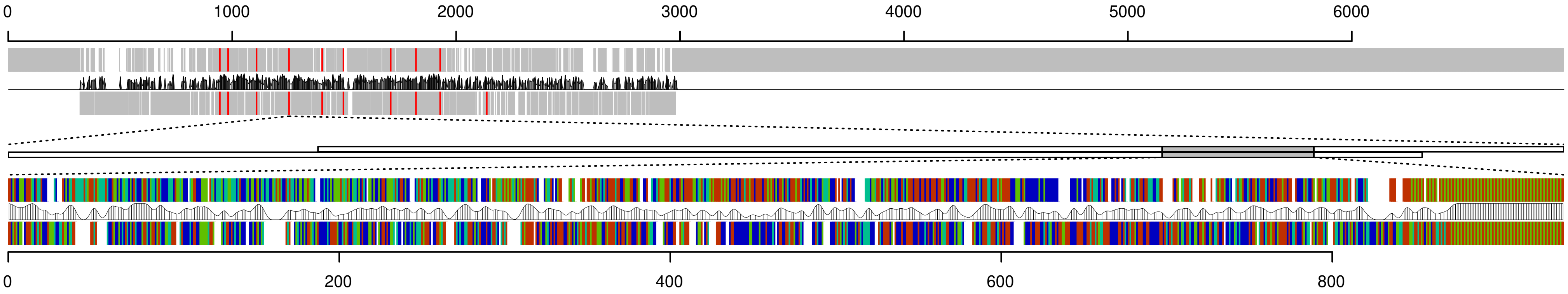

Danio rerio (ENSDART00000169846), Pteropus vampyrus (ENSPVAT00000003007)

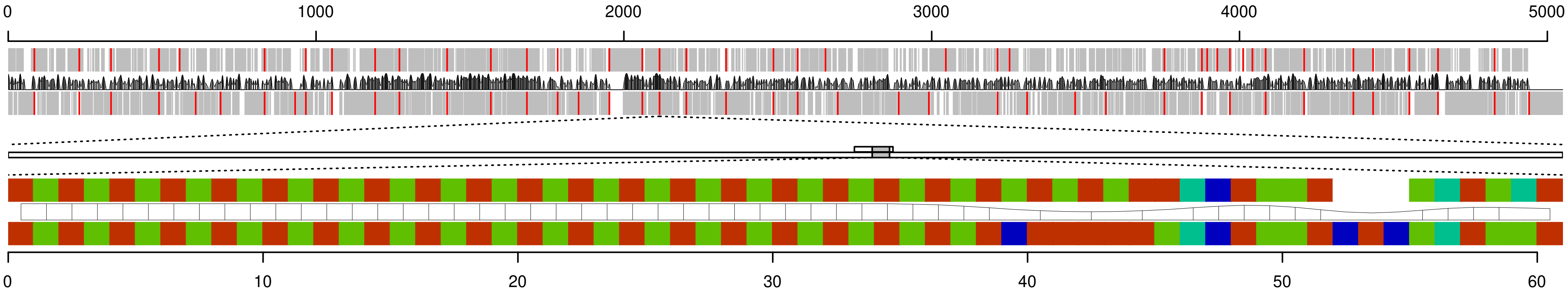

Danio rerio (ENSDART00000157621), Erinaceus europaeus (ENSEEUT00000005961)

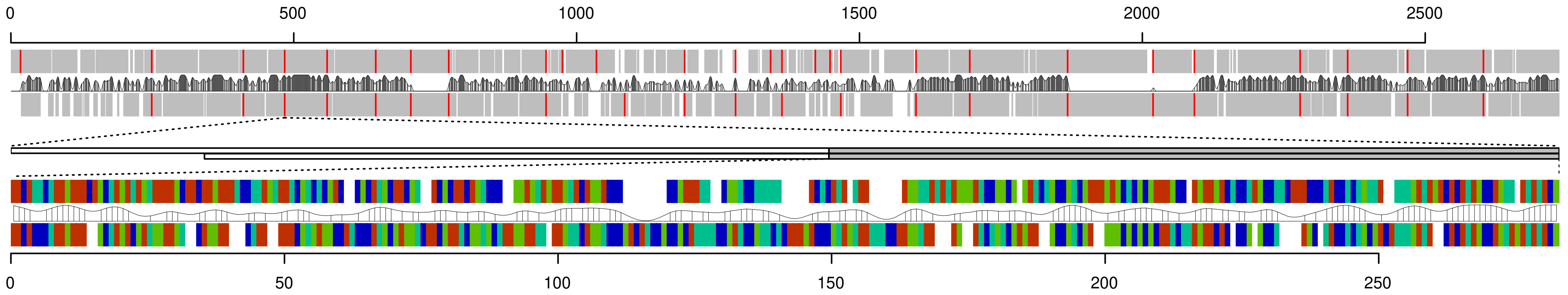

Danio rerio (ENSDART00000147520), Mus pahari (MGP_PahariEiJ_T0031853)

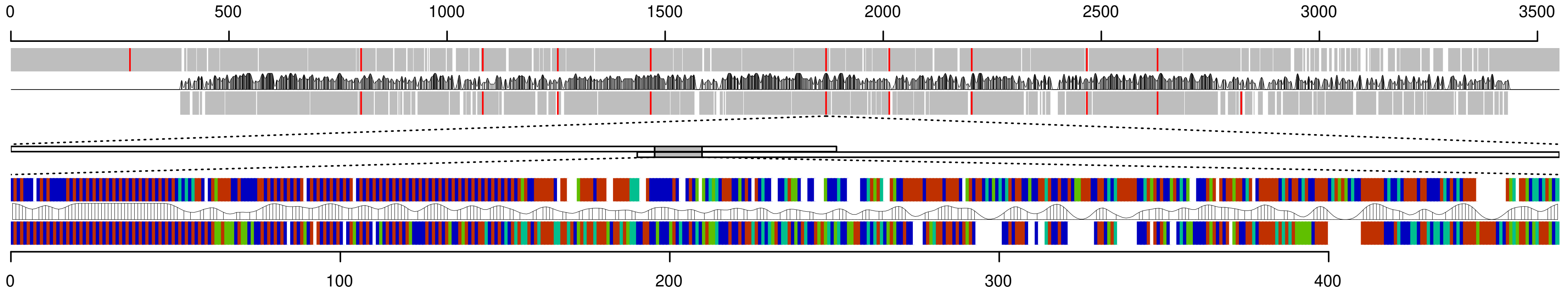

Danio rerio (ENSDART00000188914), Dipodomys ordii (ENSDORT00000000789)

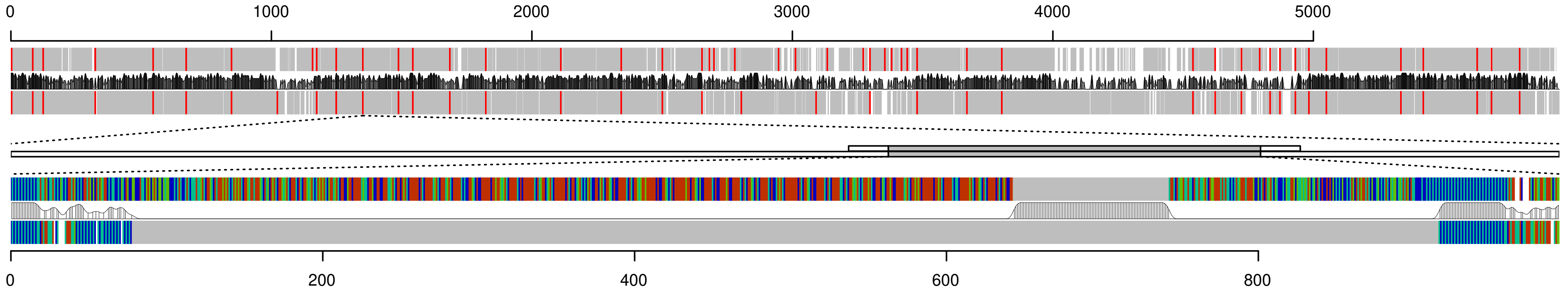

Danio rerio (ENSDART00000171986), Sorex araneus (ENSSART00000011915)

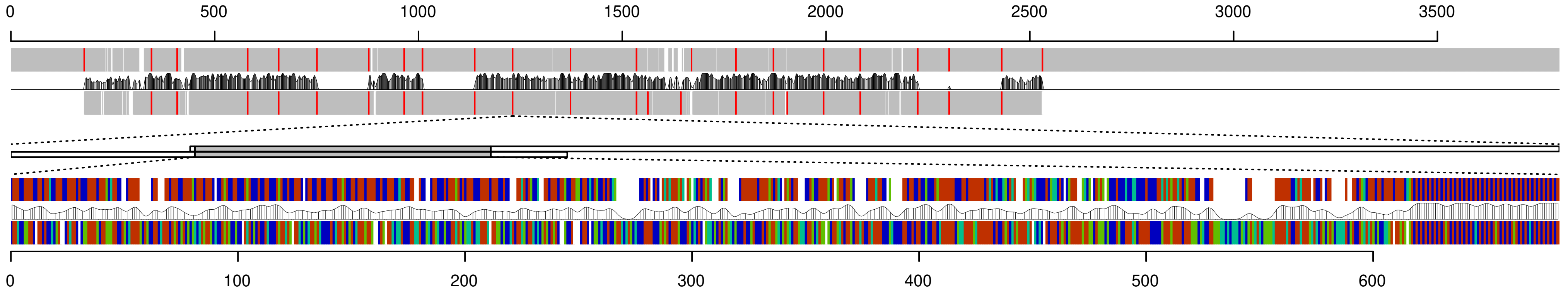

Danio rerio (ENSDART00000162359), Phascolarctos cinereus (ENSPCIT00000002620)

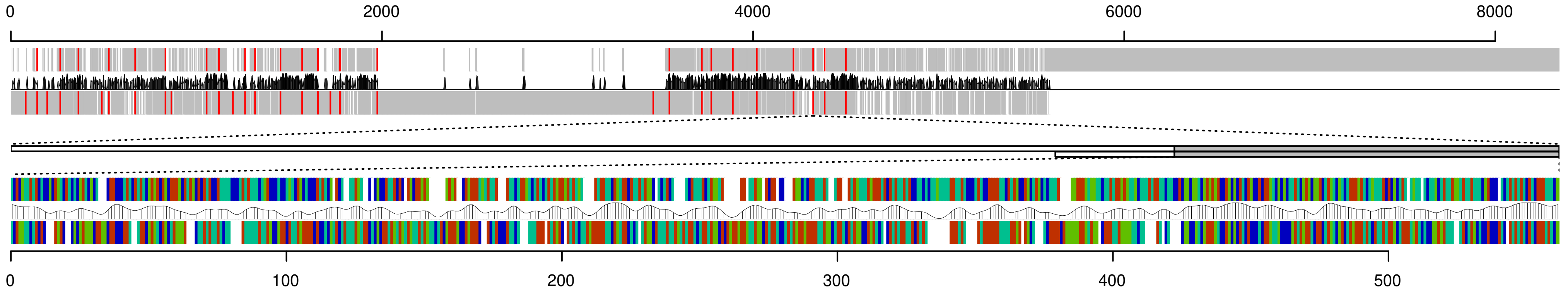

Danio rerio (ENSDART00000103350), Mesocricetus auratus (ENSMAUT00000022710)

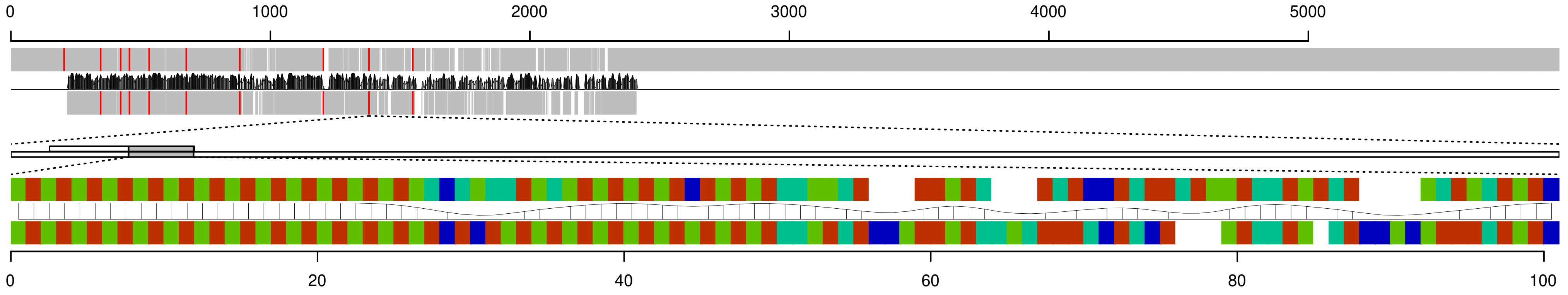

Danio rerio (ENSDART00000115085), Ailuropoda melanoleuca (ENSAMET00000010518)

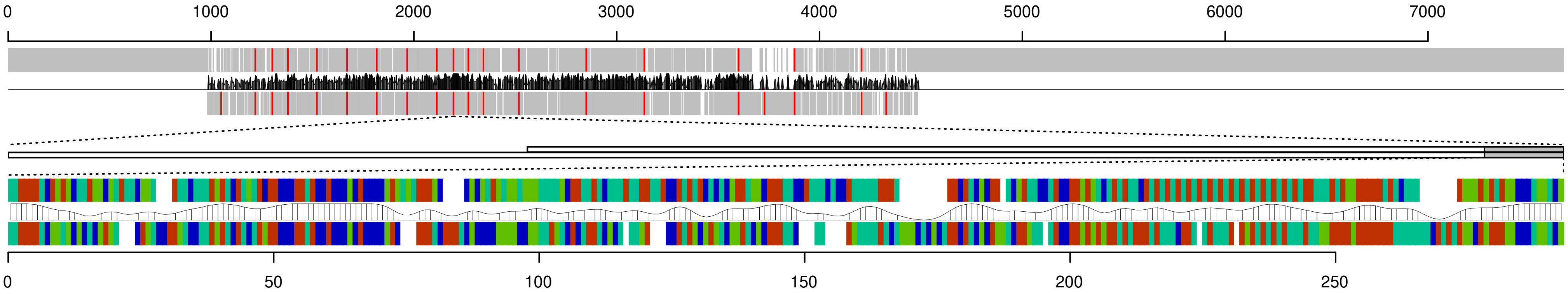

Danio rerio (ENSDART00000013066), Felis catus (ENSFCAT00000077044)

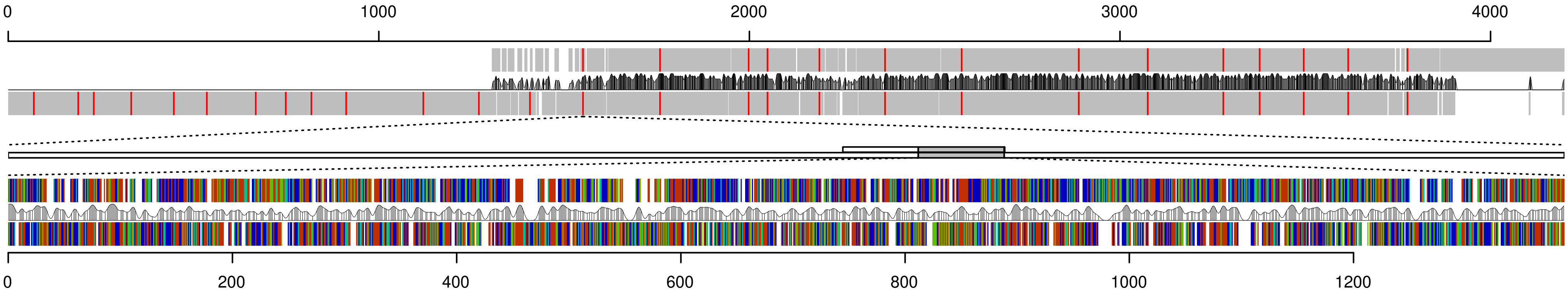

Danio rerio (ENSDART00000102304), Spermophilus dauricus (ENSSDAT00000027860)

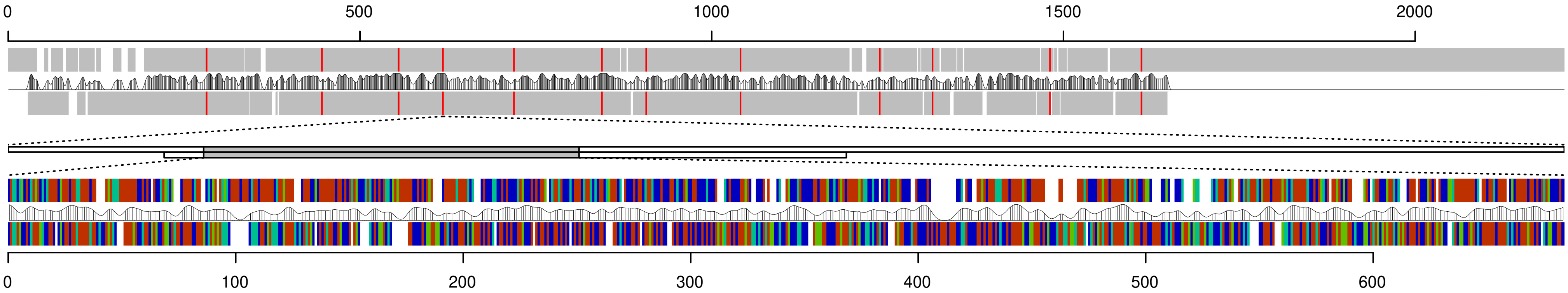

Danio rerio (ENSDART00000104256), Nannospalax galili (ENSNGAT00000019170)

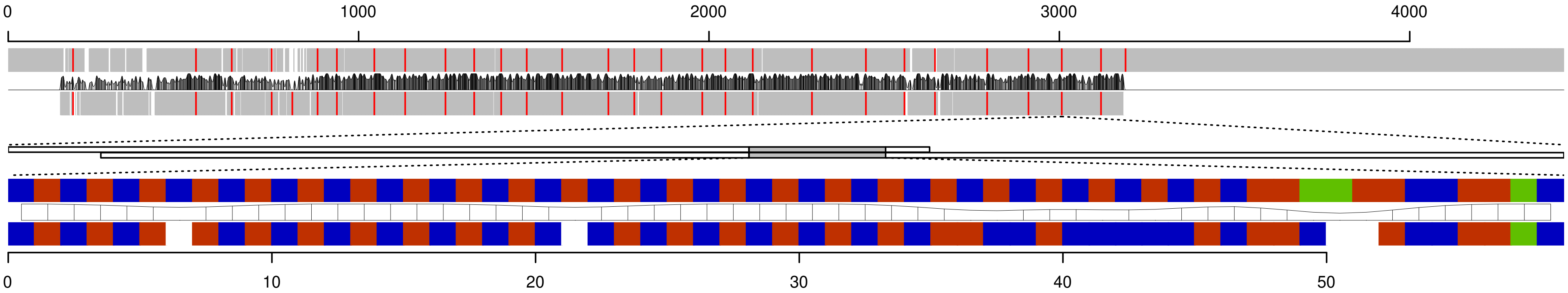

Danio rerio (ENSDART00000150159), Sorex araneus (ENSSART00000012555)

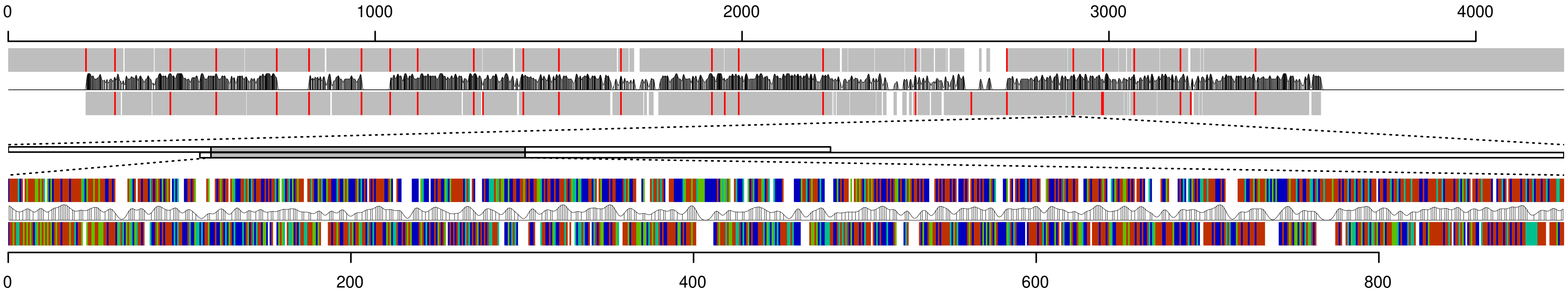

Danio rerio (ENSDART00000123574), Sus scrofa (ENSSSCT00000017094)

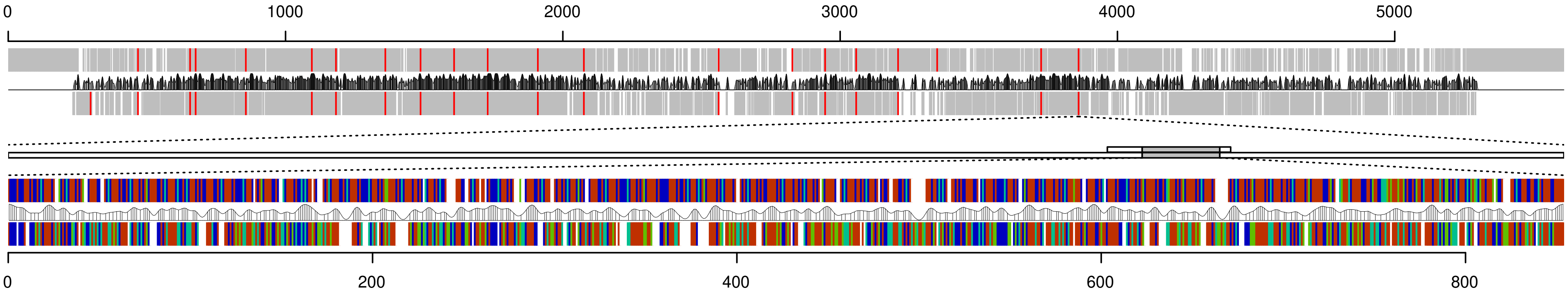

Danio rerio (ENSDART00000142584), Myotis lucifugus (ENSMLUT00000008592)

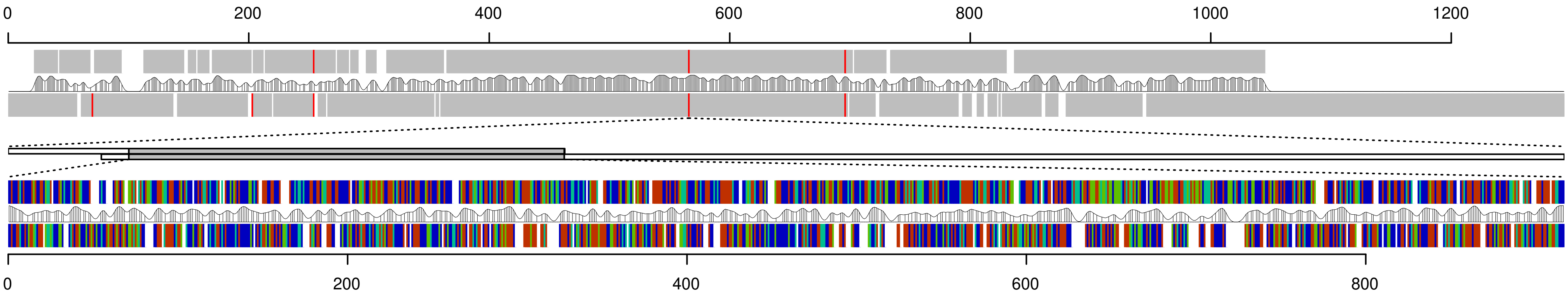

Danio rerio (ENSDART00000159528), Monodelphis domestica (ENSMODT00000084475)

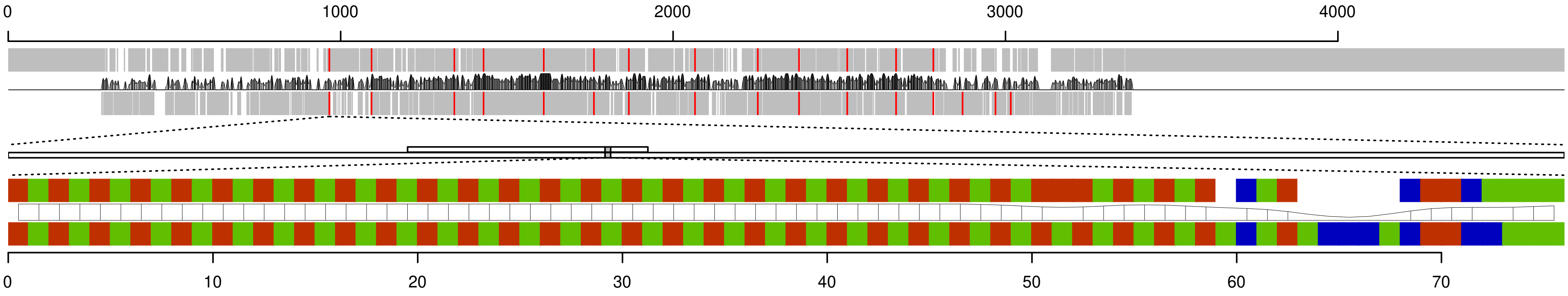

Danio rerio (ENSDART00000132602), Monodelphis domestica (ENSMODT00000003373)

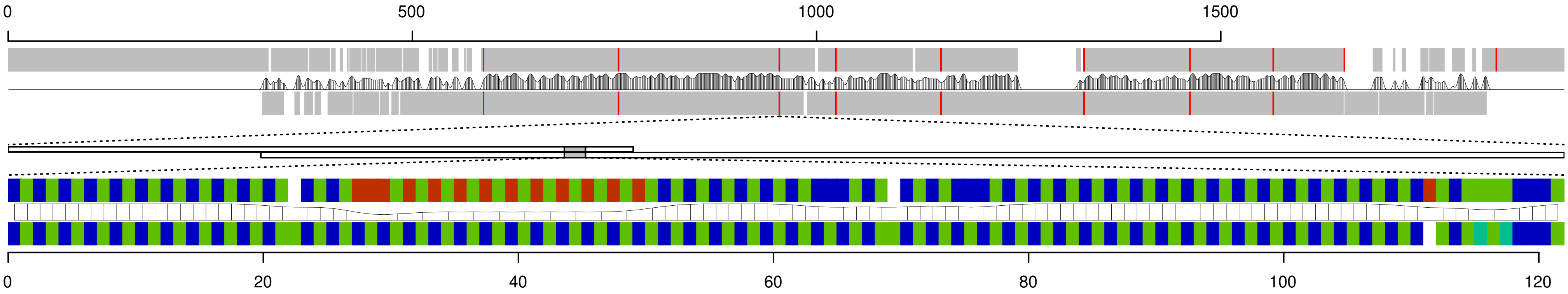

Danio rerio (ENSDART00000129750), Spermophilus dauricus (ENSSDAT00000021231)

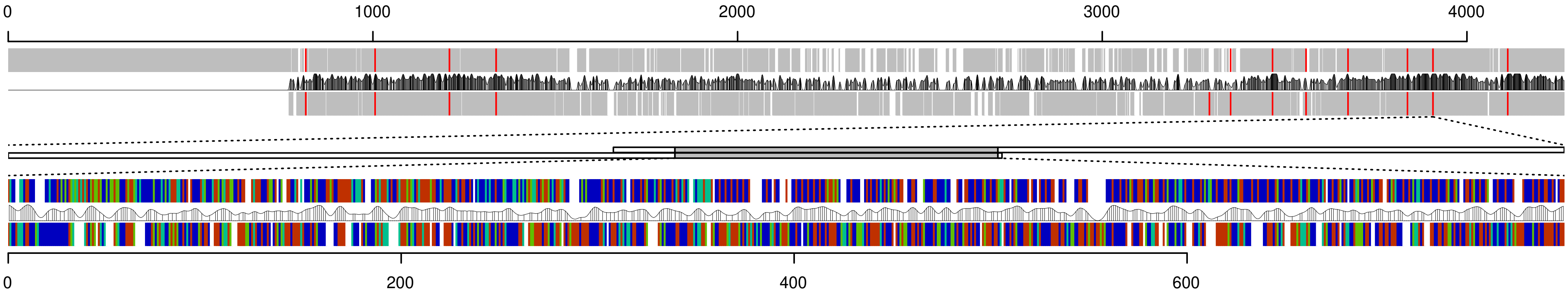

Danio rerio (ENSDART00000073564), Canis familiaris (ENSCAFT00000038024)

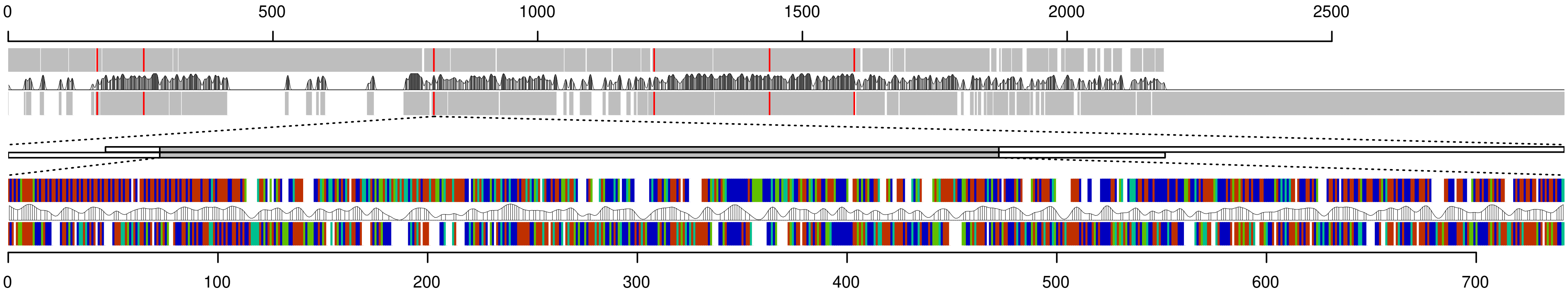

Danio rerio (ENSDART00000008532), Phascolarctos cinereus (ENSPCIT00000042596)

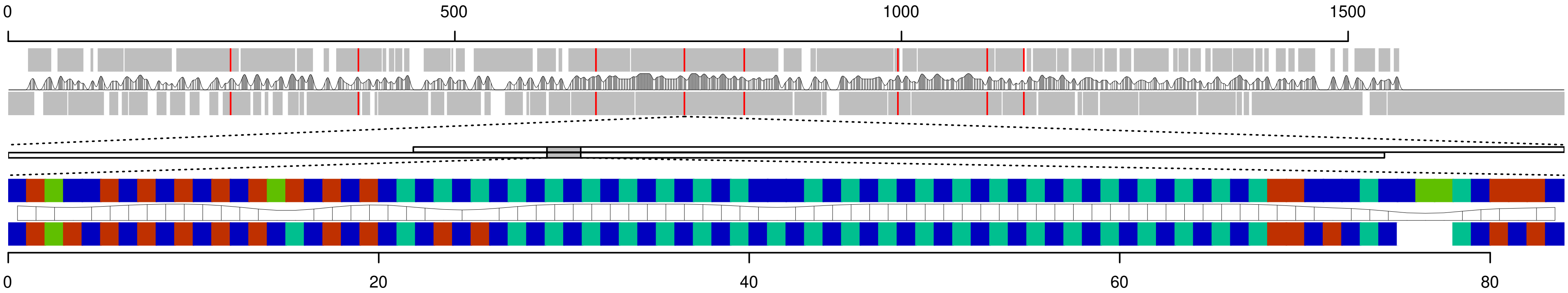

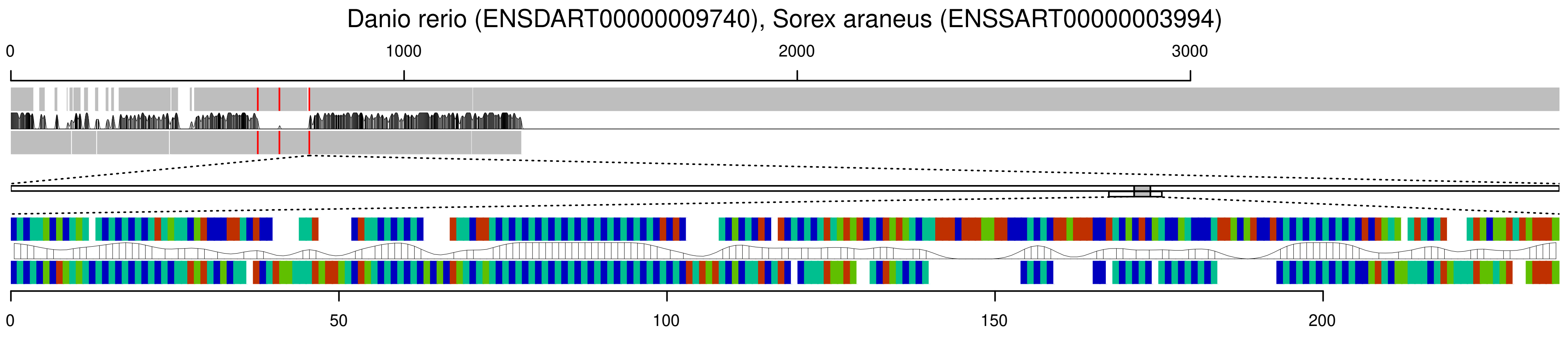
Danio rerio (ENSDART00000009740), Sorex araneus (ENSSART00000003994)
0
1000
2000
3000
0
50
100
150
200

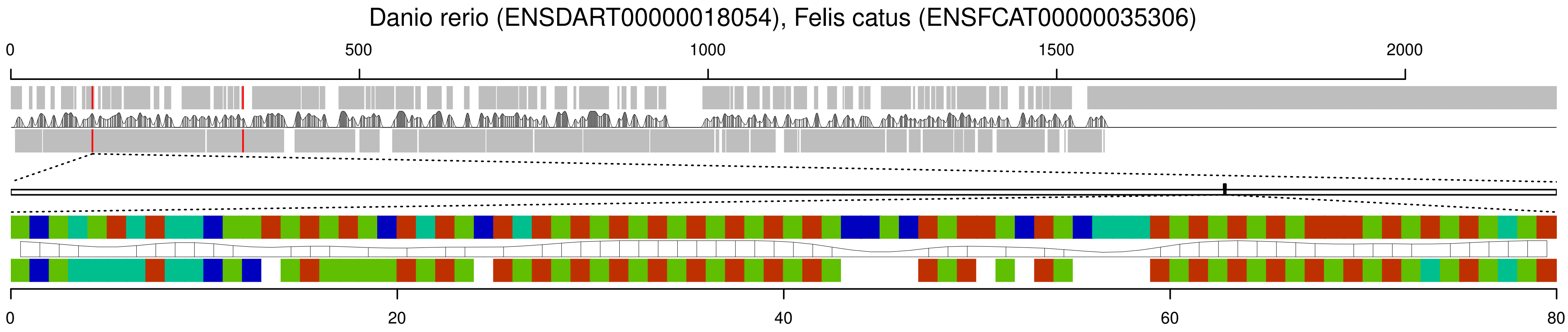
Danio rerio (ENSDART00000018054), Felis catus (ENSFCAT00000035306)
0
500
1000
1500
2000
0
20
40
60
80

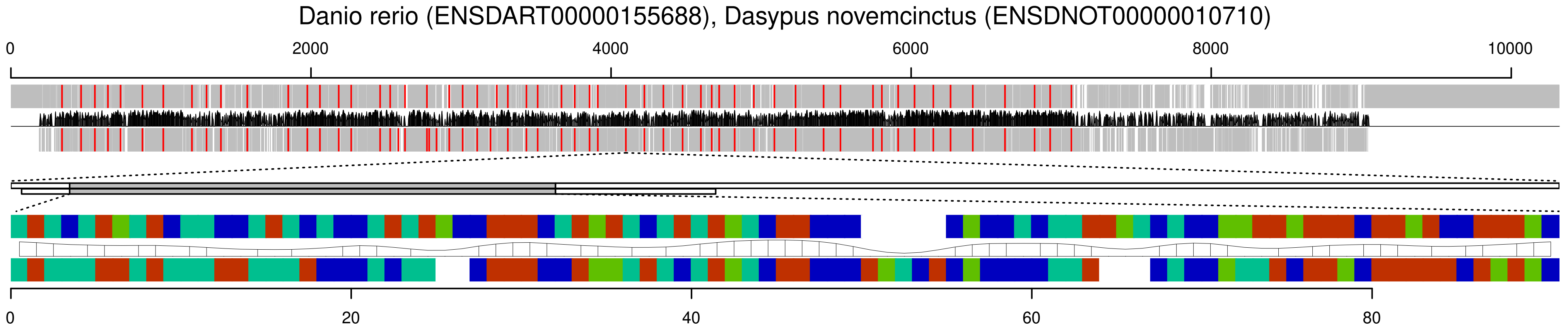
Danio rerio (ENSDART00000155688), Dasypus novemcinctus (ENSDNOT00000010710)
0
2000
4000
6000
8000
10000
0
20
40
60
80

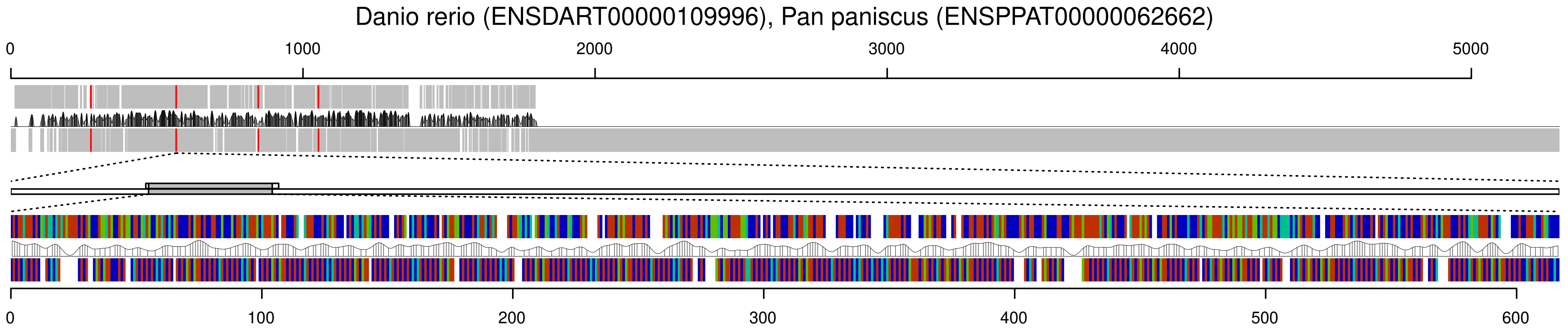
Danio rerio (ENSDART00000109996), Pan paniscus (ENSPPAT00000062662)
0
1000
2000
3000
4000
5000
0
100
200
300
400
500
600

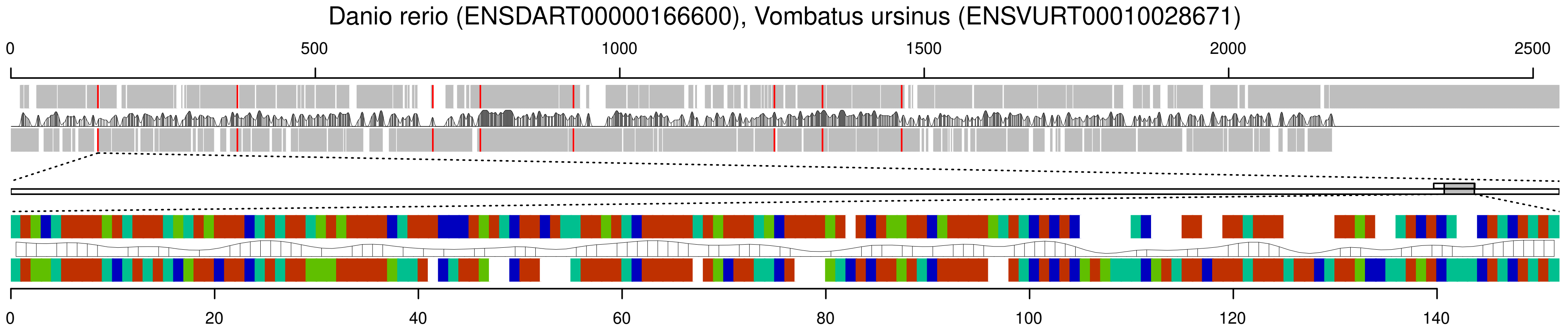
Danio rerio (ENSDART00000166600), Vombatus ursinus (ENSVURT00010028671)
0
500
1000
1500
2000
2500
0
20
40
60
80
100
120
140

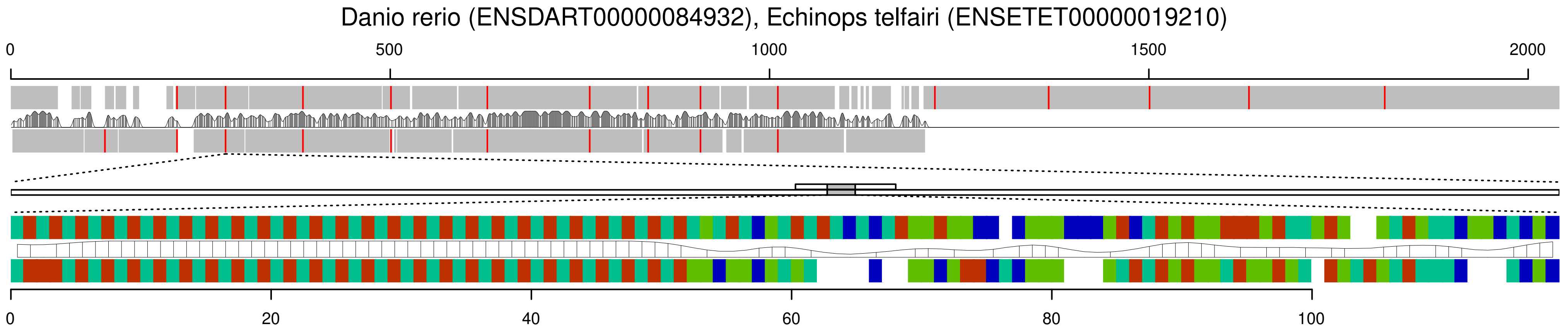
Danio rerio (ENSDART00000084932), Echinops telfairi (ENSETET00000019210)
0
500
1000
1500
2000
0
20
40
60
80
100
