# Supplementary material for: Intron size minimisation in teleosts
Source: BMC Genomics. 2022 Sep 1;23:628. doi: 10.1186/s12864-022-08760-w (PMC9438311; doi:10.1186/s12864-022-08760-w)

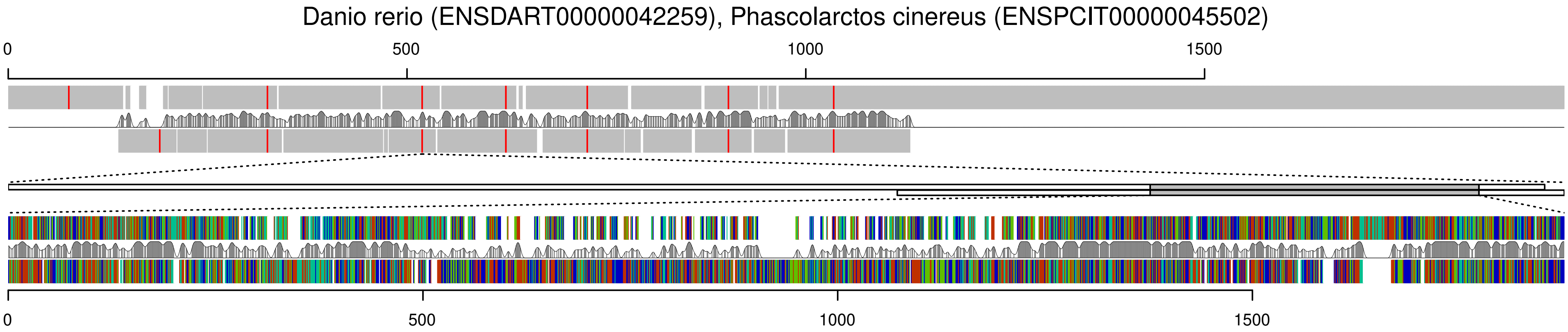
Danio rerio (ENSDART00000042259), Phascolarctos cinereus (ENSPCIT00000045502)

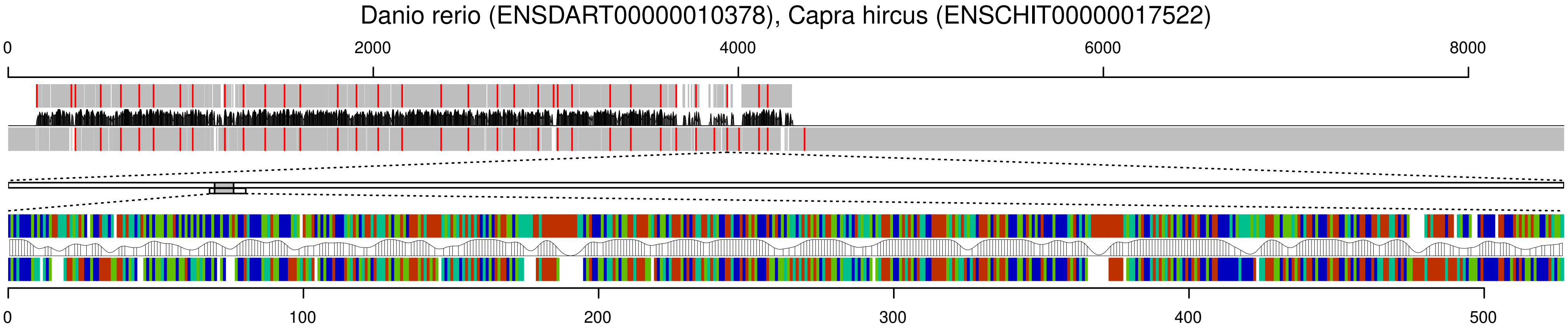
Danio rerio (ENSDART00000010378), Capra hircus (ENSCHIT00000017522)

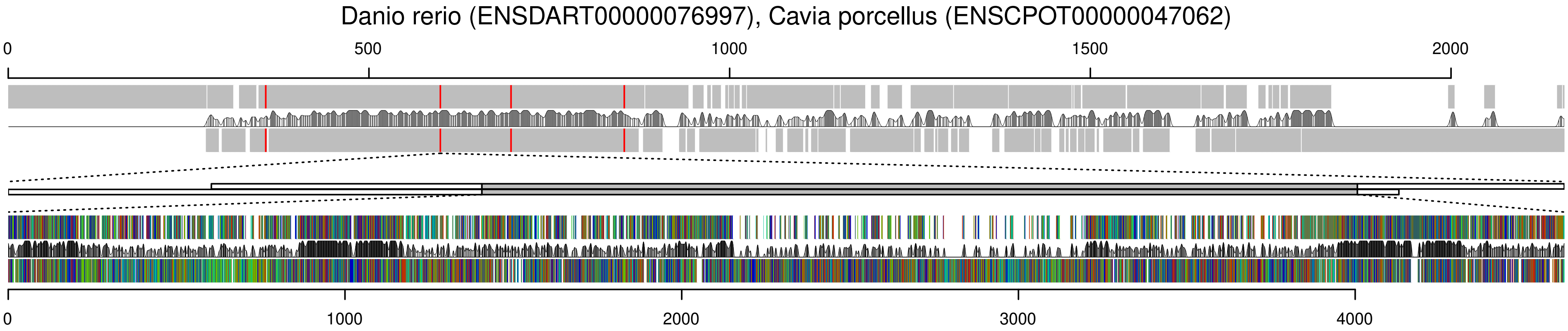
Danio rerio (ENSDART00000076997), Cavia porcellus (ENSCPOT00000047062)

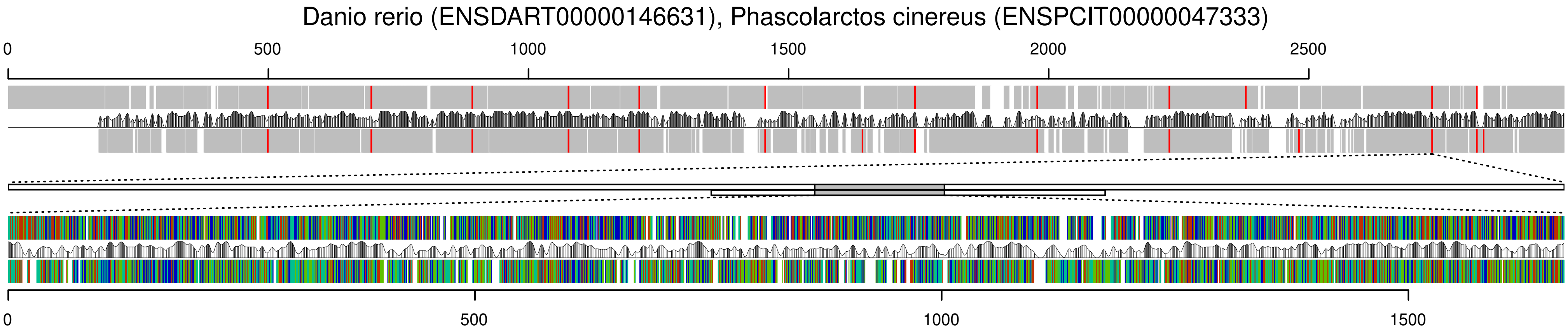
Danio rerio (ENSDART00000146631), Phascolarctos cinereus (ENSPCIT00000047333)

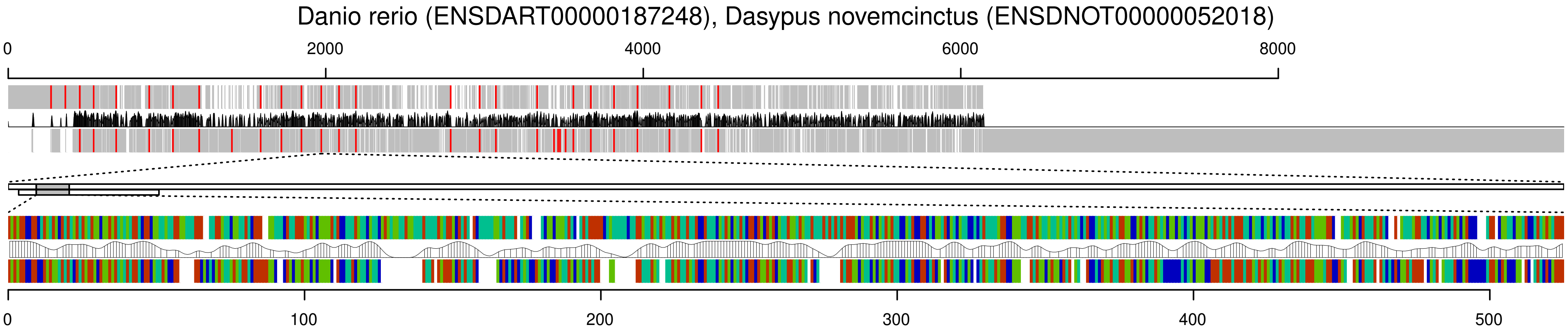
Danio rerio (ENSDART00000187248), Dasypus novemcinctus (ENSDNOT00000052018)

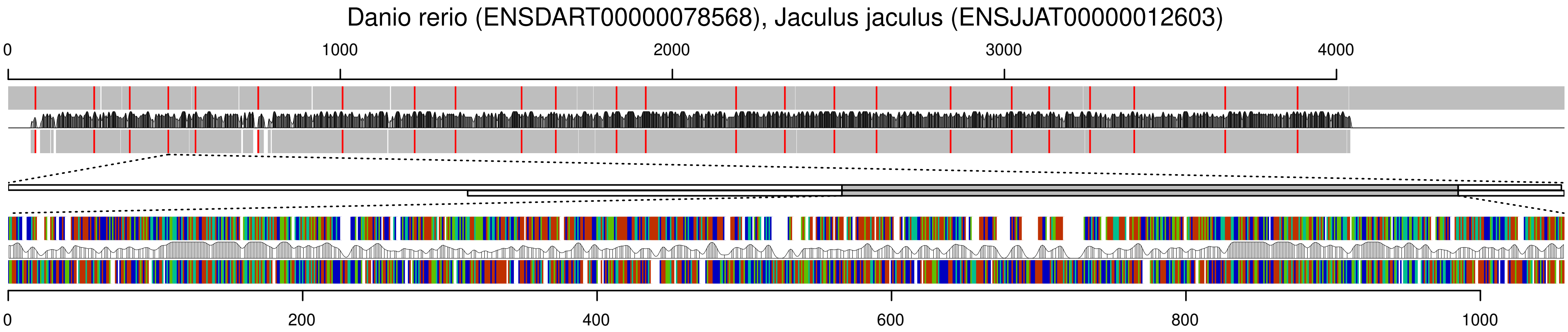
Danio rerio (ENSDART00000078568), Jaculus jaculus (ENSJJAT00000012603)

Danio rerio (ENSDART00000128595), Notamacropus eugenii (ENSMEUT00000004718)

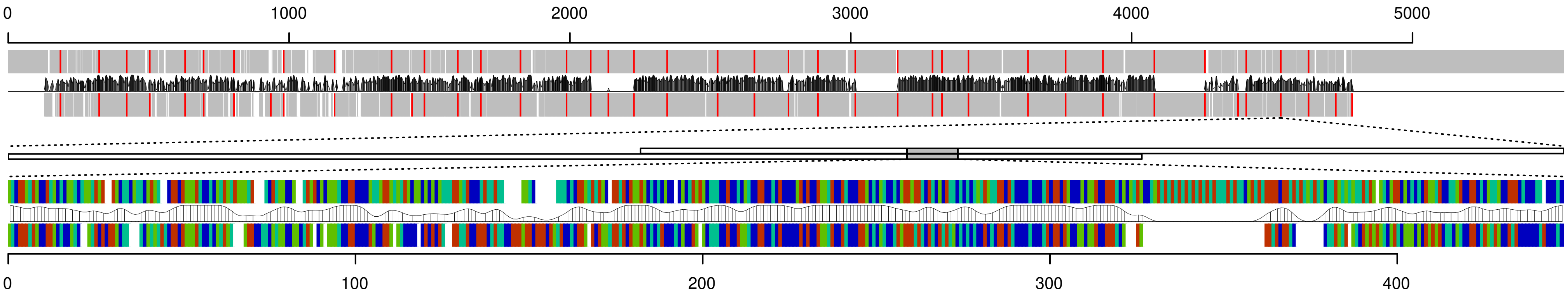

Danio rerio (ENSDART00000159101), Meriones unguiculatus (ENSMUGT00000002296)

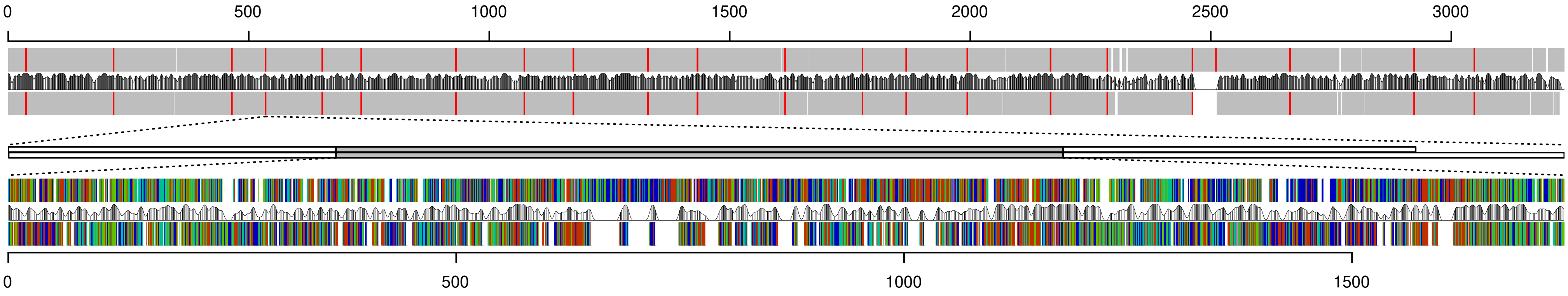

Danio rerio (ENSDART00000111842), Vombatus ursinus (ENSVURT00010029864)

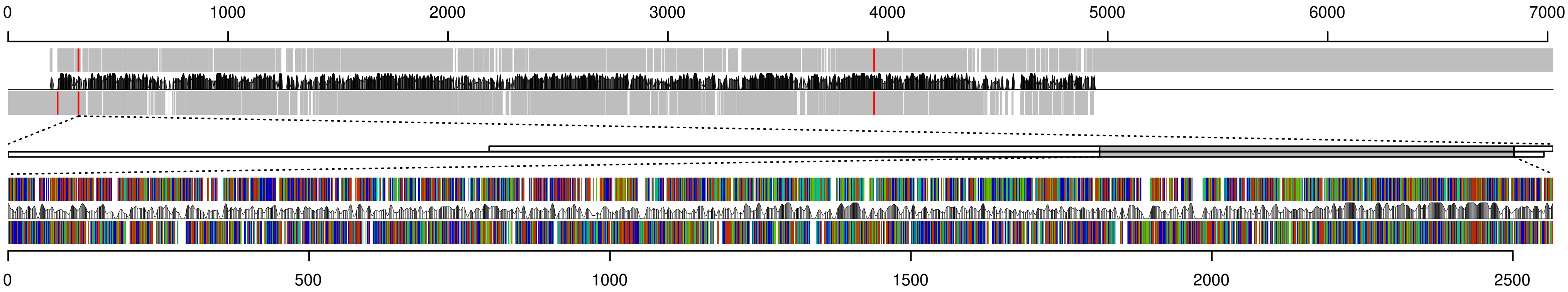

Danio rerio (ENSDART00000077435), Urocitellus parryii (ENSUPAT00010010612)

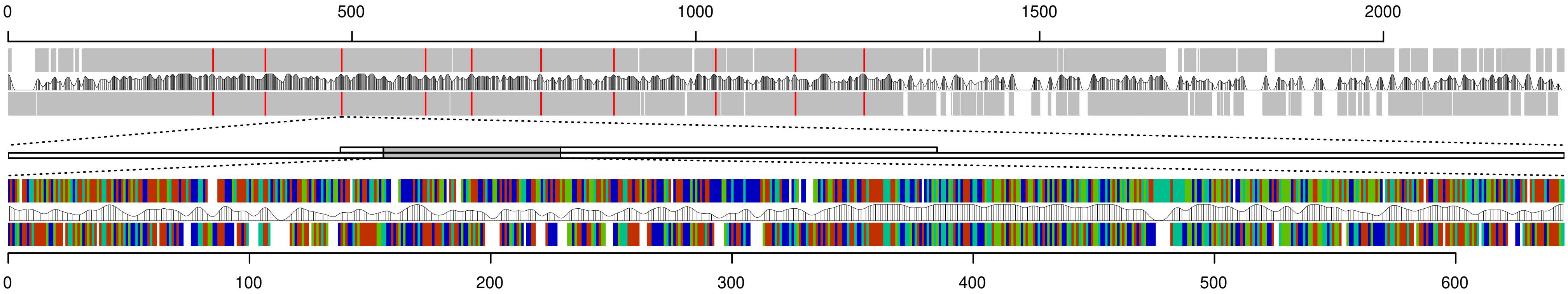

Danio rerio (ENSDART00000079536), Microtus ochrogaster (ENSMOCT00000020267)

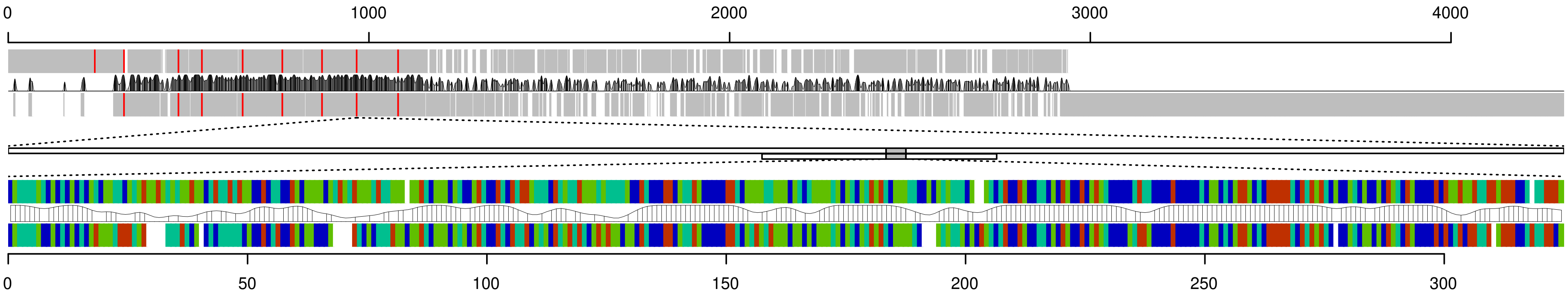

Danio rerio (ENSDART00000076596), Vicugna pacos (ENSVPAT00000005195)

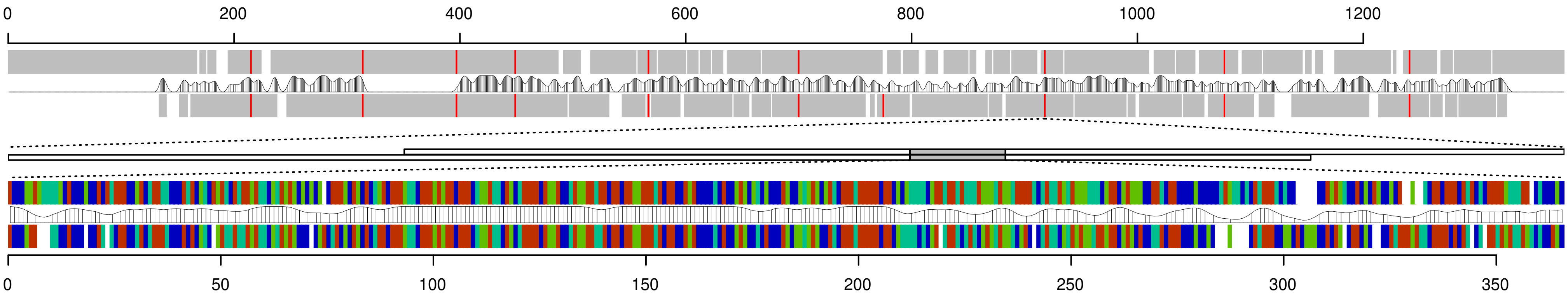

Danio rerio (ENSDART00000158367), Choloepus hoffmanni (ENSCHOT00000007047)

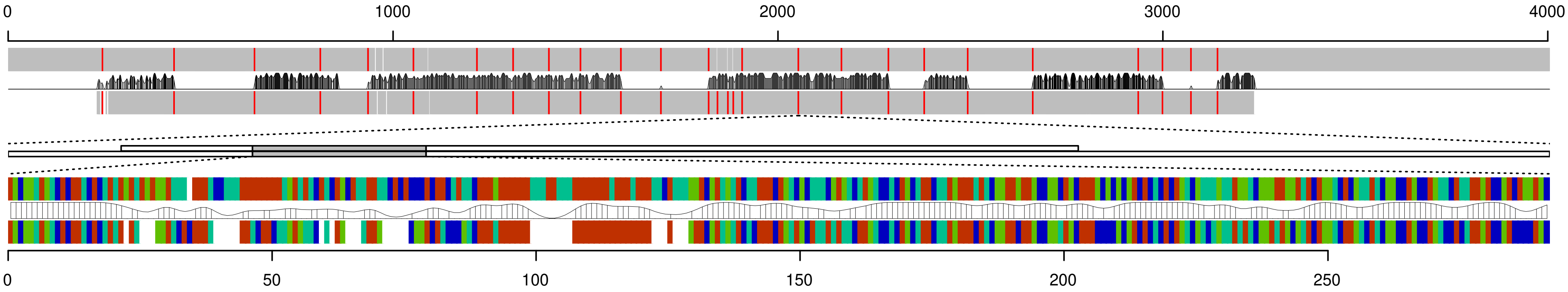

Danio rerio (ENSDART00000148257), Oryctolagus cuniculus (ENSOCUT00000031335)

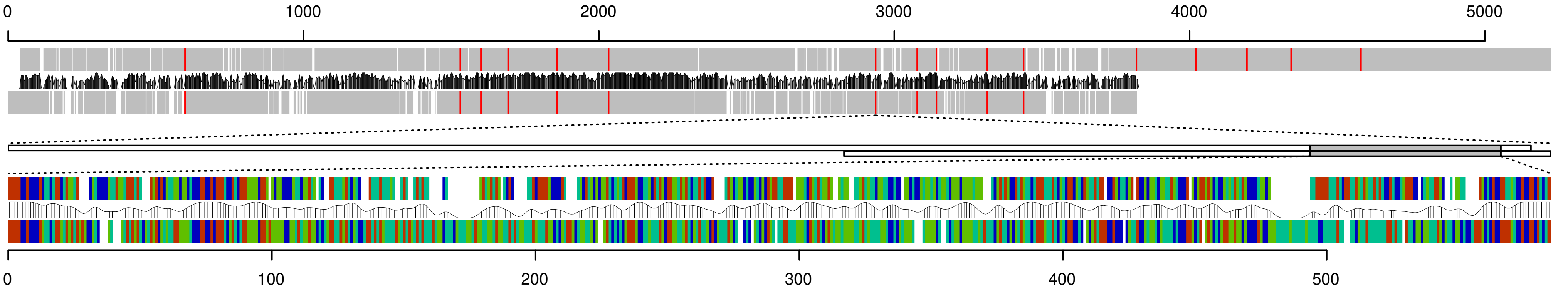

Danio rerio (ENSDART00000051278), Carlito syrichta (ENSTSYT00000044786)

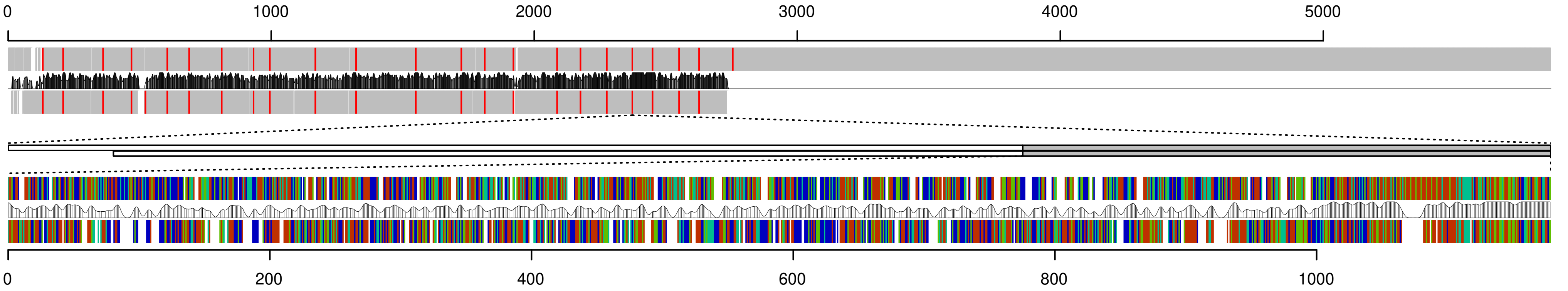

Danio rerio (ENSDART00000143809), Microcebus murinus (ENSMICT00000058891)

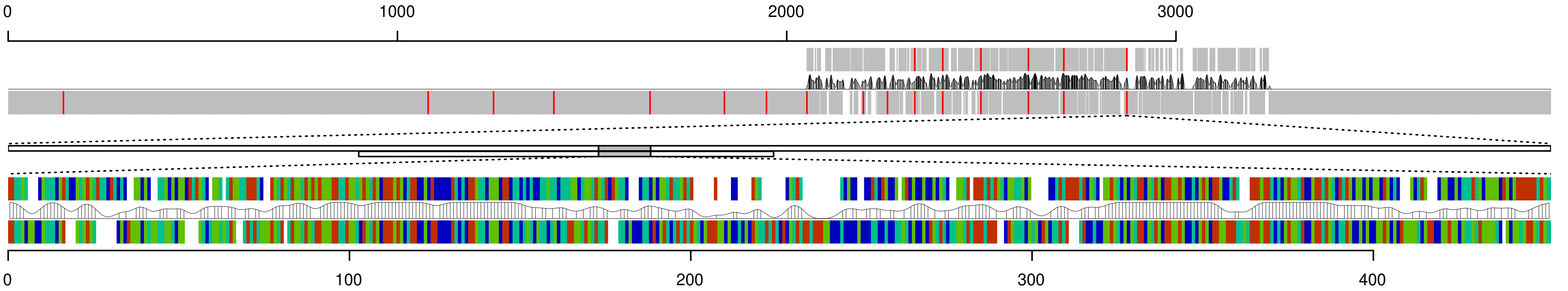

Danio rerio (ENSDART00000146299), Notamacropus eugenii (ENSMEUT00000014742)

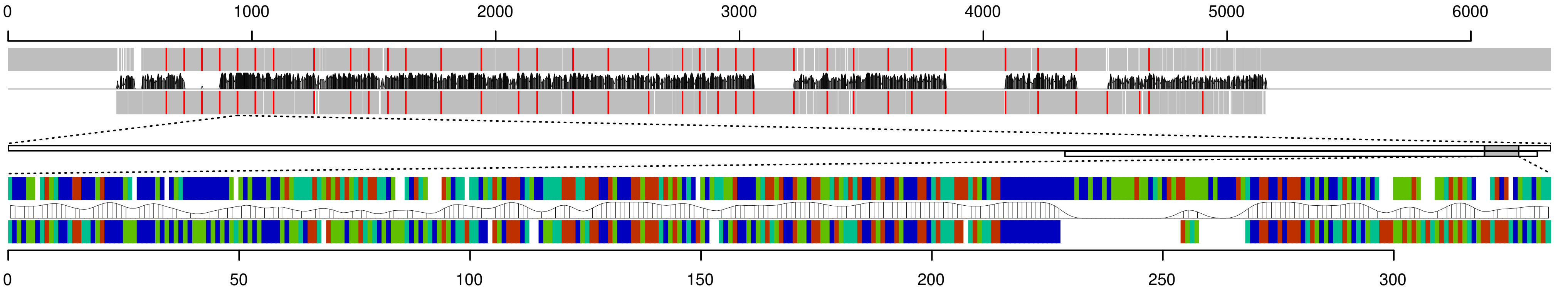

Danio rerio (ENSDART00000128690), Ictidomys tridecemlineatus (ENSSTOT00000034979)

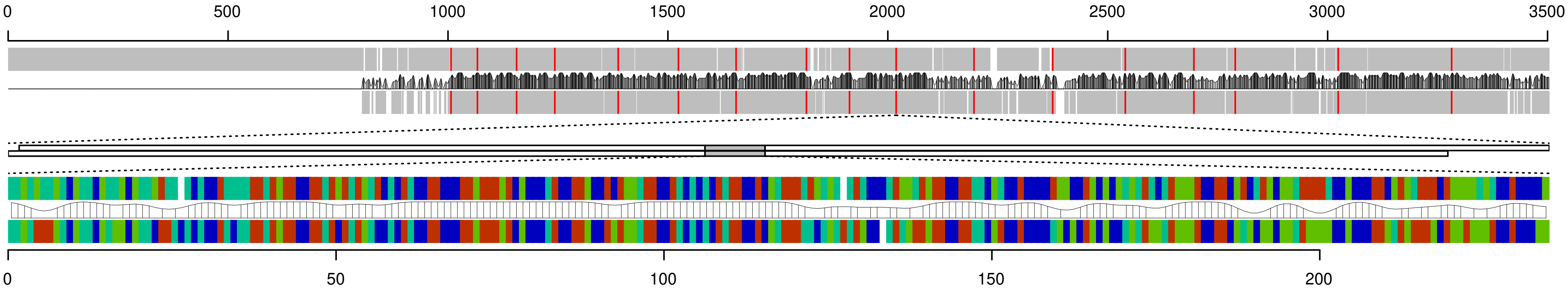

Danio rerio (ENSDART00000054689), Ochotona princeps (ENSOPRT00000010764)

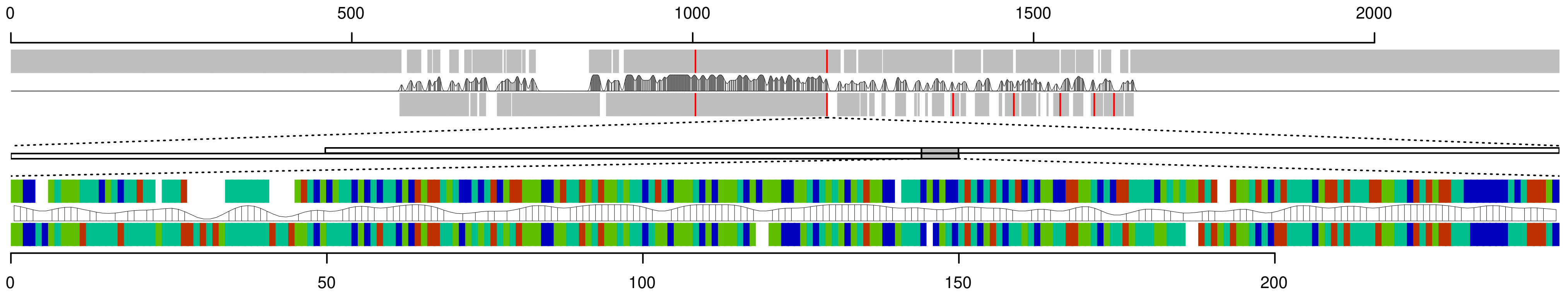

Danio rerio (ENSDART00000085051), Ursus americanus (ENSUAMT00000010584)

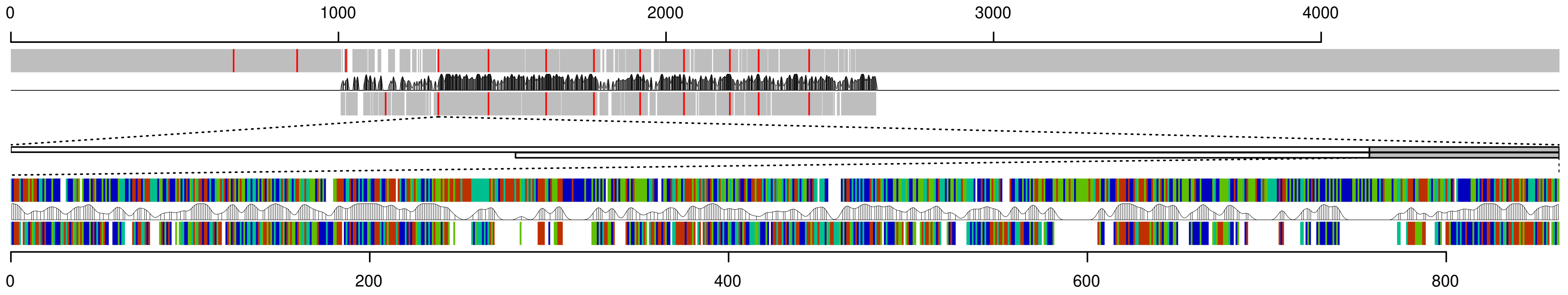

Danio rerio (ENSDART00000191722), Sorex araneus (ENSSART00000010067)

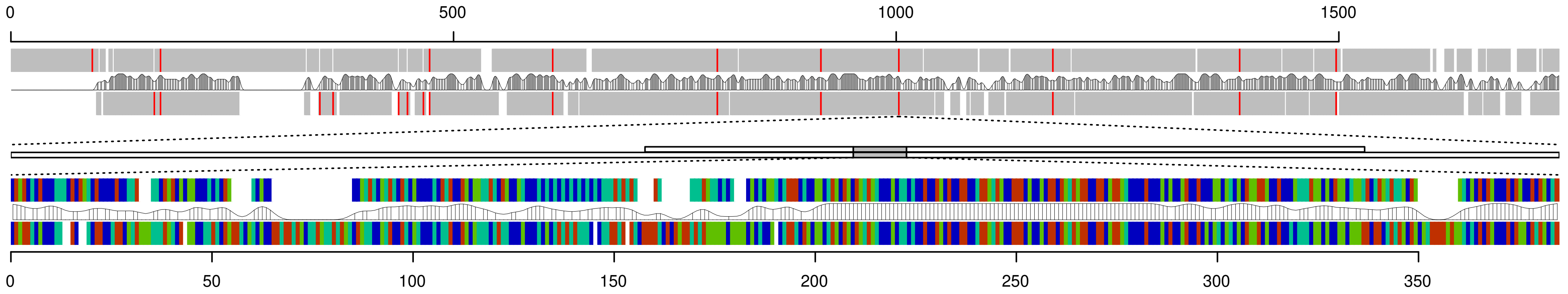

Danio rerio (ENSDART00000142755), Cavia aperea (ENSCAPT00000003296)

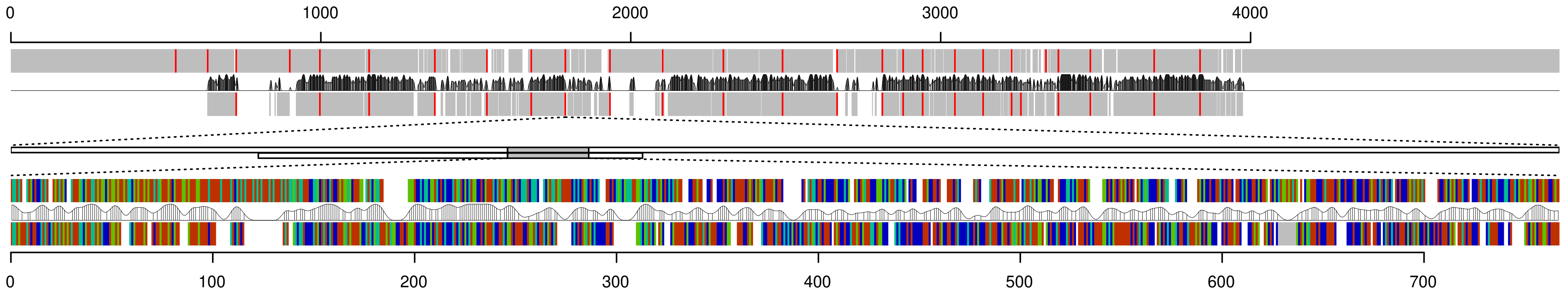

Danio rerio (ENSDART00000114288), Cercocebus atys (ENSCATT00000030017)

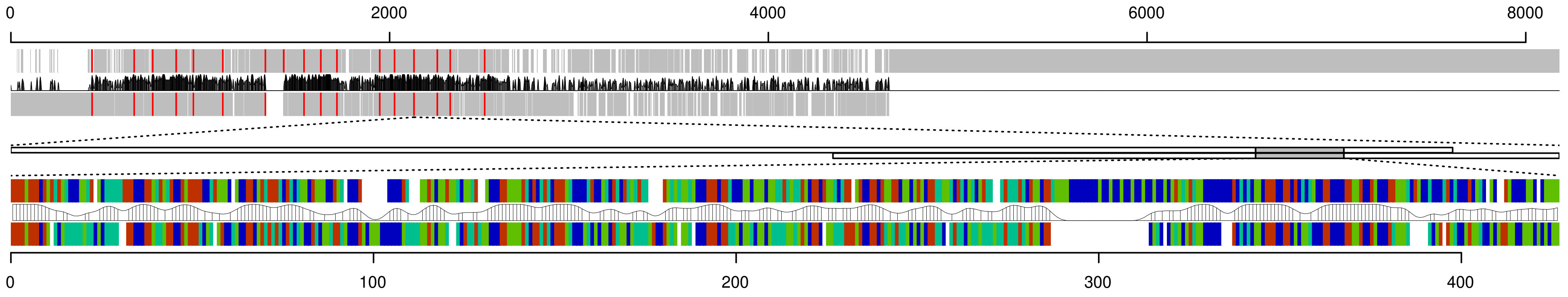

Danio rerio (ENSDART00000143390), Ornithorhynchus anatinus (ENSOANT00000008617)

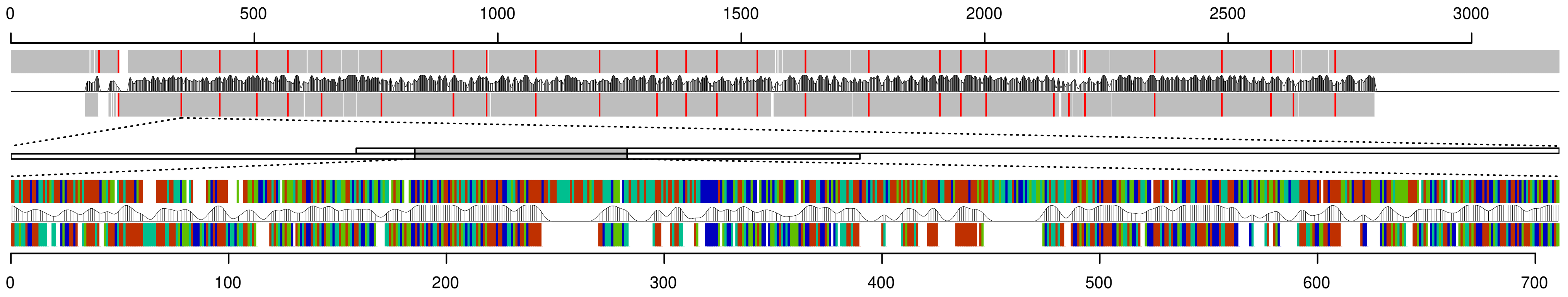

Danio rerio (ENSDART00000036419), Ictidomys tridecemlineatus (ENSSTOT00000012526)

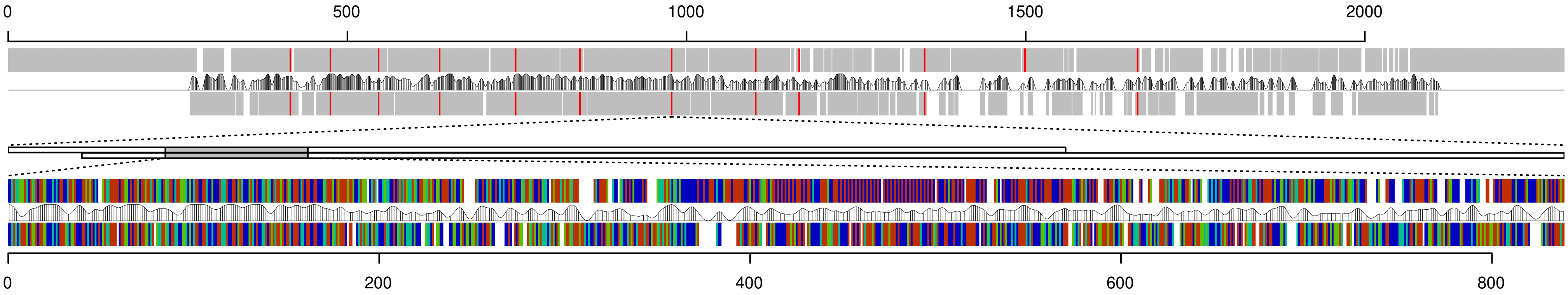

Danio rerio (ENSDART00000137232), Ornithorhynchus anatinus (ENSOANT00000014234)

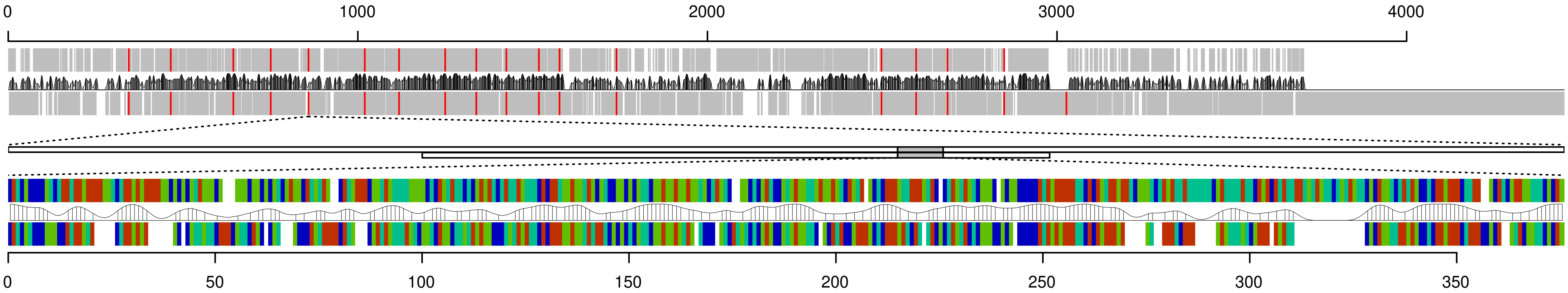

Danio rerio (ENSDART00000134121), Dipodomys ordii (ENSDORT00000004261)

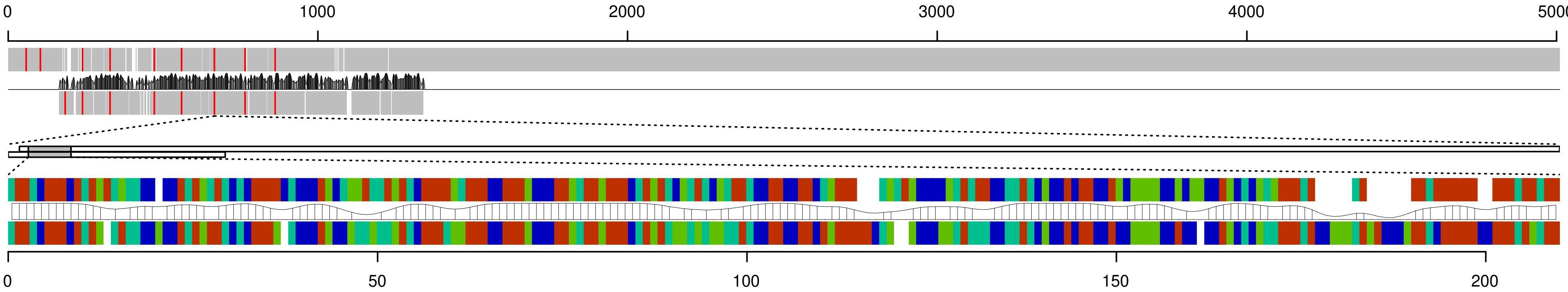

Danio rerio (ENSDART00000052404), Notamacropus eugenii (ENSMEUT00000002385)

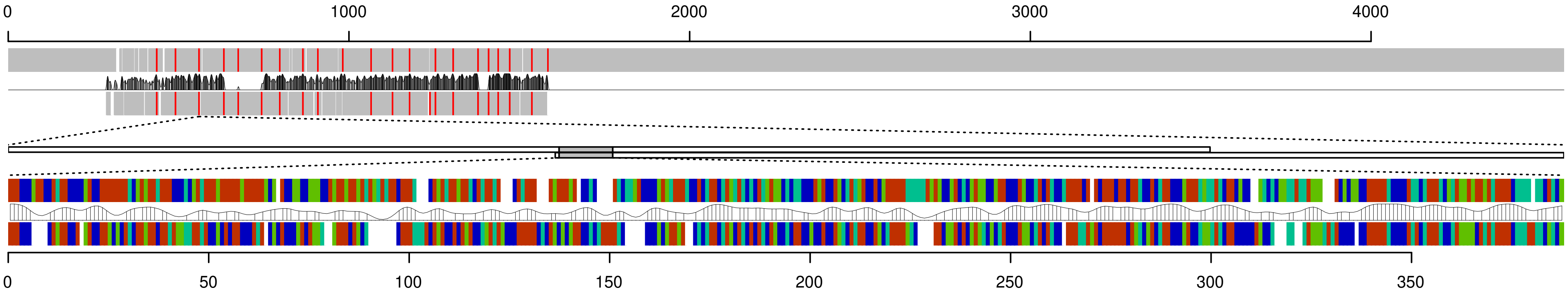

Danio rerio (ENSDART00000044128), Chinchilla lanigera (ENSCLAT00000010275)

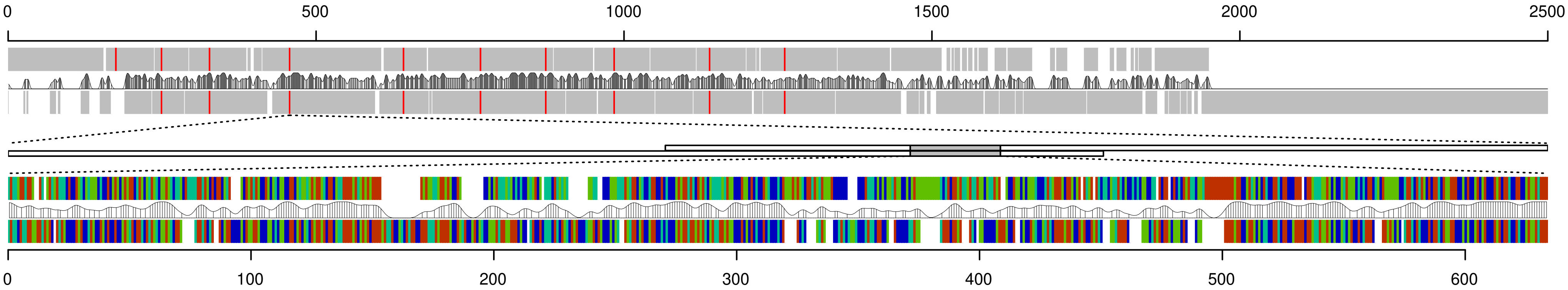

Danio rerio (ENSDART00000085051), Sarcophilus harrisii (ENSSHAT00000014019)

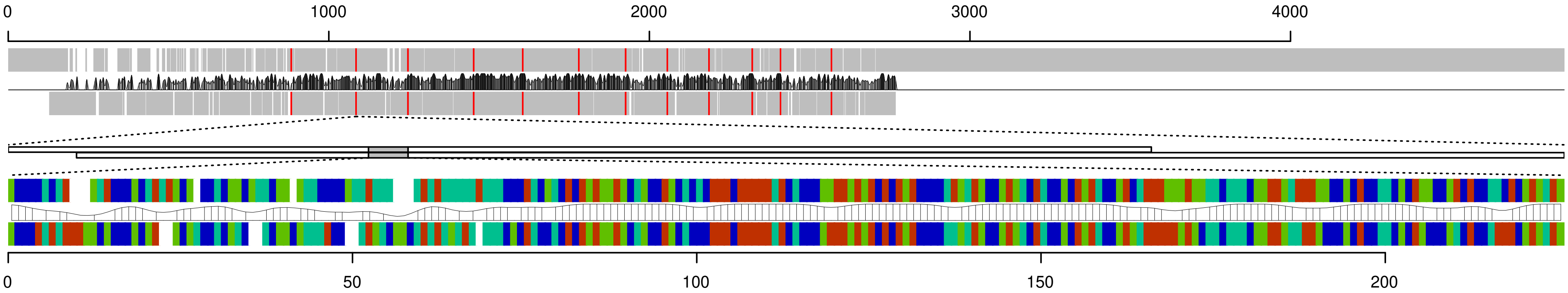

Danio rerio (ENSDART00000103434), Dipodomys ordii (ENSDORT00000009106)

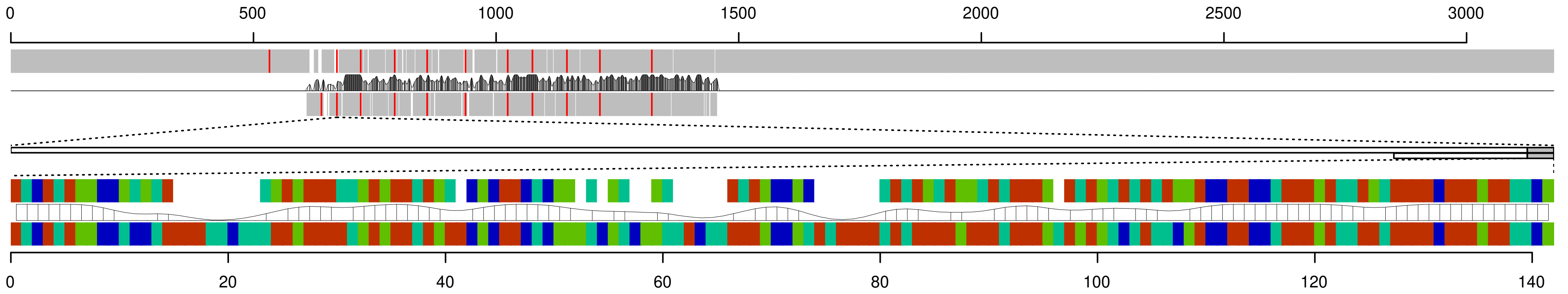

Danio rerio (ENSDART00000149957), Ornithorhynchus anatinus (ENSOANT00000018263)

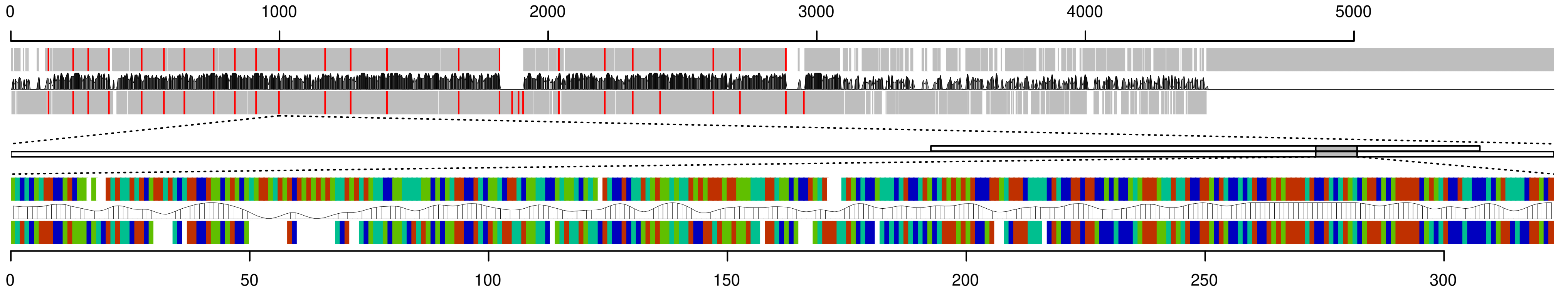

Danio rerio (ENSDART00000005562), Ursus americanus (ENSUAMT00000029107)

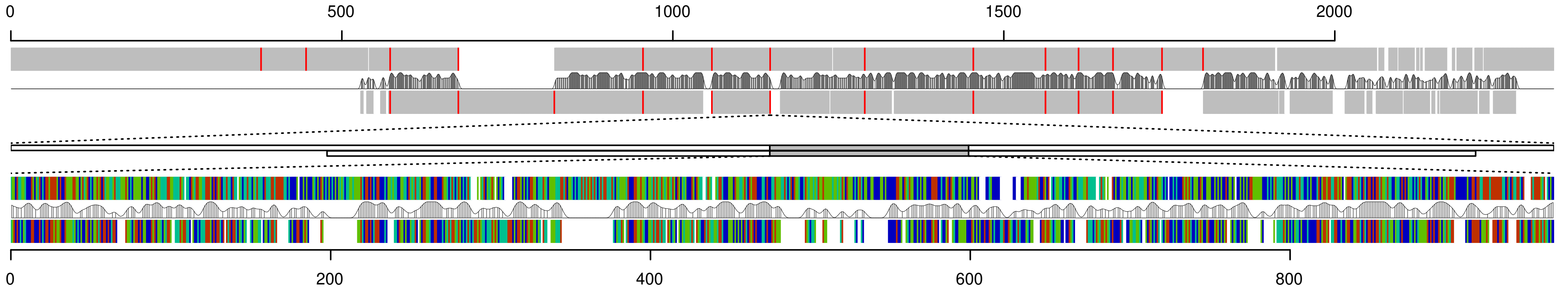

Danio rerio (ENSDART00000147443), Spermophilus dauricus (ENSSDAT00000011752)

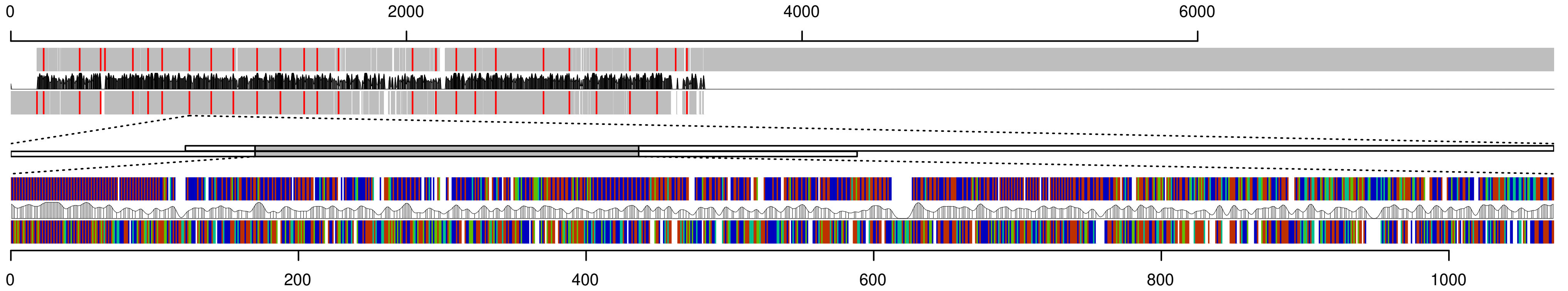

Danio rerio (ENSDART00000140890), Propithecus coquereli (ENSPCOT00000026385)

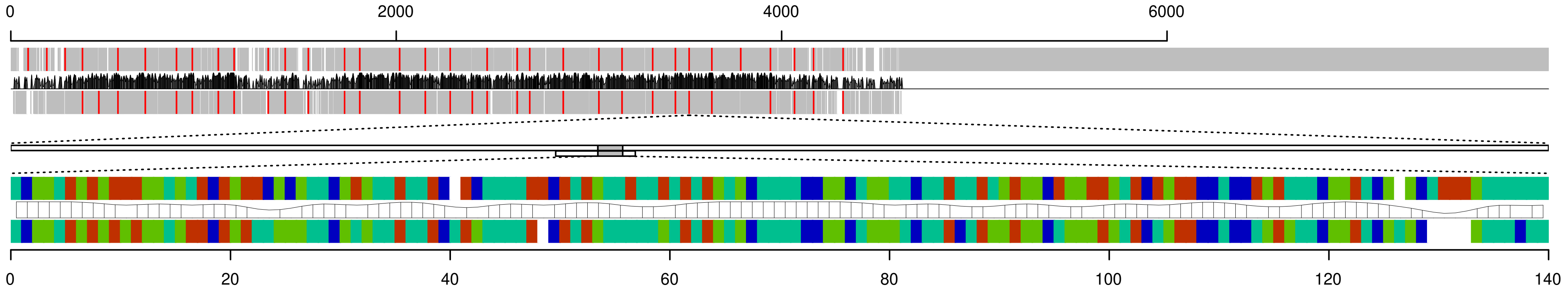

Danio rerio (ENSDART00000162317), Monodelphis domestica (ENSMODT00000007462)

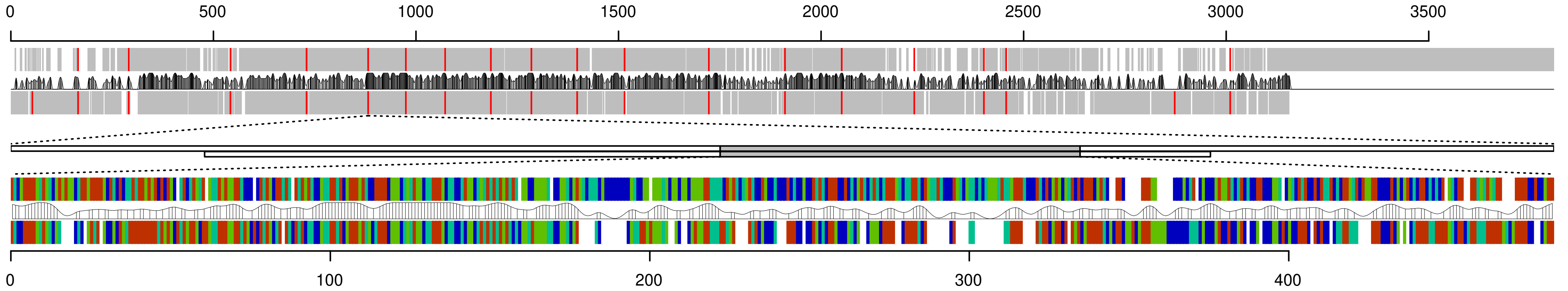

Danio rerio (ENSDART00000085481), Procavia capensis (ENSPCAT00000011535)

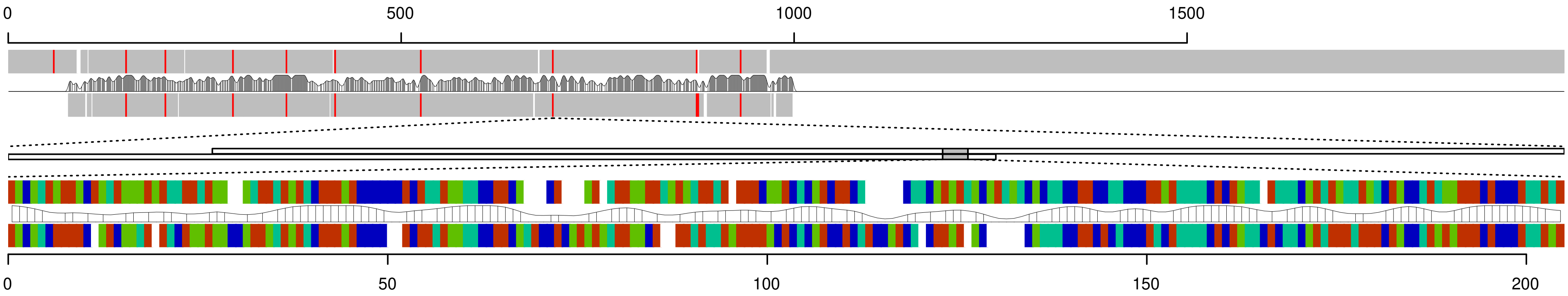

Danio rerio (ENSDART00000128690), Ochotona princeps (ENSOPRT00000003951)

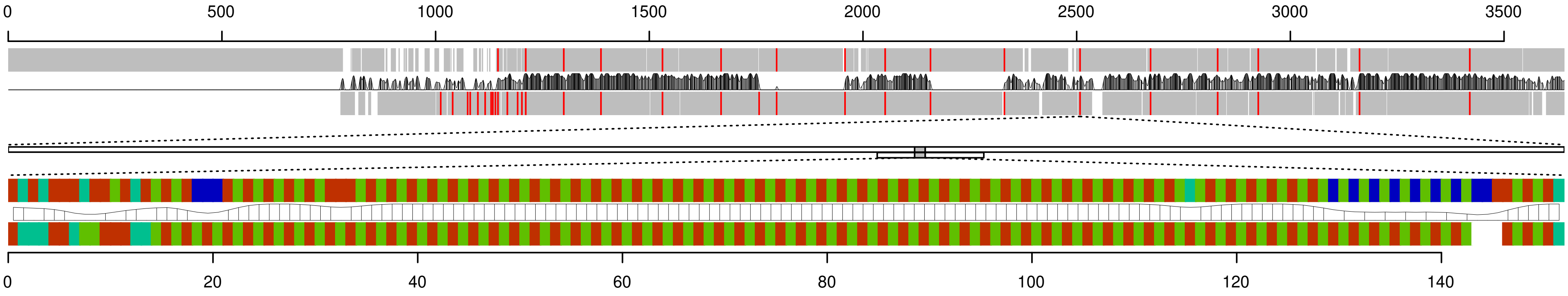

Danio rerio (ENSDART00000087441), Dasypus novemcinctus (ENSDNOT00000006855)

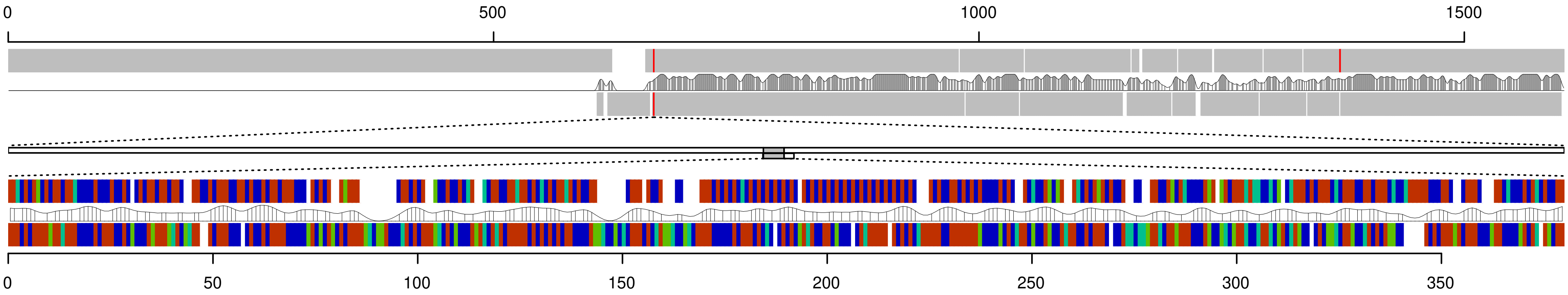

Danio rerio (ENSDART00000173228), Ochotona princeps (ENSOPRT00000011909)

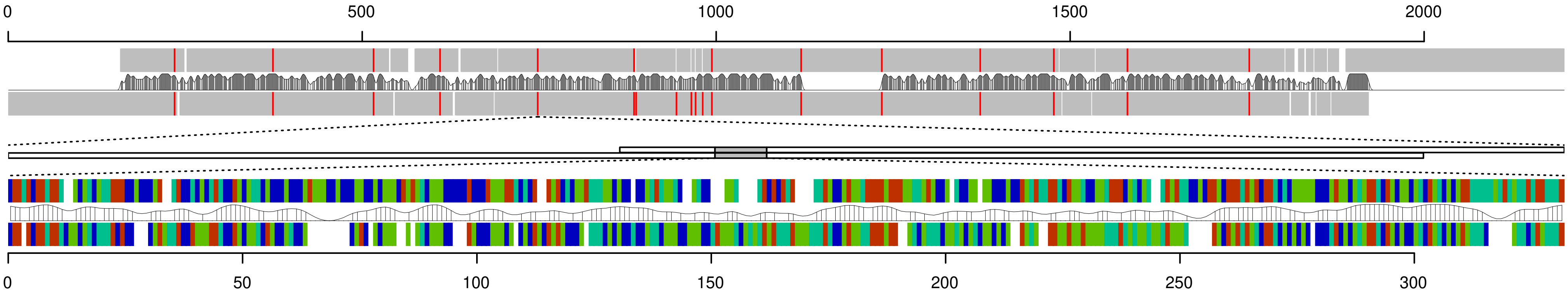

Danio rerio (ENSDART00000052404), Macaca fascicularis (ENSMFAT00000065032)

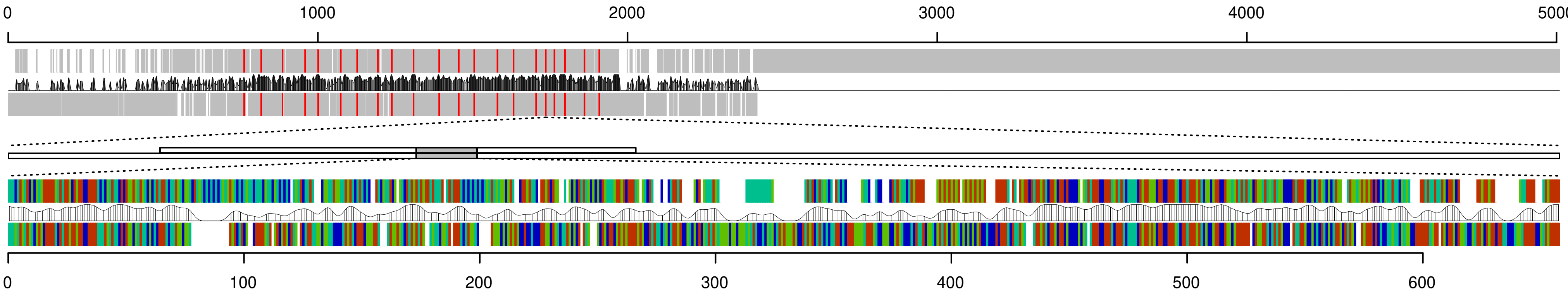

Danio rerio (ENSDART00000149395), Choloepus hoffmanni (ENSCHOT00000005015)

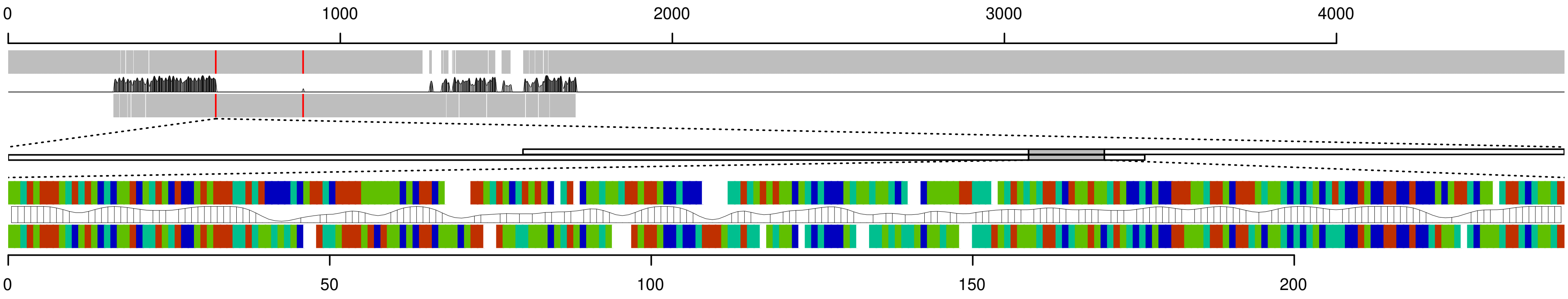

Danio rerio (ENSDART00000151193), Echinops telfairi (ENSETET00000001325)

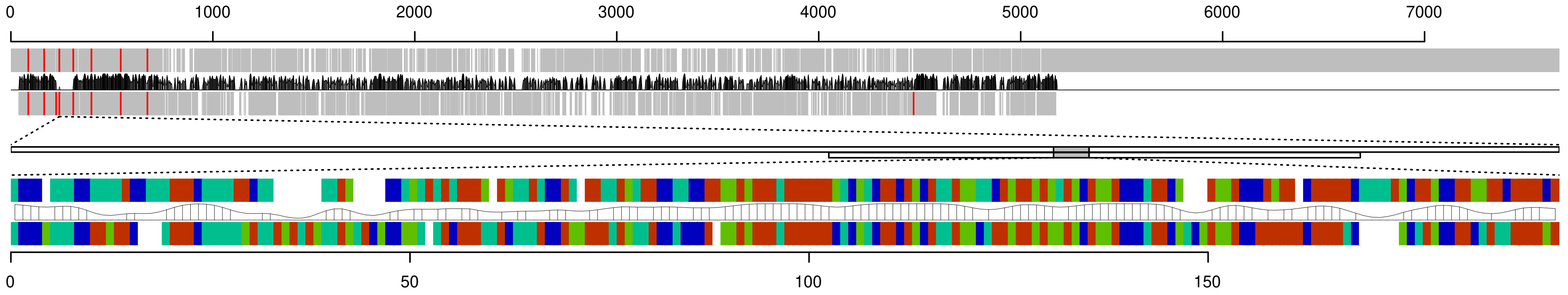

Danio rerio (ENSDART00000151193), Ailuropoda melanoleuca (ENSAMET00000020184)

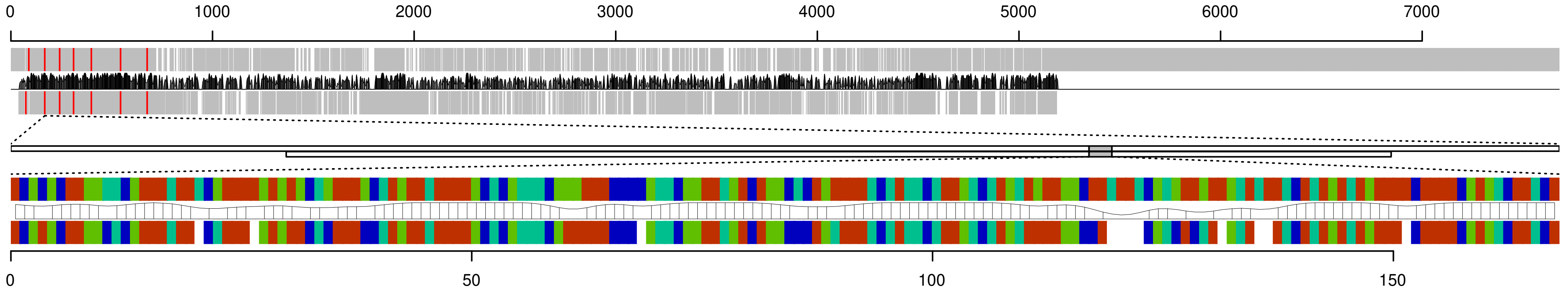

Danio rerio (ENSDART00000132691), Urocitellus parryii (ENSUPAT00010017447)

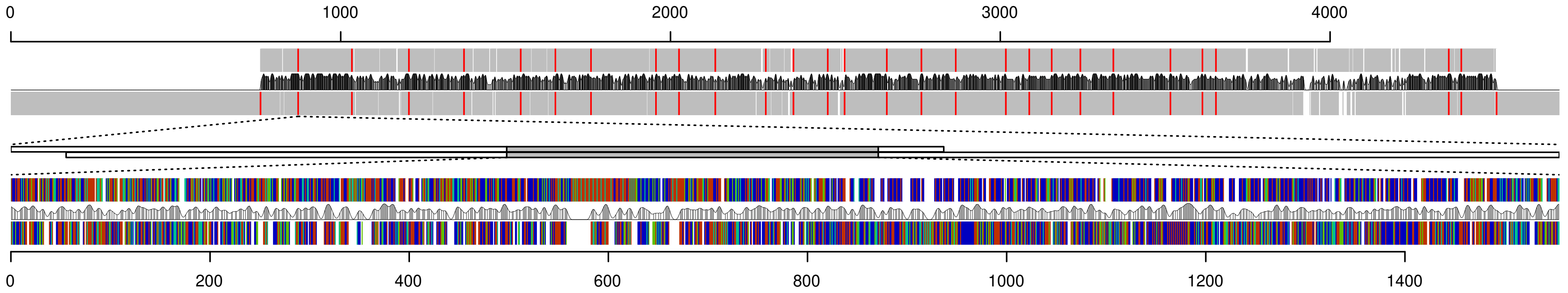

Danio rerio (ENSDART00000112160), Echinops telfairi (ENSETET00000001508)

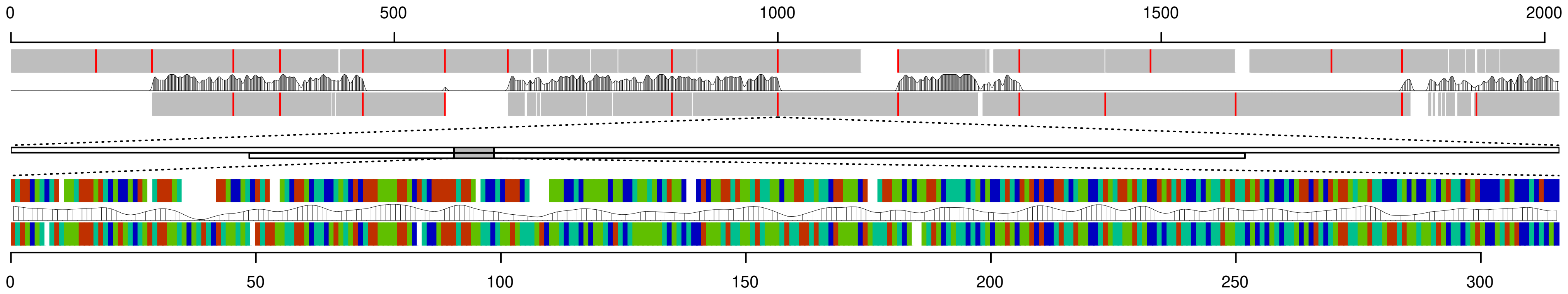

Danio rerio (ENSDART00000111823), Mus musculus (ENSMUST00000030676)

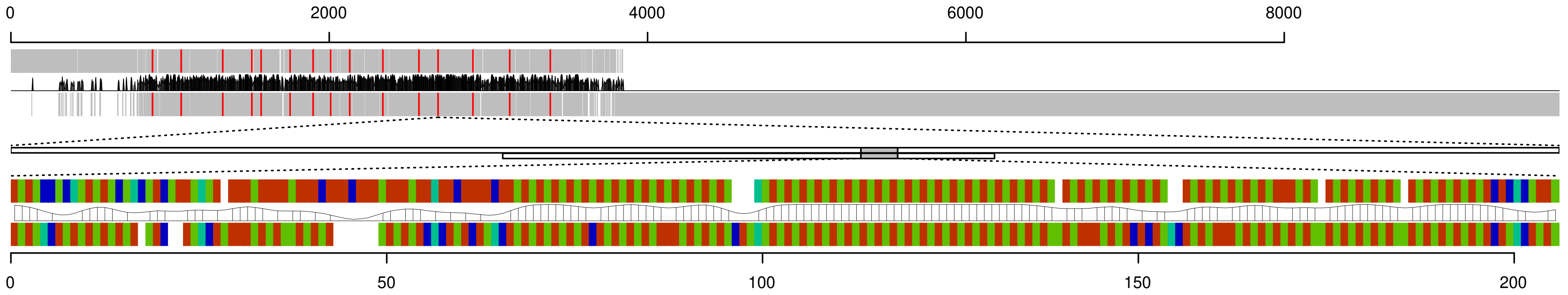

Danio rerio (ENSDART00000159727), Pteropus vampyrus (ENSPVAT00000017804)

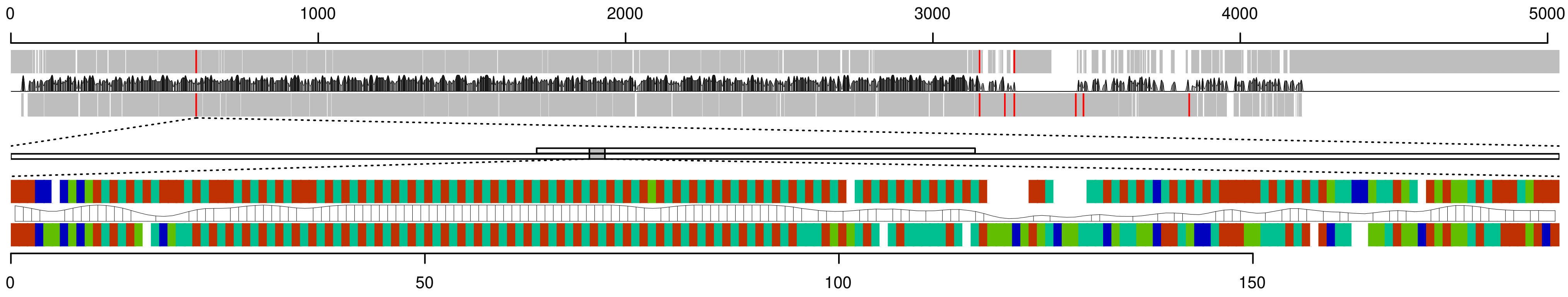

Danio rerio (ENSDART00000132405), Ursus americanus (ENSUAMT00000039830)

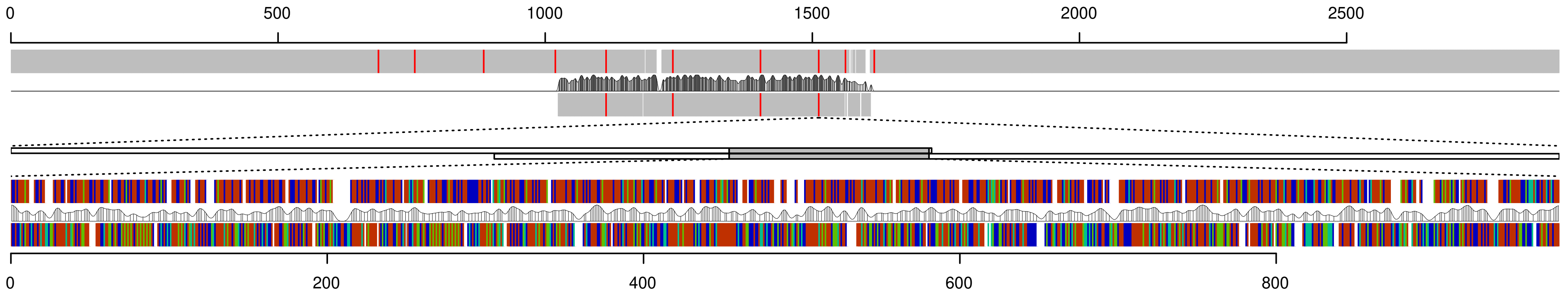

Danio rerio (ENSDART00000029824), Myotis lucifugus (ENSMLUT00000015779)

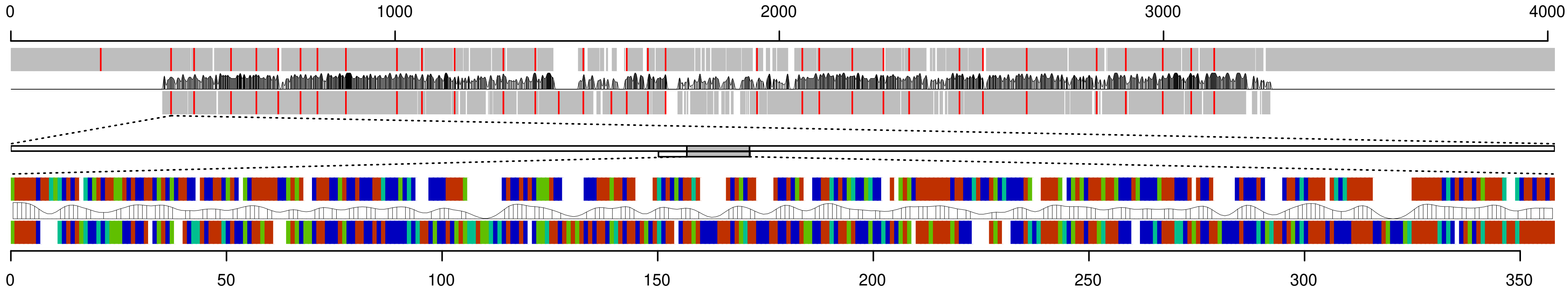

Danio rerio (ENSDART00000051271), Ursus americanus (ENSUAMT00000041194)

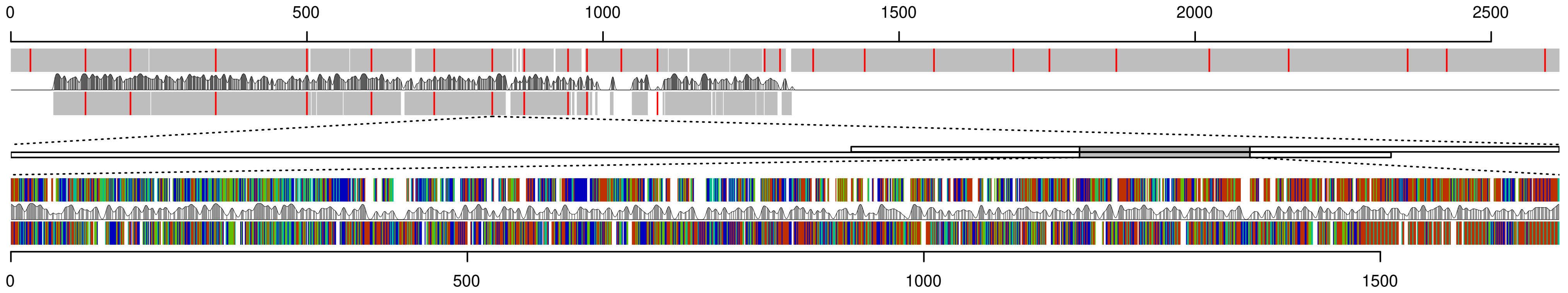

Danio rerio (ENSDART00000076596), Vombatus ursinus (ENSVURT00010015276)

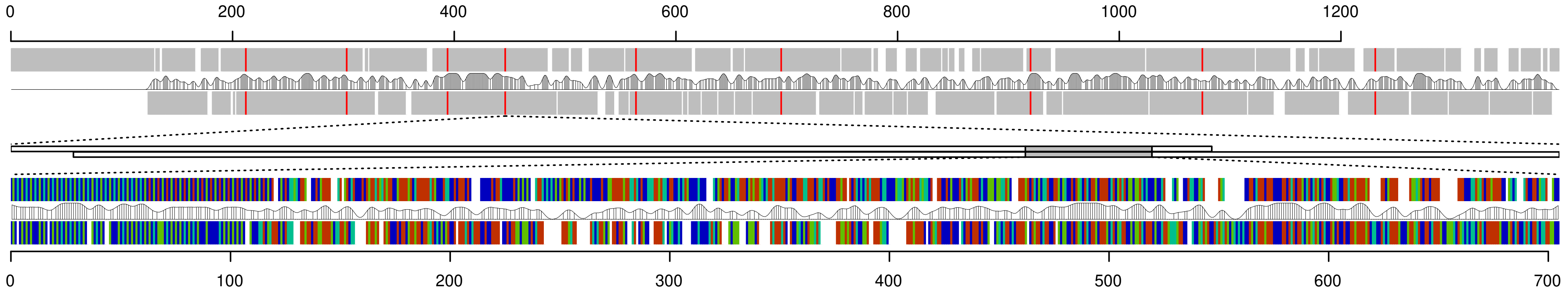

Danio rerio (ENSDART00000163449), Ictidomys tridecemlineatus (ENSSTOT00000001283)

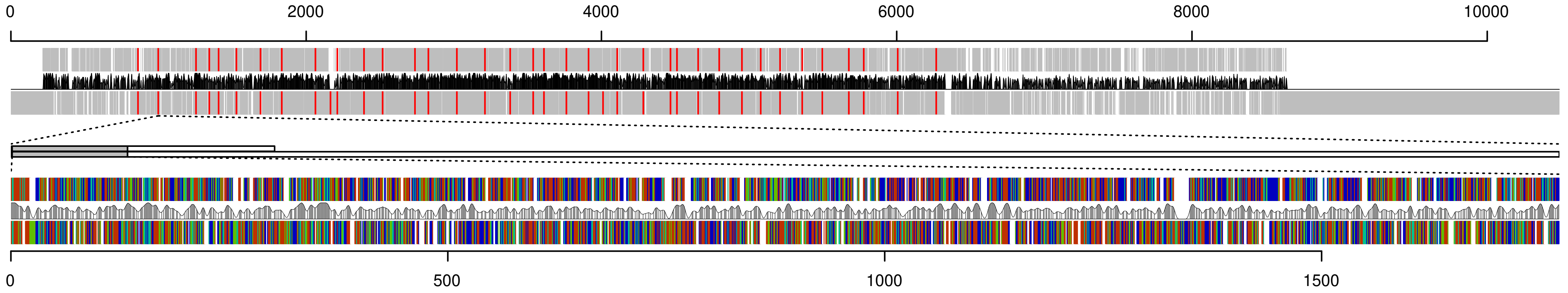

Danio rerio (ENSDART00000162945), Cebus capucinus (ENSCCAT00000021477)

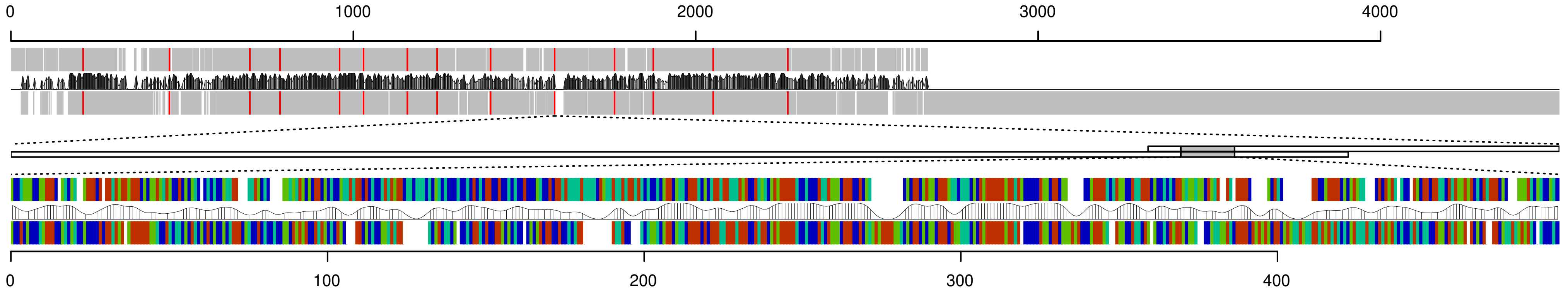

Danio rerio (ENSDART00000034004), Notamacropus eugenii (ENSMEUT00000003145)

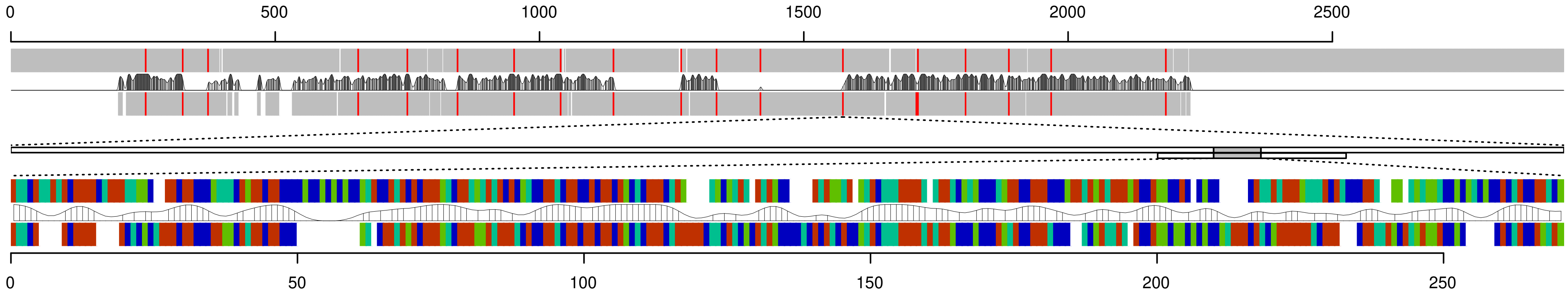

Danio rerio (ENSDART00000034004), Rhinopithecus bieti (ENSRBIT00000041980)

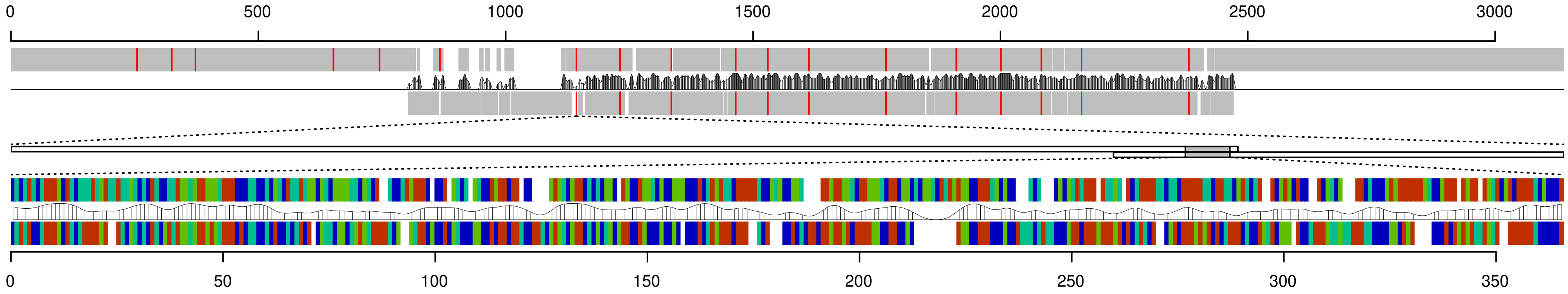

Danio rerio (ENSDART00000187248), Equus caballus (ENSECAT00000021463)

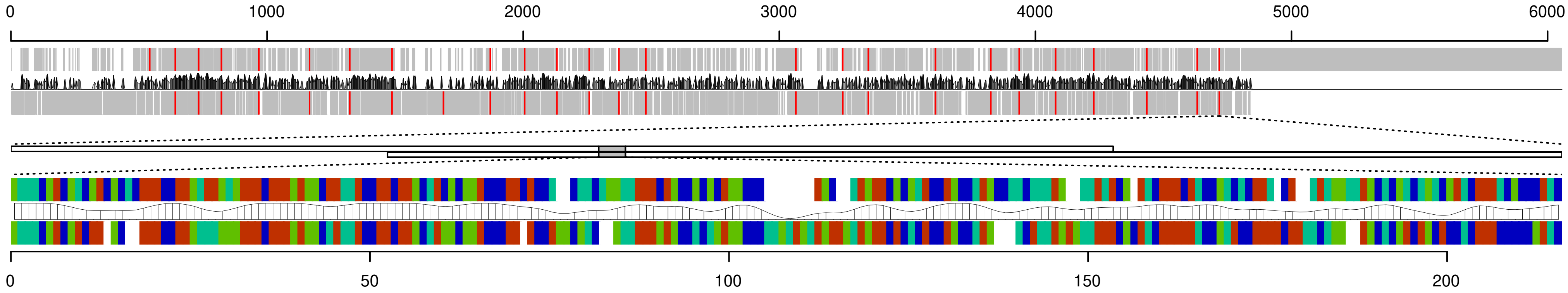

Danio rerio (ENSDART00000177989), Notamacropus eugenii (ENSMEUT00000003276)

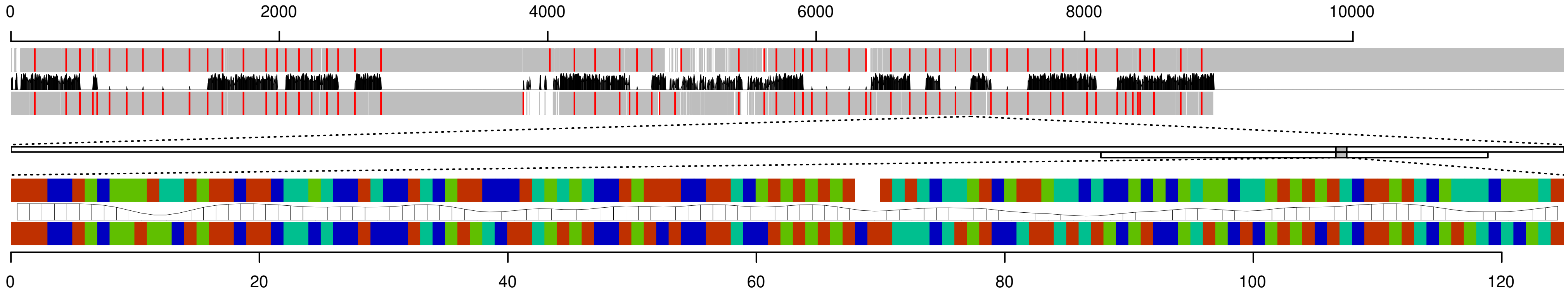

Danio rerio (ENSDART00000083367), Echinops telfairi (ENSETET00000014376)

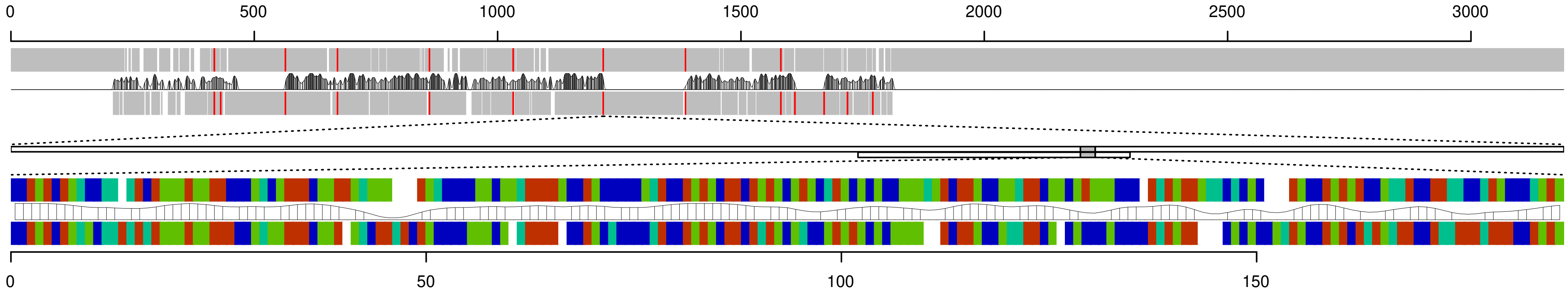

Danio rerio (ENSDART00000138748), Echinops telfairi (ENSETET00000010924)

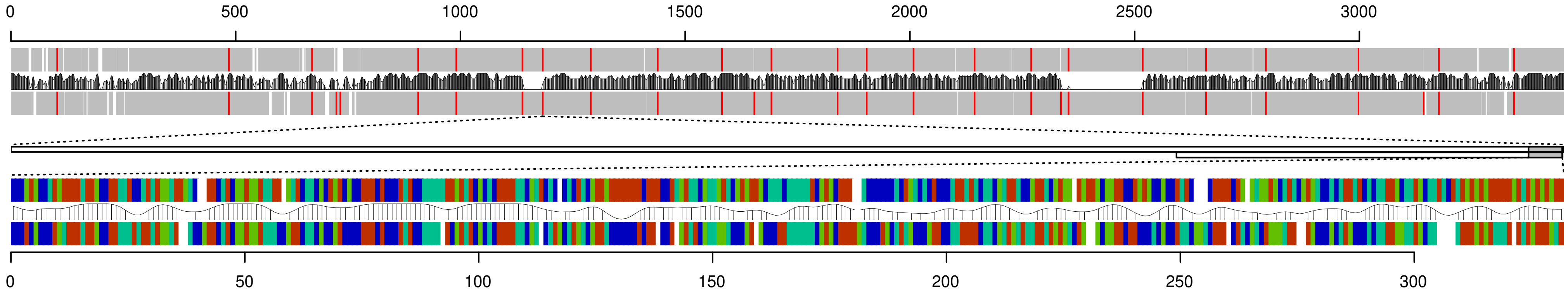

Supplement: Supplementary file 6 — Transcript and intron alignments for points in Fig. S14. Each panel shows the maximally scoring alignment between D. rerio and teleost intron orthologues (lower) and transcript alignment (upper) used to establish the intron orthology. Grey, white and red parts indicate aligned exonic sequence, gaps and positions of intron meta-characters respectively. Colours in intron alignment represent bases (A blue, C cyan, G green, T brown, N grey, gap white). Curves lying between sequence representations show a normal kernel density smoothed estimate of local similarity (9 bp window, standard deviation two); vertical lines indicate matches. Region between exon and intron alignments indicates the location of the maximally scoring alignment in the introns. Upper sequence D. rerio. Files 6–10 and 11–15 contain alignments to teleost and mammalian sequences respectively. Each file corresponds to one panel in Fig. S17 and to one specific teleost size class: Files 6,11: long (E,J), 7,12: medium (D,I), 8,13: short.2 (C,H), 9,14 short (B, G) and 10,15 ctl (A,F). [file 12864_2022_8760_MOESM6_ESM.zip › 12864_2022_8760_MOESM11_ESM.pdf]
