# Supplementary material for: Intron size minimisation in teleosts
Source: BMC Genomics. 2022 Sep 1;23:628. doi: 10.1186/s12864-022-08760-w (PMC9438311; doi:10.1186/s12864-022-08760-w)

Danio rerio (ENSDART00000005562), Ornithorhynchus anatinus (ENSOANT00000002524)

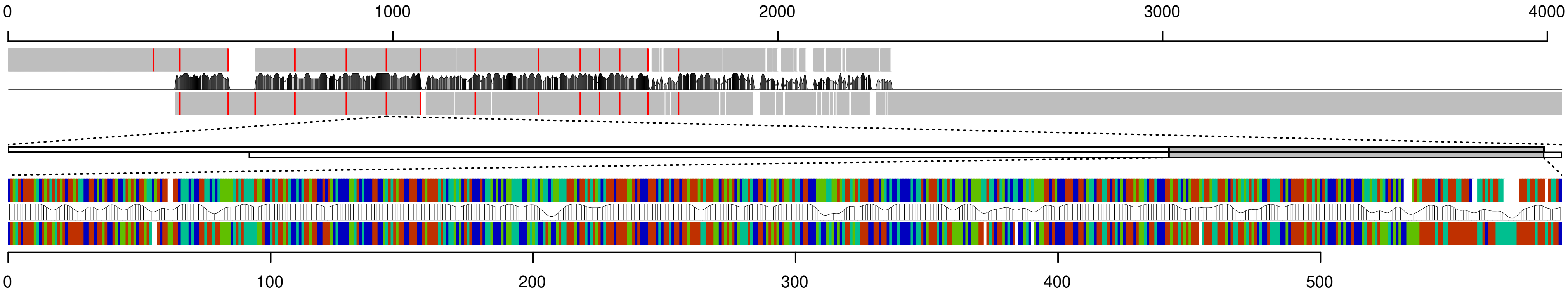

Danio rerio (ENSDART00000163766), Chinchilla lanigera (ENSCLAT00000002823)

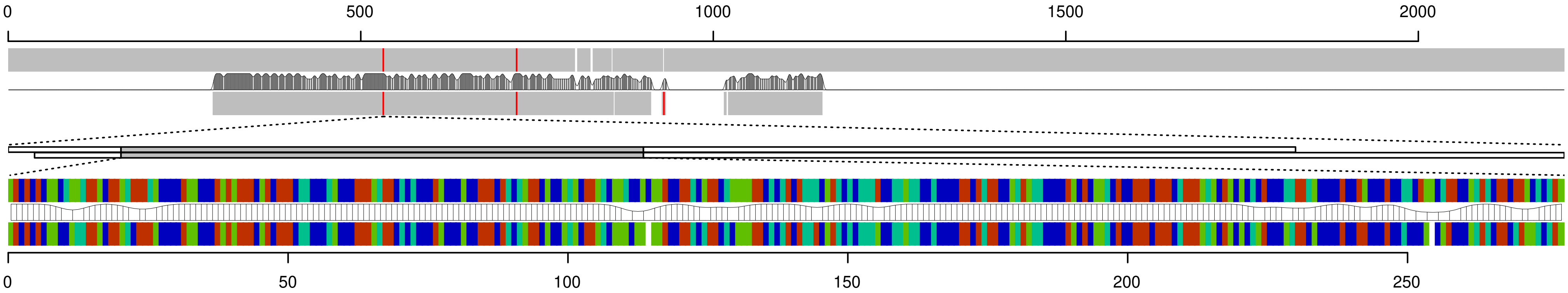

Danio rerio (ENSDART00000122037), Phascolarctos cinereus (ENSPCIT00000044855)

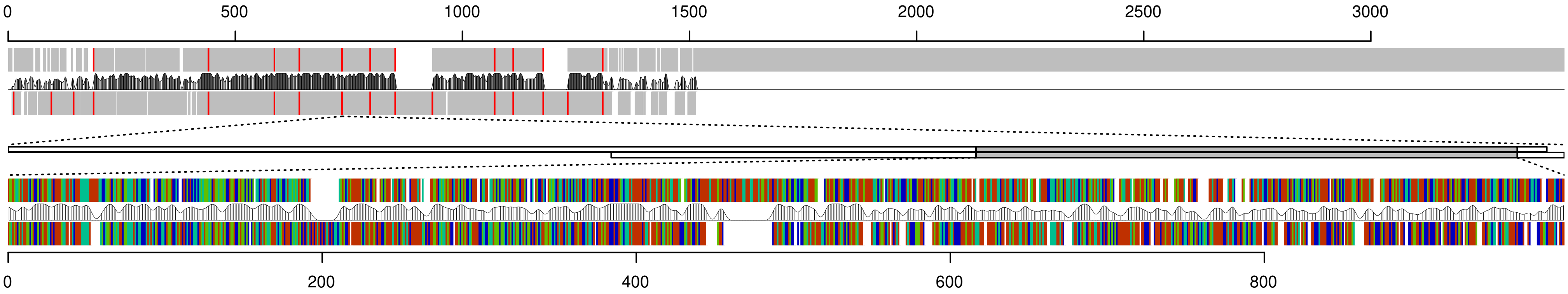

Danio rerio (ENSDART00000111537), Sorex araneus (ENSSART00000011686)

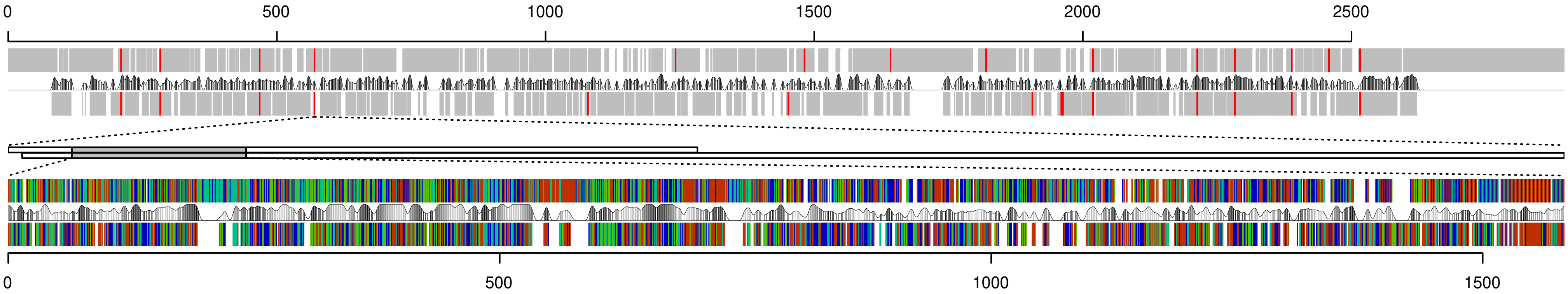

Danio rerio (ENSDART00000159464), Monodelphis domestica (ENSMODT00000010289)

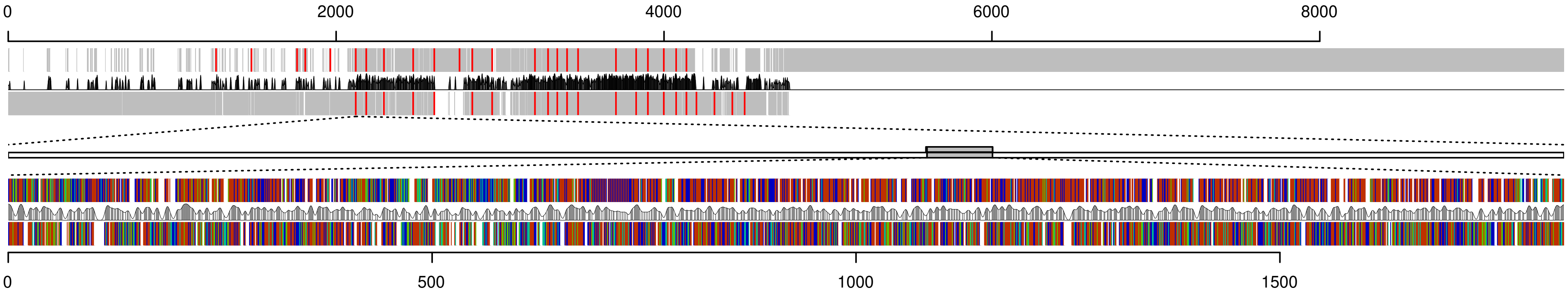

Danio rerio (ENSDART00000122037), Sarcophilus harrisii (ENSSHAT00000010831)

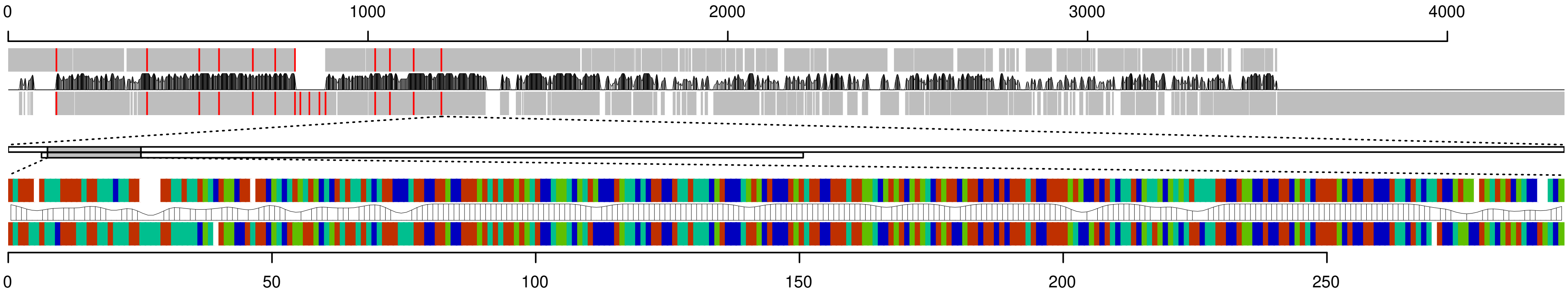

Danio rerio (ENSDART00000074789), Chinchilla lanigera (ENSCLAT00000010866)

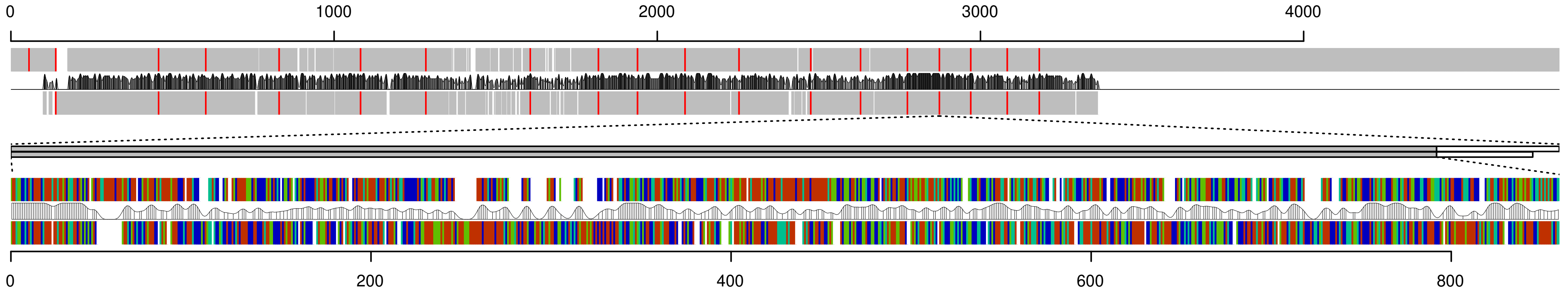

Danio rerio (ENSDART00000165458), Phascolarctos cinereus (ENSPCIT00000000717)

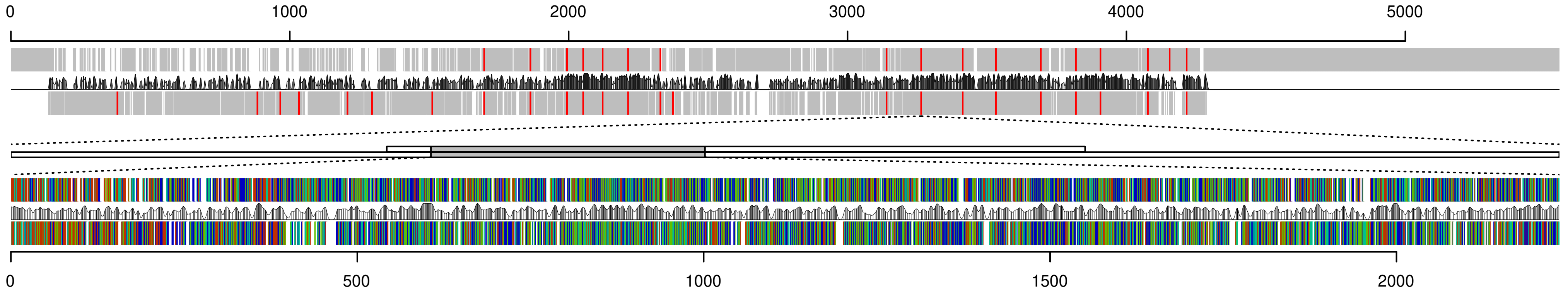

Danio rerio (ENSDART00000077435), Erinaceus europaeus (ENSEEUT00000008849)

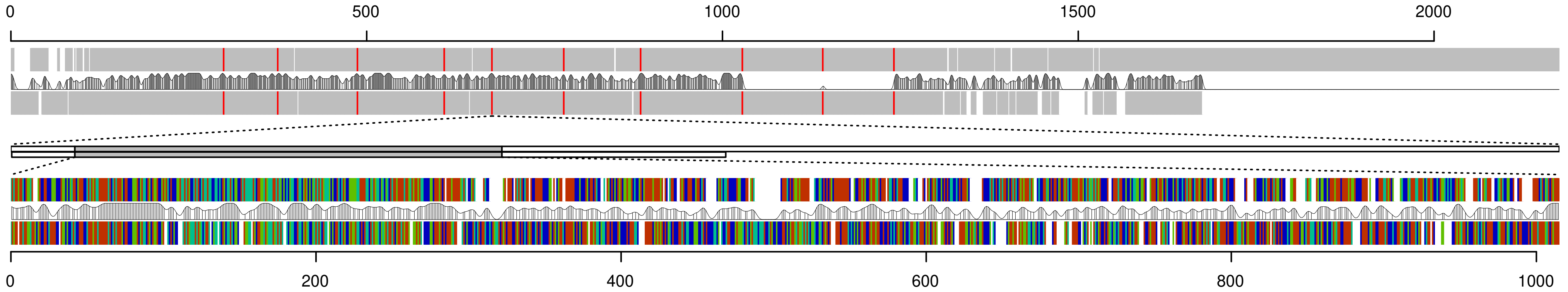

Danio rerio (ENSDART00000103434), Cebus capucinus (ENSCCAT00000048423)

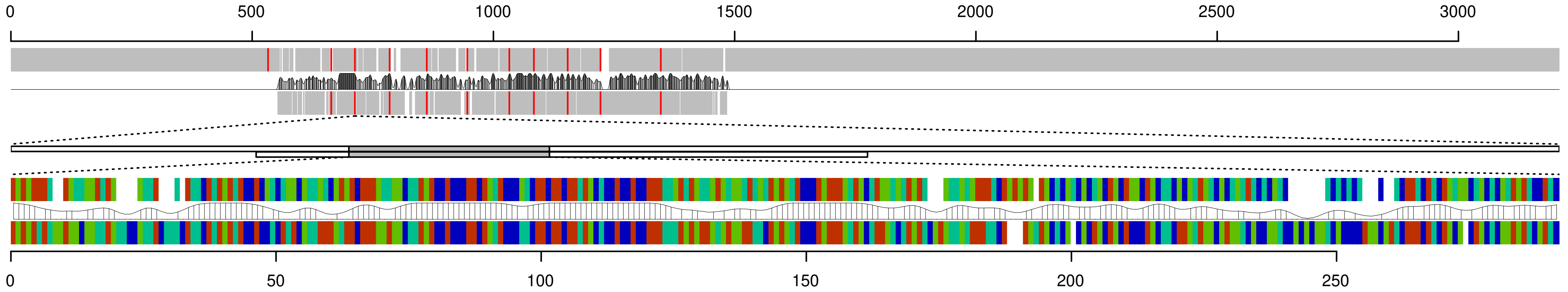

Danio rerio (ENSDART00000148257), Ornithorhynchus anatinus (ENSOANT00000009330)

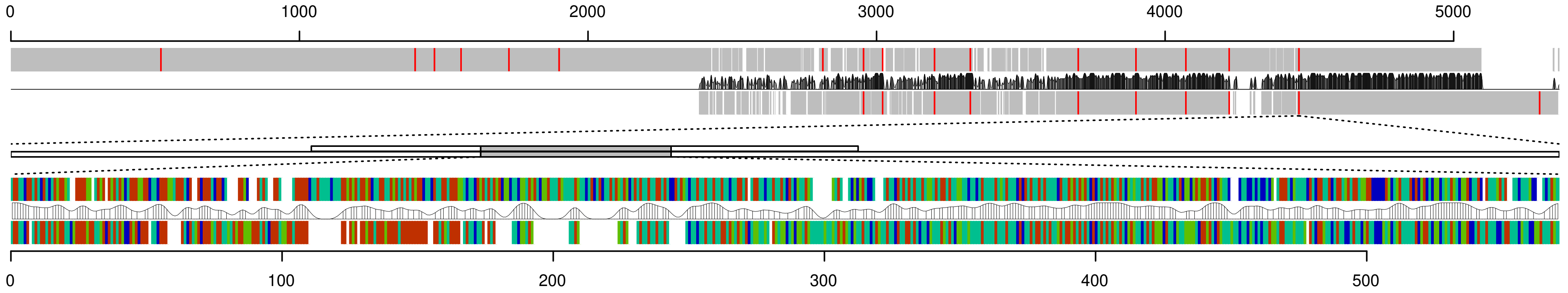

Danio rerio (ENSDART00000103146), Microcebus murinus (ENSMICT00000011703)

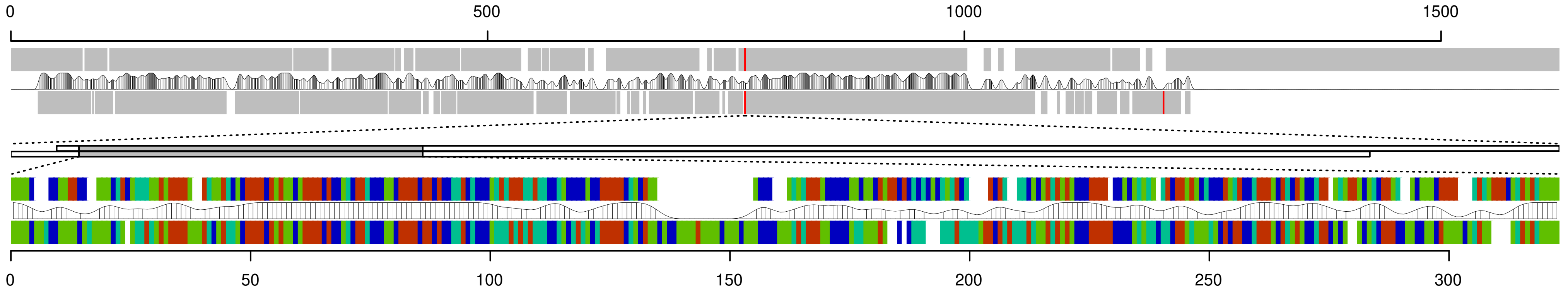

Danio rerio (ENSDART00000127923), Sarcophilus harrisii (ENSSHAT00000009757)

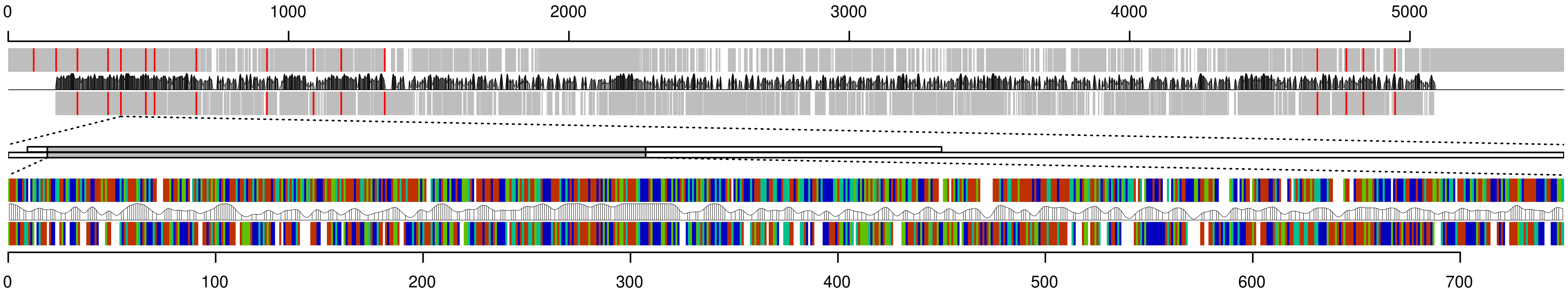

Danio rerio (ENSDART00000052404), Chinchilla lanigera (ENSCLAT00000007630)

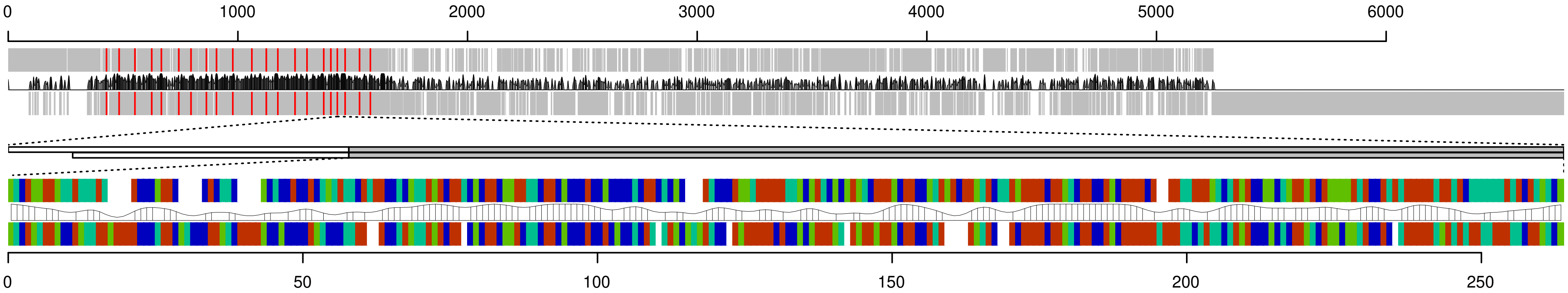

Danio rerio (ENSDART00000112329), Felis catus (ENSFCAT00000084875)

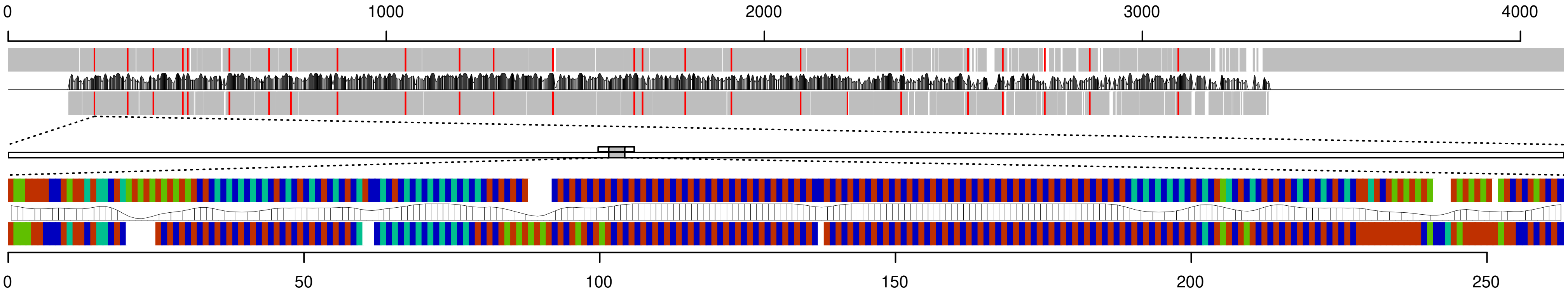

Danio rerio (ENSDART00000158495), Equus caballus (ENSECAT00000065838)

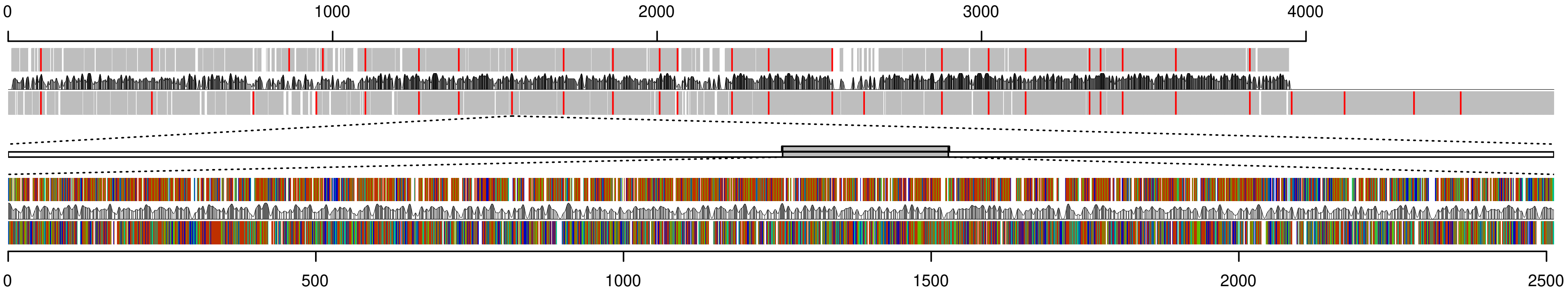

Danio rerio (ENSDART00000187248), Bos mutus (ENSBMUT00000035849)

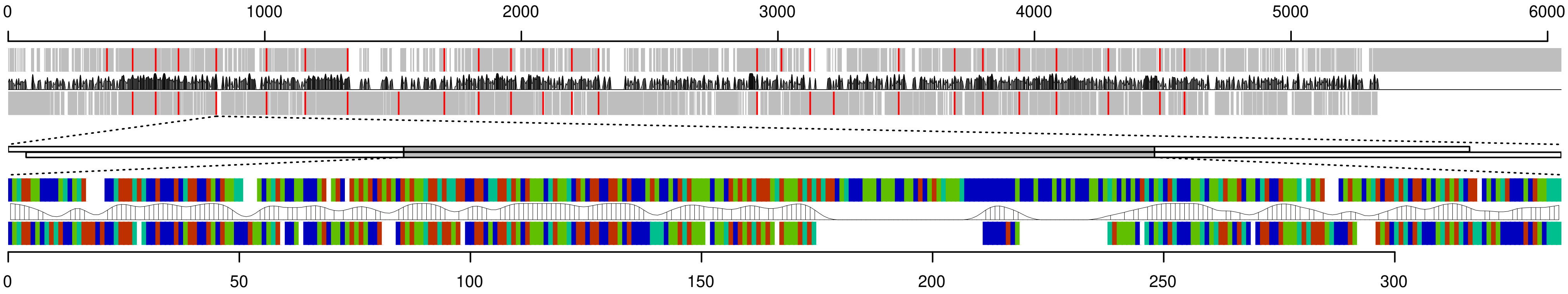

Danio rerio (ENSDART00000129598), Loxodonta africana (ENSLAFT00000014287)

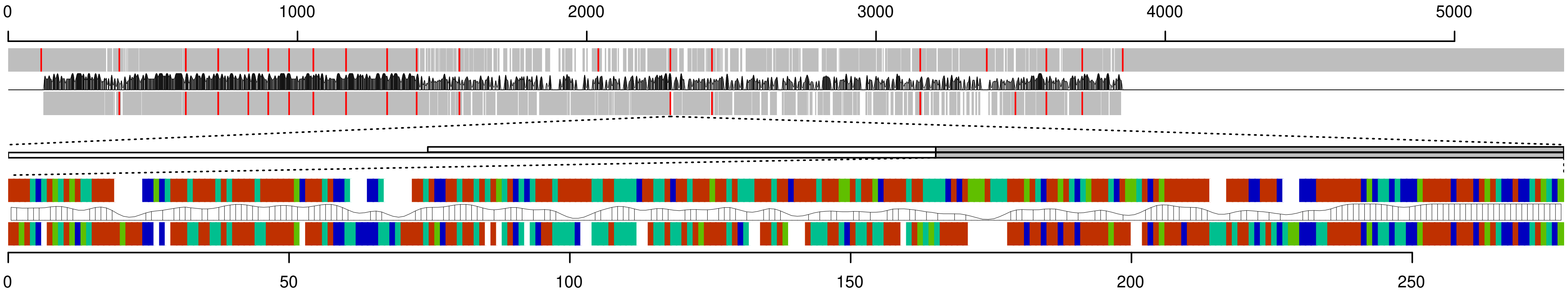

Danio rerio (ENSDART00000017185), Ochotona princeps (ENSOPRT00000013626)

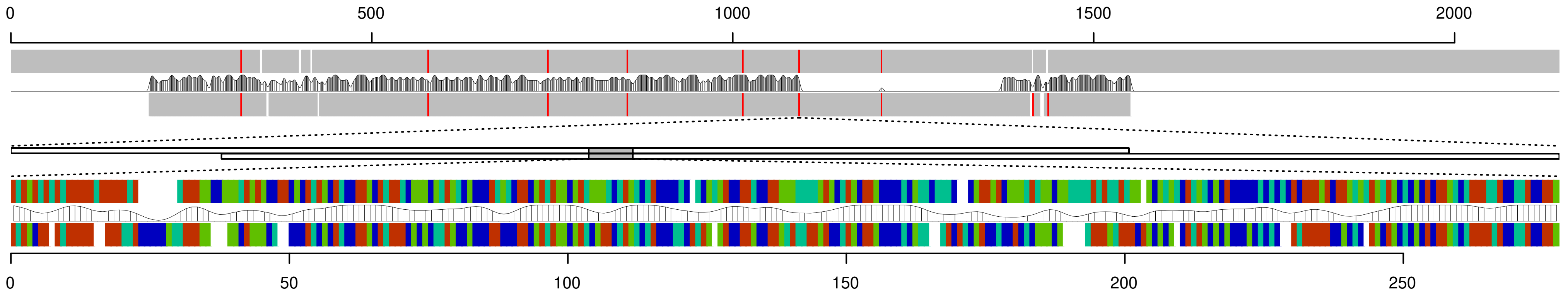

Danio rerio (ENSDART00000141595), Ailuropoda melanoleuca (ENSAMET00000011305)

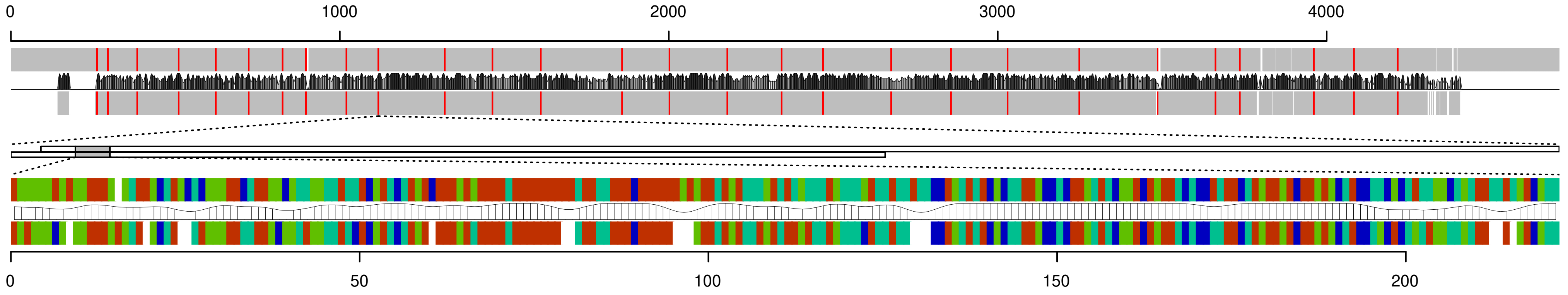

Danio rerio (ENSDART00000010378), Dipodomys ordii (ENSDORT00000012605)

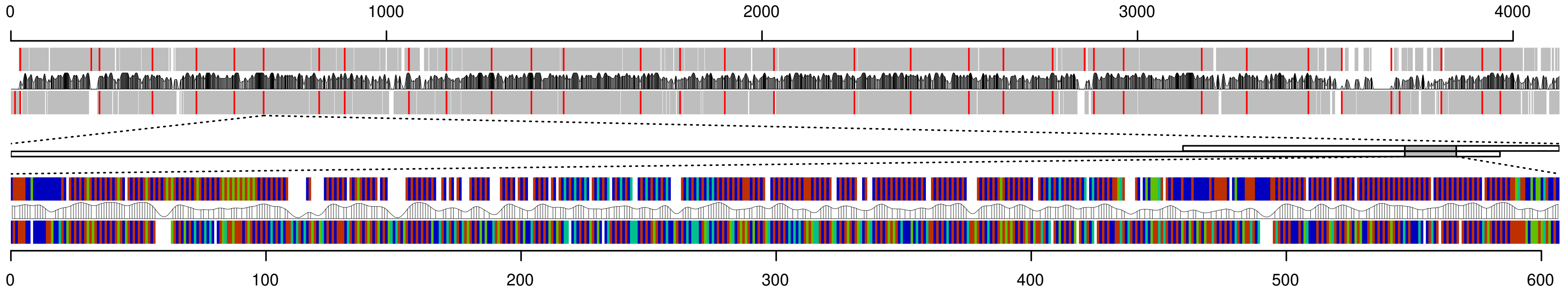

Danio rerio (ENSDART00000129303), Notamacropus eugenii (ENSMEUT00000002247)

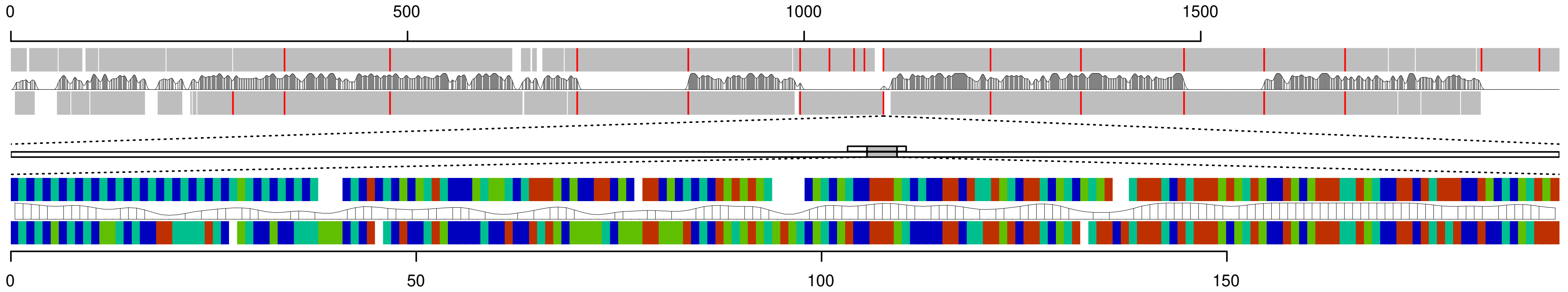

Danio rerio (ENSDART00000162942), Canis familiaris (ENSCAFT00000062120)

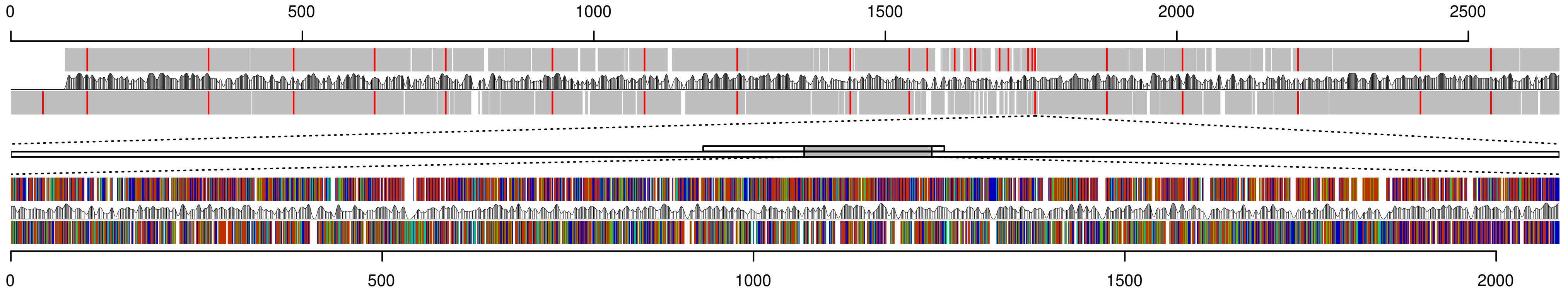

Danio rerio (ENSDART00000138167), Dasypus novemcinctus (ENSDNOT00000010663)

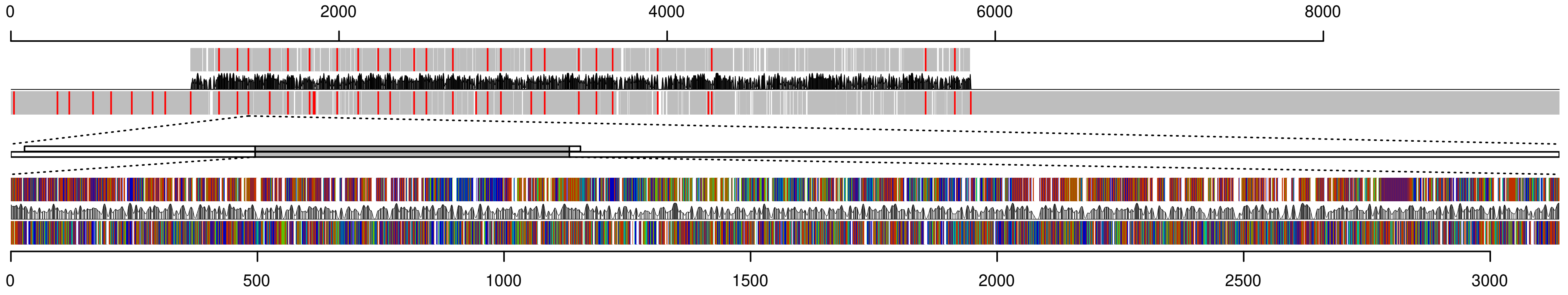

Danio rerio (ENSDART00000165609), Monodelphis domestica (ENSMODT00000015386)

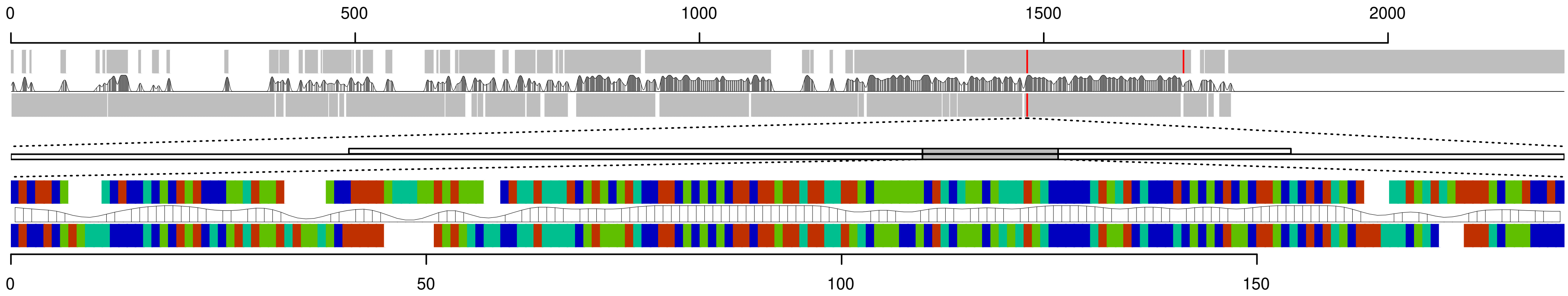

Danio rerio (ENSDART00000051278), Microtus ochrogaster (ENSMOCT00000011972)

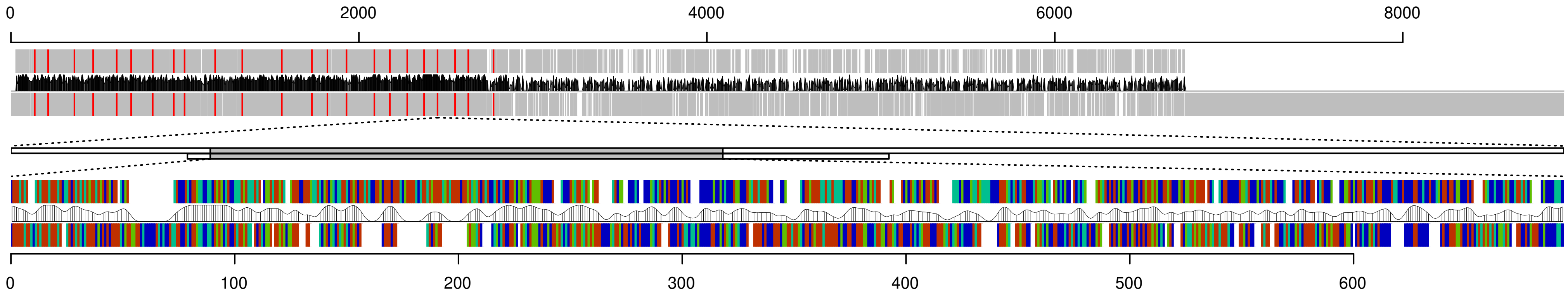

Danio rerio (ENSDART00000134661), Carlito syrichta (ENSTSYT00000007465)

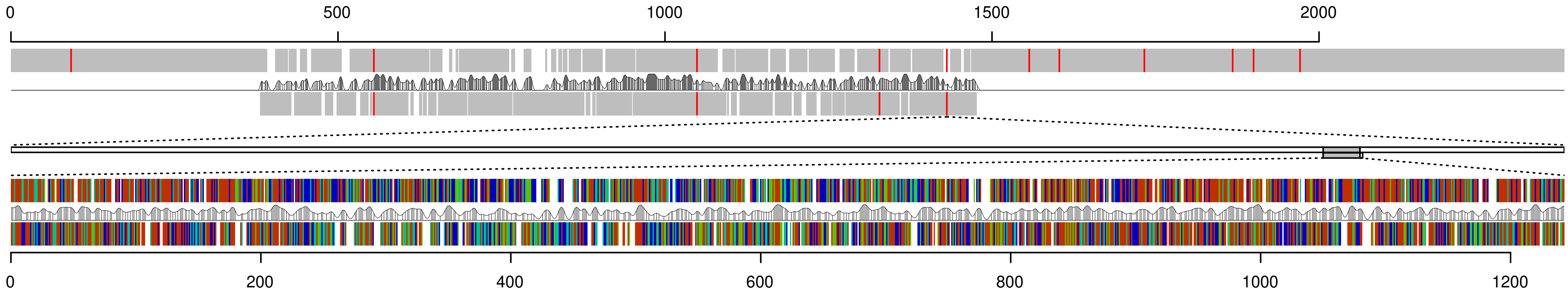

Danio rerio (ENSDART00000049226), Otolemur garnettii (ENSOGAT00000001943)

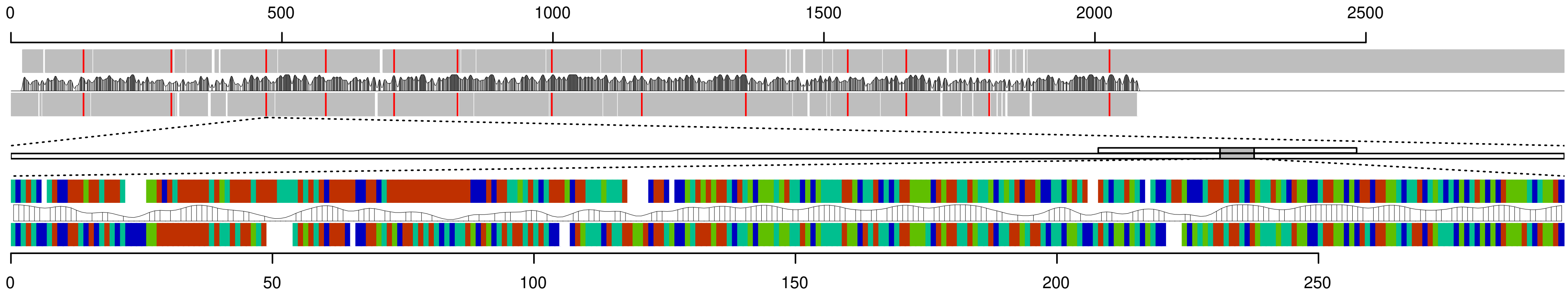

Danio rerio (ENSDART00000076596), Sarcophilus harrisii (ENSSHAT00000018877)

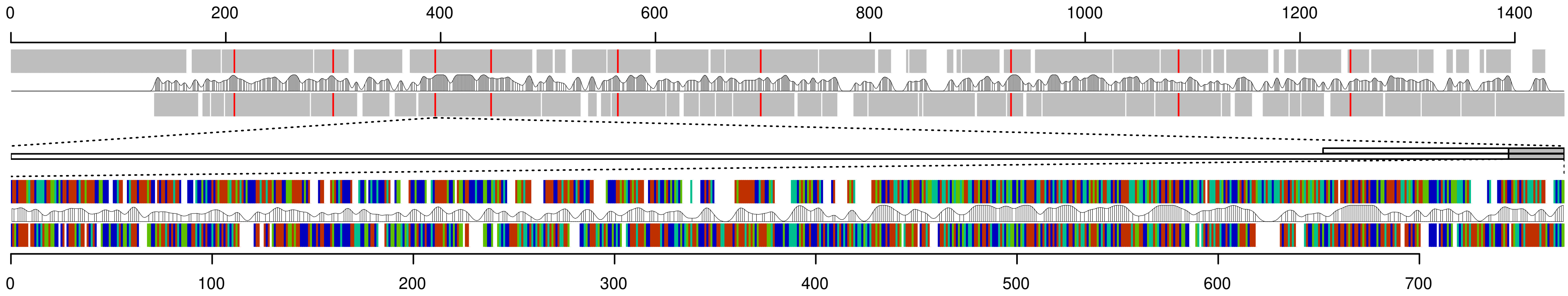

Danio rerio (ENSDART00000005562), Myotis lucifugus (ENSMLUT00000008788)

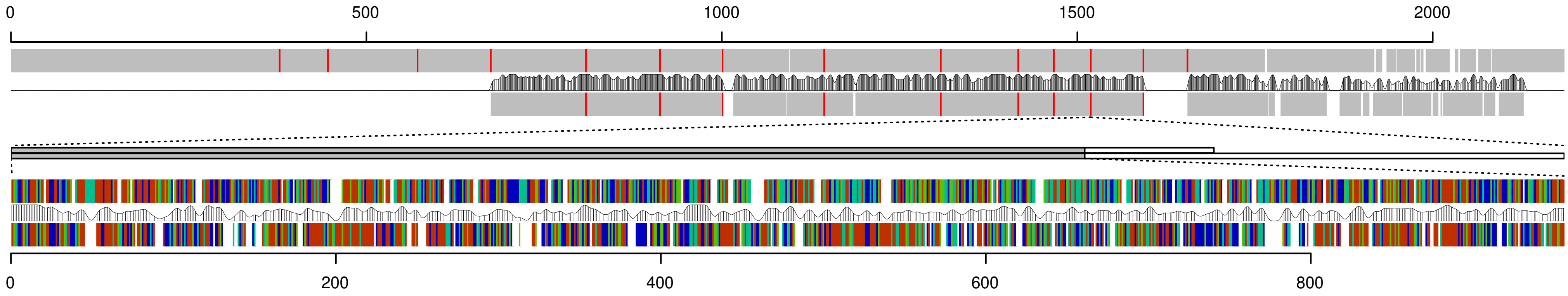

Danio rerio (ENSDART00000098791), Notamacropus eugenii (ENSMEUT00000008281)

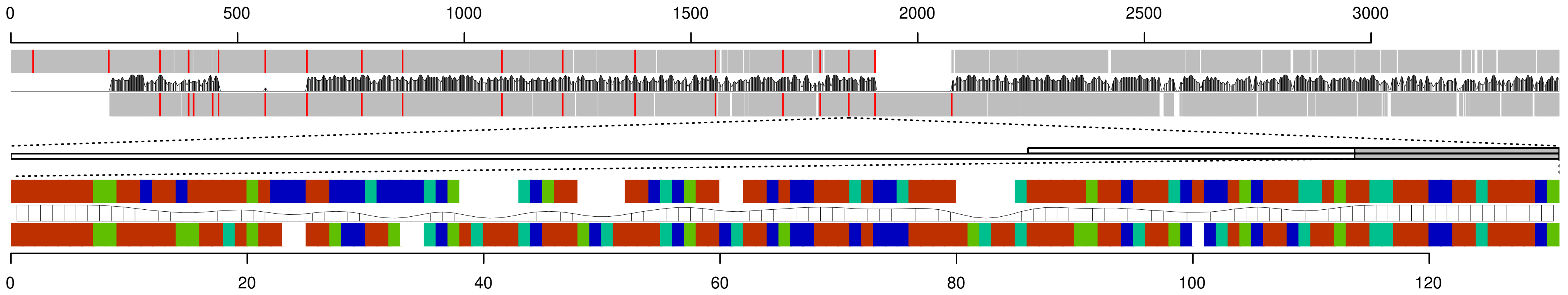

Danio rerio (ENSDART00000079341), Microcebus murinus (ENSMICT00000072925)

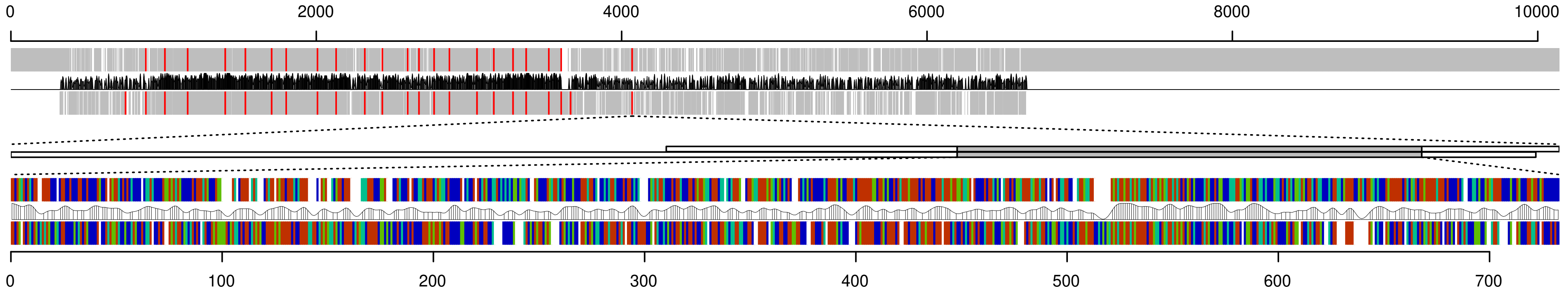

Danio rerio (ENSDART00000124660), Pteropus vampyrus (ENSPVAT00000012832)

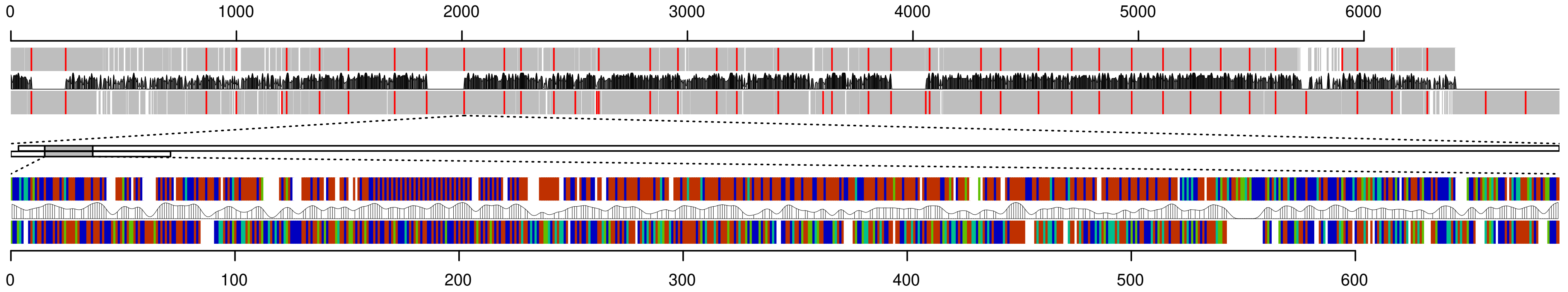

Danio rerio (ENSDART00000167052), Spermophilus dauricus (ENSSDAT00000004247)

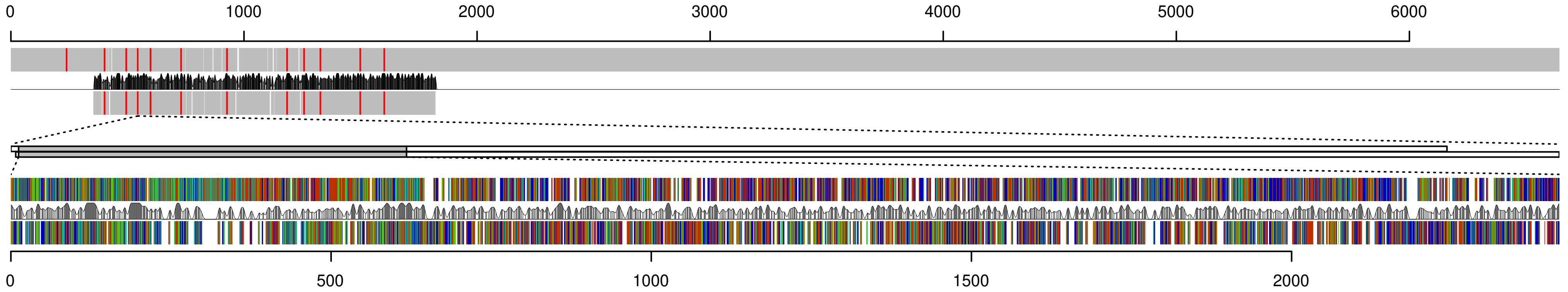

Danio rerio (ENSDART00000005562), Pan paniscus (ENSPPAT00000036662)

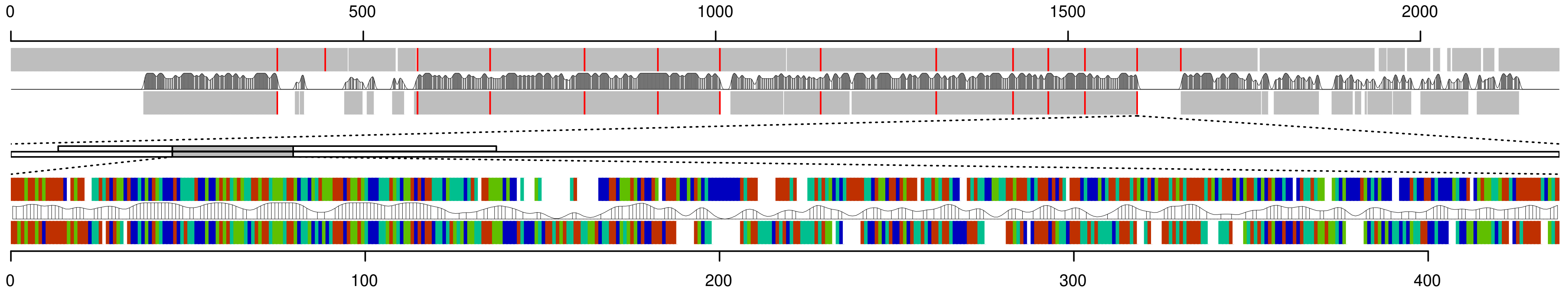

Danio rerio (ENSDART00000193891), Mus pahari (MGP_PahariEiJ_T0090177)

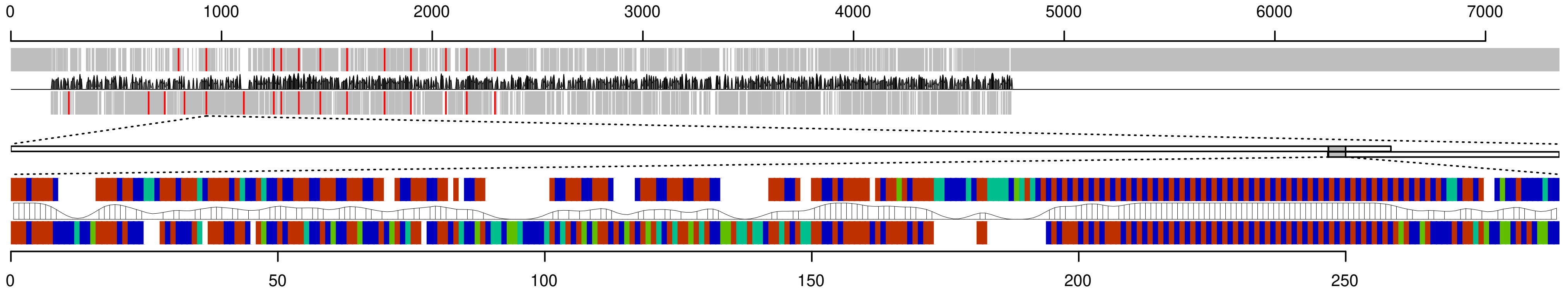

Danio rerio (ENSDART00000165680), Notamacropus eugenii (ENSMEUT00000004673)

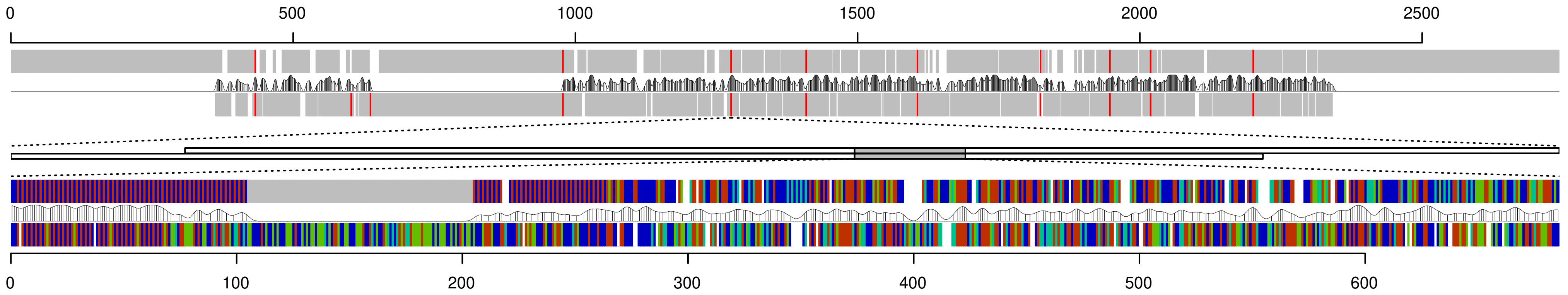

Danio rerio (ENSDART00000142818), Jaculus jaculus (ENSJJAT00000007974)

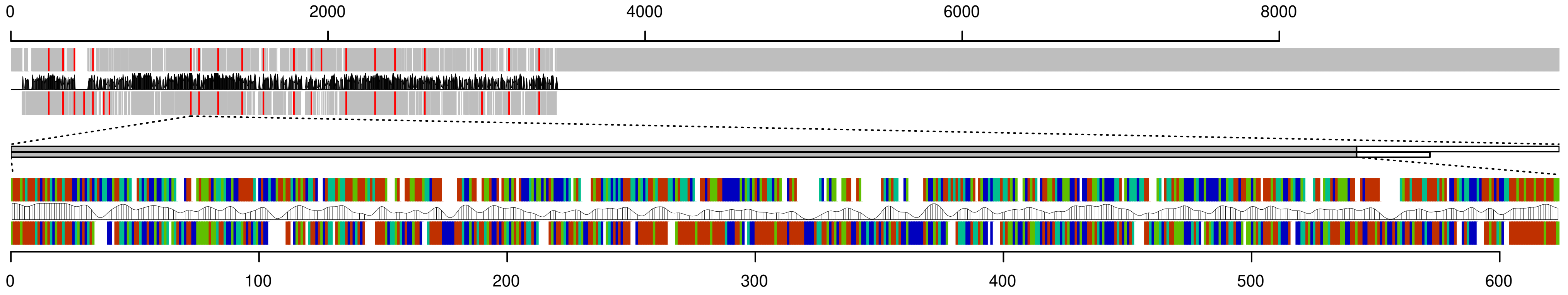

Danio rerio (ENSDART00000172320), Spermophilus dauricus (ENSSDAT00000028333)

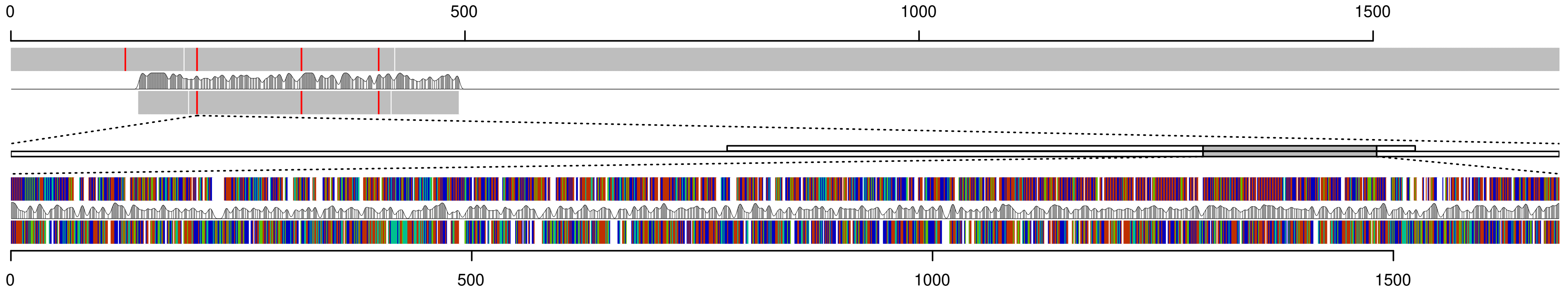

Danio rerio (ENSDART00000099027), Mus spicilegus (ENSMSIT00000021255)

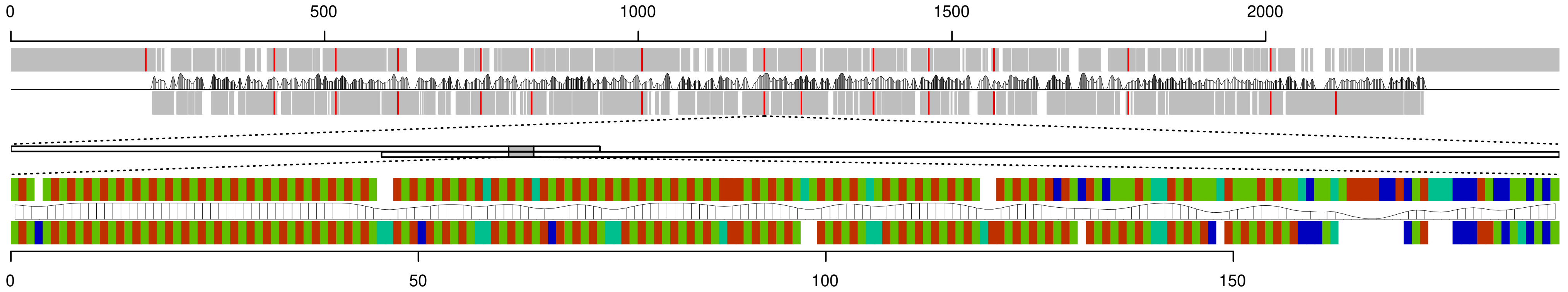

Danio rerio (ENSDART00000128415), Notamacropus eugenii (ENSMEUT00000011344)

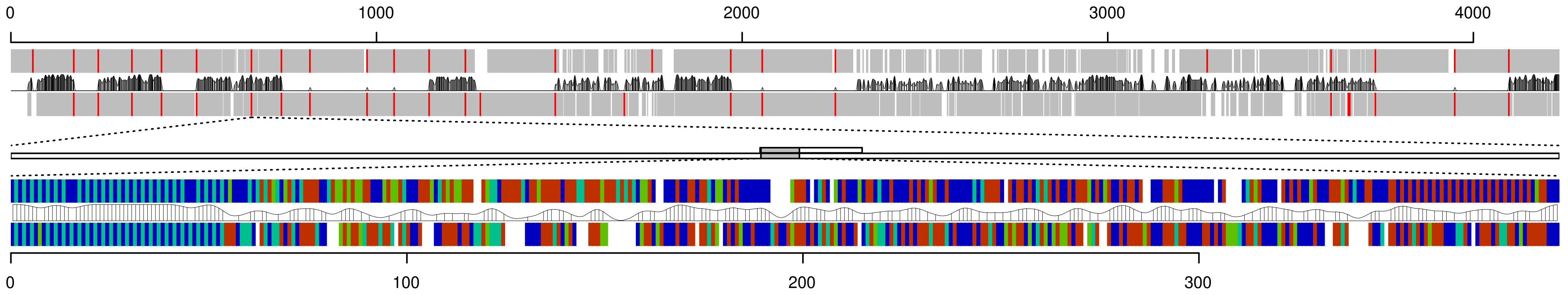

Danio rerio (ENSDART00000127923), Mus musculus (ENSMUST00000035112)

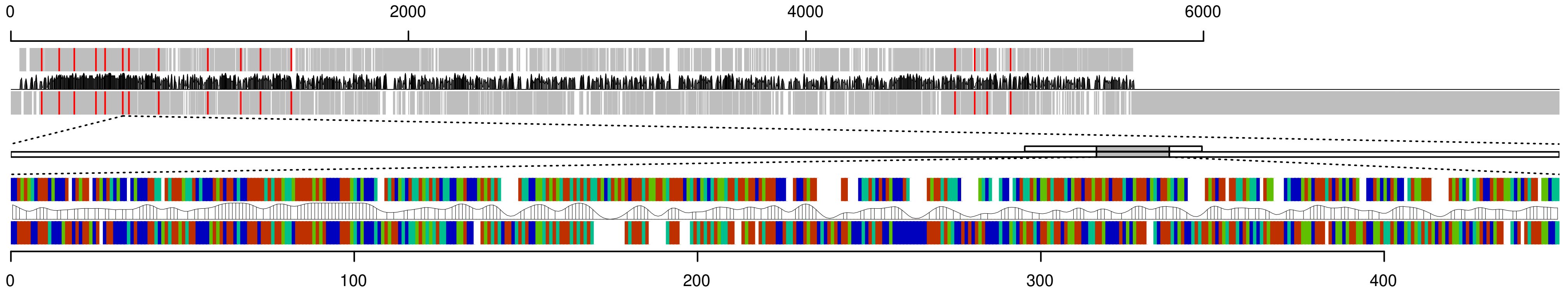

Danio rerio (ENSDART00000052656), Meriones unguiculatus (ENSMUGT00000022092)

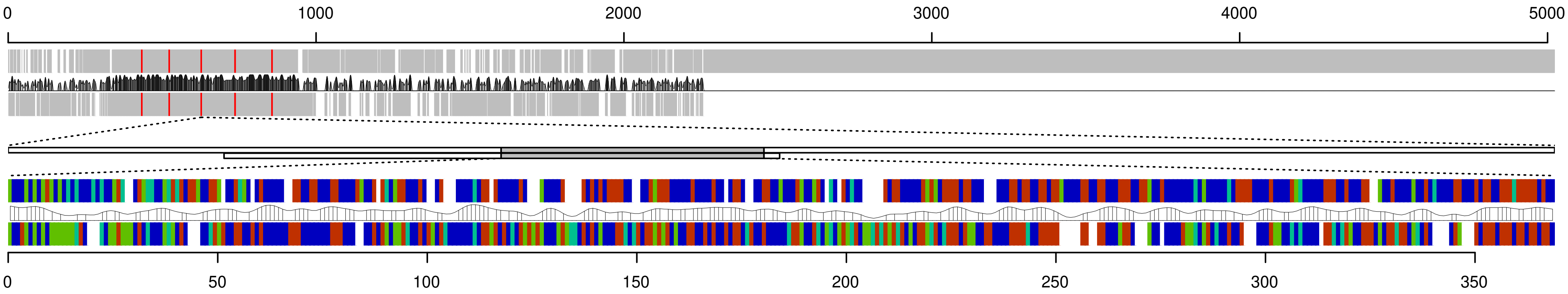

Danio rerio (ENSDART00000156751), Vulpes vulpes (ENSVVUT00000032749)

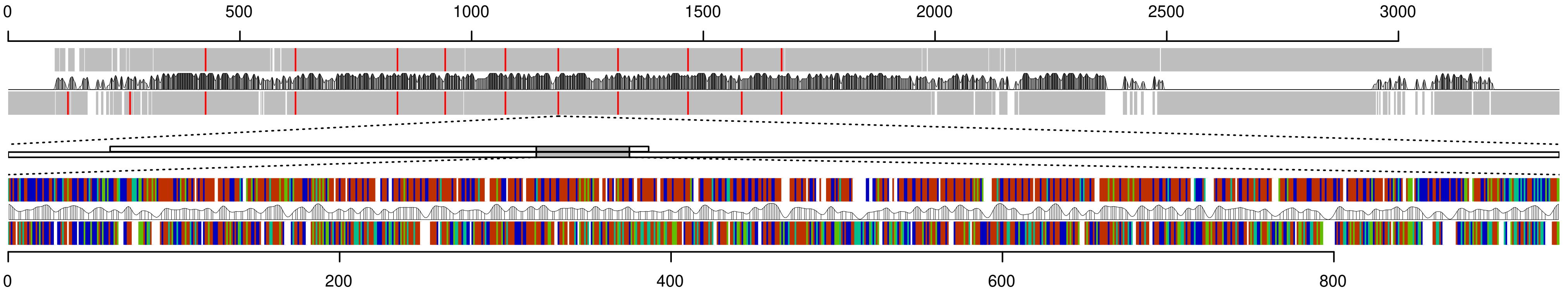

Danio rerio (ENSDART00000115394), Aotus nancymaae (ENSANAT00000057109)

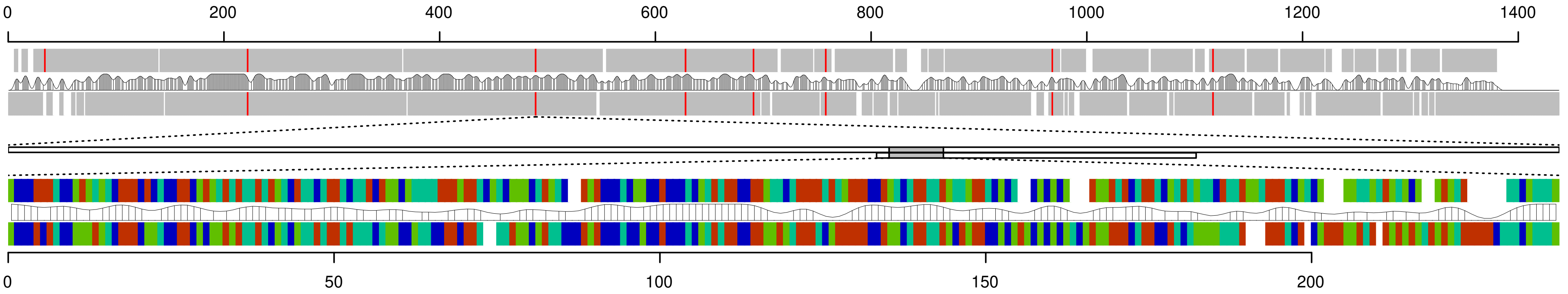

Danio rerio (ENSDART00000171324), Procavia capensis (ENSPCAT00000015877)

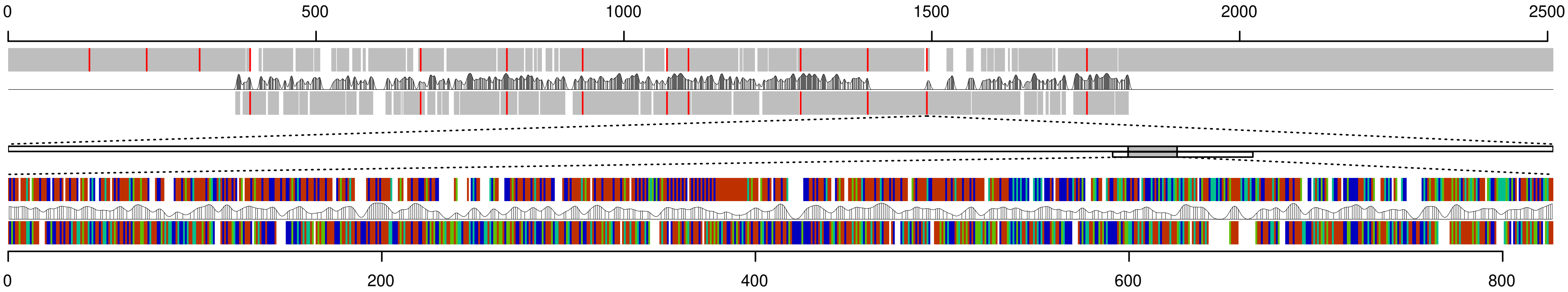

Danio rerio (ENSDART00000138204), Dasypus novemcinctus (ENSDNOT00000051824)

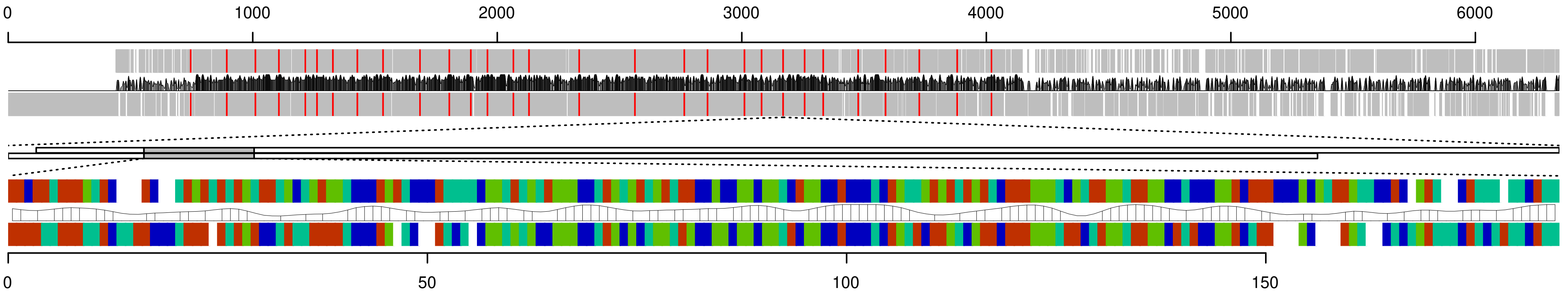

Danio rerio (ENSDART00000172520), Microcebus murinus (ENSMICT00000041744)

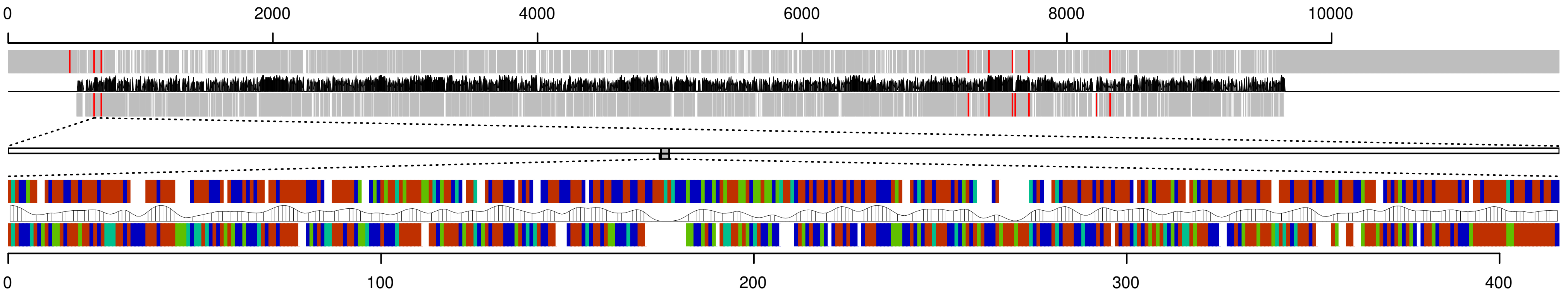

Danio rerio (ENSDART00000162777), Vombatus ursinus (ENSVURT00010024353)

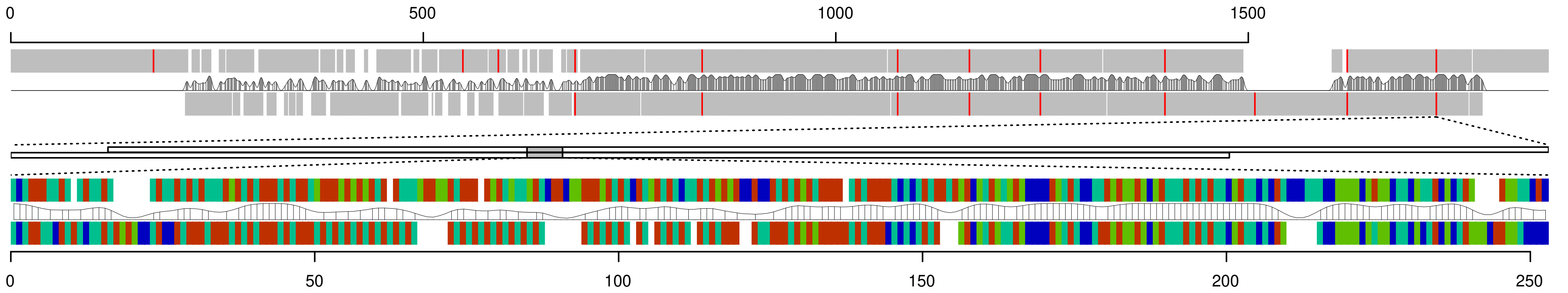

Danio rerio (ENSDART00000012194), Notamacropus eugenii (ENSMEUT00000010477)

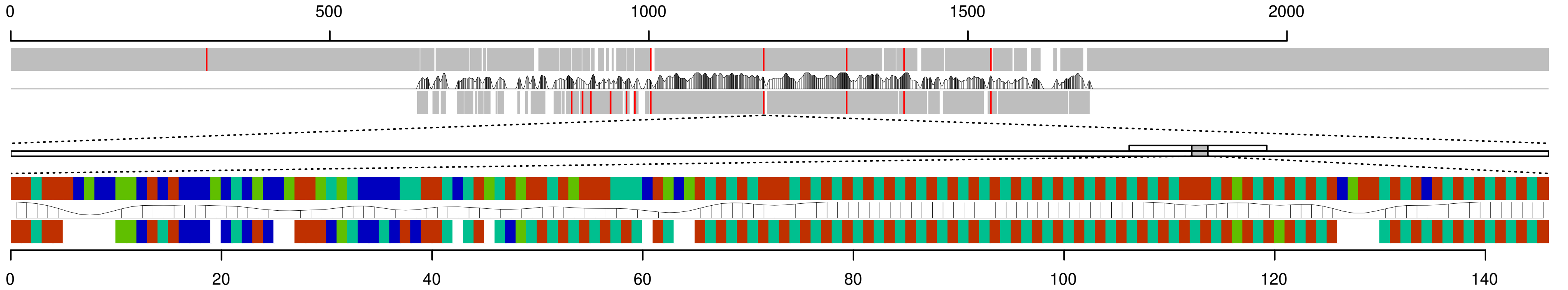

Danio rerio (ENSDART00000031895), Rattus norvegicus (ENSRNOT00000086849)

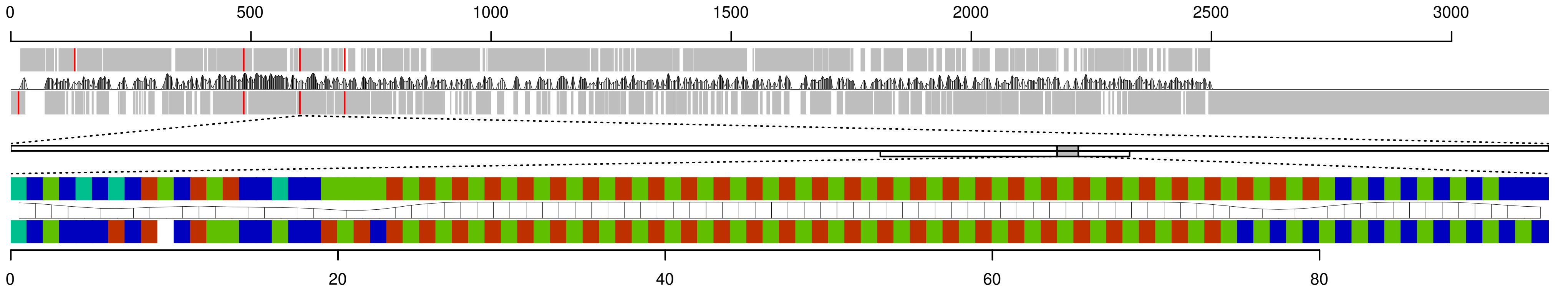

Danio rerio (ENSDART00000042134), Macaca fascicularis (ENSMFAT00000070691)

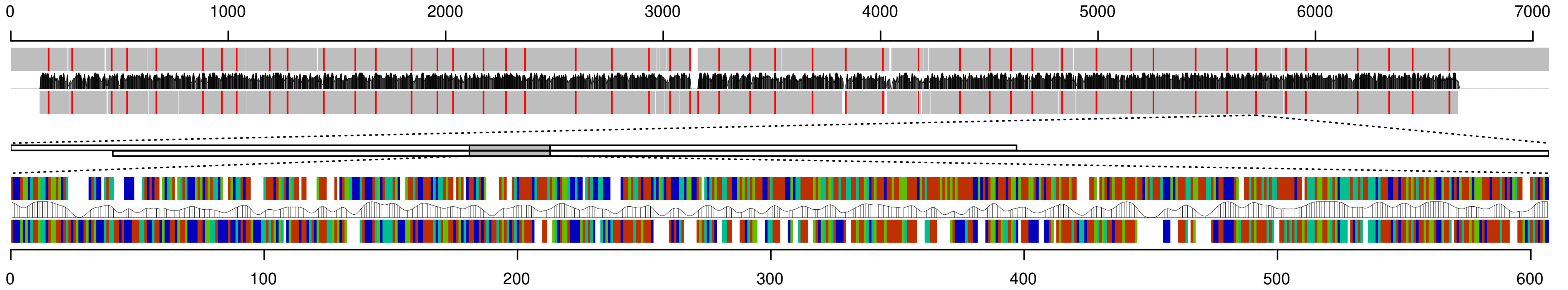

Danio rerio (ENSDART00000138964), Mus musculus (ENSMUST00000167391)

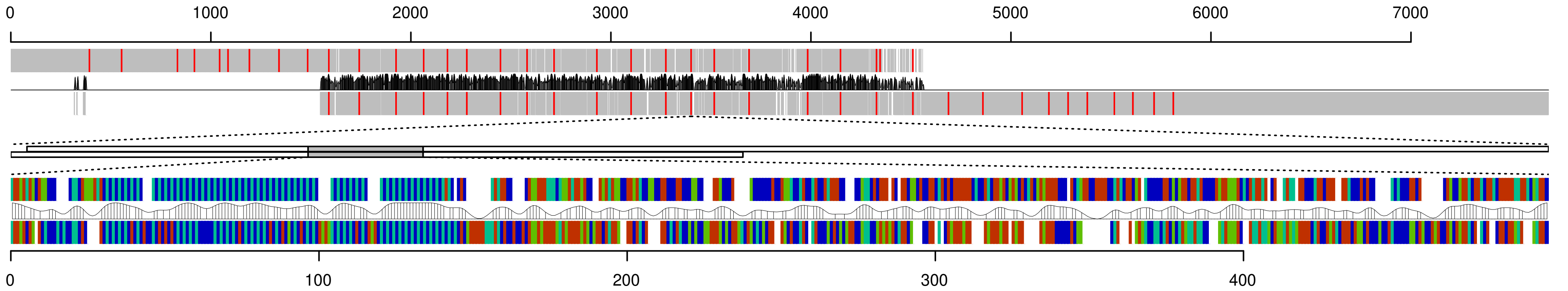

Danio rerio (ENSDART00000135864), Monodelphis domestica (ENSMODT00000080178)

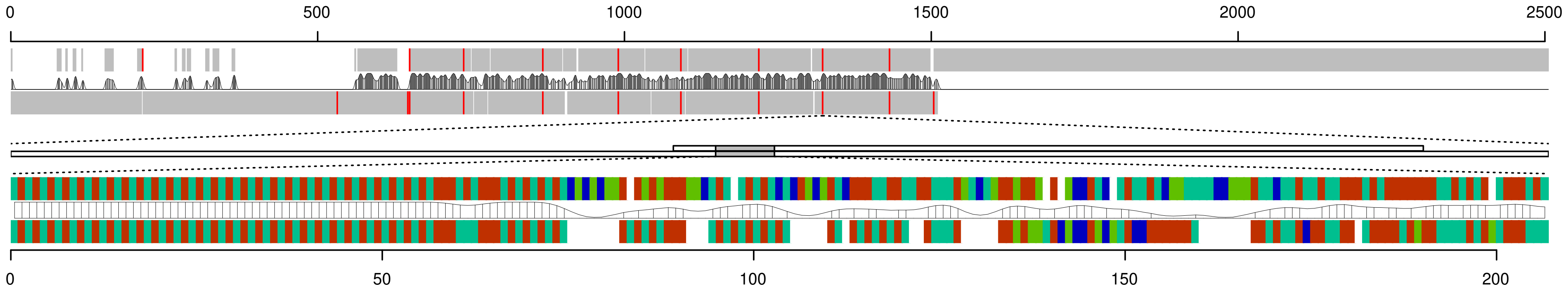

Danio rerio (ENSDART00000051893), Sus scrofa (ENSSSCT00000042436)

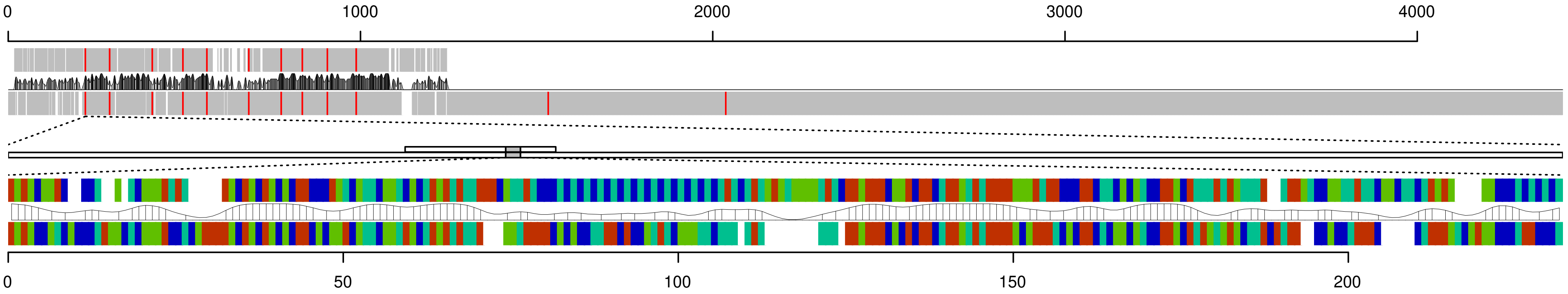

Danio rerio (ENSDART00000106186), Sus scrofa (ENSSSCT00000053248)

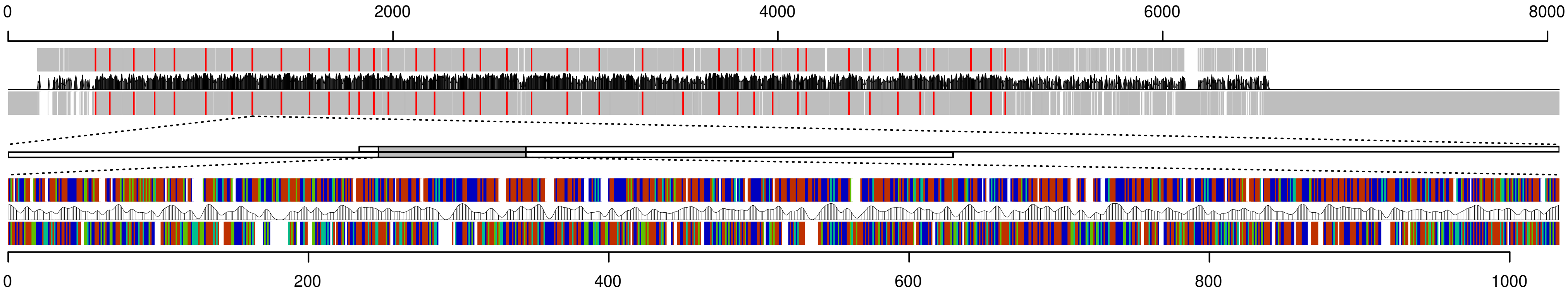

Danio rerio (ENSDART00000146084), Choloepus hoffmanni (ENSCHOT00000002876)

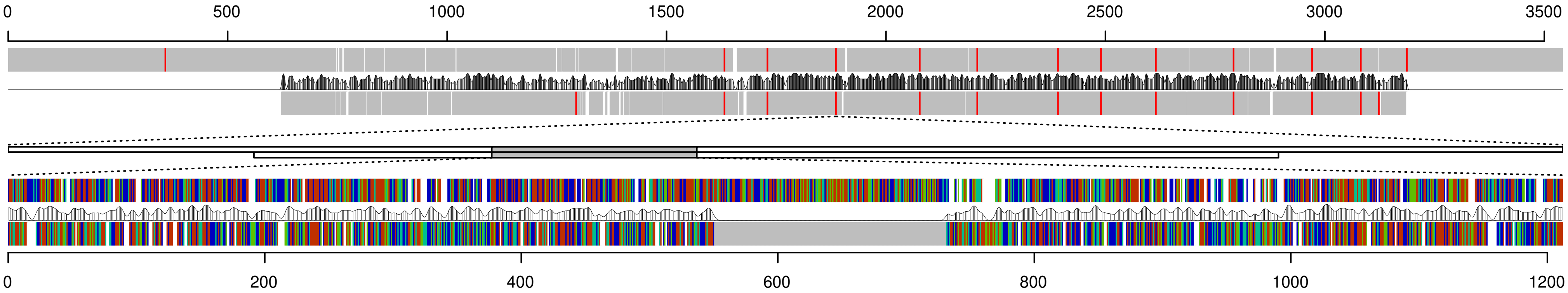

Danio rerio (ENSDART00000160582), Mus musculus (ENSMUST00000203528)

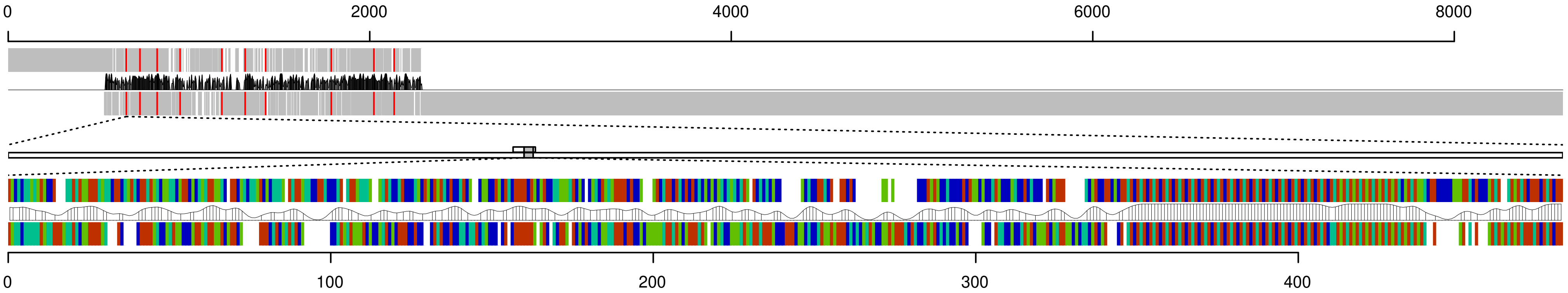

Danio rerio (ENSDART00000155020), Vombatus ursinus (ENSVURT00010019818)

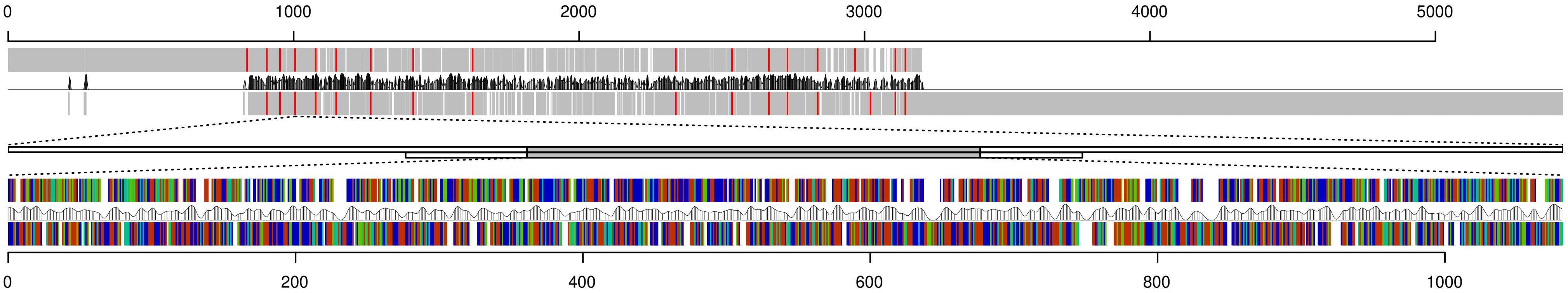

Danio rerio (ENSDART00000146787), Oryctolagus cuniculus (ENSOCUT00000012485)

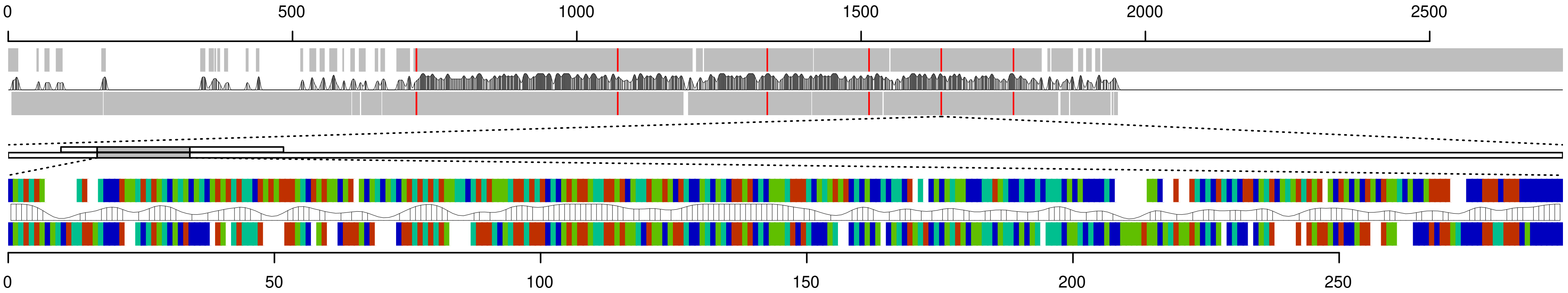

Supplement: Supplementary file 6 — Transcript and intron alignments for points in Fig. S14. Each panel shows the maximally scoring alignment between D. rerio and teleost intron orthologues (lower) and transcript alignment (upper) used to establish the intron orthology. Grey, white and red parts indicate aligned exonic sequence, gaps and positions of intron meta-characters respectively. Colours in intron alignment represent bases (A blue, C cyan, G green, T brown, N grey, gap white). Curves lying between sequence representations show a normal kernel density smoothed estimate of local similarity (9 bp window, standard deviation two); vertical lines indicate matches. Region between exon and intron alignments indicates the location of the maximally scoring alignment in the introns. Upper sequence D. rerio. Files 6–10 and 11–15 contain alignments to teleost and mammalian sequences respectively. Each file corresponds to one panel in Fig. S17 and to one specific teleost size class: Files 6,11: long (E,J), 7,12: medium (D,I), 8,13: short.2 (C,H), 9,14 short (B, G) and 10,15 ctl (A,F). [file 12864_2022_8760_MOESM6_ESM.zip › 12864_2022_8760_MOESM12_ESM.pdf]
