# Supplementary material for: Intron size minimisation in teleosts
Source: BMC Genomics. 2022 Sep 1;23:628. doi: 10.1186/s12864-022-08760-w (PMC9438311; doi:10.1186/s12864-022-08760-w)

Danio rerio (ENSDART00000115296), Pygocentrus nattereri (ENSPNAT00000019561)

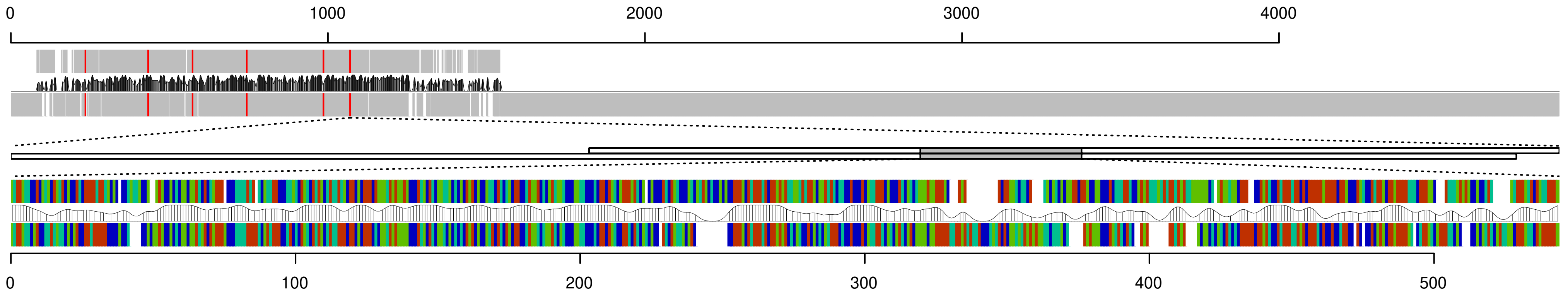

Danio rerio (ENSDART00000064412), Astyanax mexicanus (ENSAMXT00000000419)

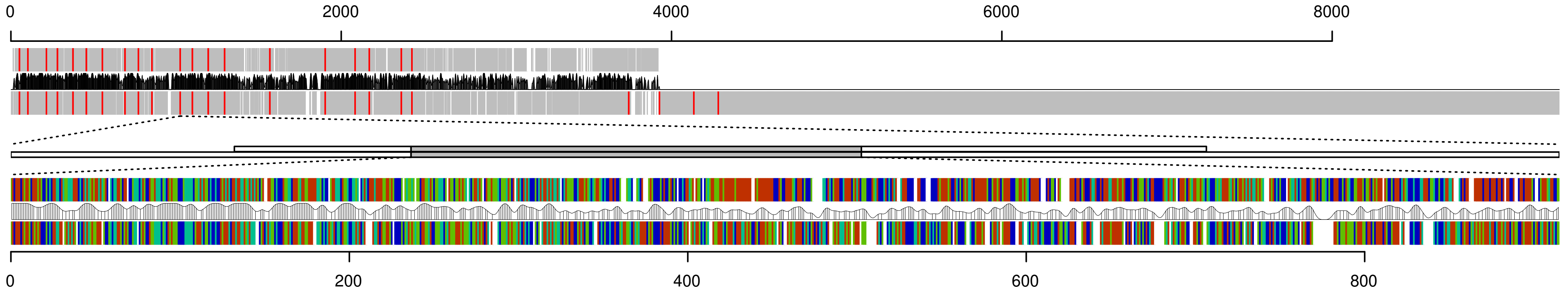

Danio rerio (ENSDART00000148314), Pygocentrus nattereri (ENSPNAT00000024463)

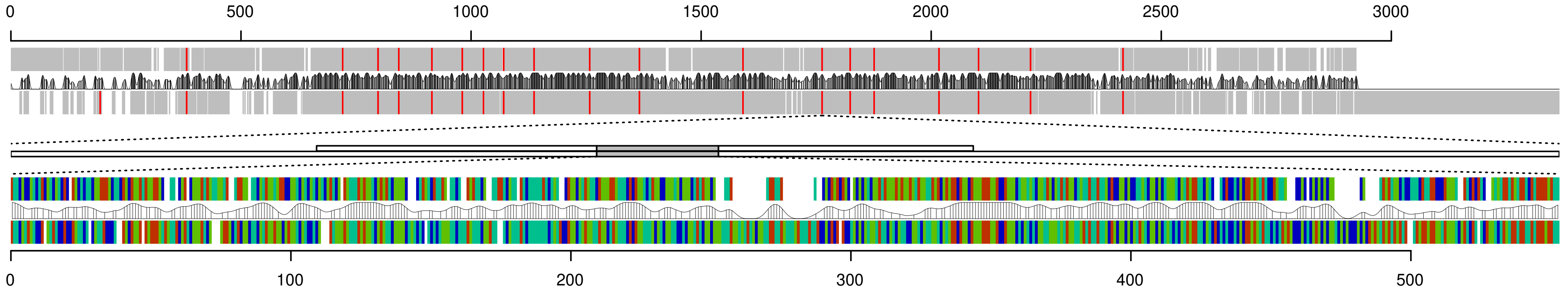

Danio rerio (ENSDART00000149744), Denticeps clupeoides (ENSDCDT00000075728)

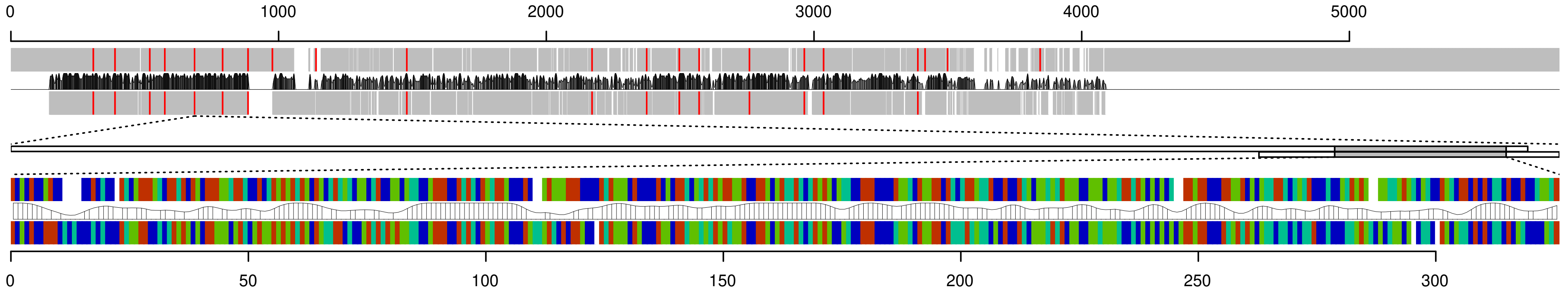

Danio rerio (ENSDART00000014536), Ictalurus punctatus (ENSIPUT00000005184)

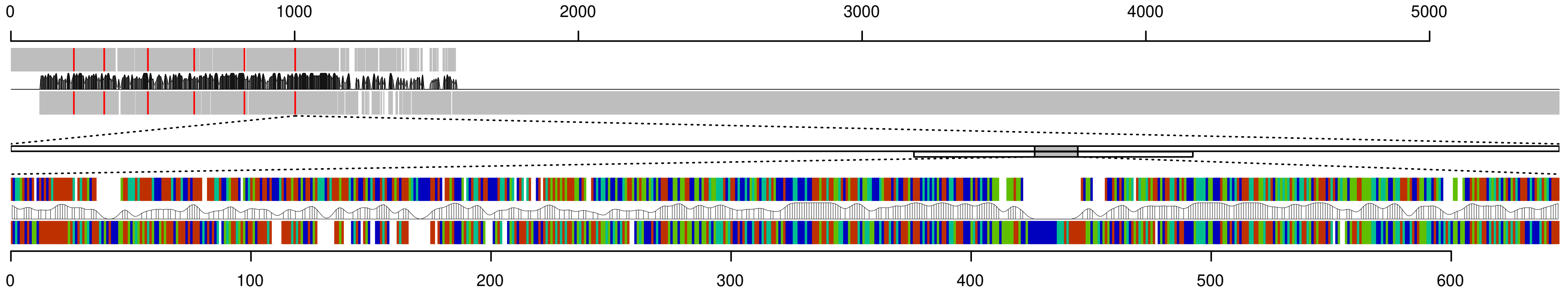

Danio rerio (ENSDART00000134735), Pygocentrus nattereri (ENSPNAT00000020552)

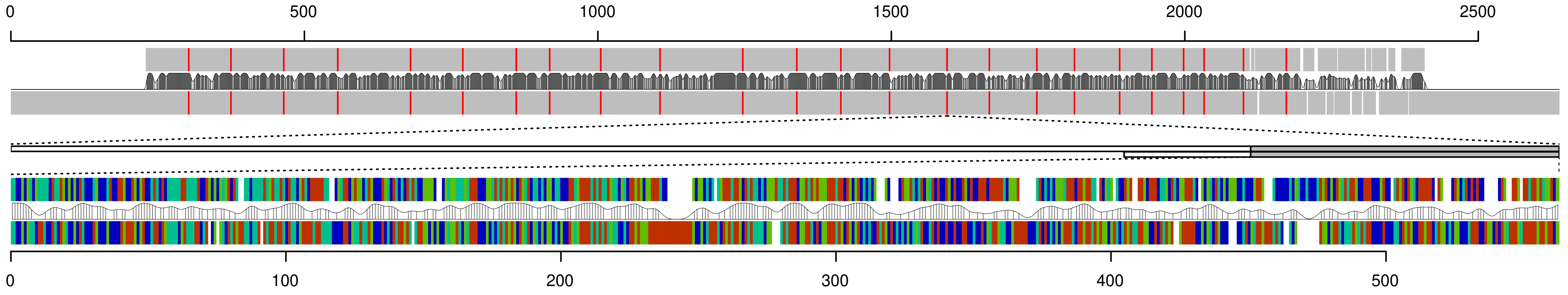

Danio rerio (ENSDART00000171496), Electrophorus electricus (ENSEEET00000026309)

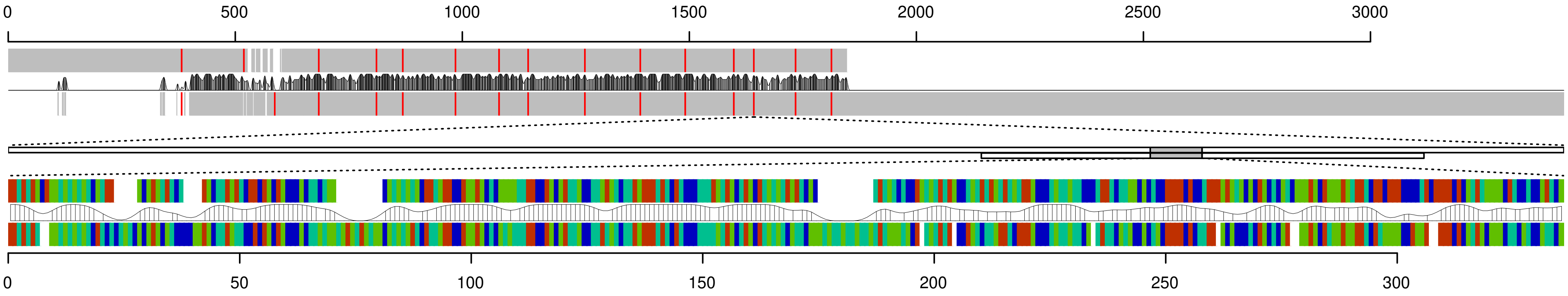

Danio rerio (ENSDART00000109978), Amphilophus citrinellus (ENSACIT00000028942)

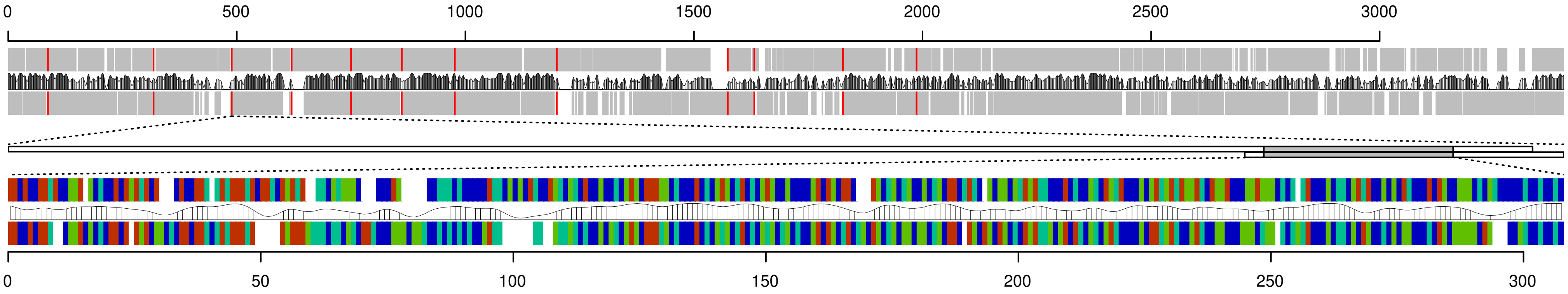

Danio rerio (ENSDART00000042895), Gadus morhua (ENSGMOT00000016177)

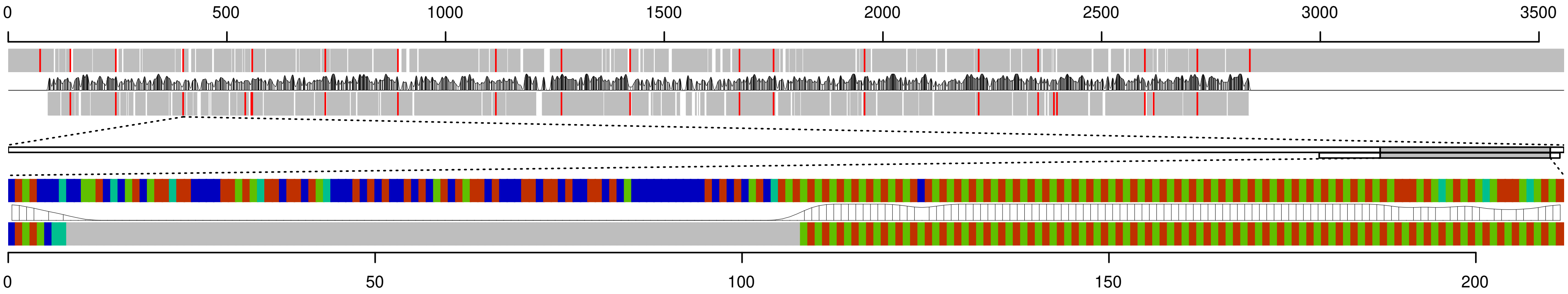

Danio rerio (ENSDART00000173293), Pygocentrus nattereri (ENSPNAT00000003328)

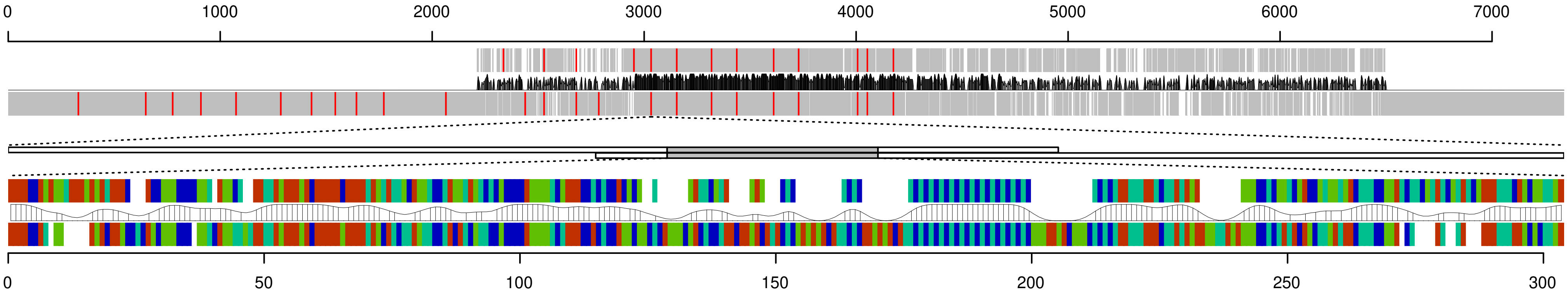

Danio rerio (ENSDART00000020969), Ictalurus punctatus (ENSIPUT00000002970)

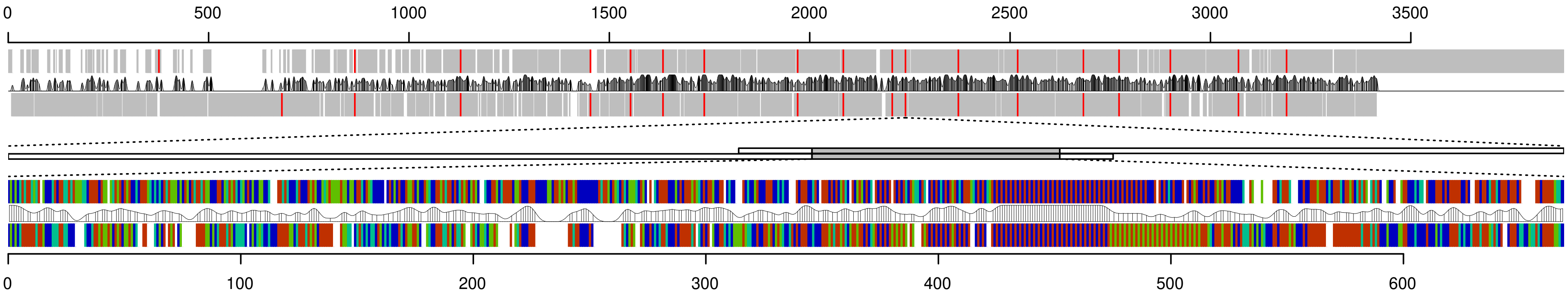

Danio rerio (ENSDART00000102552), Pygocentrus nattereri (ENSPNAT00000005462)

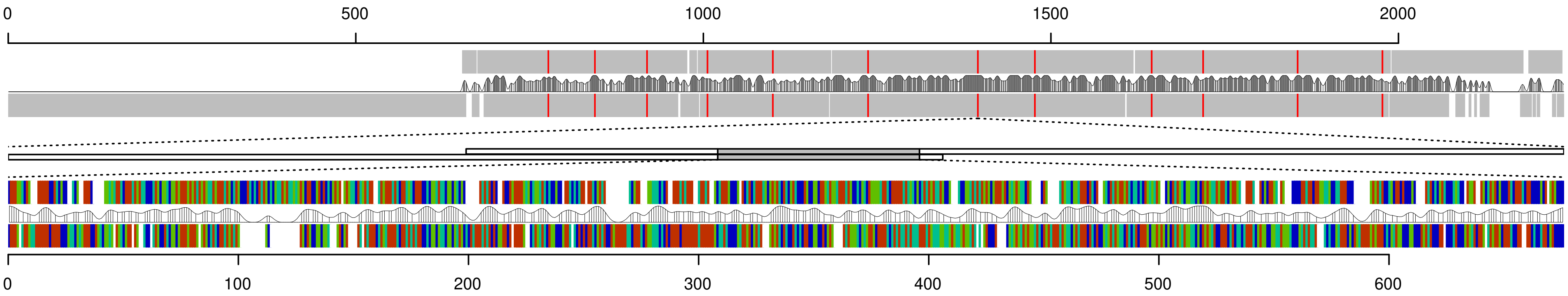

Danio rerio (ENSDART00000086202), Pygocentrus nattereri (ENSPNAT00000012824)

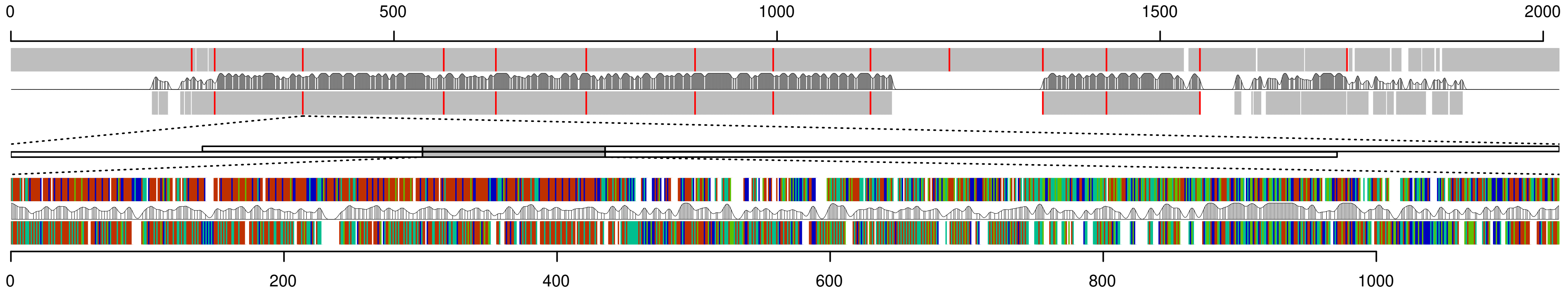

Danio rerio (ENSDART00000048095), Cottoperca gobio (ENSCGOT00000043946)

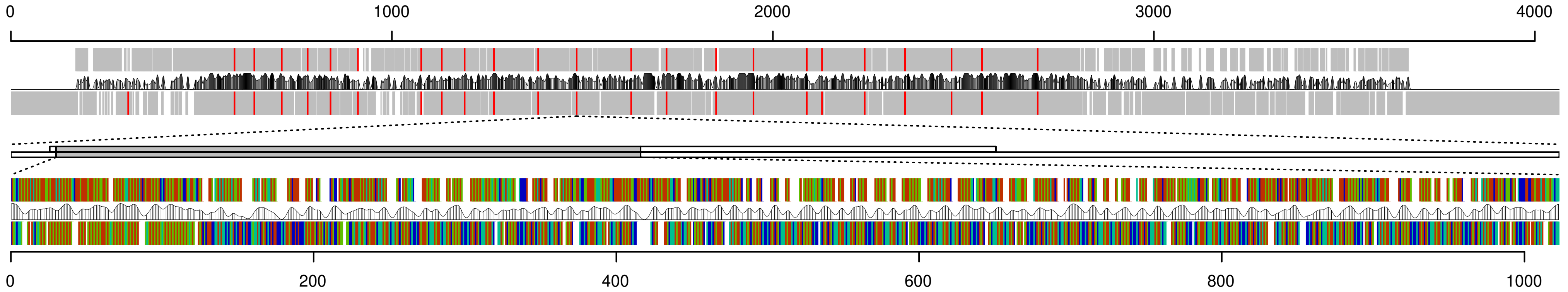

Danio rerio (ENSDART00000026903), Hucho hucho (ENSHHUT00000043576)

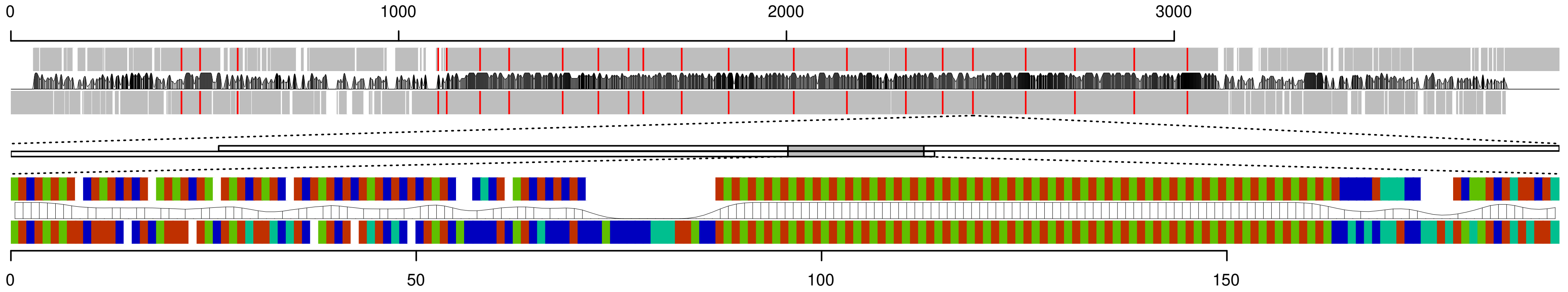

Danio rerio (ENSDART00000005119), Pygocentrus nattereri (ENSPNAT00000041206)

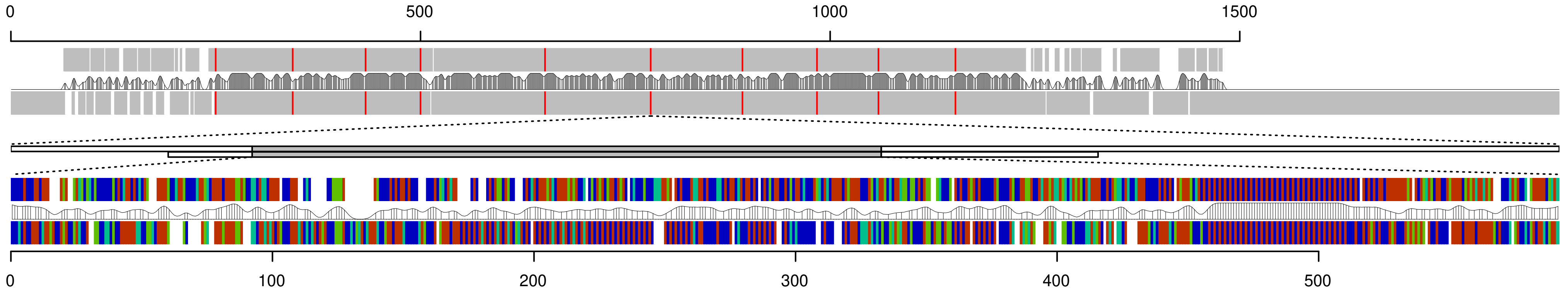

Danio rerio (ENSDART00000003789), Gadus morhua (ENSGMOT00000018397)

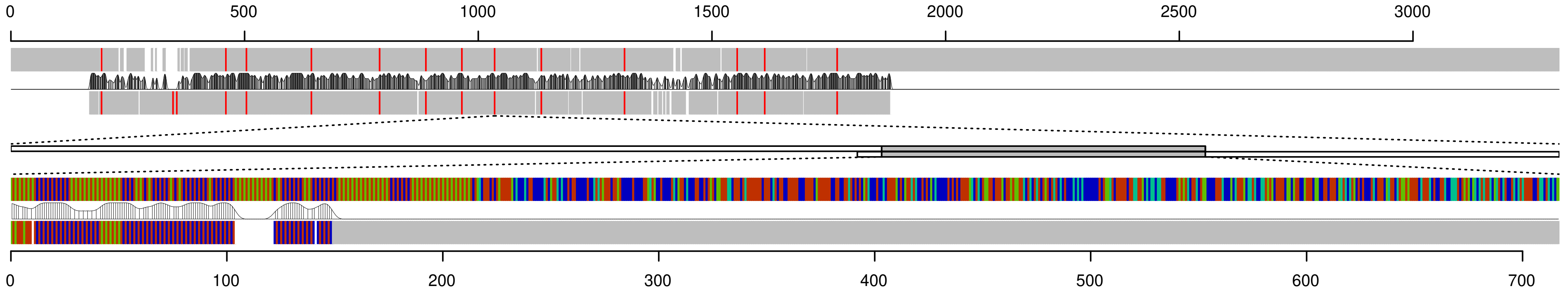

Danio rerio (ENSDART00000149744), Astyanax mexicanus (ENSAMXT00000021901)

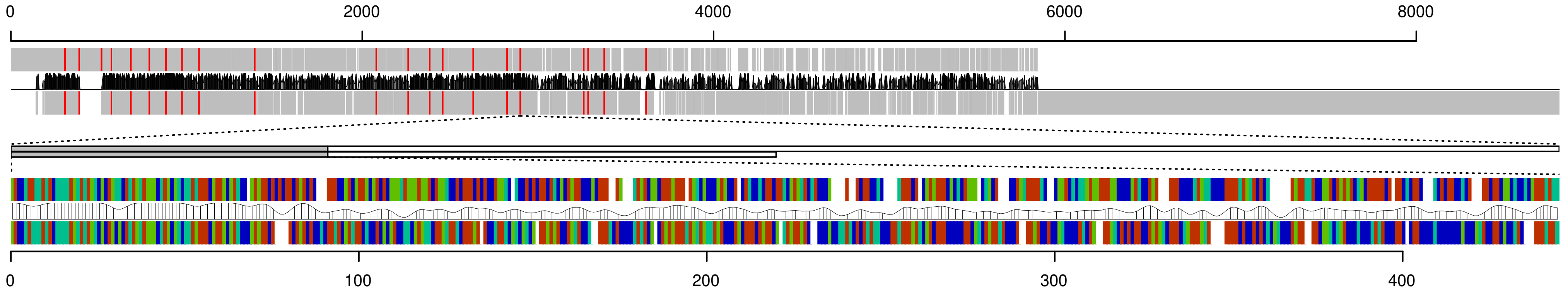

Danio rerio (ENSDART00000193590), Astyanax mexicanus (ENSAMXT00000019810)

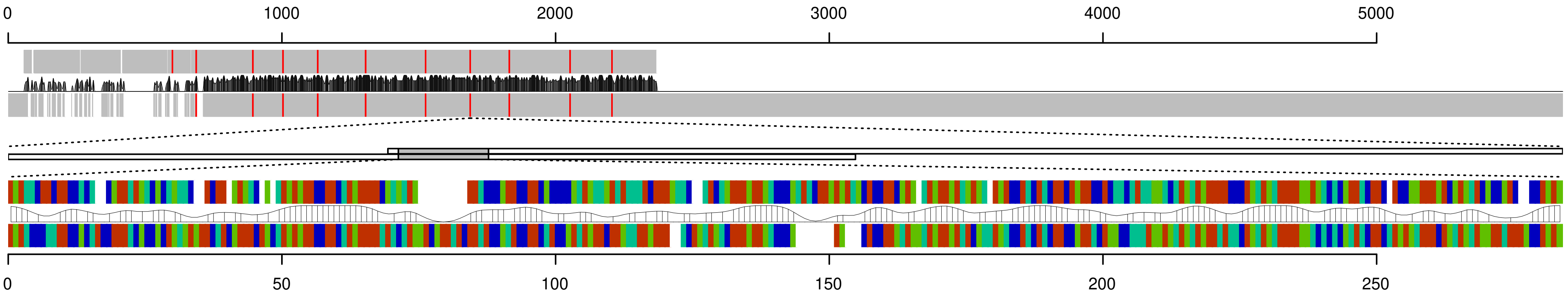

Danio rerio (ENSDART00000157937), Pygocentrus nattereri (ENSPNAT00000020995)

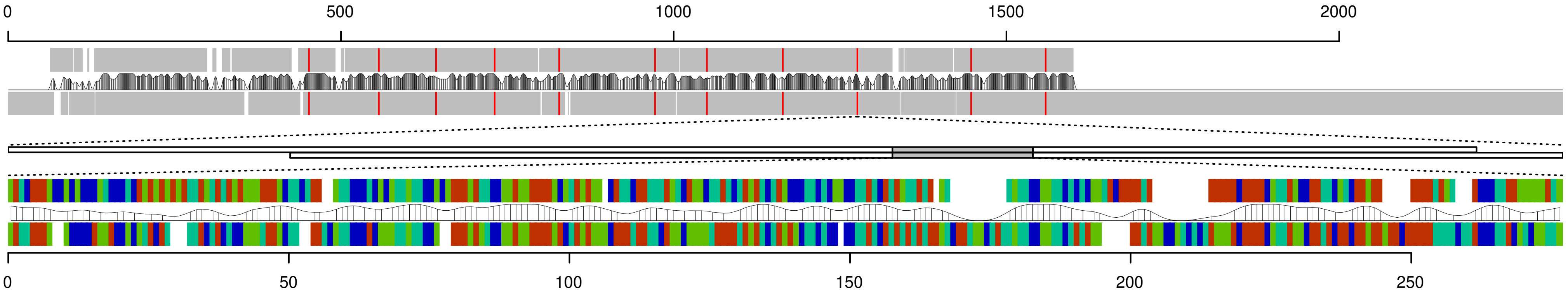

Danio rerio (ENSDART00000160285), Astyanax mexicanus (ENSAMXT00000018760)

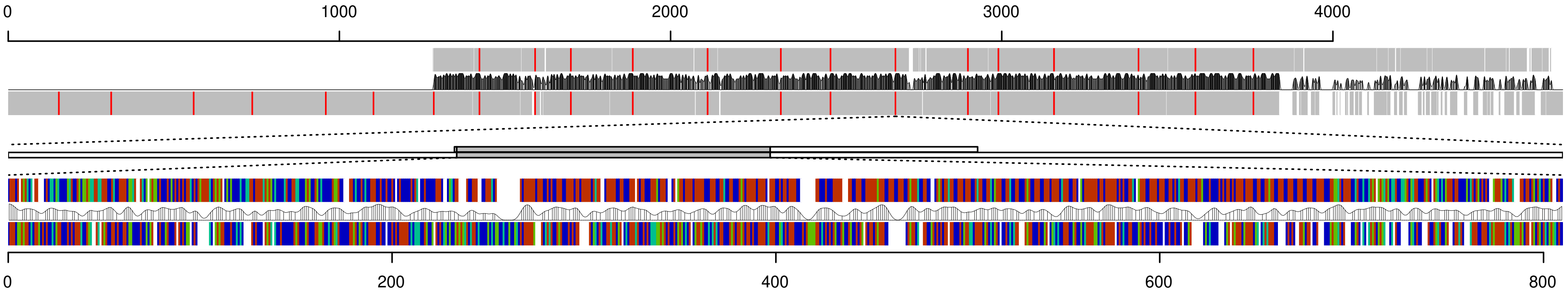

Danio rerio (ENSDART00000036419), Astyanax mexicanus (ENSAMXT00000004678)

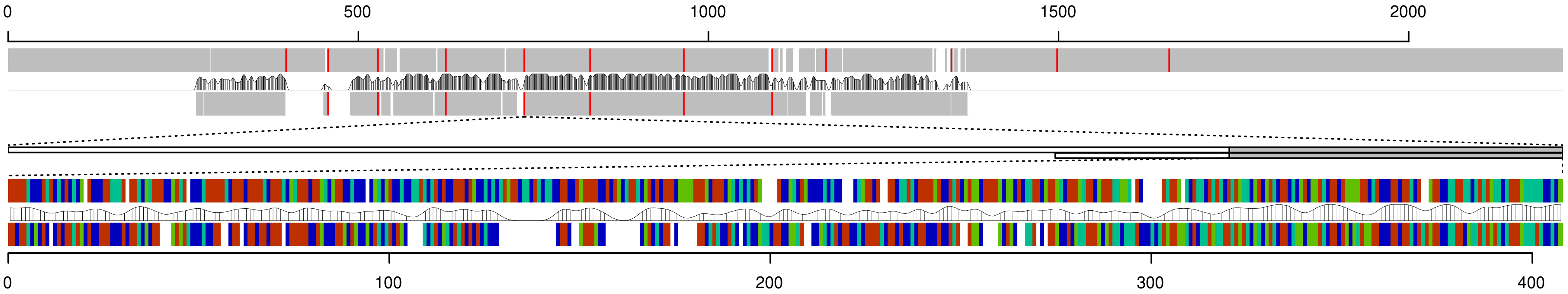

Danio rerio (ENSDART00000155721), Astyanax mexicanus (ENSAMXT00000018378)

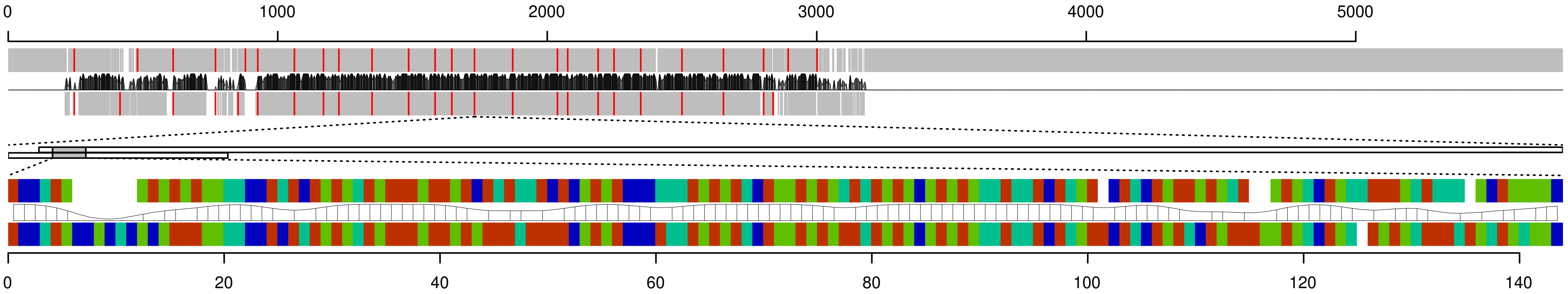

Danio rerio (ENSDART00000122458), Pygocentrus nattereri (ENSPNAT00000039877)

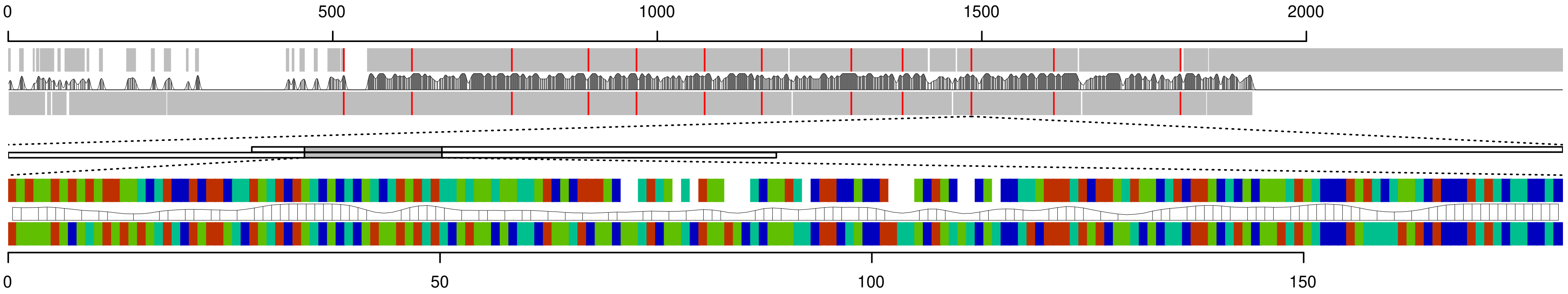

Danio rerio (ENSDART00000168530), Clupea harengus (ENSCHAT00000015543)

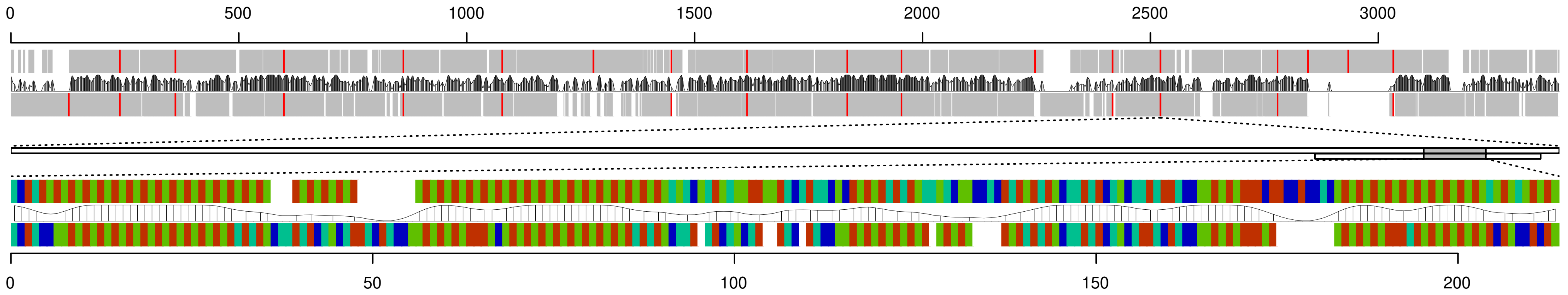

Danio rerio (ENSDART00000074777), Astyanax mexicanus (ENSAMXT00000010570)

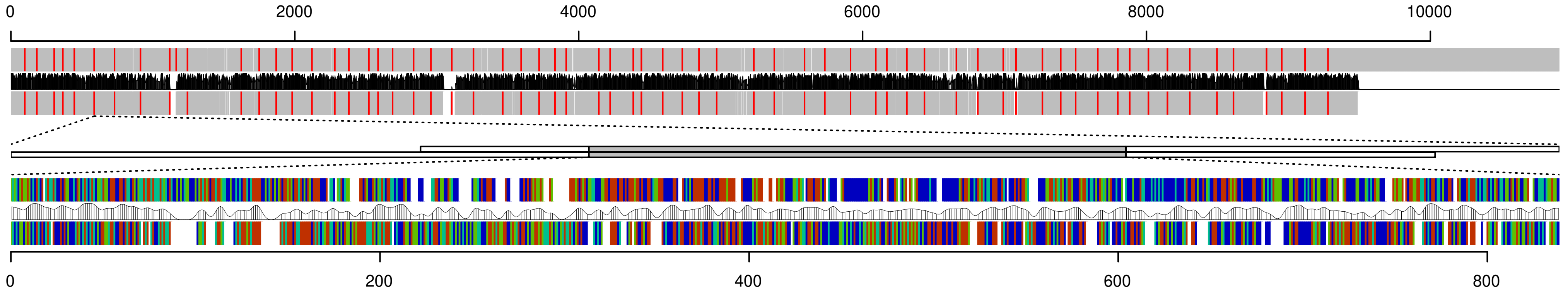

Danio rerio (ENSDART00000077887), Scleropages formosus (ENSSFOT00015034695)

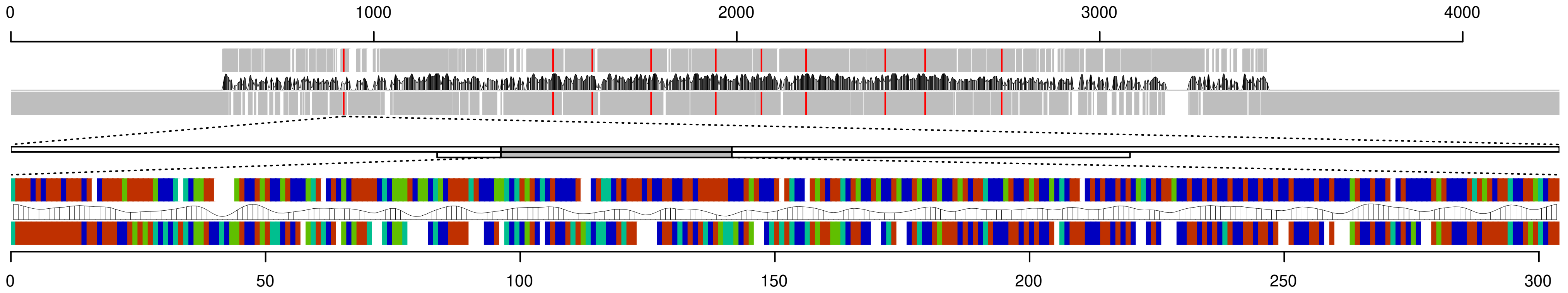

Danio rerio (ENSDART00000016105), Astyanax mexicanus (ENSAMXT00000019093)

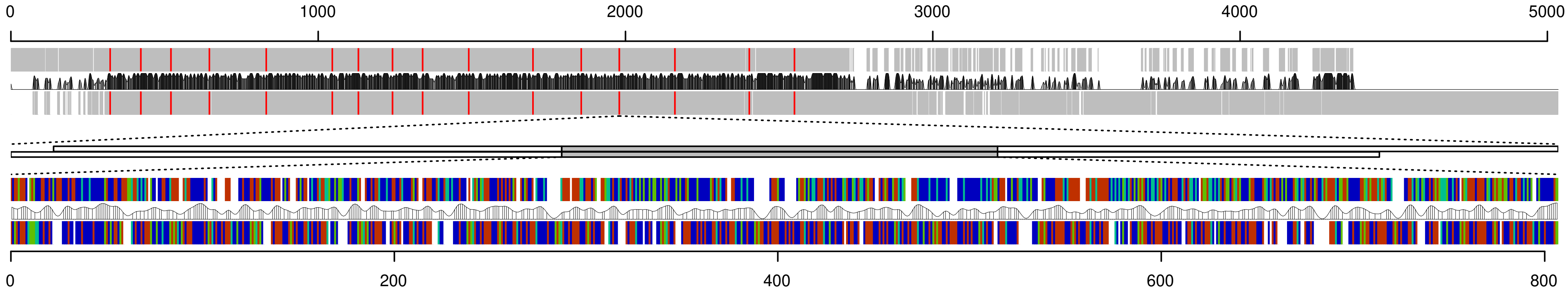

Danio rerio (ENSDART00000172618), Pygocentrus nattereri (ENSPNAT00000012776)

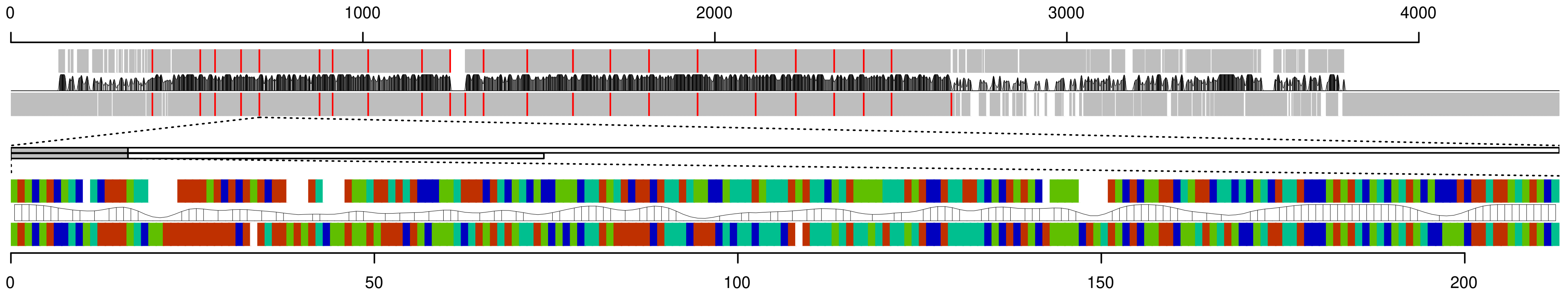

Danio rerio (ENSDART00000163449), Pygocentrus nattereri (ENSPNAT00000017825)

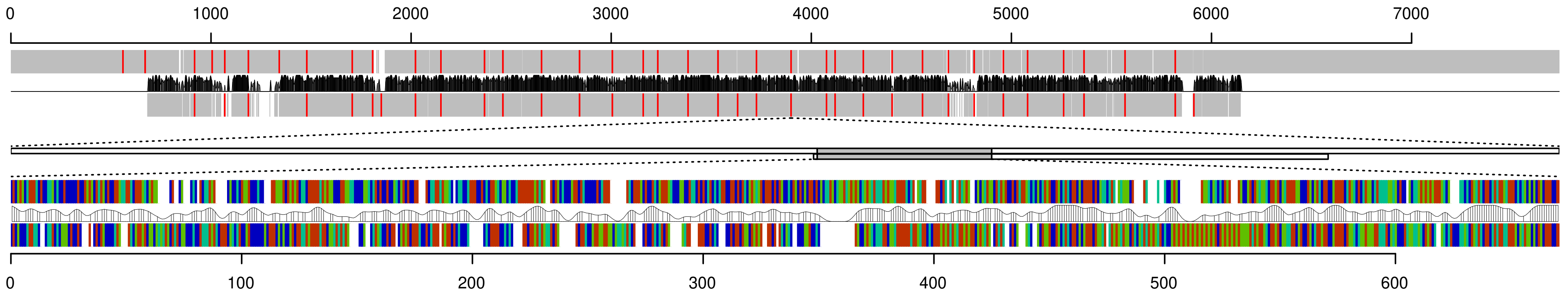

Danio rerio (ENSDART00000121614), Astyanax mexicanus (ENSAMXT00000051338)

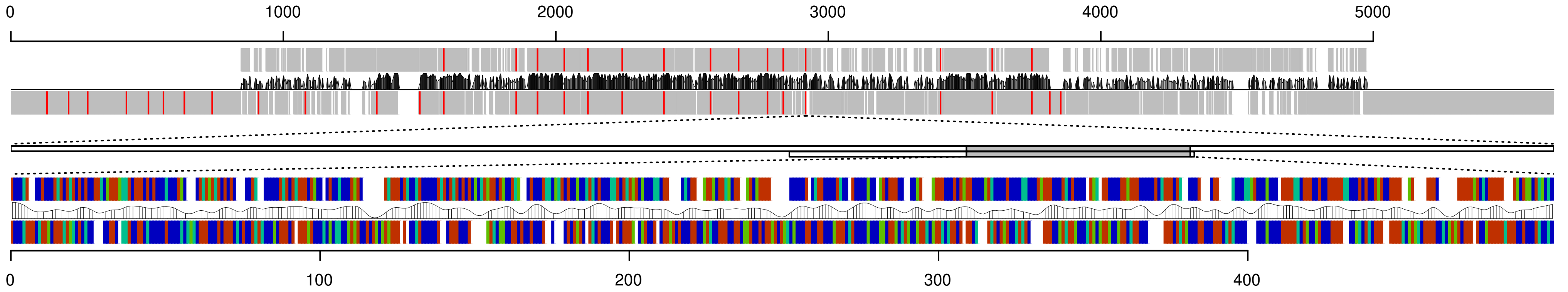

Danio rerio (ENSDART00000037140), Amphiprion percula (ENSAPET00000035384)

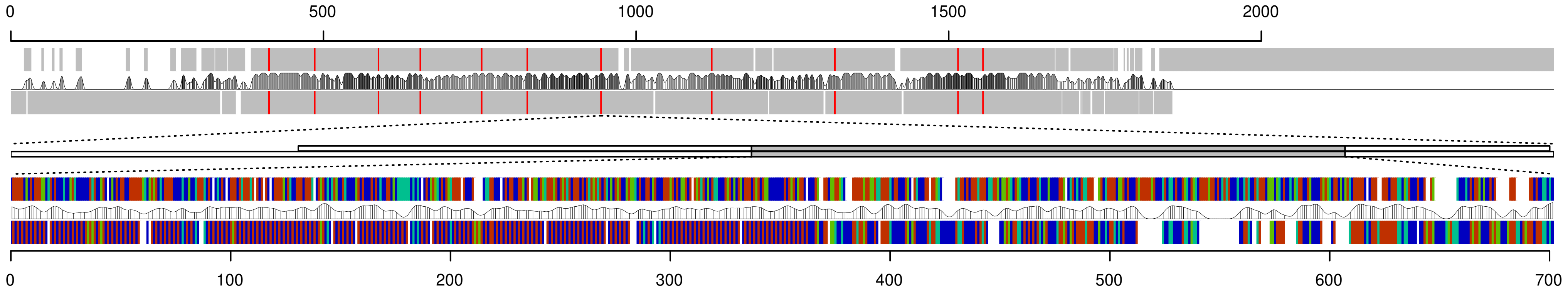

Danio rerio (ENSDART00000143710), Amphiprion percula (ENSAPET00000007857)

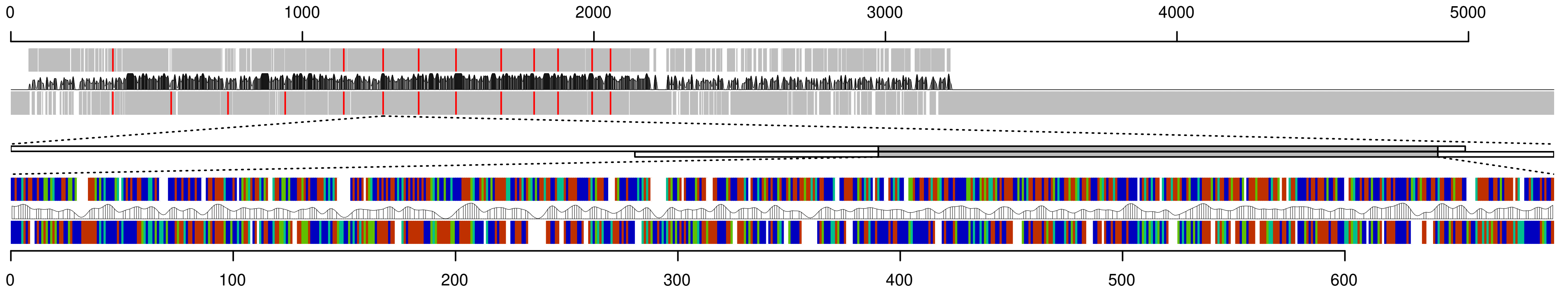

Danio rerio (ENSDART00000109130), Astyanax mexicanus (ENSAMXT00000004883)

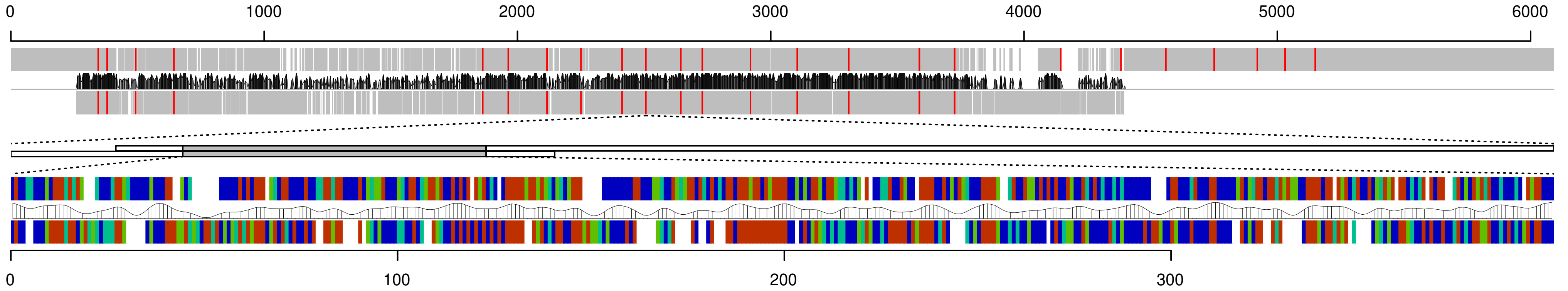

Danio rerio (ENSDART00000090254), Oryzias melastigma (ENSOMET00000016638)

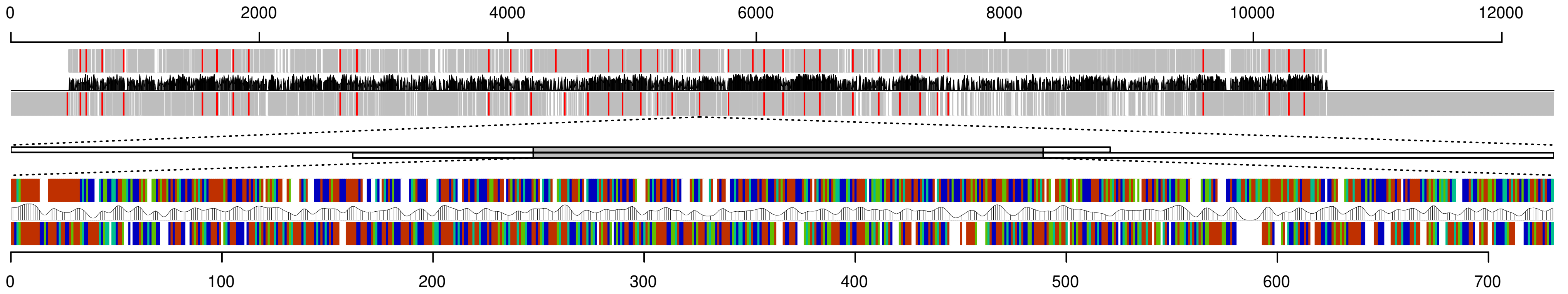

Danio rerio (ENSDART00000139710), Pygocentrus nattereri (ENSPNAT00000022121)

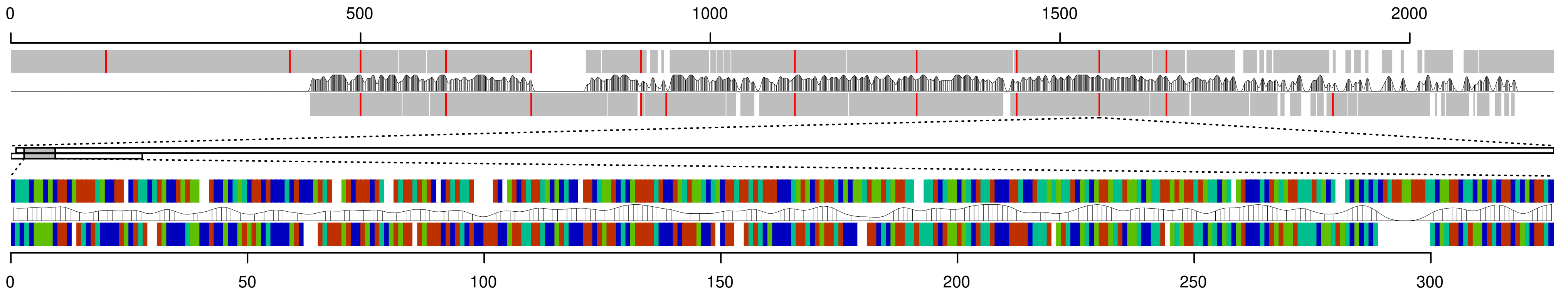

Danio rerio (ENSDART00000076061), Astyanax mexicanus (ENSAMXT00000051503)

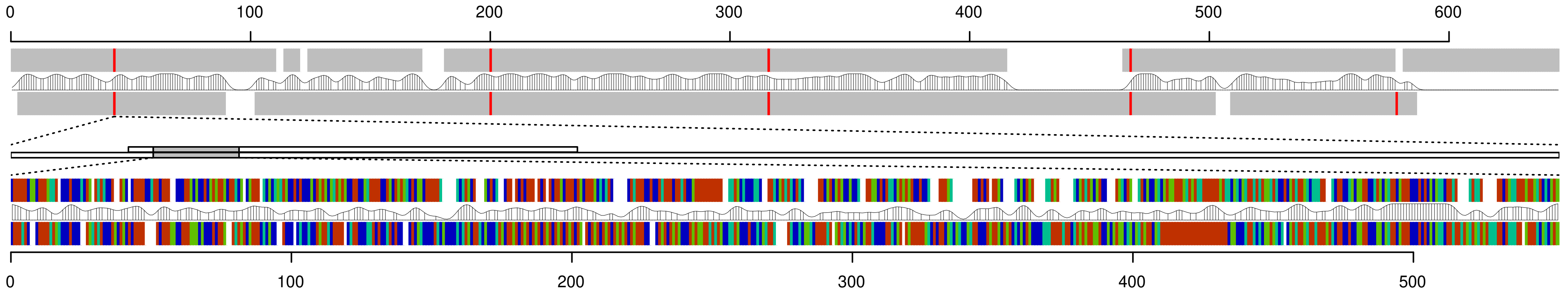

Danio rerio (ENSDART00000109980), Poecilia formosa (ENSPFOT00000030021)

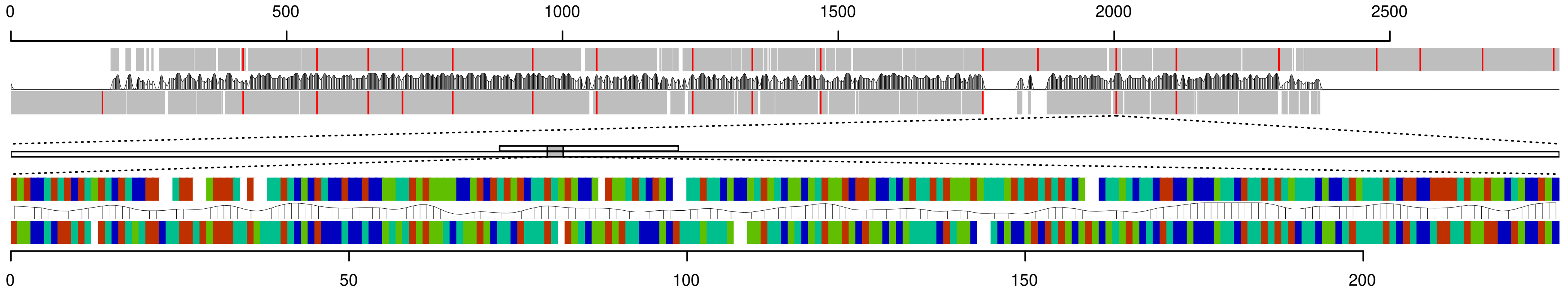

Danio rerio (ENSDART00000168469), Electrophorus electricus (ENSEEET00000017355)

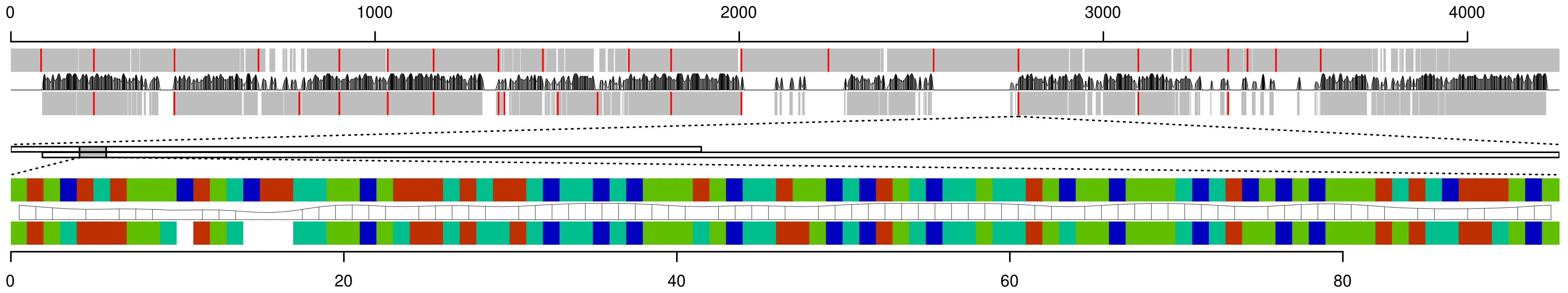

Danio rerio (ENSDART00000105590), Gouania willdenowi (ENSGWIT00000051188)

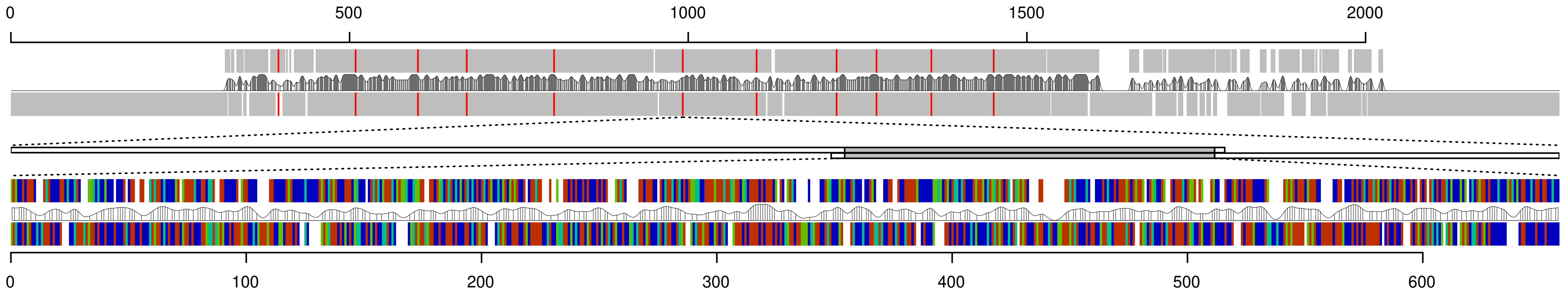

Danio rerio (ENSDART00000170562), Pygocentrus nattereri (ENSPNAT00000005663)

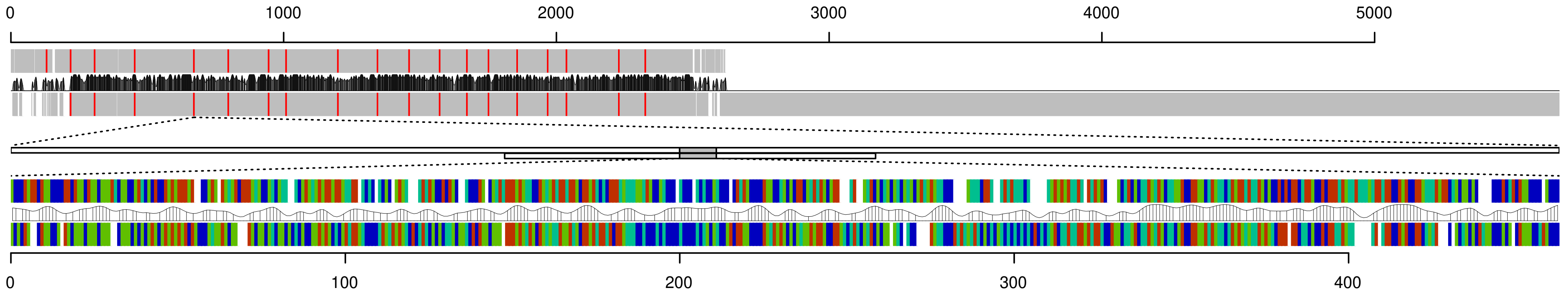

Danio rerio (ENSDART00000130413), Cottoperca gobio (ENSCGOT00000033968)

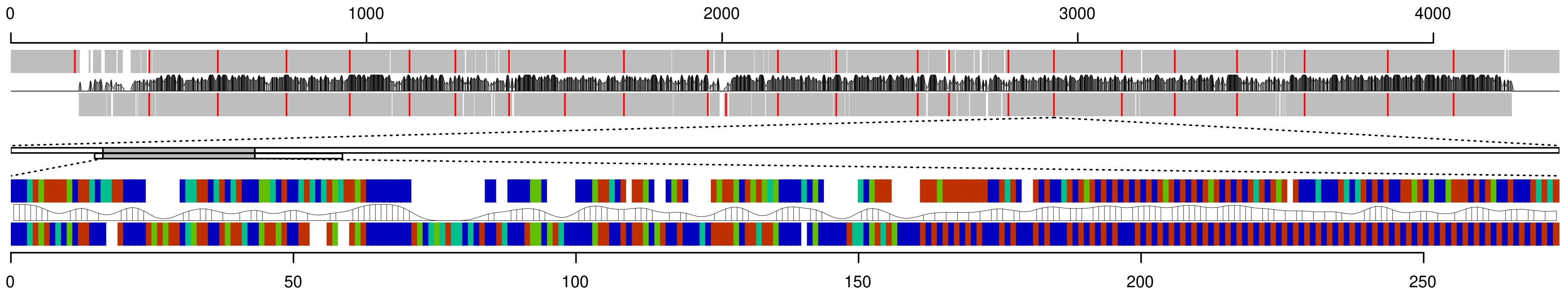

Danio rerio (ENSDART00000079695), Esox lucius (ENSELUT00000017565)

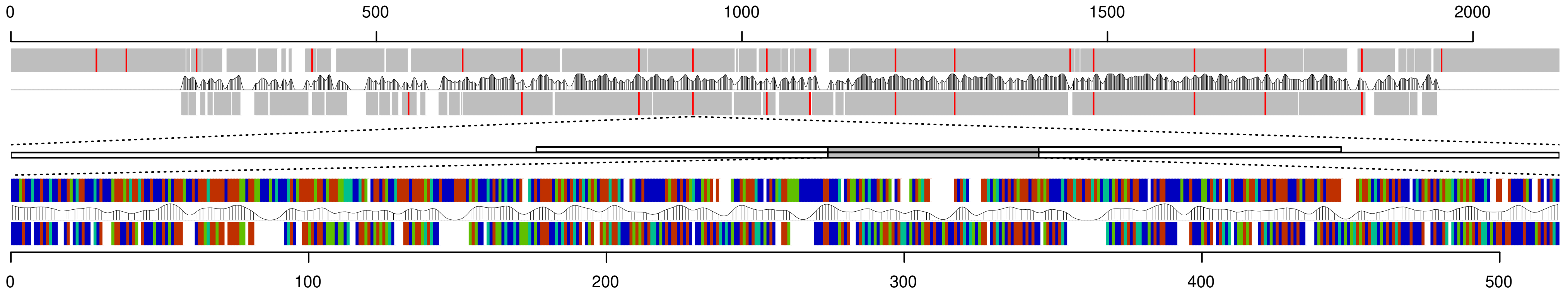

Danio rerio (ENSDART00000140386), Pygocentrus nattereri (ENSPNAT00000034920)

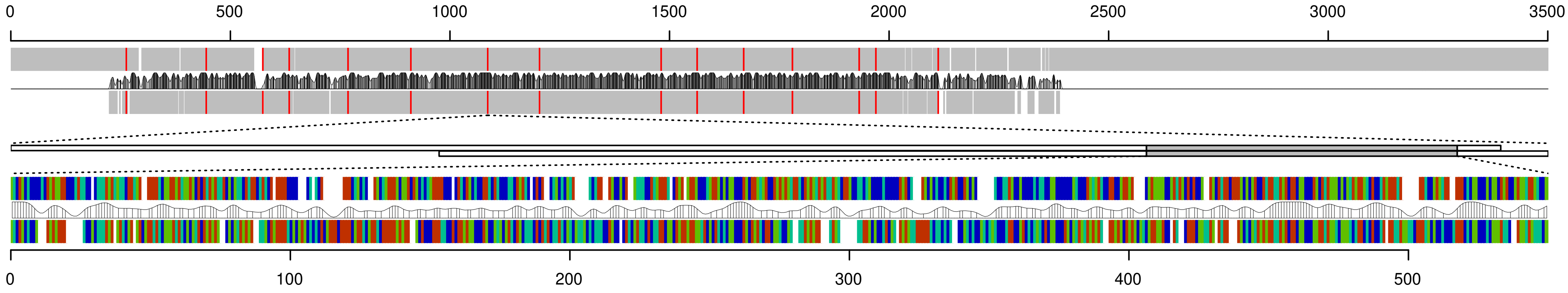

Danio rerio (ENSDART00000171531), Cottoperca gobio (ENSCGOT00000040294)

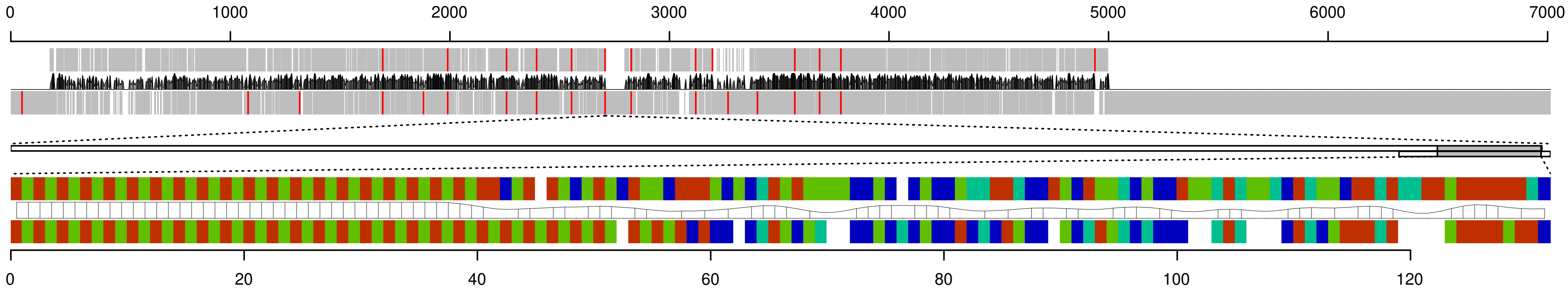

Danio rerio (ENSDART00000158445), Ictalurus punctatus (ENSIPUT00000021557)

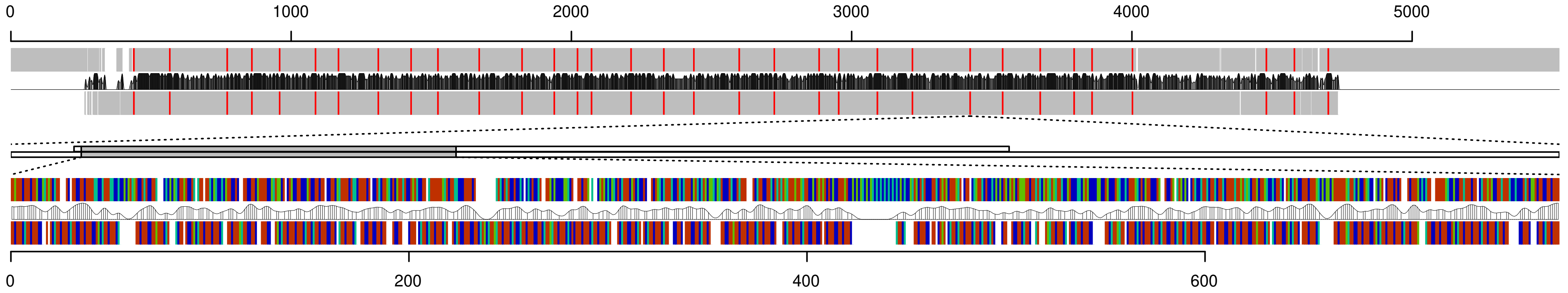

Danio rerio (ENSDART00000017599), Pygocentrus nattereri (ENSPNAT00000013031)

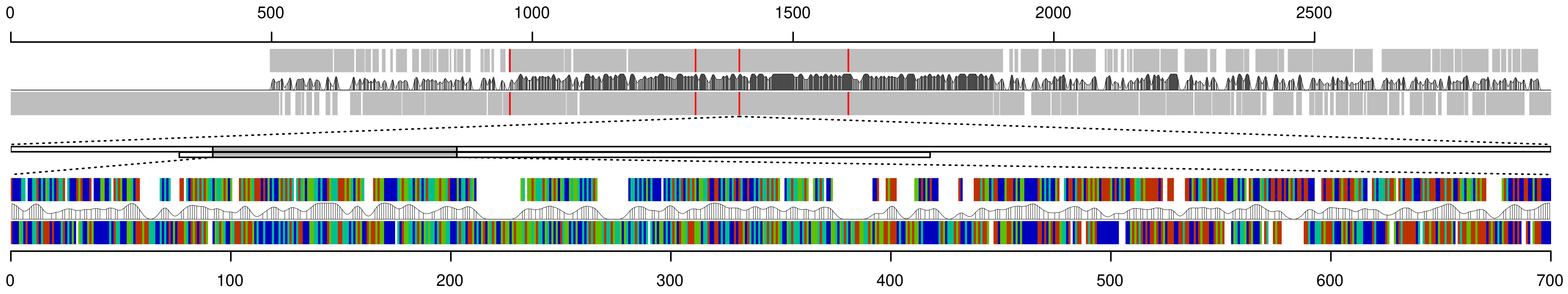

Danio rerio (ENSDART00000073564), Betta splendens (ENSBSLT00000034380)

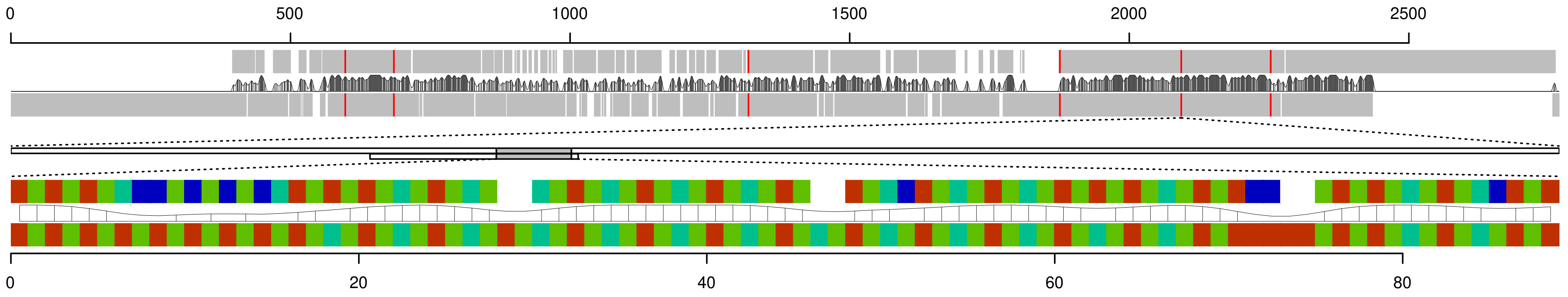

Danio rerio (ENSDART00000134918), Amphiprion percula (ENSAPET00000009144)

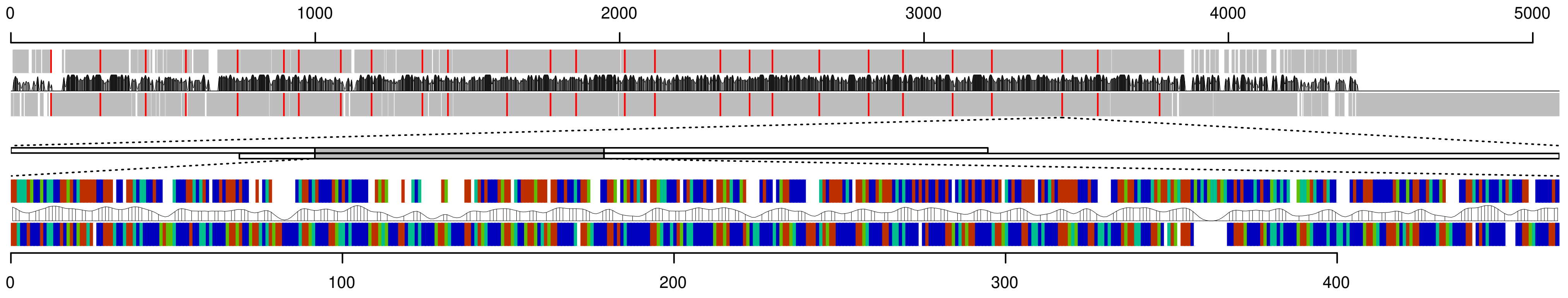

Danio rerio (ENSDART00000081772), Cottoperca gobio (ENSCGOT00000041917)

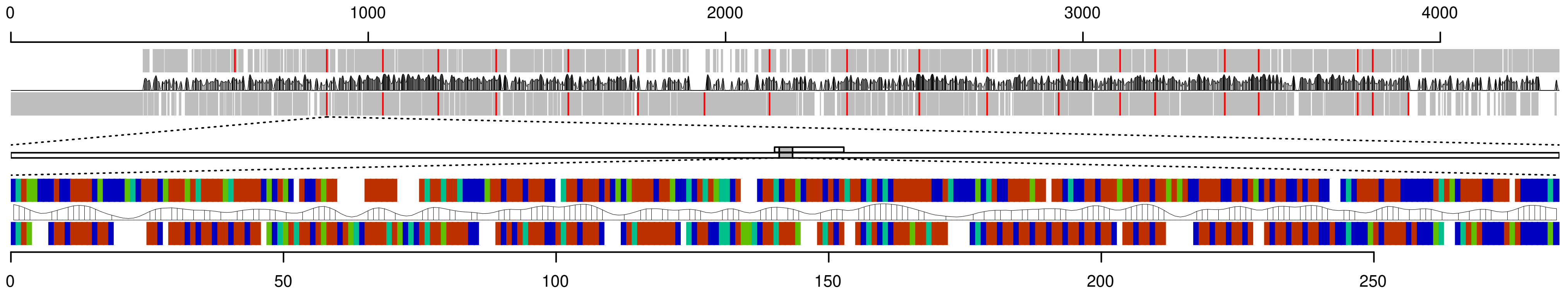

Danio rerio (ENSDART00000149254), Poecilia latipinna (ENSPLAT00000024880)

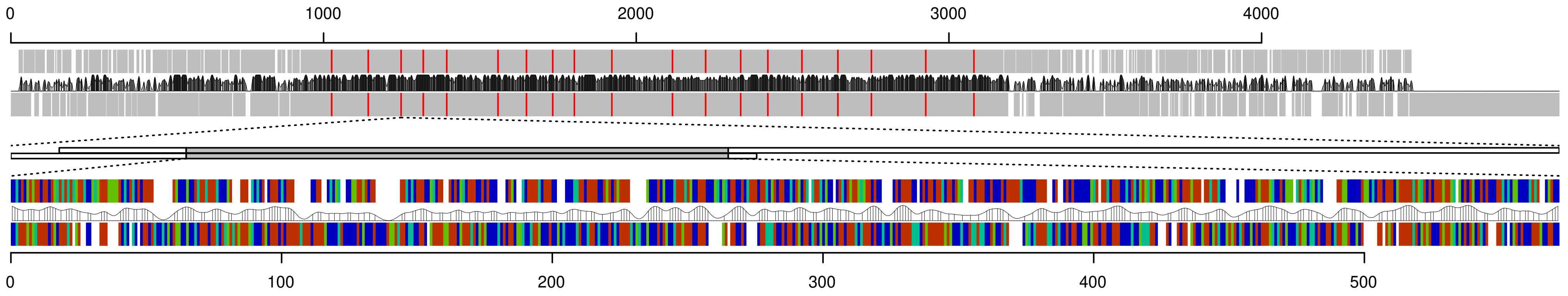

Danio rerio (ENSDART00000048469), Astyanax mexicanus (ENSAMXT00000002337)

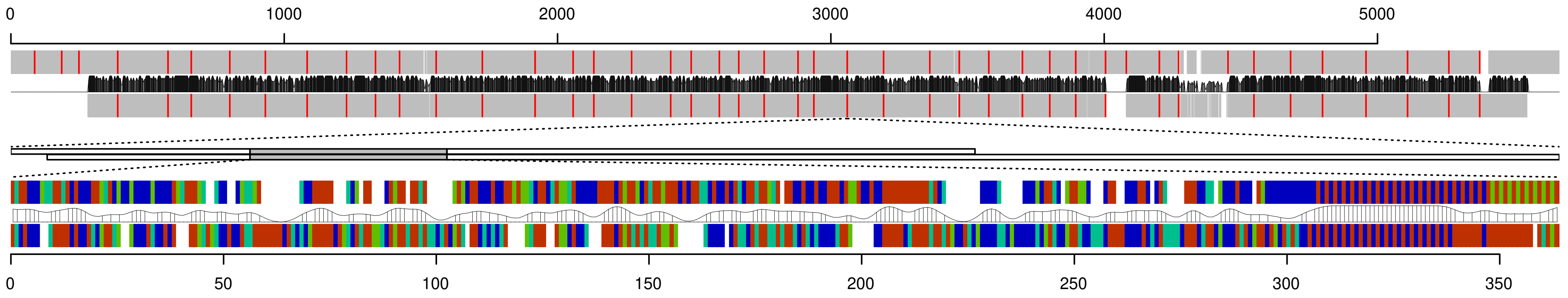

Danio rerio (ENSDART00000157919), Astyanax mexicanus (ENSAMXT00000012542)

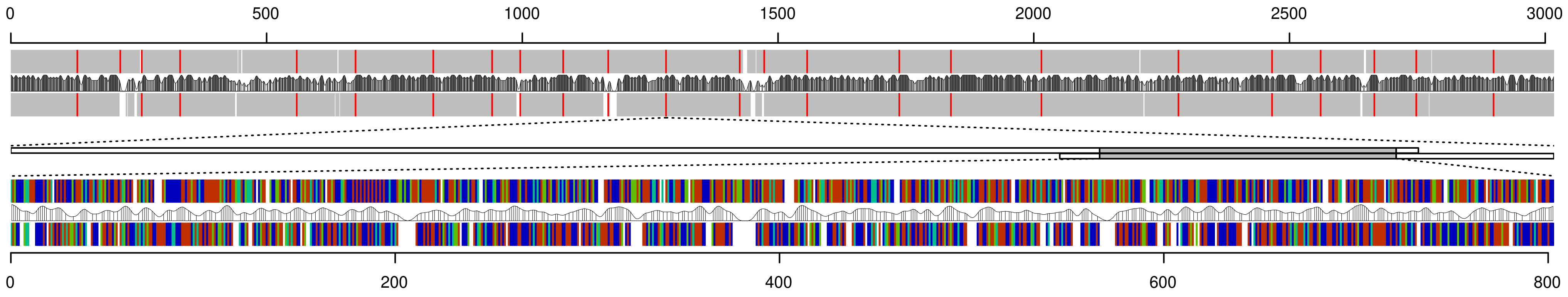

Danio rerio (ENSDART00000110552), Pygocentrus nattereri (ENSPNAT00000000222)

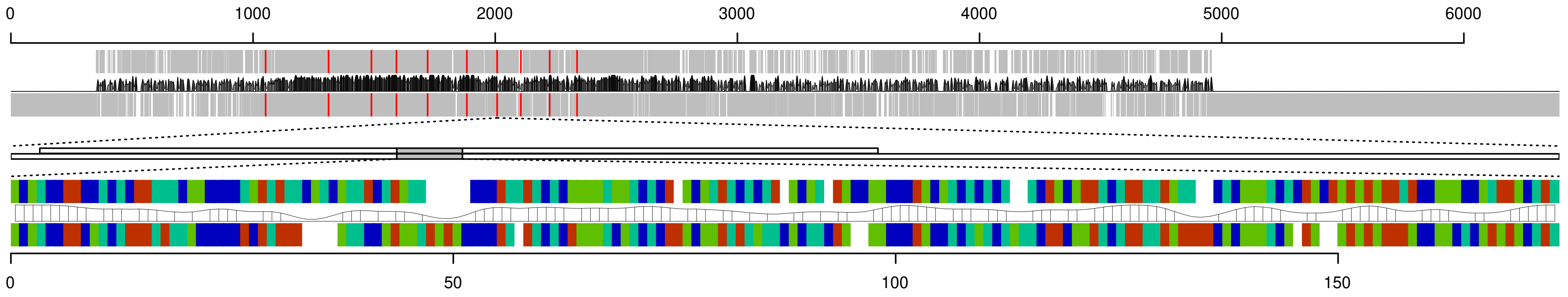

Danio rerio (ENSDART00000138911), Acanthochromis polyacanthus (ENSAPOT00000024725)

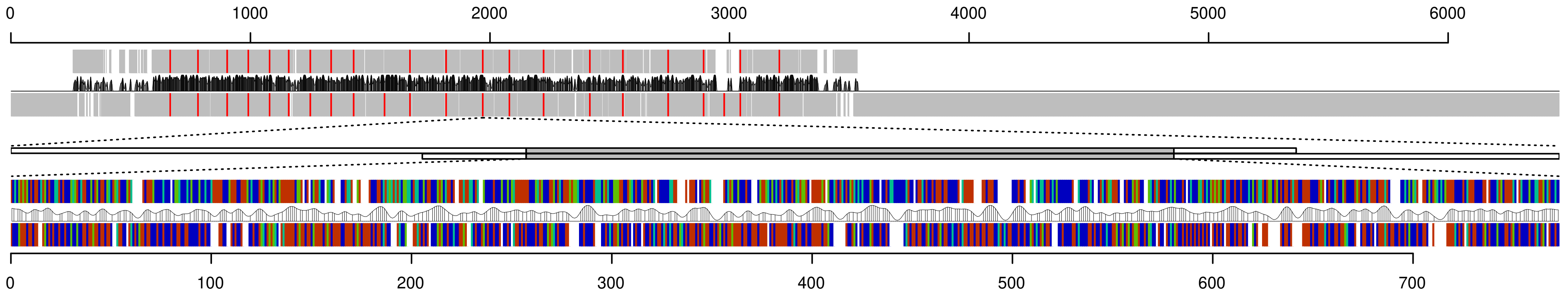

Danio rerio (ENSDART00000012581), Amphiprion percula (ENSAPET00000016221)

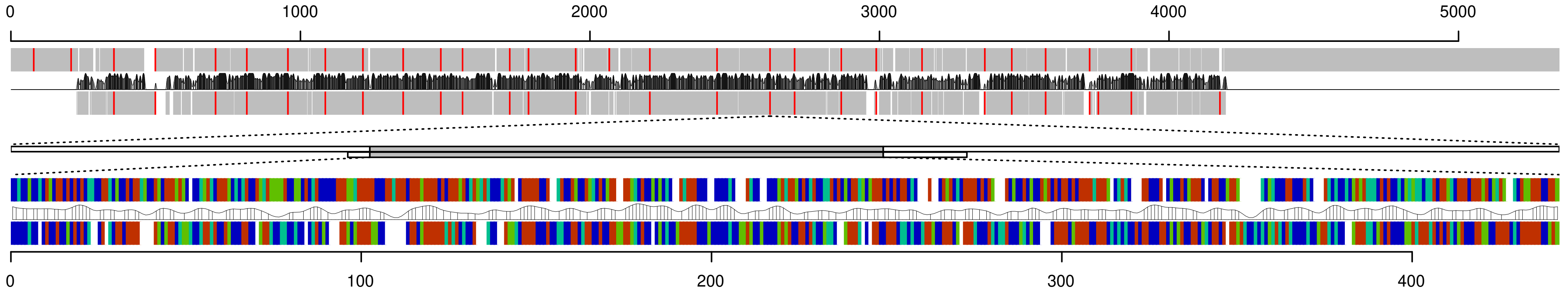

Danio rerio (ENSDART00000086765), Pygocentrus nattereri (ENSPNAT00000004176)

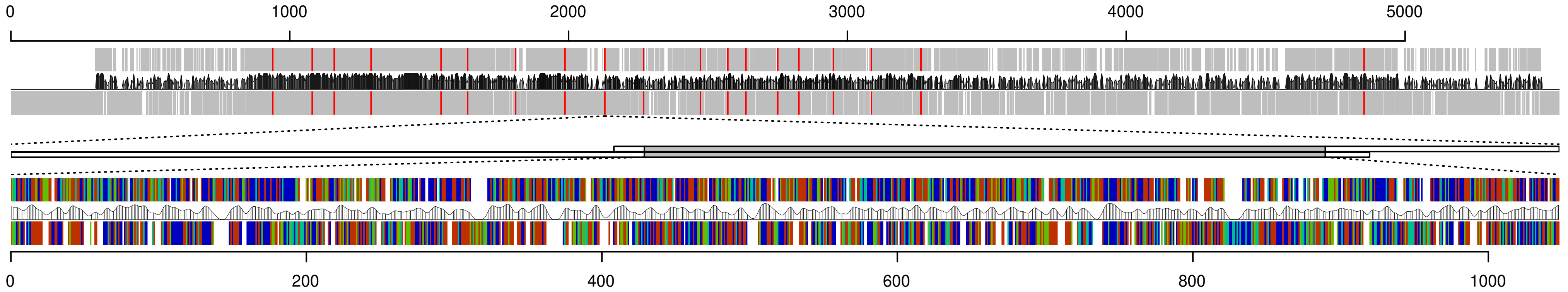

Danio rerio (ENSDART00000052349), Pygocentrus nattereri (ENSPNAT00000011370)

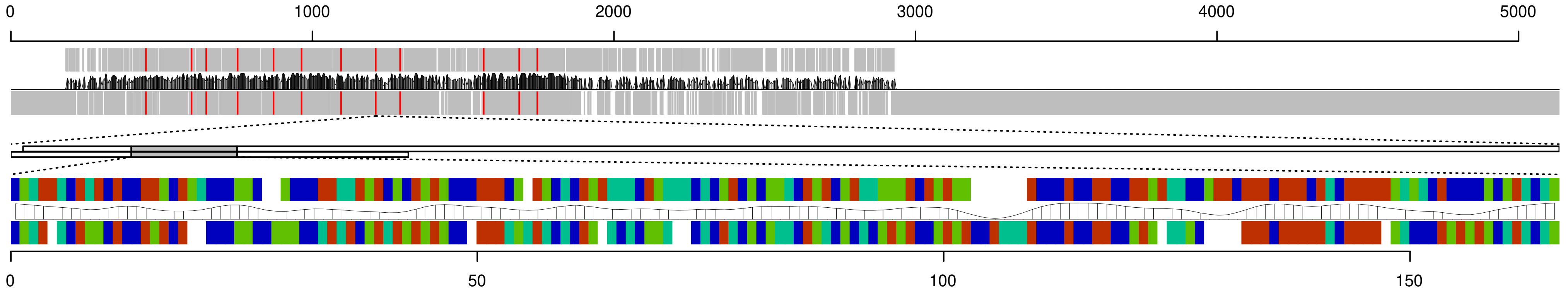

Danio rerio (ENSDART00000129776), Cynoglossus semilaevis (ENSCSET00000010526)

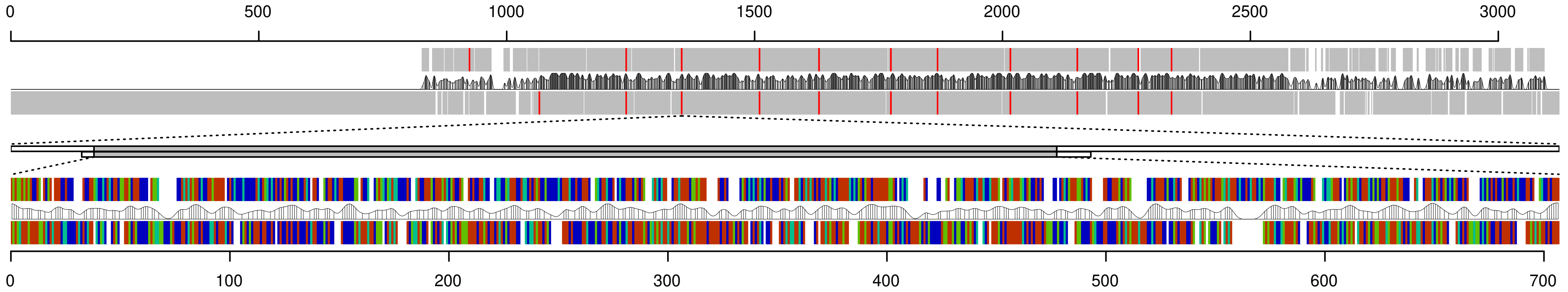

Danio rerio (ENSDART00000149688), Amphiprion ocellaris (ENSAOCT00000002957)

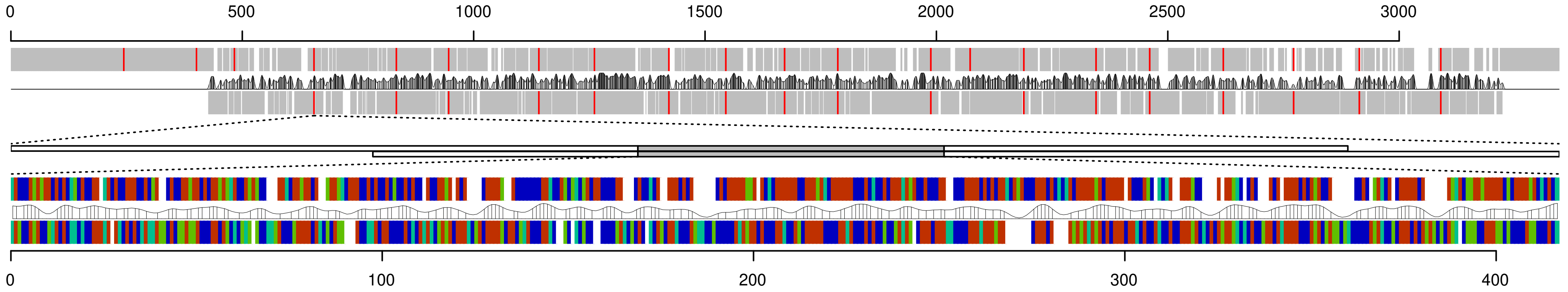

Supplement: Supplementary file 6 — Transcript and intron alignments for points in Fig. S14. Each panel shows the maximally scoring alignment between D. rerio and teleost intron orthologues (lower) and transcript alignment (upper) used to establish the intron orthology. Grey, white and red parts indicate aligned exonic sequence, gaps and positions of intron meta-characters respectively. Colours in intron alignment represent bases (A blue, C cyan, G green, T brown, N grey, gap white). Curves lying between sequence representations show a normal kernel density smoothed estimate of local similarity (9 bp window, standard deviation two); vertical lines indicate matches. Region between exon and intron alignments indicates the location of the maximally scoring alignment in the introns. Upper sequence D. rerio. Files 6–10 and 11–15 contain alignments to teleost and mammalian sequences respectively. Each file corresponds to one panel in Fig. S17 and to one specific teleost size class: Files 6,11: long (E,J), 7,12: medium (D,I), 8,13: short.2 (C,H), 9,14 short (B, G) and 10,15 ctl (A,F). [file 12864_2022_8760_MOESM6_ESM.zip › 12864_2022_8760_MOESM10_ESM.pdf]
