# Supplementary material for: Intron size minimisation in teleosts
Source: BMC Genomics. 2022 Sep 1;23:628. doi: 10.1186/s12864-022-08760-w (PMC9438311; doi:10.1186/s12864-022-08760-w)

Danio rerio (ENSDART00000165458), Astyanax mexicanus (ENSAMXT00000049747)

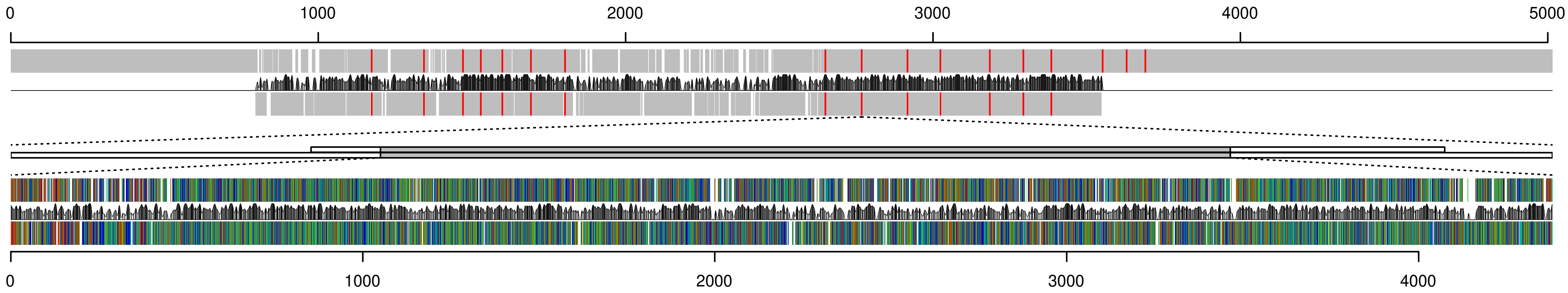

Danio rerio (ENSDART00000148257), Astyanax mexicanus (ENSAMXT00000037919)

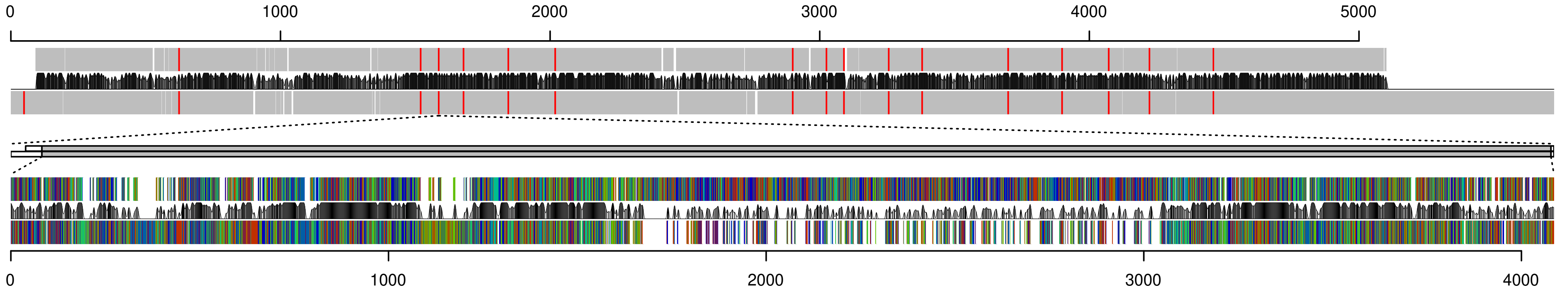

Danio rerio (ENSDART00000111537), Pygocentrus nattereri (ENSPNAT00000007900)

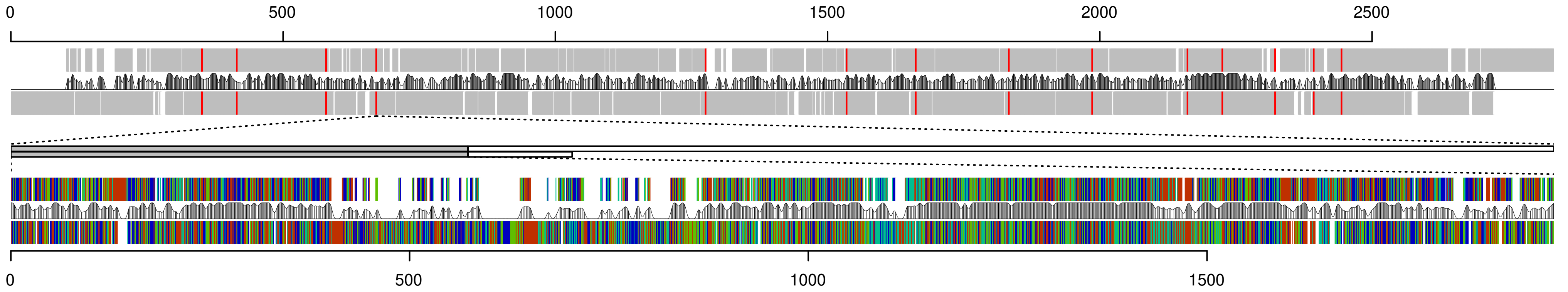

Danio rerio (ENSDART00000005562), Astyanax mexicanus (ENSAMXT00000057256)

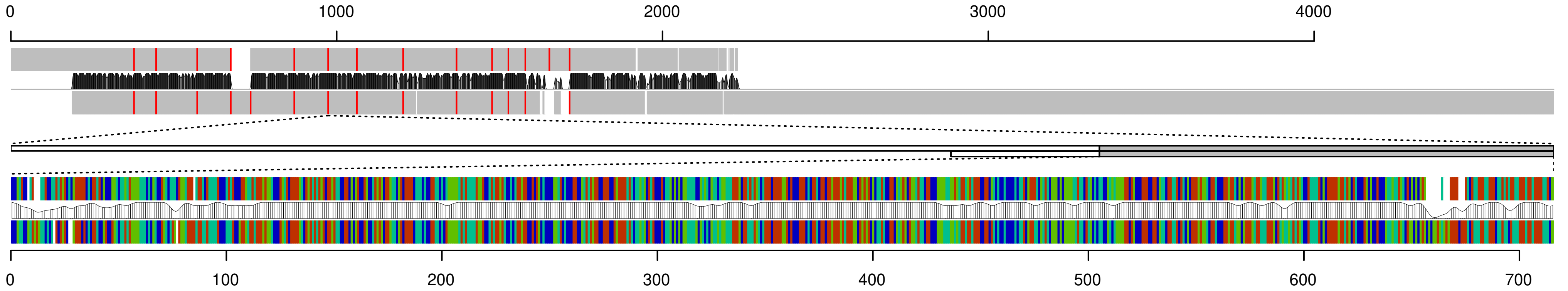

Danio rerio (ENSDART00000142818), Electrophorus electricus (ENSEEET00000038793)

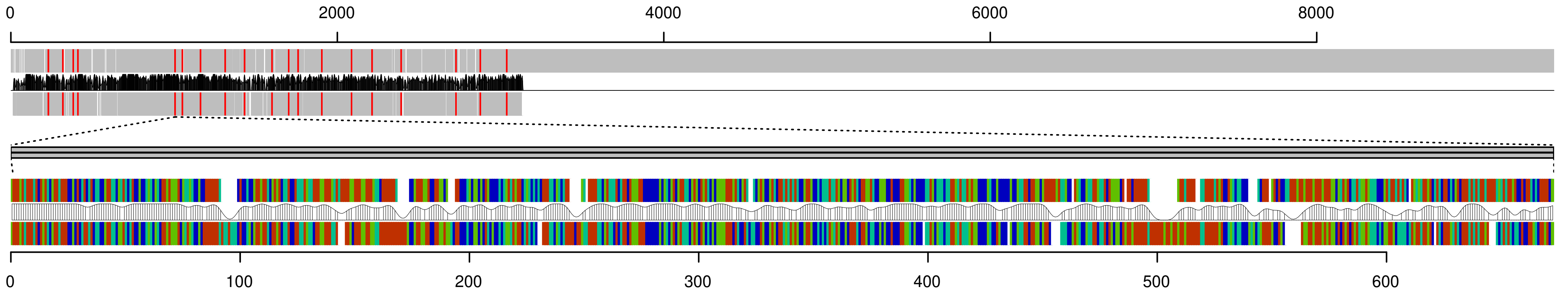

Danio rerio (ENSDART00000074789), Pygocentrus nattereri (ENSPNAT00000027912)

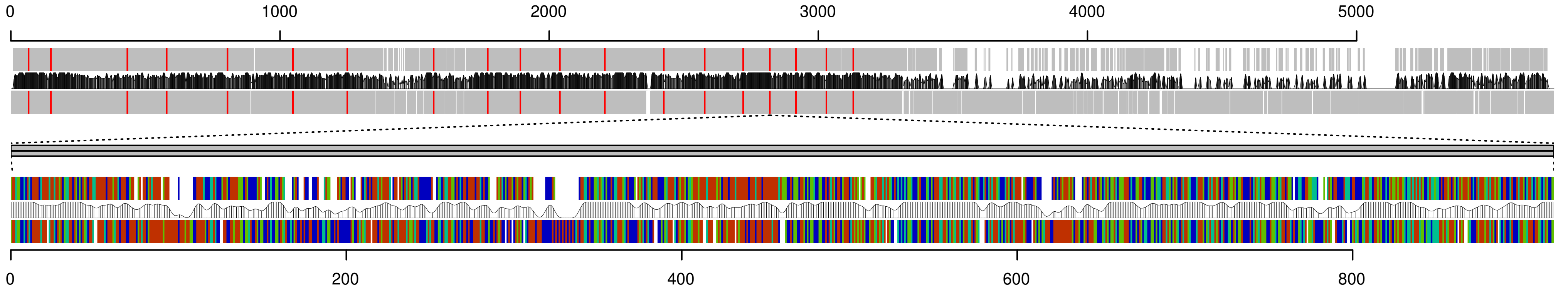

Danio rerio (ENSDART00000163766), Ictalurus punctatus (ENSIPUT00000014636)

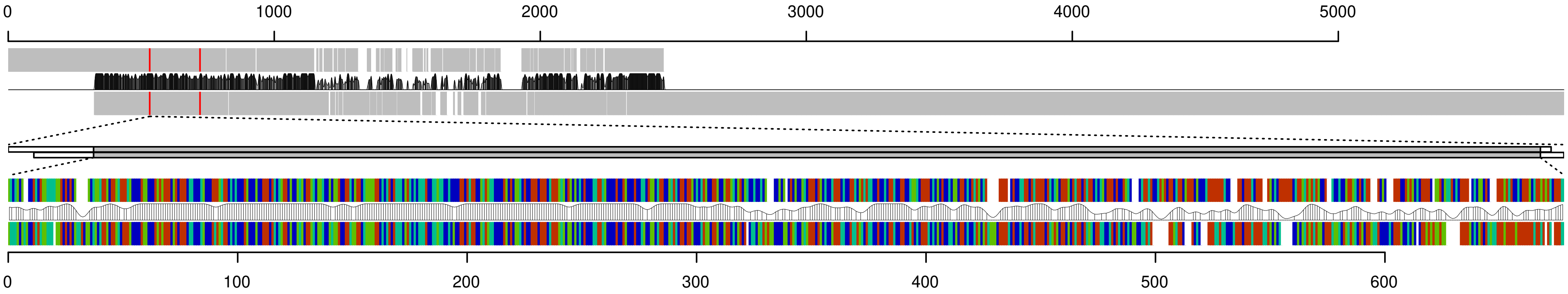

Danio rerio (ENSDART00000077304), Pygocentrus nattereri (ENSPNAT00000007963)

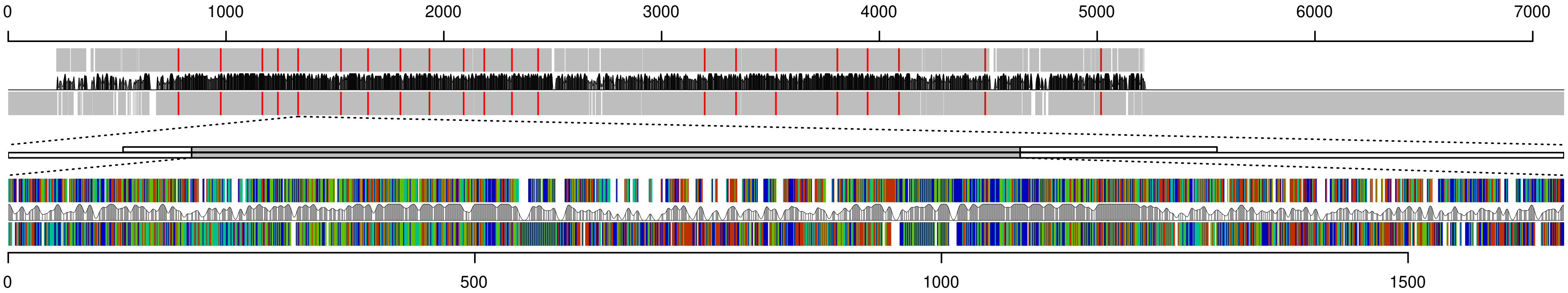

Danio rerio (ENSDART00000103146), Astyanax mexicanus (ENSAMXT00000050659)

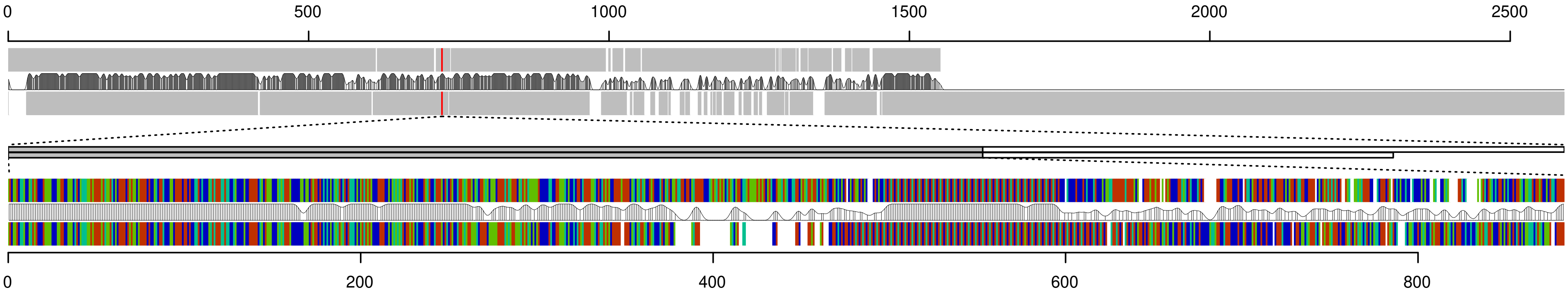

Danio rerio (ENSDART00000122037), Pygocentrus nattereri (ENSPNAT00000025552)

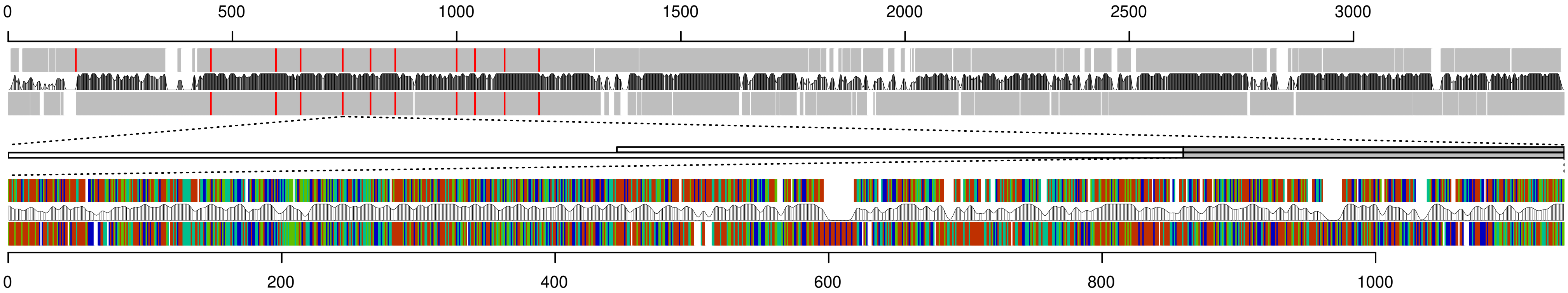

Danio rerio (ENSDART00000111537), Pygocentrus nattereri (ENSPNAT00000007900)

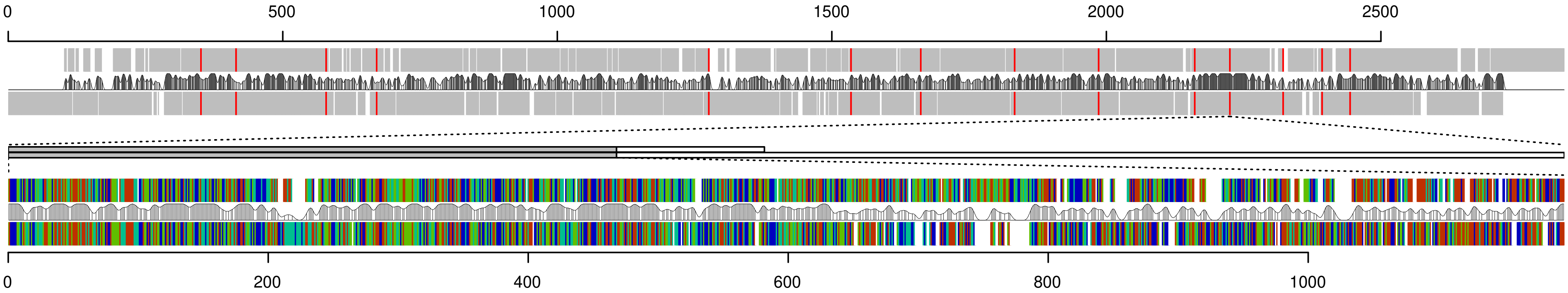

Danio rerio (ENSDART00000126490), Electrophorus electricus (ENSEEET00000040603)

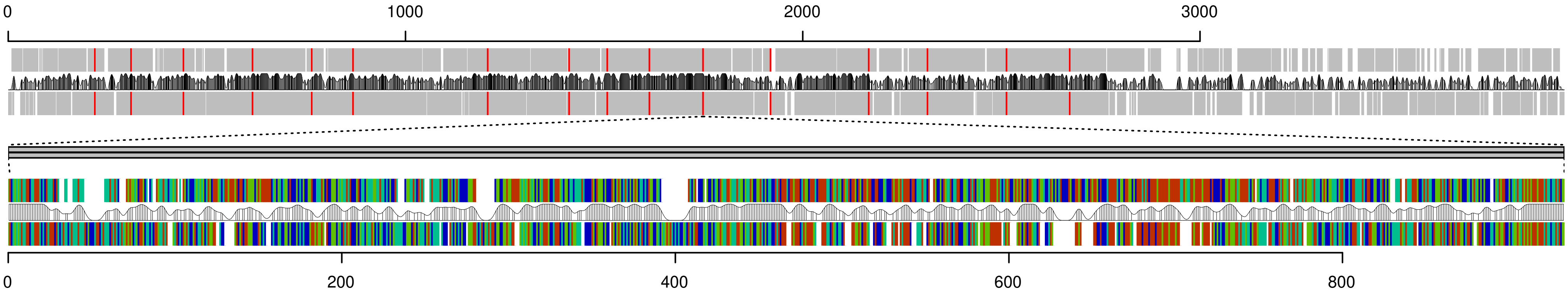

Danio rerio (ENSDART00000148257), Astyanax mexicanus (ENSAMXT00000037919)

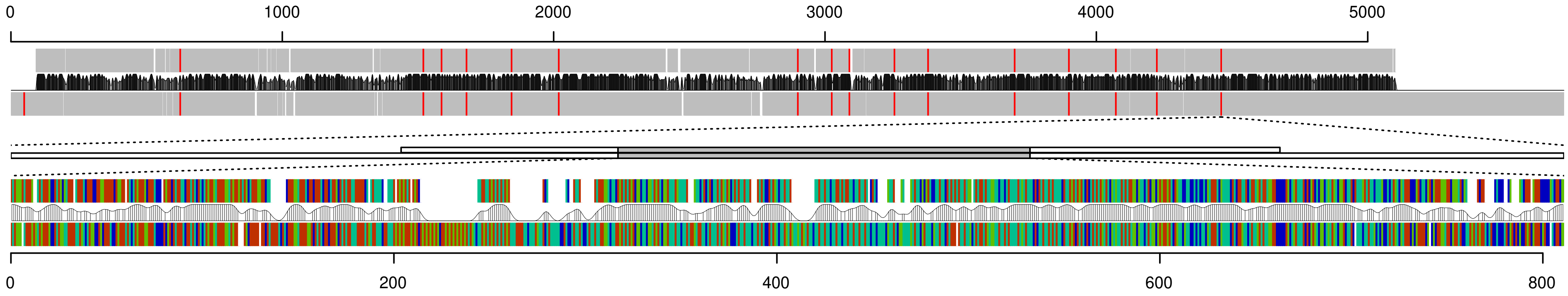

Danio rerio (ENSDART00000005562), Astyanax mexicanus (ENSAMXT00000057256)

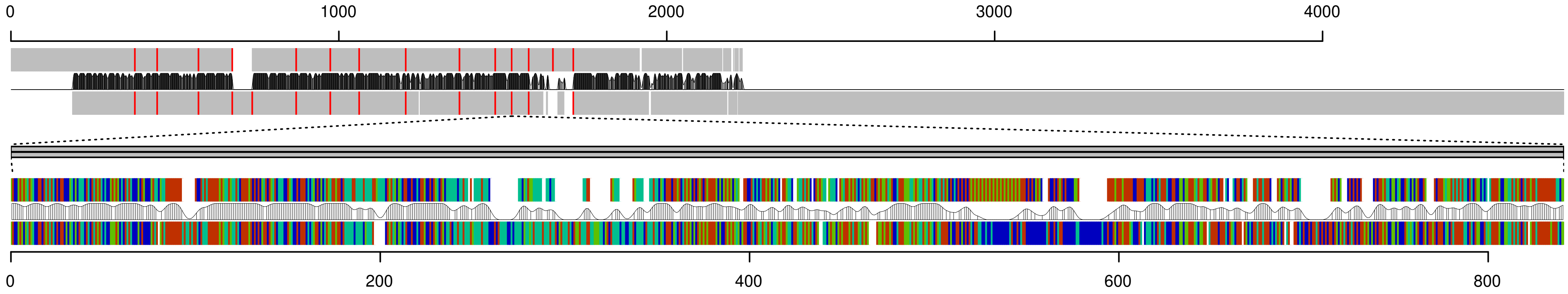

Danio rerio (ENSDART00000017269), Pygocentrus nattereri (ENSPNAT00000001289)

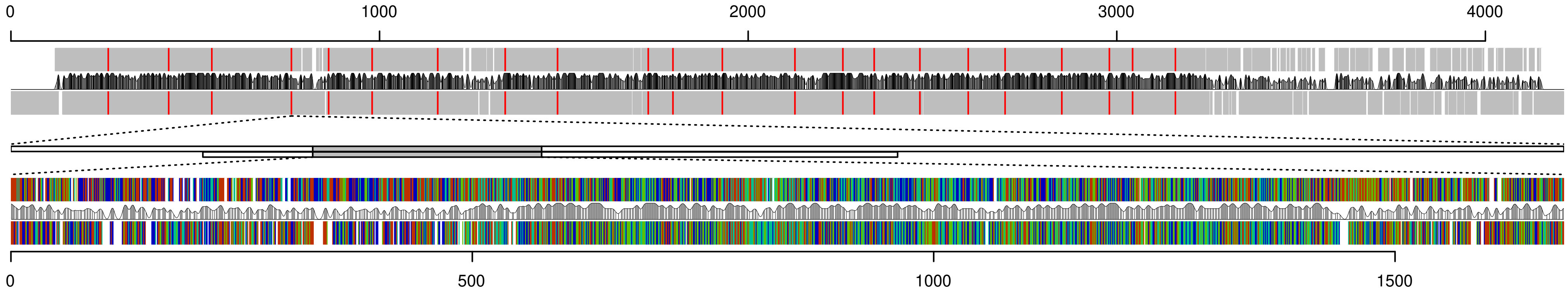

Danio rerio (ENSDART00000036419), Pygocentrus nattereri (ENSPNAT00000009693)

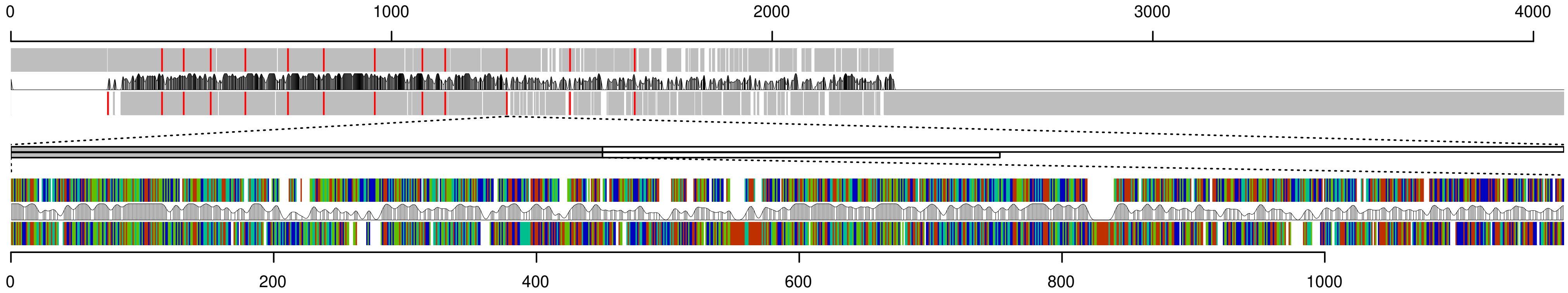

Danio rerio (ENSDART00000103434), Ictalurus punctatus (ENSIPUT00000010200)

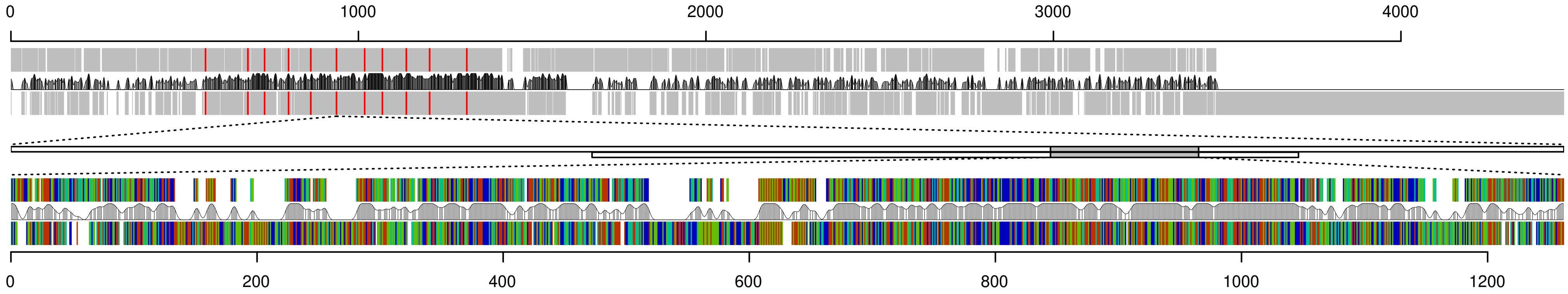

Danio rerio (ENSDART00000036419), Pygocentrus nattereri (ENSPNAT00000009693)

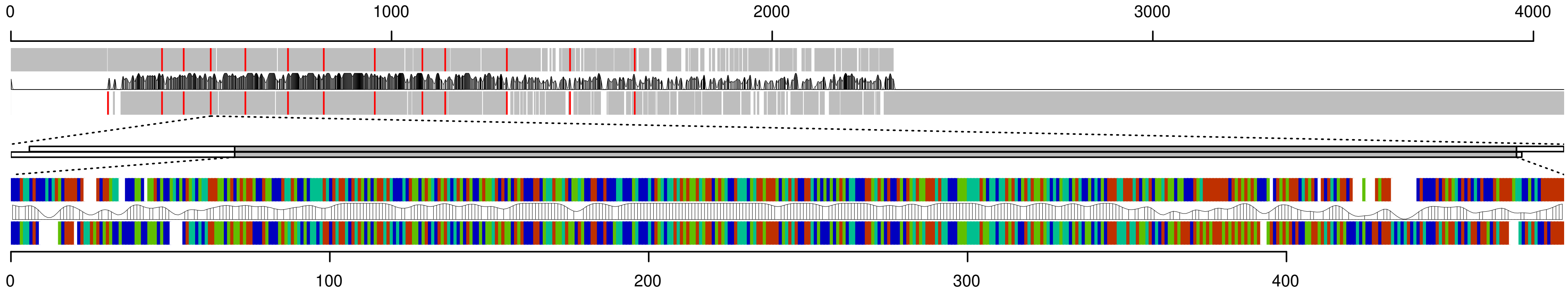

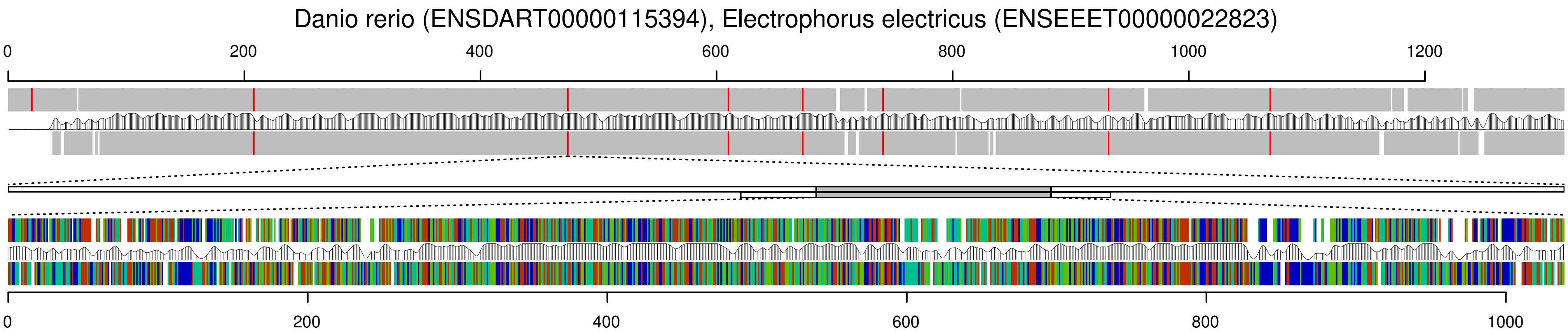
Danio rerio (ENSDART00000115394), Electrophorus electricus (ENSEEET00000022823)
0 200 400 600 800 1000 1200
0 200 400 600 800 1000

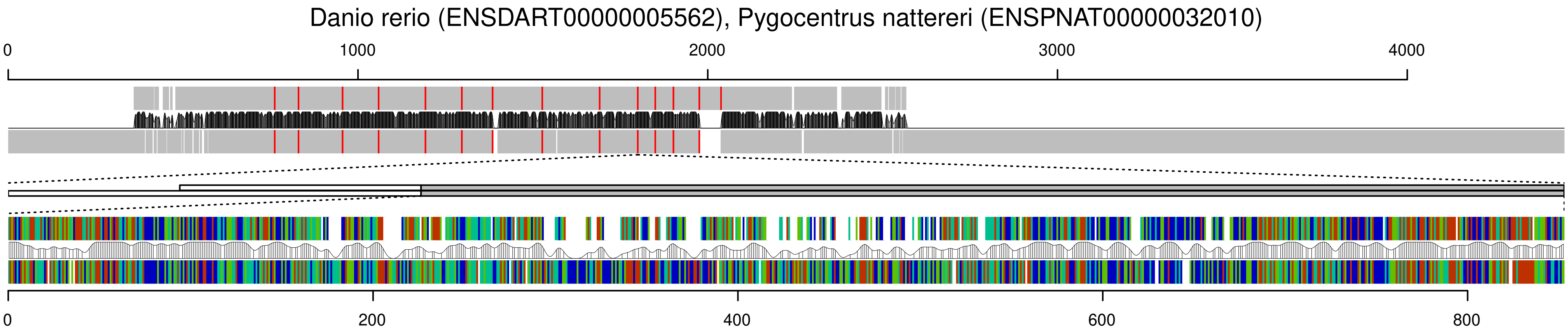
Danio rerio (ENSDART00000005562), Pygocentrus nattereri (ENSPNAT00000032010)
0 1000 2000 3000 4000
0 200 400 600 800

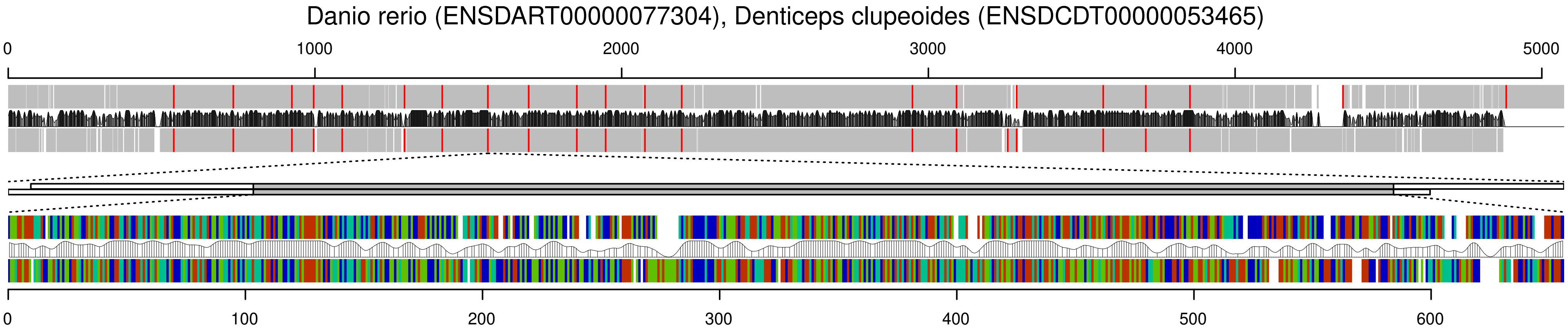
Danio rerio (ENSDART00000077304), Denticeps clupeoides (ENSDCDT00000053465)
0 1000 2000 3000 4000 5000
0 100 200 300 400 500 600

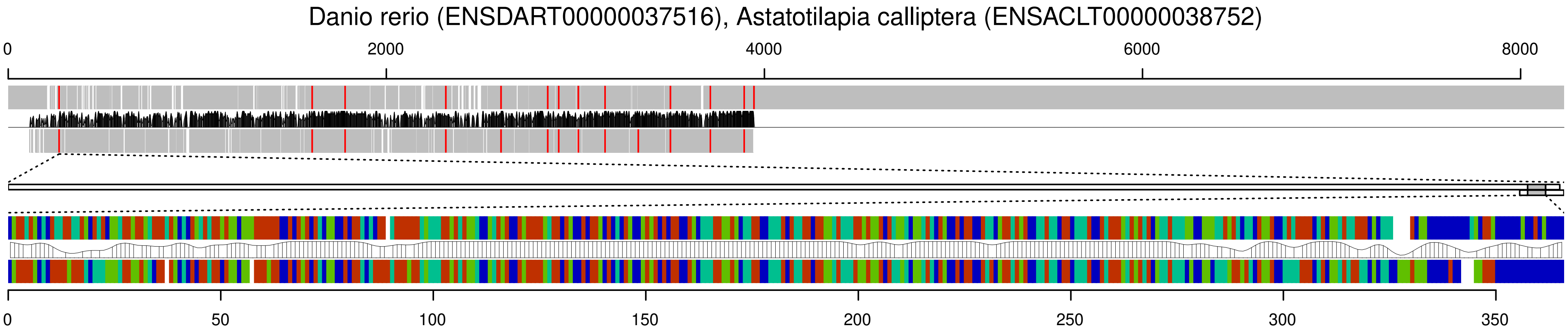
Danio rerio (ENSDART00000037516), Astatotilapia calliptera (ENSACLT00000038752)
0 2000 4000 6000 8000
0 50 100 150 200 250 300 350

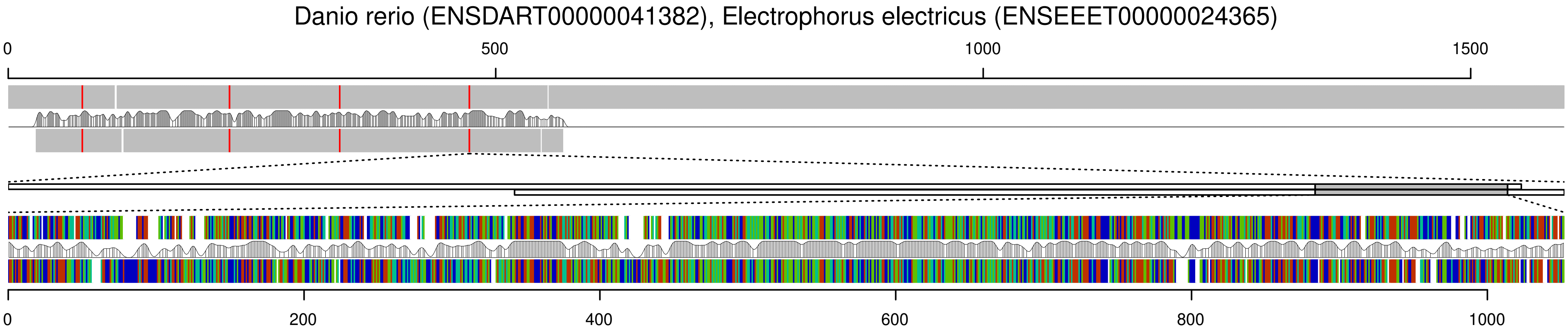
Danio rerio (ENSDART00000041382), Electrophorus electricus (ENSEEET00000024365)
0 500 1000 1500
0 200 400 600 800 1000

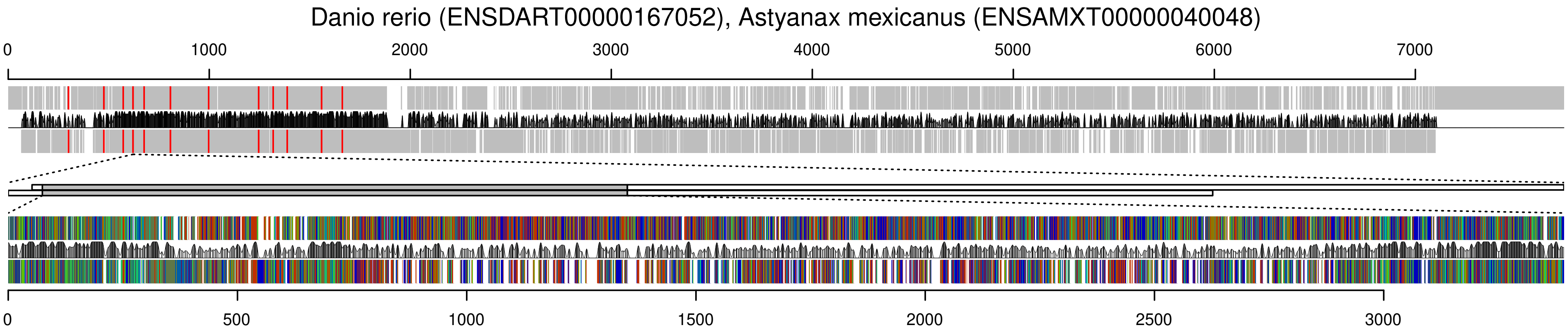
Danio rerio (ENSDART00000167052), Astyanax mexicanus (ENSAMXT00000040048)
0 1000 2000 3000 4000 5000 6000 7000
0 500 1000 1500 2000 2500 3000

Danio rerio (ENSDART00000114288), Ictalurus punctatus (ENSIPUT00000037725)

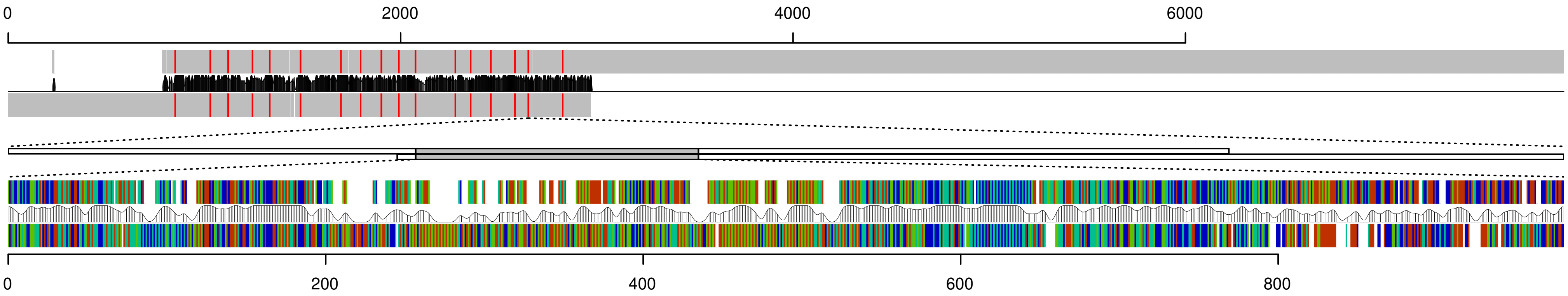

Danio rerio (ENSDART00000027756), Pygocentrus nattereri (ENSPNAT00000010620)

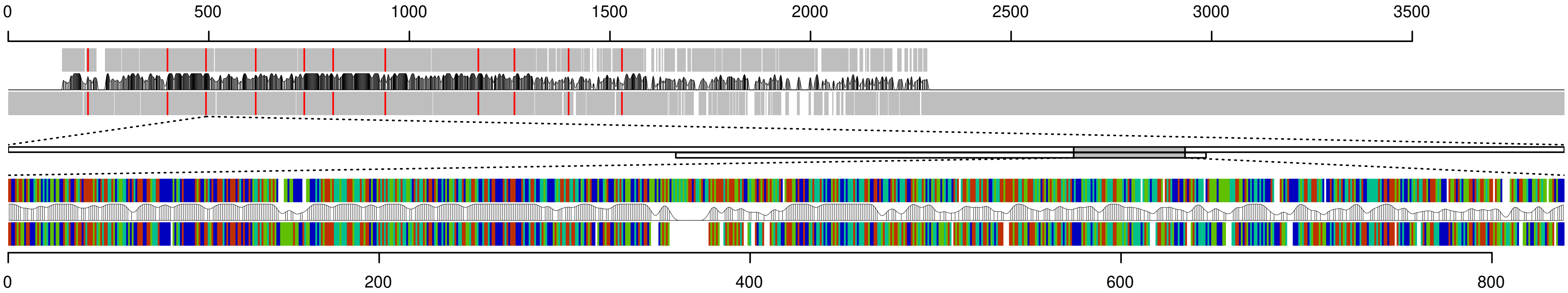

Danio rerio (ENSDART00000131175), Pygocentrus nattereri (ENSPNAT00000006950)

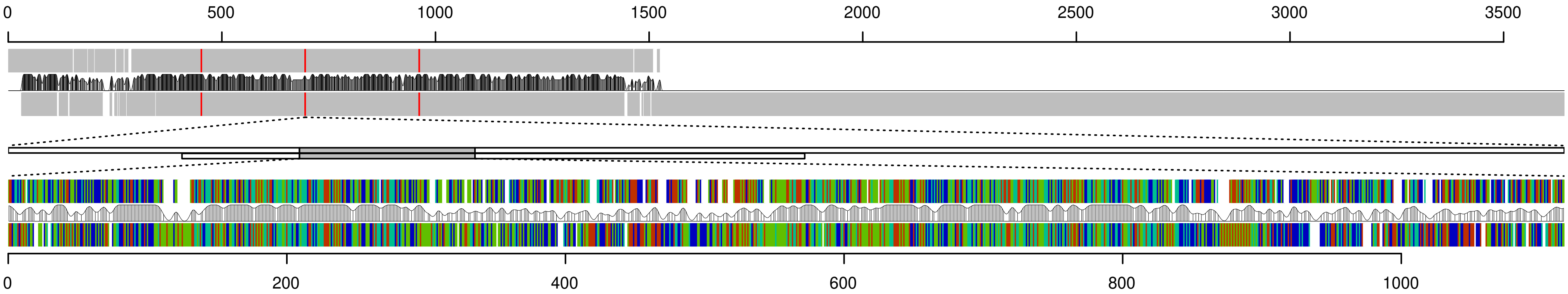

Danio rerio (ENSDART00000155020), Denticeps clupeoides (ENSDCDT00000027220)

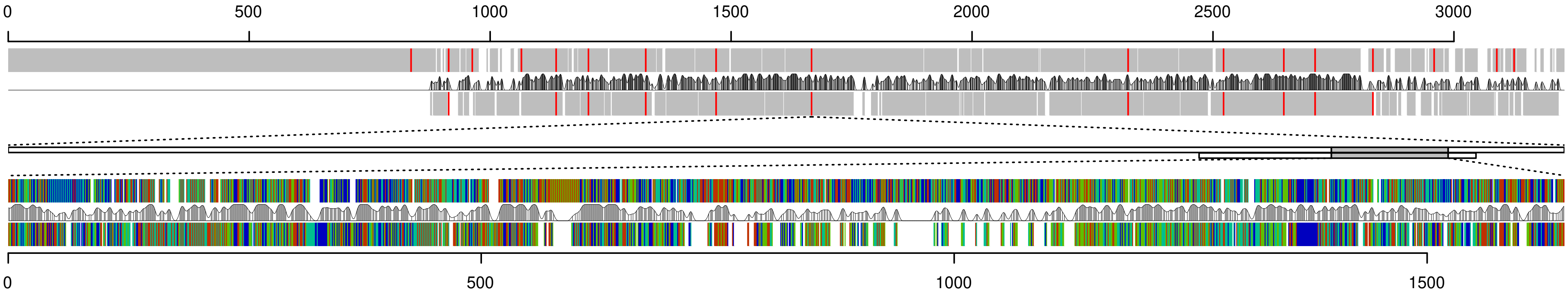

Danio rerio (ENSDART00000025621), Ictalurus punctatus (ENSIPUT00000014871)

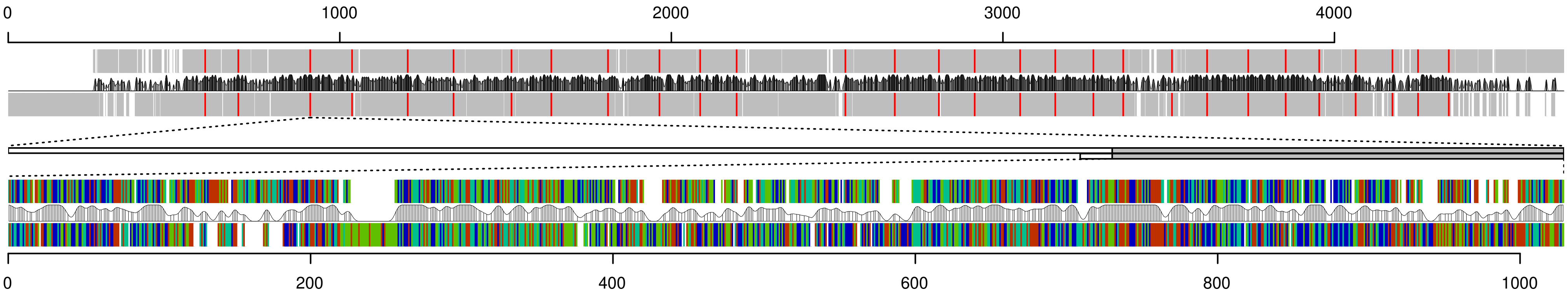

Danio rerio (ENSDART00000042134), Pygocentrus nattereri (ENSPNAT00000009097)

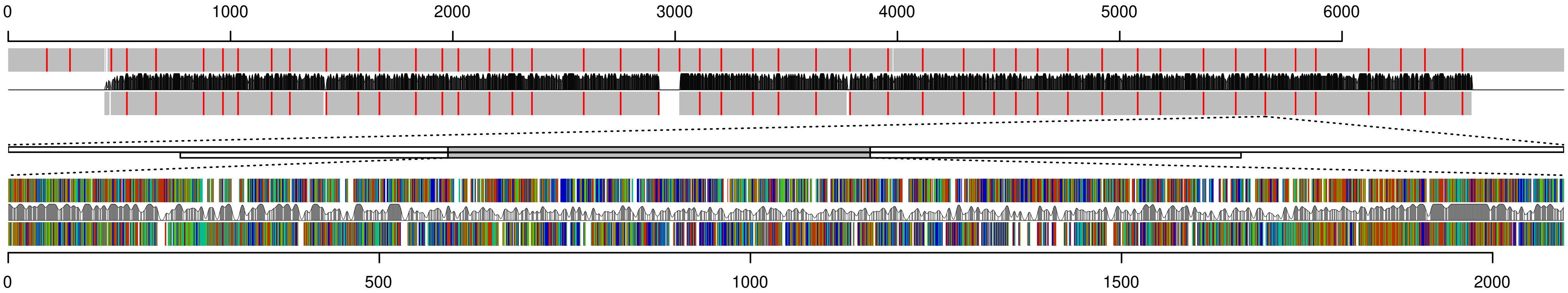

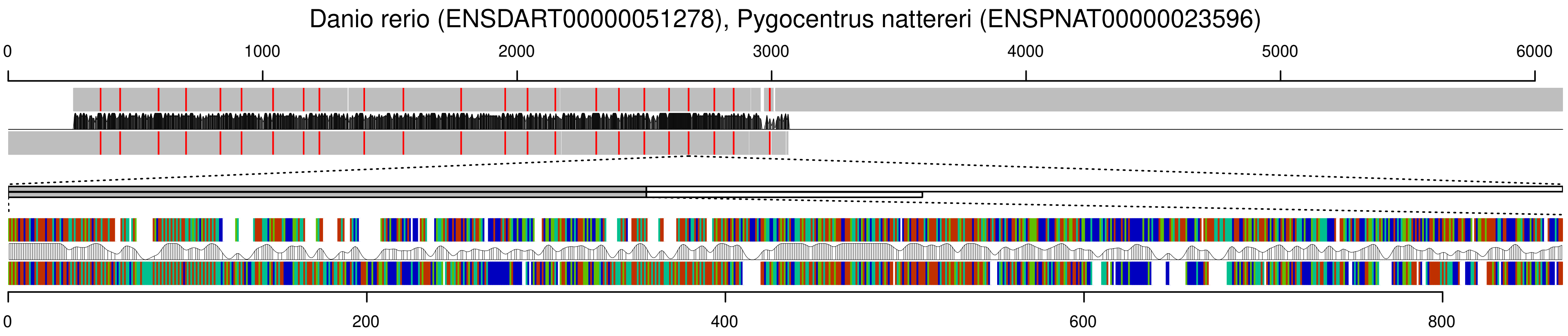
Danio rerio (ENSDART00000051278), Pygocentrus nattereri (ENSPNAT00000023596)
0 1000 2000 3000 4000 5000 6000
0 200 400 600 800

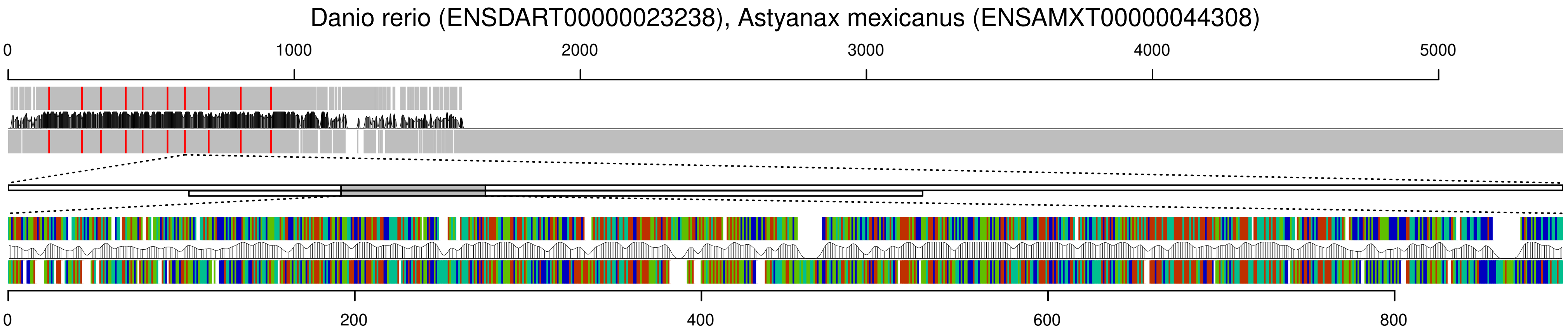
Danio rerio (ENSDART00000023238), Astyanax mexicanus (ENSAMXT00000044308)
0 1000 2000 3000 4000 5000
0 200 400 600 800

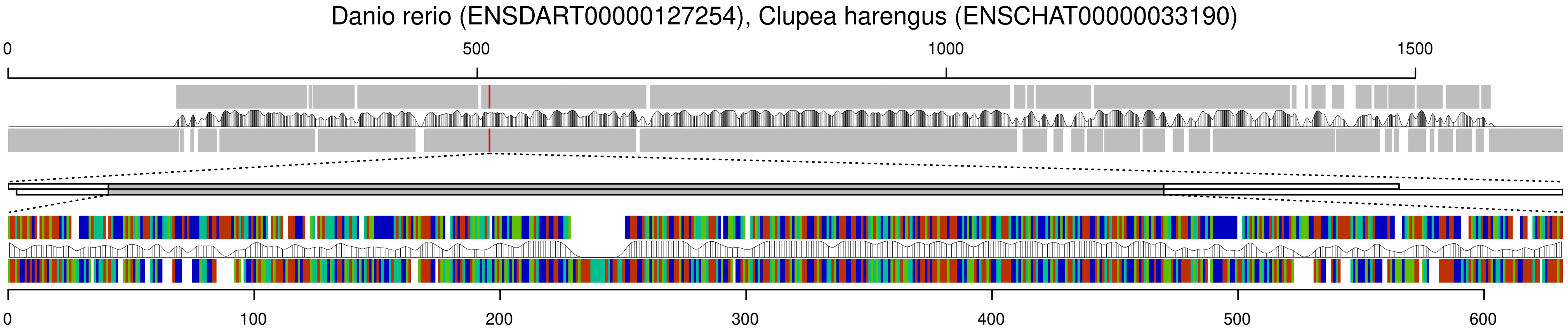
Danio rerio (ENSDART00000127254), Clupea harengus (ENSCHAT00000033190)
0 500 1000 1500
0 100 200 300 400 500 600

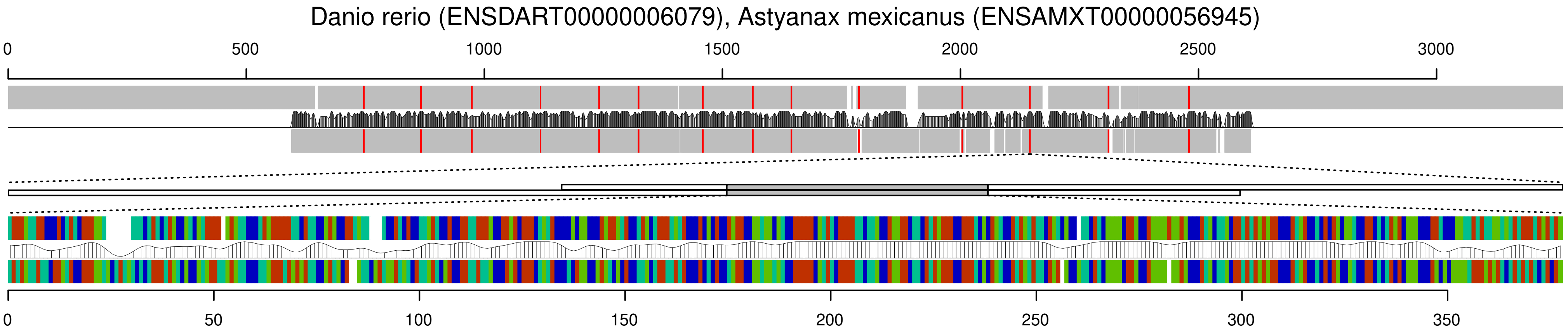
Danio rerio (ENSDART00000006079), Astyanax mexicanus (ENSAMXT00000056945)
0 500 1000 1500 2000 2500 3000
0 50 100 150 200 250 300 350

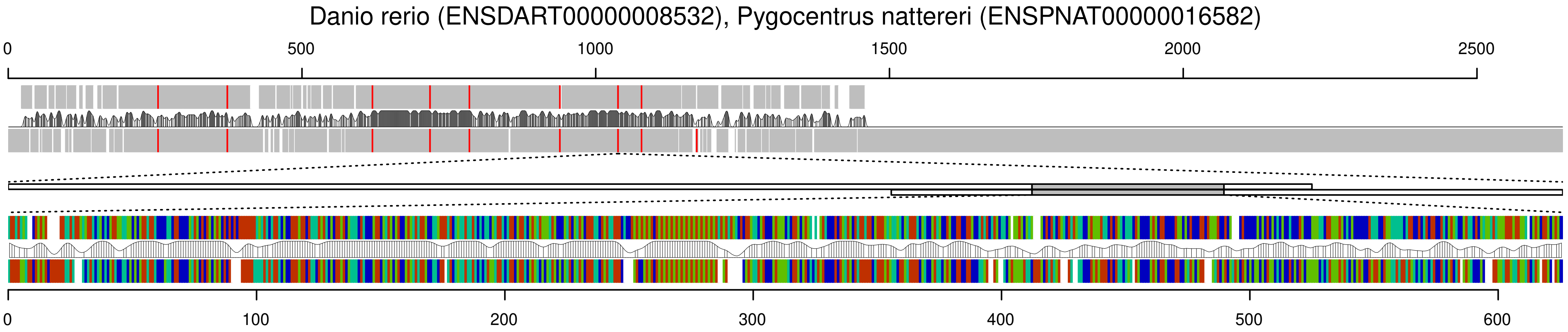
Danio rerio (ENSDART00000008532), Pygocentrus nattereri (ENSPNAT00000016582)
0 500 1000 1500 2000 2500
0 100 200 300 400 500 600

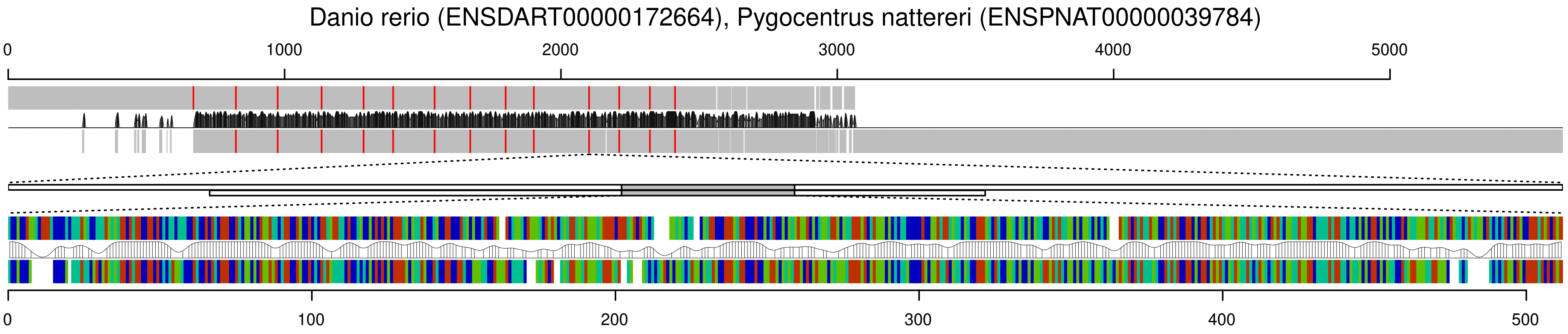
Danio rerio (ENSDART00000172664), Pygocentrus nattereri (ENSPNAT00000039784)
0 1000 2000 3000 4000 5000
0 100 200 300 400 500

Danio rerio (ENSDART00000164566), Astyanax mexicanus (ENSAMXT00000038376)

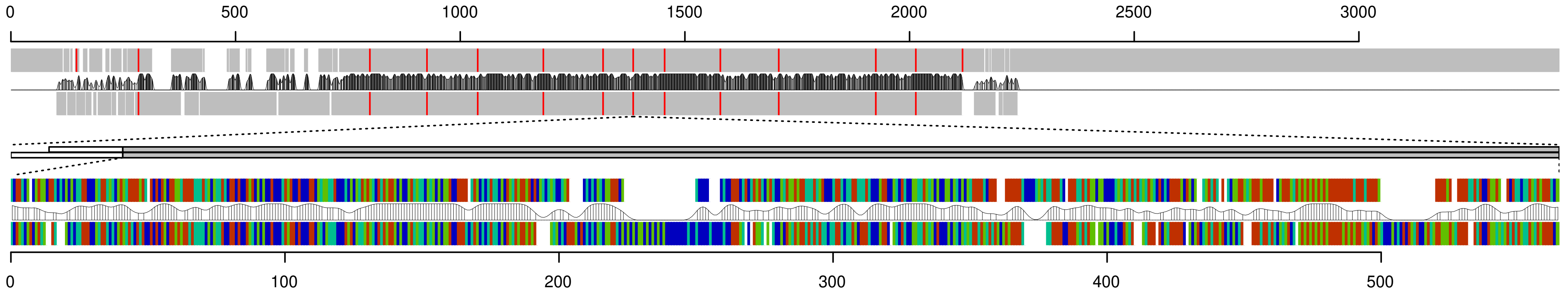

Danio rerio (ENSDART00000017185), Electrophorus electricus (ENSEEET00000024827)

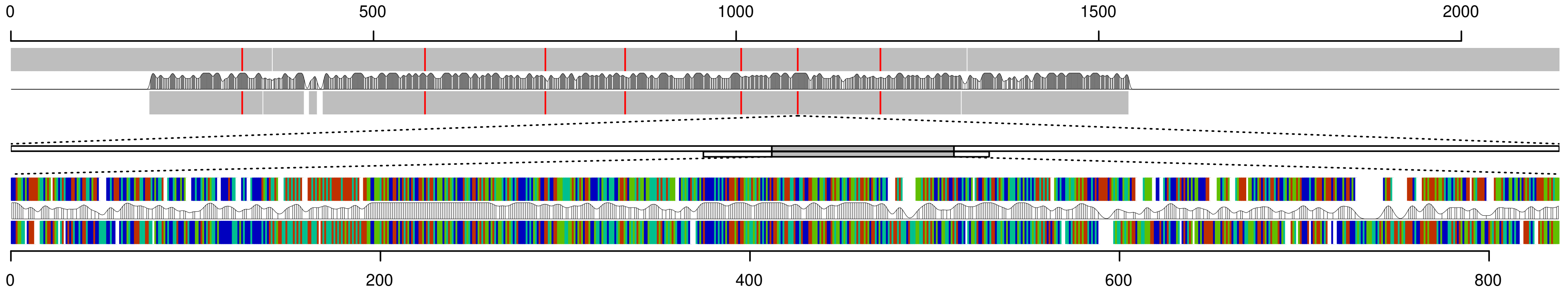

Danio rerio (ENSDART00000076496), Pygocentrus nattereri (ENSPNAT00000035790)

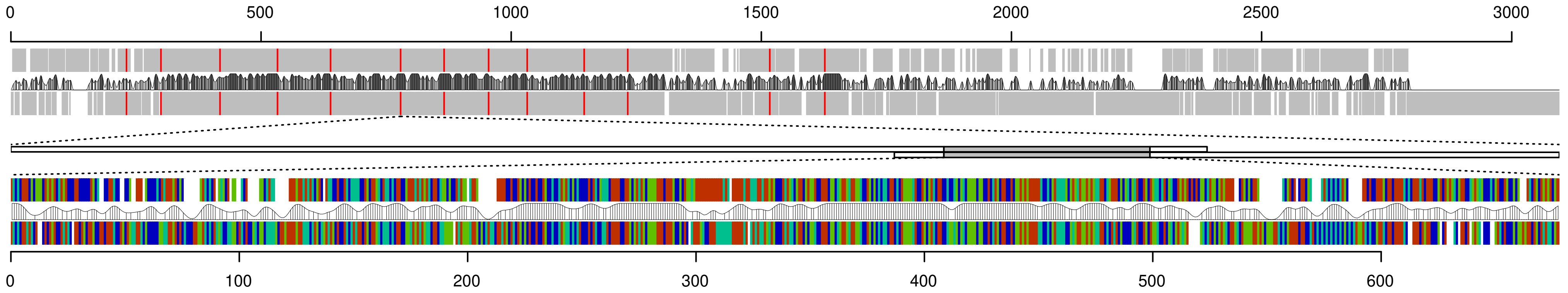

Danio rerio (ENSDART00000005562), Pygocentrus nattereri (ENSPNAT00000032010)

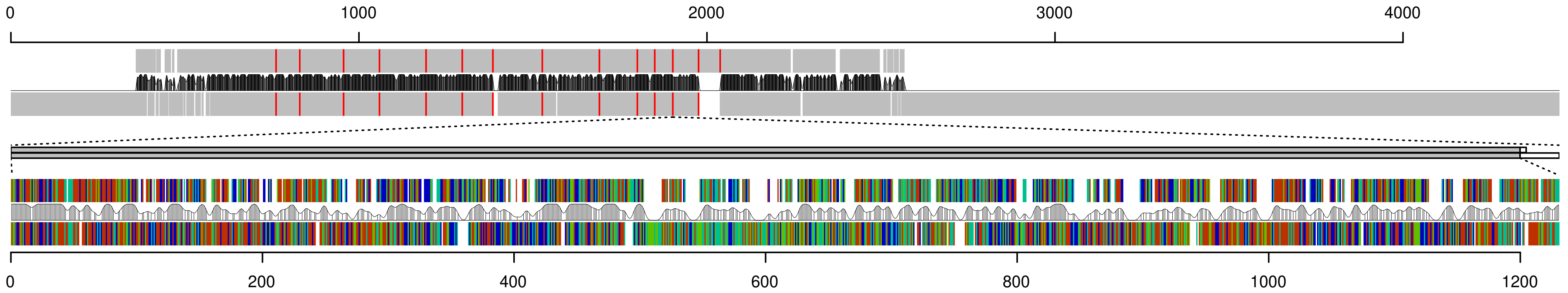

Danio rerio (ENSDART00000128690), Pygocentrus nattereri (ENSPNAT00000017498)

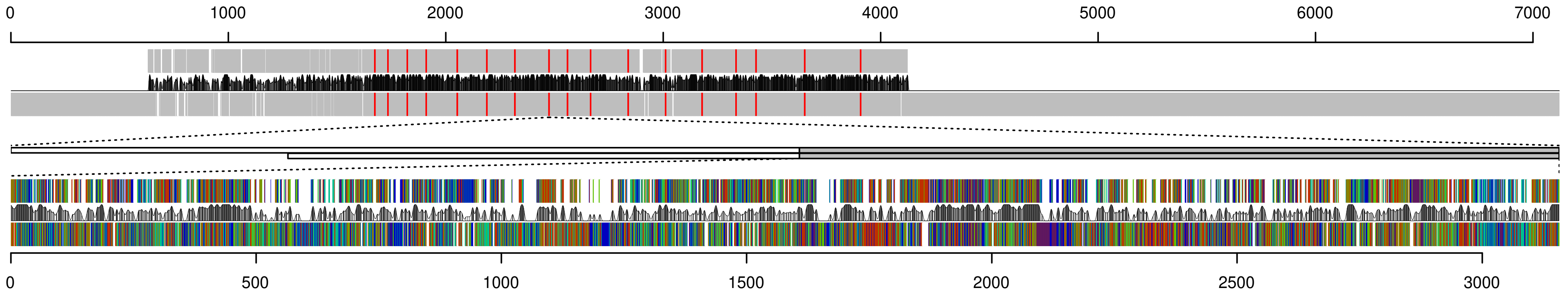

Danio rerio (ENSDART00000155432), Astyanax mexicanus (ENSAMXT00000046096)

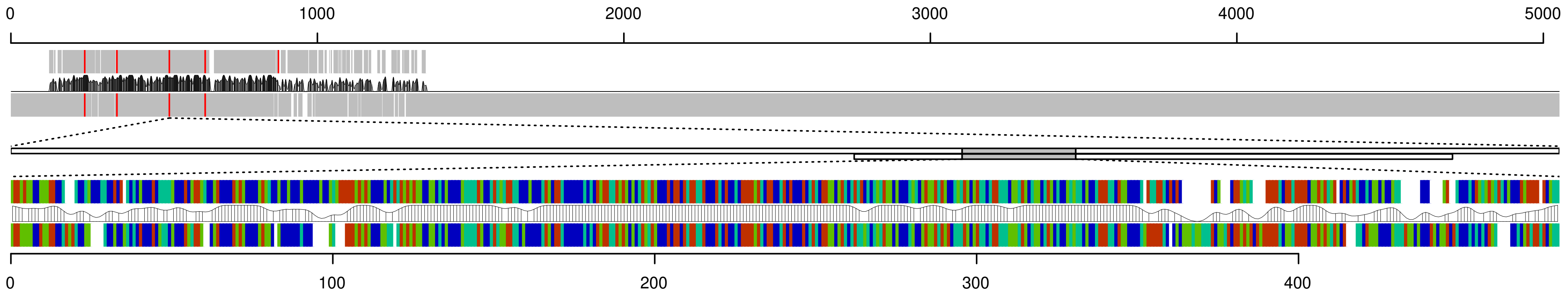

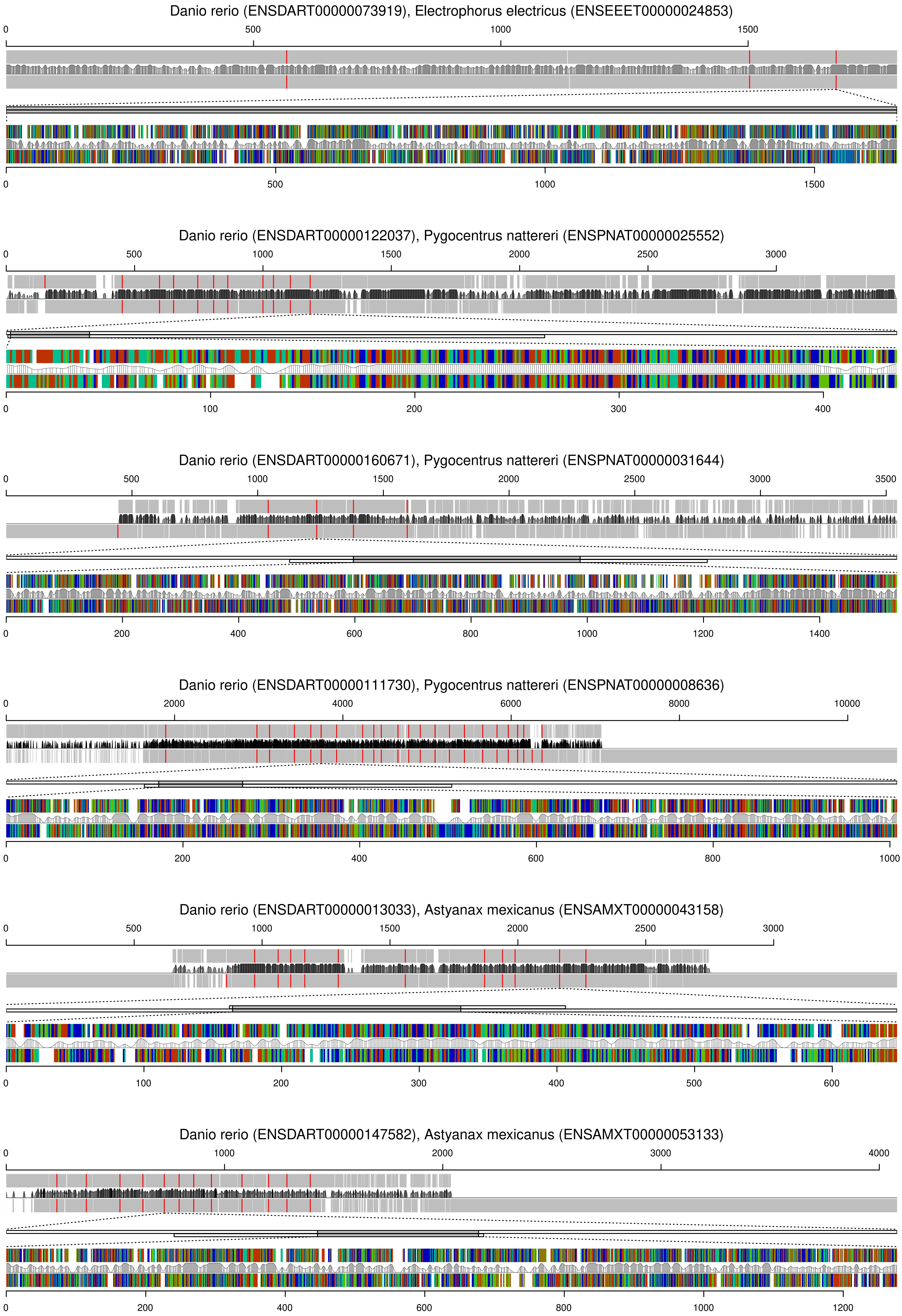
Danio rerio (ENSDART00000073919), Electrophorus electricus (ENSEEET00000024853)
0 500 1000 1500
0 500 1000 1500
Danio rerio (ENSDART00000122037), Pygocentrus nattereri (ENSPNAT00000025552)
0 500 1000 1500 2000 2500 3000
0 100 200 300 400
Danio rerio (ENSDART00000160671), Pygocentrus nattereri (ENSPNAT00000031644)
0 500 1000 1500 2000 2500 3000 3500
0 200 400 600 800 1000 1200 1400
Danio rerio (ENSDART00000111730), Pygocentrus nattereri (ENSPNAT00000008636)
0 2000 4000 6000 8000 10000
0 200 400 600 800 1000
Danio rerio (ENSDART00000013033), Astyanax mexicanus (ENSAMXT00000043158)
0 500 1000 1500 2000 2500 3000
0 100 200 300 400 500 600
Danio rerio (ENSDART00000147582), Astyanax mexicanus (ENSAMXT00000053133)
0 1000 2000 3000 4000
0 200 400 600 800 1000 1200

Danio rerio (ENSDART00000086902), Electrophorus electricus (ENSEEET00000047266)

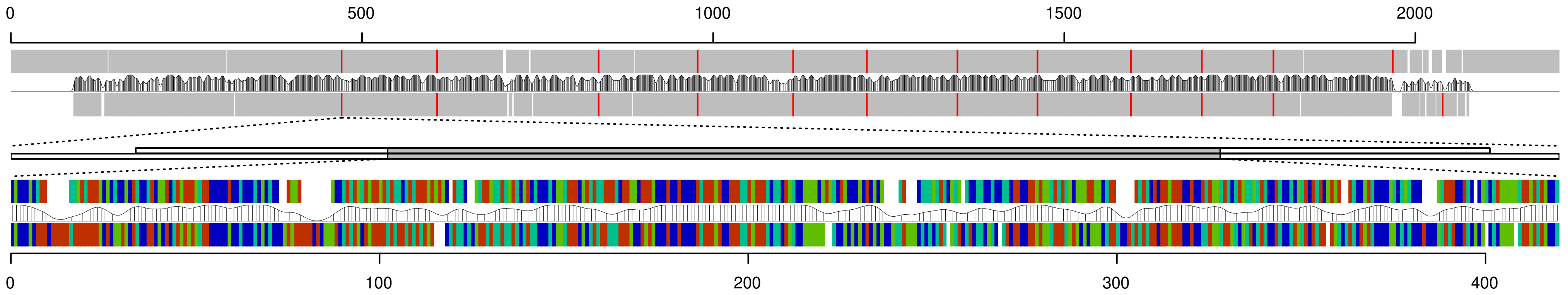

Danio rerio (ENSDART00000008532), Ictalurus punctatus (ENSIPUT00000036752)

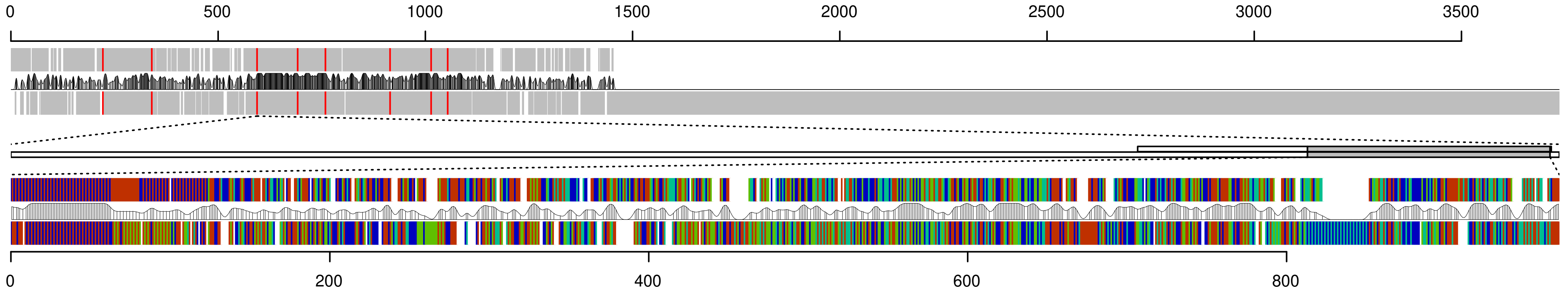

Danio rerio (ENSDART00000113030), Pygocentrus nattereri (ENSPNAT00000041147)

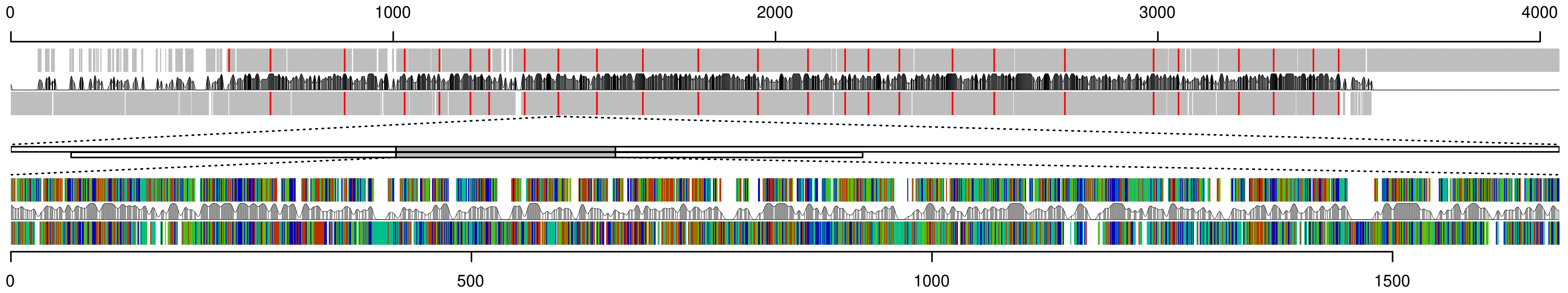

Danio rerio (ENSDART00000085009), Pygocentrus nattereri (ENSPNAT00000017688)

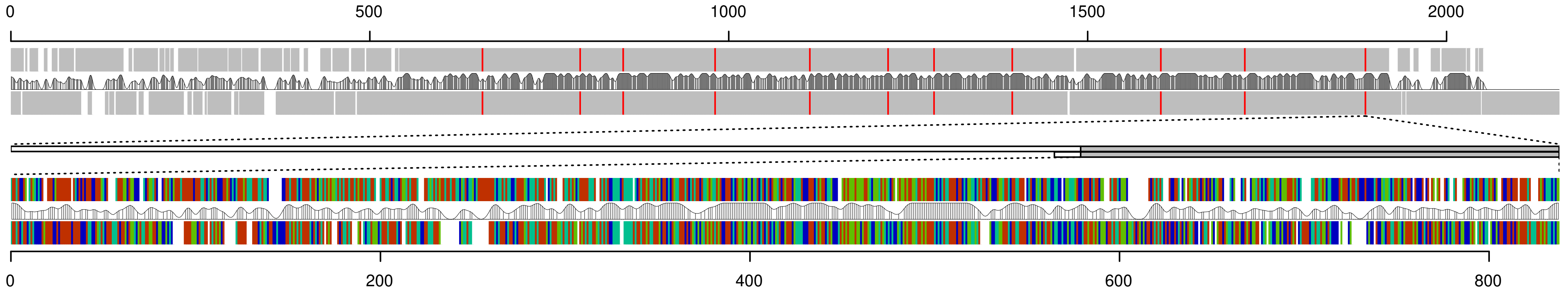

Danio rerio (ENSDART00000162838), Pygocentrus nattereri (ENSPNAT00000022013)

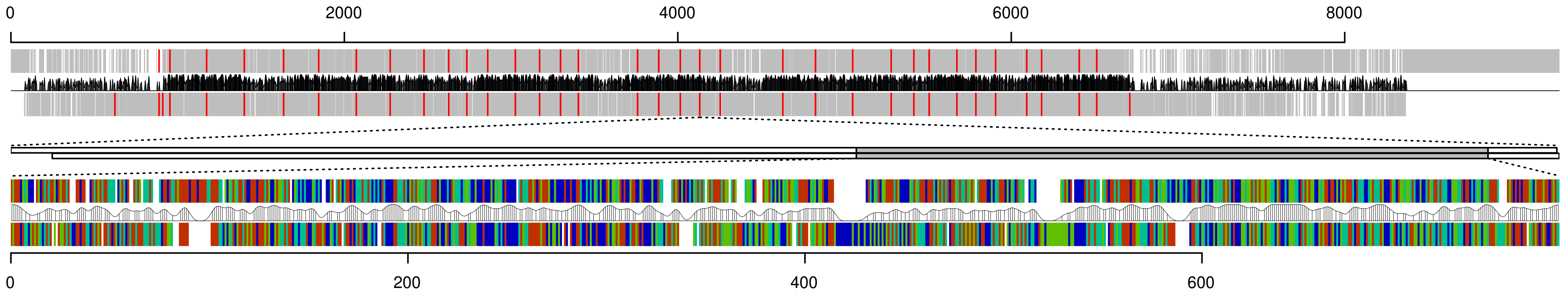

Danio rerio (ENSDART00000155020), Pygocentrus nattereri (ENSPNAT00000039454)

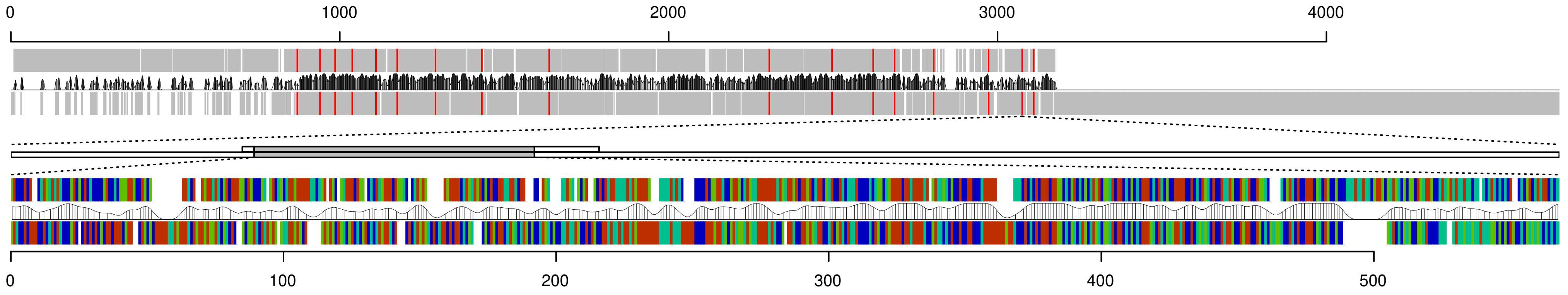

Danio rerio (ENSDART00000127214), Pygocentrus nattereri (ENSPNAT00000003048)

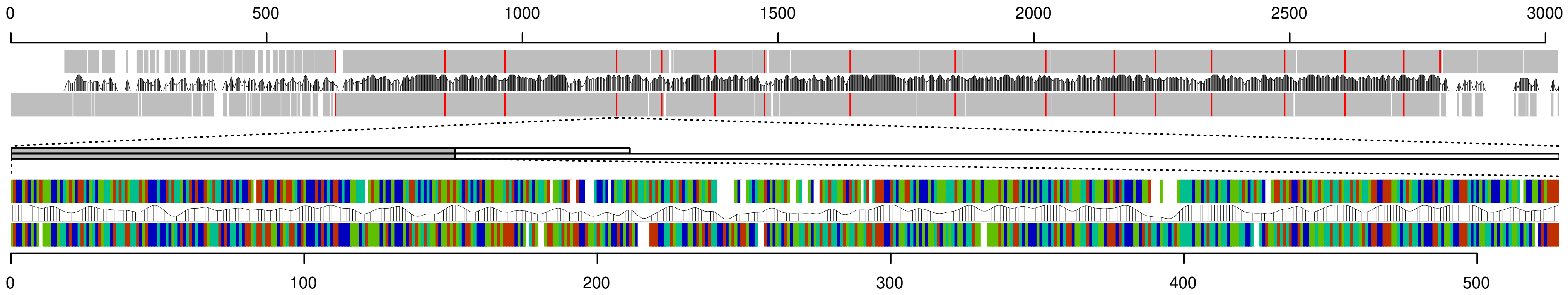

Danio rerio (ENSDART00000156000), Pygocentrus nattereri (ENSPNAT00000039723)

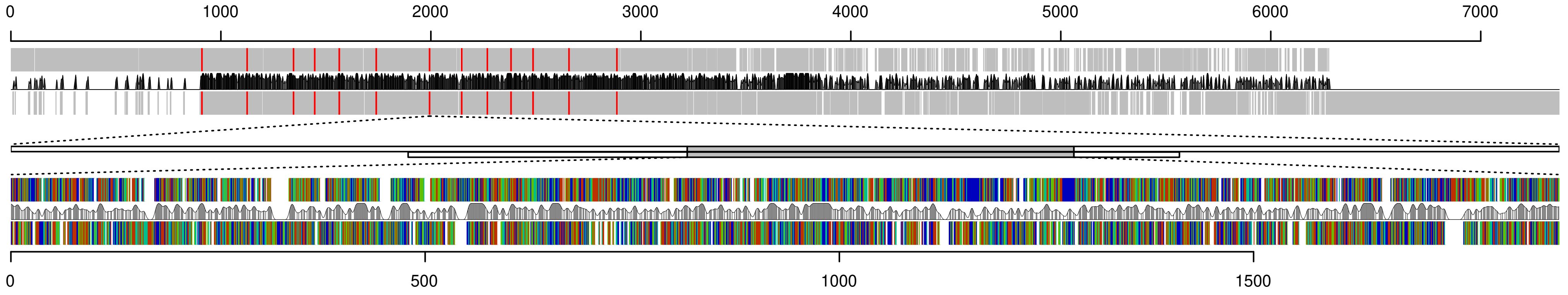

Danio rerio (ENSDART00000077435), Electrophorus electricus (ENSEEET00000033698)

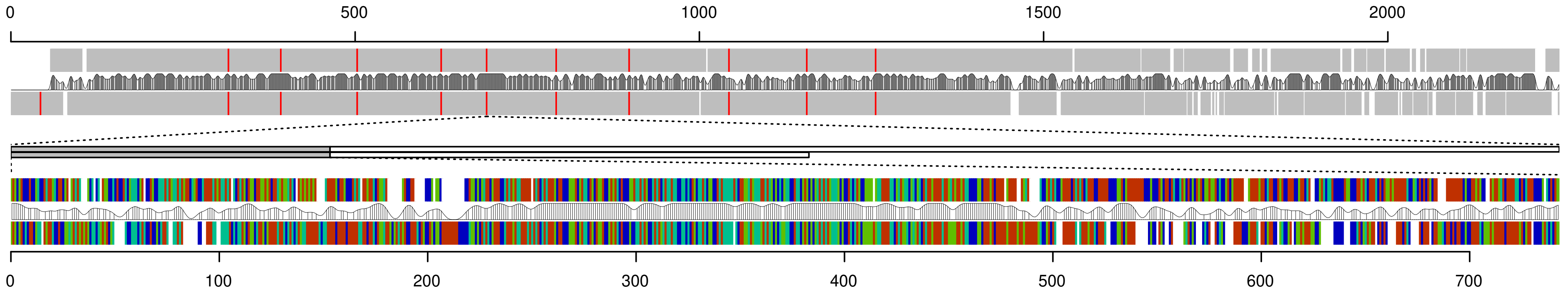

Danio rerio (ENSDART00000051271), Pygocentrus nattereri (ENSPNAT00000023573)

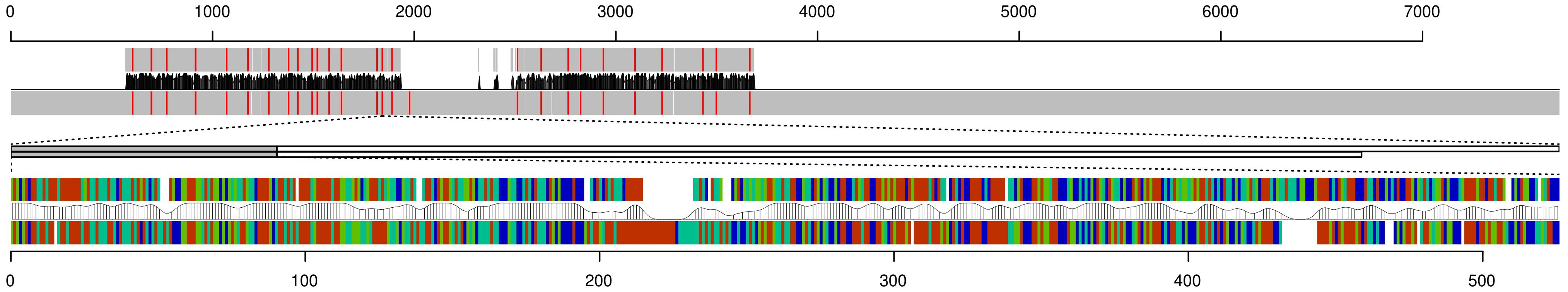

Danio rerio (ENSDART00000134735), Pygocentrus nattereri (ENSPNAT00000020552)

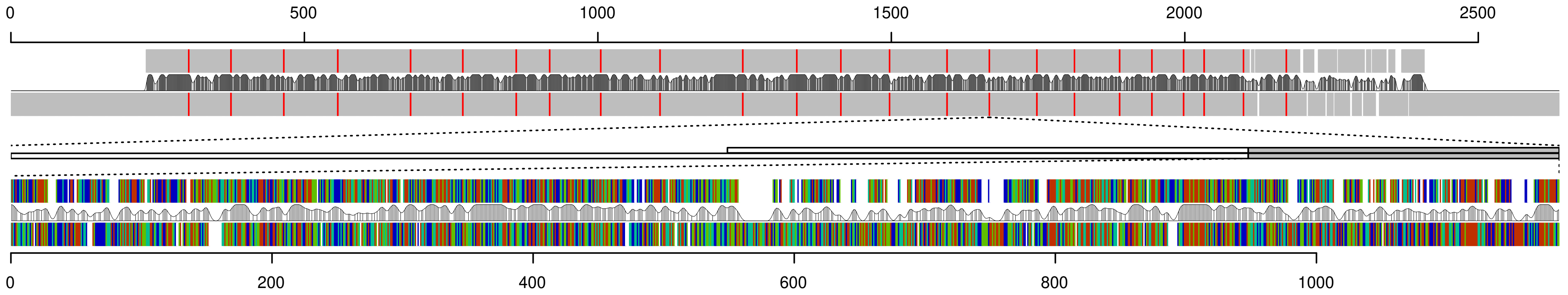

Danio rerio (ENSDART00000171324), Pygocentrus nattereri (ENSPNAT00000011594)

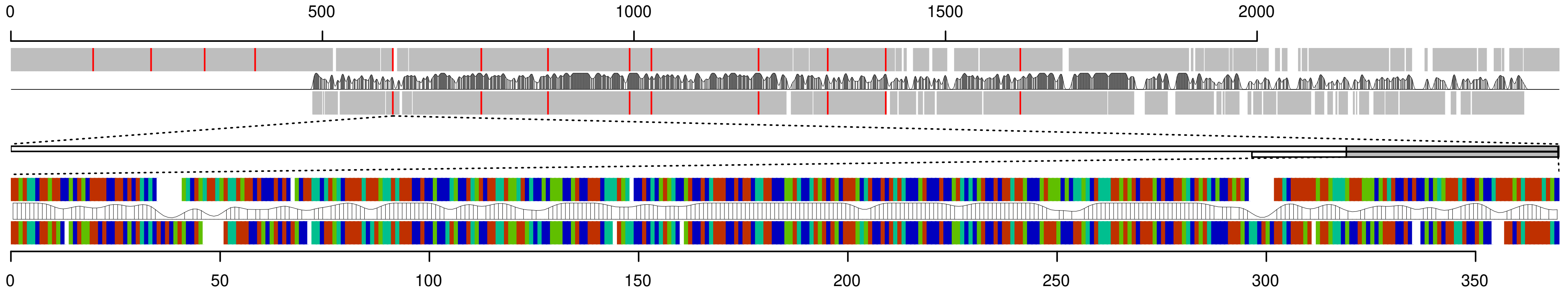

Supplement: Supplementary file 6 — Transcript and intron alignments for points in Fig. S14. Each panel shows the maximally scoring alignment between D. rerio and teleost intron orthologues (lower) and transcript alignment (upper) used to establish the intron orthology. Grey, white and red parts indicate aligned exonic sequence, gaps and positions of intron meta-characters respectively. Colours in intron alignment represent bases (A blue, C cyan, G green, T brown, N grey, gap white). Curves lying between sequence representations show a normal kernel density smoothed estimate of local similarity (9 bp window, standard deviation two); vertical lines indicate matches. Region between exon and intron alignments indicates the location of the maximally scoring alignment in the introns. Upper sequence D. rerio. Files 6–10 and 11–15 contain alignments to teleost and mammalian sequences respectively. Each file corresponds to one panel in Fig. S17 and to one specific teleost size class: Files 6,11: long (E,J), 7,12: medium (D,I), 8,13: short.2 (C,H), 9,14 short (B, G) and 10,15 ctl (A,F). [file 12864_2022_8760_MOESM6_ESM.zip › 12864_2022_8760_MOESM7_ESM.pdf]
