# Supplementary material for: Intron size minimisation in teleosts
Source: BMC Genomics. 2022 Sep 1;23:628. doi: 10.1186/s12864-022-08760-w (PMC9438311; doi:10.1186/s12864-022-08760-w)

Danio rerio (ENSDART00000010378), Pygocentrus nattereri (ENSPNAT00000021172)

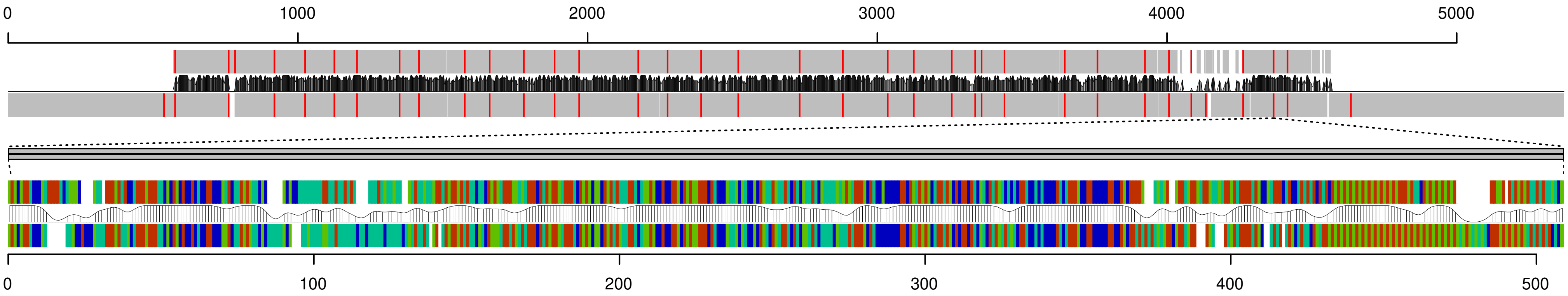

Danio rerio (ENSDART00000085009), Pygocentrus nattereri (ENSPNAT00000017688)

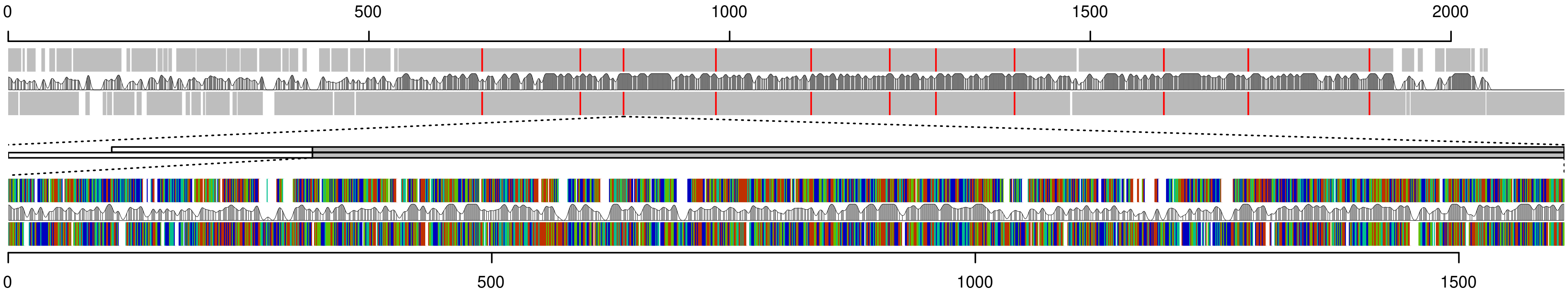

Danio rerio (ENSDART00000134809), Pygocentrus nattereri (ENSPNAT00000013103)

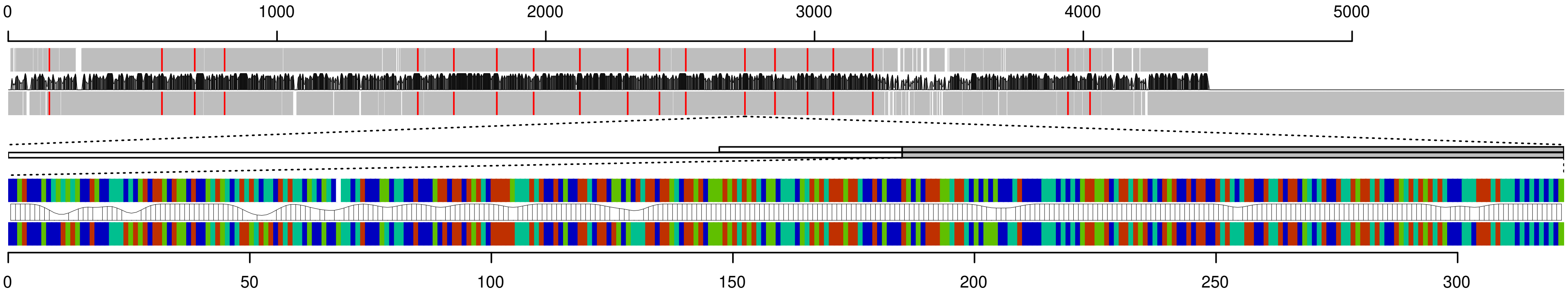

Danio rerio (ENSDART00000170482), Astyanax mexicanus (ENSAMXT00000021131)

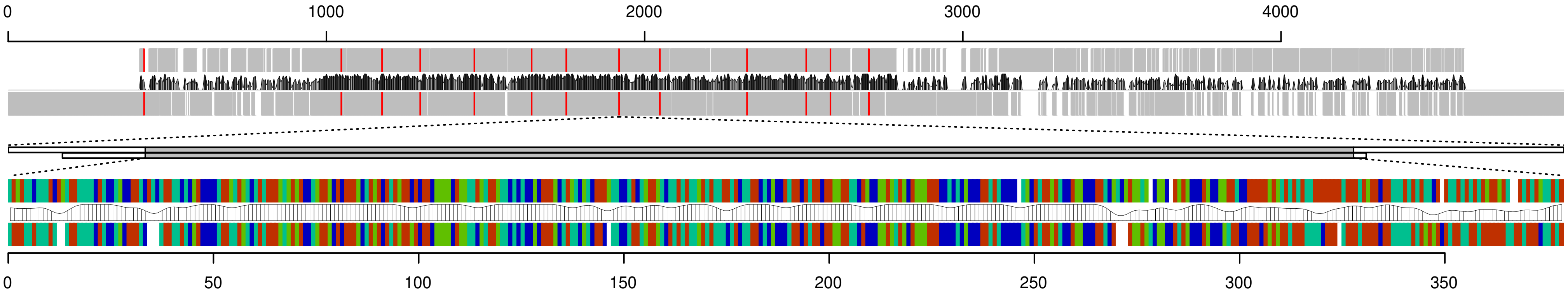

Danio rerio (ENSDART00000134547), Astyanax mexicanus (ENSAMXT00000045696)

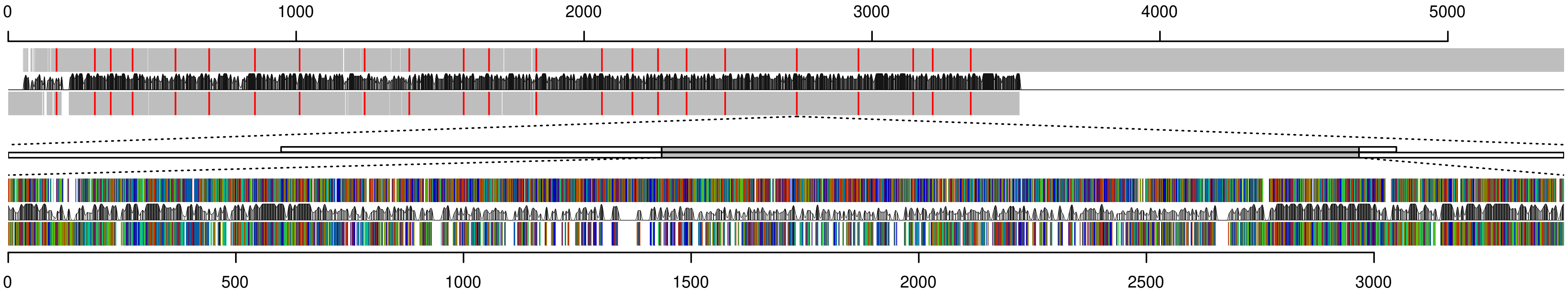

Danio rerio (ENSDART00000187248), Pygocentrus nattereri (ENSPNAT00000013019)

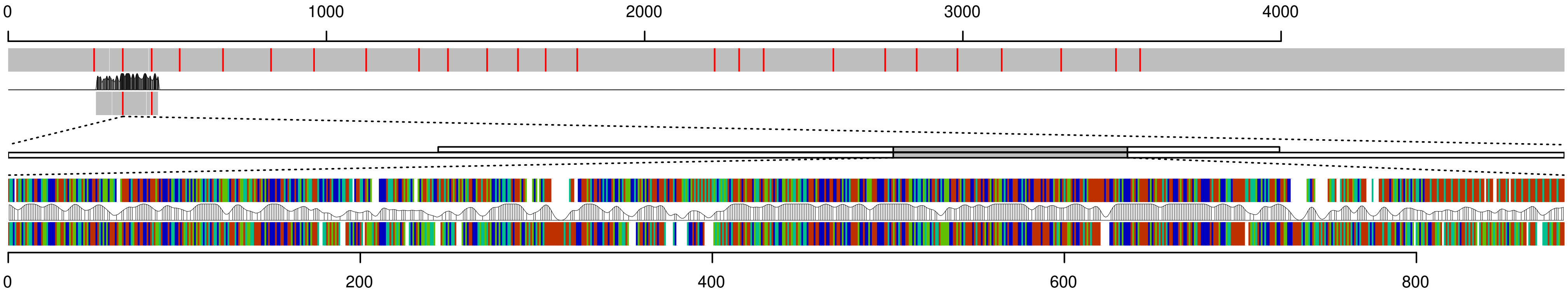

Danio rerio (ENSDART00000077304), Pygocentrus nattereri (ENSPNAT00000007963)

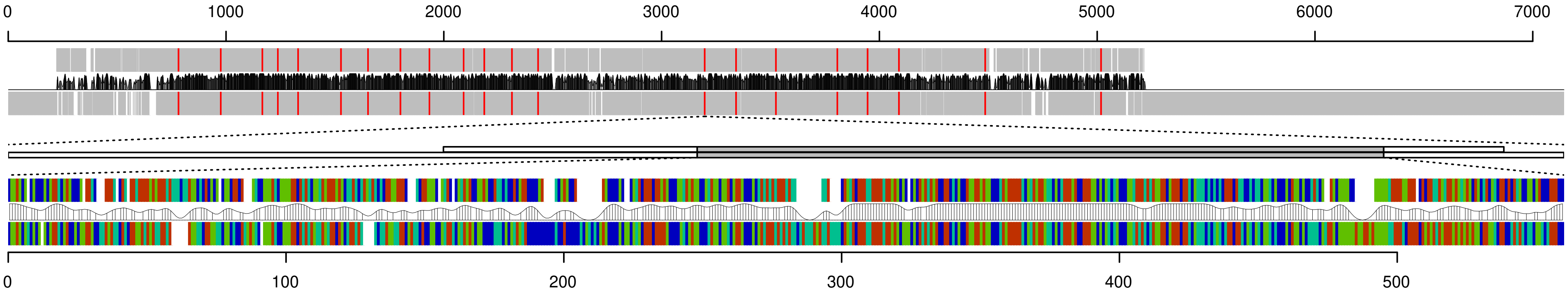

Danio rerio (ENSDART00000154660), Pygocentrus nattereri (ENSPNAT00000036076)

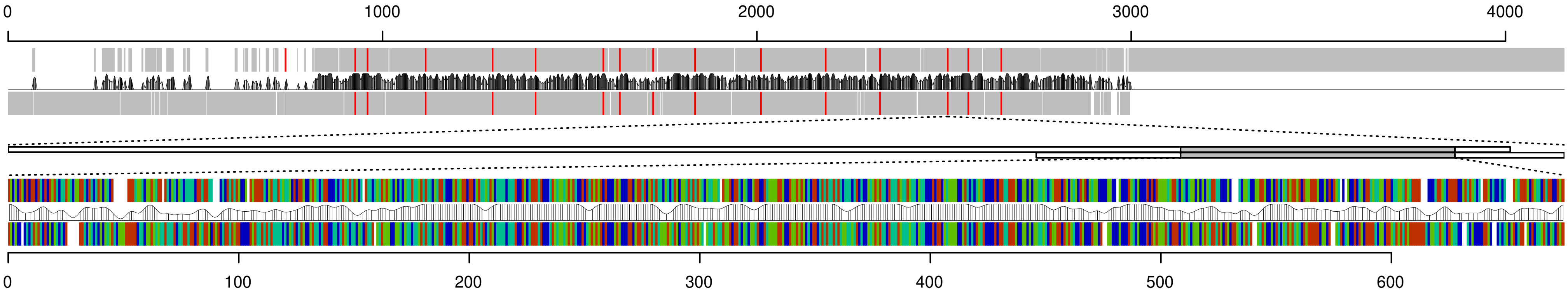

Danio rerio (ENSDART00000077304), Pygocentrus nattereri (ENSPNAT00000007963)

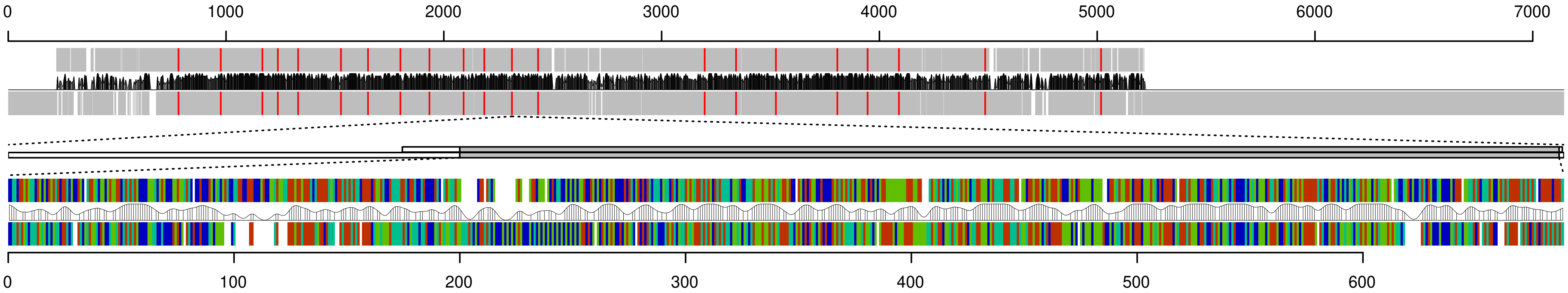

Danio rerio (ENSDART00000104496), Astyanax mexicanus (ENSAMXT00000046670)

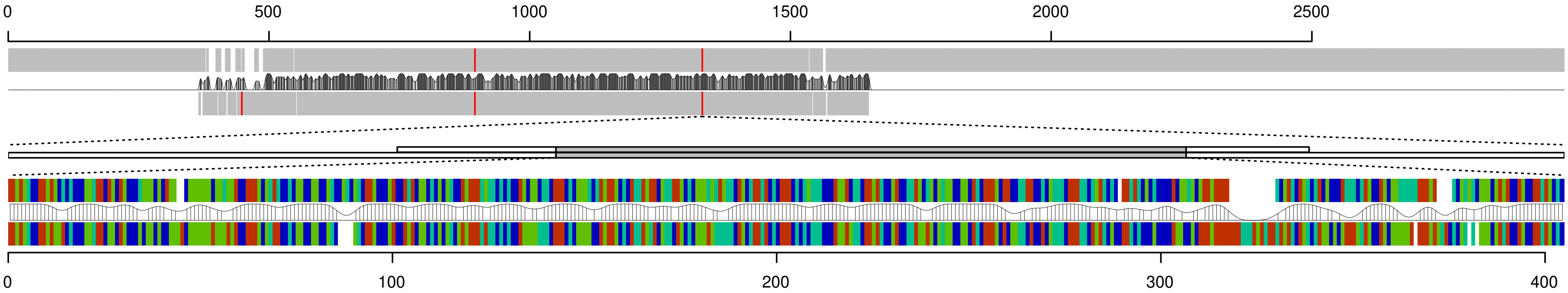

Danio rerio (ENSDART00000134121), Pygocentrus nattereri (ENSPNAT00000025000)

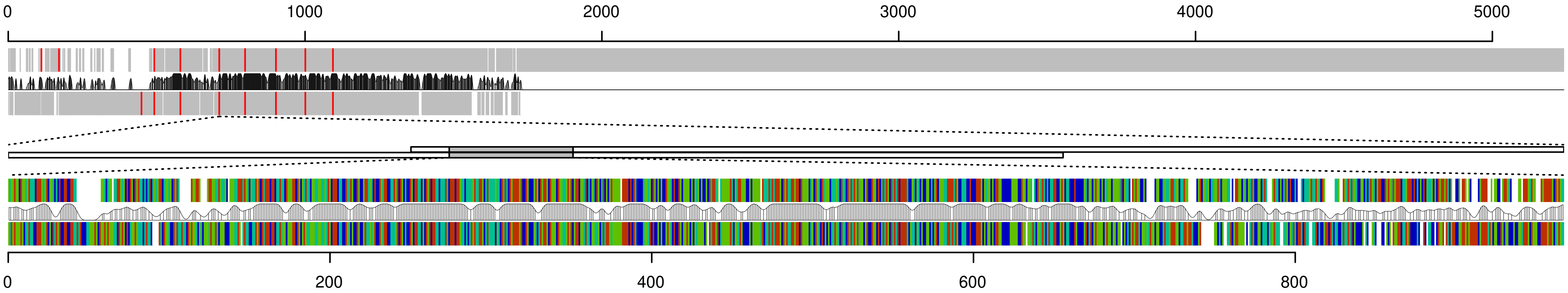

Danio rerio (ENSDART00000079165), Pygocentrus nattereri (ENSPNAT00000016274)

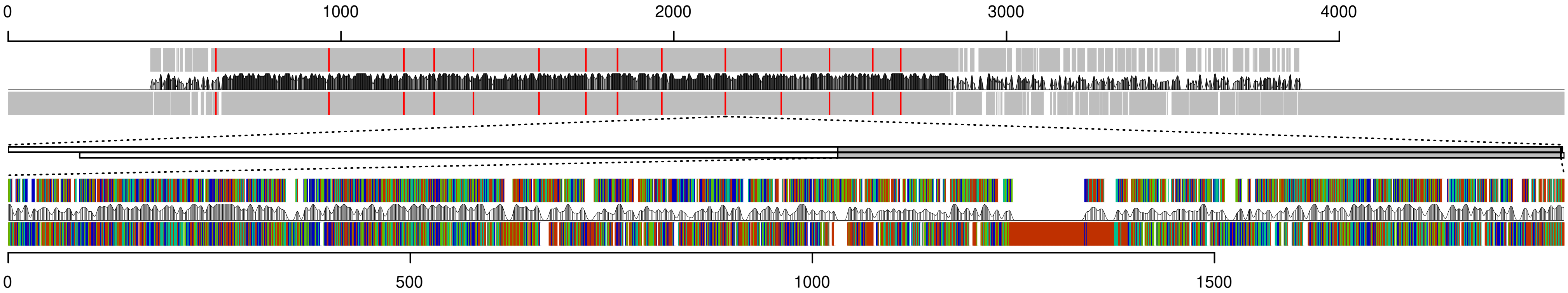

Danio rerio (ENSDART00000132405), Clupea harengus (ENSCHAT00000067485)

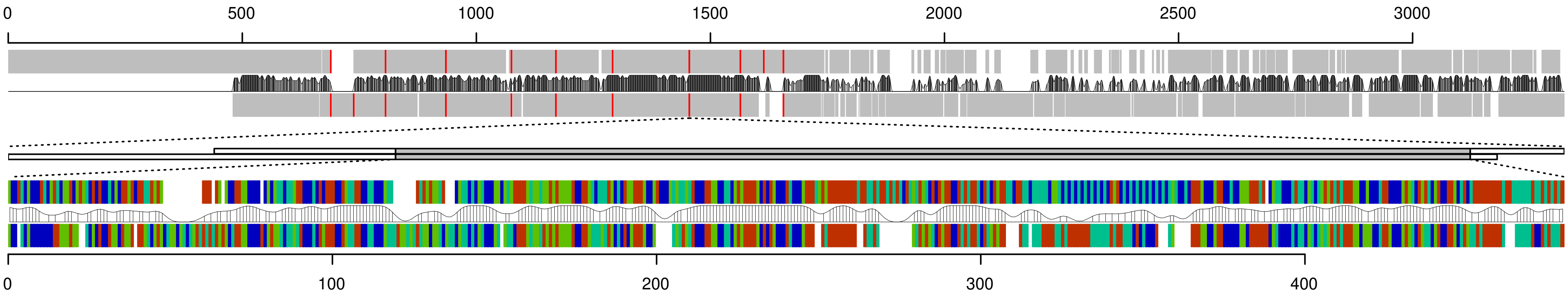

Danio rerio (ENSDART00000025031), Clupea harengus (ENSCHAT00000040962)

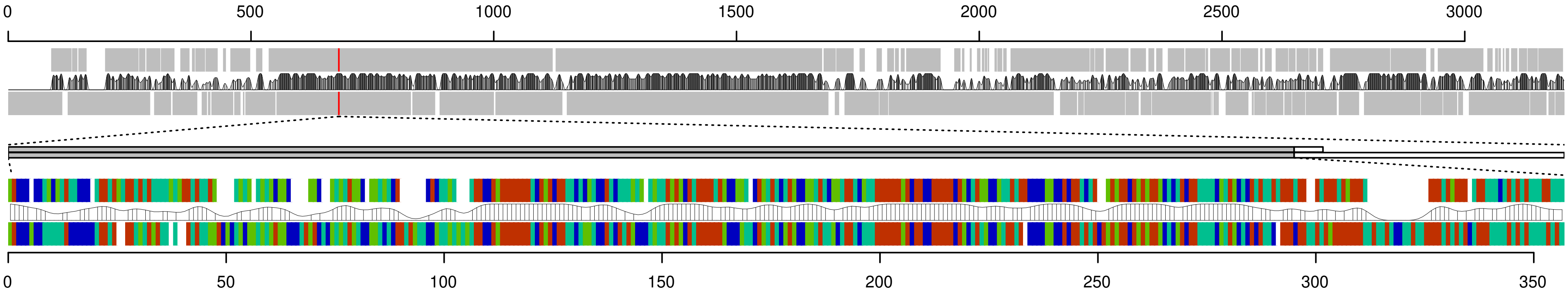

Danio rerio (ENSDART00000065255), Pygocentrus nattereri (ENSPNAT00000032870)

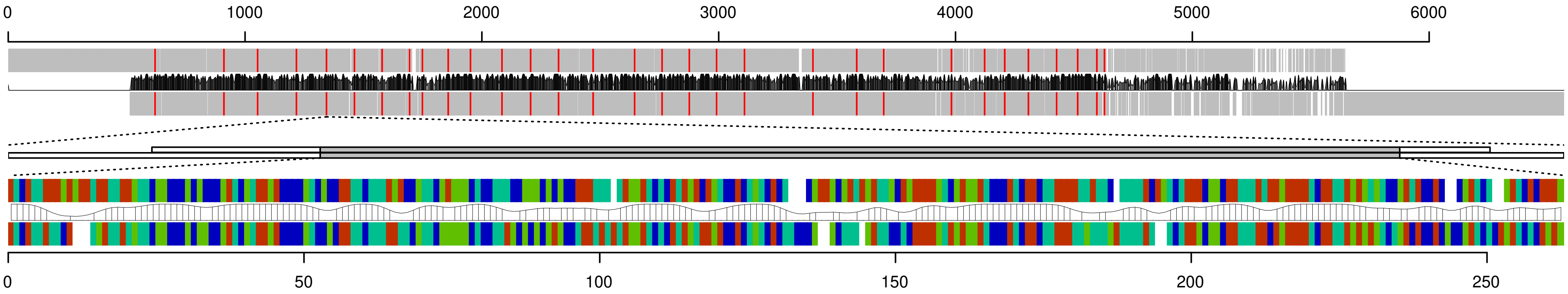

Danio rerio (ENSDART00000157621), Electrophorus electricus (ENSEEET00000012973)

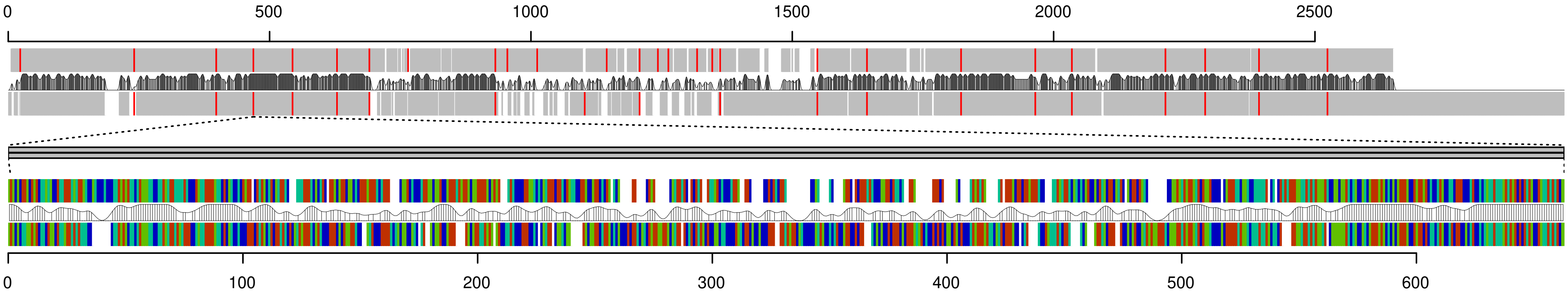

Danio rerio (ENSDART00000075730), Pygocentrus nattereri (ENSPNAT00000030232)

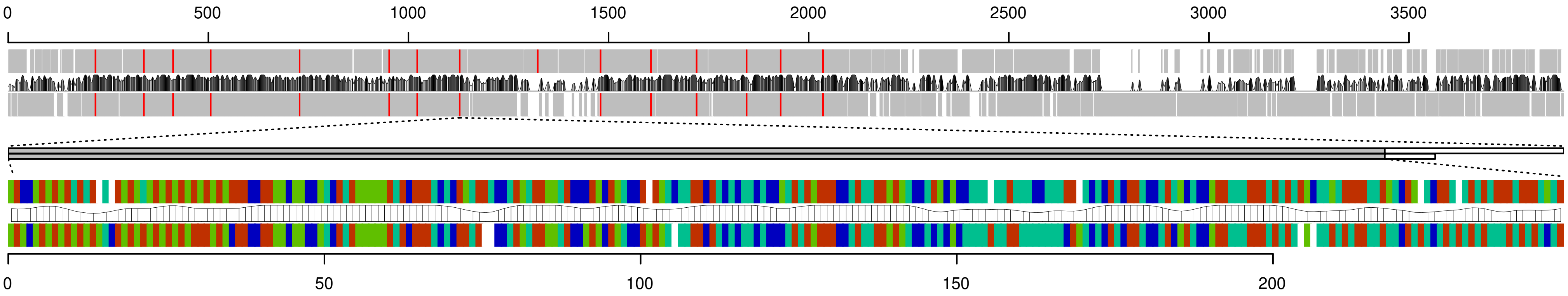

Danio rerio (ENSDART00000173789), Pygocentrus nattereri (ENSPNAT00000022531)

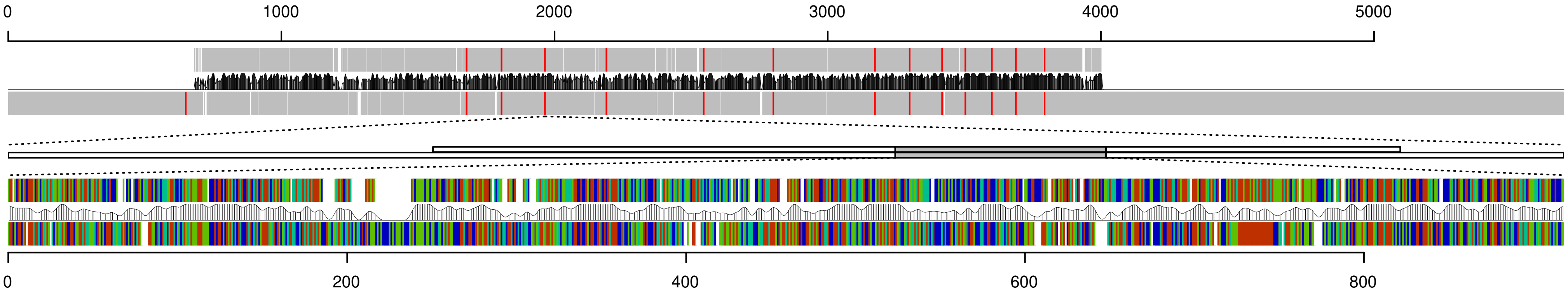

Danio rerio (ENSDART00000111856), Pygocentrus nattereri (ENSPNAT00000037894)

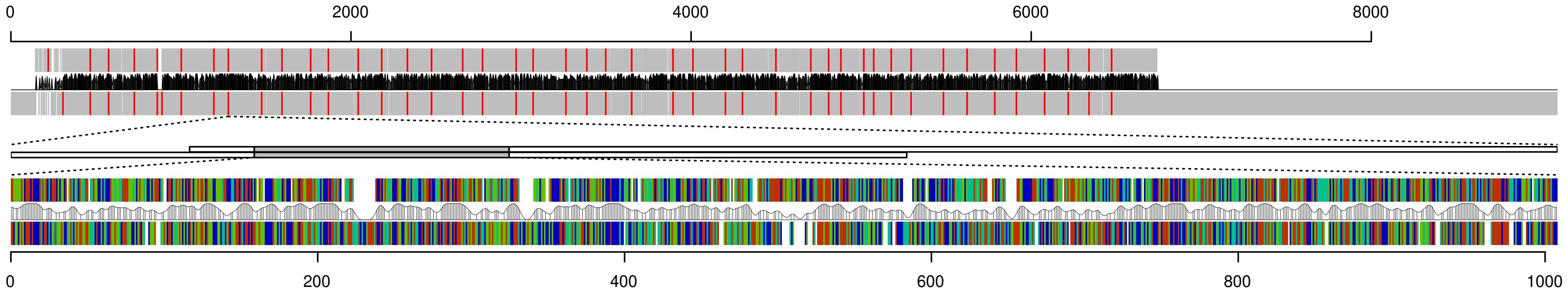

Danio rerio (ENSDART00000037516), Pygocentrus nattereri (ENSPNAT00000026474)

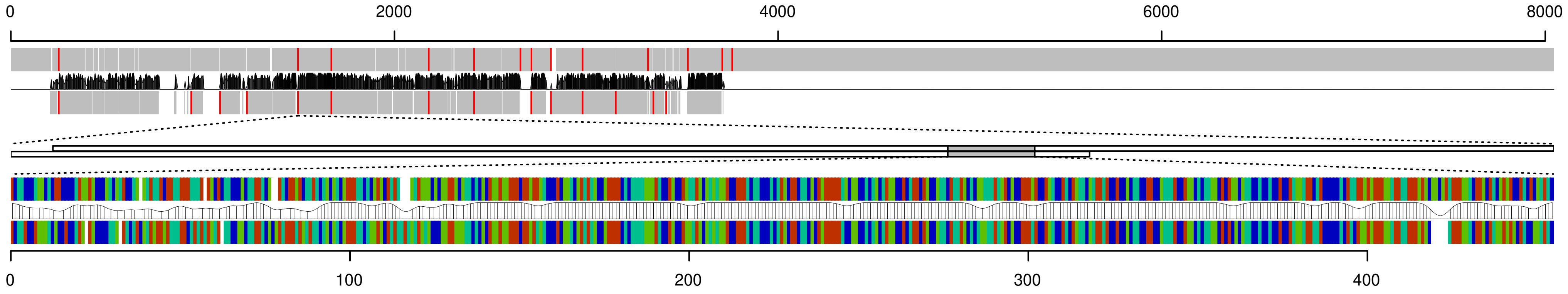

Danio rerio (ENSDART00000170482), Ictalurus punctatus (ENSIPUT00000031334)

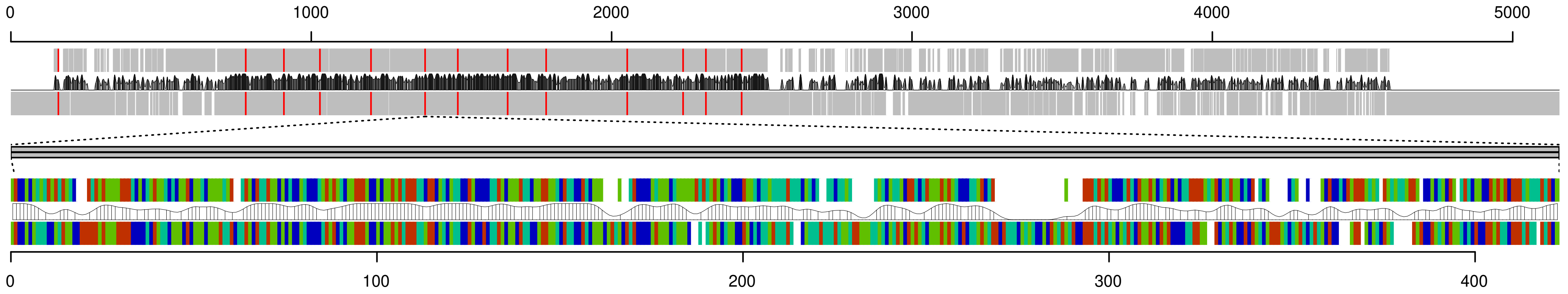

Danio rerio (ENSDART00000026635), Ictalurus punctatus (ENSIPUT00000004030)

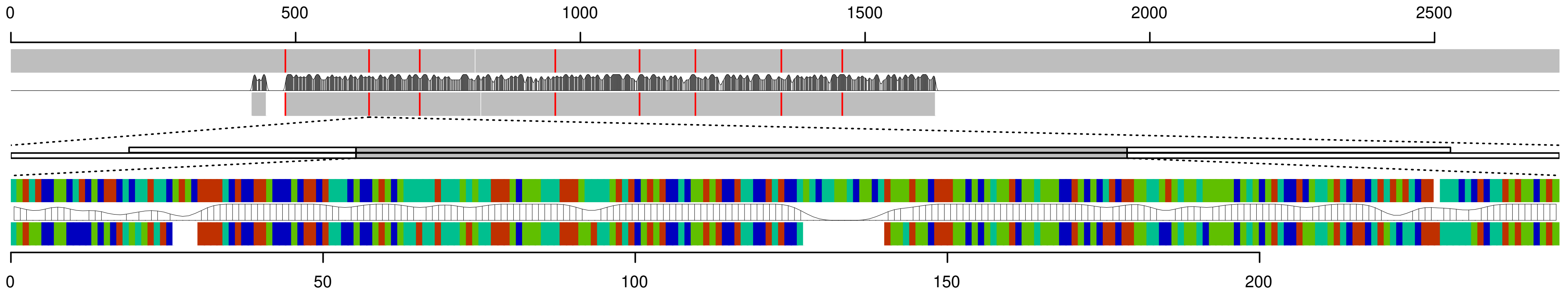

Danio rerio (ENSDART00000134564), Astyanax mexicanus (ENSAMXT00000046426)

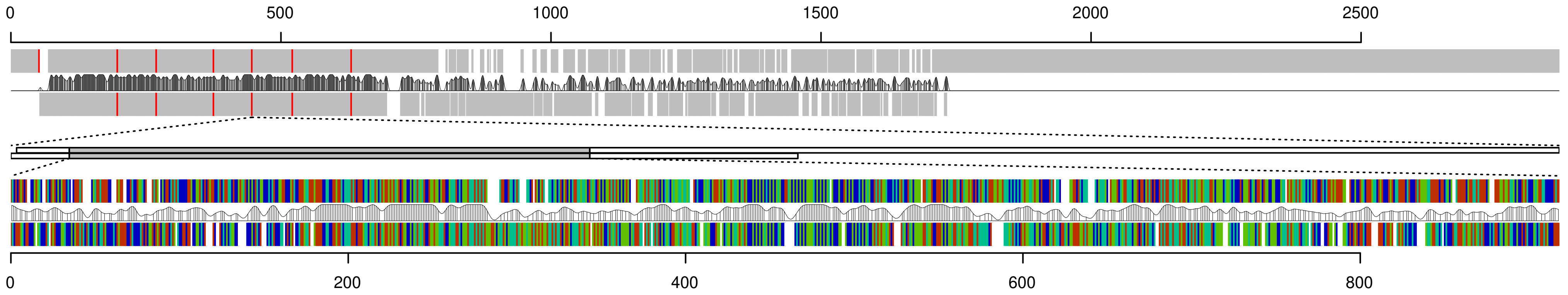

Danio rerio (ENSDART00000155112), Astyanax mexicanus (ENSAMXT00000046572)

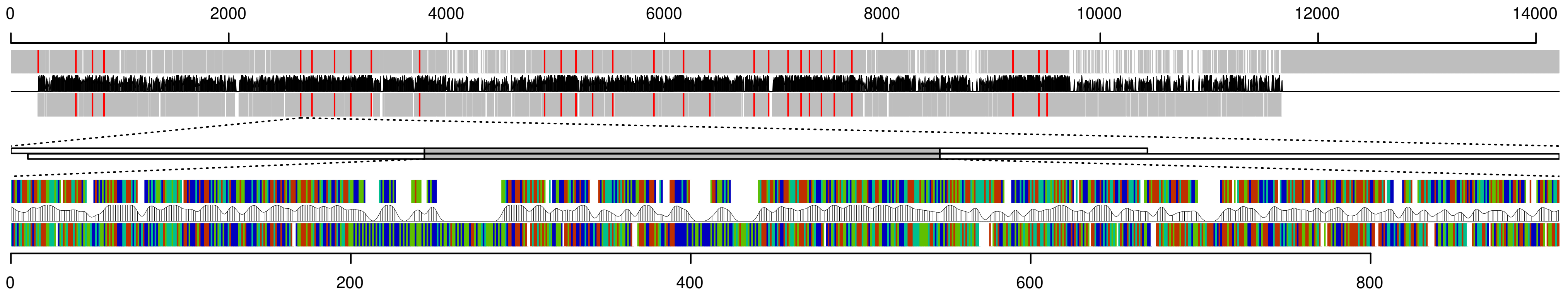

Danio rerio (ENSDART00000135049), Pygocentrus nattereri (ENSPNAT00000015012)

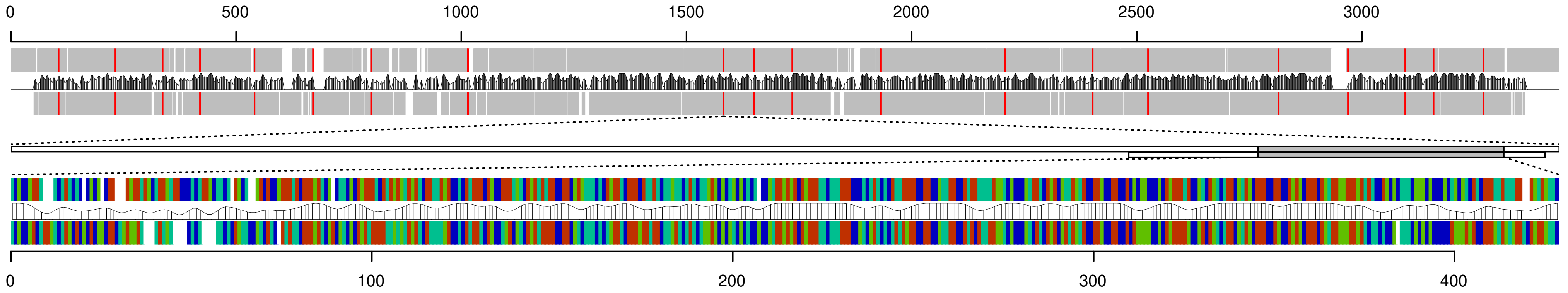

Danio rerio (ENSDART00000013033), Pygocentrus nattereri (ENSPNAT00000011329)

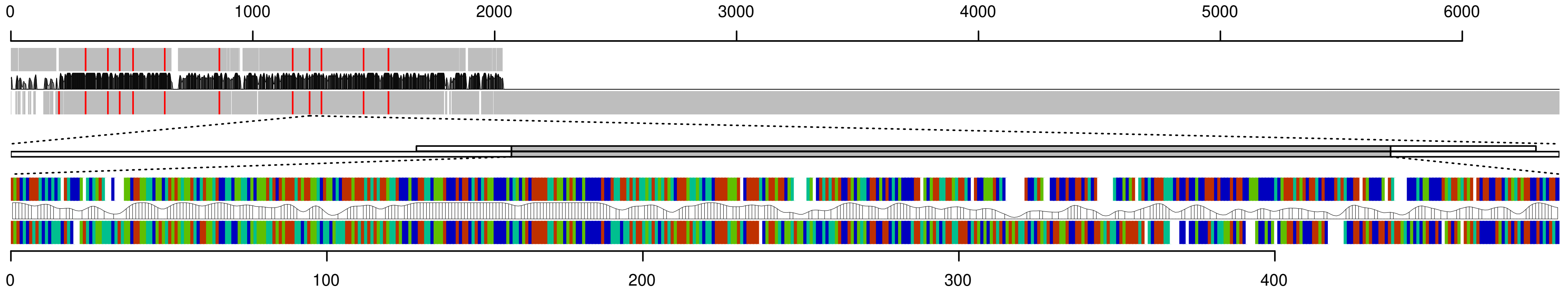

Danio rerio (ENSDART00000111537), Electrophorus electricus (ENSEEET00000048097)

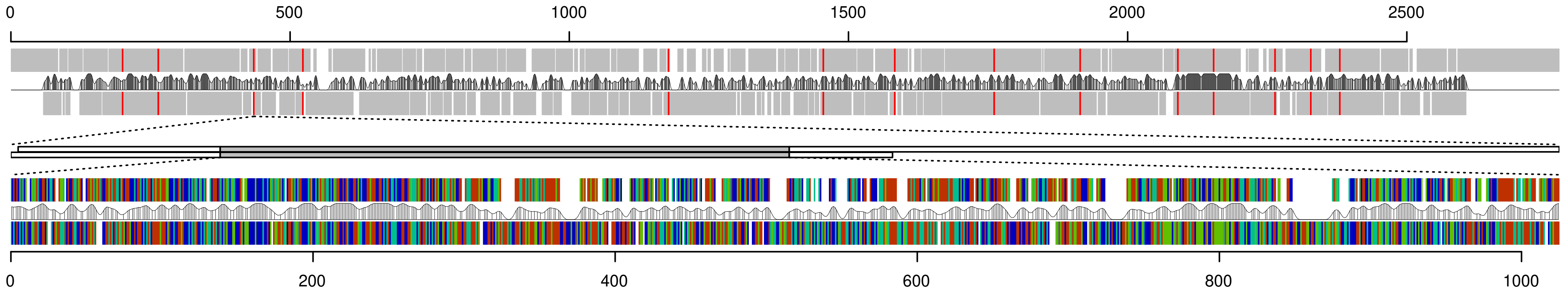

Danio rerio (ENSDART00000162359), Pygocentrus nattereri (ENSPNAT00000011108)

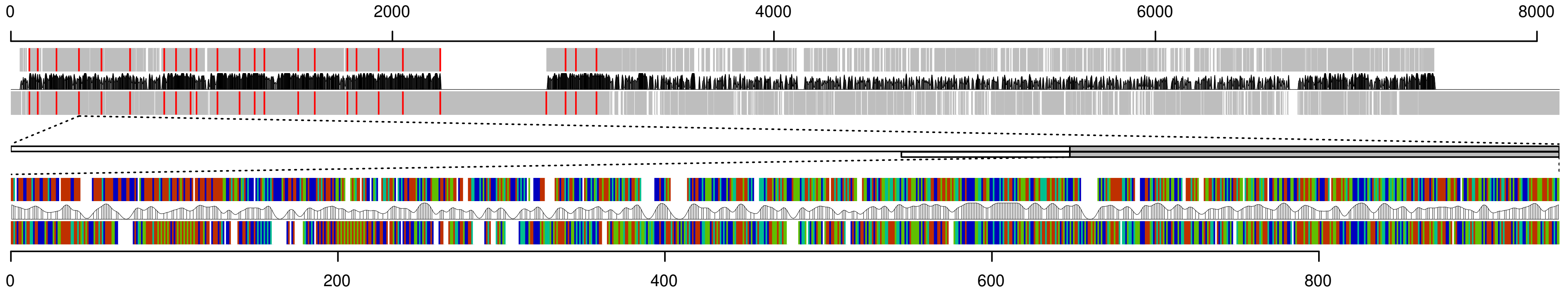

Danio rerio (ENSDART00000162359), Pygocentrus nattereri (ENSPNAT00000011108)

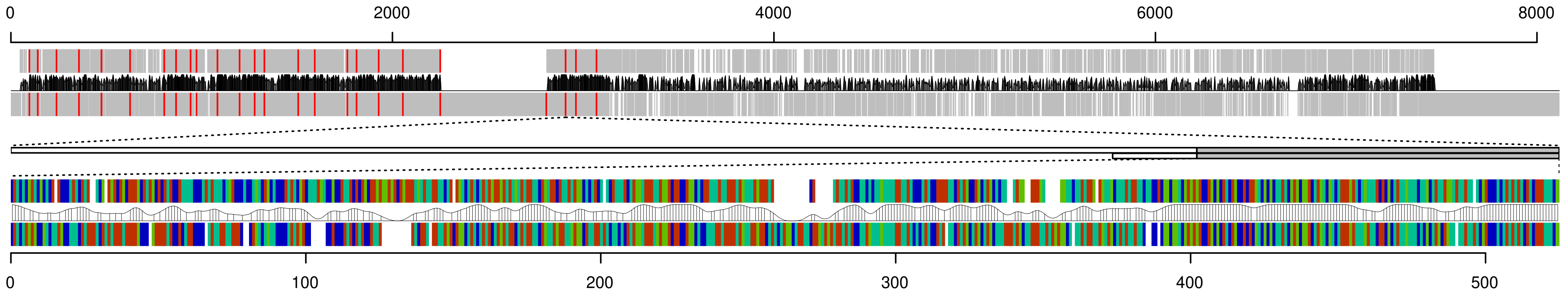

Danio rerio (ENSDART00000142755), Astyanax mexicanus (ENSAMXT00000054253)

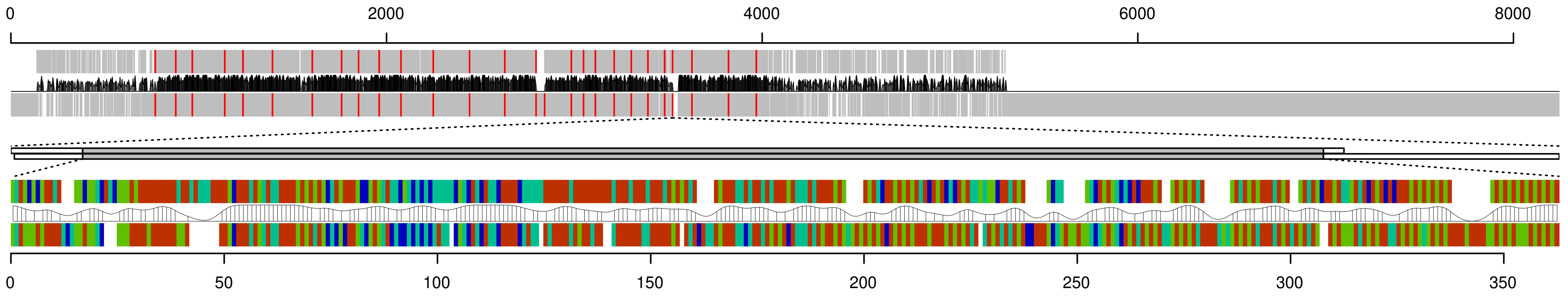

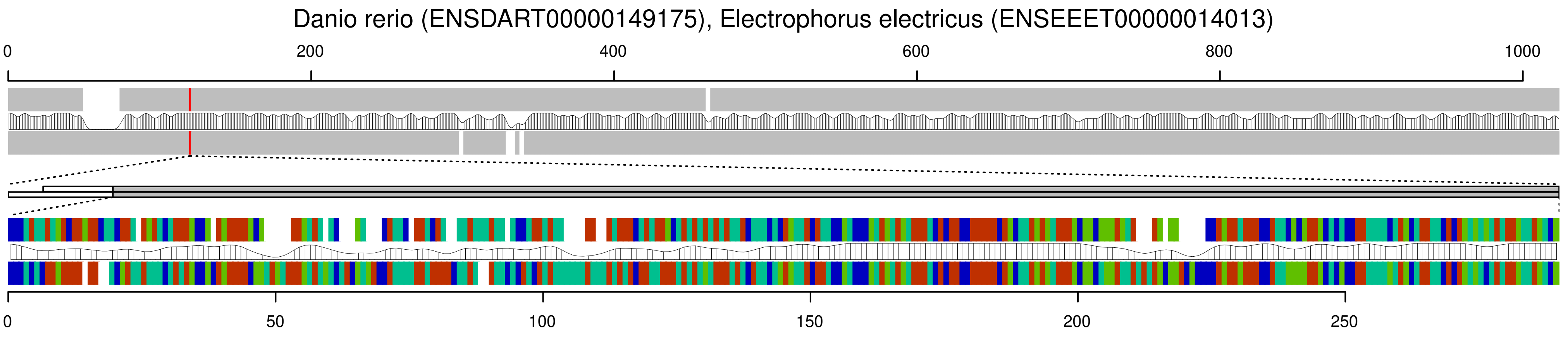
Danio rerio (ENSDART00000149175), Electrophorus electricus (ENSEEET00000014013)
0 200 400 600 800 1000
0 50 100 150 200 250

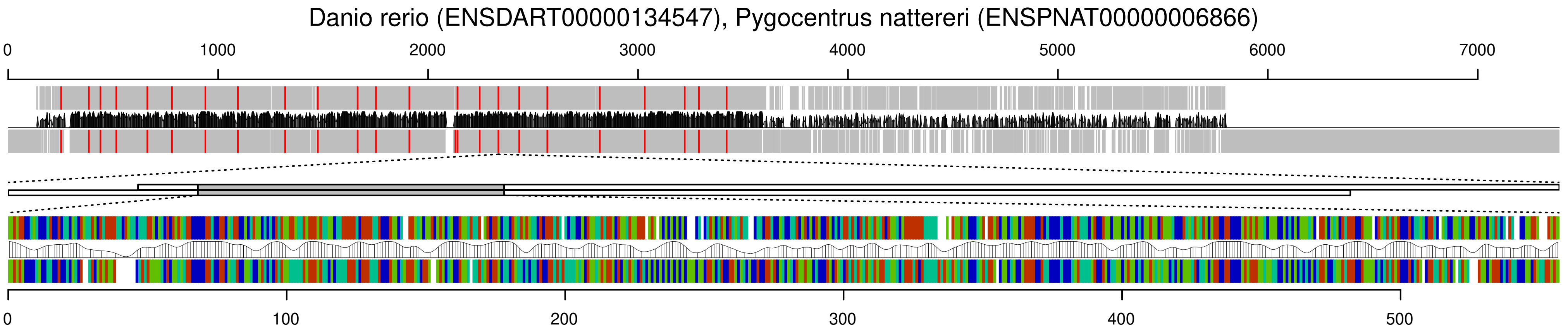
Danio rerio (ENSDART00000134547), Pygocentrus nattereri (ENSPNAT00000006866)
0 1000 2000 3000 4000 5000 6000 7000
0 100 200 300 400 500

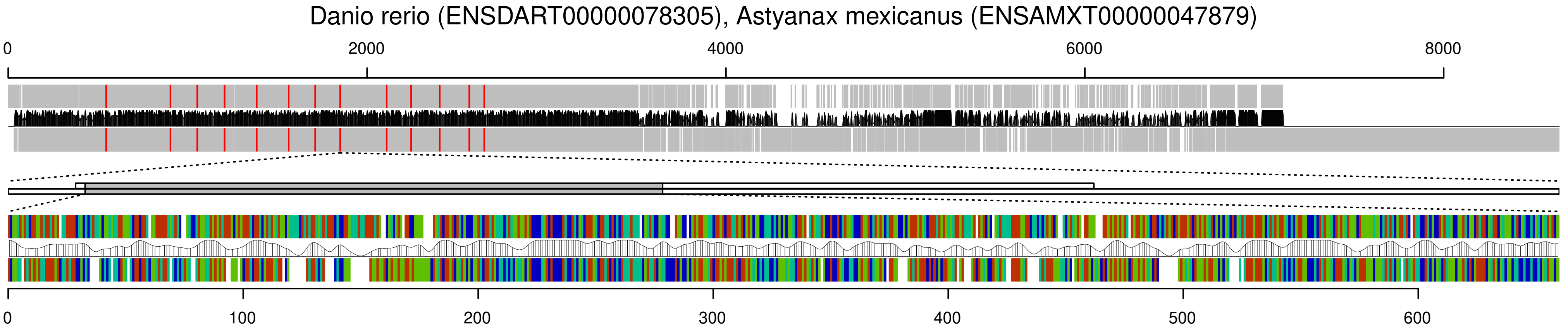
Danio rerio (ENSDART00000078305), Astyanax mexicanus (ENSAMXT00000047879)
0 2000 4000 6000 8000
0 100 200 300 400 500 600

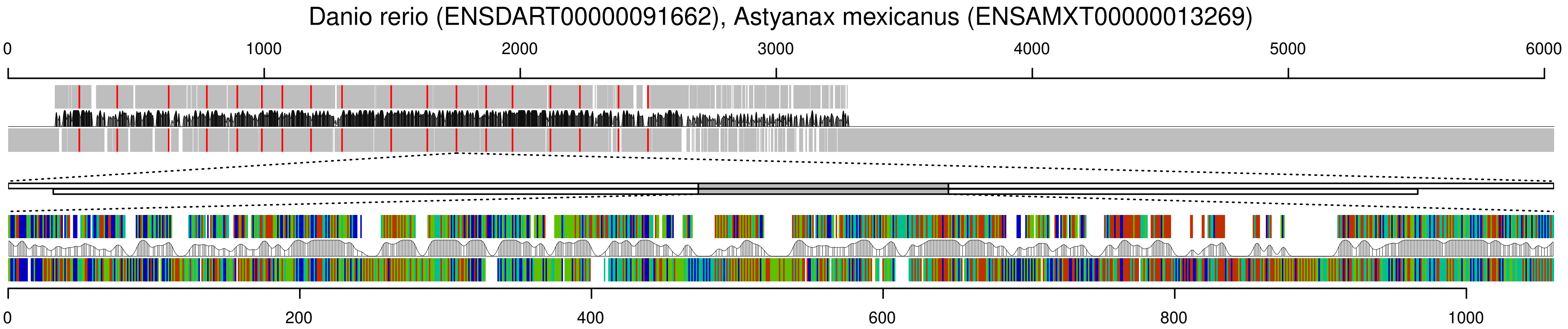
Danio rerio (ENSDART00000091662), Astyanax mexicanus (ENSAMXT00000013269)
0 1000 2000 3000 4000 5000 6000
0 200 400 600 800 1000

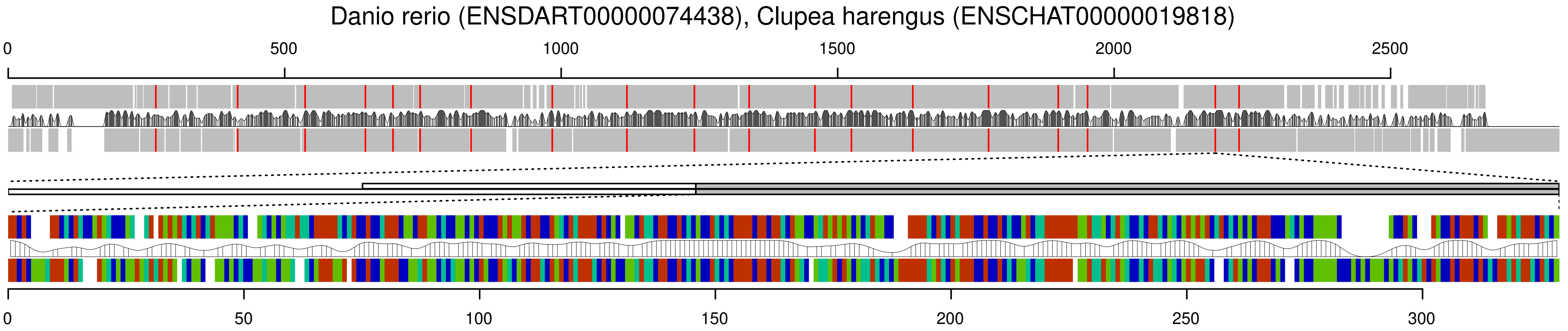
Danio rerio (ENSDART00000074438), Clupea harengus (ENSCHAT00000019818)
0 500 1000 1500 2000 2500
0 50 100 150 200 250 300

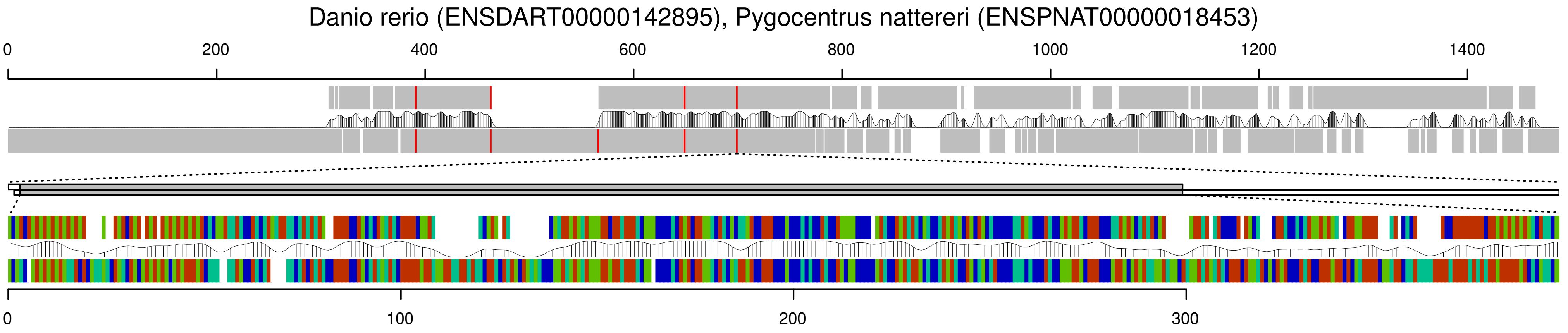
Danio rerio (ENSDART00000142895), Pygocentrus nattereri (ENSPNAT00000018453)
0 200 400 600 800 1000 1200 1400
0 100 200 300

Danio rerio (ENSDART00000132602), Clupea harengus (ENSCHAT00000018202)

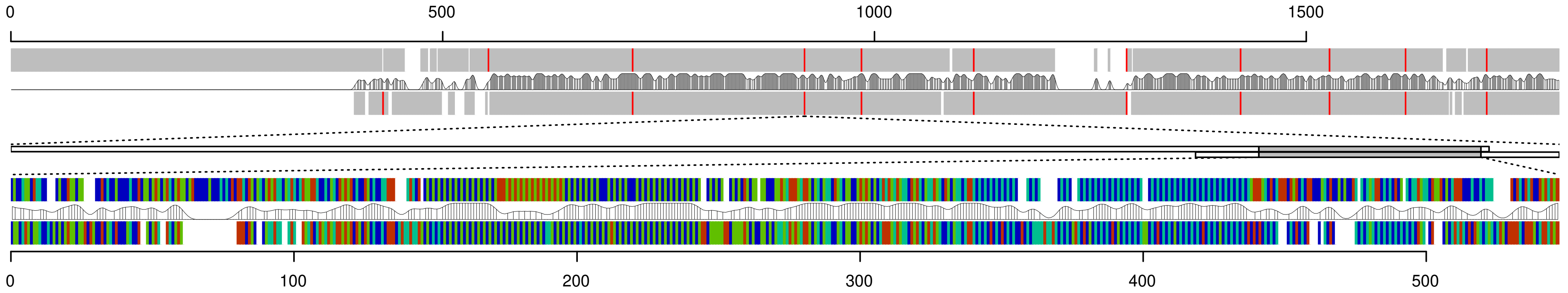

Danio rerio (ENSDART00000061659), Astyanax mexicanus (ENSAMXT00000017498)

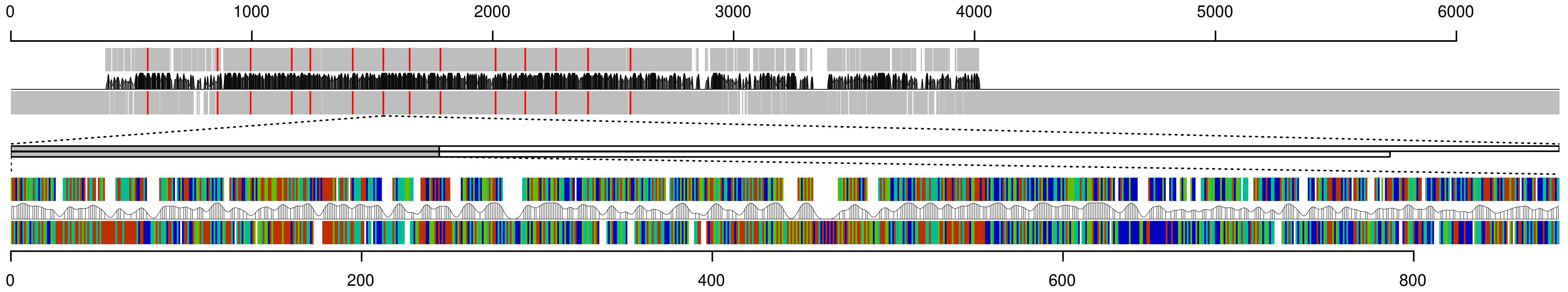

Danio rerio (ENSDART00000004333), Pygocentrus nattereri (ENSPNAT00000006580)

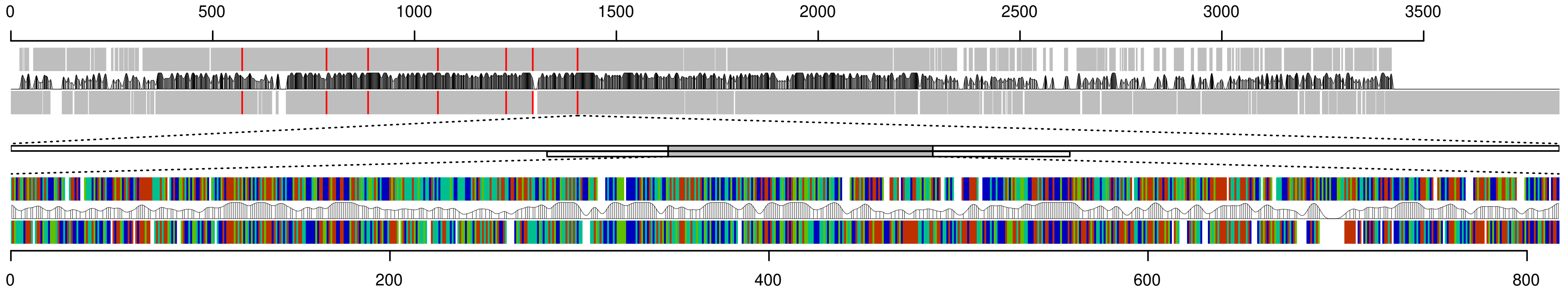

Danio rerio (ENSDART00000177399), Astyanax mexicanus (ENSAMXT00000000957)

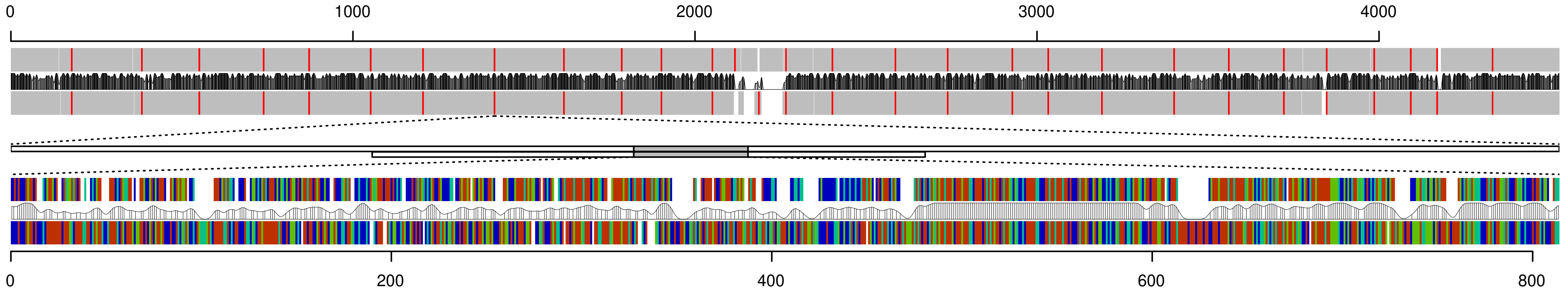

Danio rerio (ENSDART00000023238), Pygocentrus nattereri (ENSPNAT00000006262)

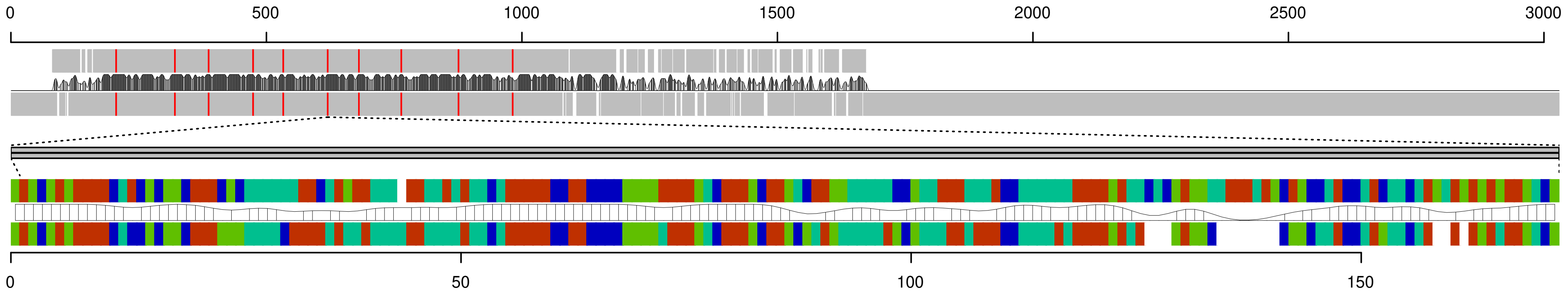

Danio rerio (ENSDART00000134809), Pygocentrus nattereri (ENSPNAT00000013103)

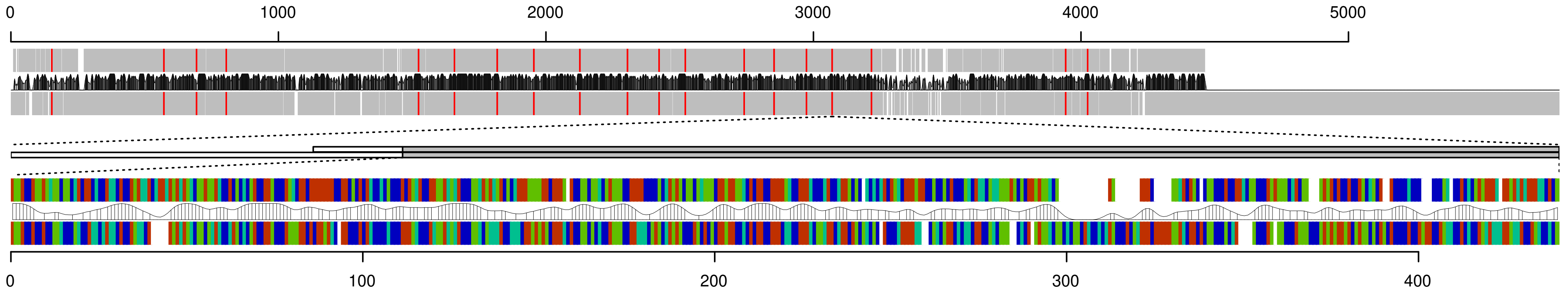

Danio rerio (ENSDART00000057644), Hucho hucho (ENSHHUT00000073433)

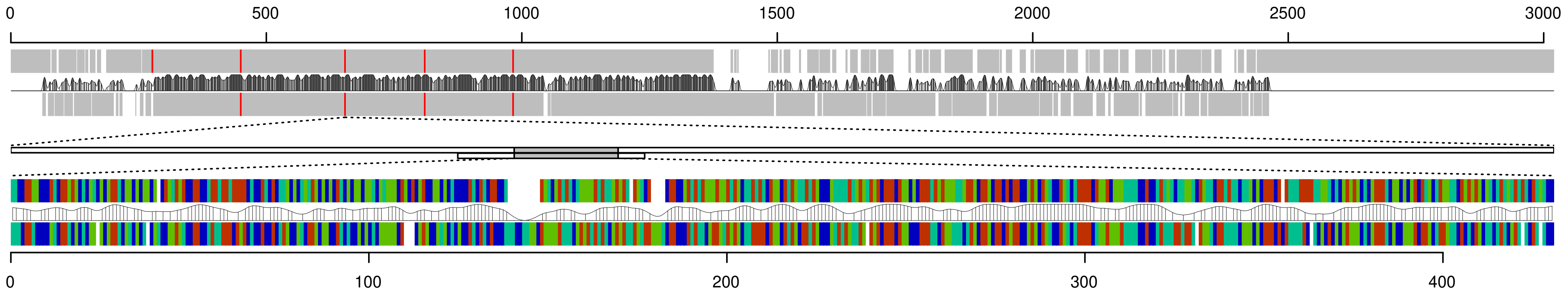

Danio rerio (ENSDART00000012194), Clupea harengus (ENSCHAT00000064750)

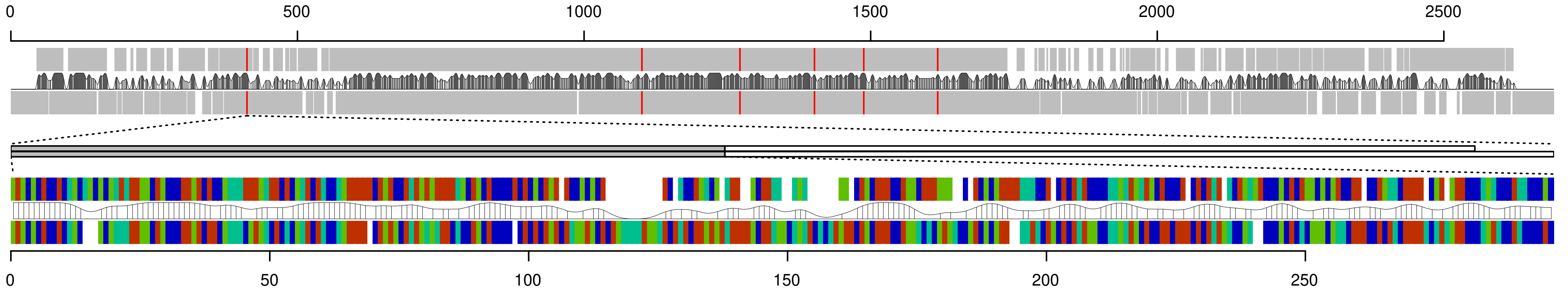

Danio rerio (ENSDART00000144186), Astyanax mexicanus (ENSAMXT00000002919)

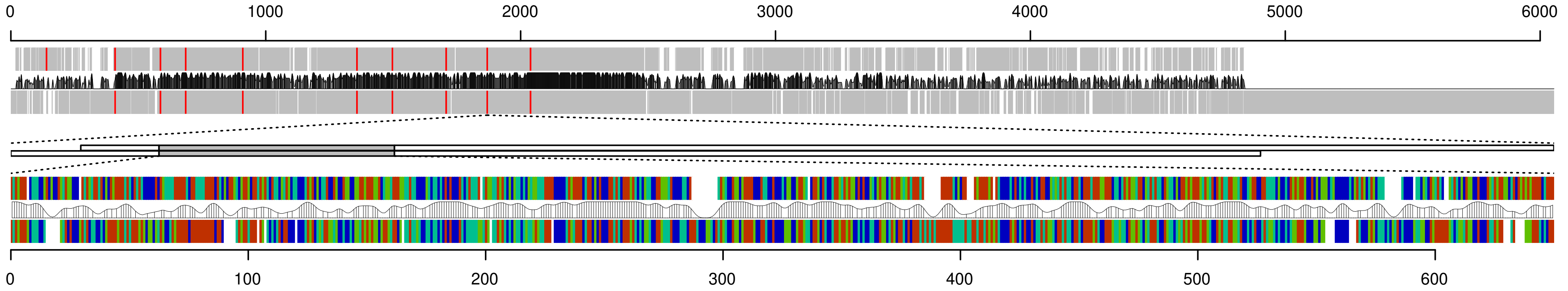

Danio rerio (ENSDART00000149478), Hucho hucho (ENSHHUT00000091607)

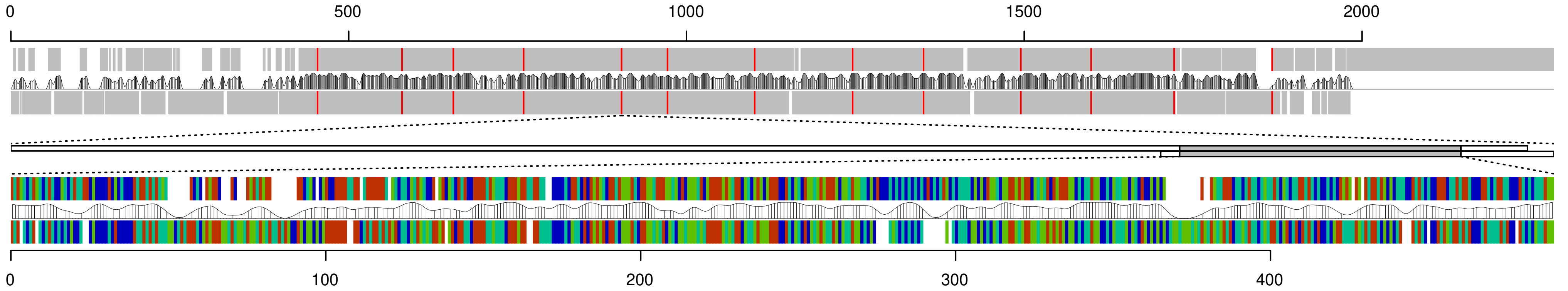

Danio rerio (ENSDART00000082664), Gadus morhua (ENSGMOT00000002098)

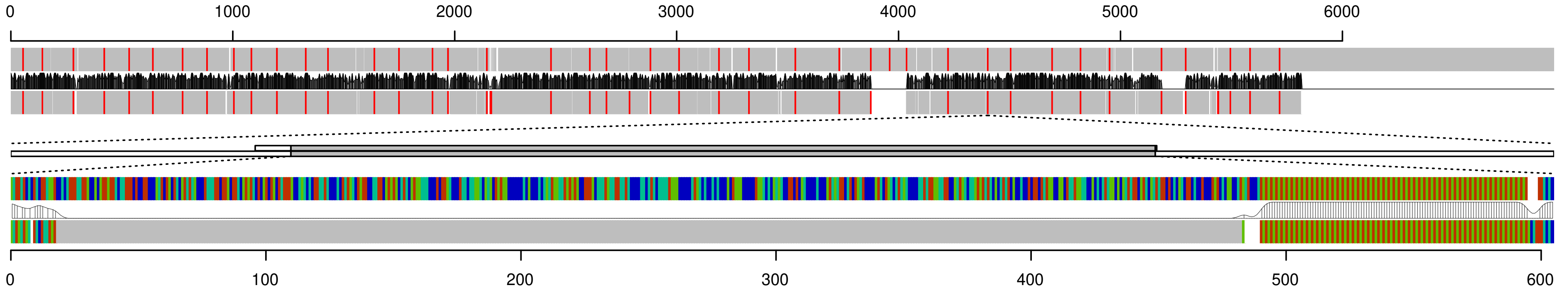

Danio rerio (ENSDART00000102304), Ictalurus punctatus (ENSIPUT00000025505)

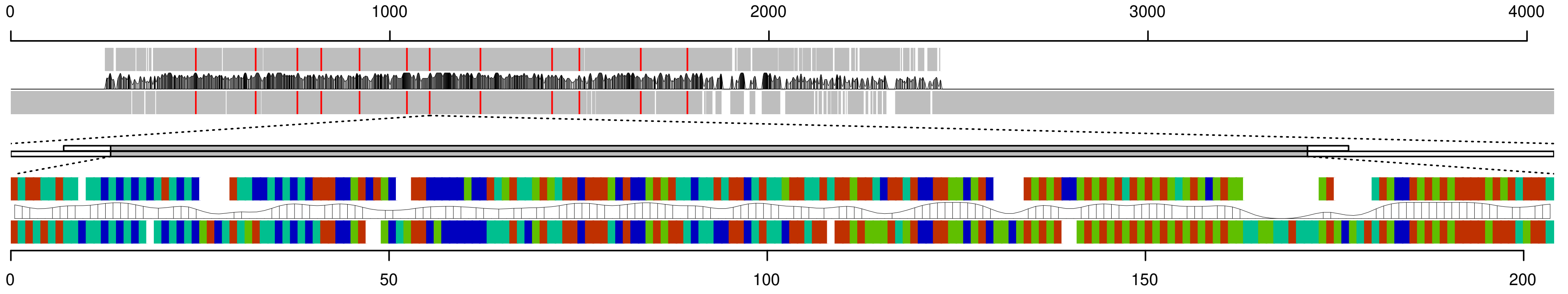

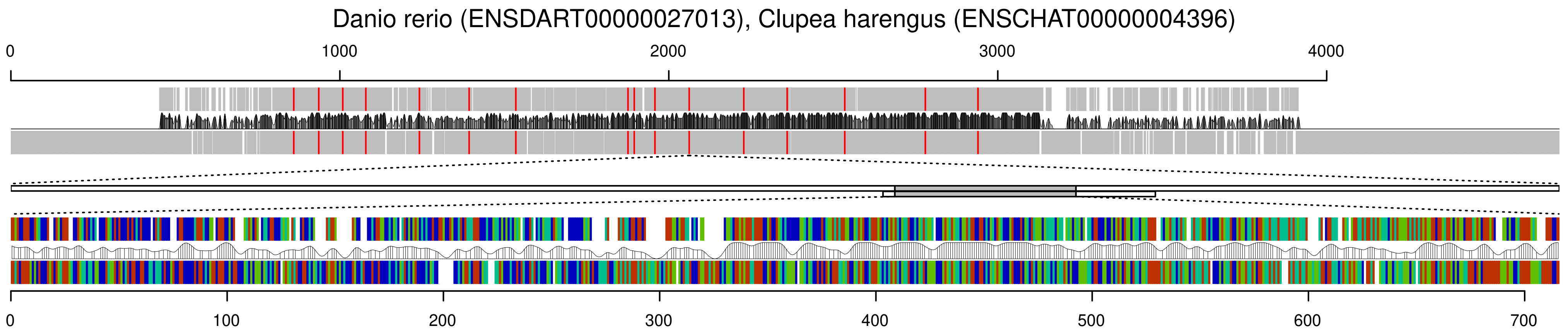
Danio rerio (ENSDART00000027013), Clupea harengus (ENSCHAT00000004396)
0 1000 2000 3000 4000
0 100 200 300 400 500 600 700

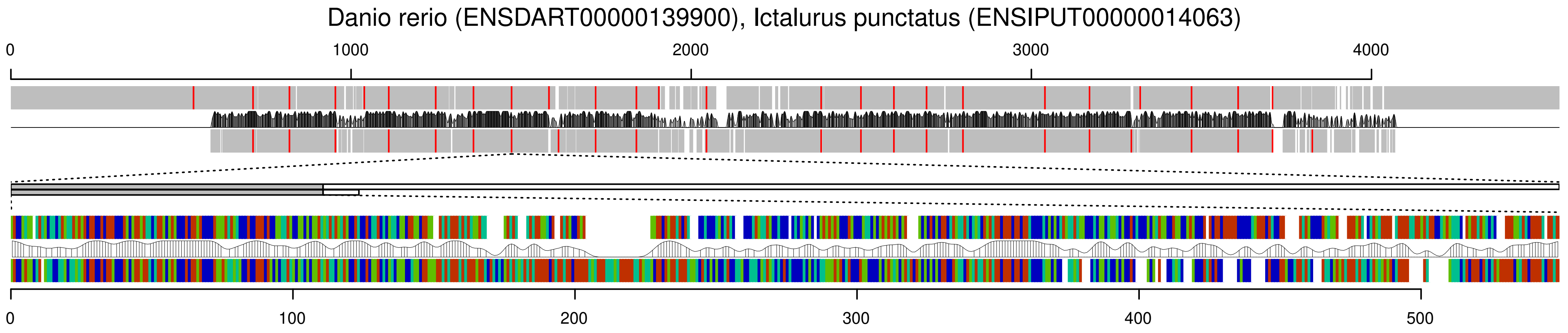
Danio rerio (ENSDART00000139900), Ictalurus punctatus (ENSIPUT00000014063)
0 1000 2000 3000 4000
0 100 200 300 400 500

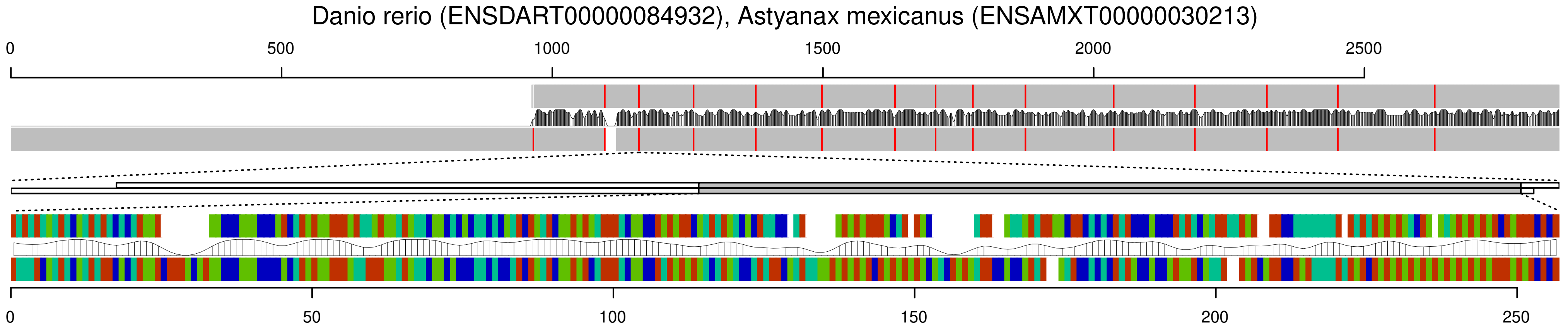
Danio rerio (ENSDART00000084932), Astyanax mexicanus (ENSAMXT00000030213)
0 500 1000 1500 2000 2500
0 50 100 150 200 250

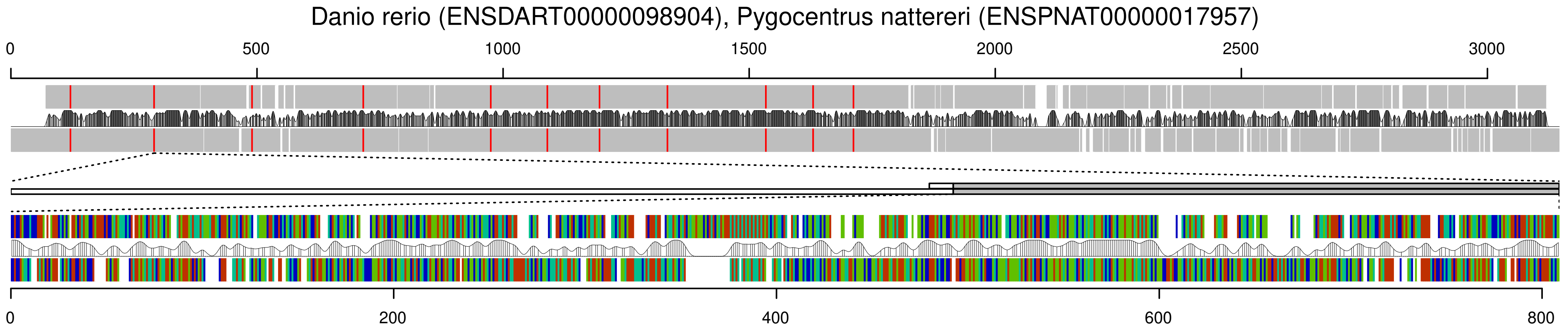
Danio rerio (ENSDART00000098904), Pygocentrus nattereri (ENSPNAT00000017957)
0 500 1000 1500 2000 2500 3000
0 200 400 600 800

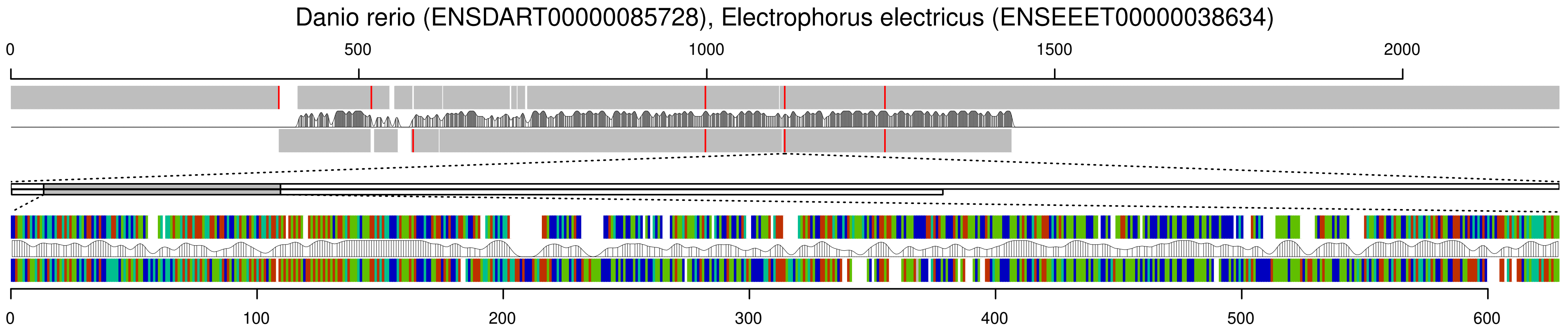
Danio rerio (ENSDART00000085728), Electrophorus electricus (ENSEEET00000038634)
0 500 1000 1500 2000
0 100 200 300 400 500 600

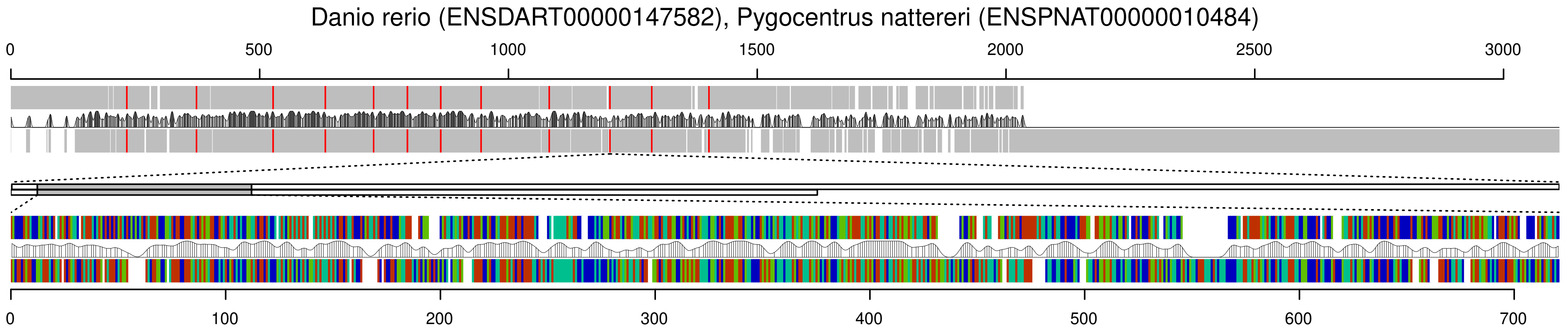
Danio rerio (ENSDART00000147582), Pygocentrus nattereri (ENSPNAT00000010484)
0 500 1000 1500 2000 2500 3000
0 100 200 300 400 500 600 700

Danio rerio (ENSDART00000051139), Pygocentrus nattereri (ENSPNAT00000024748)

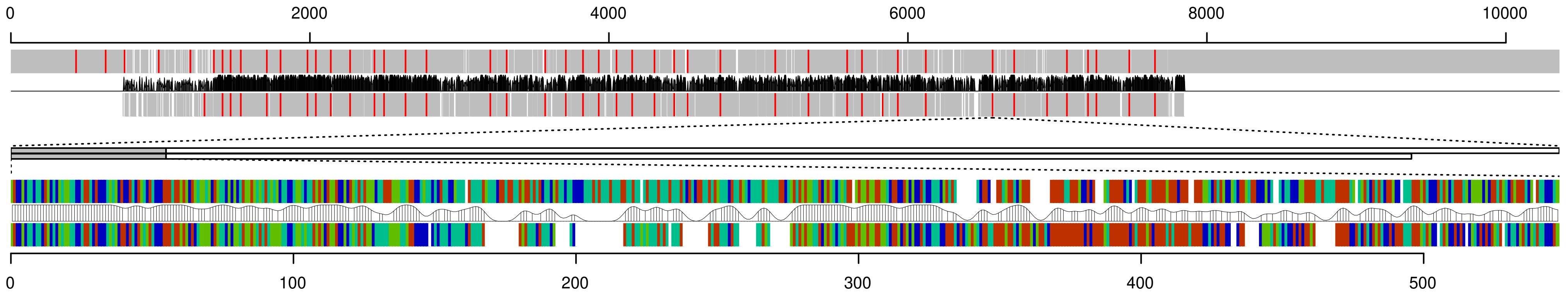

Danio rerio (ENSDART00000138687), Clupea harengus (ENSCHAT00000034760)

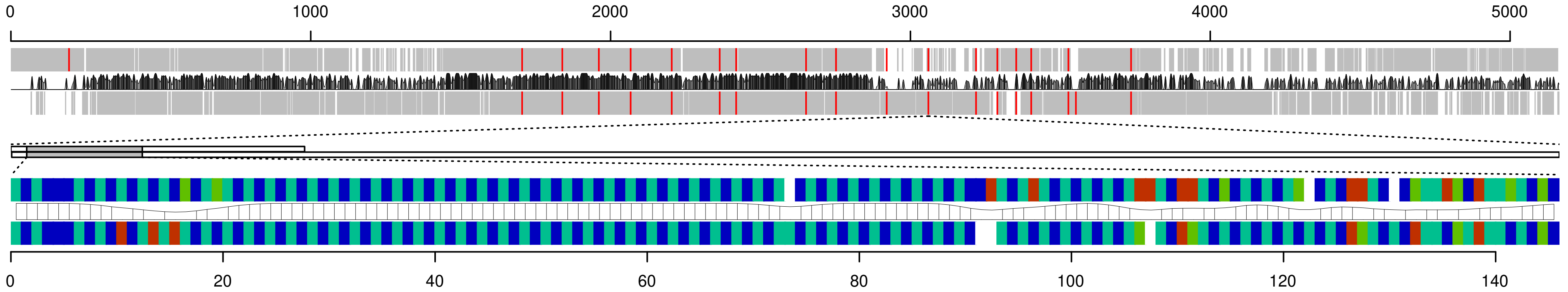

Danio rerio (ENSDART00000149957), Scleropages formosus (ENSSFOT00015020778)

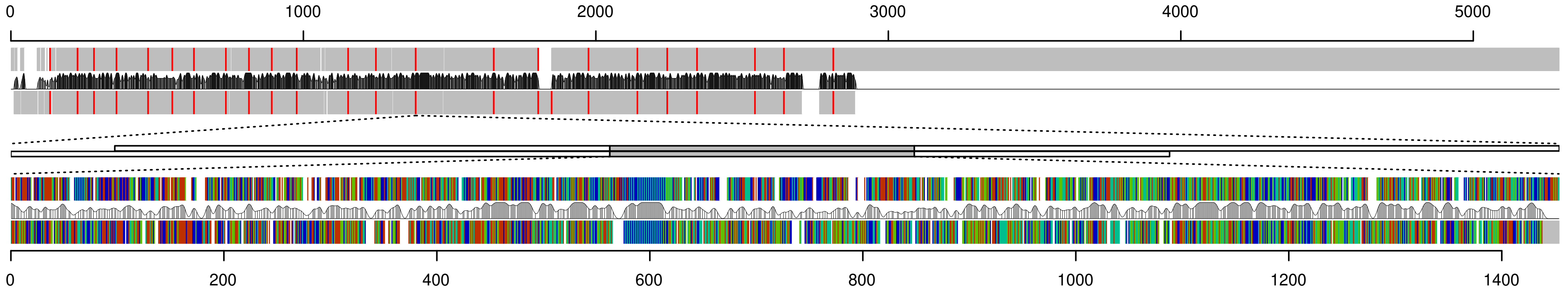

Danio rerio (ENSDART00000153140), Pygocentrus nattereri (ENSPNAT00000009521)

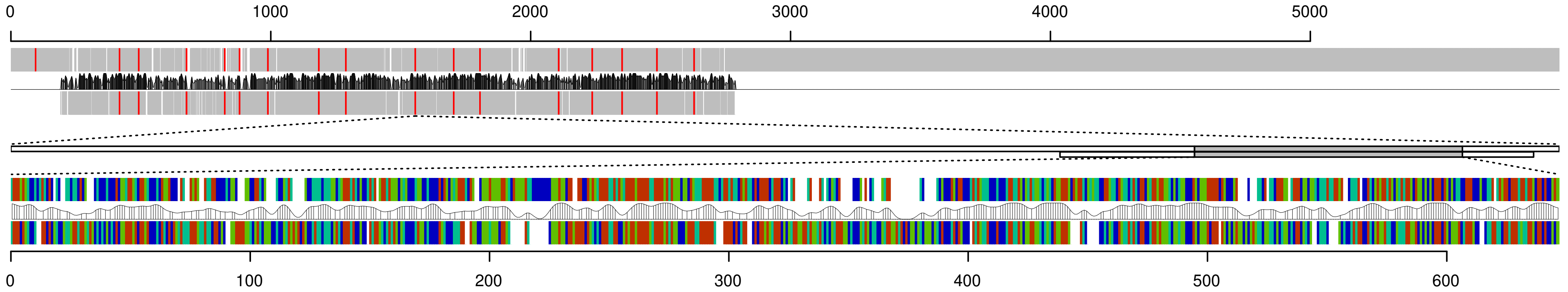

Danio rerio (ENSDART00000055395), Astyanax mexicanus (ENSAMXT00000014922)

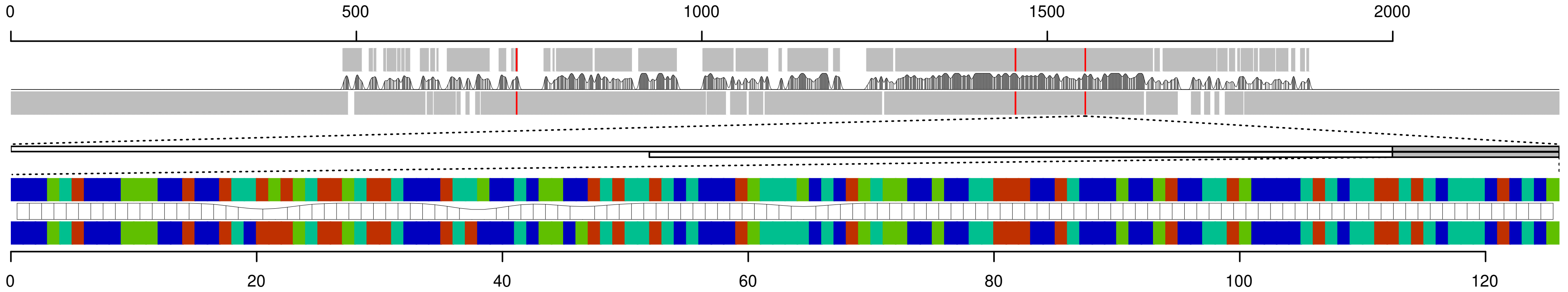

Danio rerio (ENSDART00000011447), Astyanax mexicanus (ENSAMXT00000055004)

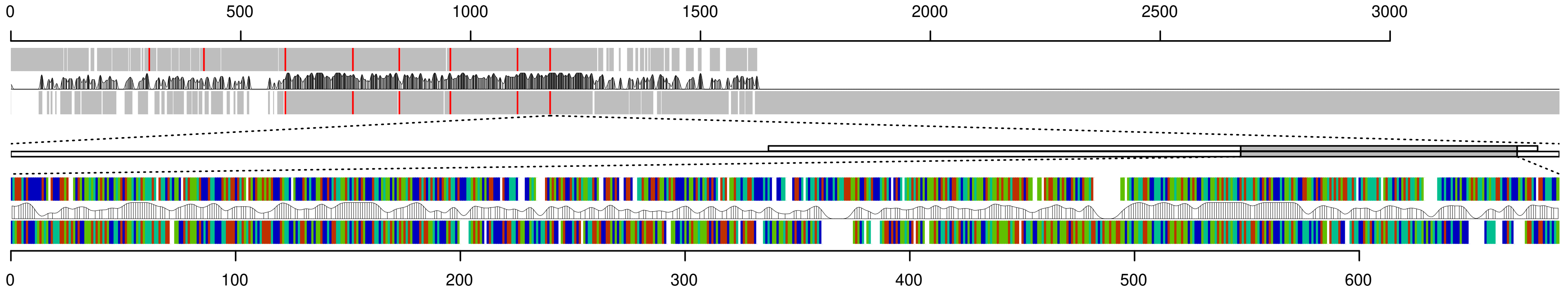

Supplement: Supplementary file 6 — Transcript and intron alignments for points in Fig. S14. Each panel shows the maximally scoring alignment between D. rerio and teleost intron orthologues (lower) and transcript alignment (upper) used to establish the intron orthology. Grey, white and red parts indicate aligned exonic sequence, gaps and positions of intron meta-characters respectively. Colours in intron alignment represent bases (A blue, C cyan, G green, T brown, N grey, gap white). Curves lying between sequence representations show a normal kernel density smoothed estimate of local similarity (9 bp window, standard deviation two); vertical lines indicate matches. Region between exon and intron alignments indicates the location of the maximally scoring alignment in the introns. Upper sequence D. rerio. Files 6–10 and 11–15 contain alignments to teleost and mammalian sequences respectively. Each file corresponds to one panel in Fig. S17 and to one specific teleost size class: Files 6,11: long (E,J), 7,12: medium (D,I), 8,13: short.2 (C,H), 9,14 short (B, G) and 10,15 ctl (A,F). [file 12864_2022_8760_MOESM6_ESM.zip › 12864_2022_8760_MOESM8_ESM.pdf]
